# Global, regional, and national causes of under-5 mortality in 2000–19: an updated systematic analysis with implications for the Sustainable Development Goals

**DOI:** 10.1016/S2352-4642(21)00311-4

**Published:** 2022-02

**Authors:** Jamie Perin, Amy Mulick, Diana Yeung, Francisco Villavicencio, Gerard Lopez, Kathleen L Strong, David Prieto-Merino, Simon Cousens, Robert E Black, Li Liu

**Affiliations:** a Department of International Health, Johns Hopkins University, Baltimore, MD, USA; b Department of Population, Family, and Reproductive Health, Johns Hopkins University, Baltimore, MD, USA; c Epidemiology and Population Health, London School of Tropical Medicine & Hygiene, London, UK; d Department of Maternal, Newborn, Child and Adolescent Health and Ageing, World Health Organization, Geneva, Switzerland

## Abstract

**Background:**

Causes of mortality are a crucial input for health systems for identifying appropriate interventions for child survival. We present an updated series of cause-specific mortality for neonates and children younger than 5 years from 2000 to 2019.

**Methods:**

We updated cause-specific mortality estimates for neonates and children aged 1–59 months, stratified by level (low, moderate, or high) of mortality. We made a substantial change in the statistical methods used for previous estimates, transitioning to a Bayesian framework that includes a structure to account for unreported causes in verbal autopsy studies. We also used systematic covariate selection in the multinomial framework, gave more weight to nationally representative verbal autopsy studies using a random effects model, and included mortality due to tuberculosis.

**Findings:**

In 2019, there were 5·30 million deaths (95% uncertainty range 4·92–5·68) among children younger than 5 years, primarily due to preterm birth complications (17·7%, 16·1–19·5), lower respiratory infections (13·9%, 12·0–15·1), intrapartum-related events (11·6%, 10·6–12·5), and diarrhoea (9·1%, 7·9–9·9), with 49·2% (47·3–51·9) due to infectious causes. Vaccine-preventable deaths, such as for lower respiratory infections, meningitis, and measles, constituted 21·7% (20·4–25·6) of under-5 deaths, and many other causes, such as diarrhoea, were preventable with low-cost interventions. Under-5 mortality has declined substantially since 2000, primarily because of a decrease in mortality due to lower respiratory infections, diarrhoea, preterm birth complications, intrapartum-related events, malaria, and measles. There is considerable variation in the extent and trends in cause-specific mortality across regions and for different strata of all-cause under-5 mortality.

**Interpretation:**

Progress is needed to improve child health and end preventable deaths among children younger than 5 years. Countries should strategize how to reduce mortality among this age group using interventions that are relevant to their specific causes of death.

**Funding:**

Bill & Melinda Gates Foundation; WHO.

## Introduction

In 2019, an estimated 5·30 million children died before their fifth birthday, a substantial reduction from the estimated 9·92 million deaths of children younger than 5 years in 2000. This decline reflects efforts across multiple platforms by an array of organisations and governments in the period of the Millennium Development Goals (MDGs) in 2000–2015,^1^ and in the subsequent period of the Sustainable Development Goals (SDGs). This reduction represents a decline in under-5 mortality rate from 75 deaths per 1000 livebirths in 2000, to 38 deaths per 1000 livebirths in 2019. However, despite such an extraordinary reduction, the global under-5 mortality rate is still well above the target of 25 deaths per 1000 livebirths as defined by SDG 3.2.^2^

To identify appropriate targets and interventions to address mortality in children younger than 5 years, a broad understanding of the causes of mortality among this age group is needed, both globally and in individual countries, many of which do not have functioning vital registration systems to directly report underlying causes of death. Such estimates are likely to increase in importance as mortality rates decline and targets for excess mortality among children become less conspicuous. In this study, we provide an updated series of estimates for cause-specific mortality among neonates aged 0–27 days and children aged 1–59 months,^3^ using additional primary input data (ie, verbal autopsy and vital registration data) on causes of death and a major update in the statistical methods. With these improvements, we present a complete time series for cause-specific mortality from 2000 to 2019 for children younger than 5 years and discuss progress on selected causes of death towards SDG 3.2.

**Research in context**

**Evidence before this study**

This research group has been actively developing systematic cause of death estimates for neonates and children younger than 5 years since 2003. The group was previously known as the WHO and UNICEF's Child Health Epidemiology Reference Group, and as of 2013 is the Maternal and Child Epidemiology Estimation. Our estimates for 2000–16 were published and incorporated into the WHO Global Health Estimates, with an update for 2000–17 made available as a white paper. In this Article, we present new estimates based on updated modelling methods augmented by new data from a systematic review. We identified child cause-of-death studies published between Jan 1, 2015, and Dec 31, 2017 in PubMed, Scopus, Web of Science, Embase, Cochrane, Global Health Ovid, Global Index Medicus (including LILACS, MSEAR, WPRIM, IMEMR, WHOLIS, and AIM), IndMed, PAHO, and Popline using our previous search strategies and study inclusion and exclusion criteria. Other research groups have presented similar studies of causes of death among children younger than age 5 years.

**Added value of this study**

We have extended our cause-specific mortality estimates up to 2019, including data from a systematic review, and updated methods to make covariate selection more systematic and to flexibly account for unreported causes of death. We have also used random effects to give more weight to nationally representative verbal autopsy data.

**Implications of all the available evidence**

Cause-specific mortality estimates can inform policies and programmes to reduce mortality among neonates and children younger than 5 years. Future research should concentrate on estimating cause-specific mortality in subnational areas, strengthening vital and sample registration systems, and better accounting for the misclassification and associated uncertainty in verbal autopsy algorithms for cause assignment. Continued investment and refinement in these methods will result in improved and reliable cause-specific mortality estimates for children under age 5 years, which can support programmes to reach Sustainable Development Goal 3.2 by 2030.

## Methods

### Updates on estimation methods

This update includes a substantial change in the statistical methods. Previously, for countries without high coverage vital registration systems, we estimated cause-specific mortality for neonates and children aged 1–59 months stratified by level of (ie, low, moderate, or high) mortality in a frequentist framework.^3^ This approach meant that covariate selection was necessarily conducted for each cause separately because of the large number of covariate combinations in the multinomial setting. Additionally, this frequentist framework made the estimation of random effects computationally difficult, although random effects are known to increase the reliability of predictions.^4^

For this update, we used Bayesian inference and implemented the least absolute shrinkage and selection operator (LASSO) for covariate selection in a mixed-effects multinomial model.^5^ We used a multinomial likelihood by which the log odds for each cause, relative to a base cause, were estimated on a conditional set of covariates; the effect of these covariates (aside from intercepts) was penalised by LASSO. Cross validation determined the appropriate amount of penalisation on multinomial regression coefficients for high and low mortality strata within each age group, in which out-of-sample cross validation error was based on the difference between empirical and predicted causes, with causes predicted by both fixed and random effects. When cross validation did not identify a clear best amount of penalisation, higher degrees of penalisation were favoured to avoid overfitting.

These Bayesian models also incorporated study-level random effects, which allowed nationally representative verbal autopsy studies to have more weight in country estimates than in previous methods. We also developed an extension of the Bayesian framework, including a misclassification matrix between true and reported causes of death specified within study, so that we could model the same set of true causes across verbal autopsy studies reporting different causes.^5^ This misclassification matrix is not estimated but determined by the causes reported and specific to each study. This matrix translates between the observed causes and the causes of interest, which are generally non-zero but not necessarily reported by all studies in our systematic review. In countries where the mortality among children younger than 5 years declined substantially over the period of interest, we are also now using model averaging to ensure a smooth transition in the distribution of causes between the high and low mortality eras.

This new series of cause-specific mortality estimates covers the period from 2000 to 2019. Our database of covariates for projecting causes of mortality^3^ has also been updated and extrapolated for countries for 2000–19. Additionally, we have updated the systematic review for data inputs to our models for high mortality countries to include verbal autopsy studies as recent as 2017 for both age groups. Together with the standard cause categories that we previously estimated, we also now include mortality among children younger than 5 years due to tuberculosis, as reported by WHO Global Tuberculosis Programme.

### Country estimates

In countries without high-quality vital registration and high mortality (≥20 deaths per 1000 livebirths for neonates; ≥35 deaths per 1000 livebirths for children aged 1–59 months),^3^ causes were predicted using a Bayesian LASSO for extrapolating cause-specific mortality fractions from verbal autopsy studies identified in a systematic review (table 1, appendix pp 6–28). In countries with low mortality (<10 deaths per 1000 livebirths for neonates; <25 deaths per 1000 livebirths for children aged 1–59 months) but without high-quality vital registration,^3^ causes were estimated using a Bayesian LASSO for extrapolating cause-specific mortality fractions from high-quality vital registration data.

**Table 1:** New and total input data by estimation method

	**New input data**			**Total input data**		
	Data points	Deaths	Countries	Data points	Deaths	Countries
**Neonates**						
High-quality vital registration	404 (28%)	658 836 (21%)	73	1460	3 126 336	73
Low mortality model	413 (17%)	328 023 (14%)	70	2417	2 366 534	72
Moderate or high mortality model	119 (49%)	90 126 (47%)	17	243	190 245	43
**Children aged 1–59 months**						
High-quality vital registration	416 (27%)	449 854 (18%)	76	1520	2 493 617	76
Low mortality model	299 (18%)	308 753 (19%)	73	1663	1 646 909	76
Moderate or high mortality model	302 (58%)	104 170 (22%)	19	520	476 494	46

Data are n (% of total input data).

Countries or specific years within countries with moderate mortality (10–20 deaths per 1000 livebirths for neonates; 25–35 deaths per 1000 livebirths for children aged 1–59 months) were estimated by averaging the high-mortality and low-mortality models proportionately relative to their mortality rate. Details for high-mortality and low-mortality models, including cross validation results and coefficient estimates, are included in the appendix (pp 32–41). Sensitivity analyses that hold constant the degree of penalisation and vary the effect given to nationally representative verbal autopsy studies by changing the limit of the standard deviation for the random effects are shown in the appendix (pp 44–55). We restricted the influence of random effects to be small in all models for low-mortality countries, where verbal autopsy studies are not typically conducted and nationally representative causes of death are generally not measured. For high-mortality countries, we restricted the SD of random effects to be 0·14 or less, corresponding to a change in odds for a specific cause within 30% for most countries. For all estimates, model convergence was assessed by examining the parameter traces and the Gelman–Rubin statistics, which were less than 1·1 for all parameters. The plot of the iterative parameter traces is included in the appendix (pp 58–90).

Causes of death in countries with high-functioning vital registration systems, defined by previously reported criteria,^3^ are estimated using empirical data and the International Classification of Diseases 10 mortality coding (appendix p 32). Causes for countries with high-functioning vital registration are not modelled. Instead, reported causes are grouped according to previously reported classifications for each specific year that has available data on vital registration.^3^ For years that do not have available data on vital registration causes of death, the proportion of mortality is interpolated (between reporting periods) or extrapolated for recent years (after 2018) beyond reporting periods. Along with these estimates for countries with high-functioning vital registration, empirical estimates for causes of death are also derived for China from the Chinese National Maternal and Child Health Surveillance System.^6^ For all countries without high-quality vital registration, HIV deaths were estimated using data from UNAIDS. For all countries, the proportion of mortality by cause is used from these empirical and modelled estimates and applied to the mortality envelope from the UN Inter-agency group for Child Mortality Estimation.^7^

### Neonates

The proportion of neonatal mortality due to tetanus, lower respiratory infections, preterm birth complications, intrapartum-related events, sepsis, congenital abnormalities, and diarrhoea were estimated. For low-mortality modelled countries, we chose preterm birth complications as the reference cause in the multinomial Bayesian model because of its high burden. Additionally, in countries with low neonatal mortality, diarrhoea deaths among neonates were assumed to be zero, and tetanus was estimated using data from the WHO Immunization Programme. In countries with moderate-to-high neonatal mortality, we chose intrapartum-related events as the reference cause in the multinomial Bayesian model because we expected this to have a large burden. For all countries with modelled estimates, meningitis was estimated as a proportion of sepsis deaths on the basis of estimates from the 2019 Global Burden of Disease (GBD) study.^8^

### Children aged 1–59 months

The proportion of child mortality due to lower respiratory infections, diarrhoea, meningitis, injury, malaria, congenital abnormalities, and perinatal causes (ie, premature birth and intrapartum-related events) were estimated for children aged 1–59 months. For countries with both low and moderate-to-high mortality, we used lower respiratory infections as the reference cause in the multinomial Bayesian model for its high burden. For moderate-to-high mortality countries, the relative proportions from GBD estimates^8^ were used to estimate the fractions of mortality from perinatal causes due to preterm birth complications and intrapartum-related events. For countries outside sub-Saharan Africa, regardless of mortality strata, the number of deaths due to malaria were those reported in the 2020 WHO World Malaria Report.^9^ For countries with high malaria endemicity (in sub-Saharan Africa) the number of deaths due to malaria is estimated by the high-mortality model. The implementation of the *Haemophilus influenzae* type b vaccine (Hib) in 2000, pneumococcal conjugate vaccine (PCV) after 2008, and rotavirus vaccine in 2012 was not fully reflected in data from our systematic review; therefore, we have adjusted mortality due to lower respiratory infections and meningitis for areas with coverage of Hib and PCV and diarrhoea mortality for rotavirus vaccine coverage as in previous estimates.^3^

### Measles, tuberculosis, and crisis deaths

For all countries with modelled estimates, measles was estimated by the WHO Immunization, Vaccinations, and Biologicals Programme. Estimated pulmonary tuberculosis deaths from the WHO Global Tuberculosis Programme^10^ were assumed as a fraction of mortality due to lower respiratory infections, whereas extrapulmonary tuberculosis deaths were assumed as a fraction of other communicable causes of deaths estimated from the multinomial Bayesian LASSO. Extrapulmonary tuberculosis among children can be under-recognised in some areas;^11^ therefore, in the case that these deaths exceeded the fraction of other communicable causes, the excess was reassigned as a fraction of deaths due to lower respiratory infections.

We also accounted for deaths that were related to crises among children younger than 5 years. Crises are defined as events isolated to less than 5 years with more than ten deaths of children younger than 5 years, constituting more than 10% of overall under-5 mortality, and having a crisis-related under-5 mortality rate of 200 deaths per 1000 livebirths, as defined by UN-IGME.^7^, ^12^ The number of deaths estimated by UN-IGME as being due to crisis were attributed to specific causes depending on the nature of each crisis. Crisis deaths related to natural disasters, such as tsunamis and earthquakes, were attributed to injury and constituted most crisis deaths, as in previous estimates.^3^ However, deaths were attributed to malnutrition (other group 1) for the 2015–19 conflict in Yemen,^13^ to the Zika virus (congenital) for the 2016 Zika epidemic in Brazil,^14^ and to all causes pro rata for the 2015–19 political unrest in Venezuela.^15^

### Estimates reporting

We used the UNICEF global regions definitions to analyse the results. We present the average annual rate of reduction by underlying cause during the MDG period (2000–15) and the first few years of the SDG period (2015–19) for a country in each mortality stratum having a large number of under-5 deaths to guide child survival strategy in the remaining years of the SDG era, assuming that the fraction of all-cause mortality would be the same when the SDG target all-cause rate was reached. We did not account for uncertainty in this examination.

These estimates have been reviewed and approved by representatives of all 194 WHO Member States through a process of country consultation and are released as an official series of cause-specific mortality reported by WHO. We have included a GATHER checklist for these estimates (appendix pp 92–93) to promote transparency and replicability of global health estimates.^16^

All analyses were conducted in R (version 4.0.3) with Bayesian models estimated in JAGS (version 4.2.0).

### Uncertainty reporting

We estimated uncertainty for cause-specific proportions and numbers of deaths on the basis of the uncertainty of primary inputs. Uncertainty for modelled proportions was based on the posterior distribution of multinomial regression parameters and the posterior distribution of random effect estimates. For countries with nationally representative verbal autopsy studies, only these studies' random effects contributed to random effect uncertainty.^5^ Uncertainty for the number of deaths due to measles, tuberculosis, and HIV were included using a Monte Carlo simulation, which also accounted for variability in the effect estimates used for the post-hoc adjustment. We also accounted for uncertainty in the number of deaths due to all causes by taking samples from the posterior distribution of the estimated mortality envelopes by age.^7^

### Role of the funding source

The funder of the study had no role in study design, data collection, data analysis, data interpretation, or writing of the report. The corresponding author had full access to all the data in the study and had final responsibility for the decision to submit for publication.

## Results

**Figure 1:**
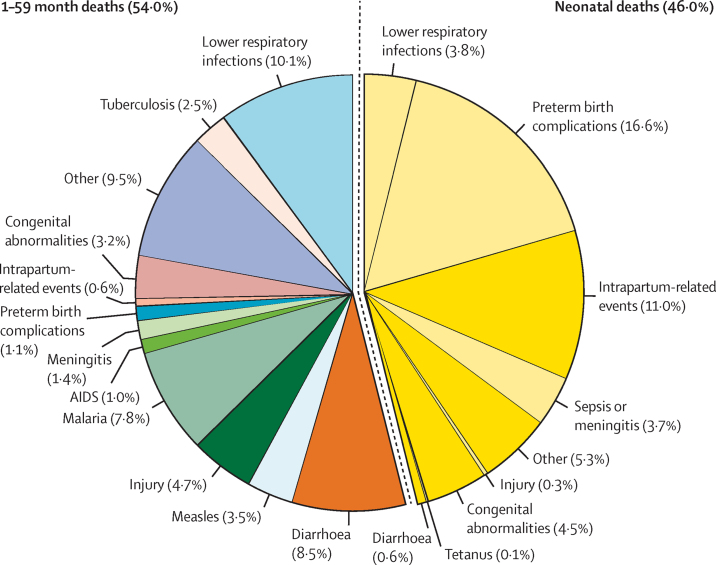
Global causes of under-5 deaths in 2019 Deaths of neonates (aged 0–27 days) are on the right-hand side and deaths of children aged 1–59 months are on the left-hand side.

In 2019, there were 5·30 million deaths (95% uncertainty range [UR] 4·92–5·68) among children younger than 5 years (ie, aged 0–59 months). The leading causes of death among children younger than 5 years were preterm birth complications (0·94 million [0·86–1·06]; 17·7% [16·1–19·5]), lower respiratory infections (0·74 million [0·62–0·84]; 13·9% [12·0–15·1]), and intrapartum-related events (0·62 million [0·57–0·70]; 11·6% [10·6–12·6]), altogether constituting 43·2% of under-5 deaths (figure 1; table 2). Of the 5·30 million deaths, 2·44 million (2·29–2·68; 46·0% [43·5–48·7]) were among neonates, and 2·61 million (2·43–2·92; 49·2% [47·3–51·9]) were due to infectious causes. Vaccine-preventable deaths, such as for lower respiratory infections, meningitis, and measles, constituted 21·7% (20·4–25·6) of under-5 deaths.

**Table 2:** Estimated number of deaths by cause and cause-specific mortality rate in 2019

	Estimated number in millions (95% UR*)	**Cause-specific mortality rate per 1000 live births (95% UR)**
**Children aged 0–59 months**		
Preterm birth complications	0·94 (0·86–1·06)	6·72 (6·15–7·60)
Lower respiratory infections†	0·74 (0·62–0·84)	5·30 (4·44–6·01)
Intrapartum-related events	0·62 (0·57–0·70)	4·41 (4·10–4·98)
Diarrhoea	0·48 (0·40–0·55)	3·47 (2·88–3·94)
Congenital abnormalities	0·40 (0·38–0·45)	2·89 (2·69–3·25)
Sepsis or meningitis	0·27 (0·24–0·31)	1·93 (1·71–2·19)
Other conditions	1·85 (1·74–2·11)	13·23 (12·47–15·12)
**Neonates aged 0–27 days**		
Preterm birth complications	0·88 (0·81–1·00)	6·31 (5·77–7·17)
Intrapartum-related events	0·58 (0·53–0·65)	4·18 (3·81–4·68)
Congenital abnormalities	0·24 (0·21–0·28)	1·70 (1·49–2·01)
Lower respiratory infections†	0·20 (0·17–0·26)	1·46 (1·23–1·83)
Sepsis or meningitis	0·20 (0·17–0·23)	1·41 (1·23–1·67)
Diarrhoea	0·03 (0·03–0·04)	0·24 (0·20–0·30)
Tetanus	0·01 (0·01–0·01)	0·06 (0·04–0·07)
Other conditions	0·30 (0·25–0·36)	2·13 (1·78–2·60)
**Children aged 1–59 months**		
Lower respiratory infections†	0·54 (0·43–0·61)	3·84 (3·05–4·40)
Diarrhoea	0·45 (0·37–0·51)	3·23 (2·65–3·68)
Malaria	0·42 (0·34–0·50)	2·98 (2·40–3·56)
Injuries	0·25 (0·23–0·29)	1·79 (1·61–2·05)
Measles	0·18 (0·12–0·46)	1·31 (0·83–3·32)
Congenital abnormalities	0·17 (0·15–0·19)	1·20 (1·10–1·37)
Meningitis	0·07 (0·06–0·08)	0·53 (0·43–0·59)
Preterm birth complications	0·06 (0·05–0·06)	0·41 (0·33–0·43)
AIDS	0·06 (0·04–0·08)	0·40 (0·29–0·58)
Intrapartum-related events	0·03 (0·04–0·05)	0·23 (0·25–0·33)
Other conditions	0·64 (0·55–0·71)	4·57 (3·91–5·07)

Other conditions among children aged 1–59 months included causes originated during the perinatal period, cancer, severe malnutrition, and other specified causes. UR=uncertainty range.

* UR is defined as the 2·5–97·5 centile.

† Lower respiratory infections were formerly referred to as pneumonia.

Among neonates aged 0–27 days, underlying causes of death were primarily preterm birth complications (0·88 million [95% UR 0·81–1·00]; 36·1% [UR 33·3–39·1]), intrapartum-related events (0·58 million [0·53–0·65]; 23·9% [22·3–25·3]), congenital abnormalities (0·24 million [0·21–0·28]; 9·7% [8·5–11·2]), and lower respiratory infections (0·20 million [0·17–0·26]; 8·3% [7·2–10·1]), altogether accounting for 78·1% (75·8–80·3) of neonatal mortality.

Among children aged 1–59 months, mortality was primarily attributable to lower respiratory infections (0·54 million [95% UR 0·43–0·61]; 18·7% [UR 15·3–20·2]), diarrhoea (0·45 million [0·37–0·51]; 15·7% [13·5–16·9]), malaria (0·42 million [0·34–0·50]; 15·8% [13·5–16·9]), and injuries (0·25 million [0·23–0·29]; 8·8% [7·8–9·5]), altogether accounting for 57·8% (50·5–60·8) of deaths in this age group.

**Figure 2:**
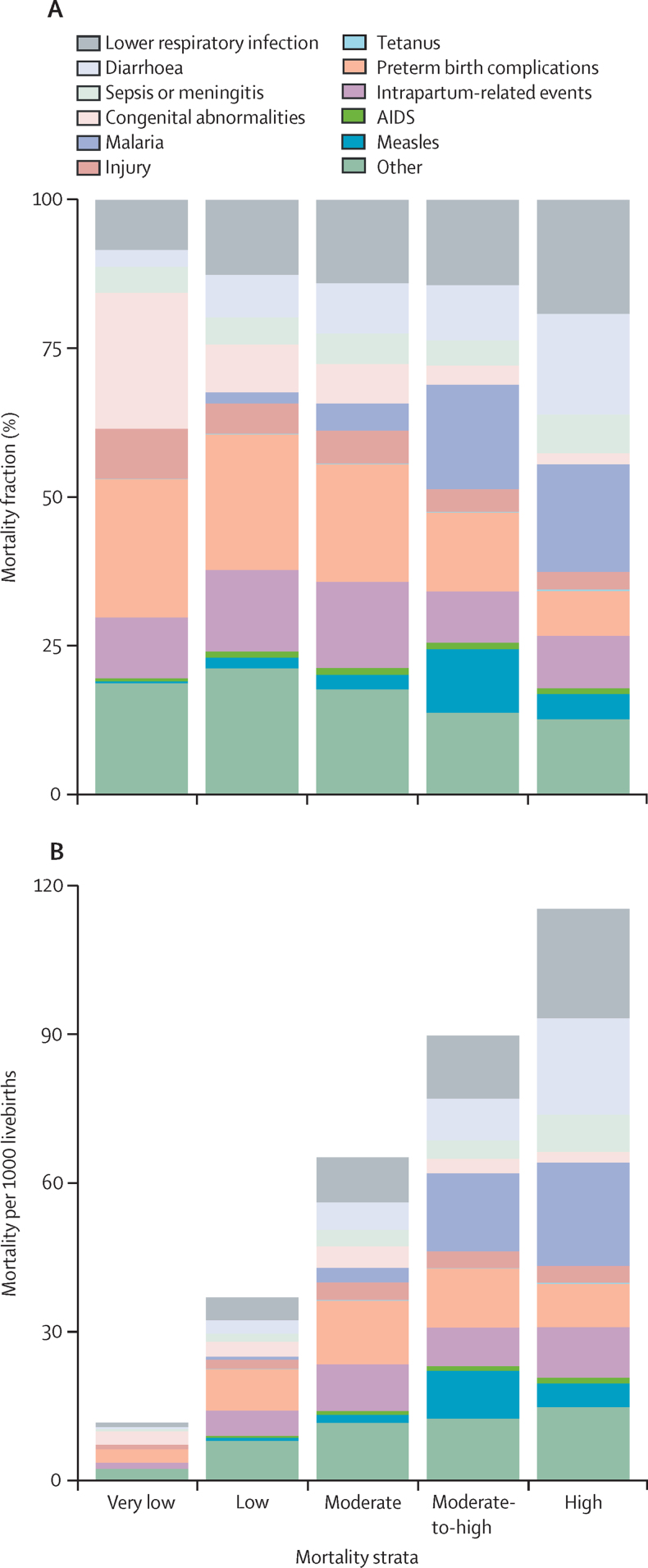
Cause-specific under-5 mortality in 2019 (A) Cause-of-death mortality fraction. (B) Mortality per 1000 livebirths. Mortality strata are defined as very low (<25 deaths per 1000 livebirths), low (25–50 deaths per 1000 livebirths), moderate (50–75 deaths per 1000 livebirths), moderate-to-high (75–100 deaths per 1000 livebirths), and high (>100 deaths per 1000 livebirths).

Cause-specific mortality in 2019 varied considerably by geographical area and by all-cause mortality strata (figure 2). In six countries with all-cause mortality of 100 or higher in 2019 (in order of descending mortality rate: Nigeria, Central African Republic, Somalia, Chad, Guinea, and Sierra Leone), lower respiratory infections (19·2% [95% UR 15·5–20·7]), malaria (18·1% [13·7–20·2]), and diarrhoea (16·8% [13·4–18·3]) were the most prominent causes, together accounting for 0·61 million (54·1% [43·5–56·8]) of 1·12 million under-5 deaths in these countries. There were 11 countries (in order of descending mortality rate: South Sudan, Democratic Republic of the Congo, Liberia, Mali, Benin, Lesotho, Burkina Faso, Equatorial Guinea, Guinea-Bissau, Niger, and Cote d'Ivoire) with all-cause under-5 mortality between 75 and 100 per 1000 livebirths, having a total of 0·74 million deaths. The most prominent causes in these countries were malaria (0·13 million [0·10–0·18]; 17·7% [15·0–20·3]), lower respiratory infections (0·11 million [0·08–0·14]; 14·3% [12·5–16·0]), and preterm birth complications (0·10 million [0·07–0·14], 13·2% [10·6–16·5]). By contrast, there were 121 countries (appendix p 96) with very low all-cause under-5 mortality rates below 25 deaths per 1000 livebirths. Among these countries, the most prominent causes of death were due to preterm birth complications (0·16 million [0·15–0·18]; 23·3% of 0·68 million deaths [21·6–24·8%), intrapartum-related events (0·07 million [0·06–0·08]; 10·2% [9·3–11·2]), and lower respiratory infections (0·06 million [0·05–0·06]; 8·4% [7·1–9·2]). Cause-specific mortality also varied by UNICEF region. Malaria (18·0% [15·0–19·7]) and lower respiratory infections (16·8% [14·3–18·0]) had the highest proportions in West and Central Africa; preterm birth complications (24·1% [19·9–28·8]) and intrapartum-related events (16·0%, [13·6–17·8]) were highest in South Asia; and preterm birth complications (20·7% [18·4–22·8]) and congenital abnormalities (16·7% [14·1–18·1]) were highest in East Asia and the Pacific region. Cause fractions in 2019 for all UNICEF regions are available in the appendix (pp 98–99).

The ten countries with the highest number of under-5 deaths are Nigeria, India, Pakistan, Democratic Republic of the Congo, Ethiopia, China, Indonesia, Tanzania, Bangladesh, and Angola. Together, these ten countries have 3·15 million under-5 deaths, which represents 59·5% of the global burden. The most prevalent cause of under-5 mortality in these countries in 2019 was preterm birth complications, ranging from 6·3% to 25·7% of all under-5 mortality, equivalent to cause-specific mortality rates of between 1·3 deaths per 1000 livebirths and 14·4 deaths per 1000 livebirths. Lower respiratory infections (5·4–19·5%; 0·6–21·7 deaths per 1000 livebirths) and intrapartum-related events (7·4–20·6%; 1·1–13·6 deaths per 1000 livebirths) were also relatively common in these high-burden countries.

**Figure 3:**
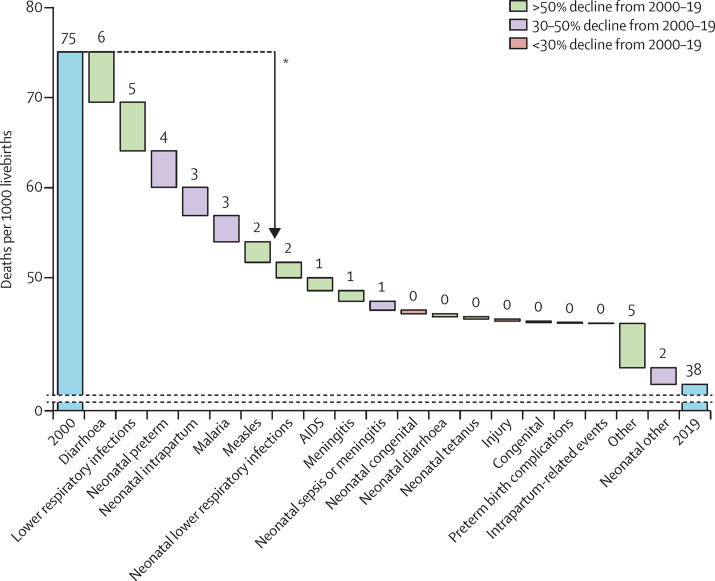
Global trends in cause-specific mortality rates in neonates and children aged 1–59 months, 2000–19 The length of each bar represents the amount of change in all-cause under five mortality rate attributable to changes in the mortality rate for each cause. The numbers above the bars are the change in each cause-specific mortality rate. *63% of the reduction is due to lower respiratory infections, diarrhoea, neonatal preterm birth complications, neonatal intrapartum-related events, malaria, and measles.

The global decrease in under-5 mortality is primarily attributable to decreases in the number of deaths caused by diarrhoea (8·9 deaths per 1000 livebirths [95% UR 8·4–9·3] in 2000 to 3·2 deaths per 1000 livebirths [2·6–3·7] in 2019), lower respiratory infections (9·3 [9·1–10·0] to 3·8 [3·1–4·4]), neonatal preterm birth complications (10·4 [9·3–11·8] to 6·3 [5·8-7·2]), neonatal intrapartum-related events (7·4 [6·6–7·9] to 4·2 [3·8–4·7]), malaria (5·9, [5·5–6·4] to 3·0 [2·4–3·6]), and measles (3·6 [2·7–5·0] to 1·3 [0·8–3·3]). Changes in these causes constitute 63·3% (58·5–66·9) of the total decrease in the under-5 mortality rate from 2000. Global declines for all causes from 2000 to 2019 are shown in figure 3. Since 2000, the proportion of mortality due to lower respiratory infections has declined from 16·6% (14·8–17·9) to 14·0% (12·0–15·1) and diarrhoea mortality has declined from 12·6% (12·0–13·1) to 9·1% (7·9–9·9), whereas the proportion of mortality due to preterm birth complications has increased from 14·5% (13·0–16·1) to 17·7% (16·1–19·4) and mortality due to intrapartum-related events has increased from 10·1% (9·1–10·8) to 11·6% (10·6–12·5).

In West and Central Africa, the decrease in under-5 mortality was accompanied by modest changes in the composition of underlying causes of mortality, with mortality due to malaria declining from 24·2% (95% UR 22·3–25·9) to 18·0% (15·0–19·7). In South Asia, lower respiratory infections declined from 17·5% (15·2–20·6) to 11·7% (9·6–14·4) of all under-5 deaths, and preterm birth complications increased from 17·6% (13·7–21·9) to 24·1% (19·9–28·8). Cause-specific mortality for all UNICEF regions in 2000 and 2019 are available in the appendix (pp 98–99).

**Figure 4:**
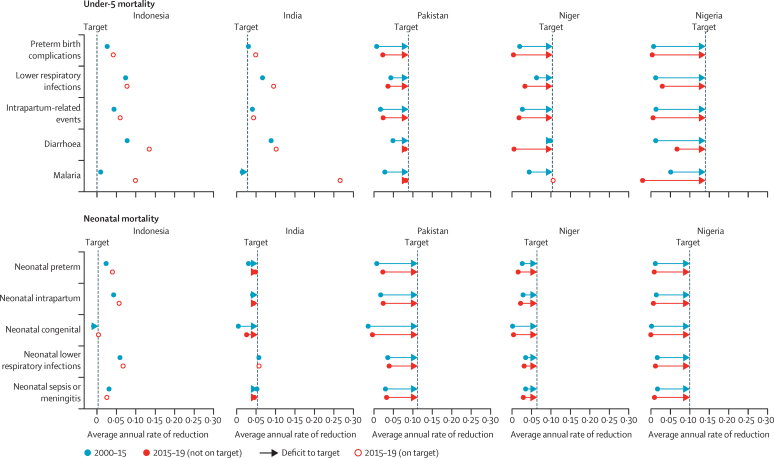
Projected cause-specific progress needed to meet the SDG child survival target for under-5 mortality by 2030 The SDG target for mortality in children younger than 5 years is <25 deaths per 1000 livebirths, and that among neonates is <12 per 1000 livebirths. Progress is shown for countries with very low (<25 deaths per 1000 livebirths; eg, Indonesia), low (25–50 deaths per 1000 livebirths; eg, India), moderate (50–75 deaths per 1000 livebirths; eg, Pakistan), moderate-to-high (75–100 deaths per 1000 livebirths; eg, Niger), and high under-5 mortality (>100 deaths per 1000 livebirths; eg, Nigeria). SDG=Sustainable Development Goals.

Among countries with very low under-5 mortality, decline in Indonesia accelerated in the SDG period (2015–19) compared with the MDG period (2000–15) for several cause-specific mortality rates, including diarrhoea, malaria, and preterm birth complications (figure 4). Among countries with low under-5 mortality, India has also had a fast decline for most causes of death in the SDG period compared with the MDG period, although among neonates, mortality due to congenital abnormalities and intrapartum-related events is declining more slowly than other causes of mortality. Average annual rate of reduction in under-5 mortality by cause is also shown for Pakistan, where, although most causes are declining more rapidly in the SDG than during the MDG period, declines are not fast enough to meet the SDG targets by 2030, especially for mortality due to lower respiratory infections, preterm birth complications, and congenital abnormalities.

Among countries with moderate-to-high under-5 mortality, Niger had under-5 mortality due to malaria declining at the target rate; however, lower respiratory infections, diarrhoea, and preterm birth complications all declined more slowly in the SDG period than in the MDG period. Similarly, in Nigeria, most causes are declining more slowly in the SDG period than the MDG period, with the exception of lower respiratory infections and diarrhoea. Comparisons between these two time periods for the other countries are in the appendix (pp 102–270).

## Discussion

In this analysis we have updated estimates of cause-specific mortality for children younger than 5 years in all 194 WHO Member States from 2000 to 2019. We have improved our methods for estimating cause-specific fractions of mortality for both moderate-to-high and low-mortality settings. We have enabled covariate selection in the multinomial model using the Bayesian LASSO with a robust out-of-sample method for determining the degree of parameter restriction. We have also made our estimates more responsive to country data by systematically giving nationally representative verbal autopsy studies more weight in country-specific estimates through random effects.

There are differences in this series compared with previous estimates. Estimated measles deaths were high in 2019, with more than 100 000 deaths in 2019 compared with less than 10 000 previously reported in 2017.^12^ This high level is related to several measles epidemics that occurred in 2019.^17^, ^18^ We also estimated higher other causes for neonates in this series than previous estimates, due primarily to new studies with verbal autopsies reporting more non-specific causes (6·2% of studies from the 1980s *vs* 22% of studies from 2010 or later). We also estimated lower proportions of deaths caused by sepsis and higher proportions of deaths caused by lower respiratory infections than in previous estimates. Many verbal autopsy studies in our systematic review do not distinguish between sepsis and lower respiratory infections among neonates, given the difficulties in diagnosing these infections with verbal autopsies.^19^ Studies since 2010 have reported higher deaths from lower respiratory infections than deaths from sepsis, with 56% deaths attributable to lower respiratory infections of the total of lower respiratory infections and sepsis deaths in the 2000s relative to 66% in studies after 2010.

Estimates have also changed because of methodological updates. Deaths due to preterm birth complications have declined in some countries with national verbal autopsy studies that report low fractions of mortality due to preterm birth complications, notably in Nigeria, as these studies have more weight in the final estimates through random effects. For children aged 1 to 59 months we estimate higher malaria deaths than in previous estimates, partly because of the additional effect of studies in Nigeria, Niger, and Mozambique, and partly because of the updated covariates (eg, in Democratic Republic of Congo and Chad). Other countries have updated cause-specific mortality because of the incorporation of national verbal autopsy studies, including Bangladesh, India, Mozambique, and Tanzania (appendix pp 44–55).

More country data is needed to improve estimates of cause-specific mortality, especially given the new methods that we have presented, which can now use country-specific data to its full advantage. Estimates will benefit from additional cause-of-death data from low-income and middle-income countries collected through verbal autopsy studies and sample registration systems specific to national circumstances. All countries, even those without high-quality vital registration, can systematically inform their cause-specific mortality with nationally representative cause-of-death data.

These estimates represent an effort to increase the transparency of cause-specific mortality estimates. Comprehensive estimates are available for cause-specific under-5 mortality from the GBD series, which use complicated models requiring time and resource intensive computing that is inaccessible to most of the world's governments.^8^, ^20^ The methods and primary data sources for GBD are different than those reported in this study. There is broad agreement between these two series of estimates at the global level for 2019, with similar cause fractions among children aged younger than 5 years for lower respiratory infections (13·8% in this study *vs* 13·3% in GBD), malaria (7·9% *vs* 7·1%), and diarrhoea (9·1% *vs* 9·9%), but less so for preterm birth complications (17·8% *vs* 13·2%) and measles (3·5% *vs* 1·4%). This series is also in contrast to the Global Health Estimates (GHE), released by WHO and covering the same time period from 2000 to 2019.^21^ The GHE are based on the 2000–17 estimates from this series and, therefore, differ from the data presented in our study. Compared with these estimates, globally for 2019, GHE estimated a higher proportion of neonatal sepsis and congenital abnormalities. For children aged 1–59 months in 2019, GHE estimates are generally lower for both measles and malaria than as presented in this study.

These estimates have implications for policies and programmes to prevent mortality among children younger than 5 years. Vaccine-preventable illnesses such as measles, lower respiratory infections, and meningitis were still the underlying cause for 21·7% of under-5 deaths in 2019. Some types of diarrhoea are also preventable with vaccines (eg, rotavirus), and most deaths due to diarrhoea are preventable with low-cost interventions such as oral rehydration therapy and zinc.^22^ Malaria is also preventable with low-cost interventions such as insecticide-treated bed nets and treatable with artemisinin-based combination therapy.^23^ High-mortality areas in general need to improve access to health care, including preventive care and treatments for life-threatening conditions.^24^ More research is needed to improve logistics in increasing access to health care and implementing vaccine campaigns for low-income areas, including how to address vaccine hesitancy.^25^ Such efforts will reduce preventable deaths and are also likely to improve child health and wellbeing, as mandated by the expanded focus of the SDG to include the morbidity and thriving of all children, exemplified by the child health redesign.^26^ For high-mortality countries where infectious causes are still the main causes of mortality burden, mass distribution of azithromycin could be considered (along with a strategy to mitigate antibiotic resistance); estimates here could be used to gauge the resulting cost–benefit ratio of such programmes.^27^, ^28^ There are also low-cost interventions, such as neonatal resuscitation for intrapartum-related events,^29^ which have the potential to reduce neonatal mortality in low resource settings. Countries are also likely to benefit from increased access to health care including facility delivery and emergency obstetric care, as well as increased quality of delivery care in facilities.^30^ More research is needed into how to effectively scale up interventions for neonatal survival and into what is most beneficial in low-income areas.

As child mortality declines globally, preventable deaths in children under-5 years could become increasingly concentrated in subnational areas and hotspots.^31^ It is possible that the methods shown here could be adapted for subnational estimation. Sample registration systems would be informative in any exercise to model subnational causes of death and are useful in identifying high mortality areas within countries as well as the underlying causes of mortality. Sample registration systems, even those in development, have the added benefit of building capacity in low-income areas within countries,^32^ and could benefit future models and estimates. Such capacity could also build local and regional expertise for examining and estimating cause-specific mortality. Among the 194 WHO Member States, 81 countries did not contribute data on cause-specific under-5 mortality either from high-quality registration data or from our systematic review, many of which could benefit from sample registration systems or national verbal autopsy studies.

Estimates of cause-specific child mortality in 2020 and future years are faced with uncertainty due to the COVID-19 pandemic. Although children younger than 5 years are among the lowest risk group for mortality due to COVID-19, child mortality could be affected indirectly by health system stress and economic conditions. There is evidence that child mortality has been affected in some countries, for example, with increased neonatal mortality^33^ and reduced mortality due to injury.^34^ In areas with moderate-to-high under-5 mortality, there has been less direct reporting and, therefore, additional uncertainty. Further research is needed to understand the effects of COVID-19 on child mortality and causes of deaths.

Our estimates have some limitations. We did not estimate cause-specific mortality by sex. We estimated causes of death in countries that are transitioning from moderate to low child mortality using model averaging, but the uncertainty in the relative contribution of each model is not included in our estimate of uncertainty. Other sources of uncertainty are also not accounted for, including uncertainty introduced when estimating covariates. However, we do account for variability across studies in the number of deaths reported by assuming a multinomial distribution for the number of deaths by cause. We also have not included misclassification error due to cause assignment in verbal autopsy in our uncertainty estimation. We expect that our uncertainty estimates are made narrower by these exclusions. We did not assess bias in the causes of death for studies included in our systematic review, and there is likely to be unexplained heterogeneity in reported cause distributions. Our systematic review was not registered and our protocol for analysis was also not registered.

These estimates also have considerable strengths. The estimates presented in this study cover a more recent time period than previous results, come from a statistical model that is systematic in covariate selection, and incorporates empirical data directly from nationally representative studies (appendix pp 44–55). We have provided a comprehensive time series of cause of death estimates for all countries with transparent methods and publicly available source data, which enables future replication. With country random effects, we also hope to promote primary data collection in countries without high-quality vital registration.

Civil registration and vital statistics are needed for all countries regardless of income or resources. However, while vital registration systems are in development, we provide these results to inform public health practitioners and decision makers about what interventions are needed and where they are needed most. Many deaths in children younger than 5 years have already been prevented in response to the SDG targets set for 2030. Along with continued effort and investment, high-quality cause of death estimates can assist countries and the international community to accelerate these efforts and end preventable child deaths so that all countries can meet SDG 3.2 by 2030.

**This online publication has been corrected. The corrected version first appeared at thelancet.com/child-adolescent on December 15, 2021**

## Data sharing

The source code, primary inputs, and cause of death data collected in the systematic review is publicly available for research purposes at https://github.com/amulick/MCEE-u5mort2019.

## Declaration of interests

We declare no competing interests.

## References

[1] UN (Sept 15, 2015). The millennium development goals report 2015. https://www.un.org/millenniumgoals/2015_MDG_Report/pdf/MDG%202015%20rev%20(July%201).pdf.

[2] UN General Assembly (September, 2015). Transforming our world: the 2030 Agenda for Sustainable Development. https://sustainabledevelopment.un.org/post2015/transformingourworld.

[3] Liu L, Oza S, Hogan D (2016). Global, regional, and national causes of under-5 mortality in 2000–15: an updated systematic analysis with implications for the Sustainable Development Goals. Lancet.

[4] Efron B (2012).

[5] Mulick AR, Oza S, Prieto-Merino D, Villavicencio F, Cousens S, Perin J (2021). A Bayesian hierarchical model with integrated covariate selection and misclassification matrices to estimate neonatal and child causes of death. medRxiv.

[6] He C, Liu L, Chu Y (2017). National and subnational all-cause and cause-specific child mortality in China, 1996–2015: a systematic analysis with implications for the Sustainable Development Goals. Lancet Glob Health.

[7] UN Inter-agency Group for Child Mortality Estimation (September, 2020). Levels and trends in child mortality 2020. https://www.unicef.org/reports/levels-and-trends-child-mortality-report-2020.

[8] Wang H, Abbas KM, Abbasifard M (2020). Global age-sex-specific fertility, mortality, healthy life expectancy (HALE), and population estimates in 204 countries and territories, 1950–2019: a comprehensive demographic analysis for the Global Burden of Disease Study 2019. Lancet.

[9] WHO (Nov 30, 2020). World malaria report 2020: 20 years of global progress and challenges. https://www.who.int/publications/i/item/9789240015791.

[10] WHO (Oct 15, 2020). Global tuberculosis report 2020. https://www.who.int/publications/i/item/9789240013131.

[11] Perez-Velez CM, Marais BJ (2012). Tuberculosis in children. N Engl J Med.

[12] WHO (December, 2018). MCEE-WHO methods and data sources for child causes of death 2000-2017. https://www.who.int/healthinfo/global_burden_disease/childcod_methods_2000_2017.pdf.

[13] UNICEF (Dec 12, 2016). Malnutrition amongst children in Yemen at an all-time high, warns UNICEF. https://www.unicef.org/press-releases/malnutrition-amongst-children-yemen-all-time-high-warns-unicef.

[14] França GV, Schuler-Faccini L, Oliveira WK (2016). Congenital Zika virus syndrome in Brazil: a case series of the first 1501 livebirths with complete investigation. Lancet.

[15] Daniels JP (2019). Venezuela in crisis. Lancet Infect Dis.

[16] Stevens GA, Alkema L, Black RE (2016). Guidelines for accurate and transparent health estimates reporting: the GATHER statement. PLoS Med.

[17] Finnegan KE, Haruna J, Cordier LF (2020). Rapid response to a measles outbreak in Ifanadiana District, Madagascar. medRxiv.

[18] WHO (Jan 7, 2020). Deaths from Democratic Republic of the Congo measles outbreak top 6000. https://www.afro.who.int/news/deaths-democratic-republic-congo-measles-outbreak-top-6000.

[19] Ahmed I, Ali SM, Amenga-Etego S (2018). Population-based rates, timing, and causes of maternal deaths, stillbirths, and neonatal deaths in south Asia and sub-Saharan Africa: a multi-country prospective cohort study. Lancet Glob Health.

[20] Mathers CD (2020). History of global burden of disease assessment at the World Health Organization. Arch Public Health.

[21] WHO (2020). Global Health Estimates 2019: deaths by cause, age, sex, by country and by region, 2000–2019. https://www.who.int/data/gho/data/themes/mortality-and-global-health-estimates.

[22] Nalin DR, Cash RA (2018). 50 years of oral rehydration therapy: the solution is still simple. Lancet.

[23] Davis WA, Clarke PM, Siba PM (2011). Cost-effectiveness of artemisinin combination therapy for uncomplicated malaria in children: data from Papua New Guinea. Bull World Health Organ.

[24] Okwaraji YB, Cousens S, Berhane Y, Mulholland K, Edmond K (2012). Effect of geographical access to health facilities on child mortality in rural Ethiopia: a community based cross sectional study. PLoS One.

[25] Larson Williams A, Mitrovich R, Mwananyanda L, Gill C (2019). Maternal vaccine knowledge in low- and middle-income countries-and why it matters. Hum Vaccin Immunother.

[26] Requejo J, Strong K (2021).

[27] Keenan JD, Arzika AM, Maliki R (2019). Longer-term assessment of azithromycin for reducing childhood mortality in Africa. N Engl J Med.

[28] Keenan JD, Bailey RL, West SK (2018). Azithromycin to reduce childhood mortality in sub-Saharan Africa. N Engl J Med.

[29] Khanam R, Baqui AH, Syed MIM (2018). Can facility delivery reduce the risk of intrapartum complications-related perinatal mortality? Findings from a cohort study. J Glob Health.

[30] Mukuru M, Kiwanuka SN, Gibson L, Ssengooba F (2021). Challenges in implementing emergency obstetric care (EmOC) policies: perspectives and behaviours of frontline health workers in Uganda. Health Policy Plan.

[31] Burke M, Heft-Neal S, Bendavid E (2016). Sources of variation in under-5 mortality across sub-Saharan Africa: a spatial analysis. Lancet Glob Health.

[32] Phillips DE, AbouZahr C, Lopez AD (2015). Are well functioning civil registration and vital statistics systems associated with better health outcomes?. Lancet.

[33] Kc A, Gurung R, Kinney MV (2020). Effect of the COVID-19 pandemic response on intrapartum care, stillbirth, and neonatal mortality outcomes in Nepal: a prospective observational study. Lancet Glob Health.

[34] Hartnett KP, Kite-Powell A, DeVies J (2020). Impact of the COVID-19 pandemic on emergency department visits—United States, January 1, 2019–May 30, 2020. MMWR Morb Mortal Wkly Rep.

