# Correction to *Lancet Child Adolesc Health* 2021; published online Nov 17. https://doi.org/10.1016/S2352-4642(21)00311-4

**DOI:** 10.1016/S2352-4642(21)00382-5

**Published:** 2022-01

*Perin J, Mulick A, Yeung D, et al. Global, regional, and national causes of under-5 mortality in 2000–19: an updated systematic analysis with implications for the Sustainable Development Goals.* Lancet Child Adolesc Health *2021; published online Nov 17. https://doi.org/10.1016/S2352-4642(21)00311-4*—In table 2 of this Article, the estimations for children aged 0–59 months with preterm birth complications, lower respiratory infections, diarrhoea, and congenital abnormalities and for children aged 1–59 months with other conditions were incorrect. These corrections have been made to the online version as of Dec 15, 2021, and will be made to the printed version.

