# Supplementary material for: Global, regional, and national causes of under-5 mortality in 2000–19: an updated systematic analysis with implications for the Sustainable Development Goals
Source: Lancet Child Adolesc Health. 2022 Feb;6(2):106–15. doi: 10.1016/S2352-4642(21)00311-4 (PMC8786667; doi:10.1016/S2352-4642(21)00311-4)
Supplement: Supplementary appendix [file mmc1.pdf]

THE LANCET
Child & Adolescent Health

Supplementary appendix

This appendix formed part of the original submission and has been peer reviewed. We post it as supplied by the authors.

Supplement to: Perin J, Mulick A, Yeung D, et al. Global, regional, and national causes of under-5 mortality in 2000–19: an updated systematic analysis with implications for the Sustainable Development Goals. *Lancet Child Adolesc Health* 2021; published online Nov 17. https://doi.org/10.1016/S2352-4642(21)00311-4.

Progress towards the Sustainable Development Goals: Global, regional, and national causes of under-five mortality during 2000-2019

Appendices

2021-11-05

Contents

Appendix 1 Details of the updated systematic review **5**

Appendix 2: Updated study data points **29**

Appendix 3: Method details **31**
ICD codes and cause of death 31
Methods neonatal mortality 33
Methods 1-59 month mortality 38

Appendix 4: Weight of random effects in nationally represetative studies **43**

Appendix 5: Convergence of fixed effects (trace plots) **57**

Appendix 6: GATHER checklist **91**

Appendix 7: Countries with very low under-5 mortality **95**

Appendix 8: Distribution of causes of child death in UNICEF regions in 2000 and 2019 **97**

Appendix 9: Average Annual rate of reduction by cause in all countries with 50 or more under-five deaths in 2019 for 2000-2015 and 2015-2019 **101**

Appendix 1 Details of the updated systematic review

Appendix 1: Details of the updated systematic review of the neonatal and postneonatal VA studies published between January 1, 2015 and December 31, 2017, no language restrictions

Database	Search Terms	Date conducted
Pubmed	((((((((("Infant"[mesh] OR "Infant, Newborn"[mesh] OR "child"[mesh] OR "Child, Preschool"[Mesh] OR "Minors"[Mesh] OR "infant"[tw] OR "infants"[tw] OR "neonate"[tw] OR "neonates"[tw] OR "neonatal"[tw] OR "newborn"[tw] OR "newborns"[tw] OR "new-born"[tw] OR "new-borns"[tw] OR "baby"[tw] OR "babies"[tw] OR "Premature"[tw] OR "preterm"[tw] OR "pre term"[tw] OR "child"[tw] OR "children"[tw] OR "youth"[tw] OR "youths"[tw] OR "young people"[tw] OR "childhood"[tw] OR "toddler"[tw] OR "toddlers"[tw] OR "kid"[tw] OR "kids"[tw] OR "young patient"[tw] OR "young patients"[tw] OR "boy"[tw] OR "boys"[tw] OR "girl"[tw] OR "girls"[tw] OR "young age"[tw] OR "pediatric"[tw] OR "pre-schooler"[tw] OR "preschooler"[tw] OR "under 5"[tw] OR "under five"[tw] OR "under fives"[tw] OR "less than five"[tw] OR "perinatal"[tw]))<br>AND<br>("Mortality"[MeSH] OR "Mortality"[tw] OR "Mortalities"[tw] OR "fatal"[tw] OR "fatality"[tw] OR "fatalities"[tw] OR "Death"[MeSH] OR "death"[tw] OR "deaths"[tw])))<br>AND<br>((((((((((("Meningitis"[Mesh] OR "Meningitis"[tw] OR "Meningitides"[tw] OR "Pachymeningitis"[tw] OR "Pachymeningitides"[tw] OR "Arachnoiditis"[tw] OR "Arachnoiditides"[tw] OR "Arachnoid Membrane inflammation"[tw] OR "Arachnoid Membrane inflammations"[tw] OR "Meningoencephalitides"[tw] OR "Meningoencephalitis"[tw] OR "Meningocephalitis"[tw] OR "Cerebromeningitis" OR "Cerebromeningitides" OR "Encephalomeningitis"[tw] OR "Encephalomeningitides"[tw] OR "Haemophilus influenzae"[tw] OR "Haemophilus parainfluenzae"[tw] OR "Cerebritis"[tw] OR "Cerebritides"[tw] OR "Waterhouse Friderichsen Syndrome"[tw] OR "Waterhouse Friederichsen Syndrome"[tw] OR "Waterhouse-Friderichsen Syndrome"[tw] OR "Waterhouse-Friederichsen Syndrome"[tw] OR "Purpura Fulminans"[tw] OR "Meningococcal"[tw] OR "Meningeal"[tw] OR "Cerebral Cryptococcosis"[tw] OR "Cerebral Cryptococcoses"[tw] OR "Toruloma"[tw] OR "Torulomas"[tw] OR "Lymphocytic Choriomeningitis"[tw] OR "Armstrong Syndrome"[tw] OR "Armstrong's Syndrome"[tw] OR "Encephalitis"[Mesh] AND "Encephalitis"[tw] OR "Encephalitides"[tw] OR "encephalitis"[tw] OR "brain inflammation"[tw] OR "brain inflammations"[tw] OR "Rasmussen syndrome"[tw] OR "Rasmussen's syndrome"[tw] OR "Cerebral Ventriculitis"[tw] OR "Cerebral Ventriculitides"[tw] OR "Infectious Ventriculitis"[tw] OR "Infectious Ventriculitides"[tw] OR "Encephalomyelitis"[tw] OR "Meningoencephalitides"[tw] OR "Meningoencephalitis"[tw] OR "Cerebromeningitis"[tw] OR "Cerebromeningitides"[tw] OR "Encephalomeningitis"[tw] OR "Encephalomeningitides"[tw] OR "myeloencephalitis"[tw] OR "Lupus Vasculitis"[tw] OR "Central Nervous System Lupus"[tw] OR "Lupus Erythematosis"[tw] OR "Lupus Erythematosus"[tw] OR "brain vasculitis"[tw] OR "brain angiitis"[tw] OR "brain arteritis"[tw] OR "cerebral arteritis"[tw] OR "cerebral vasculitis" OR "Neisseria meningitidis"[Mesh] OR "Neisseria meningitidis"[tw] OR "Neisseria meningitides"[tw] OR "Micrococcus intracellularis"[tw] OR "Neisseria weichselbaumi"[tw] OR "Meningococcus"[tw] OR "meningococcemia"[tw] OR "meningococci"[tw] OR "Meningococcal Infections"[Mesh] OR "meningococceal"[tw] OR "meningococcaemia"[tw] OR "sepsis"[MeSH] OR "sepsis"[tw] OR "Pyemia"[tw] OR "Pyemias"[tw] OR "Pyohemia"[tw] OR "Pyohemias"[tw] OR "Pyaemia"[tw] OR "Pyaemias"[tw] OR "Septicemia"[tw] OR "Septicemias"[tw] OR "Blood Poisoning"[tw] OR "Blood Poisonings"[tw] OR "septic"[tw] OR "sepses"[tw])) OR ("premature birth"[MeSH] OR "premature"[tw] OR "preterm"[tw] OR "pre-mature"[tw] OR "pre-term"[tw] OR "prematurity"[tw] OR "prematuritas"[tw])) OR ("rubella"[MeSH] OR "rubella"[tw] OR "measles"[tw] OR "epidemic roseola" OR "necrosis"[MeSH] OR "necrosis"[tw] OR "necrotic"[tw] OR "necroses"[tw] OR "necrotizing"[tw] OR "Infarction"[tw] OR "infractions"[tw] OR "birth injuries"[MeSH] OR "birth injuries"[tw] OR "birth injury"[tw] OR "birth trauma"[tw] OR "Obstetric Paralyses"[tw] OR "Obstetric Paralysis"[tw] OR "Obstetrical Paralyses"[tw] OR "Obstetrical Paralysis"[tw] OR "birth damage"[tw] OR "birth lesion"[tw] OR "birth palsy"[tw] OR "birth paralysis"[tw] OR "obstetric palsy"[tw] OR "obstetrical palsy"[tw] OR "brachial palsy"[tw] OR "brachial paralysis"[tw] OR "brachial plexus paralysis"[tw] OR "delivery trauma"[tw] OR "arm paralysis"[tw] OR "paralysis brachialis"[tw] OR "jaundice"[MeSH] OR "jaundice"[tw] OR "jaundices"[tw] OR "Icterus"[tw] OR "bronze baby syndrome"[tw] OR "erythroleukoblastosis"[tw] OR "hemorrhage"[MeSH] OR "hemorrhage"[tw] OR "haemorrhage"[tw] OR "haemorrhagic"[tw] OR "Hemorrhagic"[tw])) OR ("tetanus"[MeSH] OR "tetanus"[tw] OR "tetani"[tw] OR “diphtheria-tetanus-pertussis”[tw])) OR ("asphyxia"[MeSH] OR "asphyxia"[tw] OR "Asphyxia Neonatorum"[MesH] OR "Asphyxia Neonatorum"[tw] OR "hypoxia"[tw] OR "Asphyxias"[tw] OR "Suffocation"[tw] OR "Suffocations"[tw] OR "anoxia"[tw] OR ("intrapartum"[tw] AND "related"[tw]))) OR ("congenital abnormalities"[MeSH] OR "congenital"[tw] OR "Deformities"[tw] OR "Deformity"[tw] OR "Birth Defects"[tw] OR "Birth Defect"[tw] OR "abnormality"[tw] OR "abnormalities"[tw] OR "malformation"[tw] OR "malformations"[tw] OR "malformative"[tw] OR "malformed"[tw] OR "neural tube defects"[MeSH] OR "neural tube"[tw] OR "Craniorachischisis"[tw] OR "Craniorachischises"[tw] OR "Diastematomyelia"[tw] OR "Diastematomyelias"[tw] OR "Tethered Cord"[tw] OR "Spinal Dysraphism"[tw] OR "Tethered Spinal Cord"[tw] OR "Spinal Dysraphisms"[tw] OR "Iniencephaly"[tw] OR "Iniencephalies"[tw] OR "Neurenteric"[tw] OR "Neuroenteric"[tw] OR "Spinal Cord Myelodysplasia"[tw] OR "Spinal Cord Myelodysplasias"[tw] OR "Acrania"[tw] OR "Acranias"[tw] OR	January 12, 2018

	"Exencephaly"[tw] OR "Exencephalies"[tw] OR "dysgraphia"[tw] OR "dysraphic"[tw] OR "dysraphism"[tw] OR "dysraphy"[tw] OR "NTD"[tw] OR "NTDS"[tw] OR "sudden infant death syndrome"[MeSH] OR "sudden infant death"[tw] OR "sid"[tw] OR "SIDS"[tw] OR "cot death"[tw] OR "cot deaths"[tw] OR "crib death"[tw] OR "fetal alcohol spectrum disorders"[MeSH] OR "Fetal alcohol spectrum"[tw] OR "Foetal alcohol spectrum"[tw] OR "FASD"[tw] OR "FASDs"[tw] OR "Fetal Alcohol Syndrome"[tw] OR "Alcohol-Related Birth Defects"[tw] OR "Alcohol Related Birth Defects"[tw] OR "Alcohol Related Neurodevelopmental Disorder"[tw] OR "FAE"[tw] OR "Fetal Alcohol Effects"[tw] OR "FAEs"[tw] OR "Fetal Alcohol Syndrome"[tw] OR "Fetus Alcohol Syndrome"[tw] OR "Foetal alcohol syndrome"[tw])) OR ("Nutrition disorders"[MeSH] OR “malnutrition"[tw] OR "Nutritional"[tw] OR "Undernutrition"[tw] OR "Malnourishment"[tw] OR " Malnourishments"[tw] OR "Deficiency"[tw] OR "Deficiencies"[tw] OR "Refeeding Syndrome"[tw] OR “Starvation"[tw] OR "Starvations"[tw] OR "Famine”[tw] OR “Famines”[tw] OR “deficient"[tw] OR "underfeeding"[tw] OR "undernourishment"[tw] OR “nutrition”[tw] OR “Hypervitaminosis”[tw])) OR ("Malaria"[Mesh] OR "malaria"[tw] OR "malarias"[tw] OR "Plasmodium Infection"[tw] OR "Plasmodium Infections"[tw] OR "Plasmodium falciparum"[Mesh] OR "Plasmodium falciparum"[tw] OR "Plasmodium falciparums"[tw] OR "malaria, falciparum"[mesh] OR "blackwater fever"[tw] OR "blackwater fevers"[tw] OR "black water fever"[tw] OR "black water fevers"[tw] OR "remittent fever"[tw] OR "paludism"[tw] OR "plasmodium infection"[tw] OR "plasmodium infections"[tw] OR "marsh fever"[tw] OR "falciparum infection"[tw] OR "Plasmodium vivax"[Mesh] OR "Plasmodium vivax"[tw] OR "Plasmodium vivaxs"[tw] OR "malaria, vivax"[mesh] OR "vivax infection"[tw] OR "Plasmodium ovale"[Mesh] OR "Plasmodium ovale"[tw] OR "Plasmodium ovales"[tw] OR "Plasmodium malariae"[Mesh] OR "Plasmodium malariae"[tw])) OR ("Pneumonia"[Mesh] OR "pneumonia"[tw] OR "pneumonias"[tw] OR "pneumonitis"[tw] OR "pulmonary inflammation"[tw] OR "pulmonary inflammations"[tw] OR "lung inflammation"[tw] OR "Lung Inflammations"[tw] OR "inflammatory lung disease"[tw] OR "lobitis"[tw] OR "peripneumonia"[tw] OR "pleuropneumonia"[tw] OR "pleuropneumonitis"[tw] OR "pneumonitis"[tw] OR "pulmonal inflammation"[tw] OR "pulmonary inflammation"[tw] OR "pulmonic inflammation"[tw] OR "bronchopneumonia"[tw] OR "bronchopneumonias"[tw] OR "Respiratory Tract Infections"[Mesh] OR "Respiratory Tract Infections"[tw] OR "Respiratory Tract Infection"[tw] OR "Respiratory Infection"[tw] OR "Respiratory Infections"[tw] OR "whooping cough"[tw] OR "pertussis"[tw] OR "pulmonary tract infection"[tw] OR "respiration infection"[tw] OR "Bronchiolitis"[tw] OR "bronchitis"[tw] OR "bronchopulmonary infection"[tw] OR "Croup"[mesh] OR "Croup"[tw] OR "diphtheria"[tw] OR "laryngitis"[tw] OR "Severe Acute Respiratory Syndrome"[Mesh] OR "Severe Acute Respiratory Syndrome"[tw] OR "SARS"[tw] OR "Acute Chest Syndrome"[Mesh] OR "acute chest syndrome"[tw] OR "acute chest syndromes"[tw] OR "Acute Lower Respiratory Infection"[tw] OR "Acute Lower Respiratory Infections"[tw] OR "ALRI"[tw] OR "LRI"[tw] OR "ARI"[tw] OR "respiration tract infection"[tw] OR "respiration tract infections"[tw] OR "legionnaire disease"[tw] OR "legionnaires' disease"[tw] OR "legionnaires disease"[tw] OR "Pontiac fever"[tw] OR "lung infiltrate"[tw] OR "lung infiltration"[tw] OR "pulmonary infiltrate"[tw] OR "pulmonary infiltration"[tw] OR "pneumonic lung"[tw] OR "rds"[tw] OR "respiratory distress"[tw] OR "respiration distress"[tw])) OR ("Diarrhea"[Mesh] OR "Diarrhea"[tw] OR "Diarrheas"[tw] OR "Diarrheal"[tw] OR "diarrhoeal"[tw] OR "diarrhoea"[tw] OR "diarrhoeas"[tw] OR "Dysentery"[Mesh] OR "Dysentery"[tw] OR "dysenteria"[tw] OR "enteritis"[tw] OR "enteritides"[tw] OR "Cholera"[Mesh] OR "Cholera"[tw] OR "Choleras"[tw] OR "vibrio Cholerae"[tw] OR "Gastroenteritis"[Mesh] OR "gastroenteritis"[tw] OR "gastroenteritides"[tw] OR "gastro enteritis"[tw] OR "gastroduodenitis"[tw] OR "gastrointestinal acute infection"[tw] OR "gastrointestinal acute infections"[tw] OR "gastrointestinal infection"[tw] OR "gastrointestinal infections"[tw] OR "digestive tract infection"[tw] OR "digestive tract infections"[tw] OR "digestive infection"[tw] OR "gastrointestinal tract infection"[tw] OR "gastrointestine tract infection"[tw])) OR "Noncommunicable Diseases"[MeSH] OR "Noncommunicable"[tw] OR "Non-communicable"[tw] OR "communicable"[tw] OR "non-infectious"[tw] OR "infectious"[tw] OR "noninfectious"[tw] OR "Communicable Diseases"[MeSH] OR "Infant, Newborn, Diseases"[MeSH] OR "newborn"[tw] OR "neonatal"[tw] OR "perinatal"[tw])))<br>AND<br>("Argentina"[tw] OR "Bolivia"[tw] OR "Brazil"[tw] OR "Brasil"[tw] OR "Chile"[tw] OR "Colombia"[tw] OR "Ecuador"[tw] OR "French Guiana"[tw] OR "Guyana"[tw] OR "Paraguay"[tw] OR "Peru"[tw] OR "Suriname"[tw] OR "Uruguay"[tw] OR "Venezuela"[tw] OR "Mexico"[tw] OR "Belize"[tw] OR "Costa Rica"[tw] OR "El Salvador"[tw] OR "Guatemala"[tw] OR "Honduras"[tw] OR "Nicaragua"[tw] OR "Puerto Rico"[tw] OR "Panama"[tw] OR "West Indies"[tw] OR "Antigua"[tw] OR "Barbuda"[tw] OR "Bahamas"[tw] OR "Barbados"[tw] OR "Cuba"[tw] OR "Dominica"[tw] OR "Dominican Republic"[tw] OR "Grenada"[tw] OR "Guadeloupe"[tw] OR "Haiti"[tw] OR "Jamaica"[tw] OR "Martinique"[tw] OR "Antilles"[tw] OR "Anguilla"[tw] OR "Saint Kitts"[tw] OR "St Kitts"[tw] OR "Saint Lucia"[tw] OR "St Lucia"[tw] OR "Saint Vincent"[tw] OR "St Vincent"[tw] OR "Trinidad "[tw] OR "Tobago"[tw] OR "Virgin Islands"[tw] OR "Kazakhstan"[tw] OR "Kyrgyzstan"[tw] OR "Tajikistan"[tw] OR "Turkmenistan"[tw] OR "Uzbekistan"[tw] OR "Borneo"[tw] OR "Brunei"[tw] OR "Cambodia"[tw] OR "East Timor"[tw] OR "Indonesia"[tw] OR "Laos"[tw] OR "Malaysia"[tw] OR "Mekong Valley"[tw] OR "Myanmar"[tw] OR "Burma"[tw] OR "Philippines"[tw] OR "Singapore"[tw] OR "Thailand"[tw] OR "Vietnam"[tw] OR "Bangladesh"[tw] OR "Bhutan"[tw] OR "India"[tw] OR "Nepal"[tw] OR "Pakistan"[tw] OR "Sri Lanka"[tw] OR "China"[tw] OR "Korea"[tw] OR "Macao"[tw] OR "Macau"[tw] OR "Mongolia"[tw] OR "Taiwan"[tw] OR "Afghanistan"[tw] OR "Bahrain"[tw] OR "Iran"[tw] OR "Iraq"[tw] OR "Israel"[tw] OR "Jordan"[tw] OR "Kuwait"[tw] OR "Lebanon"[tw] OR "Oman"[tw] OR "Qatar"[tw] OR "Saudi Arabia"[tw] OR "Syria"[tw] OR "Turkey"[tw] OR "United Arab Emirates"[tw] OR "Yemen"[tw] OR "Fiji"[tw] OR "New Caledonia"[tw] OR "Papua New Guinea"[tw] OR "Vanuatu"[tw] OR "Micronesia"[tw] OR "Melanesia"[tw] OR "Guam"[tw] OR "Palau"[tw] OR "Polynesia"[tw] OR "Samoa"[tw] OR	

	"Tonga"[tw] OR "Armenia"[tw] OR "Azerbaijan"[tw] OR "Georgia"[tw] OR "Albania"[tw] OR "Estonia"[tw] OR "Latvia"[tw] OR "Lithuania"[tw] OR "Bosnia"[tw] OR "Herzegovina"[tw] OR "Serbia"[tw] OR "Bulgaria"[tw] OR "Belarus"[tw] OR "Croatia"[tw] OR "Czech Republic"[tw] OR "Hungary"[tw] OR "Macedonia"[tw] OR "Moldova"[tw] OR "Montenegro"[tw] OR "Poland"[tw] OR "Romania"[tw] OR "Russia"[tw] OR "Bashkiria"[tw] OR "Dagestan"[tw] OR "Slovakia"[tw] OR "Slovenia"[tw] OR "Ukraine"[tw] OR "Cameroon"[tw] OR "Central African Republic"[tw] OR "Chad"[tw] OR "Congo"[tw] OR "Equatorial Guinea"[tw] OR "Gabon"[tw] OR "Burundi"[tw] OR "Djibouti"[tw] OR "Eritrea"[tw] OR "Ethiopia"[tw] OR "Kenya"[tw] OR "Rwanda"[tw] OR "Somalia"[tw] OR "Sudan"[tw] OR "Tanzania"[tw] OR "Uganda"[tw] OR "Angola"[tw] OR "Botswana"[tw] OR "Lesotho"[tw] OR "Malawi"[tw] OR "Mozambique"[tw] OR "Namibia"[tw] OR "South Africa"[tw] OR "Swaziland"[tw] OR "Zambia"[tw] OR "Zimbabwe"[tw] OR "Benin"[tw] OR "Burkina Faso"[tw] OR "Cote d'Ivoire"[tw] OR "Gambia"[tw] OR "Ghana"[tw] OR "Guinea"[tw] OR "Guinea-Bissau"[tw] OR "Liberia"[tw] OR "Mali"[tw] OR "Mauritania"[tw] OR "Niger"[tw] OR "Nigeria"[tw] OR "Senegal"[tw] OR "Sierra Leone"[tw] OR "Togo"[tw] OR "Algeria"[tw] OR "Egypt"[tw] OR "Libya"[tw] OR "Morocco"[tw] OR "Tunisia"[tw] OR "Comoros"[tw] OR "Madagascar"[tw] OR "Mauritius"[tw] OR "Reunion"[tw] OR "Seychelles"[tw] OR “Cabo Verde”[tw] OR “Kiribati”[tw] OR “Marshall Islands”[tw] OR “Nauru”[tw] OR “Niue”[tw] OR “Sao Tome and Principe”[tw] OR “Solomon Islands”[tw] OR “South Sudan”[tw] OR "province"[tw] OR "provinces"[tw] OR "Developing Countries"[MeSH] OR "Developing"[tw] OR "Least Developed"[tw] OR "Less-Developed"[tw] OR "Less Developed"[tw] OR "Under-Developed"[tw] OR "Under Developed"[tw] OR "UnderDeveloped"[tw] OR "third-world"[tw] OR "third world"[tw] OR "Africa"[MeSH] OR "Africa"[tw] OR "Caribbean Region"[MeSH] OR "Caribbean Region"[tw] OR "West Indies"[tw] OR "Pacific Islands"[MeSH] OR "Pacific Islands"[tw] OR "Micronesia"[tw] OR "Melanesia"[tw] OR "Polynesia"[tw] OR "Mexico"[MeSH] OR "Mexico"[tw] OR "Latin America"[MeSH] OR "Latin America"[tw] OR "South America"[MeSH] OR "South America"[tw] OR "Indian Ocean Islands"[MeSH] OR "Indian Ocean Islands"[tw] OR "Pemba"[tw] OR "Cocos"[tw] OR "Maldives"[tw] OR "Central America"[MeSH] OR "Central America"[tw] OR "Asia"[MeSH] OR "Asia"[tw] OR "far east"[tw] OR “province”[tw] OR “provinces”[tw] OR “district”[tw] OR “districts”[tw] OR “prefecture”[tw] OR “prefectures”[tw] OR “county” OR “counties”[tw] OR “municipality”[tw] OR “municipalities”[tw])))<br>NOT<br>("animals"[MeSH] NOT ("humans"[MeSH] AND "animals"[MeSH]))))<br>NOT<br>("case reports"[pt] OR "editorial"[pt] OR "comment"[pt] OR "practice guideline"[pt])	
IndMed	Infant OR child OR Minors OR infants OR neonate OR neonates OR neonatal OR newborn OR newborns OR child OR children OR youth OR youths OR childhood OR toddler OR toddlers OR kid OR young OR pediatric OR under 5 OR under five OR less than five OR perinatal<br>AND<br>Mortality OR Death OR deaths OR cause of death OR mortalities OR causes of death OR case fatality OR fatal OR fatalities OR fatality<br>AND<br>Diarrhea OR Cholera OR Pneumonia OR Respiratory Infections OR Malaria OR Meningitis OR Encephalitis OR Dysentery OR gastroenteritis OR digestive tract infection OR gastrointestinal infection OR congenital abnormality OR preterm OR asphyxia OR tetanus	January 11, 2018
Global Index Medicus	(tw:("Infant" OR "Infant, Newborn" OR "child" OR "Child, Preschool" OR "Minors" OR "infant" OR "infants" OR "neonate" OR "neonates" OR "neonatal" OR "newborn" OR "newborns" OR "new-born" OR "new-borns" OR "baby" OR "babies" OR "Premature" OR "preterm" OR "pre term" OR "child" OR "children" OR "youth" OR "youths" OR "young people" OR "childhood" OR "toddler" OR "toddlers" OR "kid" OR "kids" OR "young patient" OR "young patients" OR "boy" OR "boys" OR "girl" OR "girls" OR "young age" OR "pediatric" OR "pre-schooler" OR "preschooler" OR "under 5" OR "under five" OR "under fives" OR "less than five" OR "perinatal"))<br>AND<br>(tw:("Mortality" OR "Mortality" OR "Mortalities" OR "fatal" OR "fatality" OR "fatalities" OR "Death" OR "death" OR " deaths" OR "Child Mortality"))<br>AND<br>(tw:("Diarrhea" OR "Diarrhea" OR "Diarrheas" OR "Diarrheal" OR "diarrhoeal" OR "diarrhoea" OR "diarrhoeas" OR "Dysentery" OR "Dysentery" OR "dysenteria" OR "enteritis" OR "enteritides" OR "Cholera" OR "Cholera" OR "Choleras" OR "vibrio Cholerae" OR "Gastroenteritis" OR "gastroenteritis" OR "gastroenteritides" OR "gastro enteritis" OR "gastroduodenitis" OR "gastrointestinal acute infection" OR "gastrointestinal acute infections" OR "gastrointestinal infection" OR "gastrointestinal infections" OR "digestive tract infection" OR "digestive tract infections" OR "digestive infection" OR "gastrointestinal tract infection" OR "gastrointestine tract infection" OR "Pneumonia" OR "pneumonia" OR "pneumonias" OR "pneumonitis" OR "pulmonary inflammation" OR "pulmonary inflammations" OR "lung inflammation" OR "Lung Inflammations" OR "inflammatory lung disease" OR "lobitis" OR "peripneumonia" OR "pleuropneumonia" OR "pleuropneumonitis" OR "pneumonitis" OR "pulmonal inflammation" OR "pulmonary inflammation" OR "pulmonic inflammation" OR "bronchopneumonia" OR "bronchopneumonias" OR "Respiratory Tract Infections" OR "Respiratory Tract Infections" OR "Respiratory Tract Infection" OR "Respiratory Infection" OR "Respiratory Infections" OR "whooping cough" OR "pertussis" OR "pulmonary tract infection" OR "respiration infection" OR "Bronchiolitis" OR " bronchitis" OR "bronchopulmonary infection" OR "Croup" OR "Croup" OR "diphtheria" OR "laryngitis" OR	January 11, 2018

	"Severe Acute Respiratory Syndrome" OR "Severe Acute Respiratory Syndrome" OR "SARS" OR "Acute Chest Syndrome" OR "acute chest syndrome" OR "acute chest syndromes" OR "Acute Lower Respiratory Infection" OR "Acute Lower Respiratory Infections" OR "ALRI" OR "LRI" OR "ARI" OR "respiration tract infection" OR "respiration tract infections" OR "legionnaire disease" OR "legionnaires' disease" OR "legionnaires disease" OR "Pontiac fever" OR "lung infiltrate" OR "lung infiltration" OR "pulmonary infiltrate" OR "pulmonary infiltration" OR "pneumonic lung" OR "rds" OR "respiratory distress" OR "respiration distress" OR "Malaria" OR "malaria" OR "malarias" OR "Plasmodium Infection" OR "Plasmodium Infections" OR "Plasmodium falciparum" OR "Plasmodium falciparum" OR "Plasmodium falciparums" OR "malaria, falciparum" OR "blackwater fever" OR "blackwater fevers" OR "black water fever" OR "black water fevers" OR "remittent fever" OR "paludism" OR "plasmodium infection" OR "plasmodium infections" OR "marsh fever" OR "falciparum infection" OR "Plasmodium vivax" OR "Plasmodium vivax" OR "Plasmodium vivaxs" OR "malaria, vivax" OR "vivax infection" OR "Plasmodium ovale" OR "Plasmodium ovale" OR "Plasmodium ovales" OR "Plasmodium malariae" OR "Plasmodium malariae" OR "Meningitis" OR "Meningitis" OR "Meningitides" OR "Pachymeningitis" OR "Pachymeningitides" OR "Arachnoiditis" OR "Arachnoiditides" OR "Arachnoid Membrane inflammation" OR "Arachnoid Membrane inflammations" OR "Meningoencephalitides" OR "Meningoencephalitis" OR "Meningocephalitis" OR "Cerebromeningitis" OR "Encephalomeningitis" OR "Encephalomeningitides" OR "Haemophilus influenzae" OR "Haemophilus parainfluenzae" OR "Cerebromeningitides" OR "Cerebritis" OR "Cerebritides" OR "Waterhouse Friderichsen Syndrome" OR "Waterhouse Friederichsen Syndrome" OR "Waterhouse-Friderichsen Syndrome" OR "Waterhouse-Friederichsen Syndrome" OR "Purpura Fulminans" OR "Meningococcal" OR "Meningeal" OR "Cerebral Cryptococcosis" OR "Cerebral Cryptococcoses" OR "Toruloma" OR "Torulomas" OR "Lymphocytic Choriomeningitis" OR "Armstrong Syndrome" OR "Armstrong's Syndrome" OR "Encephalitis" "Encephalitis" OR "Encephalitides" OR "encephalitis" OR "brain inflammation" OR "brain inflammations" OR "Rasmussen syndrome" OR "Rasmussen's syndrome" OR "Cerebral Ventriculitis" OR "Cerebral Ventriculitides" OR "Infectious Ventriculitis" OR "Infectious Ventriculitides" OR "Encephalomyelitis" OR "Meningoencephalitides" OR "Meningoencephalitis" OR "Cerebromeningitis" OR "myeloencephalitis" OR "Lupus Vasculitis" OR "Central Nervous System Lupus" OR "Lupus Erythematosis" OR "Lupus Erythematosus" OR "brain vasculitis" OR "brain angiitis" OR "brain arteritis" OR "cerebral arteritis" OR "cerebral vasculitis" OR "Neisseria meningitidis" OR "Neisseria meningitidis" OR "Neisseria meningitides" OR "Micrococcus intracellularis" OR "Neisseria weichselbaumi" OR "Meningococcus" OR "meningococcemia" OR "meningococci" OR "Meningococcal Infections" OR "meningococceal" OR "meningococcaemia" OR "sepsis" OR "sepsis" OR "Pyemia" OR "Pyemias" OR "Pyohemia" OR "Pyohemias" OR "Pyaemia" OR "Pyaemias" OR "Septicemia" OR "Septicemias" OR "Blood Poisoning" OR "Blood Poisonings" OR "septic" OR "sepses" OR "congenital abnormalities" OR "congenital" OR "Deformities" OR "Deformity" OR "Birth Defects" OR "Birth Defect" OR "abnormality" OR "abnormalities" OR "malformation" OR "malformations" OR "malformative" OR "malformed" OR "neural tube defects" OR "neural tube" OR "Craniorachischisis" OR "Craniorachischises" OR "Diastematomyelia" OR "Diastematomyelias" OR "Tethered Cord" OR "Spinal Dysraphism" OR "Tethered Spinal Cord" OR "Spinal Dysraphisms" OR "Iniencephaly" OR "Iniencephalies" OR "Neurenteric" OR "Neuroenteric" OR "Spinal Cord Myelodysplasia" OR "Spinal Cord Myelodysplasias" OR "Acrania" OR "Acranias" OR "Exencephaly" OR "Exencephalies" OR "dysgraphia" OR "dysraphic" OR "dysraphism" OR "dysraphy" OR "NTD" OR "NTDS" OR "sudden infant death syndrome" OR "sudden infant death" OR "sid" OR "SIDS" OR "cot death" OR "cot deaths" OR "crib death" OR "fetal alcohol spectrum disorders" OR "Fetal alcohol spectrum" OR "Foetal alcohol spectrum" OR "FASD" OR "FASDs" OR "Fetal Alcohol Syndrome" OR "Alcohol-Related Birth Defects" OR "Alcohol Related Birth Defects" OR "Alcohol Related Neurodevelopmental Disorder" OR "FAE" OR "Fetal Alcohol Effects" OR "FAEs" OR "Fetal Alcohol Syndrome" OR "Fetus Alcohol Syndrome" OR "Foetal alcohol syndrome" OR "premature birth" OR "premature" OR "preterm" OR "pre-mature" OR "pre-term" OR "prematurity" OR "prematuritas" OR "asphyxia" OR "asphyxia" OR "Asphyxia Neonatorum" OR "Asphyxia Neonatorum" OR "hypoxia" OR "Asphyxias" OR "Suffocation" OR "Suffocations" OR "anoxia" OR ("intrapartum" AND "related") OR "tetanus" OR "tetanus" OR "tetani" OR "diphtheria-tetanus-pertussis" OR "rubella" OR "rubella" OR "measles" OR "epidemic roseola" OR "necrosis" OR "necrosis" OR "necrotic" OR "necroses" OR "necrotizing" OR "Infarction" OR "infractions" OR "birth injuries" OR "birth injuries" OR "birth injury" OR "birth trauma" OR "Obstetric Paralyses" OR "Obstetric Paralysis" OR "Obstetrical Paralyses" OR "Obstetrical Paralysis" OR "birth damage" OR "birth lesion" OR "birth palsy" OR "birth paralysis" OR "obstetric palsy" OR "obstetrical palsy" OR "brachial palsy" OR "brachial paralysis" OR "brachial plexus paralysis" OR "delivery trauma" OR "arm paralysis" OR "paralysis brachialis" OR "jaundice" OR "jaundice" OR "jaundices" OR "Icterus" OR "bronze baby syndrome" OR "erythroleukoblastosis" OR "hemorrhage" OR "hemorrhage" "haemorrhage" OR "haemorrhagic" OR "Hemorrhagic" OR "Nutrition disorders" OR "malnutrition" OR "Nutritional" OR "Undernutrition" OR "Malnourishment" OR " Malnourishments" OR "Deficiency" OR "Deficiencies" OR "Refeeding Syndrome" OR "Starvation" OR "Starvations" OR "Famine" OR "Famines" OR "deficient" OR "underfeeding" OR "undernourishment" OR "nutrition" OR "Hypervitaminosis" OR "Noncommunicable Diseases" OR "Noncommunicable" OR "Non-communicable" OR "communicable" OR "non-infectious" OR "infectious" OR "noninfectious" OR "Communicable Diseases" OR "Infant, Newborn, Diseases" OR "newborn" OR "neonatal" OR "perinatal"))<br>AND<br>(tw:("Argentina" OR "Bolivia" OR "Brazil" OR "Brasil" OR "Chile" OR "Colombia" OR "Ecuador" OR "French Guiana" OR "Guyana" OR "Paraguay" OR "Peru" OR "Suriname" OR "Uruguay" OR "Venezuela" OR "Mexico" OR "Belize" OR "Costa Rica" OR "El Salvador" OR "Guatemala" OR "Honduras" OR "Nicaragua" OR "Puerto Rico" OR "Panama" OR "West Indies" OR "Antigua" OR "Barbuda" OR "Bahamas" OR "Barbados" OR "Cuba" OR "Dominica" OR "Dominican Republic"	

	OR "Grenada" OR "Guadeloupe" OR "Haiti" OR "Jamaica" OR "Martinique" OR "Antilles" OR "Anguilla" OR "Saint Kitts" OR "St Kitts" OR "Saint Lucia" OR "St Lucia" OR "Saint Vincent" OR "St Vincent" OR "Trinidad " OR "Tobago" OR "Virgin Islands" OR "Kazakhstan" OR "Kyrgyzstan" OR "Tajikistan" OR "Turkmenistan" OR "Uzbekistan" OR "Borneo" OR "Brunei" OR "Cambodia" OR "East Timor" OR "Indonesia" OR "Laos" OR "Malaysia" OR "Mekong Valley" OR "Myanmar" OR "Burma" OR "Philippines" OR "Singapore" OR "Thailand" OR "Vietnam" OR "Bangladesh" OR "Bhutan" OR "India" OR "Nepal" OR "Pakistan" OR "Sri Lanka" OR "China" OR "Korea" OR "Macao" OR "Macau" OR "Mongolia" OR "Taiwan" OR "Afghanistan" OR "Bahrain" OR "Iran" OR "Iraq" OR "Israel" OR "Jordan" OR "Kuwait" OR "Lebanon" OR "Oman" OR "Qatar" OR "Saudi Arabia" OR "Syria" OR "Turkey" OR "United Arab Emirates" OR "Yemen" OR "Fiji" OR "New Caledonia" OR "Papua New Guinea" OR "Vanuatu" OR "Micronesia" OR "Melanesia" OR "Guam" OR "Palau" OR "Polynesia" OR "Samoa" OR "Tonga" OR "Armenia" OR "Azerbaijan" OR "Georgia" OR "Albania" OR "Estonia" OR "Latvia" OR "Lithuania" OR "Bosnia" OR "Herzegovina" OR "Serbia" OR "Bulgaria" OR "Belarus" OR "Croatia" OR "Czech Republic" OR "Hungary" OR "Macedonia" OR "Moldova" OR "Montenegro" OR "Poland" OR "Romania" OR "Russia" OR "Bashkiria" OR "Dagestan" OR "Slovakia" OR "Slovenia" OR "Ukraine" OR "Cameroon" OR "Central African Republic" OR "Chad" OR "Congo" OR "Equatorial Guinea" OR "Gabon" OR "Burundi" OR "Djibouti" OR "Eritrea" OR "Ethiopia" OR "Kenya" OR "Rwanda" OR "Somalia" OR "Sudan" OR "Tanzania" OR "Uganda" OR "Angola" OR "Botswana" OR "Lesotho" OR "Malawi" OR "Mozambique" OR "Namibia" OR "South Africa" OR "Swaziland" OR "Zambia" OR "Zimbabwe" OR "Benin" OR "Burkina Faso" OR "Cote d'Ivoire" OR "Gambia" OR "Ghana" OR "Guinea" OR "Guinea-Bissau" OR "Liberia" OR " Mali" OR "Mauritania" OR "Niger" OR "Nigeria" OR "Senegal" OR "Sierra Leone" OR "Togo" OR "Algeria" OR "Egypt" OR "Libya" OR "Morocco" OR "Tunisia" OR "Comoros" OR "Madagascar" OR "Mauritius" OR "Reunion" OR "Seychelles" OR "Cabo Verde" OR "Kiribati" OR "Marshall Islands" OR "Nauru" OR "Niue" OR "Sao Tome" OR "Solomon Island" OR "South Sudan" OR "Developing Countries" OR "Developing" OR "Least Developed" OR "Less-Developed" OR "Less Developed" OR "Under-Developed" OR "Under Developed" OR "UnderDeveloped" OR "third-world" OR "third world" OR "Africa" OR "Africa" OR "Caribbean Region" OR "Caribbean Region" OR "West Indies" OR "Pacific Islands" OR "Pacific Islands" OR "Micronesia" OR "Melanesia" OR "Polynesia" OR "Mexico" OR "Mexico" OR "Latin America" OR "Latin America" OR "South America" OR "South America" OR "Indian Ocean Islands" OR "Indian Ocean Islands" OR "Pemba" OR "Cocos" OR "Maldives" OR "Central America" OR "Central America" OR "Asia" OR "Asia" OR "far east" OR "eastern europe" OR "eastern europe" OR "province" OR "provinces" OR "district" OR "districts" OR "prefecture" OR "prefectures" OR "county" OR "counties" OR "municipality" OR "municipalities"))<br>AND<br>(db:("LILACS" OR "IMSEAR" OR "WPRIM" OR "IMEMR" OR "WHOLIS" OR "AIM"))<br>AND<br>(instance:"ghl")	
Embase	(((('child'/exp OR 'minor (person)'/exp OR 'infant':ab,ti OR 'infants':ab,ti OR 'neonate':ab,ti OR 'neonates':ab,ti OR 'neonatal':ab,ti OR 'newborn':ab,ti OR 'newborns':ab,ti OR 'new-born':ab,ti OR 'new-borns':ab,ti OR 'baby':ab,ti OR 'babies':ab,ti OR 'premature':ab,ti OR 'preterm':ab,ti OR 'pre term':ab,ti OR 'child':ab,ti OR 'children':ab,ti OR 'youth':ab,ti OR 'youths':ab,ti OR 'young people':ab,ti OR 'childhood':ab,ti OR 'toddler':ab,ti OR 'toddlers':ab,ti OR 'kid':ab,ti OR 'kids':ab,ti OR 'young patient':ab,ti OR 'young patients':ab,ti OR 'boy':ab,ti OR 'boys':ab,ti OR 'girl':ab,ti OR 'girls':ab,ti OR 'young age':ab,ti OR 'pediatric':ab,ti OR 'pre-schooler':ab,ti OR 'preschooler':ab,ti OR 'under 5':ab,ti OR 'under five':ab,ti OR 'under fives':ab,ti OR 'less than five':ab,ti OR 'perinatal':ab,ti)<br>AND<br>(('mortality'/exp OR 'death'/exp OR 'mortality':ab,ti OR 'death':ab,ti OR 'deaths':ab,ti OR 'mortalities':ab,ti OR 'case fatality':ab,ti OR 'fatal':ab,ti OR 'fatalities':ab,ti OR 'fatality':ab,ti)<br>AND<br>((('diarrhea'/exp OR 'dysentery'/exp OR 'cholera'/exp OR 'gastroenteritis'/exp OR 'diarrhea':ab,ti OR 'diarrheas':ab,ti OR 'diarrheal':ab,ti OR 'diarrhoeal':ab,ti OR 'diarrhoea':ab,ti OR 'diarrhoeas':ab,ti OR 'dysentery':ab,ti OR 'dysenteria':ab,ti OR 'enteritis':ab,ti OR 'enteritides':ab,ti OR 'cholera':ab,ti OR 'choleras':ab,ti OR 'vibrio cholerae':ab,ti OR 'gastroenteritis':ab,ti OR 'gastroenteritides':ab,ti OR 'gastro enteritis':ab,ti OR 'gastroduodenitis':ab,ti OR 'gastrointestinal acute infection':ab,ti OR 'gastrointestinal acute infections':ab,ti OR 'gastrointestinal infection':ab,ti OR 'gastrointestinal infections':ab,ti OR 'digestive tract infection':ab,ti OR 'digestive tract infections':ab,ti OR 'digestive infection':ab,ti OR 'gastrointestinal tract infection':ab,ti OR 'gastrointestine tract infection':ab,ti OR 'pneumonia'/exp OR 'respiratory tract infection'/exp OR 'bronchitis'/exp OR 'croup'/exp OR 'pneumonia':ab,ti OR 'pneumonias':ab,ti OR 'pulmonary inflammations':ab,ti OR 'lung inflammation':ab,ti OR 'lung inflammations':ab,ti OR 'inflammatory lung disease':ab,ti OR 'lobitis':ab,ti OR 'peripneumonia':ab,ti OR 'pleuropneumonia':ab,ti OR 'pleuropneumonitis':ab,ti OR 'pneumonitis':ab,ti OR 'pulmonal inflammation':ab,ti OR 'pulmonary inflammation':ab,ti OR 'pulmonic inflammation':ab,ti OR 'bronchopneumonia':ab,ti OR 'bronchopneumonias':ab,ti OR 'respiratory tract infections':ab,ti OR 'respiratory tract infection':ab,ti OR 'respiratory infection':ab,ti OR 'respiratory infections':ab,ti OR 'whooping cough':ab,ti OR 'pertussis':ab,ti OR 'pulmonary tract infection':ab,ti OR 'respiration infection':ab,ti OR 'bronchiolitis':ab,ti OR 'bronchitis':ab,ti OR 'bronchopulmonary infection':ab,ti OR 'croup':ab,ti OR 'diphtheria':ab,ti OR 'laryngitis':ab,ti OR 'severe acute respiratory syndrome':ab,ti OR 'sars':ab,ti OR 'acute chest syndrome':ab,ti OR 'acute chest	January 12, 2018

	syndromes':ab,ti OR 'acute lower respiratory infection':ab,ti OR 'acute lower respiratory infections':ab,ti OR 'alri':ab,ti OR 'lri':ab,ti OR 'ari':ab,ti OR 'respiration tract infection':ab,ti OR 'respiration tract infections':ab,ti OR 'legionnaire disease':ab,ti OR 'legionnaires disease':ab,ti OR 'pontiac fever':ab,ti OR 'lung infiltrate':ab,ti OR 'lung infiltration':ab,ti OR 'pulmonary infiltrate':ab,ti OR 'pulmonary infiltration':ab,ti OR 'pneumonic lung':ab,ti OR 'rds':ab,ti OR 'respiratory distress':ab,ti OR 'respiration distress':ab,ti OR 'malaria'/exp OR 'plasmodium falciparum'/exp OR 'plasmodium vivax'/exp OR 'plasmodium malariae'/exp OR 'plasmodium ovale'/exp OR 'malaria':ab,ti OR 'malarias':ab,ti OR 'plasmodium falciparum':ab,ti OR 'plasmodium falciparums':ab,ti OR 'plasmodium vivax':ab,ti OR 'plasmodium vivaxs':ab,ti OR 'plasmodium vivax malaria':ab,ti OR 'p. falciparum infection':ab,ti OR 'p. falciparum malaria':ab,ti OR 'pernicious malaria':ab,ti OR 'plasmodium falciparum infection':ab,ti OR 'plasmodium falciparum malaria vivax':ab,ti OR 'p. vivax infection':ab,ti OR 'p. vivax malaria':ab,ti OR 'vivax infection':ab,ti OR 'vivax malaria':ab,ti OR 'blackwater fever':ab,ti OR 'blackwater fevers':ab,ti OR 'black water fever':ab,ti OR 'black water fevers':ab,ti OR 'remittent fever':ab,ti OR 'paludism':ab,ti OR 'plasmodium infection':ab,ti OR 'plasmodium infections':ab,ti OR 'marsh fever':ab,ti OR 'falciparum infection':ab,ti OR 'plasmodium ovale':ab,ti OR 'plasmodium ovales':ab,ti OR 'plasmodium malariae':ab,ti OR 'meningitis'/exp OR 'neisseria meningitidis'/exp OR 'meningococcemia'/exp OR 'encephalitis'/exp OR 'sepsis'/exp OR 'meningococcosis'/exp OR 'meningitis':ab,ti OR 'meningitides':ab,ti OR 'pachymeningitis':ab,ti OR 'pachymeningitides':ab,ti OR 'arachnoiditis':ab,ti OR 'arachnoiditides':ab,ti OR 'arachnoid membrane inflammation':ab,ti OR 'arachnoid membrane inflammations':ab,ti OR 'meningocephalitis':ab,ti OR 'encephalomeningitis':ab,ti OR 'encephalomeningitides':ab,ti OR 'haemophilus influenzae':ab,ti OR 'haemophilus parainfluenzae':ab,ti OR 'cerebromeningitides':ab,ti OR 'cerebritis':ab,ti OR 'cerebritides':ab,ti OR 'waterhouse friderichsen syndrome':ab,ti OR 'waterhouse friederichsen syndrome':ab,ti OR 'waterhouse-friderichsen syndrome':ab,ti OR 'waterhouse-friederichsen syndrome':ab,ti OR 'purpura fulminans':ab,ti OR 'meningococcal':ab,ti OR 'meningeal':ab,ti OR 'cerebral cryptococcosis':ab,ti OR 'cerebral cryptococcoses':ab,ti OR 'toruloma':ab,ti OR 'torulomas':ab,ti OR 'lymphocytic choriomeningitis':ab,ti OR 'armstrong syndrome':ab,ti OR 'neisseria meningitidis':ab,ti OR 'neisseria meningitides':ab,ti OR 'encephalitis':ab,ti OR 'encephailitis':ab,ti OR 'encephalitides':ab,ti OR 'epidemic meningitis':ab,ti OR 'n. meningitidis':ab,ti OR 'bacterial meningitis':ab,ti OR 'meningitis, meningococcal':ab,ti OR 'meningococcal infections':ab,ti OR 'meningitis, pneumococcal':ab,ti OR 'brain inflammation':ab,ti OR 'brain inflammations':ab,ti OR 'rasmussen syndrome':ab,ti OR 'cerebral ventriculitis':ab,ti OR 'cerebral ventriculitides':ab,ti OR 'infectious ventriculitis':ab,ti OR 'infectious ventriculitides':ab,ti OR 'encephalomyelitis':ab,ti OR 'meningoencephalitides':ab,ti OR 'meningoencephalitis':ab,ti OR 'cerebromeningitis':ab,ti OR 'myeloencephalitis':ab,ti OR 'lupus vasculitis':ab,ti OR 'central nervous system lupus':ab,ti OR 'lupus erythematosis':ab,ti OR 'lupus erythematosus':ab,ti OR 'brain vasculitis':ab,ti OR 'brain angiitis':ab,ti OR 'brain arteritis':ab,ti OR 'cerebral arteritis':ab,ti OR 'cerebral vasculitis':ab,ti OR 'micrococcus intracellularis':ab,ti OR 'neisseria weichselbaumi':ab,ti OR 'meningococcus':ab,ti OR 'meningococcemia':ab,ti OR 'meningococci':ab,ti OR 'meningococceal':ab,ti OR 'meningococcaemia':ab,ti OR 'sepsis':ab,ti OR 'pyemia':ab,ti OR 'pyemias':ab,ti OR 'pyohemia':ab,ti OR 'pyohemias':ab,ti OR 'pyaemia':ab,ti OR 'pyaemias':ab,ti OR 'septicemia':ab,ti OR 'septicemias':ab,ti OR 'blood poisoning':ab,ti OR 'blood poisonings':ab,ti OR 'septic':ab,ti OR 'sepses':ab,ti OR 'congenital disorder'/exp OR 'neural tube defect'/exp OR 'sudden infant death syndrome'/exp OR 'fetal alcohol syndrome'/exp OR 'congenital':ab,ti OR 'deformities':ab,ti OR 'deformity':ab,ti OR 'birth defects':ab,ti OR 'birth defect':ab,ti OR 'abnormality':ab,ti OR 'abnormalities':ab,ti OR 'malformation':ab,ti OR 'malformations':ab,ti OR 'malformative':ab,ti OR 'malformed':ab,ti OR 'neural tube':ab,ti OR 'craniorachischisis':ab,ti OR 'craniorachischises':ab,ti OR 'diastematomyelia':ab,ti OR 'diastematomyelias':ab,ti OR 'tethered cord':ab,ti OR 'spinal dysraphism':ab,ti OR 'tethered spinal cord':ab,ti OR 'spinal dysraphisms':ab,ti OR 'iniencephaly':ab,ti OR 'iniencephalies':ab,ti OR 'neurenteric':ab,ti OR 'neuroenteric':ab,ti OR 'spinal cord myelodysplasia':ab,ti OR 'spinal cord myelodysplasias':ab,ti OR 'acrania':ab,ti OR 'acranias':ab,ti OR 'exencephaly':ab,ti OR 'exencephalies':ab,ti OR 'dysgraphia':ab,ti OR 'dysraphic':ab,ti OR 'dysraphism':ab,ti OR 'dysraphy':ab,ti OR 'ntd':ab,ti OR 'ntds':ab,ti OR 'sudden infant death':ab,ti OR 'sid':ab,ti OR 'sids':ab,ti OR 'cot death':ab,ti OR 'cot deaths':ab,ti OR 'crib death':ab,ti OR 'fetal alcohol spectrum':ab,ti OR 'foetal alcohol spectrum':ab,ti OR 'fasd':ab,ti OR 'fasds':ab,ti OR 'alcohol-related birth defects':ab,ti OR 'alcohol related birth defects':ab,ti OR 'alcohol related neurodevelopmental disorder':ab,ti OR 'fae':ab,ti OR 'fetal alcohol effects':ab,ti OR 'faes':ab,ti OR 'fetal alcohol syndrome':ab,ti OR 'fetus alcohol syndrome':ab,ti OR 'foetal alcohol syndrome':ab,ti OR 'immature and premature labor'/exp OR 'premature':ab,ti OR 'preterm':ab,ti OR 'pre-mature':ab,ti OR 'pre-term':ab,ti OR 'prematurity':ab,ti OR 'prematuritas':ab,ti OR 'asphyxia'/exp OR 'newborn hypoxia'/exp OR 'suffocation'/exp OR 'anoxia'/exp OR 'asphyxia neonatorum':ab,ti OR 'hypoxia':ab,ti OR 'asphyxia':ab,ti OR 'asphyxias':ab,ti OR 'suffocation':ab,ti OR 'suffocations':ab,ti OR 'anoxia':ab,ti OR ('intrapartum':ab,ti AND 'related':ab,ti) OR 'tetanus'/exp OR 'tetanus':ab,ti OR 'tetani':ab,ti OR 'diphtheria-tetanus-pertussis':ab,ti OR 'rubella'/exp OR 'necrosis'/exp OR 'birth injuries'/exp OR 'bleeding'/exp OR 'jaundice'/exp OR 'rubella':ab,ti OR 'measles':ab,ti OR 'epidemic roseola':ab,ti OR 'necrosis':ab,ti OR 'necrotic':ab,ti OR 'necroses':ab,ti OR 'necrotizing':ab,ti OR 'infarction':ab,ti OR 'infractions':ab,ti OR 'birth injuries':ab,ti OR 'birth injury':ab,ti OR 'birth trauma':ab,ti OR 'obstetric paralyses':ab,ti OR 'obstetric paralysis':ab,ti OR 'obstetrical paralyses':ab,ti OR 'obstetrical paralysis':ab,ti OR 'birth damage':ab,ti OR 'birth lesion':ab,ti OR 'birth palsy':ab,ti OR 'birth paralysis':ab,ti OR 'obstetric palsy':ab,ti OR 'obstetrical palsy':ab,ti OR 'brachial palsy':ab,ti OR 'brachial paralysis':ab,ti OR 'brachial plexus paralysis':ab,ti OR 'delivery trauma':ab,ti OR 'arm paralysis':ab,ti OR 'paralysis brachialis':ab,ti OR 'jaundice':ab,ti OR 'jaundices':ab,ti OR 'icterus':ab,ti OR 'bronze baby syndrome':ab,ti OR 'erythroleukoblastosis':ab,ti OR 'hemorrhage':ab,ti OR 'haemorrhage':ab,ti OR 'haemorrhagic':ab,ti OR 'hemorrhagic':ab,ti OR 'nutritional disorder'/exp OR 'starvation'/exp OR 'hunger'/exp) AND ('malnutrition':ab,ti OR 'nutritional':ab,ti OR 'undernutrition':ab,ti OR 'malnourishment':ab,ti OR	

	'malnourishments':ab,ti OR 'deficiency':ab,ti OR 'deficiencies':ab,ti OR 'refeeding syndrome':ab,ti OR 'starvation':ab,ti OR 'starvations':ab,ti OR 'famine':ab,ti OR 'famines':ab,ti OR 'deficient':ab,ti OR 'underfeeding':ab,ti OR 'undernourishment':ab,ti OR 'nutrition':ab,ti OR 'hypervitaminosis':ab,ti) OR 'chronic disease'/exp OR 'communicable disease'/exp OR 'infection'/exp OR 'non communicable disease'/exp OR 'infant disease'/exp OR 'noncommunicable':ab,ti OR 'non-communicable':ab,ti OR 'communicable':ab,ti OR 'non-infectious':ab,ti OR 'infectious':ab,ti OR 'noninfectious':ab,ti OR 'newborn':ab,ti OR 'neonatal':ab,ti OR 'perinatal':ab,ti))<br>AND<br>('argentina':ab,ti OR 'bolivia':ab,ti OR 'brazil':ab,ti OR 'brasil':ab,ti OR 'chile':ab,ti OR 'colombia':ab,ti OR 'ecuador':ab,ti OR 'french guiana':ab,ti OR 'guyana':ab,ti OR 'paraguay':ab,ti OR 'peru':ab,ti OR 'suriname':ab,ti OR 'uruguay':ab,ti OR 'venezuela':ab,ti OR 'belize':ab,ti OR 'costa rica':ab,ti OR 'el salvador':ab,ti OR 'guatemala':ab,ti OR 'honduras':ab,ti OR 'nicaragua':ab,ti OR 'puerto rico':ab,ti OR 'panama':ab,ti OR 'antigua':ab,ti OR 'barbuda':ab,ti OR 'bahamas':ab,ti OR 'barbados':ab,ti OR 'cuba':ab,ti OR 'dominica':ab,ti OR 'dominican republic':ab,ti OR 'grenada':ab,ti OR 'guadeloupe':ab,ti OR 'haiti':ab,ti OR 'jamaica':ab,ti OR 'martinique':ab,ti OR 'antilles':ab,ti OR 'anguilla':ab,ti OR 'saint kitts':ab,ti OR 'st kitts':ab,ti OR 'saint lucia':ab,ti OR 'st lucia':ab,ti OR 'saint vincent':ab,ti OR 'st vincent':ab,ti OR 'trinidad':ab,ti OR 'tobago':ab,ti OR 'virgin islands':ab,ti OR 'kazakhstan':ab,ti OR 'kyrgyzstan':ab,ti OR 'tajikistan':ab,ti OR 'turkmenistan':ab,ti OR 'uzbekistan':ab,ti OR 'borneo':ab,ti OR 'brunei':ab,ti OR 'cambodia':ab,ti OR 'east timor':ab,ti OR 'indonesia':ab,ti OR 'laos':ab,ti OR 'malaysia':ab,ti OR 'mekong valley':ab,ti OR 'myanmar':ab,ti OR 'burma':ab,ti OR 'philippines':ab,ti OR 'singapore':ab,ti OR 'thailand':ab,ti OR 'vietnam':ab,ti OR 'bangladesh':ab,ti OR 'bhutan':ab,ti OR 'india':ab,ti OR 'nepal':ab,ti OR 'pakistan':ab,ti OR 'sri lanka':ab,ti OR 'china':ab,ti OR 'korea':ab,ti OR 'macao':ab,ti OR 'macau':ab,ti OR 'mongolia':ab,ti OR 'taiwan':ab,ti OR 'afghanistan':ab,ti OR 'bahrain':ab,ti OR 'iran':ab,ti OR 'iraq':ab,ti OR 'israel':ab,ti OR 'jordan':ab,ti OR 'kuwait':ab,ti OR 'lebanon':ab,ti OR 'oman':ab,ti OR 'qatar':ab,ti OR 'saudi arabia':ab,ti OR 'syria':ab,ti OR 'turkey':ab,ti OR 'united arab emirates':ab,ti OR 'yemen':ab,ti OR 'fiji':ab,ti OR 'new caledonia':ab,ti OR 'papua new guinea':ab,ti OR 'vanuatu':ab,ti OR 'guam':ab,ti OR 'palau':ab,ti OR 'samoa':ab,ti OR 'tonga':ab,ti OR 'armenia':ab,ti OR 'azerbaijan':ab,ti OR 'georgia':ab,ti OR 'albania':ab,ti OR 'estonia':ab,ti OR 'latvia':ab,ti OR 'lithuania':ab,ti OR 'bosnia':ab,ti OR 'herzegovina':ab,ti OR 'serbia':ab,ti OR 'bulgaria':ab,ti OR 'belarus':ab,ti OR 'croatia':ab,ti OR 'czech republic':ab,ti OR 'hungary':ab,ti OR 'macedonia':ab,ti OR 'moldova':ab,ti OR 'montenegro':ab,ti OR 'poland':ab,ti OR 'romania':ab,ti OR 'russia':ab,ti OR 'bashkiria':ab,ti OR 'dagestan':ab,ti OR 'slovakia':ab,ti OR 'slovenia':ab,ti OR 'ukraine':ab,ti OR 'cameroon':ab,ti OR 'central african republic':ab,ti OR 'chad':ab,ti OR 'congo':ab,ti OR 'equatorial guinea':ab,ti OR 'gabon':ab,ti OR 'burundi':ab,ti OR 'djibouti':ab,ti OR 'eritrea':ab,ti OR 'ethiopia':ab,ti OR 'kenya':ab,ti OR 'rwanda':ab,ti OR 'somalia':ab,ti OR 'sudan':ab,ti OR 'tanzania':ab,ti OR 'uganda':ab,ti OR 'angola':ab,ti OR 'botswana':ab,ti OR 'lesotho':ab,ti OR 'malawi':ab,ti OR 'mozambique':ab,ti OR 'namibia':ab,ti OR 'south africa':ab,ti OR 'swaziland':ab,ti OR 'zambia':ab,ti OR 'zimbabwe':ab,ti OR 'benin':ab,ti OR 'burkina faso':ab,ti OR 'cote d`ivoire':ab,ti OR 'gambia':ab,ti OR 'ghana':ab,ti OR 'guinea':ab,ti OR 'guinea-bissau':ab,ti OR 'liberia':ab,ti OR 'mali':ab,ti OR 'mauritania':ab,ti OR 'niger':ab,ti OR 'nigeria':ab,ti OR 'senegal':ab,ti OR 'sierra leone':ab,ti OR 'togo':ab,ti OR 'algeria':ab,ti OR 'egypt':ab,ti OR 'libya':ab,ti OR 'morocco':ab,ti OR 'tunisia':ab,ti OR 'comoros':ab,ti OR 'madagascar':ab,ti OR 'mauritius':ab,ti OR 'reunion':ab,ti OR 'seychelles':ab,ti OR 'cabo verde':ab,ti OR 'kiribati':ab,ti OR 'marshall islands':ab,ti OR 'nauru':ab,ti OR 'niue':ab,ti OR 'sao tome and principe':ab,ti OR 'solomon islands':ab,ti OR 'south sudan':ab,ti OR 'developing country'/exp OR 'africa'/exp OR 'caribbean islands'/exp OR 'south and central america'/exp OR 'mexico'/exp OR 'asia'/exp OR 'pacific islands'/exp OR 'indian ocean'/exp OR 'eastern europe'/exp OR 'developing':ab,ti OR 'least developed':ab,ti OR 'less-developed':ab,ti OR 'less developed':ab,ti OR 'under-developed':ab,ti OR 'under developed':ab,ti OR 'underdeveloped':ab,ti OR 'third-world':ab,ti OR 'third world':ab,ti OR 'africa':ab,ti OR 'caribbean region':ab,ti OR 'west indies':ab,ti OR 'pacific islands':ab,ti OR 'micronesia':ab,ti OR 'melanesia':ab,ti OR 'polynesia':ab,ti OR 'mexico':ab,ti OR 'latin america':ab,ti OR 'south america':ab,ti OR 'indian ocean islands':ab,ti OR 'pemba':ab,ti OR 'cocos':ab,ti OR 'maldives':ab,ti OR 'central america':ab,ti OR 'asia':ab,ti OR 'far east':ab,ti OR 'eastern europe':ab,ti OR 'province':ab,ti OR 'provinces':ab,ti OR 'district':ab,ti OR 'districts':ab,ti OR 'prefecture':ab,ti OR 'prefectures':ab,ti OR 'county':ab,ti OR 'counties':ab,ti OR 'municipality':ab,ti OR 'municipalities':ab,ti)))<br>NOT<br>('nonhuman':de OR 'animal experiment':de) NOT ('human' AND ('nonhuman':de OR 'animal experiment':de)))<br>NOT<br>'editorial':it OR 'letter':it OR 'note':it OR 'case report':de OR 'practice guideline':de OR 'methodology':de)	
Global Health Ovid	1 exp children/ or exp infants/ or infant.mp. or infants.mp. or neonate.mp. or neonates.mp. or neonatal.mp. or<br>newborn.mp. or newborns.mp. or new-born.mp. or new-borns.mp. or baby.mp. or babies.mp. or premature.mp. or preterm.mp.<br>or pre term.mp. or child.mp. or children.mp. or youth.mp. or youths.mp. or young people.mp. or childhood.mp. or toddler.mp. or toddlers.mp. or kid.mp. or kids.mp. or young patient.mp. or young patients.mp. or boy.mp. or boys.mp. or girl.mp. or girls.mp. or young age.mp. or pediatric.mp. or pre-schooler.mp. or preschooler.mp. or under 5.mp. or under five.mp. or under fives.mp. or less than five.mp. or perinatal.mp. [mp=abstract, title, original title, broad terms, heading words, identifiers, cabicodes] (507817)<br>2 (exp death/ and dying/) or exp mortality/ or mortality.mp. or mortalities.mp. or fatal.mp. or fatality.mp. or	January 12, 2018

	fatalities.mp. or death.mp. or deaths.mp. [mp=abstract, title, original title, broad terms, heading words, identifiers, cabicodes] (283929)<br>3 exp diarrhoea/ or exp dysentery/ or exp enterocolitis/ or exp gastroenteritis/ or exp cholera/ or Diarrhea.mp. or Diarrheas.mp. or Diarrheal.mp. or diarrhoeal.mp. or diarrhoea.mp. or diarrhoeas.mp. or dysentery.mp. or dysenteria.mp.<br>or enteritis.mp. or enteritides.mp. or Cholera.mp. or Choleras.mp. or vibrio Cholerae.mp. or gastroenteritis.mp. or gastroenteritides.mp. or gastro enteritis.mp. or gastroduodenitis.mp. or gastrointestinal acute infection.mp. or gastrointestinal acute infections.mp. or gastrointestinal infection.mp. or gastrointestinal infections.mp. or digestive tract infection.mp. or digestive tract infections.mp. or digestive infection.mp. or gastrointestinal tract infection.mp.<br>or gastrointestine tract infection.mp. or exp pneumonia/ or exp pneumonitis/ or exp lower respiratory tract infections/ or exp upper respiratory tract infections/ or exp pertussis/ or exp bronchiolitis/ or exp bronchitis/ or exp diphtheria/ or exp laryngitis/ or exp legionnaires' disease/ or exp acute respiratory distress syndrome/ or pneumonia.mp. or pneumonias.mp. or pneumonitis.mp. or pulmonary inflammation.mp. or pulmonary inflammations.mp. or lung inflammation.mp.<br>or Lung Inflammations.mp. or inflammatory lung disease.mp. or lobitis.mp. or peripneumonia.mp. or pleuropneumonia.mp. or pleuropneumonitis.mp. or pneumonitis.mp. or pulmonal inflammation.mp. or pulmonary inflammation.mp. or pulmonic inflammation.mp. or bronchopneumonia.mp. or bronchopneumonias.mp. or Respiratory Tract Infections.mp. or Respiratory Tract Infection.mp. or Respiratory Infection.mp. or Respiratory Infections.mp. or whooping cough.mp. or pertussis.mp. or pulmonary tract infection.mp. or respiration infection.mp. or Bronchiolitis.mp. or bronchitis.mp. or bronchopulmonary infection.mp. or Croup.mp. or diphtheria.mp. or laryngitis.mp. or Severe Acute Respiratory Syndrome.mp. or SARS.mp. or acute chest syndrome.mp. or acute chest syndromes.mp. or Acute Lower Respiratory Infection.mp. or Acute Lower Respiratory Infections.mp. or ALRI.mp. or LRI.mp. or ARI.mp. or respiration tract infection.mp. or respiration tract infections.mp. or legionnaire disease.mp. or legionnaires disease.mp. or Pontiac fever.mp. or lung infiltrate.mp. or lung infiltration.mp. or pulmonary infiltrate.mp. or pulmonary infiltration.mp. or pneumonic lung.mp. or rds.mp. or respiratory distress.mp. or respiration distress.mp. or exp malaria/ or exp plasmodium falciparum/ or exp Plasmodium vivax/ or exp Plasmodium ovale/ or exp Plasmodium malariae/ or malaria.mp. or malarias.mp. or Plasmodium Infection.mp.<br>or Plasmodium Infections.mp. or Plasmodium falciparum.mp. or Plasmodium falciparums.mp. or malaria, falciparum.mp. or blackwater fever.mp. or blackwater fevers.mp. or black water fever.mp. or black water fevers.mp. or remittent fever.mp.<br>or paludism.mp. or plasmodium infection.mp. or plasmodium infections.mp. or marsh fever.mp. or falciparum infection.mp.<br>or Plasmodium vivax.mp. or Plasmodium vivaxs.mp. or vivax malaria.mp. or vivax infection.mp. or Plasmodium ovale.mp. or Plasmodium ovales.mp. or ovale malaria.mp. or Plasmodium malariae.mp. or exp meningitis/ or exp meningococcal disease/ or exp encephalitis/ or exp Neisseria meningitidis/ or exp sepsis/ or Meningitis.mp. or Meningitides.mp. or Pachymeningitis.mp. or Pachymeningitides.mp. or Arachnoiditis.mp. or Arachnoiditides.mp. or Arachnoid Membrane inflammation.mp. or Arachnoid Membrane inflammations.mp. or Meningoencephalitides.mp. or Meningoencephalitis.mp. or Meningocephalitis.mp. or Cerebromeningitis.mp. or Encephalomeningitis.mp. or Encephalomeningitides.mp. or Haemophilus influenzae.mp. or Haemophilus parainfluenzae.mp. or Cerebromeningitides.mp. or Cerebritis.mp. or Cerebritides.mp. or Waterhouse Friderichsen Syndrome.mp. or Waterhouse Friederichsen Syndrome.mp. or Waterhouse-Friderichsen Syndrome.mp. or Waterhouse-Friederichsen Syndrome.mp. or Purpura Fulminans.mp. or Meningococcal.mp. or Meningeal.mp. or Cerebral Cryptococcosis.mp. or Cerebral Cryptococcoses.mp. or Toruloma.mp. or Torulomas.mp. or Lymphocytic Choriomeningitis.mp.<br>or Armstrong Syndrome.mp. or Armstrongs Syndrome.mp. or Encephalitis.mp. or Encephalitides.mp. or encephalitis.mp. or brain inflammation.mp. or brain inflammations.mp. or Rasmussen syndrome.mp. or Cerebral Ventriculitis.mp. or Cerebral Ventriculitides.mp. or Infectious Ventriculitis.mp. or Infectious Ventriculitides.mp. or Encephalomyelitis.mp. or Meningoencephalitides.mp. or Meningoencephalitis.mp. or Cerebromeningitis.mp. or myeloencephalitis.mp. or Lupus Vasculitis.mp. or Central Nervous System Lupus.mp. or Lupus Erythematosis.mp. or Lupus Erythematosus.mp. or brain vasculitis.mp. or brain angiitis.mp. or brain arteritis.mp. or cerebral arteritis.mp. or cerebral vasculitis.mp. or Neisseria meningitidis.mp. or Neisseria meningitides.mp. or Micrococcus intracellularis.mp. or Neisseria weichselbaumi.mp. or Meningococcus.mp. or meningococcemia.mp. or meningococci.mp. or meningococceal.mp. or meningococcaemia.mp. or sepsis.mp. or Pyemia.mp. or Pyemias.mp. or Pyohemia.mp. or Pyohemias.mp. or Pyaemia.mp. or Pyaemias.mp. or Septicemia.mp. or Septicemias.mp. or Blood Poisoning.mp. or Blood Poisonings.mp. or septic.mp. or sepses.mp. or exp congenital abnormalities/ or exp sudden infant death syndrome/ or exp fetal alcohol syndrome/ or congenital.mp. or Deformities.mp. or Deformity.mp. or Birth Defects.mp. or Birth Defect.mp. or abnormality.mp. or abnormalities.mp. or malformation.mp. or malformations.mp. or malformative.mp. or malformed.mp. or neural tube.mp. or Craniorachischisis.mp. or Craniorachischises.mp. or Diastematomyelia.mp. or Diastematomyelias.mp. or Tethered Cord.mp.<br>or Spinal Dysraphism.mp. or Tethered Spinal Cord.mp. or Spinal Dysraphisms.mp. or Iniencephaly.mp. or Iniencephalies.mp.	

	or Neurenteric.mp. or Neuroenteric.mp. or Spinal Cord Myelodysplasia.mp. or Spinal Cord Myelodysplasias.mp. or Acrania.mp. or Acranias.mp. or Exencephaly.mp. or Exencephalies.mp. or dysgraphia.mp. or dysraphic.mp. or dysraphism.mp.<br>or dysraphy.mp. or NTD.mp. or NTDS.mp. or sudden infant death.mp. or sid.mp. or SIDS.mp. or cot death.mp. or cot deaths.mp. or crib death.mp. or Fetal alcohol spectrum.mp. or Foetal alcohol spectrum.mp. or FASD.mp. or FASDs.mp. or Fetal Alcohol Syndrome.mp. or Alcohol-Related Birth Defects.mp. or Alcohol Related Birth Defects.mp. or Alcohol Related Neurodevelopmental Disorder.mp. or FAE.mp. or Fetal Alcohol Effects.mp. or FAEs.mp. or Fetal Alcohol Syndrome.mp. or Fetus Alcohol Syndrome.mp. or Foetal alcohol syndrome.mp. or exp prematurity/ or exp premature infants/ or premature.mp.<br>or preterm.mp. or pre-mature.mp. or pre-term.mp. or prematurity.mp. or prematuritas.mp. or exp asphyxia/ or asphyxia.mp.<br>or Asphyxia Neonatorum.mp. or hypoxia.mp. or Asphyxias.mp. or Suffocation.mp. or Suffocations.mp. or anoxia.mp. or (intrapartum and related).mp. or exp tetanus/ or tetanus.mp. or tetani.mp. or diphtheria-tetanus-pertussis.mp. or exp rubella/ or exp measles/ or exp necrosis/ or exp jaundice/ or exp haemorrhage/ or rubella.mp. or measles.mp. or epidemic roseola.mp. or necrosis.mp. or necrotic.mp. or necroses.mp. or necrotizing.mp. or Infarction.mp. or infractions.mp. or birth injuries.mp. or birth injury.mp. or birth trauma.mp. or Obstetric Paralyses.mp. or Obstetric Paralysis.mp. or Obstetrical Paralyses.mp. or Obstetrical Paralysis.mp. or birth damage.mp. or birth lesion.mp. or birth palsy.mp. or birth paralysis.mp. or obstetric palsy.mp. or obstetrical palsy.mp. or brachial palsy.mp. or brachial paralysis.mp. or brachial plexus paralysis.mp. or delivery trauma.mp. or arm paralysis.mp. or paralysis brachialis.mp. or jaundice.mp. or jaundices.mp. or Icterus.mp. or bronze baby syndrome.mp. or erythroleukoblastosis.mp. or hemorrhage haemorrhage.mp. or haemorrhagic.mp. or Hemorrhagic.mp. or exp nutritional disorders/ or exp starvation/ or exp deficiency/ or exp refeeding/ or exp starvation/ or exp famine/ or exp underfeeding/ or malnutrition.mp. or Nutritional.mp. or Undernutrition.mp. or Malnourishment.mp. or Malnourishments.mp. or Deficiency.mp. or Deficiencies.mp. or Refeeding Syndrome.mp. or Starvation.mp. or Starvations.mp. or Famine.mp. or Famines.mp. or deficient.mp. or underfeeding.mp. or undernourishment.mp. or nutrition.mp. or Hypervitaminosis.mp. or exp infectious diseases/ or exp infant disorders/ or exp childhood diseases/ or Noncommunicable.mp. or Non-communicable.mp. or communicable.mp. or non-infectious.mp. or infectious.mp. or noninfectious.mp. or newborn.mp. or neonatal.mp. or perinatal.mp. [mp=abstract, title, original title, broad terms, heading words, identifiers, cabicodes] (1017753)<br>4 (Argentina or Bolivia or Brazil or Brasil or Chile or Colombia or Ecuador or French Guiana or Guyana or Paraguay<br>or Peru or Suriname or Uruguay or Venezuela or Mexico or Belize or Costa Rica or El Salvador or Guatemala or Honduras or Nicaragua or Puerto Rico or Panama or West Indies or Antigua or Barbuda or Bahamas or Barbados or Cuba or Dominica or Dominican Republic or Grenada or Guadeloupe or Haiti or Jamaica or Martinique or Antilles or Anguilla or Saint Kitts or St Kitts or Saint Lucia or St Lucia or Saint Vincent or St Vincent or Trinidad or Tobago or Virgin Islands or Kazakhstan or Kyrgyzstan or Tajikistan or Turkmenistan or Uzbekistan or Borneo or Brunei or Cambodia or East Timor or Indonesia or Laos or Malaysia or Mekong Valley or Myanmar or Burma or Philippines or Singapore or Thailand or Vietnam or Bangladesh or Bhutan or India or Nepal or Pakistan or Sri Lanka or China or Korea or Macao or Macau or Mongolia or Taiwan or Afghanistan or Bahrain or Iran or Iraq or Israel or Jordan or Kuwait or Lebanon or Oman or Qatar or Saudi Arabia or Syria or Turkey or United Arab Emirates or Yemen or Fiji or New Caledonia or Papua New Guinea or Vanuatu or Micronesia or Melanesia or Guam or Palau or Polynesia or Samoa or Tonga or Armenia or Azerbaijan or Georgia or Albania or Estonia or Latvia or Lithuania or Bosnia or Herzegovina or Serbia or Bulgaria or Belarus or Croatia or Czech Republic or Hungary or Macedonia or Moldova or Montenegro or Poland or Romania or Russia or Bashkiria or Dagestan or Slovakia or Slovenia or Ukraine or Cameroon or Central African Republic or Chad or Congo or Equatorial Guinea or Gabon or Burundi or Djibouti or Eritrea or Ethiopia or Kenya or Rwanda or Somalia or Sudan or Tanzania or Uganda or Angola or Botswana or Lesotho or Malawi or Mozambique or Namibia or South Africa or Swaziland or Zambia or Zimbabwe or Benin or Burkina Faso or Cote d'Ivoire or Gambia or Ghana or Guinea or Guinea-Bissau or Liberia or Mali or Mauritania or Niger or Nigeria or Senegal or Sierra Leone or Togo or Algeria or Egypt or Libya or Morocco or Tunisia or Comoros or Madagascar or Mauritius or Reunion or Seychelles or Cabo Verde or Kiribati or Marshall Islands or Nauru or Niue or Sao Tome or Solomon Islands or South Sudan).mp. or exp Developing Countries/ or exp Africa/ or exp Caribbean/ or exp Pacific Islands/ or exp Mexico/ or exp Latin America/ or exp South America/ or exp Indian Ocean Islands/ or exp Central America/ or exp Asia/ or exp Central Europe/ or Developing.mp. or Least Developed.mp. or Less-Developed.mp. or Less Developed.mp. or Under-Developed.mp. or Under Developed.mp. or UnderDeveloped.mp. or third-world.mp. or third world.mp. or Africa.mp. or Caribbean Region.mp. or West Indies.mp. or Pacific Islands.mp. or Micronesia.mp. or Melanesia.mp. or Polynesia.mp. or Mexico.mp. or Latin America.mp. or South America.mp. or Indian Ocean Islands.mp. or Pemba.mp. or Cocos.mp. or Maldives.mp. or Central America.mp. or Asia.mp. or far east.mp. or eastern europe.mp. or central Europe.mp. or province.mp. or provinces.mp. or district.mp. or districts.mp. or prefecture.mp. or prefectures.mp. or county.mp. or counties.mp. or municipality.mp. or municipalities.mp. [mp=abstract, title, original title, broad terms, heading words, identifiers, cabicodes] (1215351)<br>5 (Animals not (man and animals)).od. (9520)<br>6 (correspondence or Editorial).pt. (44890)	
PAHO	(tw:("Infant" OR "Infant, Newborn" OR "child" OR "Child, Preschool" OR "Minors" OR "infant" OR "infants" OR "neonate" OR "neonates" OR "neonatal" OR "newborn" OR "newborns" OR "new-born" OR "new-borns" OR "baby" OR "babies" OR "Premature" OR "preterm" OR "pre term" OR "child" OR "children"	January 12, 2018

	OR "youth" OR "youths" OR "young people" OR "childhood" OR "toddler" OR "toddlers" OR "kid" OR "kids" OR "young patient" OR "young patients" OR "boy" OR "boys" OR "girl" OR "girls" OR "young age" OR "pediatric" OR "pre-schooler" OR "preschooler" OR "under 5" OR "under five" OR "under fives" OR "less than five" OR "perinatal"))<br>AND<br>(tw:("Mortality" OR "Mortality" OR "Mortalities" OR "fatal" OR "fatality" OR "fatalities" OR "Death" OR "death" OR " deaths" OR "Child Mortality"))<br>AND<br>(tw:("Diarrhea" OR "Diarrhea" OR "Diarrheas" OR "Diarrheal" OR "diarrhoeal" OR "diarrhoea" OR "diarrhoeas" OR "Dysentery" OR "Dysentery" OR "dysenteria" OR "enteritis" OR "enteritides" OR "Cholera" OR "Cholera" OR "Choleras" OR "vibrio Cholerae" OR "Gastroenteritis" OR "gastroenteritis" OR "gastroenteritides" OR "gastro enteritis" OR "gastroduodenitis" OR "gastrointestinal acute infection" OR "gastrointestinal acute infections" OR "gastrointestinal infection" OR "gastrointestinal infections" OR "digestive tract infection" OR "digestive tract infections" OR "digestive infection" OR "gastrointestinal tract infection" OR "gastrointestine tract infection" OR "Pneumonia" OR "pneumonia" OR "pneumonias" OR "pneumonitis" OR "pulmonary inflammation" OR "pulmonary inflammations" OR "lung inflammation" OR "Lung Inflammations" OR "inflammatory lung disease" OR "lobitis" OR "peripneumonia" OR "pleuropneumonia" OR "pleuropneumonitis" OR "pneumonitis" OR "pulmonal inflammation" OR "pulmonary inflammation" OR "pulmonic inflammation" OR "bronchopneumonia" OR "bronchopneumonias" OR "Respiratory Tract Infections" OR "Respiratory Tract Infections" OR "Respiratory Tract Infection" OR "Respiratory Infection" OR "Respiratory Infections" OR "whooping cough" OR "pertussis" OR "pulmonary tract infection" OR "respiration infection" OR "Bronchiolitis" OR " bronchitis" OR "bronchopulmonary infection" OR "Croup" OR "Croup" OR "diphtheria" OR "laryngitis" OR "Severe Acute Respiratory Syndrome" OR "Severe Acute Respiratory Syndrome" OR "SARS" OR "Acute Chest Syndrome" OR "acute chest syndrome" OR "acute chest syndromes" OR "Acute Lower Respiratory Infection" OR "Acute Lower Respiratory Infections" OR "ALRI" OR "LRI" OR "ARI" OR "respiration tract infection" OR "respiration tract infections" OR "legionnaire disease" OR "legionnaires' disease" OR "legionnaires disease" OR "Pontiac fever" OR "lung infiltrate" OR "lung infiltration" OR "pulmonary infiltrate" OR "pulmonary infiltration" OR "pneumonic lung" OR "rds" OR "respiratory distress" OR "respiration distress" OR "Malaria" OR "malaria" OR "malarias" OR "Plasmodium Infection" OR "Plasmodium Infections" OR "Plasmodium falciparum" OR "Plasmodium falciparum" OR "Plasmodium falciparums" OR "malaria, falciparum" OR "blackwater fever" OR "blackwater fevers" OR "black water fever" OR "black water fevers" OR "remittent fever" OR "paludism" OR "plasmodium infection" OR "plasmodium infections" OR "marsh fever" OR "falciparum infection" OR "Plasmodium vivax" OR "Plasmodium vivax" OR "Plasmodium vivaxs" OR "malaria, vivax" OR "vivax infection" OR "Plasmodium ovale" OR "Plasmodium ovale" OR "Plasmodium ovales" OR "Plasmodium malariae" OR "Plasmodium malariae" OR "Meningitis" OR "Meningitis" OR "Meningitides" OR "Pachymeningitis" OR "Pachymeningitides" OR "Arachnoiditis" OR "Arachnoiditides" OR "Arachnoid Membrane inflammation" OR "Arachnoid Membrane inflammations" OR "Meningoencephalitides" OR "Meningoencephalitis" OR "Meningocephalitis" OR "Cerebromeningitis" OR "Encephalomeningitis" OR "Encephalomeningitides" OR "Haemophilus influenzae" OR "Haemophilus parainfluenzae" OR "Cerebromeningitides" OR "Cerebritis" OR "Cerebritides" OR "Waterhouse Friderichsen Syndrome" OR "Waterhouse Friederichsen Syndrome" OR "Waterhouse-Friderichsen Syndrome" OR "Waterhouse-Friederichsen Syndrome" OR "Purpura Fulminans" OR "Meningococcal" OR "Meningeal" OR "Cerebral Cryptococcosis" OR "Cerebral Cryptococcoses" OR "Toruloma" OR "Torulomas" OR "Lymphocytic Choriomeningitis" OR "Armstrong Syndrome" OR "Armstrong's Syndrome" OR "Encephalitis" "Encephalitis" OR "Encephalitides" OR "encephalitis" OR "brain inflammation" OR "brain inflammations" OR "Rasmussen syndrome" OR "Rasmussen's syndrome" OR "Cerebral Ventriculitis" OR "Cerebral Ventriculitides" OR "Infectious Ventriculitis" OR "Infectious Ventriculitides" OR "Encephalomyelitis" OR "Meningoencephalitides" OR "Meningoencephalitis" OR "Cerebromeningitis" OR "myeloencephalitis" OR "Lupus Vasculitis" OR "Central Nervous System Lupus" OR "Lupus Erythematosis" OR "Lupus Erythematosus" OR "brain vasculitis" OR "brain angiitis" OR "brain arteritis" OR "cerebral arteritis" OR "cerebral vasculitis" OR "Neisseria meningitidis" OR "Neisseria meningitidis" OR "Neisseria meningitides" OR "Micrococcus intracellularis" OR "Neisseria weichselbaumi" OR "Meningococcus" OR "meningococcemia" OR "meningococci" OR "Meningococcal Infections" OR "meningococceal" OR "meningococcaemia" OR "sepsis" OR "sepsis" OR "Pyemia" OR "Pyemias" OR "Pyohemia" OR "Pyohemias" OR "Pyaemia" OR "Pyaemias" OR "Septicemia" OR "Septicemias" OR "Blood Poisoning" OR "Blood Poisonings" OR "septic" OR "sepses" OR "congenital abnormalities" OR "congenital" OR "Deformities" OR "Deformity" OR "Birth Defects" OR "Birth Defect" OR "abnormality" OR "abnormalities" OR "malformation" OR "malformations" OR "malformative" OR "malformed" OR "neural tube defects" OR "neural tube" OR "Craniorachischisis" OR "Craniorachischises" OR "Diastematomyelia" OR "Diastematomyelias" OR "Tethered Cord" OR "Spinal Dysraphism" OR "Tethered Spinal Cord" OR "Spinal Dysraphisms" OR "Iniencephaly" OR "Iniencephalies" OR "Neurenteric" OR "Neuroenteric" OR "Spinal Cord Myelodysplasia" OR "Spinal Cord Myelodysplasias" OR "Acrania" OR "Acranias" OR "Exencephaly" OR "Exencephalies" OR "dysgraphia" OR "dysraphic" OR "dysraphism" OR "dysraphy" OR "NTD" OR "NTDS" OR "sudden infant death syndrome" OR "sudden infant death" OR "sid" OR "SIDS" OR "cot death" OR "cot deaths" OR "crib death" OR "fetal alcohol spectrum disorders" OR "Fetal alcohol spectrum" OR "Foetal alcohol spectrum" OR "FASD" OR "FASDs" OR "Fetal Alcohol Syndrome" OR "Alcohol-Related Birth Defects" OR "Alcohol Related Birth Defects" OR "Alcohol Related Neurodevelopmental Disorder" OR "FAE" OR "Fetal Alcohol Effects" OR "FAEs" OR "Fetal Alcohol Syndrome" OR "Fetus Alcohol Syndrome" OR "Foetal alcohol syndrome" OR "premature birth"	

	OR "premature" OR "preterm" OR "pre-mature" OR "pre-term" OR "prematurity" OR "prematuritas" OR "asphyxia" OR "asphyxia" OR "Asphyxia Neonatorum" OR "Asphyxia Neonatorum" OR "hypoxia" OR "Asphyxias" OR "Suffocation" OR "Suffocations" OR "anoxia" OR ("intrapartum" AND "related") OR "tetanus" OR "tetanus" OR "tetani" OR "diphtheria-tetanus-pertussis" OR "rubella" OR "rubella" OR "measles" OR "epidemic roseola" OR "necrosis" OR "necrosis" OR "necrotic" OR "necroses" OR "necrotizing" OR "Infarction" OR "infractions" OR "birth injuries" OR "birth injuries" OR "birth injury" OR "birth trauma" OR "Obstetric Paralyses" OR "Obstetric Paralysis" OR "Obstetrical Paralyses" OR "Obstetrical Paralysis" OR "birth damage" OR "birth lesion" OR "birth palsy" OR "birth paralysis" OR "obstetric palsy" OR "obstetrical palsy" OR "brachial palsy" OR "brachial paralysis" OR "brachial plexus paralysis" OR "delivery trauma" OR "arm paralysis" OR "paralysis brachialis" OR "jaundice" OR "jaundice" OR "jaundices" OR "Icterus" OR "bronze baby syndrome" OR "erythroleukoblastosis" OR "hemorrhage" OR "hemorrhage" "haemorrhage" OR "haemorrhage" OR "haemorrhagic" OR "Hemorrhagic" OR "Nutrition disorders" OR "malnutrition" OR "Nutritional" OR "Undernutrition" OR "Malnourishment" OR " Malnourishments" OR "Deficiency" OR "Deficiencies" OR "Refeeding Syndrome" OR "Starvation" OR "Starvations" OR "Famine" OR "Famines" OR "deficient" OR "underfeeding" OR "undernourishment" OR "nutrition" OR "Hypervitaminosis" OR "Noncommunicable Diseases" OR "Noncommunicable" OR "Non-communicable" OR "communicable" OR "non-infectious" OR "infectious" OR "noninfectious" OR "Communicable Diseases" OR "Infant, Newborn, Diseases" OR "newborn" OR "neonatal" OR "perinatal"))<br>AND<br>(tw:("Argentina" OR "Bolivia" OR "Brazil" OR "Brasil" OR "Chile" OR "Colombia" OR "Ecuador" OR "French Guiana" OR "Guyana" OR "Paraguay" OR "Peru" OR "Suriname" OR "Uruguay" OR "Venezuela" OR "Mexico" OR "Belize" OR "Costa Rica" OR "El Salvador" OR "Guatemala" OR "Honduras" OR "Nicaragua" OR "Puerto Rico" OR "Panama" OR "West Indies" OR "Antigua" OR "Barbuda" OR "Bahamas" OR "Barbados" OR "Cuba" OR "Dominica" OR "Dominican Republic" OR "Grenada" OR "Guadeloupe" OR "Haiti" OR "Jamaica" OR "Martinique" OR "Antilles" OR "Anguilla" OR "Saint Kitts" OR "St Kitts" OR "Saint Lucia" OR "St Lucia" OR "Saint Vincent" OR "St Vincent" OR "Trinidad " OR "Tobago" OR "Virgin Islands" OR "Kazakhstan" OR "Kyrgyzstan" OR "Tajikistan" OR "Turkmenistan" OR "Uzbekistan" OR "Borneo" OR "Brunei" OR "Cambodia" OR "East Timor" OR "Indonesia" OR "Laos" OR "Malaysia" OR "Mekong Valley" OR "Myanmar" OR "Burma" OR "Philippines" OR "Singapore" OR "Thailand" OR "Vietnam" OR "Bangladesh" OR "Bhutan" OR "India" OR "Nepal" OR "Pakistan" OR "Sri Lanka" OR "China" OR "Korea" OR "Macao" OR "Macau" OR "Mongolia" OR "Taiwan" OR "Afghanistan" OR "Bahrain" OR "Iran" OR "Iraq" OR "Israel" OR "Jordan" OR "Kuwait" OR "Lebanon" OR "Oman" OR "Qatar" OR "Saudi Arabia" OR "Syria" OR "Turkey" OR "United Arab Emirates" OR "Yemen" OR "Fiji" OR "New Caledonia" OR "Papua New Guinea" OR "Vanuatu" OR "Micronesia" OR "Melanesia" OR "Guam" OR "Palau" OR "Polynesia" OR "Samoa" OR "Tonga" OR "Armenia" OR "Azerbaijan" OR "Georgia" OR "Albania" OR "Estonia" OR "Latvia" OR "Lithuania" OR "Bosnia" OR "Herzegovina" OR "Serbia" OR "Bulgaria" OR "Belarus" OR "Croatia" OR "Czech Republic" OR "Hungary" OR "Macedonia" OR "Moldova" OR "Montenegro" OR "Poland" OR "Romania" OR "Russia" OR "Bashkiria" OR "Dagestan" OR "Slovakia" OR "Slovenia" OR "Ukraine" OR "Cameroon" OR "Central African Republic" OR "Chad" OR "Congo" OR "Equatorial Guinea" OR "Gabon" OR "Burundi" OR "Djibouti" OR "Eritrea" OR "Ethiopia" OR "Kenya" OR "Rwanda" OR "Somalia" OR "Sudan" OR "Tanzania" OR "Uganda" OR "Angola" OR "Botswana" OR "Lesotho" OR "Malawi" OR "Mozambique" OR "Namibia" OR "South Africa" OR "Swaziland" OR "Zambia" OR "Zimbabwe" OR "Benin" OR "Burkina Faso" OR "Cote d'Ivoire" OR "Gambia" OR "Ghana" OR "Guinea" OR "Guinea-Bissau" OR "Liberia" OR " Mali" OR "Mauritania" OR "Niger" OR "Nigeria" OR "Senegal" OR "Sierra Leone" OR "Togo" OR "Algeria" OR "Egypt" OR "Libya" OR "Morocco" OR "Tunisia" OR "Comoros" OR "Madagascar" OR "Mauritius" OR "Reunion" OR "Seychelles" OR "Cabo Verde" OR "Kiribati" OR "Marshall Islands" OR "Nauru" OR "Niue" OR "Sao Tome" OR "Solomon Island" OR "South Sudan" OR "Developing Countries" OR "Developing" OR "Least Developed" OR "Less-Developed" OR "Less Developed" OR "Under-Developed" OR "Under Developed" OR "UnderDeveloped" OR "third-world" OR "third world" OR "Africa" OR "Africa" OR "Caribbean Region" OR "Caribbean Region" OR "West Indies" OR "Pacific Islands" OR "Pacific Islands" OR "Micronesia" OR "Melanesia" OR "Polynesia" OR "Mexico" OR "Mexico" OR "Latin America" OR "Latin America" OR "South America" OR "South America" OR "Indian Ocean Islands" OR "Indian Ocean Islands" OR "Pemba" OR "Cocos" OR "Maldives" OR "Central America" OR "Central America" OR "Asia" OR "Asia" OR "far east" OR "eastern europe" OR "eastern europe" OR "province" OR "provinces" OR "district" OR "districts" OR "prefecture" OR "prefectures" OR "county" OR "counties" OR "municipality" OR "municipalities"))<br>AND<br>(db:("PAHOIRIS" OR "PAHO"))<br>AND<br>(instance:"regional")	
Cochrane	ID Search<br>#1 MeSH descriptor: [Mortality] explode all trees<br>#2 MeSH descriptor: [Death] explode all trees<br>#3 "Mortality" or "Mortalities" or "fatal" or "fatality" or "fatalities" or "death" or " deaths":ti,ab,kw<br>#4 #1 or #2 or #3	January 12, 2018

	#5 MeSH descriptor: [Infant] explode all trees<br>#6 MeSH descriptor: [Child] explode all trees<br>#7 MeSH descriptor: [Minors] explode all trees<br>#8 "infant" or "infants" or "neonate" or "neonates" or "neonatal" or "newborn" or "newborns" or "new-born" or "new-borns" or "baby" or "babies" or "Premature" or "preterm" or "pre term" or "child" or "children" or "youth" or "youths" or "young people" or "childhood" or "toddler" or "toddlers" or "kid" or "kids" or "young patient" or "young patients" or "boy" or "boys" or "girl" or "girls" or "young age" or "pediatric" or "pre-schooler" or "preschooler" or "under 5" or "under five" or "under fives" or "less than five" or "perinatal":ti,ab,kw (Word variations have been searched)<br>#9 #5 or #6 or #7 or #8<br>#10 "Argentina" or "Bolivia" or "Brazil" or "Brasil" or "Chile" or "Colombia" or "Ecuador" or "French Guiana" or "Guyana" or "Paraguay" or "Peru" or "Suriname" or "Uruguay" or "Venezuela" or "Mexico" or "Belize" or "Costa Rica" or "El Salvador" or "Guatemala" or "Honduras" or "Nicaragua" or "Puerto Rico" or "Panama" or "West Indies" or "Antigua" or "Barbuda" or "Bahamas" or "Barbados" or "Cuba" or "Dominica" or "Dominican Republic" or "Grenada" or "Guadeloupe" or "Haiti" or "Jamaica" or "Martinique" or "Antilles" or "Anguilla" or "Saint Kitts" or "St Kitts" or "Saint Lucia" or "St Lucia" or "Saint Vincent" or "St Vincent" or "Trinidad " or "Tobago" or "Virgin Islands" or "Kazakhstan" or "Kyrgyzstan" or "Tajikistan" or "Turkmenistan" or "Uzbekistan" or "Borneo" or "Brunei" or "Cambodia" or "East Timor" or "Indonesia" or "Laos" or "Malaysia" or "Mekong Valley" or "Myanmar" or "Burma" or "Philippines" or "Singapore" or "Thailand" or "Vietnam" or "Bangladesh" or "Bhutan" or "India" or "Nepal" or "Pakistan" or "Sri Lanka" or "China" or "Korea" or "Macao" or "Macau" or "Mongolia" or "Taiwan" or "Afghanistan" or "Bahrain" or "Iran" or "Iraq" or "Israel" or "Jordan" or "Kuwait" or "Lebanon" or "Oman" or "Qatar" or "Saudi Arabia" or "Syria" or "Turkey" or "United Arab Emirates" or "Yemen" or "Fiji" or "New Caledonia" or "Papua New Guinea" or "Vanuatu" or "Micronesia" or "Melanesia" or "Guam" or "Palau" or "Polynesia" or "Samoa" or "Tonga" or "Armenia" or "Azerbaijan" or "Georgia" or "Albania" or "Estonia" or "Latvia" or "Lithuania" or "Bosnia" or "Herzegovina" or "Serbia" or "Bulgaria" or "Belarus" or "Croatia" or "Czech Republic" or "Hungary" or "Macedonia" or "Moldova" or "Montenegro" or "Poland" or "Romania" or "Russia" or "Bashkiria" or "Dagestan" or "Slovakia" or "Slovenia" or "Ukraine" or "Cameroon" or "Central African Republic" or "Chad" or "Congo" or "Equatorial Guinea" or "Gabon" or "Burundi" or "Djibouti" or "Eritrea" or "Ethiopia" or "Kenya" or "Rwanda" or "Somalia" or "Sudan" or "Tanzania" or "Uganda" or "Angola" or "Botswana" or "Lesotho" or "Malawi" or "Mozambique" or "Namibia" or "South Africa" or "Swaziland" or "Zambia" or "Zimbabwe" or "Benin" or "Burkina Faso" or "Cote d'Ivoire" or "Gambia" or "Ghana" or "Guinea" or "Guinea-Bissau" or "Liberia" or " Mali" or "Mauritania" or "Niger" or "Nigeria" or "Senegal" or "Sierra Leone" or "Togo" or "Algeria" or "Egypt" or "Libya" or "Morocco" or "Tunisia" or "Comoros" or "Madagascar" or "Mauritius" or "Reunion" or "Seychelles"<br>#11 MeSH descriptor: [Developing Countries] explode all trees<br>#12 MeSH descriptor: [Africa] explode all trees<br>#13 MeSH descriptor: [Caribbean Region] explode all trees<br>#14 MeSH descriptor: [Pacific Islands] explode all trees<br>#15 MeSH descriptor: [Mexico] explode all trees<br>#16 MeSH descriptor: [Latin America] explode all trees<br>#17 MeSH descriptor: [Indian Ocean Islands] explode all trees<br>#18 MeSH descriptor: [Asia] explode all trees<br>#19 MeSH descriptor: [Central America] explode all trees<br>#20 MeSH descriptor: [South America] explode all trees<br>#21 MeSH descriptor: [Europe, Eastern] explode all trees<br>#22 "Developing" or "Least Developed" or "Less-Developed" or "Less Developed" or "Under-Developed" or "Under Developed" or "UnderDeveloped" or "third-world" or "third world" or "Africa" or "Caribbean Region" or "West Indies" or "Pacific Islands" or "Micronesia" or "Melanesia" or "Polynesia" or "Mexico" or "Latin America" or "South America" or "Indian Ocean Islands" or "Pemba" or "Cocos" or "Maldives" or "Central America" or "Asia" or "far east" or "eastern europe":ti,ab,kw<br>#23 #11 or #12 or #13 or #14 or #15 or #16 or #17 or #18 or #19 or #20 or #21 or #22<br>#24 "province" or "provinces" or "district" or "districts" or "prefecture" or "prefectures" or "county" or "counties" or "municipality" or "municipalities":ti,ab,kw<br>#25 MeSH descriptor: [Animals] explode all trees<br>#26 case reports:pt (Word variations have been searched)<br>#27 editorial:pt (Word variations have been searched)<br>#28 comment:pt (Word variations have been searched)	

#29 practice guideline:pt (Word variations have been searched)
#30 #26 or #27 or #28 or #29
#31 #4 and #9 and (#10 or #23 or #24) not #25 not #30
[**Error**] ==> #32
#33 MeSH descriptor: [Diarrhea] explode all trees
#34 MeSH descriptor: [Dysentery] explode all trees
#35 MeSH descriptor: [Gastroenteritis] explode all trees
#36 MeSH descriptor: [Cholera] explode all trees
#37 "Diarrhea" or "Diarrheas" or "Diarrheal" or "diarrhoeal" or "diarrhoea" or "diarrhoeas" or "Dysentery" or "dysenteria" or "enteritis" or "enteritides" or "Cholera" or "Choleras" or "vibrio Cholerae" or "gastroenteritis" or "gastroenteritides" or "gastro enteritis" or "gastroduodenitis" or "gastrointestinal acute infection" or "gastrointestinal acute infections" or "gastrointestinal infection" or "gastrointestinal infections" or "digestive tract infection" or "digestive tract infections" or "digestive infection" or "gastrointestinal tract infection" or "gastrointestine tract infection":ti,ab,kw
#38 #33 or #34 or #35 or #36 or #37
#39 "pneumonia" or "pneumonias" or "pneumonitis" or "pulmonary inflammation" or "pulmonary inflammations" or "lung inflammation" or "Lung Inflammations" or "inflammatory lung disease" or "lobitis" or "peripneumonia" or "pleuropneumonia" or "pleuropneumonitis" or "pneumonitis" or "pulmonal inflammation" or "pulmonary inflammation" or "pulmonic inflammation" or "bronchopneumonia" or "bronchopneumonias" or "Respiratory Tract Infections" or "Respiratory Tract Infection" or "Respiratory Infection" or "Respiratory Infections" or "whooping cough" or "pertussis" or "pulmonary tract infection" or "respiration infection" or "Bronchiolitis" or " bronchitis" or "bronchopulmonary infection" or "Croup" or "diphtheria" or "laryngitis" or "Severe Acute Respiratory Syndrome" or "SARS" or "acute chest syndrome" or "acute chest syndromes" or "Acute Lower Respiratory Infection" or "Acute Lower Respiratory Infections" or "ALRI" or "LRI" or "ARI" or "respiration tract infection" or "respiration tract infections" or "legionnaire disease" or "legionnaires' disease" or "legionnaires disease" or "Pontiac fever" or "lung infiltrate" or "lung infiltration" or "pulmonary infiltrate" or "pulmonary infiltration" or "pneumonic lung" or "rds" or "respiratory distress" or "respiration distress":ti,ab,kw
#40 MeSH descriptor: [Croup] explode all trees
#41 MeSH descriptor: [Pneumonia] explode all trees
#42 MeSH descriptor: [Respiratory Tract Infections] explode all trees
#43 MeSH descriptor: [Severe Acute Respiratory Syndrome] explode all trees
#44 MeSH descriptor: [Acute Chest Syndrome] explode all trees
#45 #39 or #40 or #41 or #42 or #43 or #44
#46 "malaria" or "malarias" or "Plasmodium Infection" or "Plasmodium Infections" or "Plasmodium falciparum" or "Plasmodium falciparums" or "blackwater fever" or "blackwater fevers" or "black water fever" or "black water fevers" or "remittent fever" or "paludism" or "plasmodium infection" or "plasmodium infections" or "marsh fever" or "falciparum infection" or "Plasmodium vivax" or "Plasmodium vivaxs" or "vivax infection" or "Plasmodium ovale" or "Plasmodium ovales" or "Plasmodium malariae":ab,ti,kw
#47 MeSH descriptor: [Malaria] explode all trees
#48 MeSH descriptor: [Plasmodium vivax] explode all trees
#49 MeSH descriptor: [Malaria, Vivax] explode all trees
#50 MeSH descriptor: [Plasmodium ovale] explode all trees
#51 MeSH descriptor: [Plasmodium malariae] explode all trees
#52 MeSH descriptor: [Malaria, Falciparum] explode all trees
#53 MeSH descriptor: [Plasmodium falciparum] explode all trees
#54 #46 or #47 or #48 or #49 or #50 or #51 or #52 or #53
#55 MeSH descriptor: [Meningitis] explode all trees
#56 MeSH descriptor: [Encephalitis] explode all trees
#57 MeSH descriptor: [Meningococcal Infections] explode all trees
#58 MeSH descriptor: [Neisseria meningitidis] explode all trees
#59 MeSH descriptor: [Sepsis] explode all trees
#60 "Meningitis" or "Meningitides" or "Pachymeningitis" or "Pachymeningitides" or "Arachnoiditis" or "Arachnoiditides" or "Arachnoid Membrane inflammation" or "Arachnoid Membrane inflammations" or "Meningoencephalitides" or "Meningoencephalitis" or "Meningocephalitis" or

"Cerebromeningitis" or "Encephalomeningitis" or "Encephalomeningitides" or "Haemophilus influenzae" or "Haemophilus parainfluenzae" or "Cerebromeningitides" or "Cerebritis" or "Cerebritides" or "Waterhouse Friderichsen Syndrome" or "Waterhouse Friederichsen Syndrome" or "Waterhouse-Friderichsen Syndrome" or "Waterhouse-Friederichsen Syndrome" or "Purpura Fulminans" or "Meningococcal" or "Meningeal" or "Cerebral Cryptococcosis" or "Cerebral Cryptococcoses" or "Toruloma" or "Torulomas" or "Lymphocytic Choriomeningitis" or "Armstrong Syndrome" or "Armstrong's Syndrome" or "Encephalitis" or "Encephalitides" or "encephalitis" or "brain inflammation" or "brain inflammations" or "Rasmussen syndrome" or "Rasmussen's syndrome" or "Cerebral Ventriculitis" or "Cerebral Ventriculitides" or "Infectious Ventriculitis" or "Infectious Ventriculitides" or "Encephalomyelitis" or "Meningoencephalitides" or "Meningoencephalitis" or "Cerebromeningitis" or "myeloencephalitis" or "Lupus Vasculitis" or "Central Nervous System Lupus" or "Lupus Erythematosis" or "Lupus Erythematosus" or "brain vasculitis" or "brain angiitis" or "brain arteritis" or "cerebral arteritis" or "cerebral vasculitis" or "Neisseria meningitidis" or "Neisseria meningitides" or "Micrococcus intracellularis" or "Neisseria weichselbaumi" or "Meningococcus" or "meningococcemia" or "meningococci" or "meningococceal" or "meningococcaemia" or "sepsis" or "Pyemia" or "Pyemias" or "Pyohemia" or "Pyohemias" or "Pyaemia" or "Pyaemias" or "Septicemia" or "Septicemias" or "Blood Poisoning" or "Blood Poisonings" or "septic" or "sepses":ti,ab,kw

#61 #55 or #56 or #57 or #58 or #59 or #60
#62 MeSH descriptor: [Congenital Abnormalities] explode all trees
#63 MeSH descriptor: [Sudden Infant Death] explode all trees
#64 MeSH descriptor: [Neural Tube Defects] explode all trees
#65 MeSH descriptor: [Fetal Alcohol Spectrum Disorders] explode all trees
#66 "congenital" or "Deformities" or "Deformity" or "Birth Defects" or "Birth Defect" or "abnormality" or "abnormalities" or "malformation" or "malformations" or "malformative" or "malformed" or "neural tube" or "Craniorachischisis" or "Craniorachischises" or "Diastematomyelia" or "Diastematomyelias" or "Tethered Cord" or "Spinal Dysraphism" or "Tethered Spinal Cord" or "Spinal Dysraphisms" or "Iniencephaly" or "Iniencephalies" or "Neurenteric" or "Neuroenteric" or "Spinal Cord Myelodysplasia" or "Spinal Cord Myelodysplasias" or "Acrania" or "Acranias" or "Exencephaly" or "Exencephalies" or "dysgraphia" or "dysraphic" or "dysraphism" or "dysraphy" or "NTD" or "NTDS" or "sudden infant death" or "sid" or "SIDS" or "cot death" or "cot deaths" or "crib death" or "Fetal alcohol spectrum" or "Foetal alcohol spectrum" or "FASD" or "FASDs" or "Fetal Alcohol Syndrome" or "Alcohol-Related Birth Defects" or "Alcohol Related Birth Defects" or "Alcohol Related Neurodevelopmental Disorder" or "FAE" or "Fetal Alcohol Effects" or "FAEs" or "Fetal Alcohol Syndrome" or "Fetus Alcohol Syndrome" or "Foetal alcohol syndrome":ti,ab,kw
#67 #62 or #63 or #64 or #65 or #66
#68 MeSH descriptor: [Premature Birth] explode all trees
#69 "premature" or "preterm" or "pre-mature" or "pre-term" or "prematurity" or "prematuritas":ti,ab,kw
#70 #68 or #69
#71 MeSH descriptor: [Asphyxia] explode all trees
#72 MeSH descriptor: [Asphyxia Neonatorum] explode all trees
#73 "asphyxia" or "Asphyxia Neonatorum" or "hypoxia" or "Asphyxias" or "Suffocation" or "Suffocations" or "anoxia" or ("intrapartum" and "related"):ti,ab,kw
#74 #71 or #72 or #73
#75 MeSH descriptor: [Tetanus] explode all trees
#76 "tetanus" or "tetani" or "diphtheria-tetanus-pertussis":ti,ab,kw
#77 #75 or #76
#78 MeSH descriptor: [Rubella] explode all trees
#79 MeSH descriptor: [Necrosis] explode all trees
#80 MeSH descriptor: [Birth Injuries] explode all trees
#81 MeSH descriptor: [Jaundice] explode all trees
#82 MeSH descriptor: [Hemorrhage] explode all trees
#83 "rubella" or "measles" or "epidemic roseola" or "necrosis" or "necrotic" or "necroses" or "necrotizing" or "Infarction" or "infractions" or "birth injuries" or "birth injury" or "birth trauma" or "Obstetric Paralyses" or "Obstetric Paralysis" or "Obstetrical Paralyses" or "Obstetrical Paralysis" or "birth damage" or "birth lesion" or "birth palsy" or "birth paralysis" or "obstetric palsy" or "obstetrical palsy" or "brachial palsy" or "brachial paralysis" or "brachial plexus paralysis" or "delivery trauma" or "arm paralysis" or "paralysis brachialis" or "jaundice" or "jaundices" or "Icterus" or "bronze baby syndrome" or "erythroleukoblastosis" or "hemorrhage" "haemorrhage" or "haemorrhagic" or "Hemorrhagic":ti,ab,kw
#84 #78 or #79 or #80 or #81 or #82 or #83

	#85 MeSH descriptor: [Nutrition Disorders] explode all trees<br>#86 "malnutrition" or "Nutritional" or "Undernutrition" or "Malnourishment" or " Malnourishments" or "Deficiency" or "Deficiencies" or "Refeeding Syndrome" or "Starvation" or "Starvations" or "Famine" or "Famines" or "deficient" or "underfeeding" or "undernourishment" or "nutrition" or "Hypervitaminosis":ti,ab,kw<br>#87 #85 or #86<br>#88 MeSH descriptor: [Noncommunicable Diseases] explode all trees<br>#89 MeSH descriptor: [Communicable Diseases] explode all trees<br>#90 MeSH descriptor: [Infant, Newborn, Diseases] explode all trees<br>#91 "Noncommunicable" or "Non-communicable" or "communicable" or "non-infectious" or "infectious" or "noninfectious" or "newborn" or "neonatal" or "perinatal":ti,ab,kw<br>#92 #88 or #89 or #90 or #91<br>#93 (#38 or #44 or #54 or #61 or #67 or #70 or #74 or #77 or #84 or #87 or #92)<br>#94 #31 and #93 Publication Year from 2015 to 2017	
Scopus	((TITLE-ABS-KEY ("Infant" OR "Infant, Newborn" OR "child" OR "Child, Preschool" OR "Minors" OR "infant" OR "infants" OR "neonate" OR "neonates" OR "neonatal" OR "newborn" OR "newborns" OR "new-born" OR "new-borns" OR "baby" OR "babies" OR "Premature" OR "preterm" OR "pre term" OR "child" OR "children" OR "youth" OR "youths" OR "young people" OR "childhood" OR "toddler" OR "toddlers" OR "kid" OR "kids" OR "young patient" OR "young patients" OR "boy" OR "boys" OR "girl" OR "girls" OR "young age" OR "pediatric" OR "pre-schooler" OR "preschooler" OR "under 5" OR "under five" OR "under fives" OR "less than five" OR "perinatal"))<br>AND<br>((TITLE-ABS-KEY ("Mortality" OR "Mortality" OR "Mortalities" OR "fatal" OR "fatality" OR "fatalities" OR "Death" OR "death" OR " deaths" OR "Child Mortality"))<br>AND<br>((TITLE-ABS-KEY ("Diarrhea" OR "Diarrhea" OR "Diarrheas" OR "Diarrheal" OR "diarrhoeal" OR "diarrhoea" OR "diarrhoeas" OR "Dysentery" OR "Dysentery" OR "dysenteria" OR "enteritis" OR "enteritides" OR "Cholera" OR "Cholera" OR "Choleras" OR "vibrio Cholerae" OR "Gastroenteritis" OR "gastroenteritis" OR "gastroenteritides" OR "gastro enteritis" OR "gastroduodenitis" OR "gastrointestinal acute infection" OR "gastrointestinal acute infections" OR "gastrointestinal infection" OR "gastrointestinal infections" OR "digestive tract infection" OR "digestive tract infections" OR "digestive infection" OR "gastrointestinal tract infection" OR "gastrointestine tract infection") OR TITLE-ABS-KEY ("Pneumonia" OR "pneumonia" OR "pneumonias" OR "pneumonitis" OR "pulmonary inflammation" OR "pulmonary inflammations" OR "lung inflammation" OR "Lung Inflammations" OR "inflammatory lung disease" OR "lobitis" OR "peripneumonia" OR "pleuropneumonia" OR "pleuropneumonitis" OR "pneumonitis" OR "pulmonal inflammation" OR "pulmonary inflammation" OR "pulmonic inflammation" OR "bronchopneumonia" OR "bronchopneumonias" OR "Respiratory Tract Infections" OR "Respiratory Tract Infections" OR "Respiratory Tract Infection" OR "Respiratory Infection" OR "Respiratory Infections" OR "whooping cough" OR "pertussis" OR "pulmonary tract infection" OR "respiration infection" OR "Bronchiolitis" OR " bronchitis" OR "bronchopulmonary infection" OR "Croup" OR "Croup" OR "diphtheria" OR "laryngitis" OR "Severe Acute Respiratory Syndrome" OR "Severe Acute Respiratory Syndrome" OR "SARS" OR "Acute Chest Syndrome" OR "acute chest syndrome" OR "acute chest syndromes" OR "Acute Lower Respiratory Infection" OR "Acute Lower Respiratory Infections" OR "ALRI" OR "LRI" OR "ARI" OR "respiration tract infection" OR "respiration tract infections" OR "legionnaire disease" OR "legionnaires' disease" OR "legionnaires disease" OR "Pontiac fever" OR "lung infiltrate" OR "lung infiltration" OR "pulmonary infiltrate" OR "pulmonary infiltration" OR "pneumonic lung" OR "rds" OR "respiratory distress" OR "respiration distress") OR TITLE-ABS-KEY ("Malaria" OR "malaria" OR "malarias" OR "Plasmodium Infection" OR "Plasmodium Infections" OR "Plasmodium falciparum" OR "Plasmodium falciparum" OR "Plasmodium falciparums" OR "malaria, falciparum" OR "blackwater fever" OR "blackwater fevers" OR "black water fever" OR "black water fevers" OR "remittent fever" OR "paludism" OR "plasmodium infection" OR "plasmodium infections" OR "marsh fever" OR "falciparum infection" OR "Plasmodium vivax" OR "Plasmodium vivax" OR "Plasmodium vivaxs" OR "malaria, vivax" OR "vivax infection" OR "Plasmodium ovale" OR "Plasmodium ovale" OR "Plasmodium ovales" OR "Plasmodium malariae" OR "Plasmodium malariae") OR TITLE-ABS-KEY ("Meningitis" OR "Meningitis" OR "Meningitides" OR "Pachymeningitis" OR "Pachymeningitides" OR "Arachnoiditis" OR "Arachnoiditides" OR "Arachnoid Membrane inflammation" OR "Arachnoid Membrane inflammations" OR "Meningoencephalitides" OR "Meningoencephalitis" OR "Meningocephalitis" OR "Cerebromeningitis" OR "Encephalomeningitis" OR "Encephalomeningitides" OR "Haemophilus influenzae" OR "Haemophilus parainfluenzae" OR "Cerebromeningitides" OR "Cerebritis" OR "Cerebritides" OR "Waterhouse Friderichsen Syndrome" OR "Waterhouse Friederichsen Syndrome" OR "Waterhouse-Friderichsen Syndrome" OR "Waterhouse-Friederichsen Syndrome" OR "Purpura Fulminans" OR "Meningococcal" OR "Meningeal" OR "Cerebral Cryptococcosis" OR	January 14, 2018

	"Cerebral Cryptococcoses" OR "Toruloma" OR "Torulomas" OR "Lymphocytic Choriomeningitis" OR "Armstrong Syndrome" OR "Armstrong's Syndrome" OR "Encephalitis" "Encephalitis" OR "Encephalitides" OR "encephalitis" OR "brain inflammation" OR "brain inflammations" OR "Rasmussen syndrome" OR "Rasmussen's syndrome" OR "Cerebral Ventriculitis" OR "Cerebral Ventriculitides" OR "Infectious Ventriculitis" OR "Infectious Ventriculitides" OR "Encephalomyelitis" OR "Meningoencephalitides" OR "Meningoencephalitis" OR "Cerebromeningitis" OR "myeloencephalitis" OR "Lupus Vasculitis" OR "Central Nervous System Lupus" OR "Lupus Erythematosis" OR "Lupus Erythematosus" OR "brain vasculitis" OR "brain angiitis" OR "brain arteritis" OR "cerebral arteritis" OR "cerebral vasculitis" OR "Neisseria meningitidis" OR "Neisseria meningitidis" OR "Neisseria meningitides" OR "Micrococcus intracellularis" OR "Neisseria weichselbaumi" OR "Meningococcus" OR "meningococcemia" OR "meningococci" OR "Meningococcal Infections" OR "meningococceal" OR "meningococcaemia" OR "sepsis" OR "sepsis" OR "Pyemia" OR "Pyemias" OR "Pyohemia" OR "Pyohemias" OR "Pyaemia" OR "Pyaemias" OR "Septicemia" OR "Septicemias" OR "Blood Poisoning" OR "Blood Poisonings" OR "septic" OR "sepses") OR TITLE-ABS-KEY ("congenital abnormalities" OR "congenital" OR "Deformities" OR "Deformity" OR "Birth Defects" OR "Birth Defect" OR "abnormality" OR "abnormalities" OR "malformation" OR "malformations" OR "malformative" OR "malformed" OR "neural tube defects" OR "neural tube" OR "Craniorachischisis" OR "Craniorachischises" OR "Diastematomyelia" OR "Diastematomyelias" OR "Tethered Cord" OR "Spinal Dysraphism" OR "Tethered Spinal Cord" OR "Spinal Dysraphisms" OR "Iniencephaly" OR "Iniencephalies" OR "Neurenteric" OR "Neuroenteric" OR "Spinal Cord Myelodysplasia" OR "Spinal Cord Myelodysplasias" OR "Acrania" OR "Acranias" OR "Exencephaly" OR "Exencephalies" OR "dysgraphia" OR "dysraphic" OR "dysraphism" OR "dysraphy" OR "NTD" OR "NTDS" OR "sudden infant death syndrome" OR "sudden infant death" OR "sid" OR "SIDS" OR "cot death" OR "cot deaths" OR "crib death" OR "fetal alcohol spectrum disorders" OR "Fetal alcohol spectrum" OR "Foetal alcohol spectrum" OR "FASD" OR "FASDs" OR "Fetal Alcohol Syndrome" OR "Alcohol-Related Birth Defects" OR "Alcohol Related Birth Defects" OR "Alcohol Related Neurodevelopmental Disorder" OR "FAE" OR "Fetal Alcohol Effects" OR "FAEs" OR "Fetal Alcohol Syndrome" OR "Fetus Alcohol Syndrome" OR "Foetal alcohol syndrome") OR TITLE-ABS-KEY ("premature birth" OR "premature" OR "preterm" OR "pre-mature" OR "pre-term" OR "prematurity" OR "prematuritas") OR TITLE-ABS-KEY ("asphyxia" OR "asphyxia" OR "Asphyxia Neonatorum" OR "Asphyxia Neonatorum" OR "hypoxia" OR "Asphyxias" OR "Suffocation" OR "Suffocations" OR "anoxia" OR ("intrapartum" AND "related")) OR TITLE-ABS-KEY ("tetanus" OR "tetanus" OR "tetani" OR "diphtheria-tetanus-pertussis") OR TITLE-ABS-KEY ("rubella" OR "rubella" OR "measles" OR "epidemic roseola" OR "necrosis" OR "necrosis" OR "necrotic" OR "necroses" OR "necrotizing" OR "Infarction" OR "infractions" OR "birth injuries" OR "birth injuries" OR "birth injury" OR "birth trauma" OR "Obstetric Paralyses" OR "Obstetric Paralysis" OR "Obstetrical Paralyses" OR "Obstetrical Paralysis" OR "birth damage" OR "birth lesion" OR "birth palsy" OR "birth paralysis" OR "obstetric palsy" OR "obstetrical palsy" OR "brachial palsy" OR "brachial paralysis" OR "brachial plexus paralysis" OR "delivery trauma" OR "arm paralysis" OR "paralysis brachialis" OR "jaundice" OR "jaundice" OR "jaundices" OR "Icterus" OR "bronze baby syndrome" OR "erythroleukoblastosis" OR "hemorrhage" OR "hemorrhage" "haemorrhage" OR "haemorrhagic" OR "Hemorrhagic") OR TITLE-ABS-KEY ("Nutrition disorders" OR "malnutrition" OR "Nutritional" OR "Undernutrition" OR "Malnourishment" OR " Malnourishments" OR "Deficiency" OR "Deficiencies" OR "Refeeding Syndrome" OR "Starvation" OR "Starvations" OR "Famine" OR "Famines" OR "deficient" OR "underfeeding" OR "undernourishment" OR "nutrition" OR "Hypervitaminosis") OR TITLE-ABS-KEY ("Noncommunicable Diseases" OR "Noncommunicable" OR "Non-communicable" OR "communicable" OR "non-infectious" OR "infectious" OR "noninfectious" OR "Communicable Diseases" OR "Infant, Newborn, Diseases" OR "newborn" OR "neonatal" OR "perinatal"))<br>AND<br>((TITLE-ABS-KEY ("Argentina" OR "Bolivia" OR "Brazil" OR "Brasil" OR "Chile" OR "Colombia" OR "Ecuador" OR "French Guiana" OR "Guyana" OR "Paraguay" OR "Peru" OR "Suriname" OR "Uruguay" OR "Venezuela" OR "Mexico" OR "Belize" OR "Costa Rica" OR "El Salvador" OR "Guatemala" OR "Honduras" OR "Nicaragua" OR "Puerto Rico" OR "Panama" OR "West Indies" OR "Antigua" OR "Barbuda" OR "Bahamas" OR "Barbados" OR "Cuba" OR "Dominica" OR "Dominican Republic" OR "Grenada" OR "Guadeloupe" OR "Haiti" OR "Jamaica" OR "Martinique" OR "Antilles" OR "Anguilla" OR "Saint Kitts" OR "St Kitts" OR "Saint Lucia" OR "St Lucia" OR "Saint Vincent" OR "St Vincent" OR "Trinidad " OR "Tobago" OR "Virgin Islands" OR "Kazakhstan" OR "Kyrgyzstan" OR "Tajikistan" OR "Turkmenistan" OR "Uzbekistan" OR "Borneo" OR "Brunei" OR "Cambodia" OR "East Timor" OR "Indonesia" OR "Laos" OR "Malaysia" OR "Mekong Valley" OR "Myanmar" OR "Burma" OR "Philippines" OR "Singapore" OR "Thailand" OR "Vietnam" OR "Bangladesh" OR "Bhutan" OR "India" OR "Nepal" OR "Pakistan" OR "Sri Lanka" OR "China" OR "Korea" OR "Macao" OR "Macau" OR "Mongolia" OR "Taiwan" OR "Afghanistan" OR "Bahrain" OR "Iran" OR "Iraq" OR "Israel" OR "Jordan" OR "Kuwait" OR "Lebanon" OR "Oman" OR "Qatar" OR "Saudi Arabia" OR "Syria" OR "Turkey" OR "United Arab Emirates" OR "Yemen" OR "Fiji" OR "New Caledonia" OR "Papua New Guinea" OR "Vanuatu" OR "Micronesia" OR "Melanesia" OR "Guam" OR "Palau" OR "Polynesia" OR "Samoa" OR "Tonga" OR "Armenia" OR "Azerbaijan" OR "Georgia" OR "Albania" OR "Estonia" OR "Latvia" OR "Lithuania" OR "Bosnia" OR "Herzegovina" OR "Serbia" OR "Bulgaria" OR "Belarus" OR "Croatia" OR "Czech Republic" OR "Hungary" OR "Macedonia" OR "Moldova" OR "Montenegro" OR "Poland" OR "Romania" OR "Russia" OR "Bashkiria" OR "Dagestan" OR "Slovakia" OR "Slovenia" OR "Ukraine" OR "Cameroon" OR "Central African Republic" OR "Chad" OR	

	"Congo" OR "Equatorial Guinea" OR "Gabon" OR "Burundi" OR "Djibouti" OR "Eritrea" OR "Ethiopia" OR "Kenya" OR "Rwanda" OR "Somalia" OR "Sudan" OR "Tanzania" OR "Uganda" OR "Angola" OR "Botswana" OR "Lesotho" OR "Malawi" OR "Mozambique" OR "Namibia" OR "South Africa" OR "Swaziland" OR "Zambia" OR "Zimbabwe" OR "Benin" OR "Burkina Faso" OR "Cote d'Ivoire" OR "Gambia" OR "Ghana" OR "Guinea" OR "Guinea-Bissau" OR "Liberia" OR " Mali" OR "Mauritania" OR "Niger" OR "Nigeria" OR "Senegal" OR "Sierra Leone" OR "Togo" OR "Algeria" OR "Egypt" OR "Libya" OR "Morocco" OR "Tunisia" OR "Comoros" OR "Madagascar" OR "Mauritius" OR "Reunion" OR "Seychelles" OR "Cabo Verde" OR "Kiribati" OR "Marshall Islands" OR "Nauru" OR "Niue" OR "Sao Tome" OR "Solomon Islands" OR "South Sudan" OR "Developing Countries" OR "Developing" OR "Least Developed" OR "Less-Developed" OR "Less Developed" OR "Under-Developed" OR "Under Developed" OR "UnderDeveloped" OR "third-world" OR "third world" OR "Africa" OR "Africa" OR "Caribbean Region" OR "Caribbean Region" OR "West Indies" OR "Pacific Islands" OR "Pacific Islands" OR "Micronesia" OR "Melanesia" OR "Polynesia" OR "Mexico" OR "Mexico" OR "Latin America" OR "Latin America" OR "South America" OR "South America" OR "Indian Ocean Islands" OR "Indian Ocean Islands" OR "Pemba" OR "Cocos" OR "Maldives" OR "Central America" OR "Central America" OR "Asia" OR "Asia" OR "far east" OR "eastern europe" OR "eastern europe" OR "province" OR "provinces" OR "district" OR "districts" OR "prefecture" OR "prefectures" OR "county" OR "counties" OR "municipality" OR "municipalities")))))<br>AND NOT (DOCTYPE (ed) OR DOCTYPE (le) OR DOCTYPE (no) OR DOCTYPE (re))	

Popline	(((("Infant" OR "Infant, Newborn" OR "child" OR "Child, Preschool" OR "Minors" OR "infant" OR "infants" OR "neonate" OR "neonates" OR "neonatal" OR "newborn" OR "newborns" OR "new\-born" OR "new\-borns" OR "baby" OR "babies" OR "Premature" OR "preterm" OR "pre term" OR "child" OR "children" OR "youth" OR "youths" OR "young people" OR "childhood" OR "toddler" OR "toddlers" OR "kid" OR "kids" OR "young patient" OR "young patients" OR "boy" OR "boys" OR "girl" OR "girls" OR "young age" OR "pediatric" OR "pre\-schooler" OR "preschooler" OR "under 5" OR "under five" OR "under fives" OR "less than five" OR "perinatal")))<br>AND<br>((("Mortality" OR "Mortalities" OR "fatal" OR "fatality" OR "fatalities" OR "death" OR "deaths")))<br>AND<br>((("Diarrhea" OR "Diarrhea" OR "Diarrheas" OR "Diarrheal" OR "diarrhoeal" OR "diarrhoea" OR "diarrhoeas" OR "Dysentery" OR "Dysentery" OR "dysenteria" OR "enteritis" OR "enteritides" OR "Cholera" OR "Cholera" OR "Choleras" OR "vibrio Cholerae" OR "Gastroenteritis" OR "gastroenteritis" OR "gastroenteritides" OR "gastro enteritis" OR "gastroduodenitis" OR "gastrointestinal acute infection" OR "gastrointestinal acute infections" OR "gastrointestinal infection" OR "gastrointestinal infections" OR "digestive tract infection" OR "digestive tract infections" OR "digestive infection" OR "gastrointestinal tract infection" OR "gastrointestine tract infection" OR "Pneumonia" OR "pneumonia" OR "pneumonias" OR "pneumonitis" OR "pulmonary inflammation" OR "pulmonary inflammations" OR "lung inflammation" OR "Lung Inflammations" OR "inflammatory lung disease" OR "lobitis" OR "peripneumonia" OR "pleuropneumonia" OR "pleuropneumonitis" OR "pneumonitis" OR "pulmonal inflammation" OR "pulmonary inflammation" OR "pulmonic inflammation" OR "bronchopneumonia" OR "bronchopneumonias" OR "Respiratory Tract Infections" OR "Respiratory Tract Infections" OR "Respiratory Tract Infection" OR "Respiratory Infection" OR "Respiratory Infections" OR "whooping cough" OR "pertussis" OR "pulmonary tract infection" OR "respiration infection" OR "Bronchiolitis" OR " bronchitis" OR "bronchopulmonary infection" OR "Croup" OR "Croup" OR "diphtheria" OR "laryngitis" OR "Severe Acute Respiratory Syndrome" OR "Severe Acute Respiratory Syndrome" OR "SARS" OR "Acute Chest Syndrome" OR "acute chest syndrome" OR "acute chest syndromes" OR "Acute Lower Respiratory Infection" OR "Acute Lower Respiratory Infections" OR "ALRI" OR "LRI" OR "ARI" OR "respiration tract infection" OR "respiration tract infections" OR "legionnaire disease" OR "legionnaires' disease" OR "legionnaires disease" OR "Pontiac fever" OR "lung infiltrate" OR "lung infiltration" OR "pulmonary infiltrate" OR "pulmonary infiltration" OR "pneumonic lung" OR "rds" OR "respiratory distress" OR "respiration distress" OR "Malaria" OR "malaria" OR "malarias" OR "Plasmodium Infection" OR "Plasmodium Infections" OR "Plasmodium falciparum" OR "Plasmodium falciparum" OR "Plasmodium falciparums" OR "malaria, falciparum" OR "blackwater fever" OR "blackwater fevers" OR "black water fever" OR "black water fevers" OR "remittent fever" OR "paludism" OR "plasmodium infection" OR "plasmodium infections" OR "marsh fever" OR "falciparum infection" OR "Plasmodium vivax" OR "Plasmodium vivax" OR "Plasmodium vivaxs" OR "malaria, vivax" OR "vivax infection" OR "Plasmodium ovale" OR "Plasmodium ovale" OR "Plasmodium ovales" OR "Plasmodium malariae" OR "Plasmodium malariae" OR "Meningitis" OR "Meningitis" OR "Meningitides" OR "Pachymeningitis" OR "Pachymeningitides" OR "Arachnoiditis" OR "Arachnoiditides" OR "Arachnoid Membrane inflammation" OR "Arachnoid Membrane inflammations" OR "Meningoencephalitides" OR "Meningoencephalitis" OR "Meningocephalitis" OR "Cerebromeningitis" OR "Encephalomeningitis" OR "Encephalomeningitides" OR "Haemophilus influenzae" OR "Haemophilus parainfluenzae" OR "Cerebromeningitides" OR "Cerebritis" OR "Cerebritides" OR "Waterhouse Friderichsen Syndrome" OR "Waterhouse Friederichsen Syndrome" OR "Waterhouse\-Friderichsen Syndrome" OR "Waterhouse\-Friederichsen Syndrome" OR "Purpura Fulminans" OR "Meningococcal" OR "Meningeal" OR "Cerebral Cryptococcosis" OR "Cerebral Cryptococcoses" OR "Toruloma" OR "Torulomas" OR "Lymphocytic Choriomeningitis" OR "Armstrong Syndrome" OR "Armstrong's Syndrome" OR "Encephalitis" "Encephalitis" OR "Encephalitides" OR "encephalitis" OR "brain inflammation" OR "brain inflammations" OR "Rasmussen syndrome" OR "Rasmussen's syndrome" OR "Cerebral Ventriculitis" OR "Cerebral Ventriculitides" OR "Infectious Ventriculitis" OR "Infectious Ventriculitides" OR "Encephalomyelitis" OR "Meningoencephalitides" OR "Meningoencephalitis" OR "Cerebromeningitis" OR "myeloencephalitis" OR "Lupus Vasculitis" OR "Central Nervous System Lupus" OR "Lupus Erythematosis" OR "Lupus Erythematosus" OR "brain vasculitis" OR "brain angiitis" OR "brain arteritis" OR "cerebral arteritis" OR "cerebral vasculitis" OR "Neisseria meningitidis" OR "Neisseria meningitidis" OR "Neisseria meningitides" OR "Micrococcus intracellularis" OR "Neisseria weichselbaumi" OR "Meningococcus" OR "meningococcemia" OR "meningococci" OR "Meningococcal Infections" OR "meningococceal" OR "meningococcaemia" OR "sepsis" OR "sepsis" OR "Pyemia" OR "Pyemias" OR "Pyohemia" OR "Pyohemias" OR "Pyaemia" OR "Pyaemias" OR "Septicemia" OR "Septicemias" OR "Blood Poisoning" OR "Blood Poisonings" OR "septic" OR "sepses" OR "congenital abnormalities" OR "congenital" OR "Deformities" OR "Deformity" OR "Birth Defects" OR "Birth Defect" OR "abnormality" OR "abnormalities" OR "malformation" OR "malformations" OR "malformative" OR "malformed" OR "neural tube defects" OR "neural tube" OR "Craniorachischisis" OR "Craniorachischises" OR "Diastematomyelia" OR "Diastematomyelias" OR "Tethered Cord" OR "Spinal Dysraphism" OR "Tethered Spinal Cord" OR "Spinal Dysraphisms" OR "Iniencephaly" OR "Iniencephalies" OR "Neurenteric" OR "Neuroenteric" OR "Spinal Cord Myelodysplasia" OR "Spinal Cord Myelodysplasias" OR "Acrania" OR "Acranias" OR "Exencephaly" OR "Exencephalies" OR "dysgraphia" OR "dysraphic" OR "dysraphism" OR "dysraphy" OR "NTD" OR "NTDS" OR "sudden infant death syndrome" OR "sudden infant death" OR "sid" OR "SIDS" OR "cot death" OR "cot deaths" OR "crib death" OR "fetal alcohol spectrum disorders" OR "Fetal alcohol spectrum" OR "Foetal alcohol spectrum" OR "FASD" OR "FASDs" OR	January 16, 2018

	"Fetal Alcohol Syndrome" OR "Alcohol\-Related Birth Defects" OR "Alcohol Related Birth Defects" OR "Alcohol Related Neurodevelopmental Disorder" OR "FAE" OR "Fetal Alcohol Effects" OR "FAEs" OR "Fetal Alcohol Syndrome" OR "Fetus Alcohol Syndrome" OR "Foetal alcohol syndrome" OR "premature birth" OR "premature" OR "preterm" OR "pre\-mature" OR "pre\-term" OR "prematurity" OR "prematuritas" OR "asphyxia" OR "asphyxia" OR "Asphyxia Neonatorum" OR "Asphyxia Neonatorum" OR "hypoxia" OR "Asphyxias" OR "Suffocation" OR "Suffocations" OR "anoxia" OR ("intrapartum" AND "related") OR "tetanus" OR "tetanus" OR "tetani" OR "diphtheria\-tetanus\-pertussis" OR "rubella" OR "rubella" OR "measles" OR "epidemic roseola" OR "necrosis" OR "necrosis" OR "necrotic" OR "necroses" OR "necrotizing" OR "Infarction" OR "infractions" OR "birth injuries" OR "birth injuries" OR "birth injury" OR "birth trauma" OR "Obstetric Paralyses" OR "Obstetric Paralysis" OR "Obstetrical Paralyses" OR "Obstetrical Paralysis" OR "birth damage" OR "birth lesion" OR "birth palsy" OR "birth paralysis" OR "obstetric palsy" OR "obstetrical palsy" OR "brachial palsy" OR "brachial paralysis" OR "brachial plexus paralysis" OR "delivery trauma" OR "arm paralysis" OR "paralysis brachialis" OR "jaundice" OR "jaundice" OR "jaundices" OR "Icterus" OR "bronze baby syndrome" OR "erythroleukoblastosis" OR "hemorrhage" OR "hemorrhage" "haemorrhage" OR "haemorrhagic" OR "Hemorrhagic" OR "Nutrition disorders" OR "malnutrition" OR "Nutritional" OR "Undernutrition" OR "Malnourishment" OR " Malnourishments" OR "Deficiency" OR "Deficiencies" OR "Refeeding Syndrome" OR "Starvation" OR "Starvations" OR "Famine" OR "Famines" OR "deficient" OR "underfeeding" OR "undernourishment" OR "nutrition" OR "Hypervitaminosis" OR "Noncommunicable Diseases" OR "Noncommunicable" OR "Non\-communicable" OR "communicable" OR "non\-infectious" OR "infectious" OR "noninfectious" OR "Communicable Diseases" OR "Infant, Newborn, Diseases" OR "newborn" OR "neonatal" OR "perinatal")))<br>AND<br>((("Argentina" OR "Bolivia" OR "Brazil" OR "Brasil" OR "Chile" OR "Colombia" OR "Ecuador" OR "French Guiana" OR "Guyana" OR "Paraguay" OR "Peru" OR "Suriname" OR "Uruguay" OR "Venezuela" OR "Mexico" OR "Belize" OR "Costa Rica" OR "El Salvador" OR "Guatemala" OR "Honduras" OR "Nicaragua" OR "Puerto Rico" OR "Panama" OR "West Indies" OR "Antigua" OR "Barbuda" OR "Bahamas" OR "Barbados" OR "Cuba" OR "Dominica" OR "Dominican Republic" OR "Grenada" OR "Guadeloupe" OR "Haiti" OR "Jamaica" OR "Martinique" OR "Antilles" OR "Anguilla" OR "Saint Kitts" OR "St Kitts" OR "Saint Lucia" OR "St Lucia" OR "Saint Vincent" OR "St Vincent" OR "Trinidad " OR "Tobago" OR "Virgin Islands" OR "Kazakhstan" OR "Kyrgyzstan" OR "Tajikistan" OR "Turkmenistan" OR "Uzbekistan" OR "Borneo" OR "Brunei" OR "Cambodia" OR "East Timor" OR "Indonesia" OR "Laos" OR "Malaysia" OR "Mekong Valley" OR "Myanmar" OR "Burma" OR "Philippines" OR "Singapore" OR "Thailand" OR "Vietnam" OR "Bangladesh" OR "Bhutan" OR "India" OR "Nepal" OR "Pakistan" OR "Sri Lanka" OR "China" OR "Korea" OR "Macao" OR "Macau" OR "Mongolia" OR "Taiwan" OR "Afghanistan" OR "Bahrain" OR "Iran" OR "Iraq" OR "Israel" OR "Jordan" OR "Kuwait" OR "Lebanon" OR "Oman" OR "Qatar" OR "Saudi Arabia" OR "Syria" OR "Turkey" OR "United Arab Emirates" OR "Yemen" OR "Fiji" OR "New Caledonia" OR "Papua New Guinea" OR "Vanuatu" OR "Micronesia" OR "Melanesia" OR "Guam" OR "Palau" OR "Polynesia" OR "Samoa" OR "Tonga" OR "Armenia" OR "Azerbaijan" OR "Georgia" OR "Albania" OR "Estonia" OR "Latvia" OR "Lithuania" OR "Bosnia" OR "Herzegovina" OR "Serbia" OR "Bulgaria" OR "Belarus" OR "Croatia" OR "Czech Republic" OR "Hungary" OR "Macedonia" OR "Moldova" OR "Montenegro" OR "Poland" OR "Romania" OR "Russia" OR "Bashkiria" OR "Dagestan" OR "Slovakia" OR "Slovenia" OR "Ukraine" OR "Cameroon" OR "Central African Republic" OR "Chad" OR "Congo" OR "Equatorial Guinea" OR "Gabon" OR "Burundi" OR "Djibouti" OR "Eritrea" OR "Ethiopia" OR "Kenya" OR "Rwanda" OR "Somalia" OR "Sudan" OR "Tanzania" OR "Uganda" OR "Angola" OR "Botswana" OR "Lesotho" OR "Malawi" OR "Mozambique" OR "Namibia" OR "South Africa" OR "Swaziland" OR "Zambia" OR "Zimbabwe" OR "Benin" OR "Burkina Faso" OR "Cote d'Ivoire" OR "Gambia" OR "Ghana" OR "Guinea" OR "Guinea\-Bissau" OR "Liberia" OR " Mali" OR "Mauritania" OR "Niger" OR "Nigeria" OR "Senegal" OR "Sierra Leone" OR "Togo" OR "Algeria" OR "Egypt" OR "Libya" OR "Morocco" OR "Tunisia" OR "Comoros" OR "Madagascar" OR "Mauritius" OR "Reunion" OR "Seychelles" OR "Cabo Verde" OR "Kiribati" OR "Marshall Islands" OR "Nauru" OR "Niue" OR "Sao Tome" OR "Solomon Islands" OR "South Sudan" OR "Developing Countries" OR "Developing" OR "Least Developed" OR "Less\-Developed" OR "Less Developed" OR "Under\-Developed" OR "Under Developed" OR "UnderDeveloped" OR "third\-world" OR "third world" OR "Africa" OR "Africa" OR "Caribbean Region" OR "Caribbean Region" OR "West Indies" OR "Pacific Islands" OR "Pacific Islands" OR "Micronesia" OR "Melanesia" OR "Polynesia" OR "Mexico" OR "Mexico" OR "Latin America" OR "Latin America" OR "South America" OR "South America" OR "Indian Ocean Islands" OR "Indian Ocean Islands" OR "Pemba" OR "Cocos" OR "Maldives" OR "Central America" OR "Central America" OR "Asia" OR "Asia" OR "far east" OR "eastern europe" OR "eastern europe" OR "province" OR "provinces" OR "district" OR "districts" OR "prefecture" OR "prefectures" OR "county" OR "counties" OR "municipality" OR "municipalities"))))	
Web of Science	TOPIC: ("Infant" OR "Infant, Newborn" OR "child" OR "Child, Preschool" OR "Minors" OR "infant" OR "infants" OR "neonate" OR "neonates" OR "neonatal" OR "newborn" OR "newborns" OR "new-born" OR "new-borns" OR "baby" OR "babies" OR "Premature" OR "preterm" OR "pre term" OR "child" OR "children" OR "youth" OR "youths" OR "young people" OR "childhood" OR "toddler" OR "toddlers" OR "kid" OR "kids" OR "young patient" OR "young patients" OR "boy" OR "boys" OR "girl" OR "girls" OR "young age" OR "pediatric" OR "pre-schooler" OR "preschooler" OR "under 5" OR "under five" OR "under fives" OR "less than five" OR "perinatal")<br>AND	January 16, 2018

	TOPIC: ("Mortality" OR "Mortalities" OR "fatal" OR "fatality" OR "fatalities" OR "death" OR "deaths")<br>AND<br>TOPIC: ("Diarrhea" OR "Diarrhea" OR "Diarrheas" OR "Diarrheal" OR "diarrhoeal" OR "diarrhoea" OR "diarrhoeas" OR "Dysentery" OR "Dysentery" OR "dysenteria" OR "enteritis" OR "enteritides" OR "Cholera" OR "Cholera" OR "Choleras" OR "vibrio Cholerae" OR "Gastroenteritis" OR "gastroenteritis" OR "gastroenteritides" OR "gastro enteritis" OR "gastroduodenitis" OR "gastrointestinal acute infection" OR "gastrointestinal acute infections" OR "gastrointestinal infection" OR "gastrointestinal infections" OR "digestive tract infection" OR "digestive tract infections" OR "digestive infection" OR "gastrointestinal tract infection" OR "gastrointestine tract infection" OR "Pneumonia" OR "pneumonia" OR "pneumonias" OR "pneumonitis" OR "pulmonary inflammation" OR "pulmonary inflammations" OR "lung inflammation" OR "Lung Inflammations" OR "inflammatory lung disease" OR "lobitis" OR "peripneumonia" OR "pleuropneumonia" OR "pleuropneumonitis" OR "pneumonitis" OR "pulmonal inflammation" OR "pulmonary inflammation" OR "pulmonic inflammation" OR "bronchopneumonia" OR "bronchopneumonias" OR "Respiratory Tract Infections" OR "Respiratory Tract Infections" OR "Respiratory Tract Infection" OR "Respiratory Infection" OR "Respiratory Infections" OR "whooping cough" OR "pertussis" OR "pulmonary tract infection" OR "respiration infection" OR "Bronchiolitis" OR " bronchitis" OR "bronchopulmonary infection" OR "Croup" OR "Croup" OR "diphtheria" OR "laryngitis" OR "Severe Acute Respiratory Syndrome" OR "Severe Acute Respiratory Syndrome" OR "SARS" OR "Acute Chest Syndrome" OR "acute chest syndrome" OR "acute chest syndromes" OR "Acute Lower Respiratory Infection" OR "Acute Lower Respiratory Infections" OR "ALRI" OR "LRI" OR "ARI" OR "respiration tract infection" OR "respiration tract infections" OR "legionnaire disease" OR "legionnaires’ disease" OR "legionnaires disease" OR "Pontiac fever" OR "lung infiltrate" OR "lung infiltration" OR "pulmonary infiltrate" OR "pulmonary infiltration" OR "pneumonic lung" OR "rds" OR "respiratory distress" OR "respiration distress" OR "Malaria" OR "malaria" OR "malarias" OR "Plasmodium Infection" OR "Plasmodium Infections" OR "Plasmodium falciparum" OR "Plasmodium falciparum" OR "Plasmodium falciparums" OR "malaria, falciparum" OR "blackwater fever" OR "blackwater fevers" OR "black water fever" OR "black water fevers" OR "remittent fever" OR "paludism" OR "plasmodium infection" OR "plasmodium infections" OR "marsh fever" OR "falciparum infection" OR "Plasmodium vivax" OR "Plasmodium vivax" OR "Plasmodium vivaxs" OR "malaria, vivax" OR "vivax infection" OR "Plasmodium ovale" OR "Plasmodium ovale" OR "Plasmodium ovales" OR "Plasmodium malariae" OR "Plasmodium malariae" OR "Meningitis" OR "Meningitis" OR "Meningitides" OR "Pachymeningitis" OR "Pachymeningitides" OR "Arachnoiditis" OR "Arachnoiditides" OR "Arachnoid Membrane inflammation" OR "Arachnoid Membrane inflammations" OR "Meningoencephalitides" OR "Meningoencephalitis" OR "Meningocephalitis" OR "Cerebromeningitis" OR "Encephalomeningitis" OR "Encephalomeningitides" OR "Haemophilus influenzae" OR "Haemophilus parainfluenzae" OR "Cerebromeningitides" OR "Cerebritis" OR "Cerebritides" OR "Waterhouse Friderichsen Syndrome" OR "Waterhouse Friederichsen Syndrome" OR "Waterhouse-Friderichsen Syndrome" OR "Waterhouse-Friederichsen Syndrome" OR "Purpura Fulminans" OR "Meningococcal" OR "Meningeal" OR "Cerebral Cryptococcosis" OR "Cerebral Cryptococcoses" OR "Toruloma" OR "Torulomas" OR "Lymphocytic Choriomeningitis" OR "Armstrong Syndrome" OR "Armstrong’s Syndrome" OR "Encephalitis" "Encephalitis" OR "Encephalitides" OR "encephalitis" OR "brain inflammation" OR "brain inflammations" OR "Rasmussen syndrome" OR "Rasmussen’s syndrome" OR "Cerebral Ventriculitis" OR "Cerebral Ventriculitides" OR "Infectious Ventriculitis" OR "Infectious Ventriculitides" OR "Encephalomyelitis" OR "Meningoencephalitides" OR "Meningoencephalitis" OR "Cerebromeningitis" OR "myeloencephalitis" OR "Lupus Vasculitis" OR "Central Nervous System Lupus" OR "Lupus Erythematosis" OR "Lupus Erythematosus" OR "brain vasculitis" OR "brain angiitis" OR "brain arteritis" OR "cerebral arteritis" OR "cerebral vasculitis" OR "Neisseria meningitidis" OR "Neisseria meningitidis" OR "Neisseria meningitides" OR "Micrococcus intracellularis" OR "Neisseria weichselbaumi" OR "Meningococcus" OR "meningococcemia" OR "meningococci" OR "Meningococcal Infections" OR "meningococceal" OR "meningococcaemia" OR "sepsis" OR "sepsis" OR "Pyemia" OR "Pyemias" OR "Pyohemia" OR "Pyohemias" OR "Pyaemia" OR "Pyaemias" OR "Septicemia" OR "Septicemias" OR "Blood Poisoning" OR "Blood Poisonings" OR "septic" OR "sepses" OR "congenital abnormalities" OR "congenital" OR "Deformities" OR "Deformity" OR "Birth Defects" OR "Birth Defect" OR "abnormality" OR "abnormalities" OR "malformation" OR "malformations" OR "malformative" OR "malformed" OR "neural tube defects" OR "neural tube" OR "Craniorachischisis" OR "Craniorachischises" OR "Diastematomyelia" OR "Diastematomyelias" OR "Tethered Cord" OR "Spinal Dysraphism" OR "Tethered Spinal Cord" OR "Spinal Dysraphisms" OR "Iniencephaly" OR "Iniencephalies" OR "Neurenteric" OR "Neuroenteric" OR "Spinal Cord Myelodysplasia" OR "Spinal Cord Myelodysplasias" OR "Acrania" OR "Acranias" OR "Exencephaly" OR "Exencephalies" OR "dysgraphia" OR "dysraphic" OR "dysraphism" OR "dysraphy" OR "NTD" OR "NTDS" OR "sudden infant death syndrome" OR "sudden infant death" OR "sid" OR "SIDS" OR "cot death" OR "cot deaths" OR "crib death" OR "fetal alcohol spectrum disorders" OR "Fetal alcohol spectrum" OR "Foetal alcohol spectrum" OR "FASD" OR "FASDs" OR "Fetal Alcohol Syndrome" OR "Alcohol-Related Birth Defects" OR "Alcohol Related Birth Defects" OR "Alcohol Related Neurodevelopmental Disorder" OR "FAE" OR "Fetal Alcohol Effects" OR "FAEs" OR "Fetal Alcohol Syndrome" OR "Fetus Alcohol Syndrome" OR "Foetal alcohol syndrome" OR "premature birth" OR "premature" OR "preterm" OR "pre-mature" OR "pre-term" OR "prematurity" OR "prematuritas" OR "asphyxia" OR "asphyxia" OR "Asphyxia Neonatorum" OR "Asphyxia Neonatorum" OR "hypoxia" OR "Asphyxias" OR "Suffocation" OR "Suffocations" OR "anoxia" OR ("intrapartum" AND "related") OR "tetanus" OR "tetanus" OR "tetani" OR "diphtheria-tetanus-pertussis" OR "rubella" OR "rubella" OR "measles" OR "epidemic roseola" OR "necrosis" OR "necrosis" OR "necrotic" OR "necroses" OR "necrotizing" OR "Infarction" OR "infractions" OR "birth injuries" OR "birth injuries" OR "birth injury" OR "birth	

	trauma" OR "Obstetric Paralyses" OR "Obstetric Paralysis" OR "Obstetrical Paralyses" OR "Obstetrical Paralysis" OR "birth damage" OR "birth lesion" OR "birth palsy" OR "birth paralysis" OR "obstetric palsy" OR "obstetrical palsy" OR "brachial palsy" OR "brachial paralysis" OR "brachial plexus paralysis" OR "delivery trauma" OR "arm paralysis" OR "paralysis brachialis" OR "jaundice" OR "jaundice" OR "jaundices" OR "Icterus" OR "bronze baby syndrome" OR "erythroleukoblastosis" OR "hemorrhage" OR "hemorrhage" "haemorrhage" OR "haemorrhagic" OR "Hemorrhagic" OR "Nutrition disorders" OR "malnutrition" OR "Nutritional" OR "Undernutrition" OR "Malnourishment" OR " Malnourishments" OR "Deficiency" OR "Deficiencies" OR "Refeeding Syndrome" OR "Starvation" OR "Starvations" OR "Famine" OR "Famines" OR "deficient" OR "underfeeding" OR "undernourishment" OR "nutrition" OR "Hypervitaminosis" OR "Noncommunicable Diseases" OR "Noncommunicable" OR "Non-communicable" OR "communicable" OR "non-infectious" OR "infectious" OR "noninfectious" OR "Communicable Diseases" OR "Infant, Newborn, Diseases" OR "newborn" OR "neonatal" OR "perinatal")<br>AND<br>TOPIC: ("Argentina" OR "Bolivia" OR "Brazil" OR "Brasil" OR "Chile" OR "Colombia" OR "Ecuador" OR "French Guiana" OR "Guyana" OR "Paraguay" OR "Peru" OR "Suriname" OR "Uruguay" OR "Venezuela" OR "Mexico" OR "Belize" OR "Costa Rica" OR "El Salvador" OR "Guatemala" OR "Honduras" OR "Nicaragua" OR "Puerto Rico" OR "Panama" OR "West Indies" OR "Antigua" OR "Barbuda" OR "Bahamas" OR "Barbados" OR "Cuba" OR "Dominica" OR "Dominican Republic" OR "Grenada" OR "Guadeloupe" OR "Haiti" OR "Jamaica" OR "Martinique" OR "Antilles" OR "Anguilla" OR "Saint Kitts" OR "St Kitts" OR "Saint Lucia" OR "St Lucia" OR "Saint Vincent" OR "St Vincent" OR "Trinidad " OR "Tobago" OR "Virgin Islands" OR "Kazakhstan" OR "Kyrgyzstan" OR "Tajikistan" OR "Turkmenistan" OR "Uzbekistan" OR "Borneo" OR "Brunei" OR "Cambodia" OR "East Timor" OR "Indonesia" OR "Laos" OR "Malaysia" OR "Mekong Valley" OR "Myanmar" OR "Burma" OR "Philippines" OR "Singapore" OR "Thailand" OR "Vietnam" OR "Bangladesh" OR "Bhutan" OR "India" OR "Nepal" OR "Pakistan" OR "Sri Lanka" OR "China" OR "Korea" OR "Macao" OR "Macau" OR "Mongolia" OR "Taiwan" OR "Afghanistan" OR "Bahrain" OR "Iran" OR "Iraq" OR "Israel" OR "Jordan" OR "Kuwait" OR "Lebanon" OR "Oman" OR "Qatar" OR "Saudi Arabia" OR "Syria" OR "Turkey" OR "United Arab Emirates" OR "Yemen" OR "Fiji" OR "New Caledonia" OR "Papua New Guinea" OR "Vanuatu" OR "Micronesia" OR "Melanesia" OR "Guam" OR "Palau" OR "Polynesia" OR "Samoa" OR "Tonga" OR "Armenia" OR "Azerbaijan" OR "Georgia" OR "Albania" OR "Estonia" OR "Latvia" OR "Lithuania" OR "Bosnia" OR "Herzegovina" OR "Serbia" OR "Bulgaria" OR "Belarus" OR "Croatia" OR "Czech Republic" OR "Hungary" OR "Macedonia" OR "Moldova" OR "Montenegro" OR "Poland" OR "Romania" OR "Russia" OR "Bashkiria" OR "Dagestan" OR "Slovakia" OR "Slovenia" OR "Ukraine" OR "Cameroon" OR "Central African Republic" OR "Chad" OR "Congo" OR "Equatorial Guinea" OR "Gabon" OR "Burundi" OR "Djibouti" OR "Eritrea" OR "Ethiopia" OR "Kenya" OR "Rwanda" OR "Somalia" OR "Sudan" OR "Tanzania" OR "Uganda" OR "Angola" OR "Botswana" OR "Lesotho" OR "Malawi" OR "Mozambique" OR "Namibia" OR "South Africa" OR "Swaziland" OR "Zambia" OR "Zimbabwe" OR "Benin" OR "Burkina Faso" OR "Cote d'Ivoire" OR "Gambia" OR "Ghana" OR "Guinea" OR "Guinea-Bissau" OR "Liberia" OR " Mali" OR "Mauritania" OR "Niger" OR "Nigeria" OR "Senegal" OR "Sierra Leone" OR "Togo" OR "Algeria" OR "Egypt" OR "Libya" OR "Morocco" OR "Tunisia" OR "Comoros" OR "Madagascar" OR "Mauritius" OR "Reunion" OR "Seychelles" OR "Cabo Verde" OR "Kiribati" OR "Marshall Islands" OR "Nauru" OR "Niue" OR "Sao Tome" OR "Solomon Islands" OR "South Sudan" OR "Developing Countries" OR "Developing" OR "Least Developed" OR "Less-Developed" OR "Less Developed" OR "Under-Developed" OR "Under Developed" OR "UnderDeveloped" OR "third-world" OR "third world" OR "Africa" OR "Africa" OR "Caribbean Region" OR "Caribbean Region" OR "West Indies" OR "Pacific Islands" OR "Pacific Islands" OR "Micronesia" OR "Melanesia" OR "Polynesia" OR "Mexico" OR "Mexico" OR "Latin America" OR "Latin America" OR "South America" OR "South America" OR "Indian Ocean Islands" OR "Indian Ocean Islands" OR "Pemba" OR "Cocos" OR "Maldives" OR "Central America" OR "Central America" OR "Asia" OR "Asia" OR "far east" OR "eastern europe" OR "eastern europe" OR "province" OR "provinces" OR "district" OR "districts" OR "prefecture" OR "prefectures" OR "county" OR "counties" OR "municipality" OR "municipalities")<br>NOT<br>DT=(Discussion OR Editorial Material OR Letter OR Note)	
Africa-Wide Information	Select a Field (Optional): "Infant" OR "Infant, Newborn" OR "child" OR "Child, Preschool" OR "Minors" OR "infant" OR "infants" OR "neonate" OR "neonates" OR "neonatal" OR "newborn" OR "newborns" OR "new-born" OR "new-borns" OR "baby" OR "babies" OR "Premature" OR "preterm" OR "pre term" OR "child" OR "children" OR "youth" OR "youths" OR "young people" OR "childhood" OR "toddler" OR "toddlers" OR "kid" OR "kids" OR "young patient" OR "young patients" OR "boy" OR "boys" OR "girl" OR "girls" OR "young age" OR "pediatric" OR "pre-schooler" OR "preschooler" OR "under 5" OR "under five" OR "under fives" OR "less than five" OR "perinatal"<br>AND<br>Select a Field (Optional): "Mortality" OR "Mortality" OR "Mortalities" OR "fatal" OR "fatality" OR "fatalities" OR "Death" OR "death" OR " deaths" OR "Child Mortality"<br>AND<br>Select a Field (Optional): "Diarrhea" OR "Diarrhea" OR "Diarrheas" OR "Diarrheal" OR "diarrhoeal" OR "diarrhoea" OR "diarrhoeas" OR "Dysentery" OR "Dysentery" OR "dysenteria" OR "enteritis" OR "enteritides" OR "Cholera" OR "Cholera" OR "Choleras" OR "vibrio Cholerae" OR "Gastroenteritis" OR "gastroenteritis" OR "gastroenteritides" OR "gastro enteritis" OR "gastroduodenitis" OR "gastrointestinal acute infection" OR "gastrointestinal acute	January 19, 2018

	infections" OR "gastrointestinal infection" OR "gastrointestinal infections" OR "digestive tract infection" OR "digestive tract infections" OR "digestive infection" OR "gastrointestinal tract infection" OR "gastrointestine tract infection" OR "Pneumonia" OR "pneumonia" OR "pneumonias" OR "pneumonitis" OR "pulmonary inflammation" OR "pulmonary inflammations" OR "lung inflammation" OR "Lung Inflammations" OR "inflammatory lung disease" OR "lobitis" OR "peripneumonia" OR "pleuropneumonia" OR "pleuropneumonitis" OR "pneumonitis" OR "pulmonal inflammation" OR "pulmonary inflammation" OR "pulmonic inflammation" OR "bronchopneumonia" OR "bronchopneumonias" OR "Respiratory Tract Infections" OR "Respiratory Tract Infections" OR "Respiratory Tract Infection" OR "Respiratory Infection" OR "Respiratory Infections" OR "whooping cough" OR "pertussis" OR "pulmonary tract infection" OR "respiration infection" OR "Bronchiolitis" OR " bronchitis" OR "bronchopulmonary infection" OR "Croup" OR "Croup" OR "diphtheria" OR "laryngitis" OR "Severe Acute Respiratory Syndrome" OR "Severe Acute Respiratory Syndrome" OR "SARS" OR "Acute Chest Syndrome" OR "acute chest syndrome" OR "acute chest syndromes" OR "Acute Lower Respiratory Infection" OR "Acute Lower Respiratory Infections" OR "ALRI" OR "LRI" OR "ARI" OR "respiration tract infection" OR "respiration tract infections" OR "legionnaire disease" OR "legionnaires’ disease" OR "legionnaires disease" OR "Pontiac fever" OR "lung infiltrate" OR "lung infiltration" OR "pulmonary infiltrate" OR "pulmonary infiltration" OR "pneumonic lung" OR "rds" OR "respiratory distress" OR "respiration distress" OR "Malaria" OR "malaria" OR "malarias" OR "Plasmodium Infection" OR "Plasmodium Infections" OR "Plasmodium falciparum" OR "Plasmodium falciparum" OR "Plasmodium falciparums" OR "malaria, falciparum" OR "blackwater fever" OR "blackwater fevers" OR "black water fever" OR "black water fevers" OR "remittent fever" OR "paludism" OR "plasmodium infection" OR "plasmodium infections" OR "marsh fever" OR "falciparum infection" OR "Plasmodium vivax" OR "Plasmodium vivax" OR "Plasmodium vivaxs" OR "malaria, vivax" OR "vivax infection" OR "Plasmodium ovale" OR "Plasmodium ovale" OR "Plasmodium ovales" OR "Plasmodium malariae" OR "Plasmodium malariae" OR "Meningitis" OR "Meningitis" OR "Meningitides" OR "Pachymeningitis" OR "Pachymeningitides" OR "Arachnoiditis" OR "Arachnoiditides" OR "Arachnoid Membrane inflammation" OR "Arachnoid Membrane inflammations" OR "Meningoencephalitides" OR "Meningoencephalitis" OR "Meningocephalitis" OR "Cerebromeningitis" OR "Encephalomeningitis" OR "Encephalomeningitides" OR "Haemophilus influenzae" OR "Haemophilus parainfluenzae" OR "Cerebromeningitides" OR "Cerebritis" OR "Cerebritides" OR "Waterhouse Friderichsen Syndrome" OR "Waterhouse Friederichsen Syndrome" OR "Waterhouse-Friderichsen Syndrome" OR "Waterhouse-Friederichsen Syndrome" OR "Purpura Fulminans" OR "Meningococcal" OR "Meningeal" OR "Cerebral Cryptococcosis" OR "Cerebral Cryptococcoses" OR "Toruloma" OR "Torulomas" OR "Lymphocytic Choriomeningitis" OR "Armstrong Syndrome" OR "Armstrong’s Syndrome" OR "Encephalitis" "Encephalitis" OR "Encephalitides" OR "encephalitis" OR "brain inflammation" OR "brain inflammations" OR "Rasmussen syndrome" OR "Rasmussen’s syndrome" OR "Cerebral Ventriculitis" OR "Cerebral Ventriculitides" OR "Infectious Ventriculitis" OR "Infectious Ventriculitides" OR "Encephalomyelitis" OR "Meningoencephalitides" OR "Meningoencephalitis" OR "Cerebromeningitis" OR "myeloencephalitis" OR "Lupus Vasculitis" OR "Central Nervous System Lupus" OR "Lupus Erythematosis" OR "Lupus Erythematosus" OR "brain vasculitis" OR "brain angiitis" OR "brain arteritis" OR "cerebral arteritis" OR "cerebral vasculitis" OR "Neisseria meningitidis" OR "Neisseria meningitidis" OR "Neisseria meningitides" OR "Micrococcus intracellularis" OR "Neisseria weichselbaumi" OR "Meningococcus" OR "meningococcemia" OR "meningococci" OR "Meningococcal Infections" OR "meningococceal" OR "meningococcaemia" OR "sepsis" OR "sepsis" OR "Pyemia" OR "Pyemias" OR "Pyohemia" OR "Pyohemias" OR "Pyaemia" OR "Pyaemias" OR "Septicemia" OR "Septicemias" OR "Blood Poisoning" OR "Blood Poisonings" OR "septic" OR "sepses" OR "congenital abnormalities" OR "congenital" OR "Deformities" OR "Deformity" OR "Birth Defects" OR "Birth Defect" OR "abnormality" OR "abnormalities" OR "malformation" OR "malformations" OR "malformative" OR "malformed" OR "neural tube defects" OR "neural tube" OR "Craniorachischisis" OR "Craniorachischises" OR "Diastematomyelia" OR "Diastematomyelias" OR "Tethered Cord" OR "Spinal Dysraphism" OR "Tethered Spinal Cord" OR "Spinal Dysraphisms" OR "Iniencephaly" OR "Iniencephalies" OR "Neurenteric" OR "Neuroenteric" OR "Spinal Cord Myelodysplasia" OR "Spinal Cord Myelodysplasias" OR "Acrania" OR "Acranias" OR "Exencephaly" OR "Exencephalies" OR "dysgraphia" OR "dysraphic" OR "dysraphism" OR "dysraphy" OR "NTD" OR "NTDS" OR "sudden infant death syndrome" OR "sudden infant death" OR "sid" OR "SIDS" OR "cot death" OR "cot deaths" OR "crib death" OR "fetal alcohol spectrum disorders" OR "Fetal alcohol spectrum" OR "Foetal alcohol spectrum" OR "FASD" OR "FASDs" OR "Fetal Alcohol Syndrome" OR "Alcohol-Related Birth Defects" OR "Alcohol Related Birth Defects" OR "Alcohol Related Neurodevelopmental Disorder" OR "FAE" OR "Fetal Alcohol Effects" OR "FAEs" OR "Fetal Alcohol Syndrome" OR "Fetus Alcohol Syndrome" OR "Foetal alcohol syndrome" OR "premature birth" OR "premature" OR "preterm" OR "pre-mature" OR "pre-term" OR "prematurity" OR "prematuritas" OR "asphyxia" OR "asphyxia" OR "Asphyxia Neonatorum" OR "Asphyxia Neonatorum" OR "hypoxia" OR "Asphyxias" OR "Suffocation" OR "Suffocations" OR "anoxia" OR ("intrapartum" AND "related") OR "tetanus" OR "tetanus" OR "tetani" OR "diphtheria-tetanus-pertussis" OR "rubella" OR "rubella" OR "measles" OR "epidemic roseola" OR "necrosis" OR "necrosis" OR "necrotic" OR "necroses" OR "necrotizing" OR "Infarction" OR "infractions" OR "birth injuries" OR "birth injuries" OR "birth injury" OR "birth trauma" OR "Obstetric Paralyses" OR "Obstetric Paralysis" OR "Obstetrical Paralyses" OR "Obstetrical Paralysis" OR "birth damage" OR "birth lesion" OR "birth palsy" OR "birth paralysis" OR "obstetric palsy" OR "obstetrical palsy" OR "brachial palsy" OR "brachial paralysis" OR "brachial plexus paralysis" OR "delivery trauma" OR "arm paralysis" OR "paralysis brachialis" OR "jaundice" OR "jaundice" OR "jaundices" OR "Icterus" OR "bronze baby syndrome" OR "erythroleukoblastosis" OR "hemorrhage" OR "hemorrhage" "haemorrhage" OR "haemorrhagic" OR "Hemorrhagic" OR "Nutrition disorders" OR "malnutrition" OR "Nutritional" OR "Undernutrition" OR	

	"Malnourishment" OR " Malnourishments" OR "Deficiency" OR "Deficiencies" OR "Refeeding Syndrome" OR "Starvation" OR "Starvations" OR "Famine" OR "Famines" OR "deficient" OR "underfeeding" OR "undernourishment" OR "nutrition" OR "Hypervitaminosis" OR "Noncommunicable Diseases" OR "Noncommunicable" OR "Non-communicable" OR "communicable" OR "non-infectious" OR "infectious" OR "noninfectious" OR "Communicable Diseases" OR "Infant, Newborn, Diseases" OR "newborn" OR "neonatal" OR "perinatal"<br>AND<br>Select a Field (Optional): "Argentina" OR "Bolivia" OR "Brazil" OR "Brasil" OR "Chile" OR "Colombia" OR "Ecuador" OR "French Guiana" OR "Guyana" OR "Paraguay" OR "Peru" OR "Suriname" OR "Uruguay" OR "Venezuela" OR "Mexico" OR "Belize" OR "Costa Rica" OR "El Salvador" OR "Guatemala" OR "Honduras" OR "Nicaragua" OR "Puerto Rico" OR "Panama" OR "West Indies" OR "Antigua" OR "Barbuda" OR "Bahamas" OR "Barbados" OR "Cuba" OR "Dominica" OR "Dominican Republic" OR "Grenada" OR "Guadeloupe" OR "Haiti" OR "Jamaica" OR "Martinique" OR "Antilles" OR "Anguilla" OR "Saint Kitts" OR "St Kitts" OR "Saint Lucia" OR "St Lucia" OR "Saint Vincent" OR "St Vincent" OR "Trinidad " OR "Tobago" OR "Virgin Islands" OR "Kazakhstan" OR "Kyrgyzstan" OR "Tajikistan" OR "Turkmenistan" OR "Uzbekistan" OR "Borneo" OR "Brunei" OR "Cambodia" OR "East Timor" OR "Indonesia" OR "Laos" OR "Malaysia" OR "Mekong Valley" OR "Myanmar" OR "Burma" OR "Philippines" OR "Singapore" OR "Thailand" OR "Vietnam" OR "Bangladesh" OR "Bhutan" OR "India" OR "Nepal" OR "Pakistan" OR "Sri Lanka" OR "China" OR "Korea" OR "Macao" OR "Macau" OR "Mongolia" OR "Taiwan" OR "Afghanistan" OR "Bahrain" OR "Iran" OR "Iraq" OR "Israel" OR "Jordan" OR "Kuwait" OR "Lebanon" OR "Oman" OR "Qatar" OR "Saudi Arabia" OR "Syria" OR "Turkey" OR "United Arab Emirates" OR "Yemen" OR "Fiji" OR "New Caledonia" OR "Papua New Guinea" OR "Vanuatu" OR "Micronesia" OR "Melanesia" OR "Guam" OR "Palau" OR "Polynesia" OR "Samoa" OR "Tonga" OR "Armenia" OR "Azerbaijan" OR "Georgia" OR "Albania" OR "Estonia" OR "Latvia" OR "Lithuania" OR "Bosnia" OR "Herzegovina" OR "Serbia" OR "Bulgaria" OR "Belarus" OR "Croatia" OR "Czech Republic" OR "Hungary" OR "Macedonia" OR "Moldova" OR "Montenegro" OR "Poland" OR "Romania" OR "Russia" OR "Bashkiria" OR "Dagestan" OR "Slovakia" OR "Slovenia" OR "Ukraine" OR "Cameroon" OR "Central African Republic" OR "Chad" OR "Congo" OR "Equatorial Guinea" OR "Gabon" OR "Burundi" OR "Djibouti" OR "Eritrea" OR "Ethiopia" OR "Kenya" OR "Rwanda" OR "Somalia" OR "Sudan" OR "Tanzania" OR "Uganda" OR "Angola" OR "Botswana" OR "Lesotho" OR "Malawi" OR "Mozambique" OR "Namibia" OR "South Africa" OR "Swaziland" OR "Zambia" OR "Zimbabwe" OR "Benin" OR "Burkina Faso" OR "Cote d'Ivoire" OR "Gambia" OR "Ghana" OR "Guinea" OR "Guinea-Bissau" OR "Liberia" OR " Mali" OR "Mauritania" OR "Niger" OR "Nigeria" OR "Senegal" OR "Sierra Leone" OR "Togo" OR "Algeria" OR "Egypt" OR "Libya" OR "Morocco" OR "Tunisia" OR "Comoros" OR "Madagascar" OR "Mauritius" OR "Reunion" OR "Seychelles" OR “Cabo Verde” OR “Kiribati” OR “Marshall Islands” OR “Nauru” OR “Niue” OR “Sao Tome” OR “Solomon Islands” OR “South Sudan" OR "Developing Countries" OR "Developing" OR "Least Developed" OR "Less-Developed" OR "Less Developed" OR "Under-Developed" OR "Under Developed" OR "UnderDeveloped" OR "third-world" OR "third world" OR "Africa" OR "Africa" OR "Caribbean Region" OR "Caribbean Region" OR "West Indies" OR "Pacific Islands" OR "Pacific Islands" OR "Micronesia" OR "Melanesia" OR "Polynesia" OR "Mexico" OR "Mexico" OR "Latin America" OR "Latin America" OR "South America" OR "South America" OR "Indian Ocean Islands" OR "Indian Ocean Islands" OR "Pemba" OR "Cocos" OR "Maldives" OR "Central America" OR "Central America" OR "Asia" OR "Asia" OR "far east" OR "eastern europe" OR "eastern europe" OR "province" OR "provinces" OR "district" OR "districts" OR "prefecture" OR "prefectures" OR "county" OR "counties" OR "municipality" OR "municipalities"	

Appendix 2: Updated study data points

Appendix 2. Updated study data points in 2000-2019

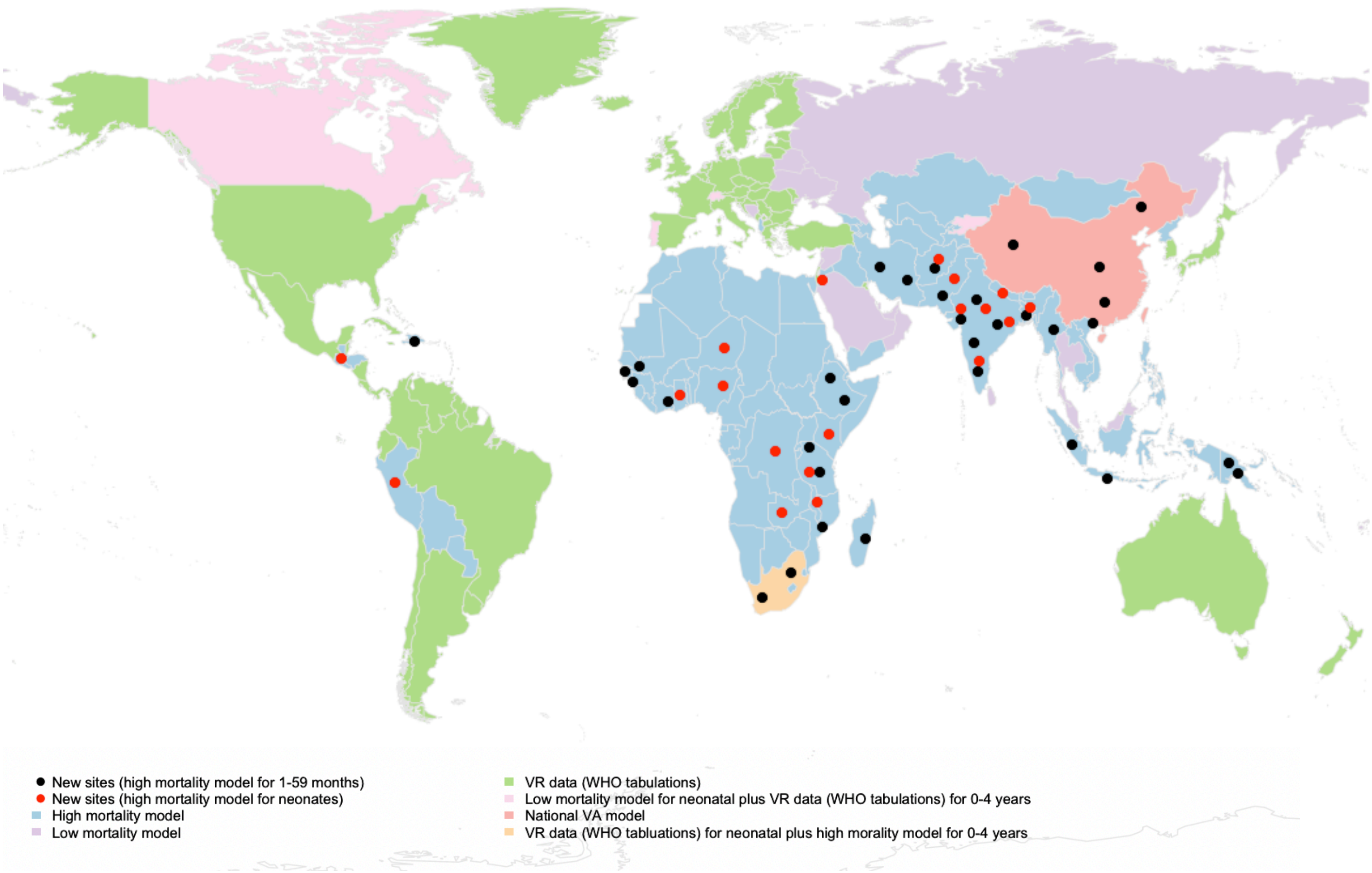

Appendix 3: Method details

ICD codes and cause of death

Appendix 3. Details of methods used to estimate causes of death in children younger than five years in 2000-2019

1. ICD codes and cause of death categories

MCEE cause name	ICD-10 code	ICD-9 code
**All causes**	A00-Y89	001-999
**I. Communicable, maternal, neonatal and nutritional conditions***	A00-B99, D50-D53, D64.9, E00- E02, E40-E64, G00, G03-G04, H65-H66, J00-J22, J85, N30, N34, N390, N70-N73, O00-P96, U04	001- 139, 243, 260- 269, 279.5-279.6, 280, 281, 285.9, 320- 323, 381- 382, 460- 466, 480- 487, 513, 614- 616, 630- 676, 760- 779
HIV/AIDS	B20-B24	279.5-279.6, 042
Diarrhoea diseases	A00-A09	001-009
Tetanus	A33-A35	33
Measles	B05	55
Meningitis/Encephalitis	A39, A83, A84-A87, G00, G03, G04	036, 047, 062-064, 320-322
Malaria	B50-B54, P373, P374	84
ARI	H65-H66, J00-J22, J85, P23	460- 466, 480- 487, 381- 382, 513, 770.0
Preterm birth complications	P01.0, P01.1, P07, P22, P25-P28, P61.2, P77	761.0-761.1, 765, 769, 770.2-770.9, 772.1, 774.2, 776.6, 777.5-777.6
Intrapartum-related complications	P01.7-P02.1, P02.4-P02.6, P03, P10-P15, P20-P21, P24, P50, P90-P91	761.7-762.1, 762.4-762.6, 763, 767-768, 770.1, 772.2, 779.0-779.2
Sepsis and other infectious conditions of the newborn	P35-P39 (excluding P37.3, P37.4)	771.0-771.2, 771.4-771.8
Other Group II	Remainder	Remainder
**II. Noncommunicable diseases**	C00-C97, D00-D48, D55-D64 (exclude D 64.9), D65- D89, E03- E34, E65-E88, F01-F99, G06- G98, H00- H61, H68-H93, I00- I99, J30-J84, J86-J98, K00-K92, L00-L98, M00-M99, N00-N28, N31-N32, N35-N64 (exclude N39.0), N75-N98, Q00-Q99	140- 242, 244- 259, 270- 279, 282-285, 286- 319, 324- 380, 383- 459, 470- 478, 490- 512, 514-611, 617-629, 680- 759 (exclude 279.5-279.6, 285.9)
Congenital abnormalities	Q00-Q99	740-759
Other Group II	Remainder	Remainder
**III. Injuries**	V01-Y89	E800-E999

Methods neonatal mortality

2. Methods 0-1 months

Models were estimated for the distribution of cause-specific neonatal mortality assuming a multinomial likelihood of deaths by cause. Reported causes were translated to causes of interest with the misclassification matrix, allowing for variable definition of "other" or residual causes across studies (for example not all studies report neonatal deaths due to diarrhea). Non-informative normal priors were used for multinomial regression coefficients that were not constrained by LASSO parameter λ. Parameter constrained by λ had double exponential priors with mean 0.

**Model; iterations**	**Covariates not constrained by λ**	**Covariates constrained by λ**	**Final λ**	**Priors**
Low mortality (early); 1000 iterations	Intercept	Gross national income (gni), fertility rate (gfr), Gini coefficient (gini), under-five mortality rate (u5mr), neonatal mortality rate (nmr), rate of low-weight births (lbwrate), DPT inoculation (dpt), antenatal care coverage (anc), women's literacy (femlit)	325	Beta (unconstrained): Normal(0, precision 4)<br><br>Beta (constrained): Double exponential(0,λ)<br><br>Random effects: Normal(0, precision 1/sd^2)<br><br>Random effect sd: Uniform(0, SD limit = 0.07)
Low mortality (late); 1000 iterations	Intercept	Gross national income (gni), Fertility rate (gfr), Gini coefficient (gini), under-five mortality rate (u5mr), neonatal mortality rate (nmr), rate of low-weight births (lbwrate), DPT inoculation (dpt), antenatal care coverage (anc), women's literacy (femlit)	400	Beta (unconstrained): Normal(0, precision 4)<br><br>Beta (constrained): Double exponential(0,λ)<br><br>Random effects: Normal(0, precision 1/sd^2)<br><br>Random effect sd: Uniform(0, SD limit = 0.07)
High mortality; 15000 iterations	Intercept, reported for early neonatal death (per.early), reported for late neonatal death (per.late)	under-five mortality rate (u5mr), neonatal mortality rate (nmr), DPT inoculation (dpt), fertility rate (gfr), rate of low-weight births (lbwrate), skilled birth attendance (sba), BCG inoculation (bcg), maternal tetanus inoculation (pab), women's literacy (femlit), sub-Sarharan Africa region (regSSA), Southeast Asia region (regSA), study distinguishes between preterm and low-birth weight (prevvslbw)	225	Beta (unconstrained): Normal(0, precision 4)<br><br>Beta (constrained): Double exponential(0,λ)<br><br>Random effects: Normal(0, precision 1/sd^2)<br><br>Random effect sd: Uniform(0, SD limit)

2.1 Low mortality early neonatal mortality model

Cross validation error to select Lambda for low mortality early neonatal model –Lambda 325

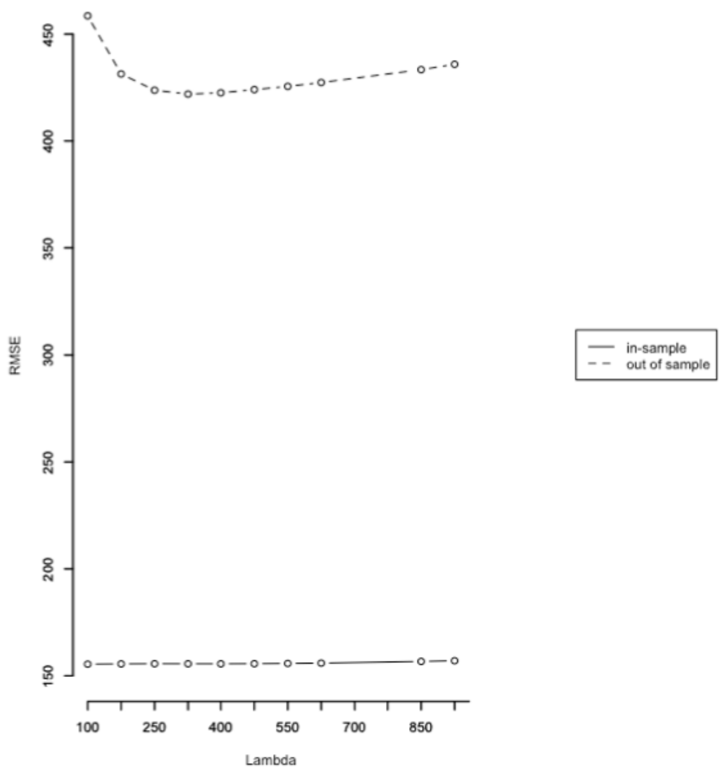

Coefficient estimates for low mortality early neonatal model (reference preterm birth complications)

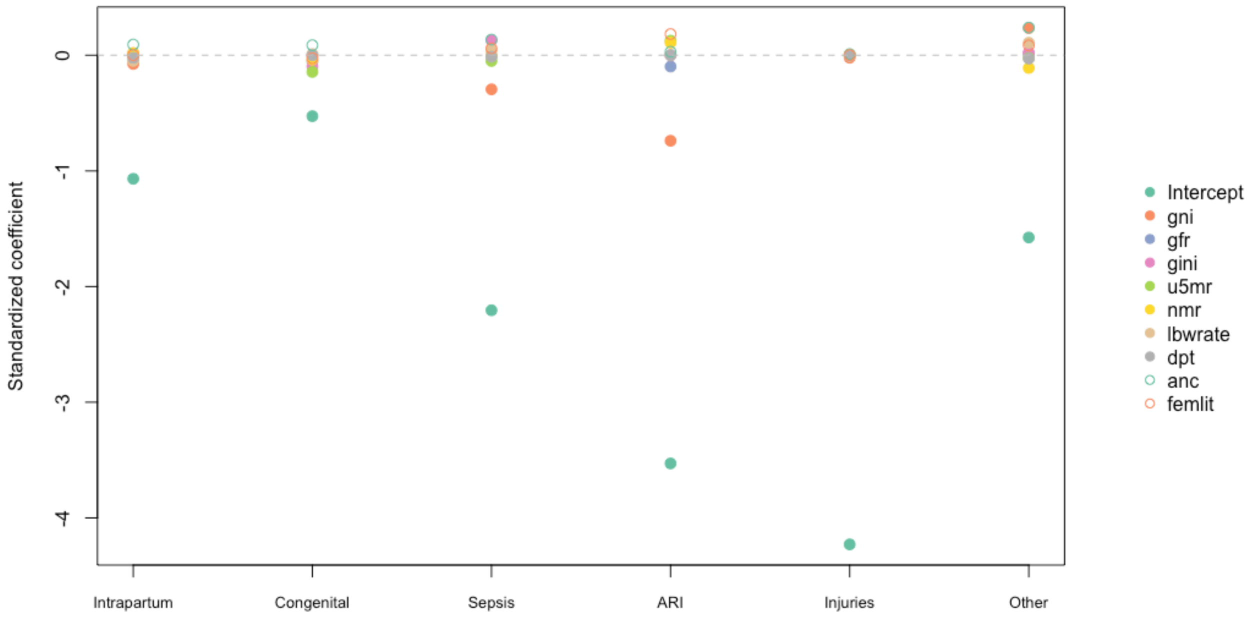

2.2 Low mortality late neonatal mortality model

Cross validation error to select Lambda for low mortality late neonatal model – Lambda 400

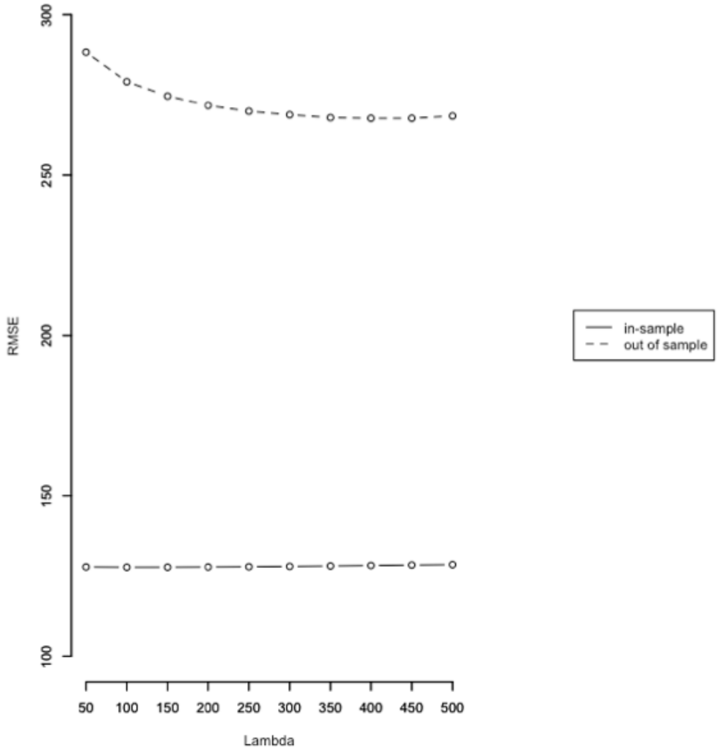

Coefficient estimates for low mortality late neonatal model (reference preterm birth complications)

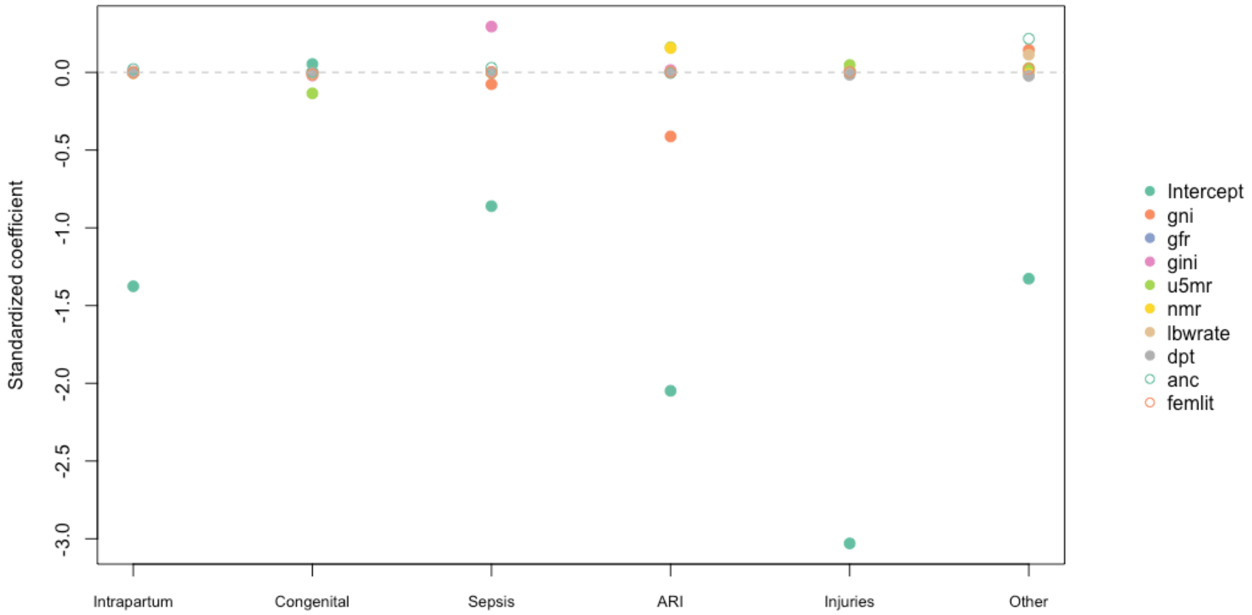

2.3 Neonatal high mortality model

Cross validation error to select Lambda for neonatal high mortality model – Lambda 225

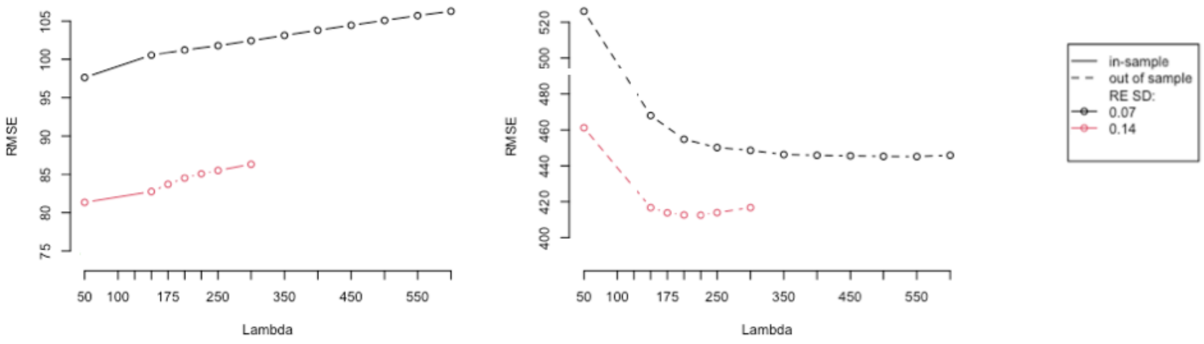

Coefficient estimates for neonatal high mortality model (reference intrapartum)

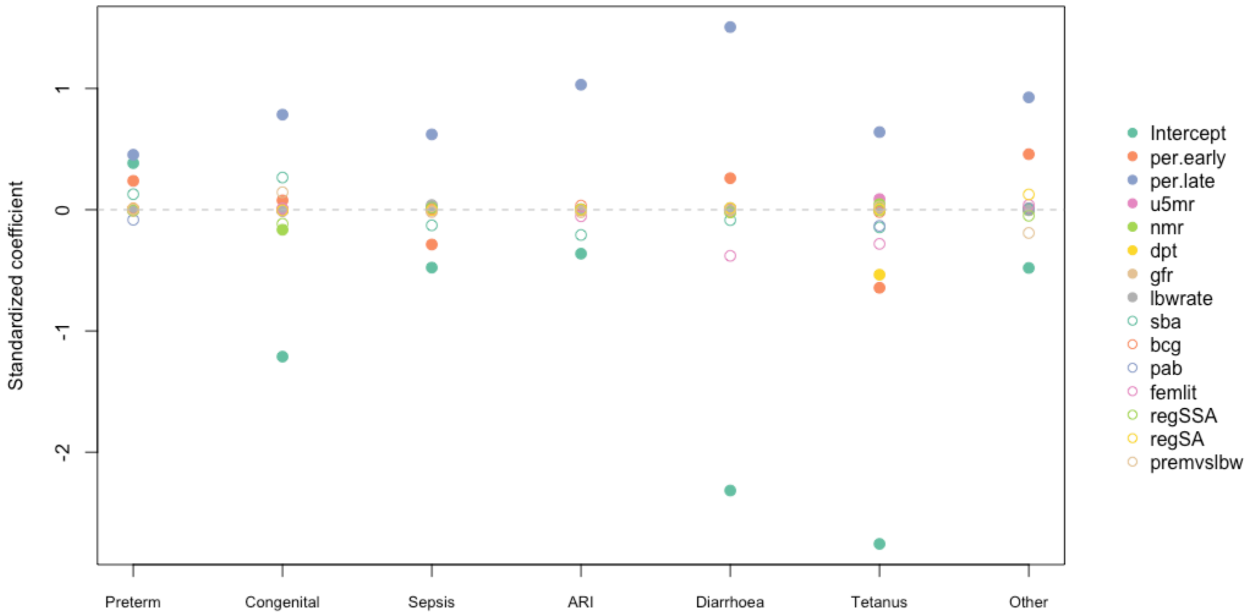

Methods 1-59 month mortality

3. Methods 1-59 months

Models were estimated for the distribution of cause-specific mortality among 1-59 month olds assumed a multinomial likelihood of deaths by cause. Reported causes were translated to causes of interest with the misclassification matrix, allowing for variable definition of “other” or residual causes across studies (for example not all studies report deaths due to deaths due to malaria). Non-informative normal priors were used for multinomial regression coefficients that were not constrained by LASSO parameter λ. Parameter constrained by λ had double exponential priors with mean 0.

**Model; iterations**	**Covariates not constrained by λ**	**Covariates constrained by λ**	**Final λ**	**Priors**
Low mortality; 30000 iterations	Intercept	Indicator of Eastern Mediterranean region, year, log under five mortality rate, log purchasing power parity, percent of population with piped water, percent of population with Hib vaccination, percent of population with skilled birth attendant	5	Beta (unconstrained): Normal(0, precision 4)<br><br>Beta (constrained): Double exponential(0,λ)<br><br>Random effects: Normal(0, precision 1/sd^2)<br><br>Random effect sd: Uniform(0, SD limit = 0.07)
High mortality; 15000 iterations	Intercept, reported for infants and children older than one month (Iagelower_1)	under-five mortality rate (u5mr), plasmodium falciparum prevalence rate (pfpr), whether country/study area was experiencing meningitis outbreak(meningitis_epi), gross national income (gni), measles vaccine coverage (mcv), year (yr_mid), prevalence of underwt among under-fives (underwt), coverage of sanitation (sanitation)	10	Beta (unconstrained): Normal(0, precision 4)<br><br>Beta (constrained): Double exponential(0,λ)<br><br>Random effects: Normal(0, precision 1/sd^2)<br><br>Random effect sd: Uniform(0, SD limit)

3. Methods 1-59 months

Models were estimated for the distribution of cause-specific mortality among 1-59 month olds assumed a multinomial likelihood of deaths by cause. Reported causes were translated to causes of interest with the misclassification matrix, allowing for variable definition of “other” or residual causes across studies (for example not all studies report deaths due to deaths due to malaria). Non-informative normal priors were used for multinomial regression coefficients that were not constrained by LASSO parameter λ. Parameter constrained by λ had double exponential priors with mean 0.

**Model; iterations**	**Covariates not constrained by λ**	**Covariates constrained by λ**	**Final λ**	**Priors**
Low mortality; 30000 iterations	Intercept	Indicator of Eastern Mediterranean region, year, log under five mortality rate, log purchasing power parity, percent of population with piped water, percent of population with Hib vaccination, percent of population with skilled birth attendant	5	Beta (unconstrained): Normal(0, precision 4)<br><br>Beta (constrained): Double exponential(0,λ)<br><br>Random effects: Normal(0, precision 1/sd^2)<br><br>Random effect sd: Uniform(0, SD limit = 0.07)
High mortality; 15000 iterations	Intercept, reported for infants and children older than one month (Iagelower_1)	under-five mortality rate (u5mr), plasmodium falciparum prevalence rate (pfpr), whether country/study area was experiencing meningitis outbreak(meningitis_epi), gross national income (gni), measles vaccine coverage (mcv), year (yr_mid), prevalence of underwt among under-fives (underwt), coverage of sanitation (sanitation)	10	Beta (unconstrained): Normal(0, precision 4)<br><br>Beta (constrained): Double exponential(0,λ)<br><br>Random effects: Normal(0, precision 1/sd^2)<br><br>Random effect sd: Uniform(0, SD limit)

3.2 1-59 month high mortality model

Cross validation error to select Lambda for 1-59 month high mortality model – Lambda 10

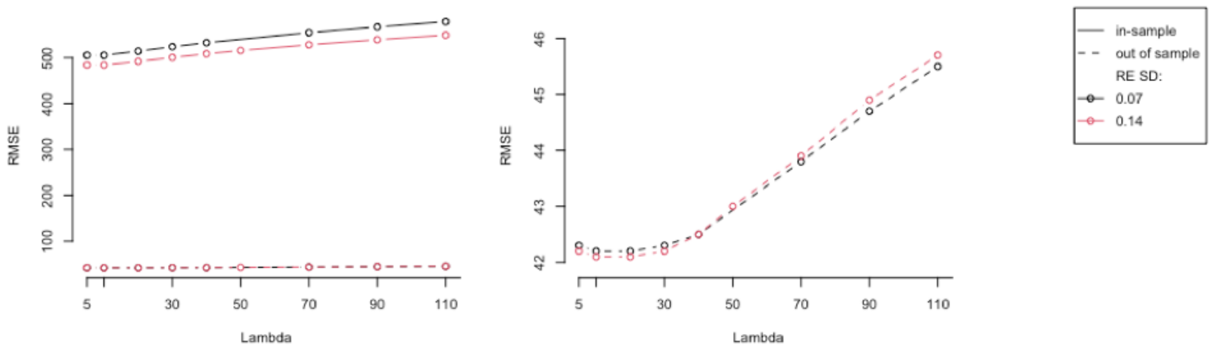

Coefficient estimates for 1-59 month high mortality model (reference ARI)

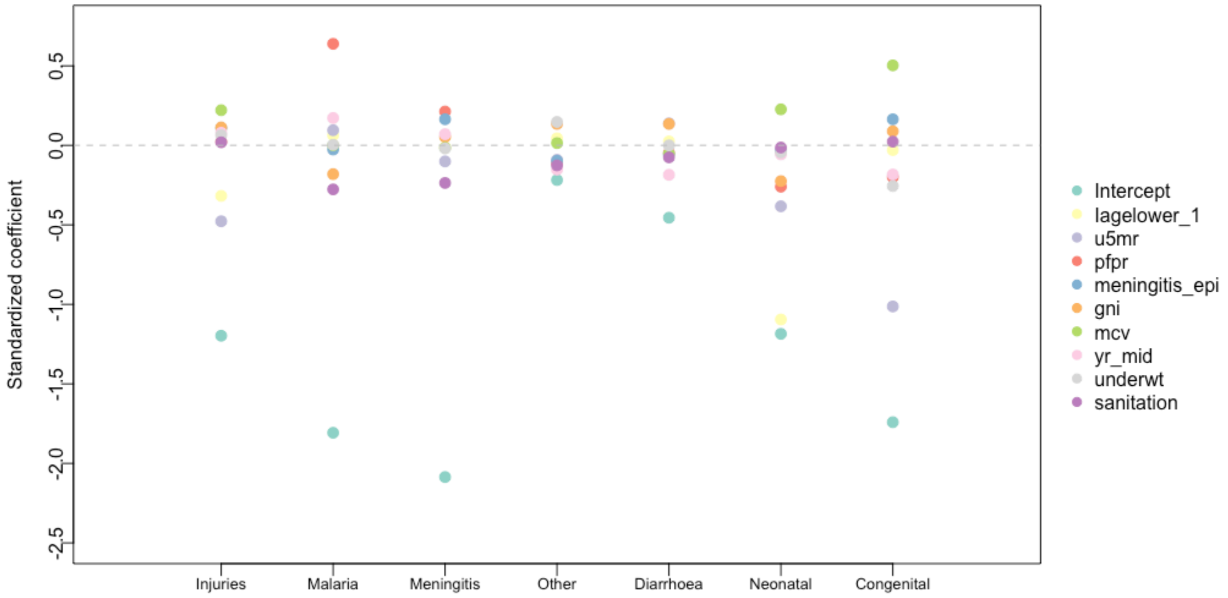

Appendix 4: Weight of random effects in nationally represetative studies

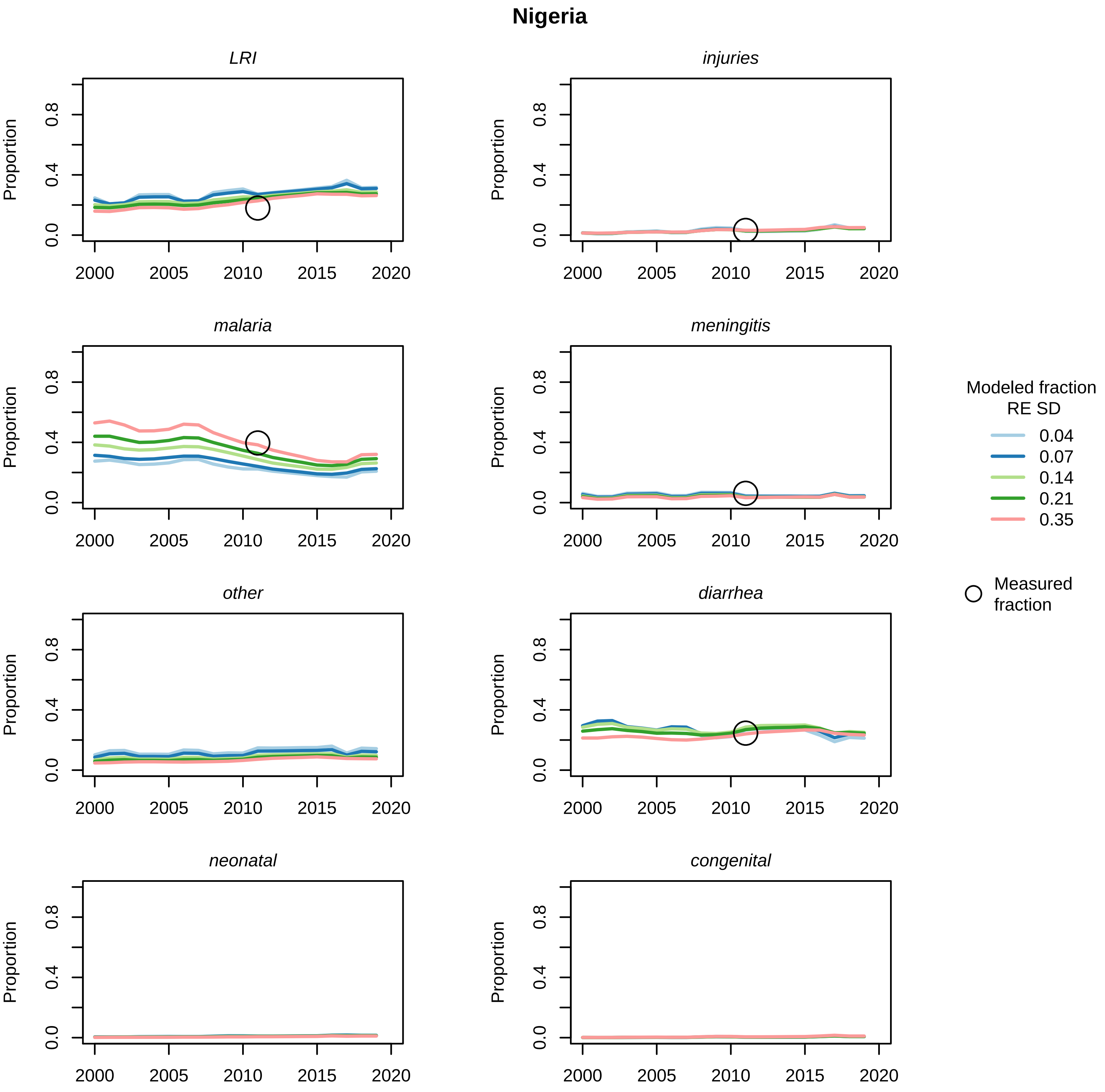
Nigeria
LRI
injuries
malaria
meningitis
other
diarrhea
neonatal
congenital
Proportion
0.0
0.4
0.8
2000
2005
2010
2015
2020
Modeled fraction
RE SD
0.04
0.07
0.14
0.21
0.35
Measured fraction

Pakistan

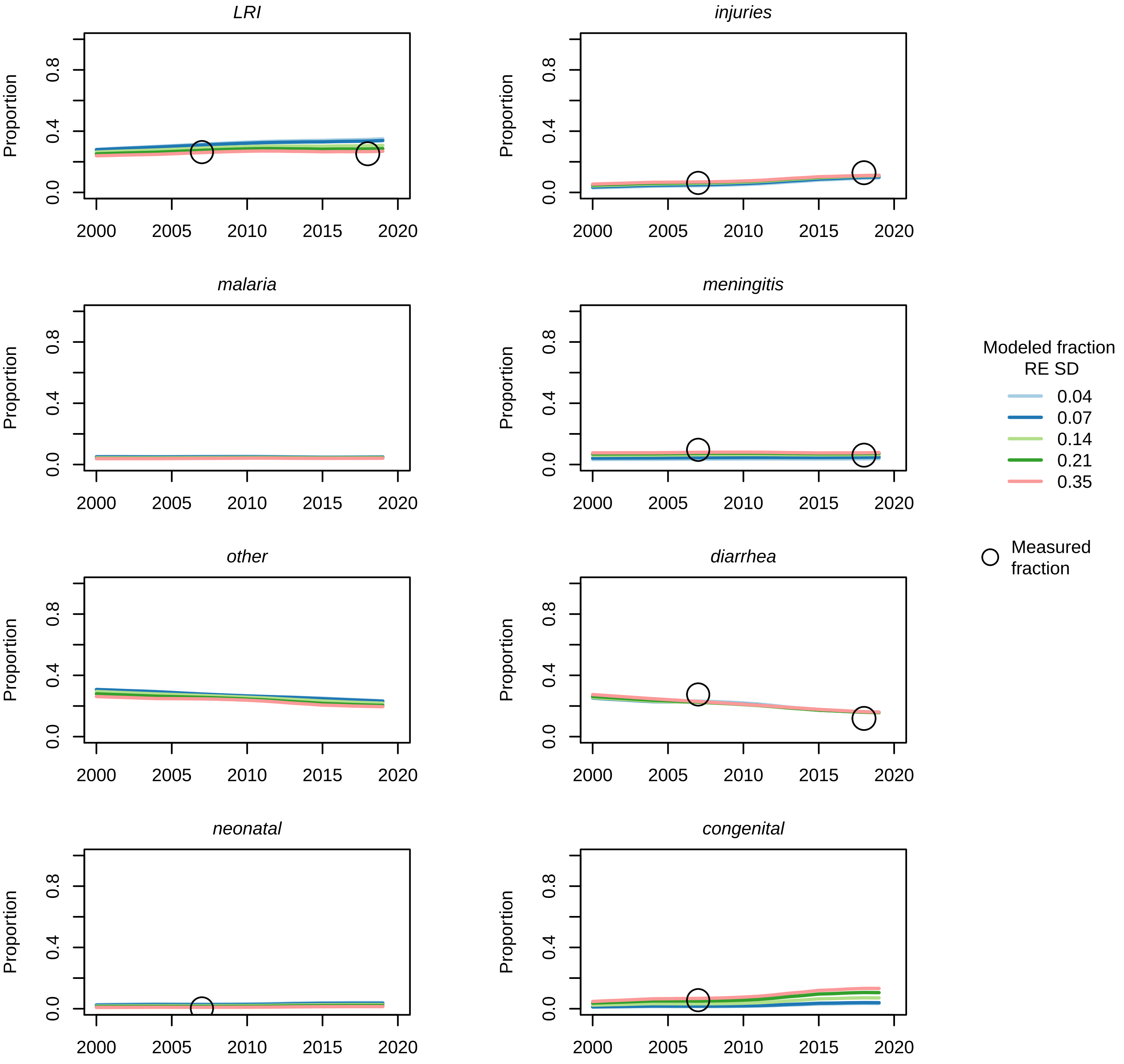
LRI
injuries
malaria
meningitis
other
diarrhea
neonatal
congenital
Proportion
0.0
0.4
0.8
2000
2005
2010
2015
2020
Modeled fraction
RE SD
0.04
0.07
0.14
0.21
0.35
Measured fraction

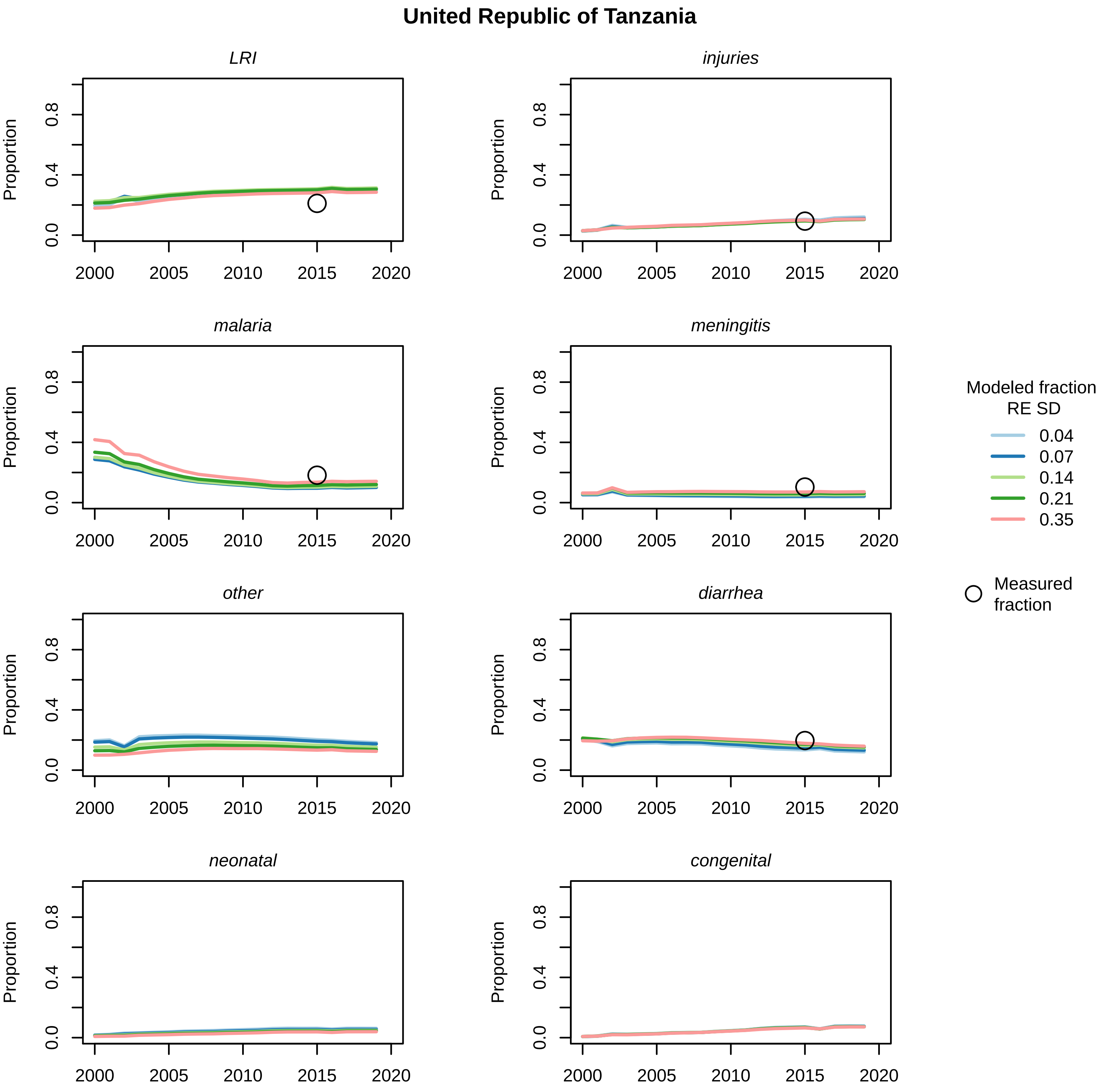

United Republic of Tanzania
LRI
injuries
malaria
meningitis
other
diarrhea
neonatal
congenital
Proportion
0.0
0.4
0.8
2000
2005
2010
2015
2020
Modeled fraction
RE SD
0.04
0.07
0.14
0.21
0.35
Measured fraction

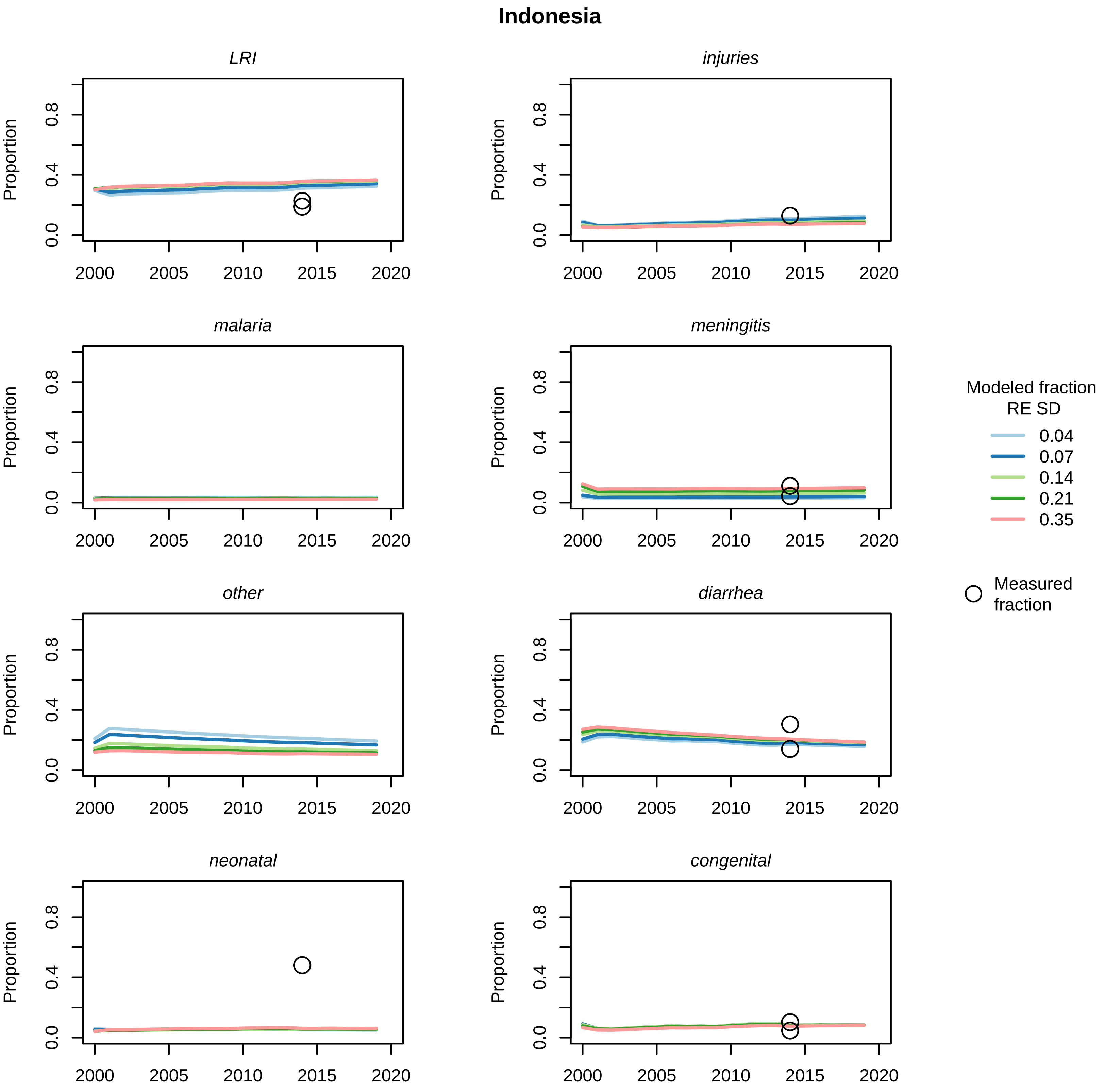

Indonesia
LRI
injuries
malaria
meningitis
other
diarrhea
neonatal
congenital
Proportion
0.0
0.4
0.8
2000
2005
2010
2015
2020
Modeled fraction RE SD
0.04
0.07
0.14
0.21
0.35
Measured fraction

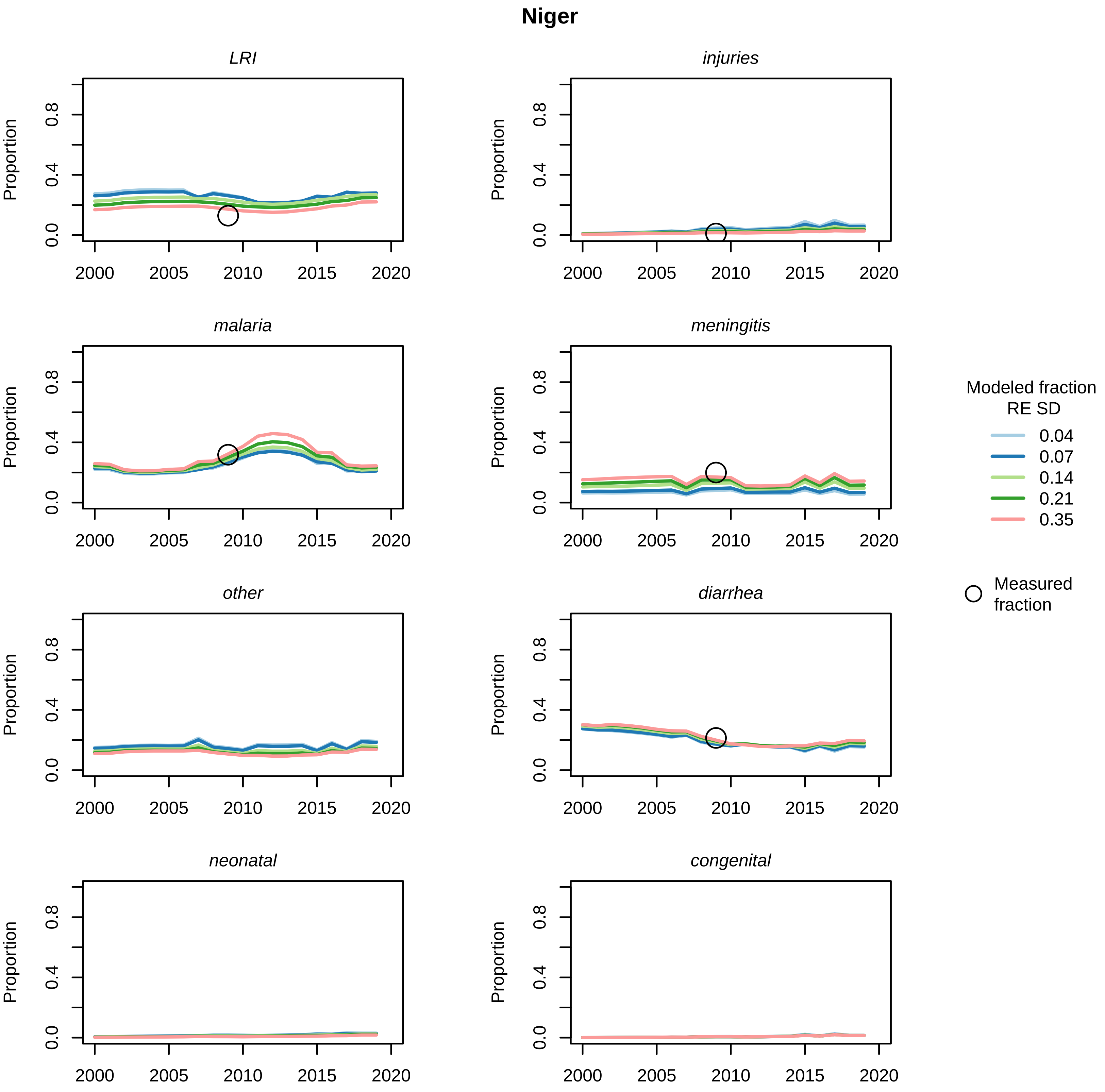

Niger
LRI
injuries
malaria
meningitis
other
diarrhea
neonatal
congenital
Proportion
0.0
0.4
0.8
2000
2005
2010
2015
2020
Modeled fraction
RE SD
0.04
0.07
0.14
0.21
0.35
Measured
fraction

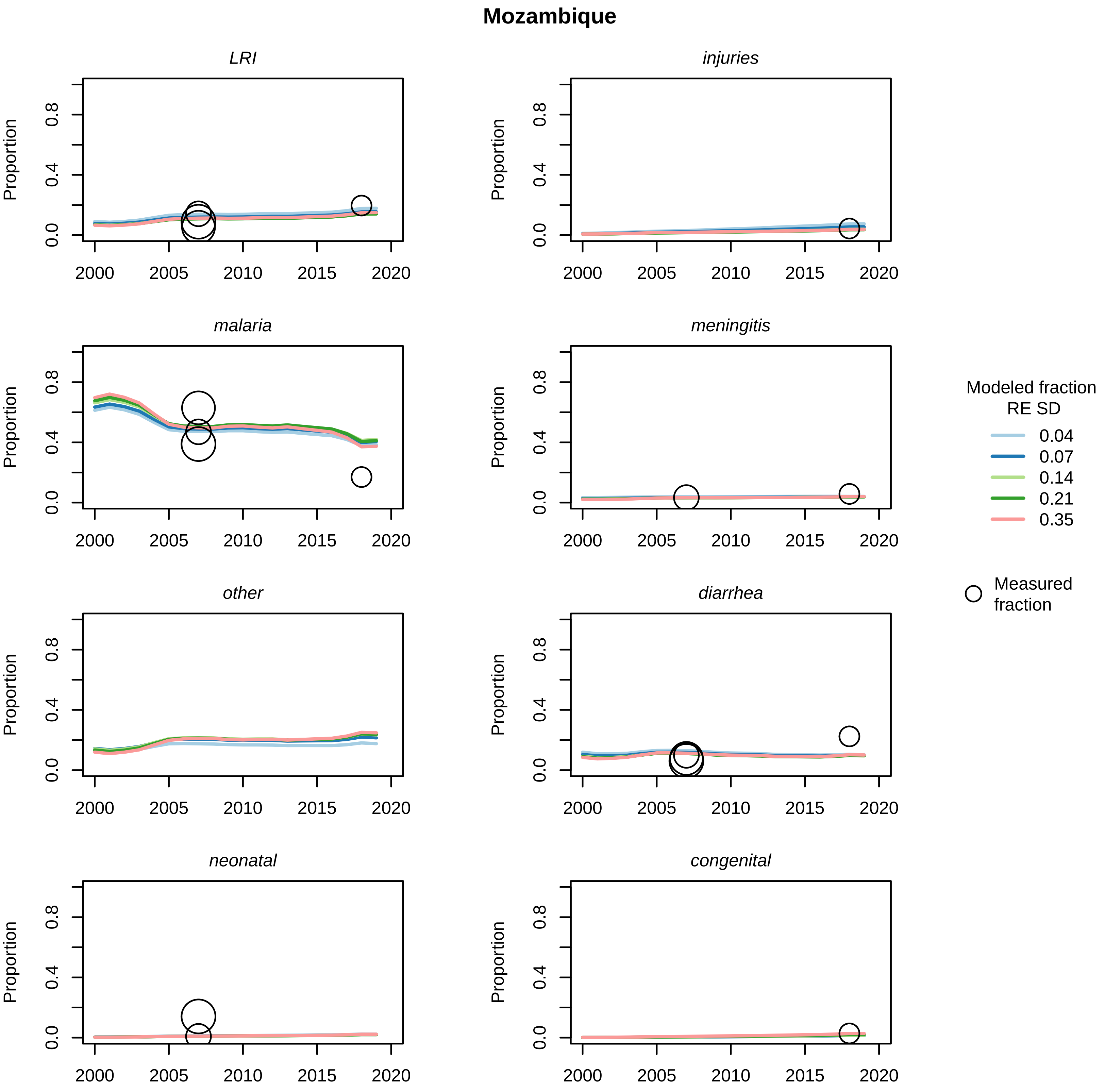
Mozambique
LRI
injuries
malaria
meningitis
other
diarrhea
neonatal
congenital
Proportion
0.0
0.4
0.8
2000
2005
2010
2015
2020
Modeled fraction
RE SD
0.04
0.07
0.14
0.21
0.35
Measured fraction

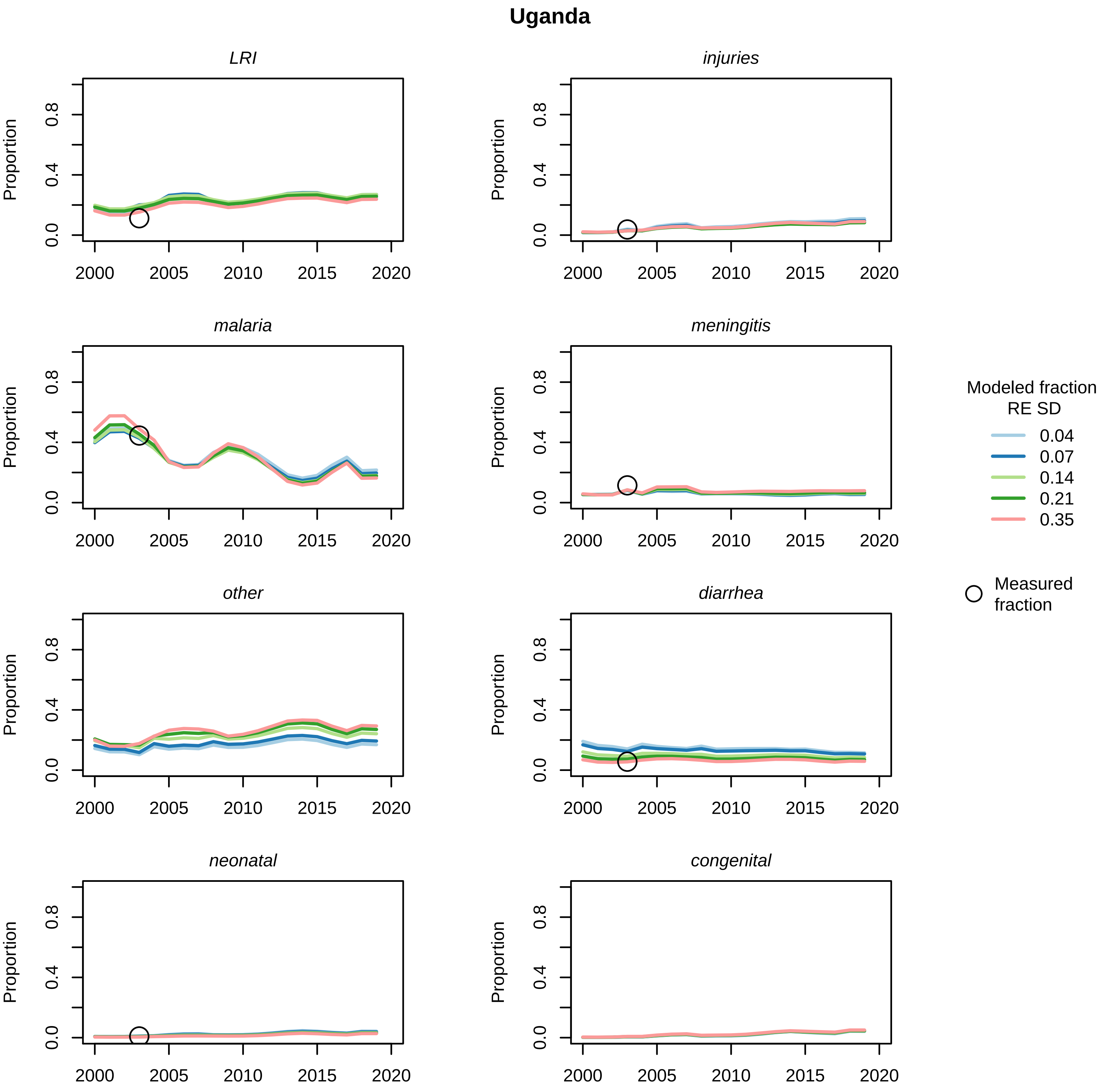

Uganda
LRI
injuries
malaria
meningitis
other
diarrhea
neonatal
congenital
Proportion
0.0
0.4
0.8
2000
2005
2010
2015
2020
Modeled fraction
RE SD
0.04
0.07
0.14
0.21
0.35
Measured
fraction

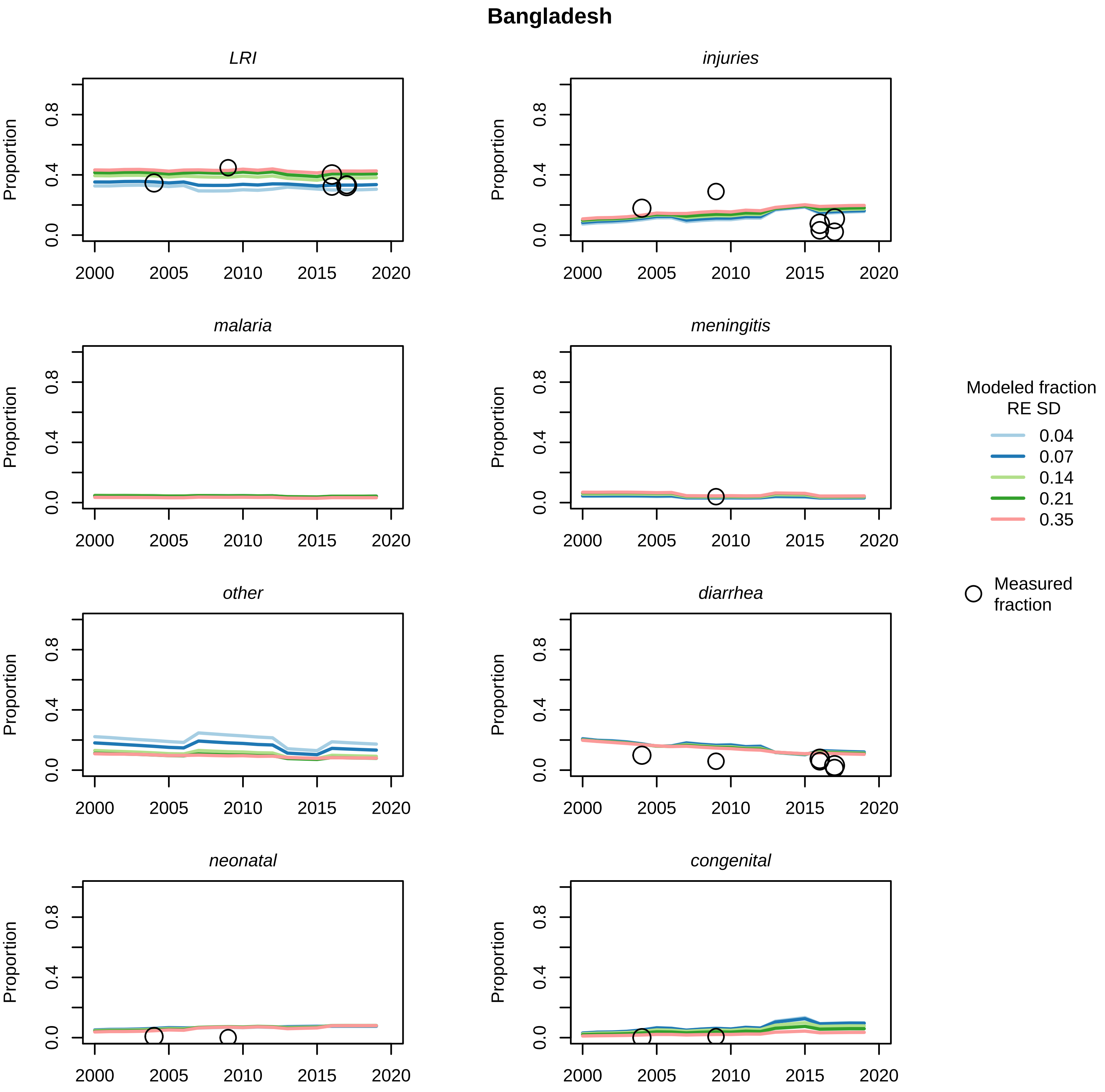
Bangladesh
LRI
injuries
malaria
meningitis
other
diarrhea
neonatal
congenital
Proportion
0.0
0.4
0.8
2000
2005
2010
2015
2020
Modeled fraction
RE SD
0.04
0.07
0.14
0.21
0.35
Measured
fraction

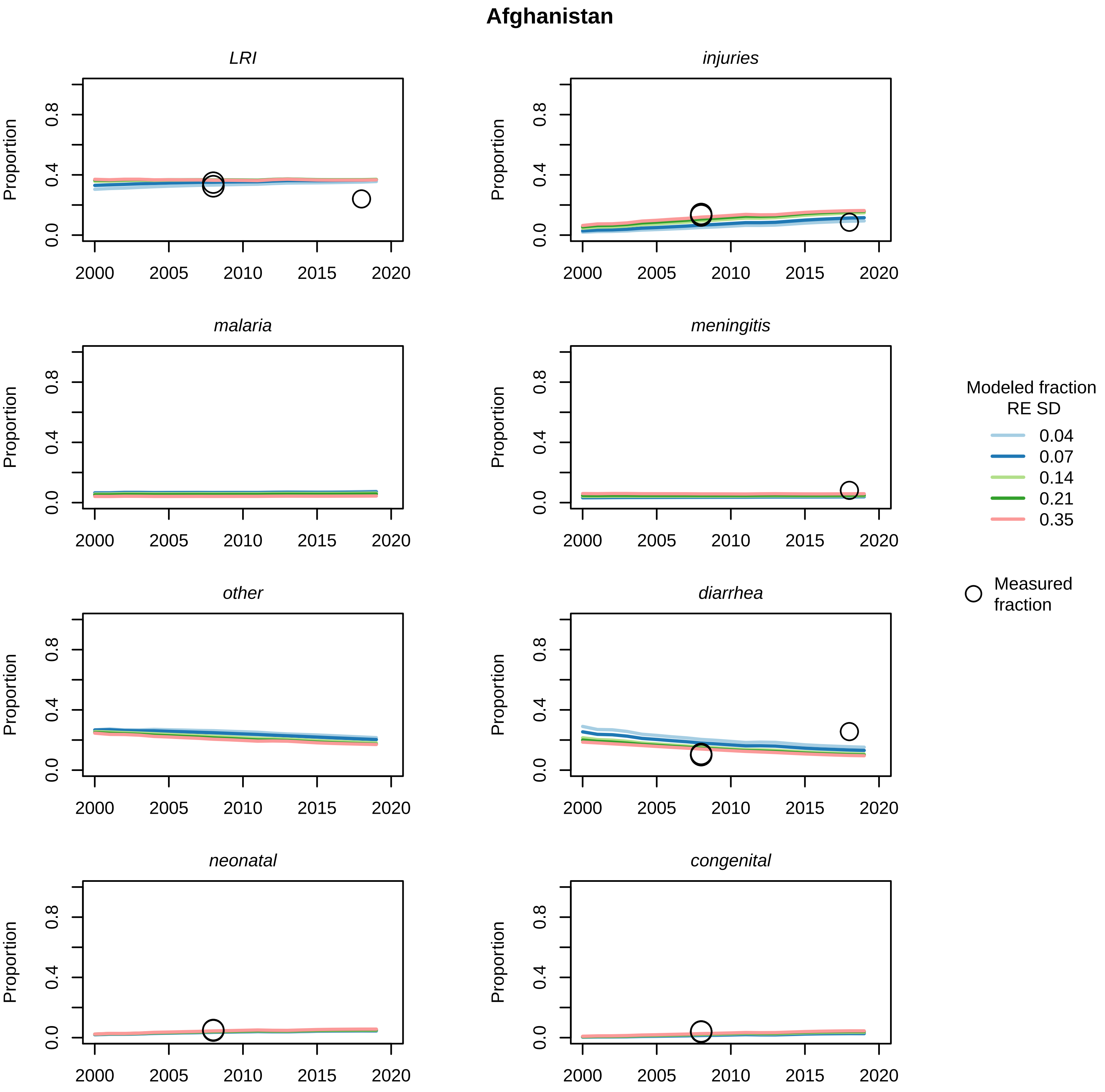
Afghanistan
LRI
injuries
malaria
meningitis
other
diarrhea
neonatal
congenital
Proportion
0.0
0.4
0.8
2000
2005
2010
2015
2020
Modeled fraction
RE SD
0.04
0.07
0.14
0.21
0.35
Measured fraction

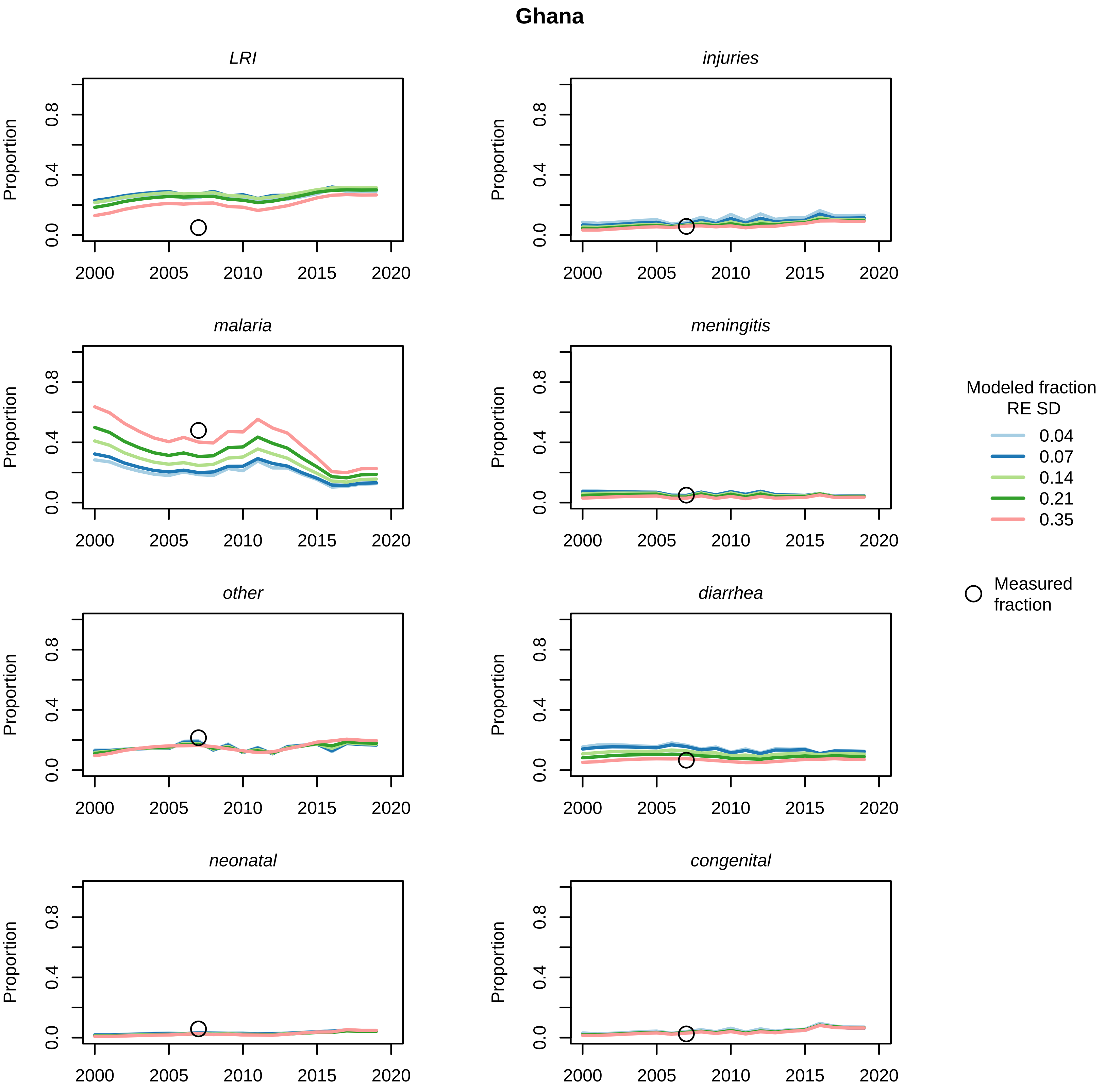
Ghana
LRI
injuries
malaria
meningitis
other
diarrhea
neonatal
congenital
Proportion
0.0
0.4
0.8
2000
2005
2010
2015
2020
Modeled fraction
RE SD
0.04
0.07
0.14
0.21
0.35
Measured
fraction

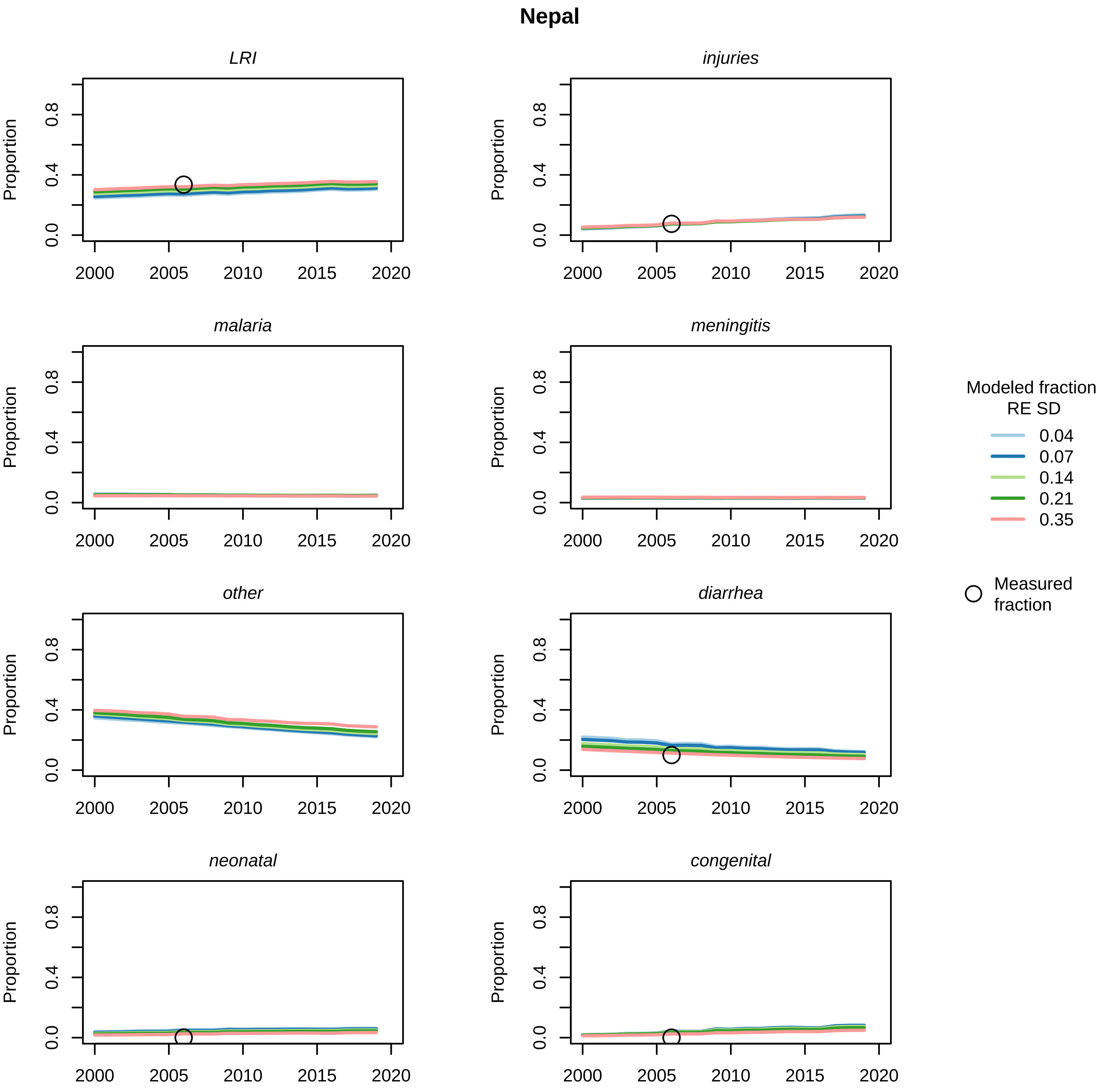
Nepal
LRI
injuries
malaria
meningitis
other
diarrhea
neonatal
congenital
Proportion
0.0
0.4
0.8
2000
2005
2010
2015
2020
Modeled fraction
RE SD
0.04
0.07
0.14
0.21
0.35
Measured fraction

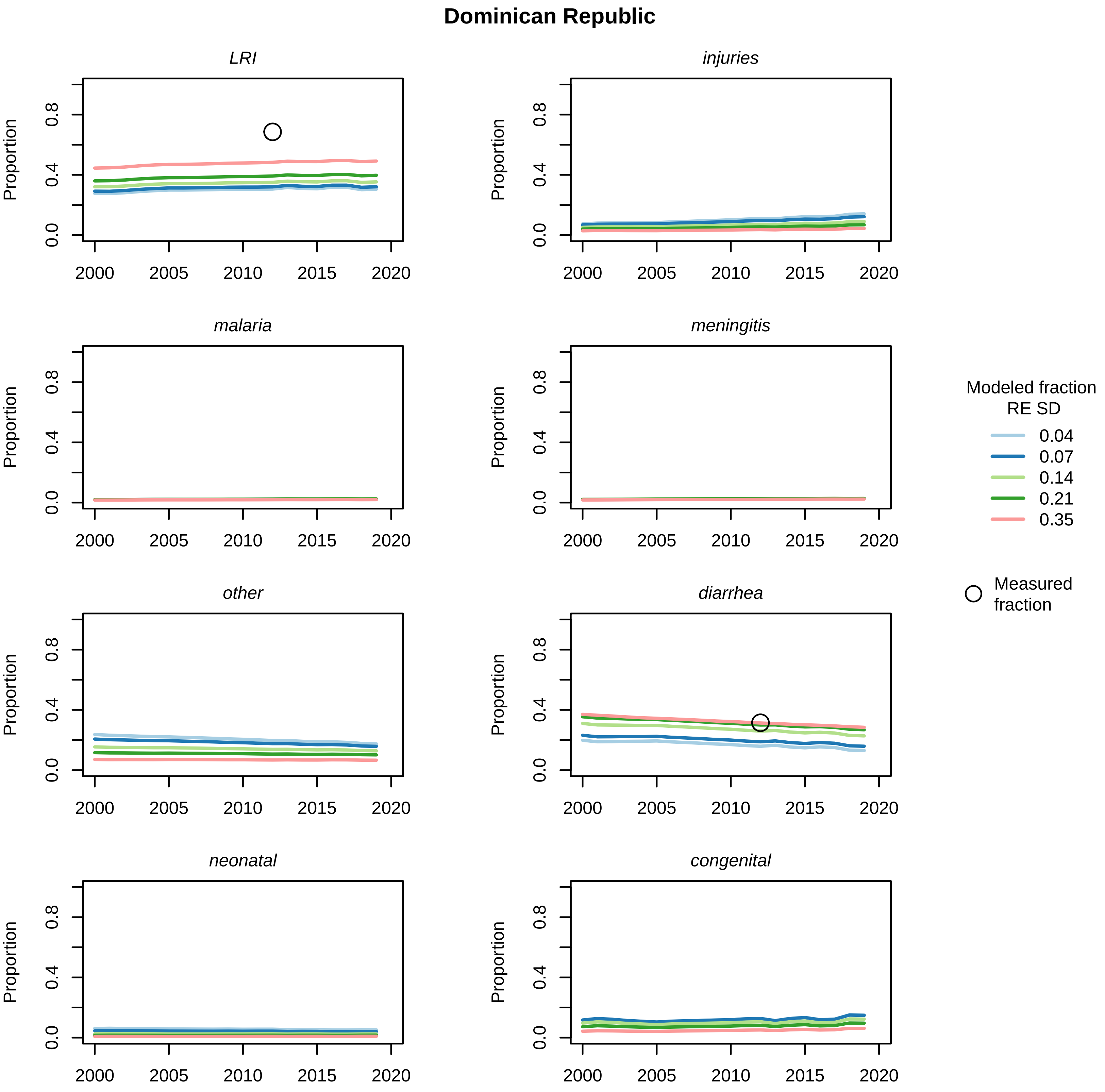

Dominican Republic
LRI
injuries
malaria
meningitis
other
diarrhea
neonatal
congenital
Proportion
0.0
0.4
0.8
2000
2005
2010
2015
2020
Modeled fraction
RE SD
0.04
0.07
0.14
0.21
0.35
Measured fraction

Appendix 5: Convergence of fixed effects (trace plots)

Early low mortality model for neonates, cause: intrapartum

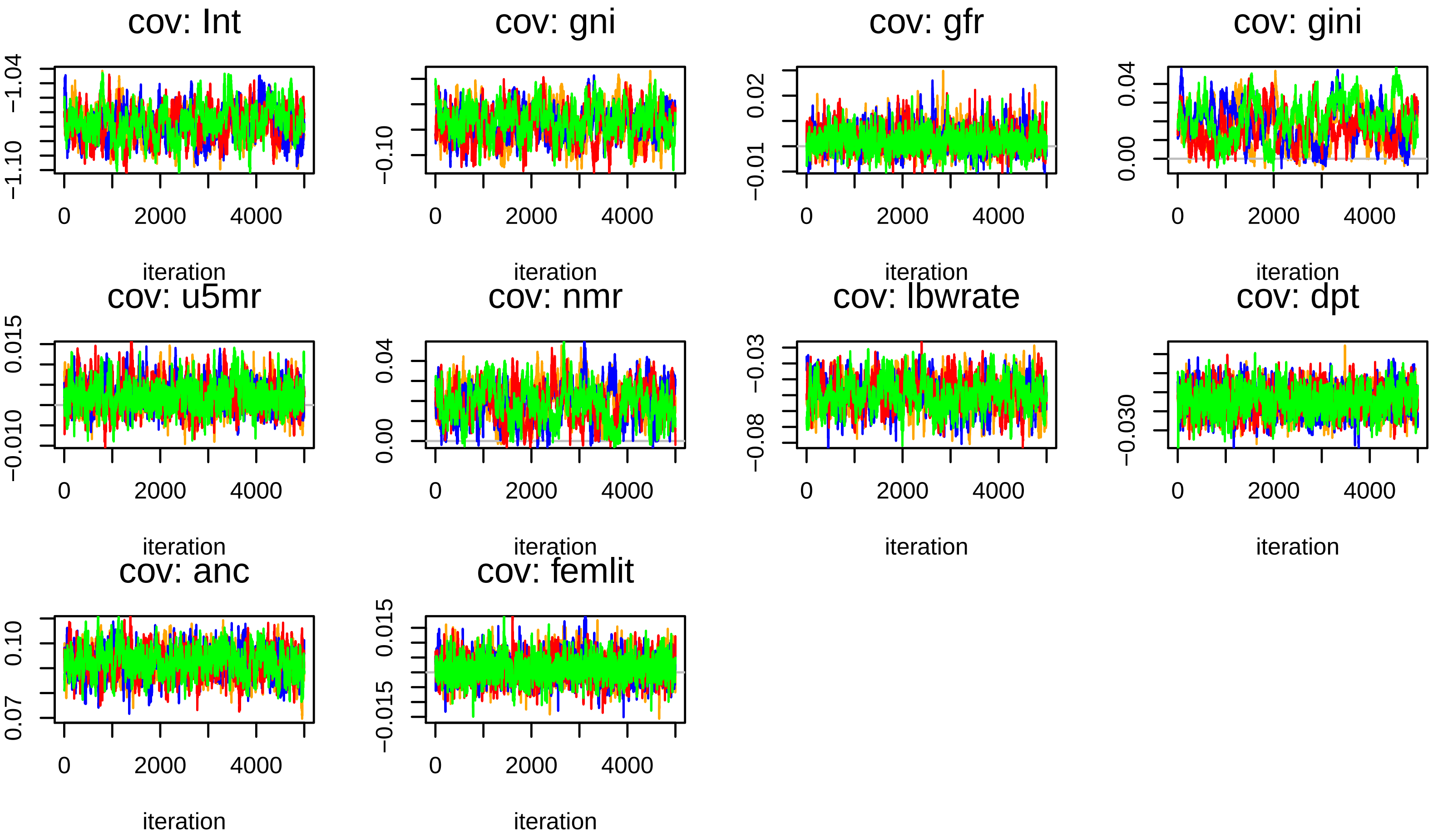

Early low mortality model for neonates, cause: congenital

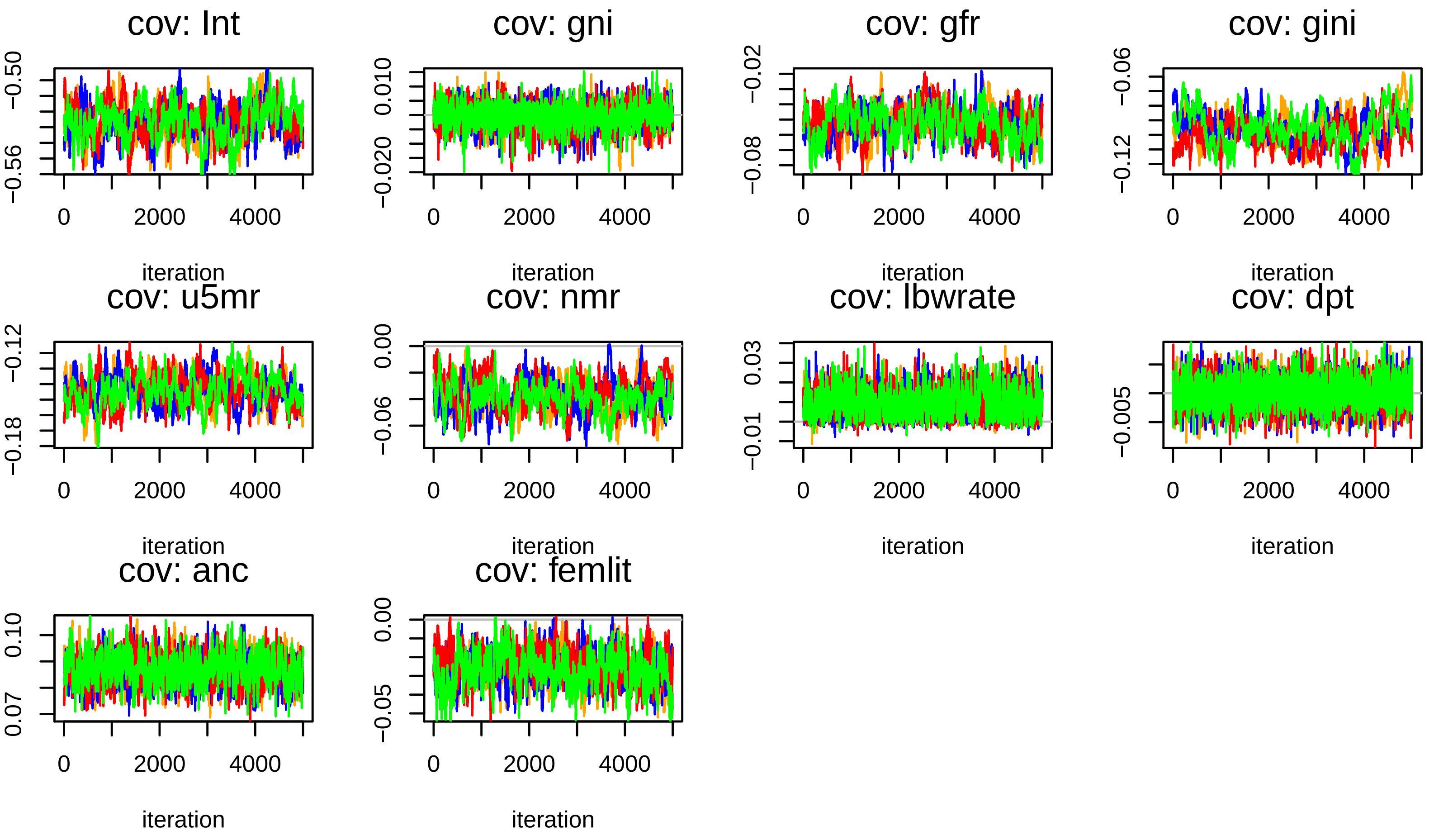

Early low mortality model for neonates, cause: sepsis

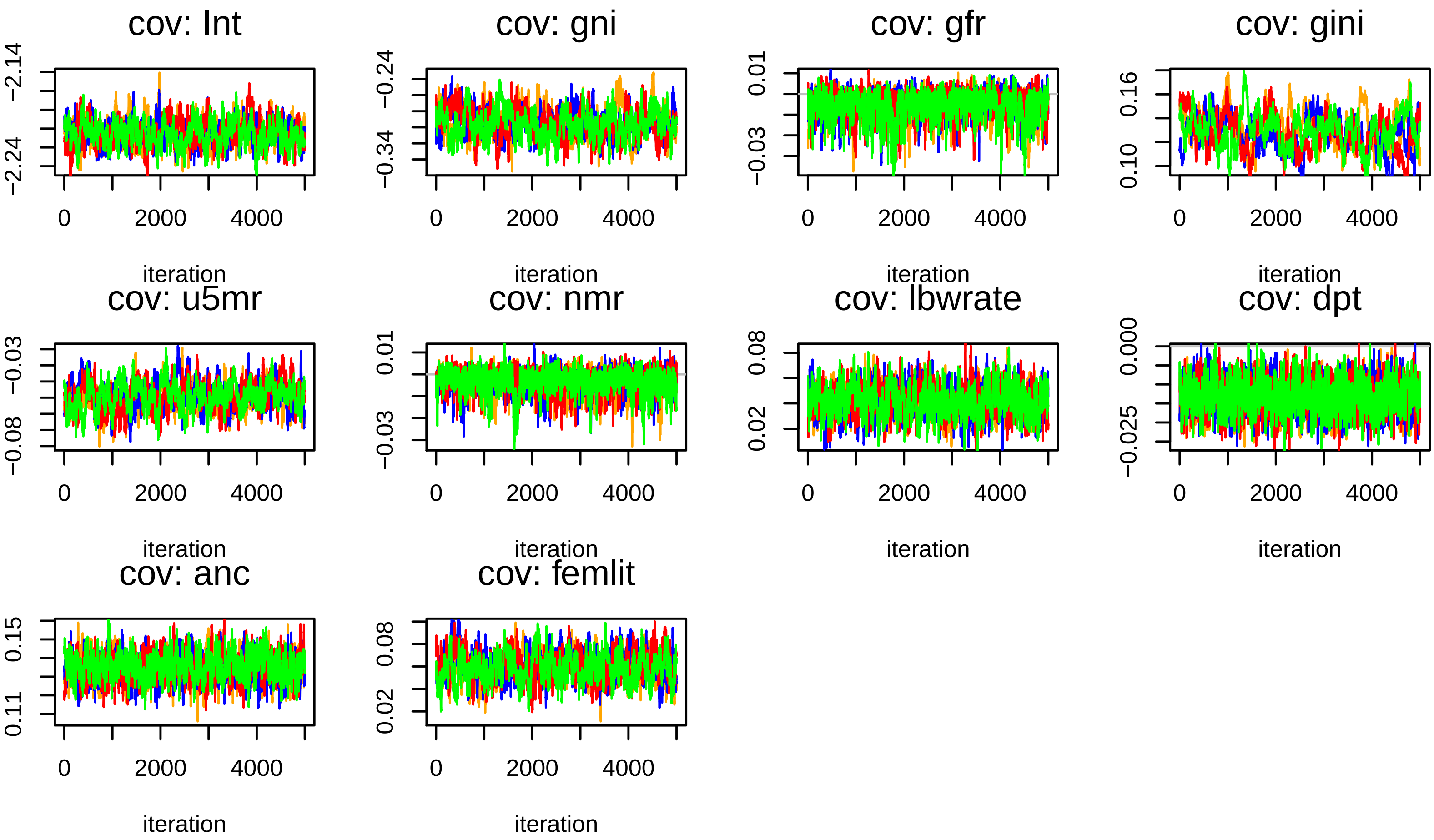

Early low mortality model for neonates, cause: pneumonia

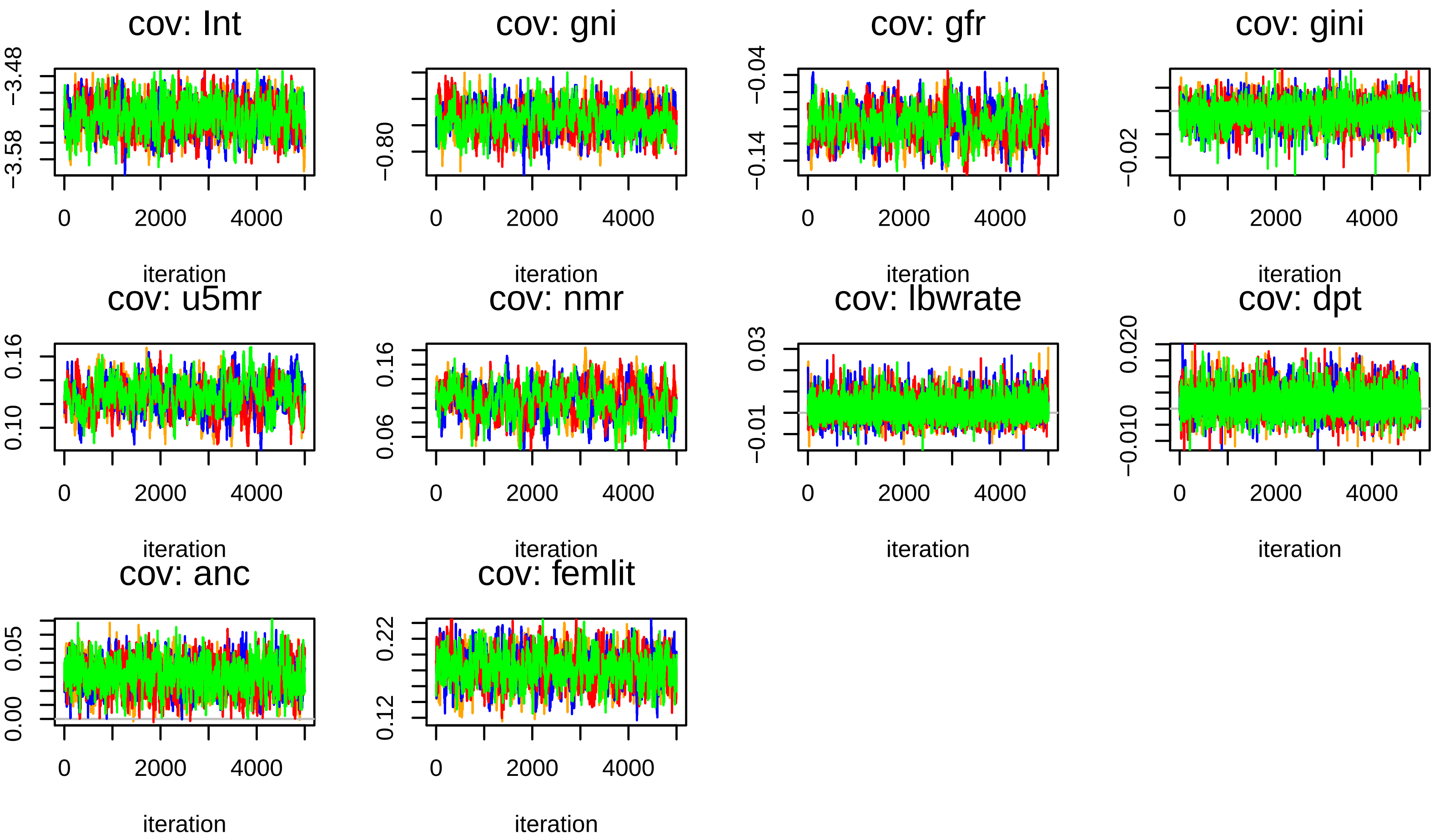

Early low mortality model for neonates, cause: injuries

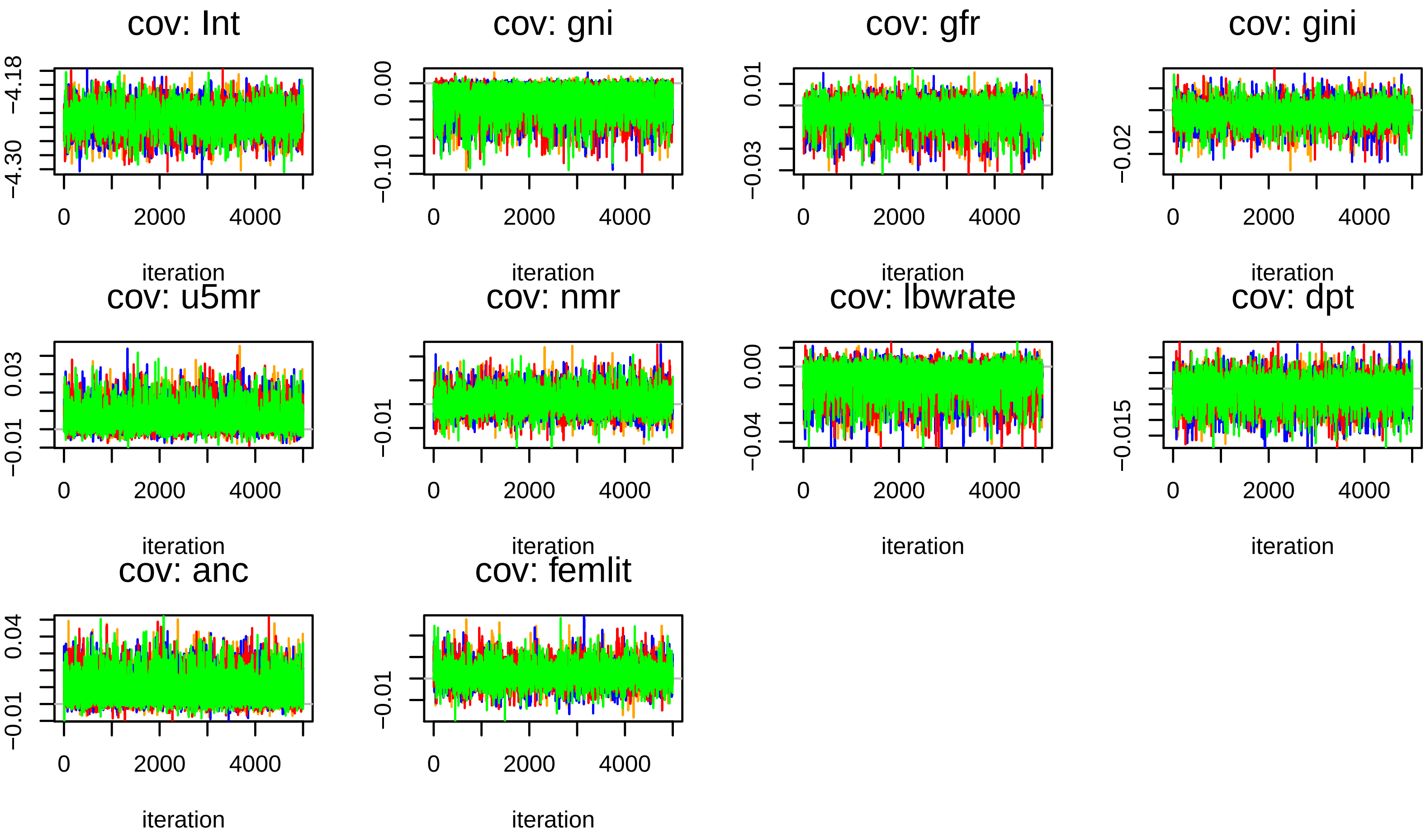

Early low mortality model for neonates, cause: other

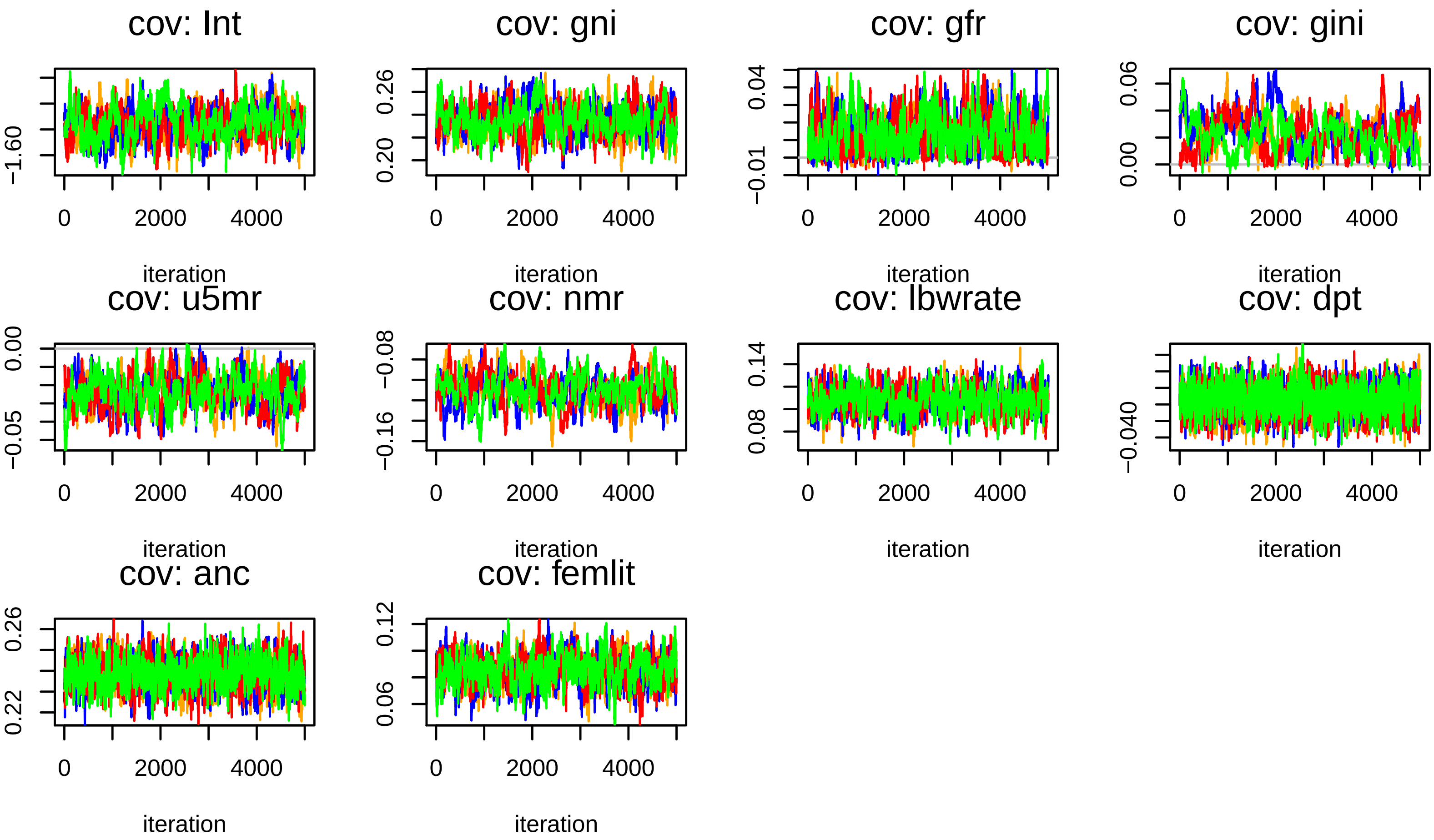

Late low mortality model for neonates, cause: intrapartum

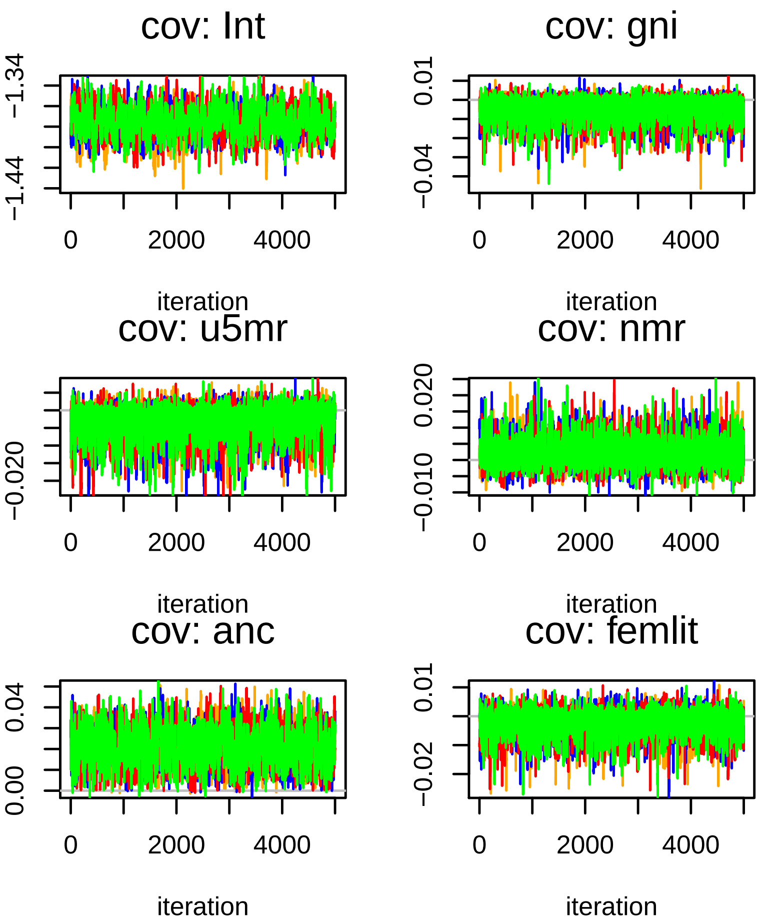

Late low mortality model for neonates, cause: congenital

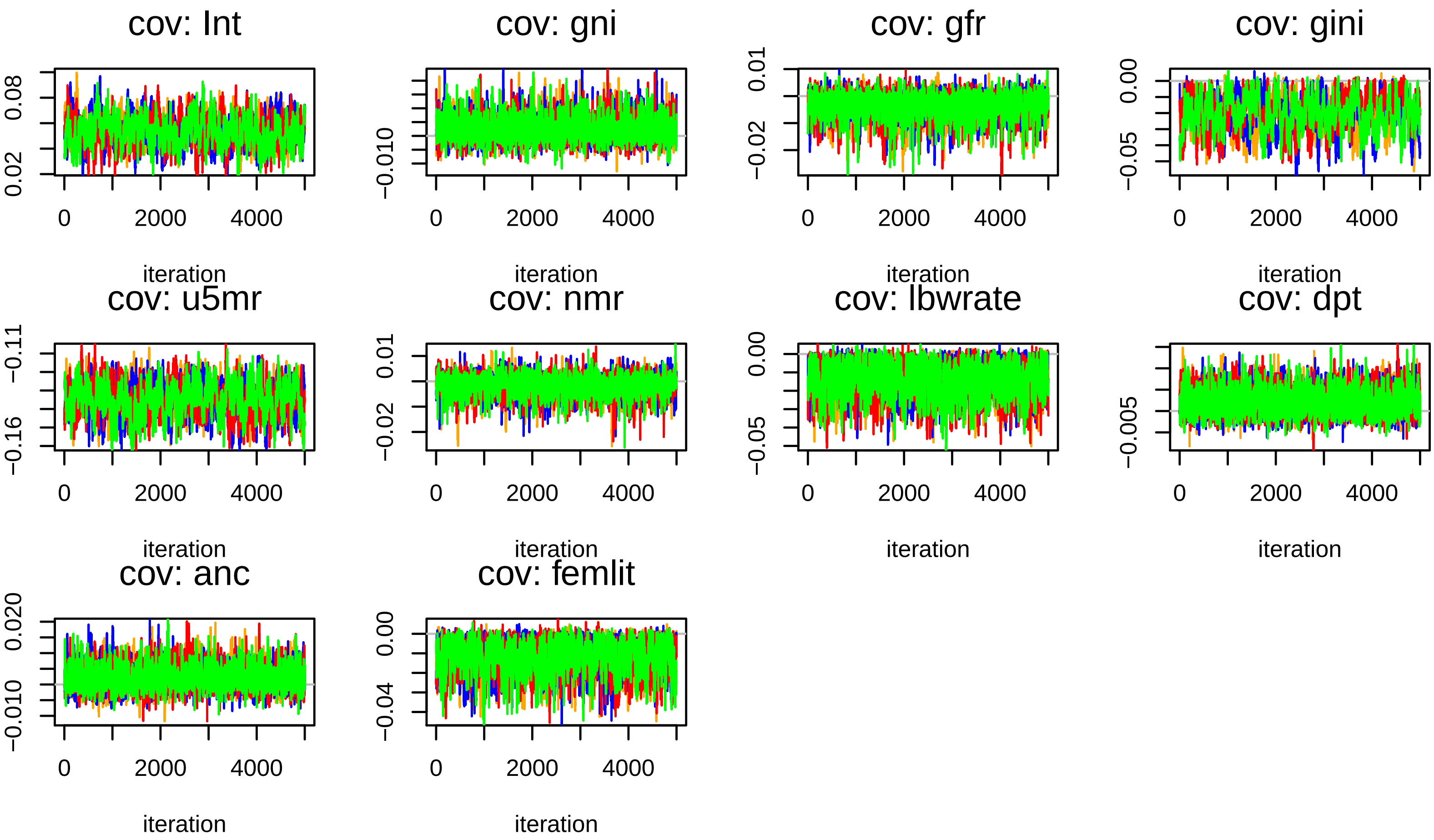

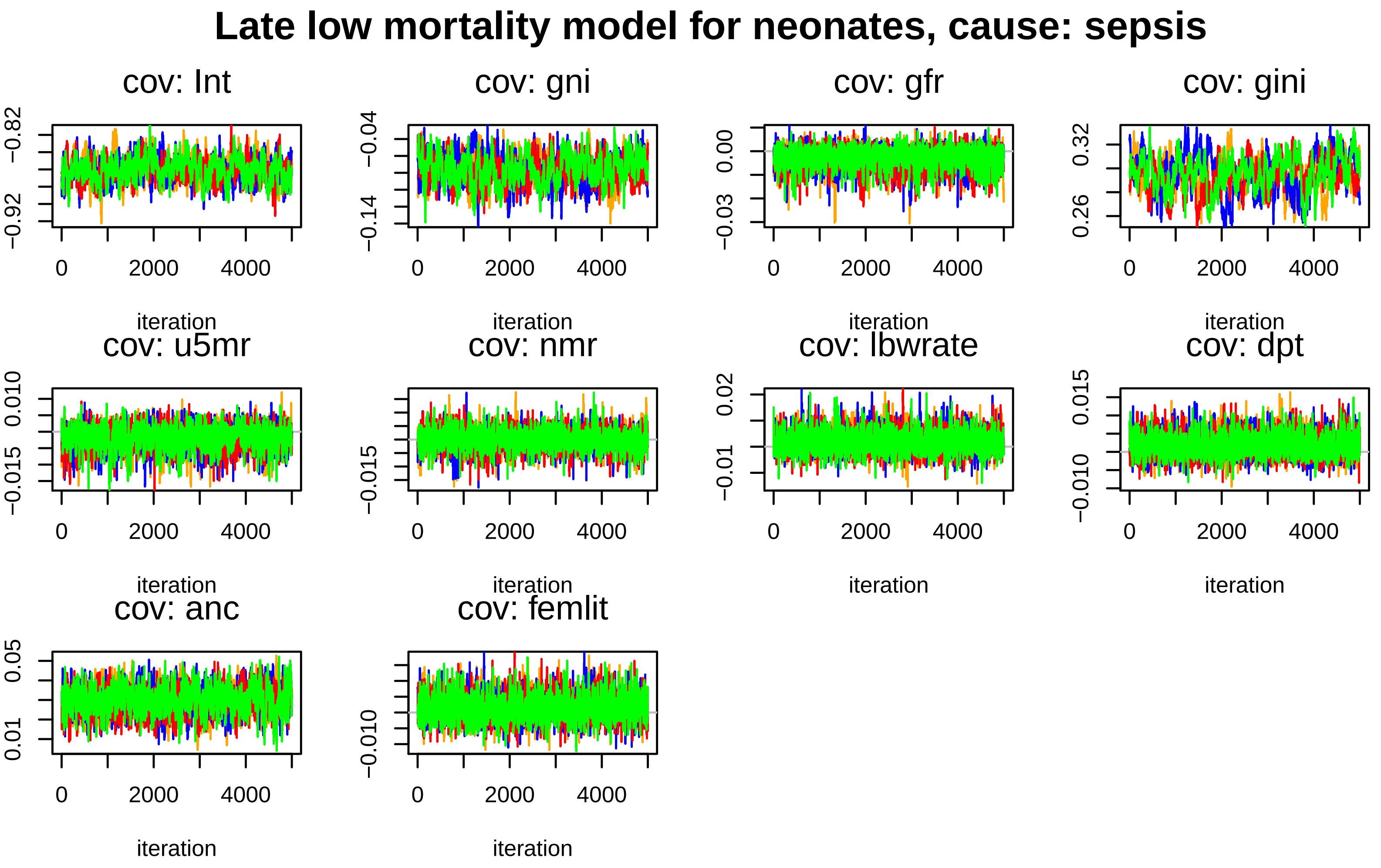

Late low mortality model for neonates, cause: sepsis
cov: Int
cov: gni
cov: gfr
cov: gini
cov: u5mr
cov: nmr
cov: lbwrate
cov: dpt
cov: anc
cov: femlit
iteration
0
2000
4000
−0.82
−0.92
−0.04
−0.14
0.00
−0.03
0.32
0.26
0.010
−0.015
0.02
−0.01
0.015
−0.010
0.05
0.01

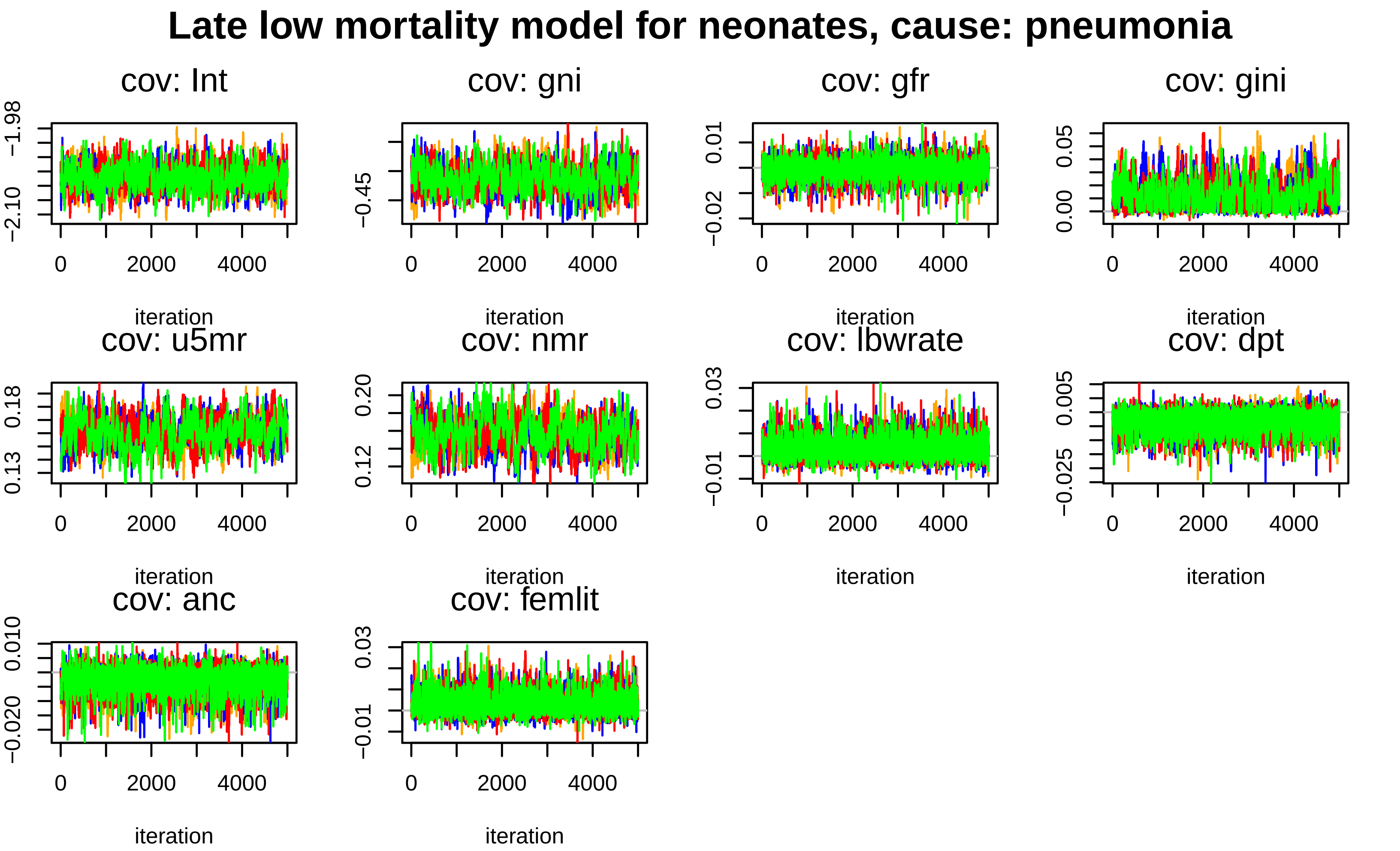
Late low mortality model for neonates, cause: pneumonia
cov: Int
cov: gni
cov: gfr
cov: gini
cov: u5mr
cov: nmr
cov: lbwrate
cov: dpt
cov: anc
cov: femlit
iteration
0
2000
4000
−1.98
−2.10
−0.45
0.01
−0.02
0.05
0.00
0.18
0.13
0.20
0.12
0.03
−0.01
0.005
−0.025
0.010
−0.020

Late low mortality model for neonates, cause: injuries

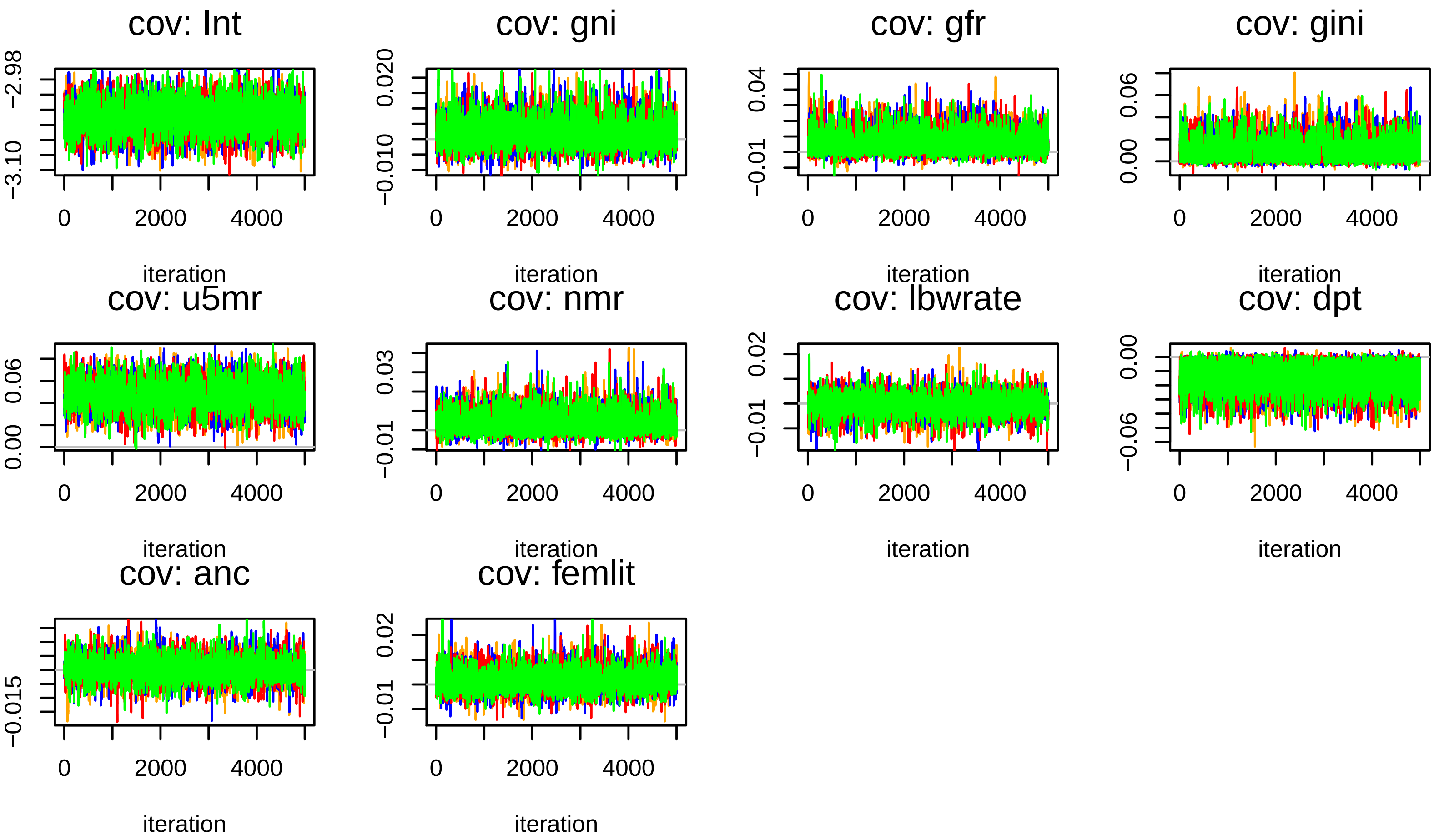

Late low mortality model for neonates, cause: other

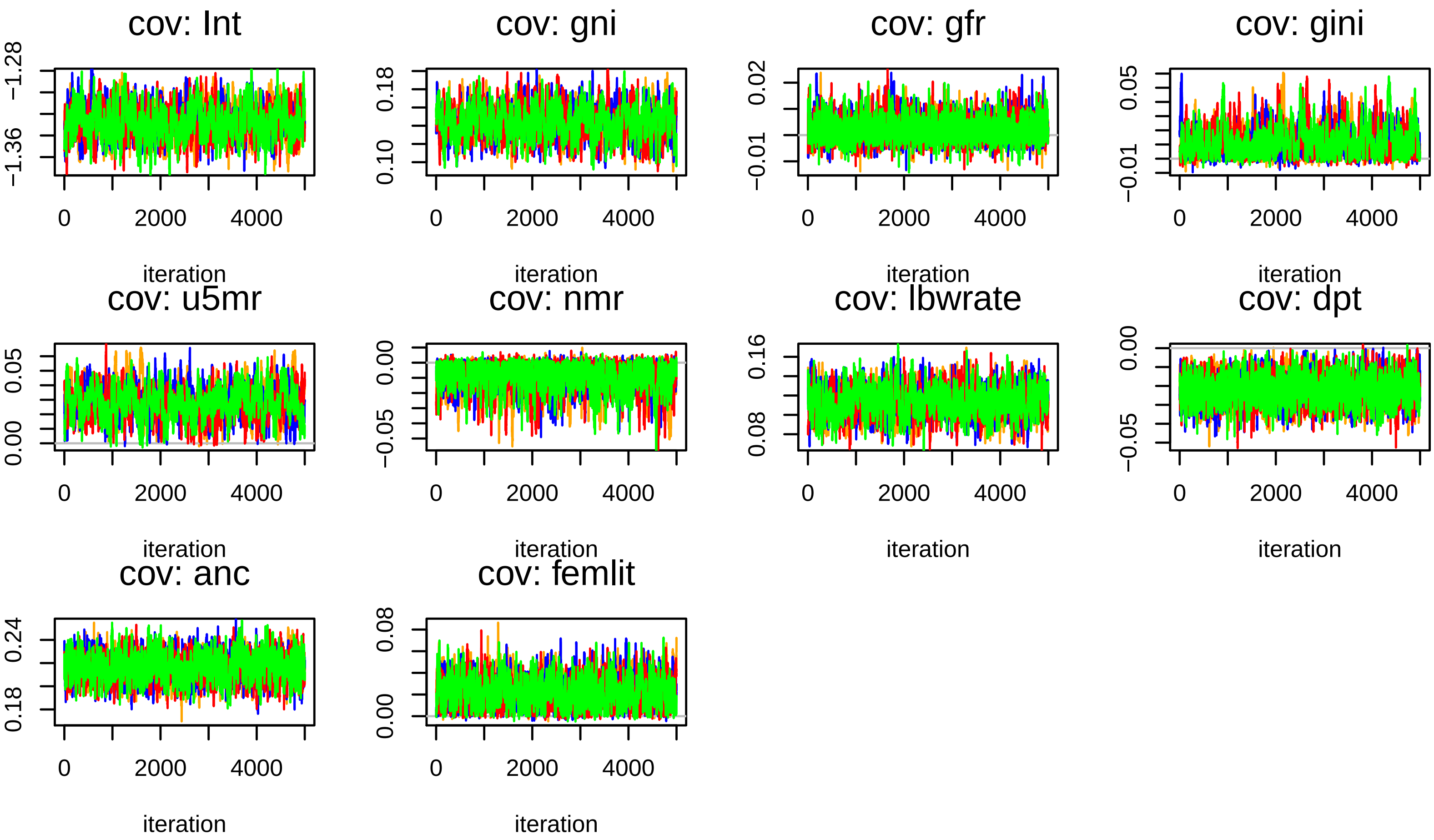

High mortality model for neonates, cause: preterm

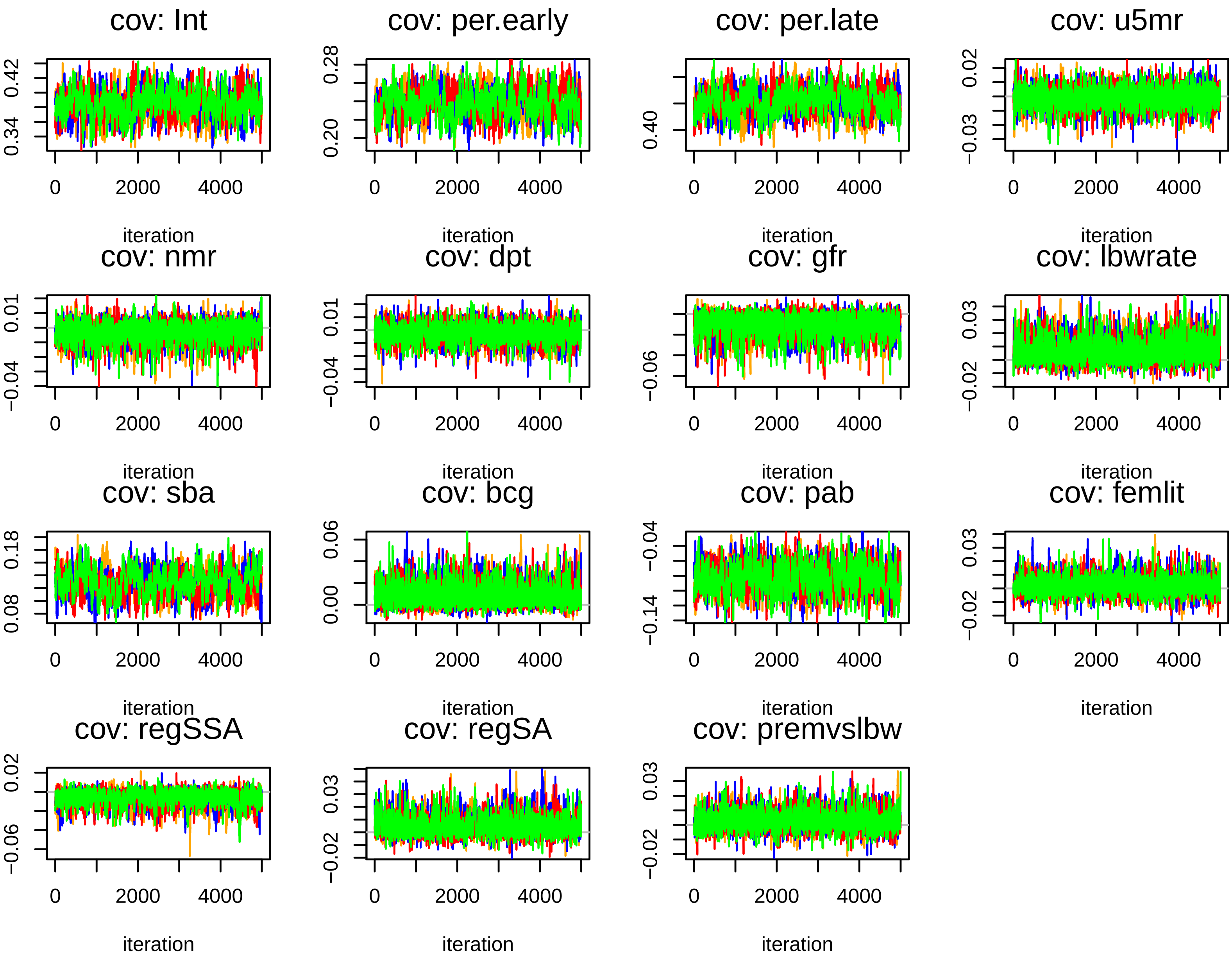

High mortality model for neonates, cause: congenital

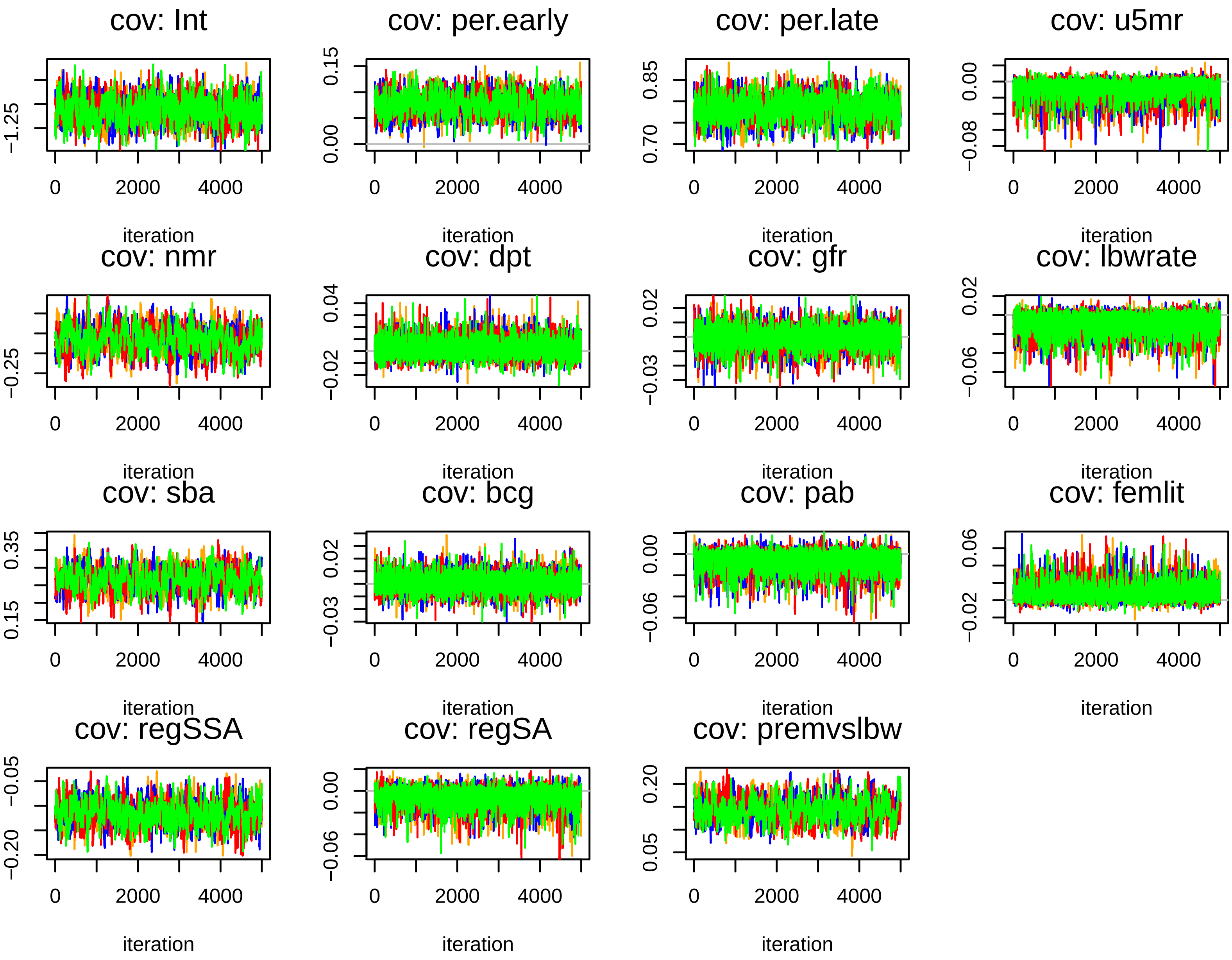

High mortality model for neonates, cause: sepsis

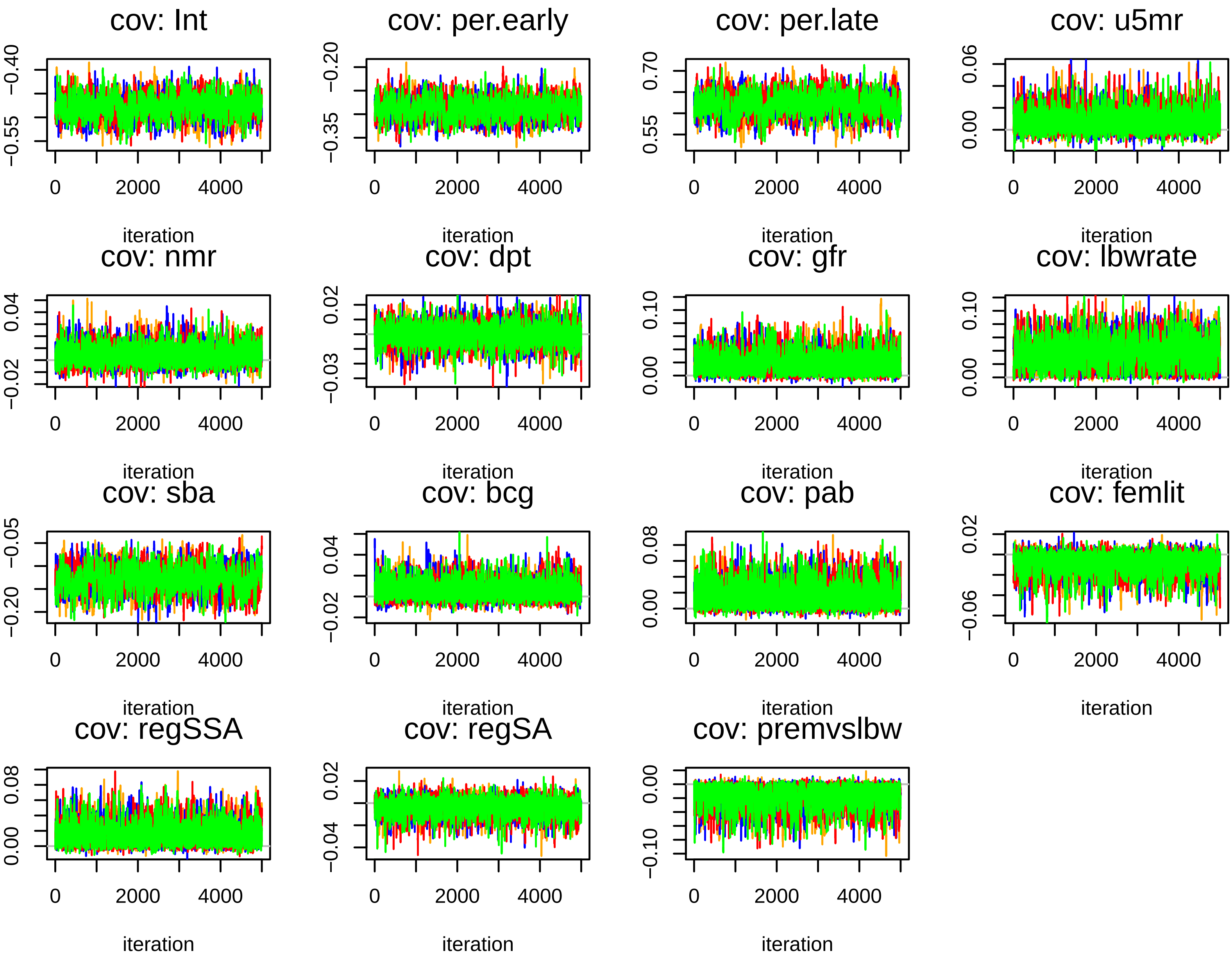

High mortality model for neonates, cause: pneumonia

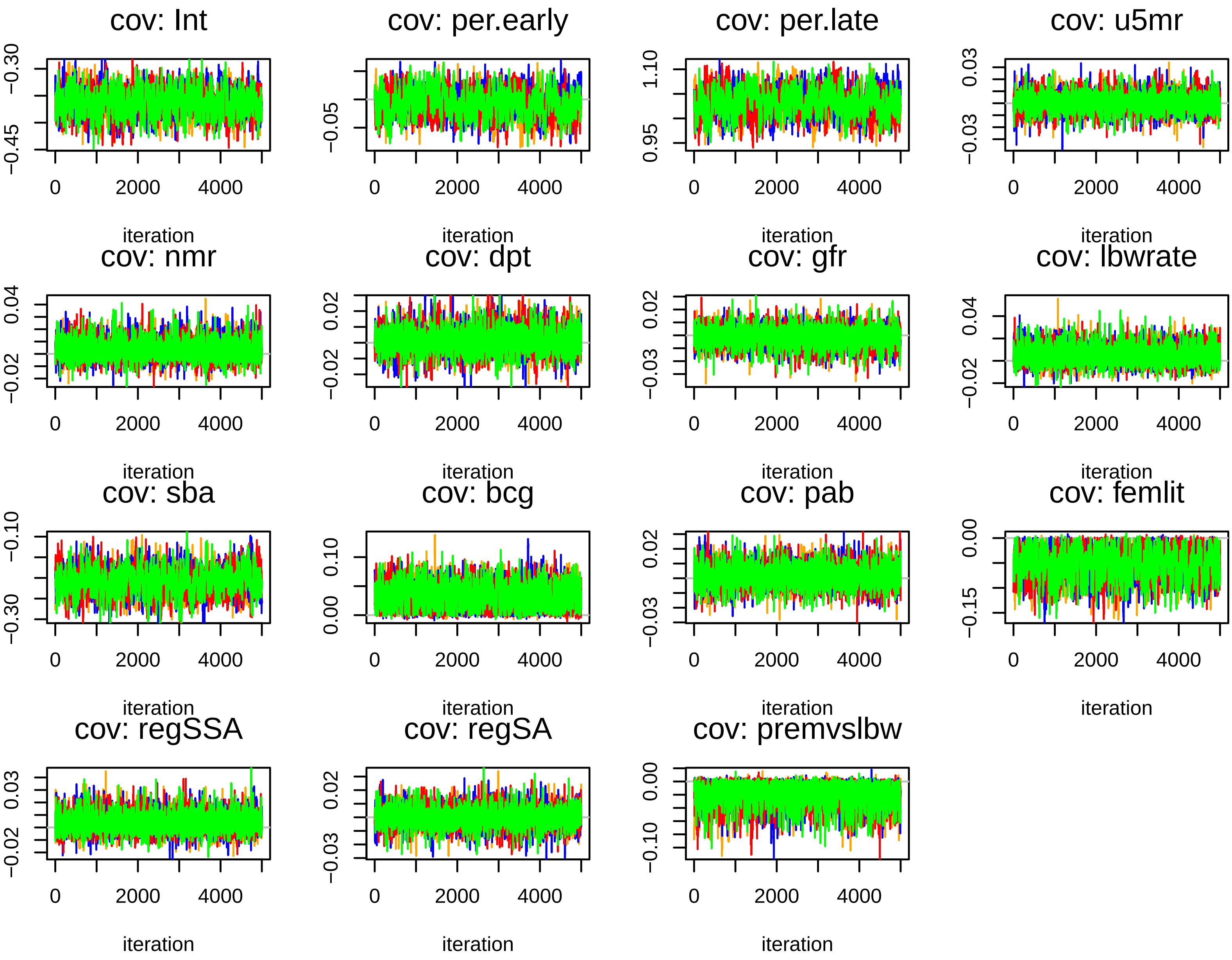

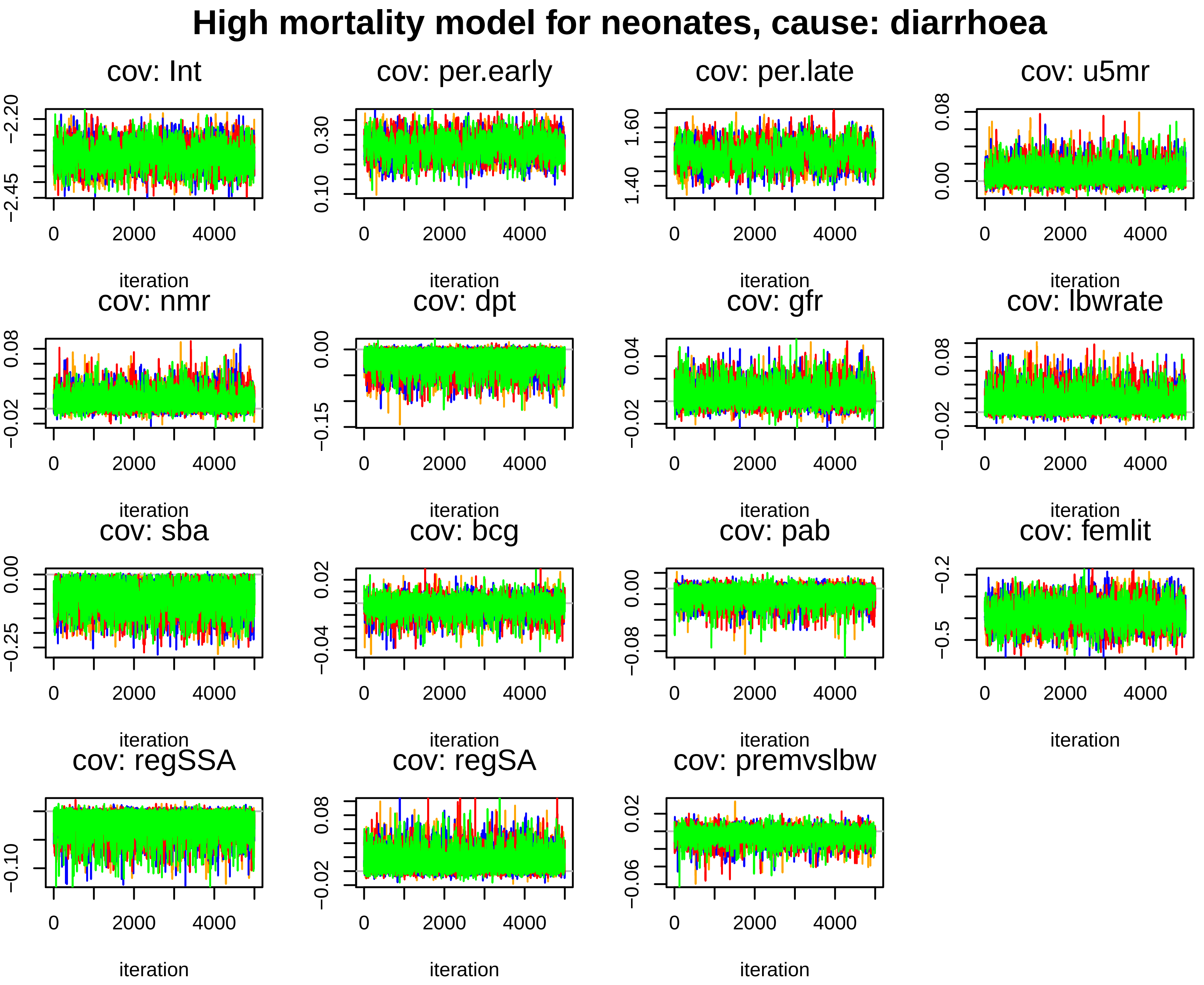
High mortality model for neonates, cause: diarrhoea
cov: Int
cov: per.early
cov: per.late
cov: u5mr
cov: nmr
cov: dpt
cov: gfr
cov: lbwrate
cov: sba
cov: bcg
cov: pab
cov: femlit
cov: regSSA
cov: regSA
cov: premvslbw
iteration
0
2000
4000

High mortality model for neonates, cause: tetanus

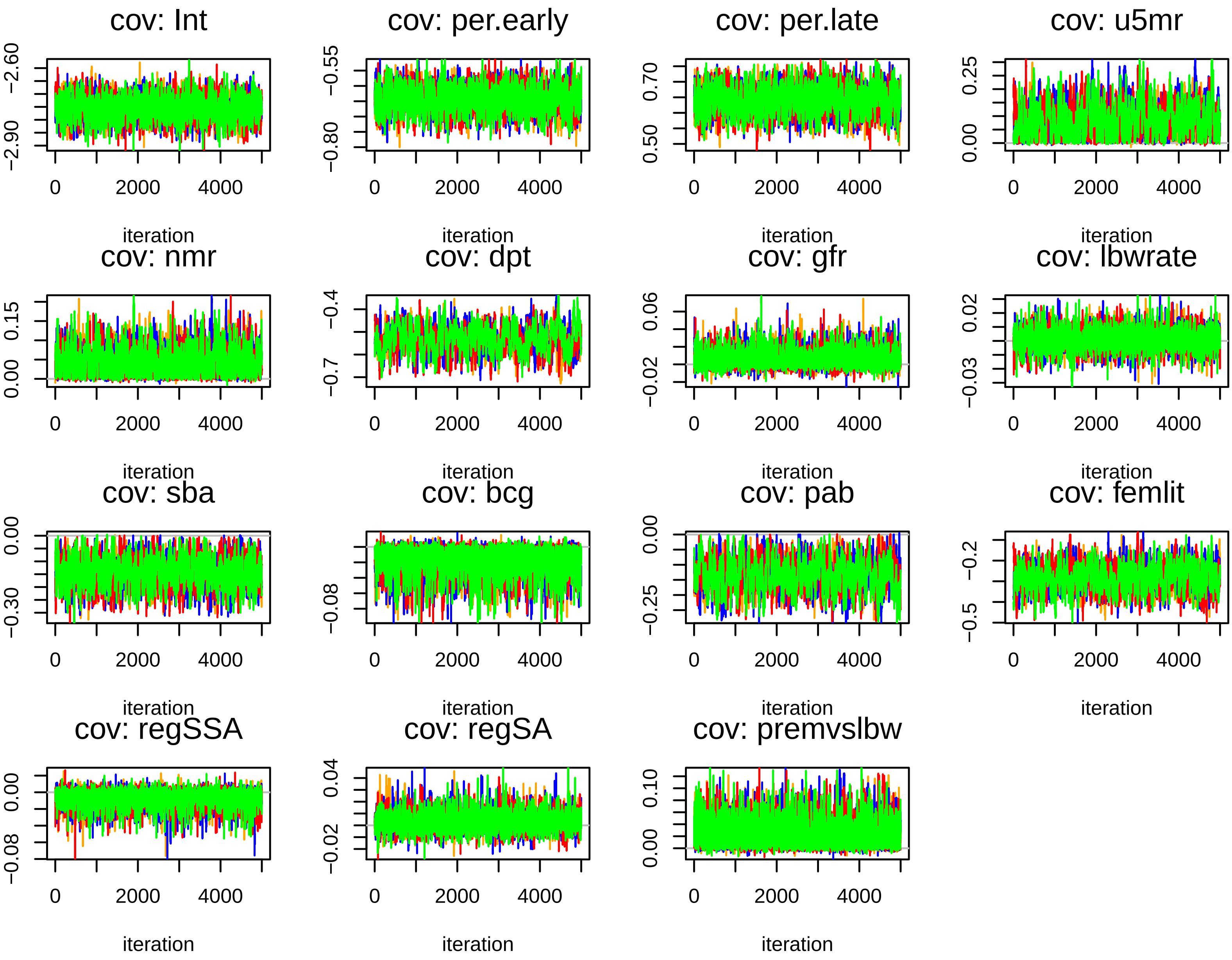

High mortality model for neonates, cause: other

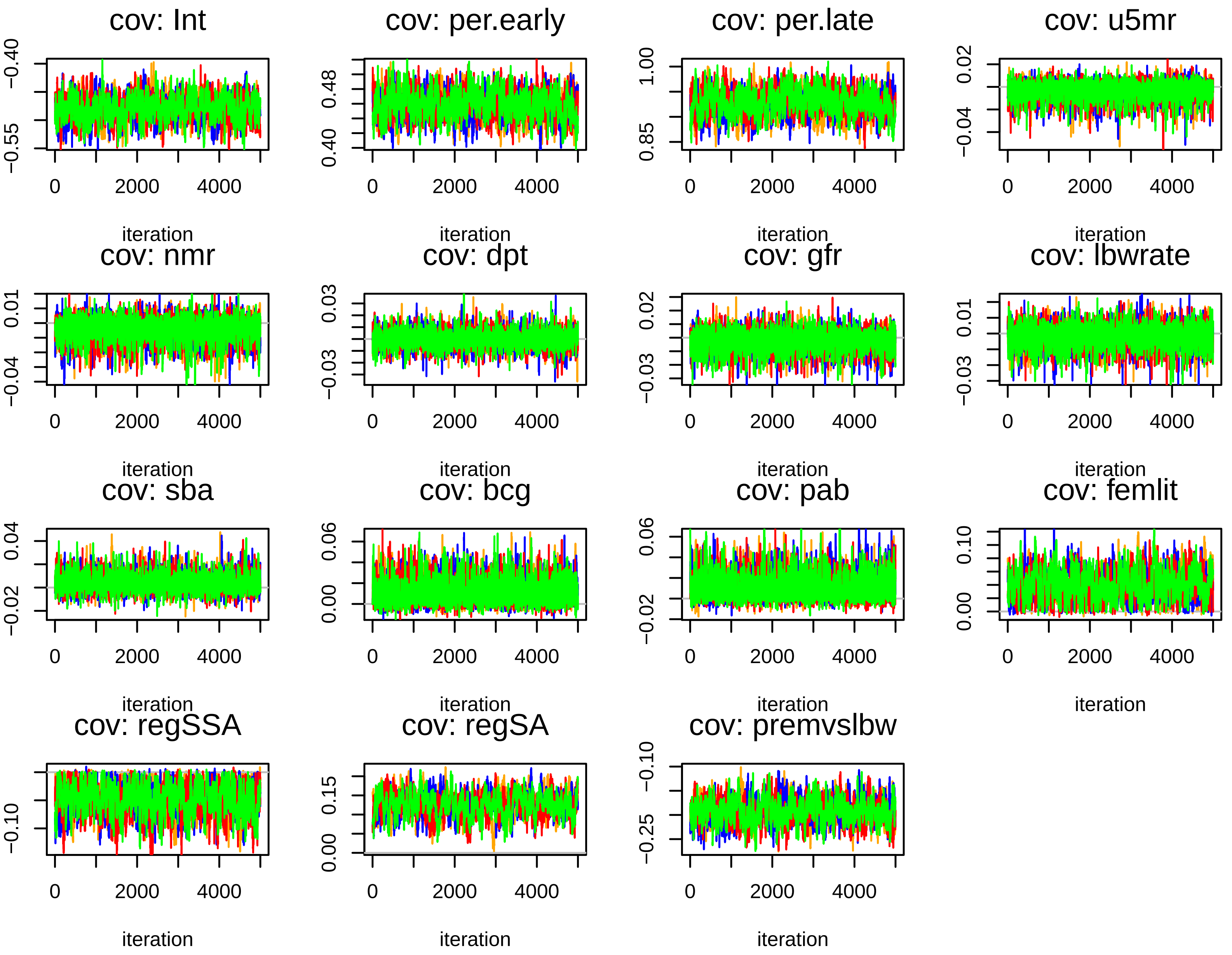

Low mortality model for 1−59 months, cause: diarrhea

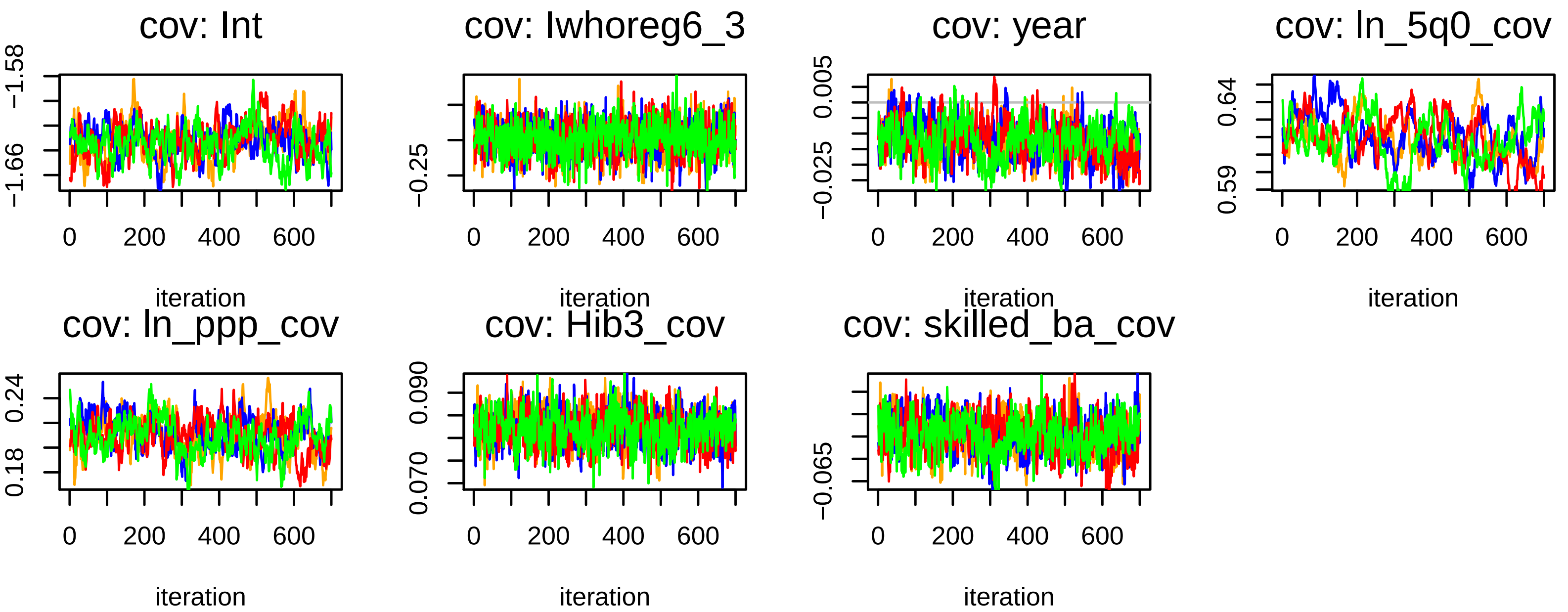

Low mortality model for 1−59 months, cause: meningitis

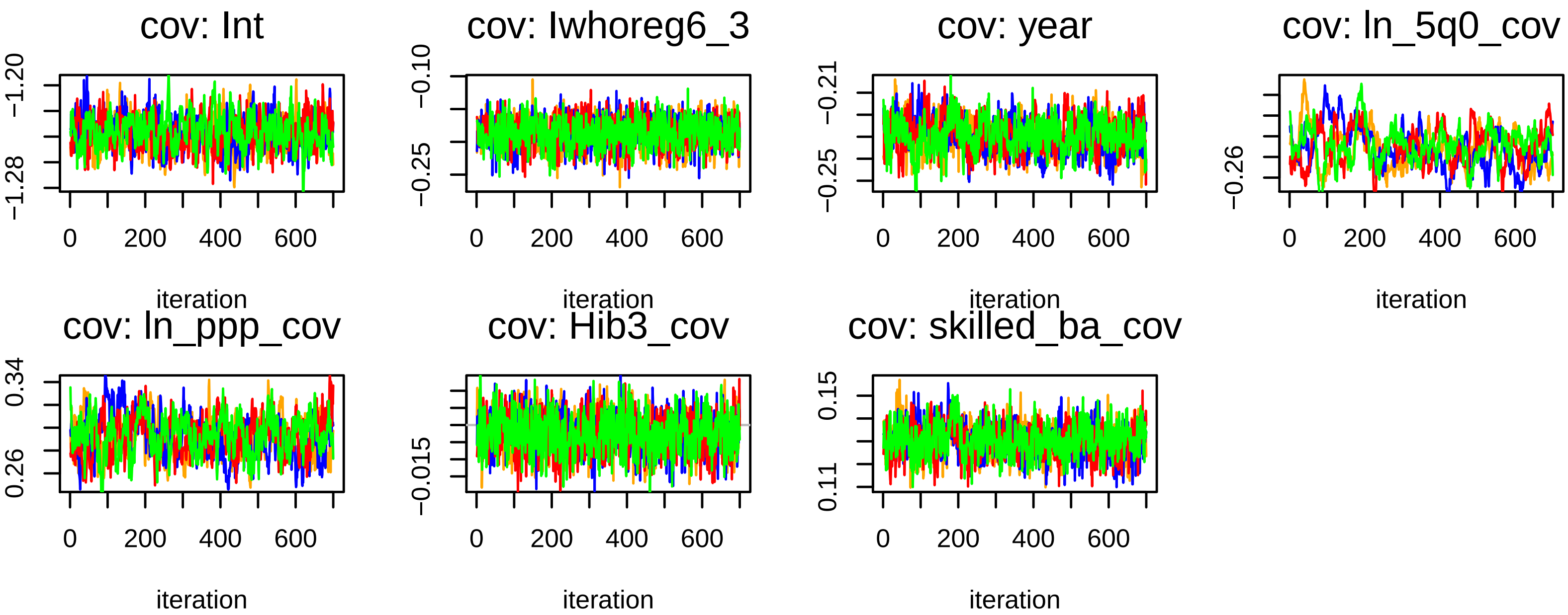

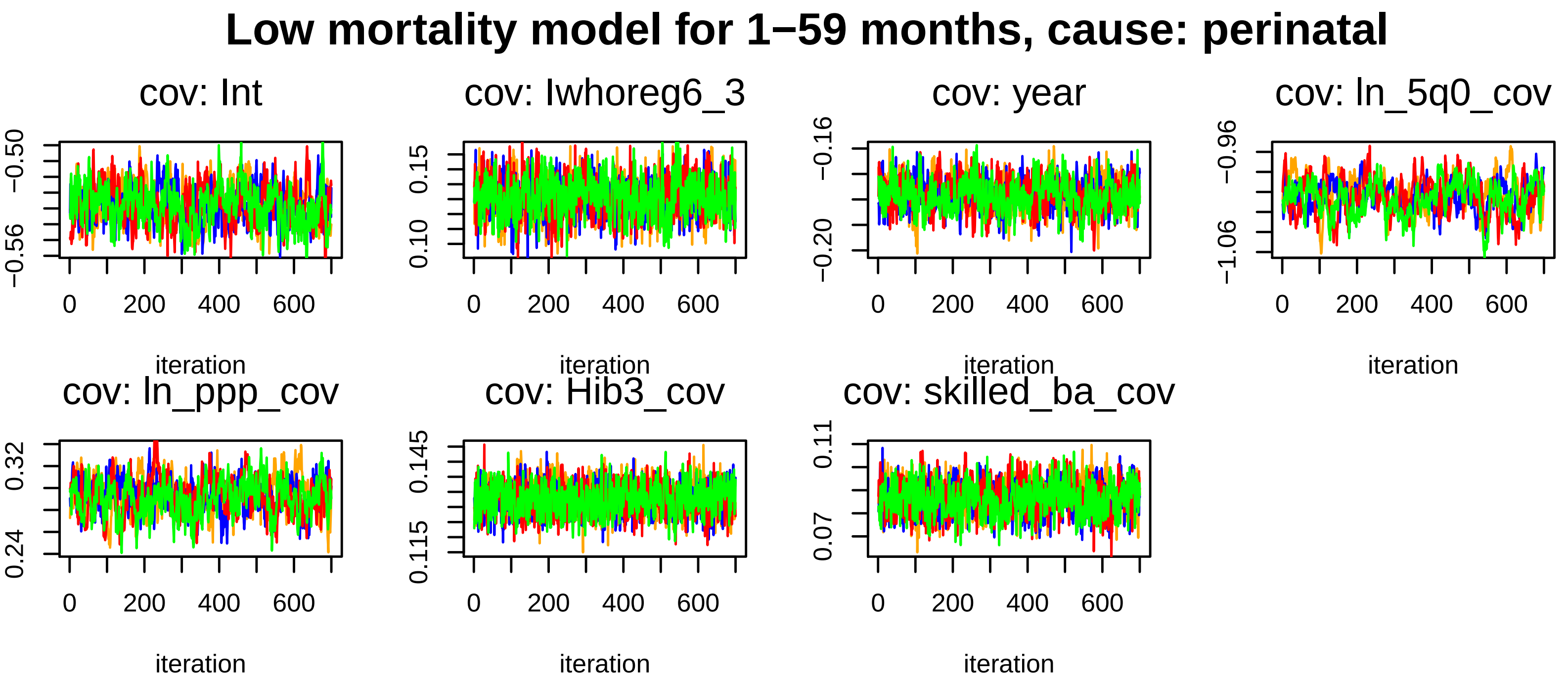
Low mortality model for 1−59 months, cause: perinatal
cov: Int
cov: lwhoreg6_3
cov: year
cov: ln_5q0_cov
cov: ln_ppp_cov
cov: Hib3_cov
cov: skilled_ba_cov
iteration

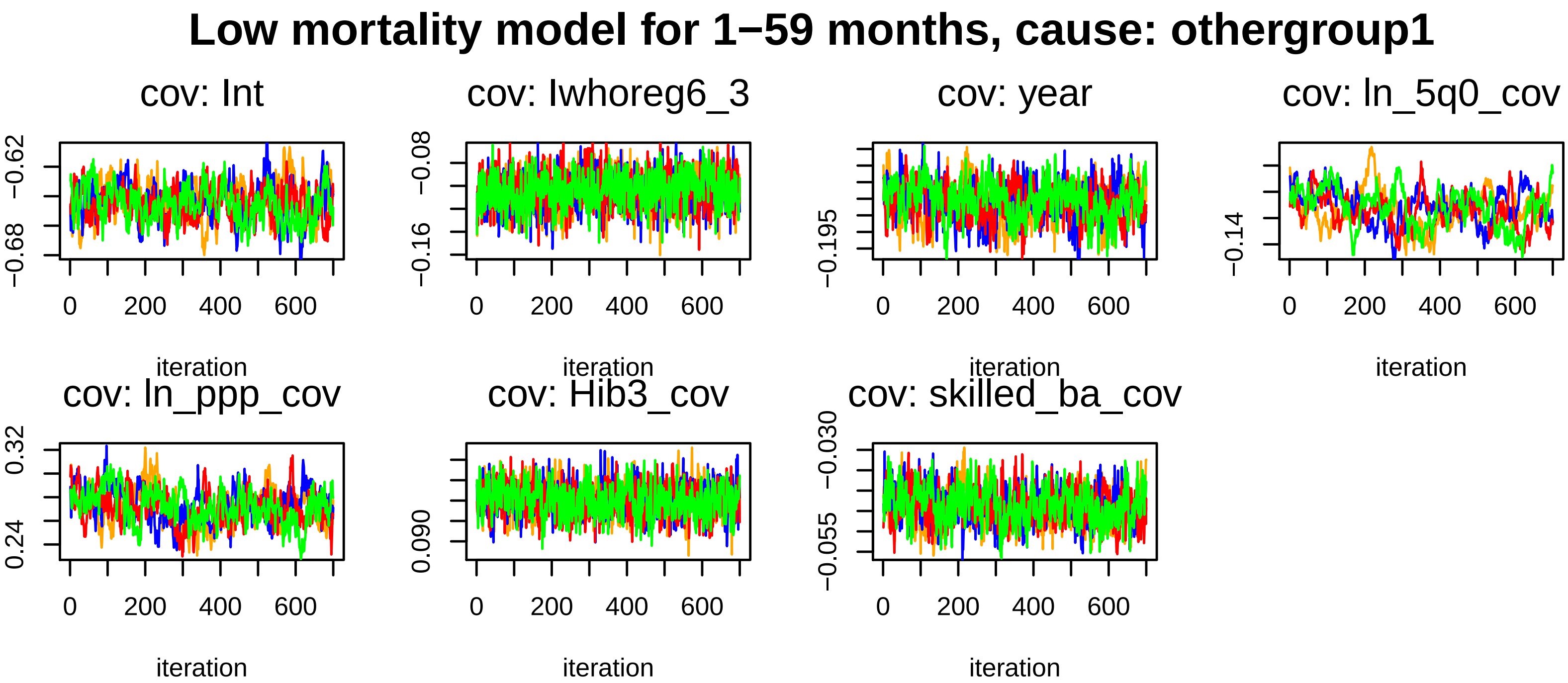
Low mortality model for 1−59 months, cause: othergroup1
cov: Int
−0.68
−0.62
cov: lwhoreg6_3
−0.16
−0.08
cov: year
−0.195
cov: ln_5q0_cov
−0.14
cov: ln_ppp_cov
0.24
0.32
cov: Hib3_cov
0.090
cov: skilled_ba_cov
−0.055
−0.030
0
200
400
600
iteration

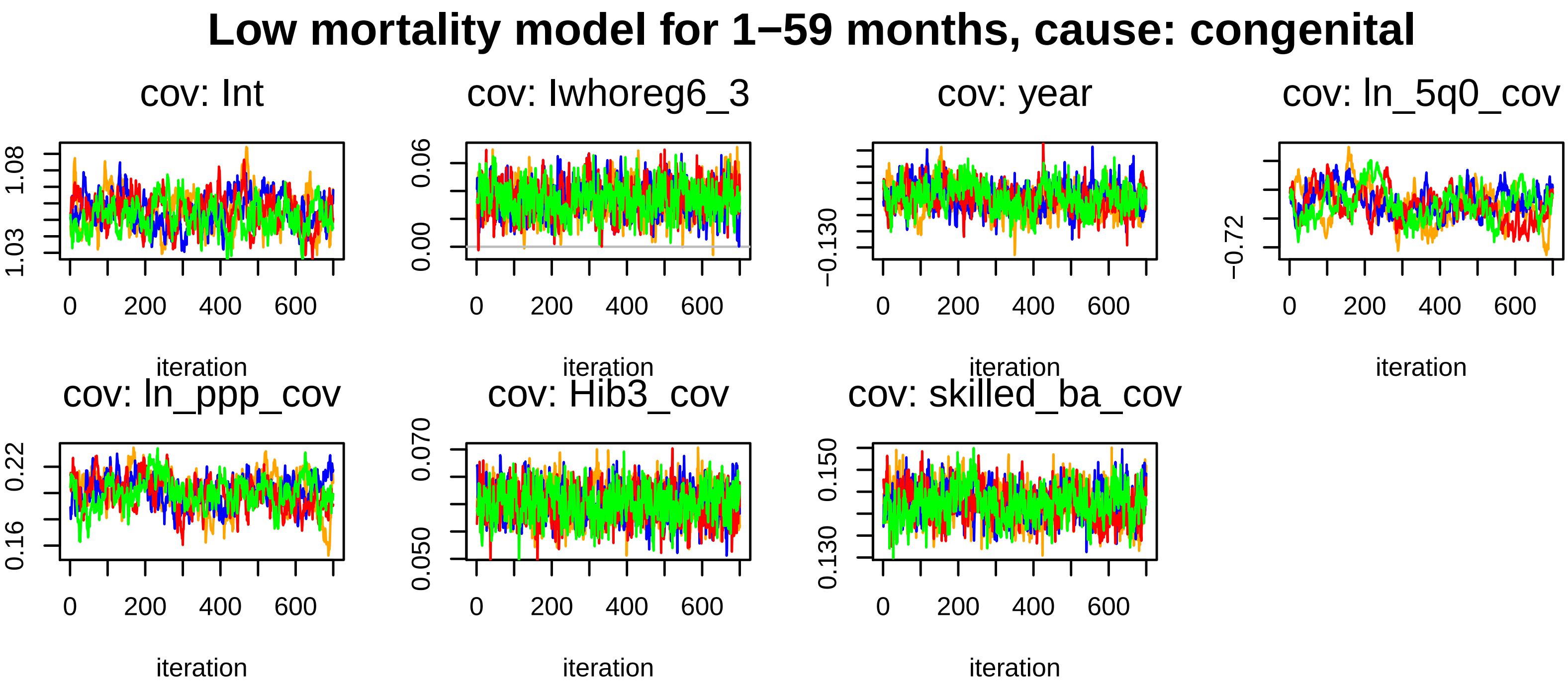

Low mortality model for 1−59 months, cause: congenital
cov: Int
cov: lwhoreg6_3
cov: year
cov: ln_5q0_cov
cov: ln_ppp_cov
cov: Hib3_cov
cov: skilled_ba_cov
iteration
1.03
1.08
0.00
0.06
−0.130
−0.72
0.16
0.22
0.050
0.070
0.130
0.150
0
200
400
600

Low mortality model for 1−59 months, cause: otherncd

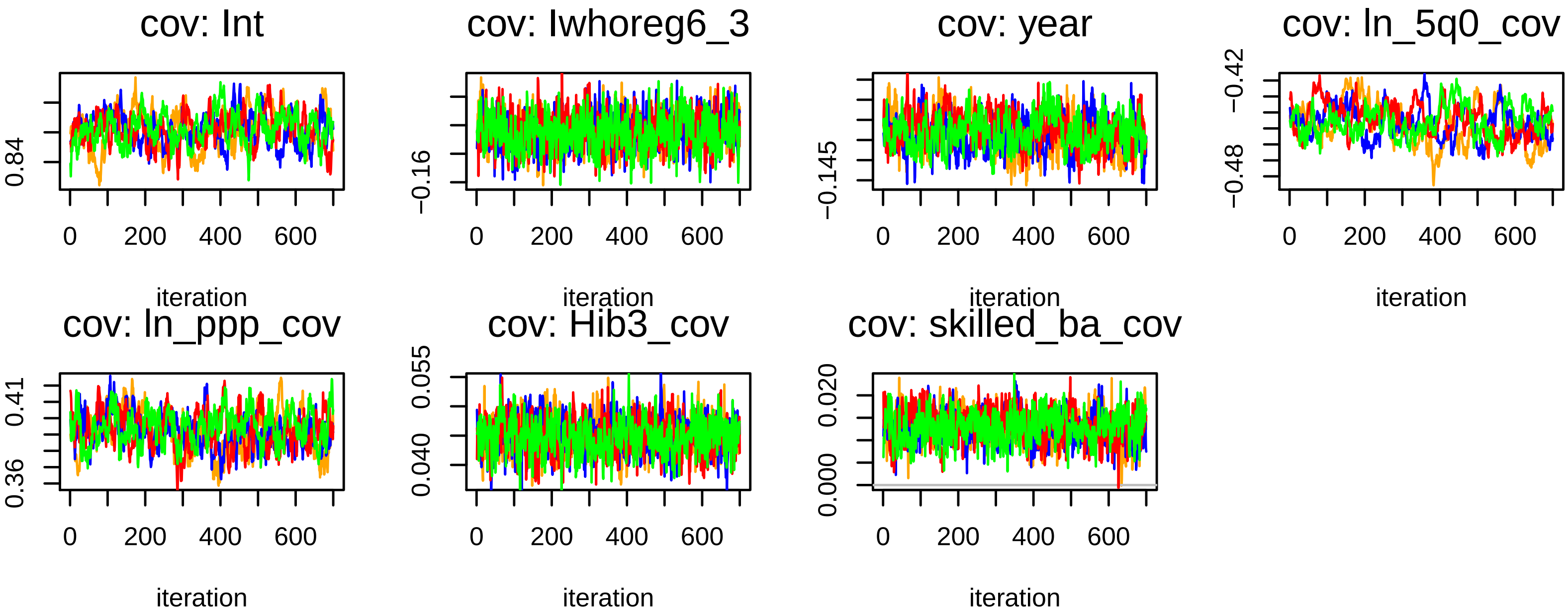

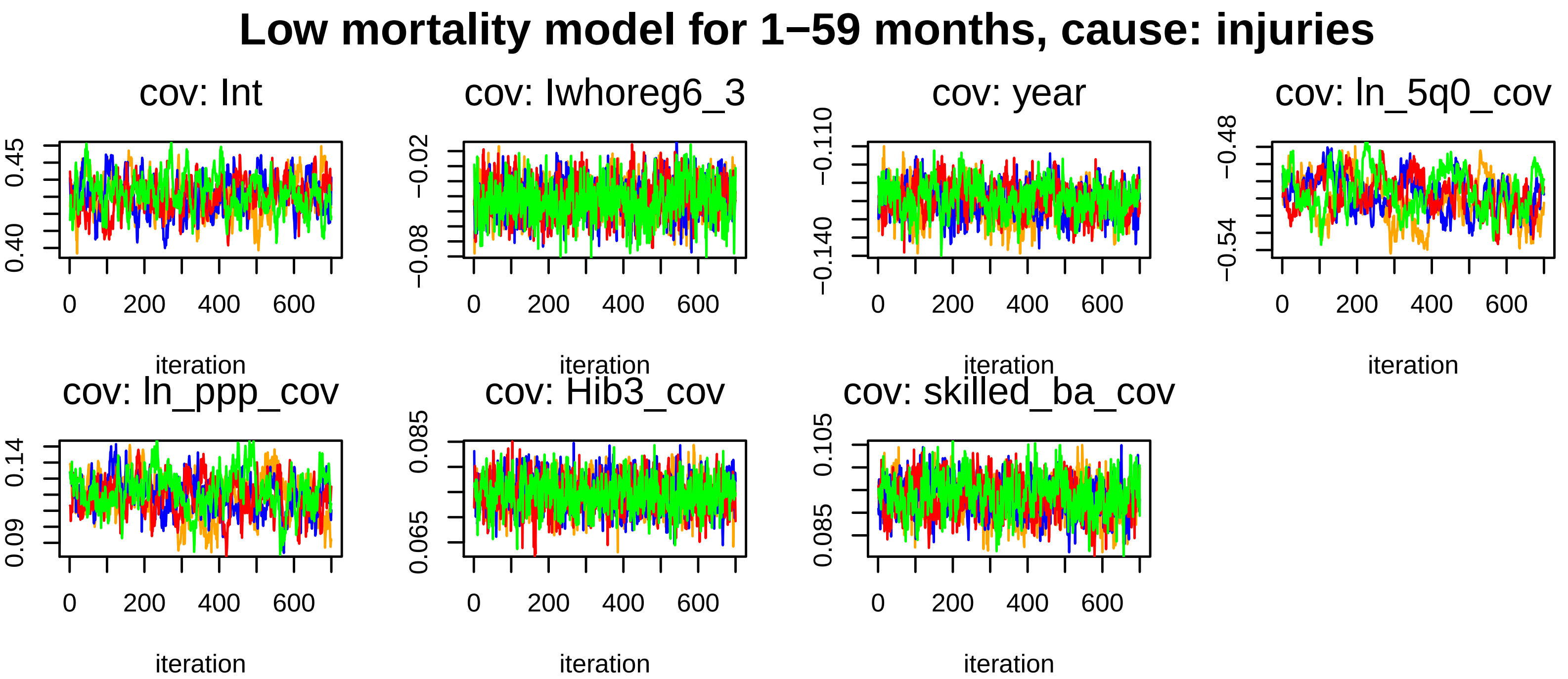
Low mortality model for 1−59 months, cause: injuries
cov: Int
cov: lwhoreg6_3
cov: year
cov: ln_5q0_cov
cov: ln_ppp_cov
cov: Hib3_cov
cov: skilled_ba_cov
iteration

High mortality model for 1−59 months, cause: injuries

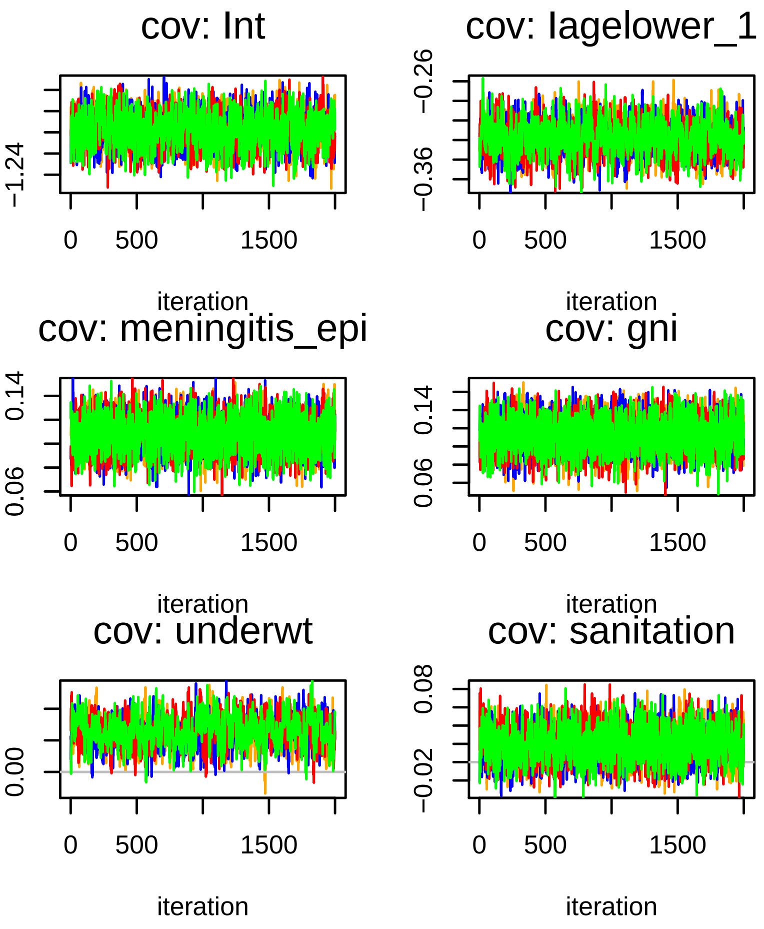

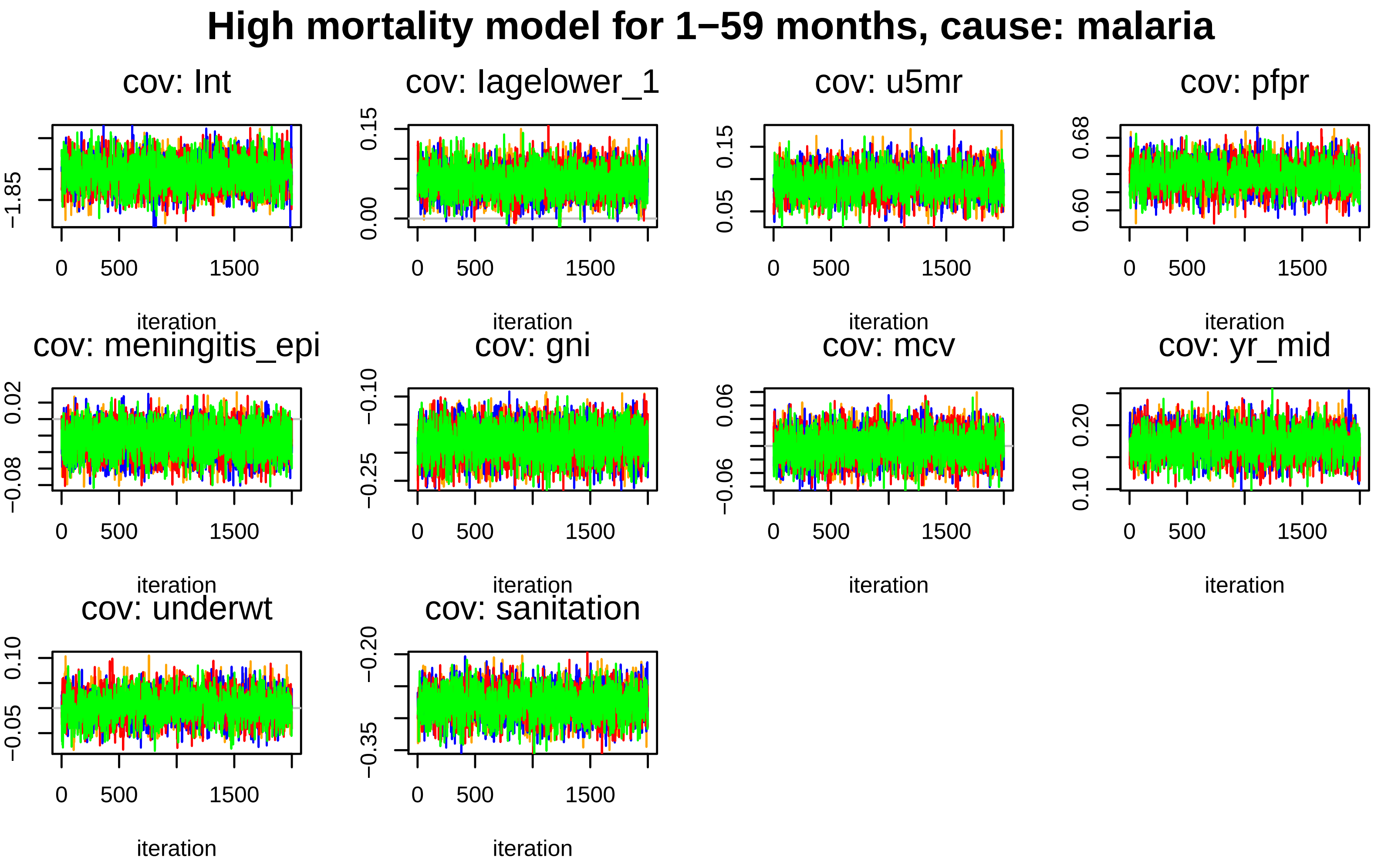
High mortality model for 1−59 months, cause: malaria
cov: Int
cov: lagelower_1
cov: u5mr
cov: pfpr
cov: meningitis_epi
cov: gni
cov: mcv
cov: yr_mid
cov: underwt
cov: sanitation
iteration

High mortality model for 1−59 months, cause: meningitis

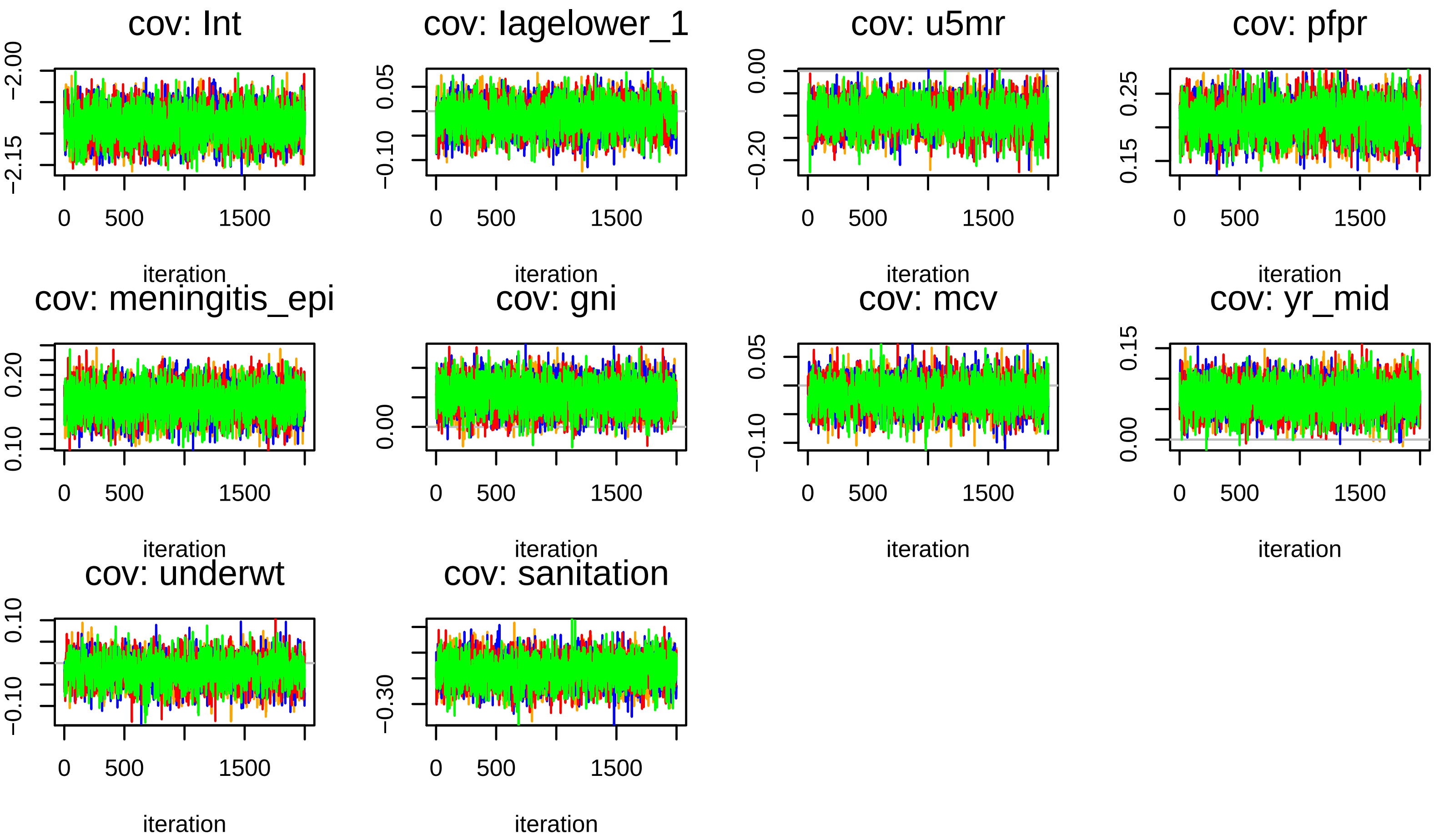

High mortality model for 1−59 months, cause: other

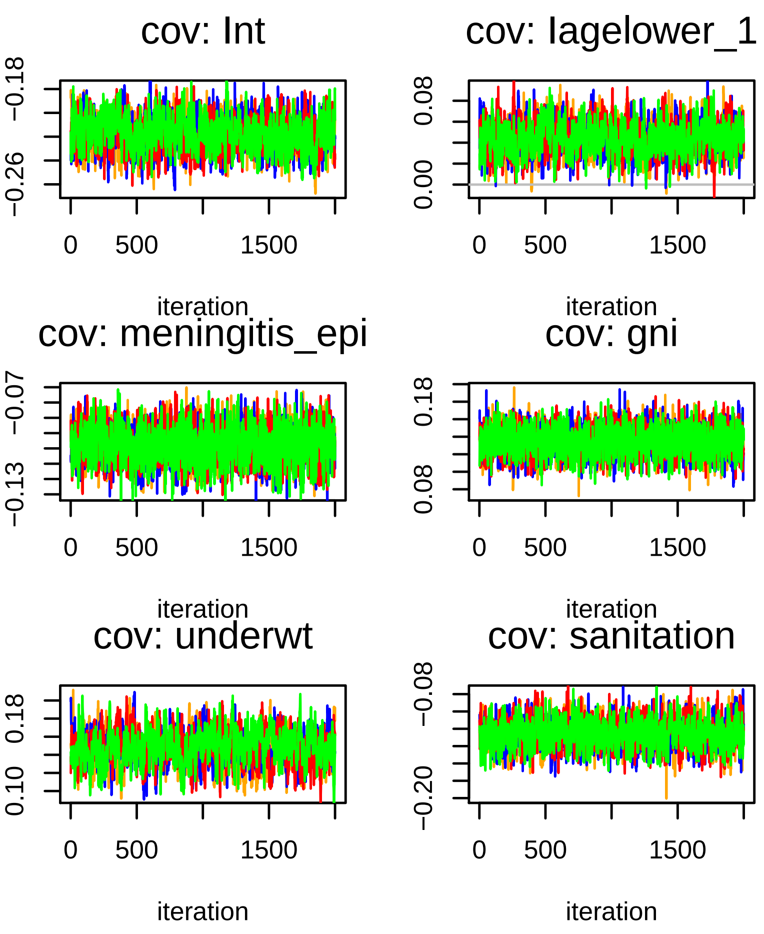

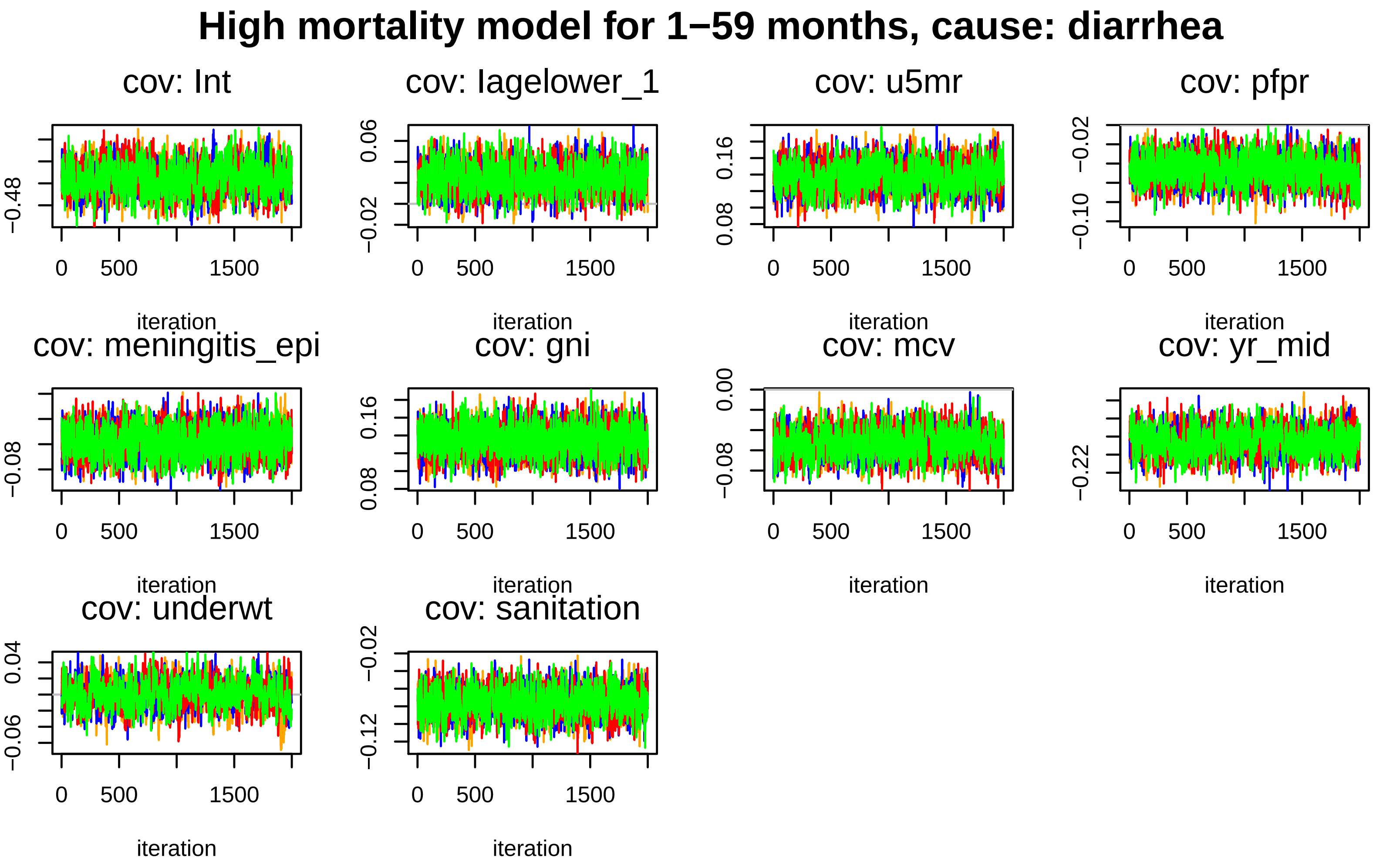
High mortality model for 1−59 months, cause: diarrhea
cov: Int
−0.48
cov: lagelower_1
0.06
−0.02
cov: u5mr
0.16
0.08
cov: pfpr
−0.02
−0.10
cov: meningitis_epi
−0.08
cov: gni
0.16
0.08
cov: mcv
0.00
−0.08
cov: yr_mid
−0.22
cov: underwt
0.04
−0.06
cov: sanitation
−0.02
−0.12
0
500
1500
iteration

High mortality model for 1−59 months, cause: neonatal

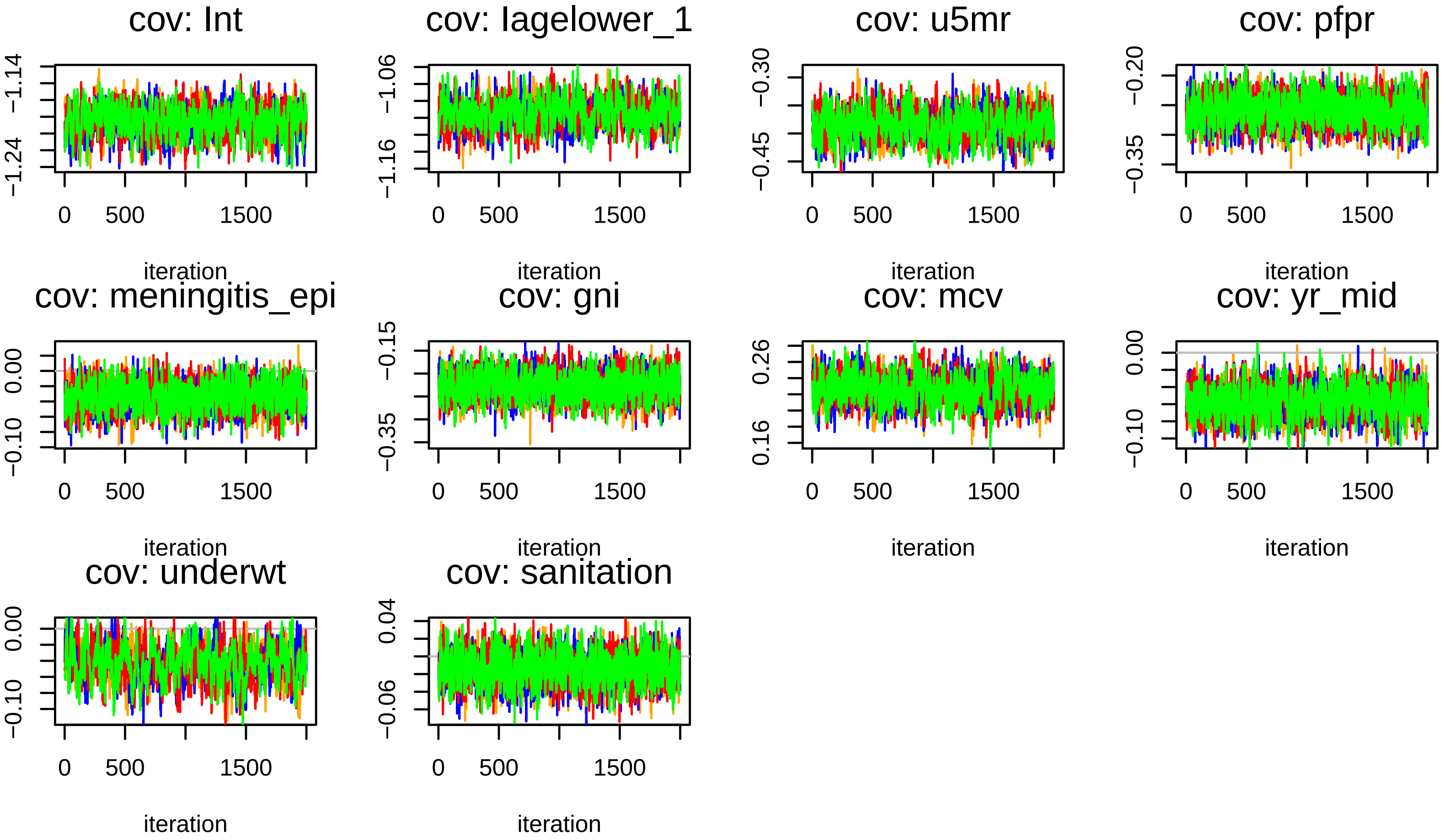

High mortality model for 1−59 months, cause: congenital

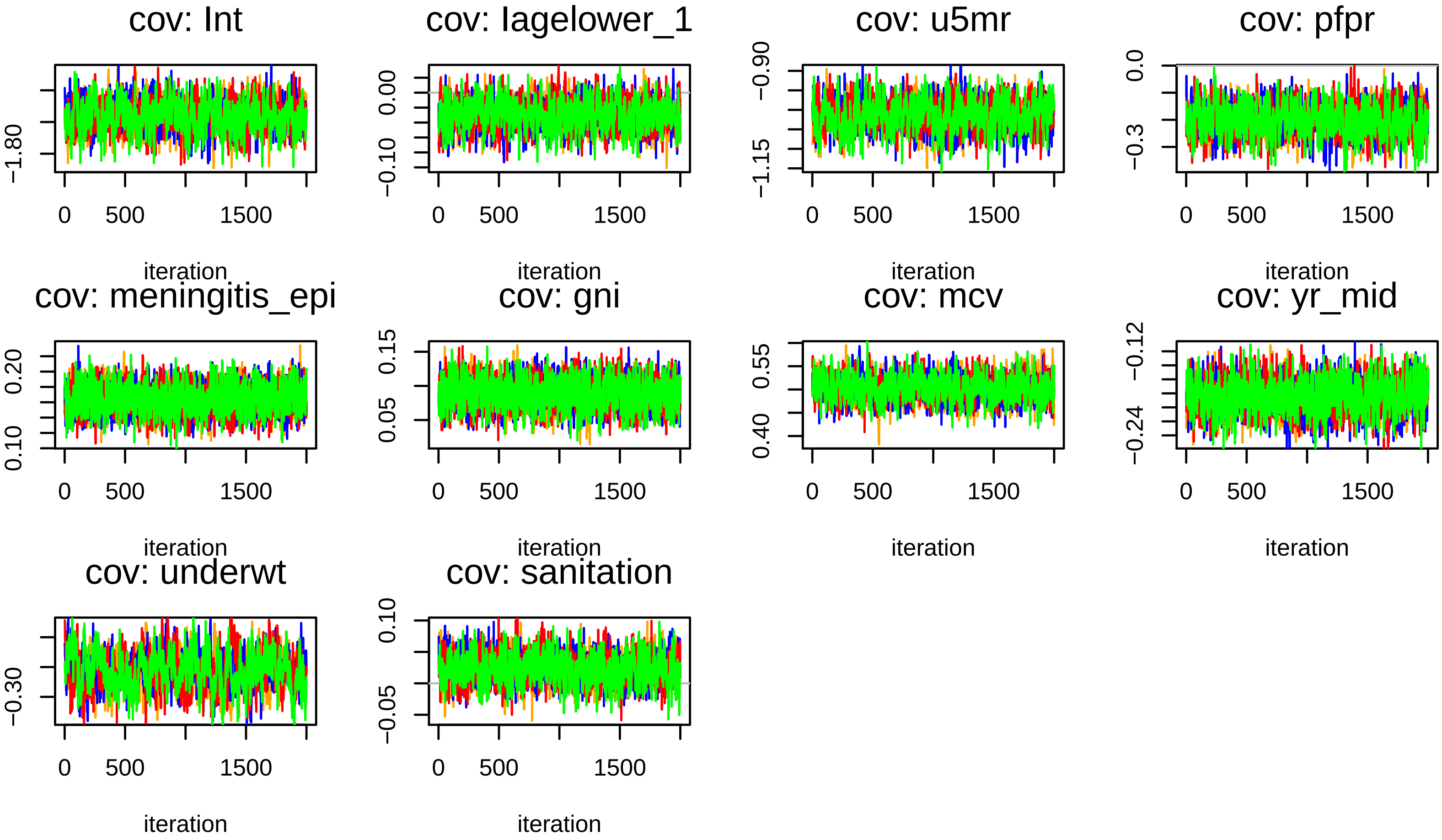

Appendix 6: GATHER checklist

GATHER
Guidelines for Accurate and Transparent Health Estimates Reporting

Checklist of information that should be included in new reports of global health estimates

Item #	Checklist item	Reported on page #
**Objectives and funding**		
**1**	Define the indicator(s), populations (including age, sex, and geographic entities), and time period(s) for which estimates were made.	Introduction (4)
**2**	List the funding sources for the work.	Panel (3)
**Data Inputs**		
For all data inputs from multiple sources that are synthesized as part of the study:		
**3**	Describe how the data were identified and how the data were accessed.	Methods (4)
**4**	Specify the inclusion and exclusion criteria. Identify all ad-hoc exclusions.	Methods (4)
**5**	Provide information on all included data sources and their main characteristics. For each data source used, report reference information or contact name/institution, population represented, data collection method, year(s) of data collection, sex and age range, diagnostic criteria or measurement method, and sample size, as relevant.	Methods (4); webappendix 1; previous publications
**6**	Identify and describe any categories of input data that have potentially important biases (e.g., based on characteristics listed in item 5).	Previous publications
For data inputs that contribute to the analysis but were not synthesized as part of the study:		
**7**	Describe and give sources for any other data inputs.	Methods (6) and previous publications
For all data inputs:		
**8**	Provide all data inputs in a file format from which data can be efficiently extracted (e.g., a spreadsheet rather than a PDF), including all relevant meta-data listed in item 5. For any data inputs that cannot be shared because of ethical or legal reasons, such as third-party ownership, provide a contact name or the name of the institution that retains the right to the data.	Provided via dropbox
**Data analysis**		
**9**	Provide a conceptual overview of the data analysis method. A diagram may be helpful.	Previous publications
**10**	Provide a detailed description of all steps of the analysis, including mathematical formulae. This description should cover, as relevant, data cleaning, data pre-processing, data adjustments and weighting of data sources, and mathematical or statistical model(s).	Methods
**11**	Describe how candidate models were evaluated and how the final model(s) were selected.	In the introduction to the methods
**12**	Provide the results of an evaluation of model performance, if done, as well as the results of any relevant sensitivity analysis.	Webappendix 2
**13**	Describe methods for calculating uncertainty of the estimates. State which sources of uncertainty were, and were not, accounted for in the uncertainty analysis.	Methods (7) and Discussion (11)
**14**	State how analytic or statistical source code used to generate estimates can be accessed.	Code provided via dropbox
**Results and Discussion**		

**15**	Provide published estimates in a file format from which data can be efficiently extracted.	provided via dropbox
**16**	Report a quantitative measure of the uncertainty of the estimates (e.g. uncertainty intervals).	Throughout results section
**17**	Interpret results in light of existing evidence. If updating a previous set of estimates, describe the reasons for changes in estimates.	Throughout discussion section
**18**	Discuss limitations of the estimates. Include a discussion of any modelling assumptions or data limitations that affect interpretation of the estimates.	At the end of the discussion

This checklist should be used in conjunction with the GATHER statement and Explanation and Elaboration document, found on gather-statement.org

Appendix 7: Countries with very low under-5 mortality

Countries with very low mortality for children under five years of age in 2019 (less than 25 per 1000 livebirths)

ISO3	Country name	ISO3	Country name
ALB	Albania	LBN	Lebanon
AND	Andorra	LBY	Libya
ARE	United Arab Emirates	LCA	Saint Lucia
ARG	Argentina	LKA	Sri Lanka
ARM	Armenia	LTU	Lithuania
ATG	Antigua and Barbuda	LUX	Luxembourg
AUS	Australia	LVA	Latvia
AUT	Austria	MAR	Morocco
AZE	Azerbaijan	MCO	Monaco
BEL	Belgium	MDA	Republic of Moldova
BGR	Bulgaria	MDV	Maldives
BHR	Bahrain	MEX	Mexico
BHS	Bahamas	MKD	Republic of North Macedonia
BIH	Bosnia and Herzegovina	MLT	Malta
BLR	Belarus	MNE	Montenegro
BLZ	Belize	MNG	Mongolia
BRA	Brazil	MUS	Mauritius
BRB	Barbados	MYS	Malaysia
BRN	Brunei Darussalam	NIC	Nicaragua
CAN	Canada	NIU	Niue
CHE	Switzerland	NLD	Netherlands
CHL	Chile	NOR	Norway
CHN	China	NZL	New Zealand
COK	Cook Islands	OMN	Oman
COL	Colombia	PAN	Panama
CPV	Cabo Verde	PER	Peru
CRI	Costa Rica	PLW	Palau
CUB	Cuba	POL	Poland
CYP	Cyprus	PRK	Democratic People's Republic of Korea
CZE	Czechia	PRT	Portugal
DEU	Germany	PRY	Paraguay
DNK	Denmark	QAT	Qatar
DZA	Algeria	ROU	Romania
ECU	Ecuador	RUS	Russian Federation
EGY	Egypt	SAU	Saudi Arabia
ESP	Spain	SGP	Singapore
EST	Estonia	SLB	Solomon Islands
FIN	Finland	SLV	El Salvador
FRA	France	SMR	San Marino
GBR	United Kingdom of Great Britain and Northern Ireland	SRB	Serbia
GEO	Georgia	SUR	Suriname
GRC	Greece	SVK	Slovakia
GRD	Grenada	SVN	Slovenia
GTM	Guatemala	SWE	Sweden
HND	Honduras	SYC	Seychelles
HRV	Croatia	SYR	Syrian Arab Republic
HUN	Hungary	THA	Thailand
IDN	Indonesia	TON	Tonga
IRL	Ireland	TTO	Trinidad and Tobago
IRN	Iran (Islamic Republic of)	TUN	Tunisia
ISL	Iceland	TUR	Turkey
ISR	Israel	TUV	Tuvalu
ITA	Italy	UKR	Ukraine
JAM	Jamaica	URY	Uruguay
JOR	Jordan	USA	United States of America
JPN	Japan	UZB	Uzbekistan
KAZ	Kazakhstan	VCT	Saint Vincent and the Grenadines
KGZ	Kyrgyzstan	VEN	Venezuela (Bolivarian Republic of)
KNA	Saint Kitts and Nevis	VNM	Viet Nam
KOR	Republic of Korea	WSM	Samoa
KWT	Kuwait		

Appendix 8: Distribution of causes of child death in UNICEF regions in 2000 and 2019

Webappendix 7. Cause-specific mortality fractions (CSMFs) by UNICEF region in 2000 and 2019

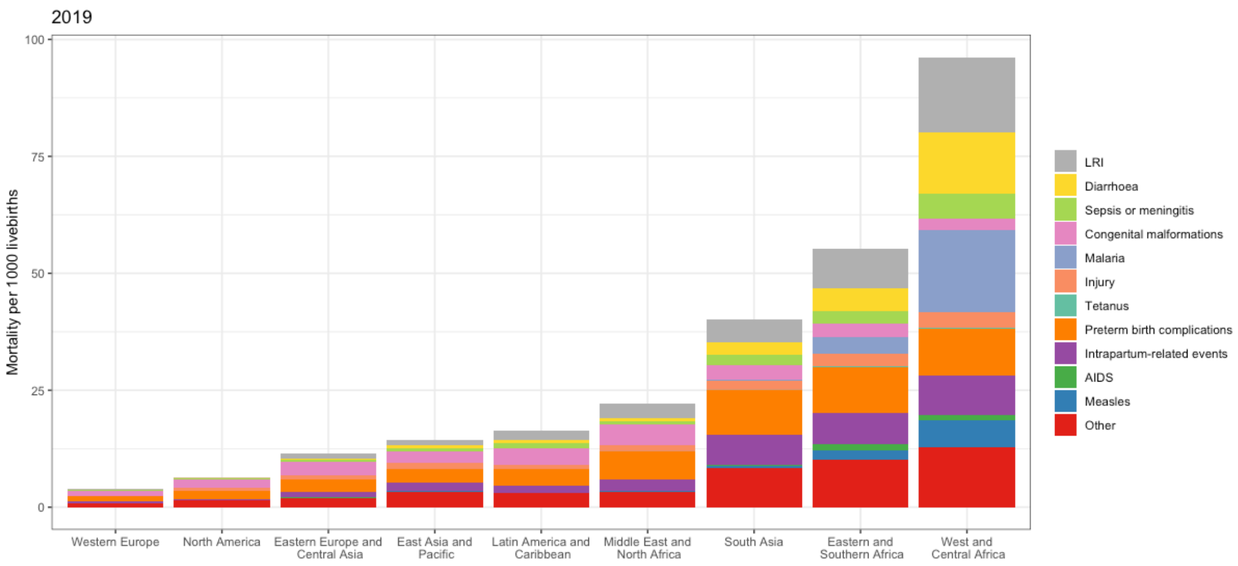

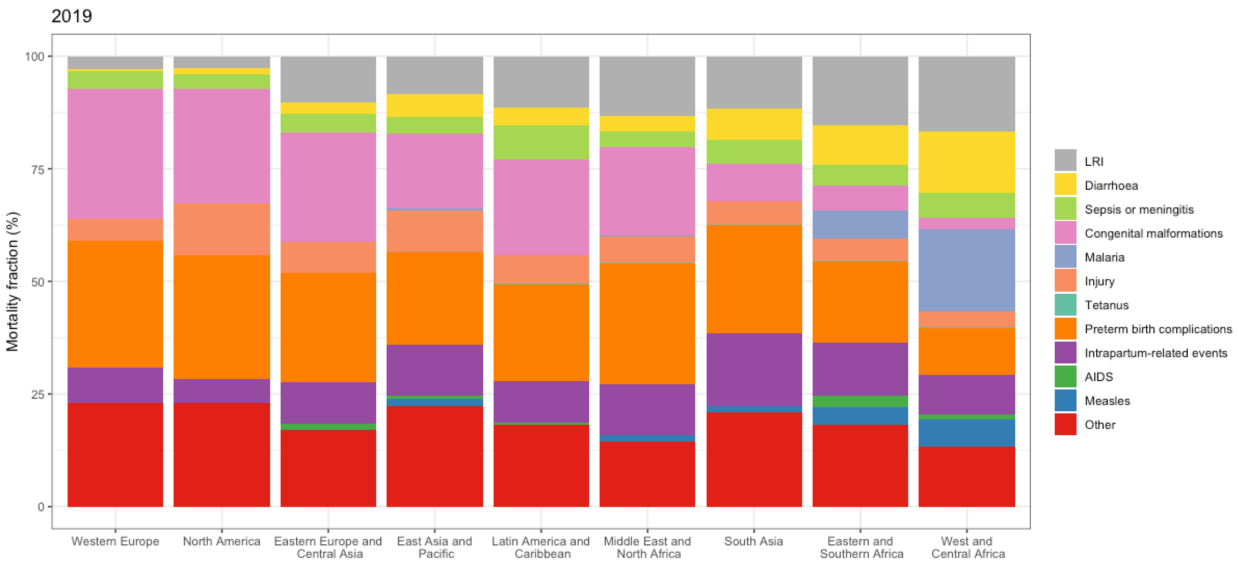

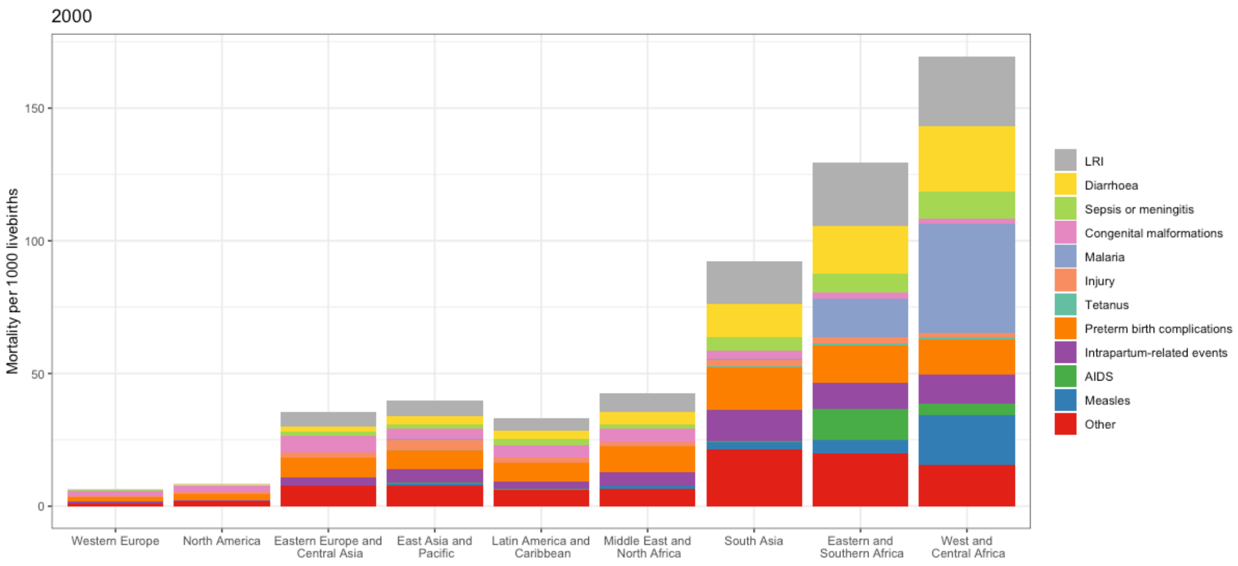

2000
Mortality per 1000 livebirths
0
50
100
150
Western Europe
North America
Eastern Europe and Central Asia
East Asia and Pacific
Latin America and Caribbean
Middle East and North Africa
South Asia
Eastern and Southern Africa
West and Central Africa
LRI
Diarrhoea
Sepsis or meningitis
Congenital malformations
Malaria
Injury
Tetanus
Preterm birth complications
Intrapartum-related events
AIDS
Measles
Other

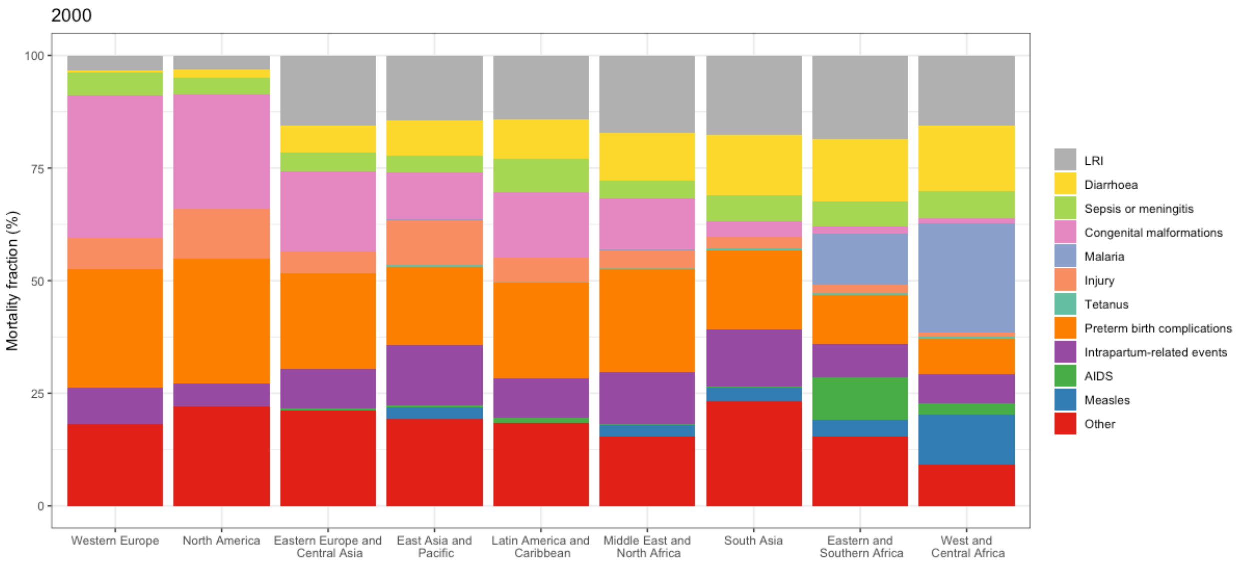

2000
Mortality fraction (%)
0
25
50
75
100
Western Europe
North America
Eastern Europe and Central Asia
East Asia and Pacific
Latin America and Caribbean
Middle East and North Africa
South Asia
Eastern and Southern Africa
West and Central Africa
LRI
Diarrhoea
Sepsis or meningitis
Congenital malformations
Malaria
Injury
Tetanus
Preterm birth complications
Intrapartum-related events
AIDS
Measles
Other

Appendix 9: Average Annual rate of reduction by cause in all countries with 50 or more under-five deaths in 2019 for 2000-2015 and 2015-2019

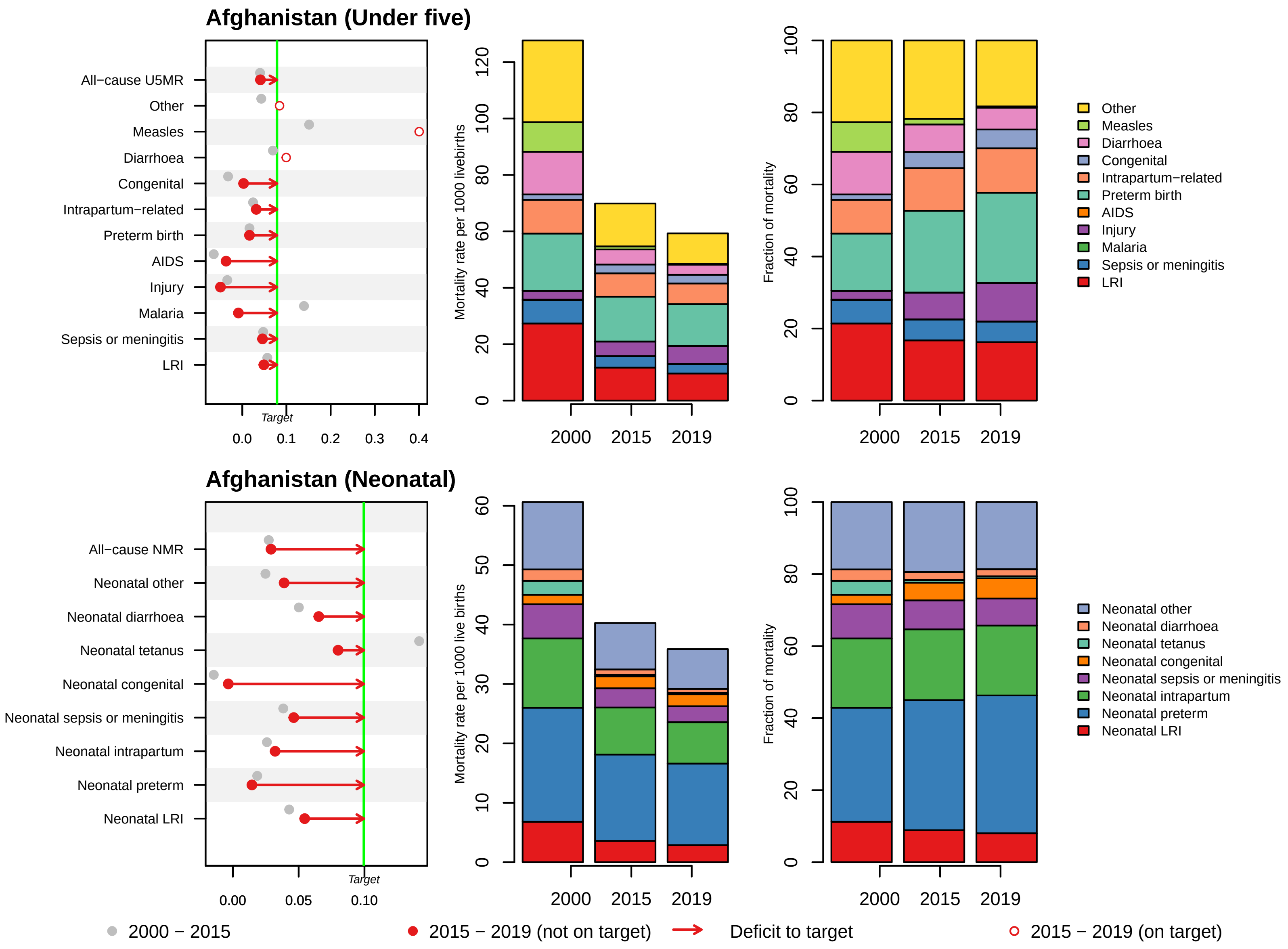

Afghanistan (Under five)
All−cause U5MR
Other
Measles
Diarrhoea
Congenital
Intrapartum−related
Preterm birth
AIDS
Injury
Malaria
Sepsis or meningitis
LRI
Target
0.0
0.1
0.2
0.3
0.4
Mortality rate per 1000 livebirths
0
20
40
60
80
100
120
2000
2015
2019
Fraction of mortality
0
20
40
60
80
100
2000
2015
2019
Other
Measles
Diarrhoea
Congenital
Intrapartum−related
Preterm birth
AIDS
Injury
Malaria
Sepsis or meningitis
LRI
Afghanistan (Neonatal)
All−cause NMR
Neonatal other
Neonatal diarrhoea
Neonatal tetanus
Neonatal congenital
Neonatal sepsis or meningitis
Neonatal intrapartum
Neonatal preterm
Neonatal LRI
Target
0.00
0.05
0.10
Mortality rate per 1000 live births
0
10
20
30
40
50
60
2000
2015
2019
Fraction of mortality
0
20
40
60
80
100
2000
2015
2019
Neonatal other
Neonatal diarrhoea
Neonatal tetanus
Neonatal congenital
Neonatal sepsis or meningitis
Neonatal intrapartum
Neonatal preterm
Neonatal LRI
2000 − 2015
2015 − 2019 (not on target)
Deficit to target
2015 − 2019 (on target)

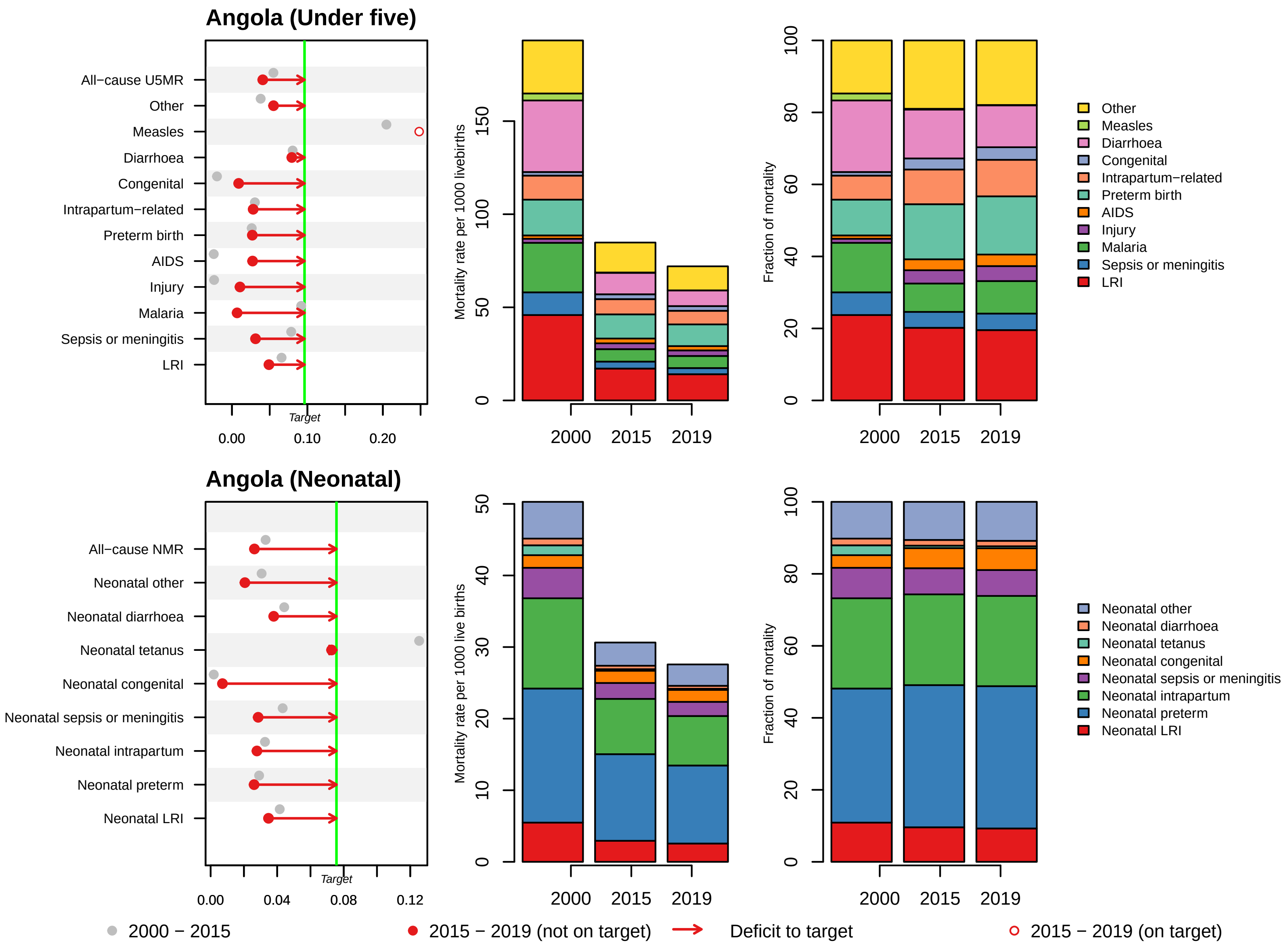

Angola (Under five)
All−cause U5MR
Other
Measles
Diarrhoea
Congenital
Intrapartum−related
Preterm birth
AIDS
Injury
Malaria
Sepsis or meningitis
LRI
Target
0.00
0.10
0.20
Mortality rate per 1000 livebirths
0
50
100
150
2000
2015
2019
Fraction of mortality
0
20
40
60
80
100
Other
Measles
Diarrhoea
Congenital
Intrapartum−related
Preterm birth
AIDS
Injury
Malaria
Sepsis or meningitis
LRI
Angola (Neonatal)
All−cause NMR
Neonatal other
Neonatal diarrhoea
Neonatal tetanus
Neonatal congenital
Neonatal sepsis or meningitis
Neonatal intrapartum
Neonatal preterm
Neonatal LRI
Target
0.00
0.04
0.08
0.12
Mortality rate per 1000 live births
0
10
20
30
40
50
2000
2015
2019
Fraction of mortality
0
20
40
60
80
100
Neonatal other
Neonatal diarrhoea
Neonatal tetanus
Neonatal congenital
Neonatal sepsis or meningitis
Neonatal intrapartum
Neonatal preterm
Neonatal LRI
2000 − 2015
2015 − 2019 (not on target)
Deficit to target
2015 − 2019 (on target)

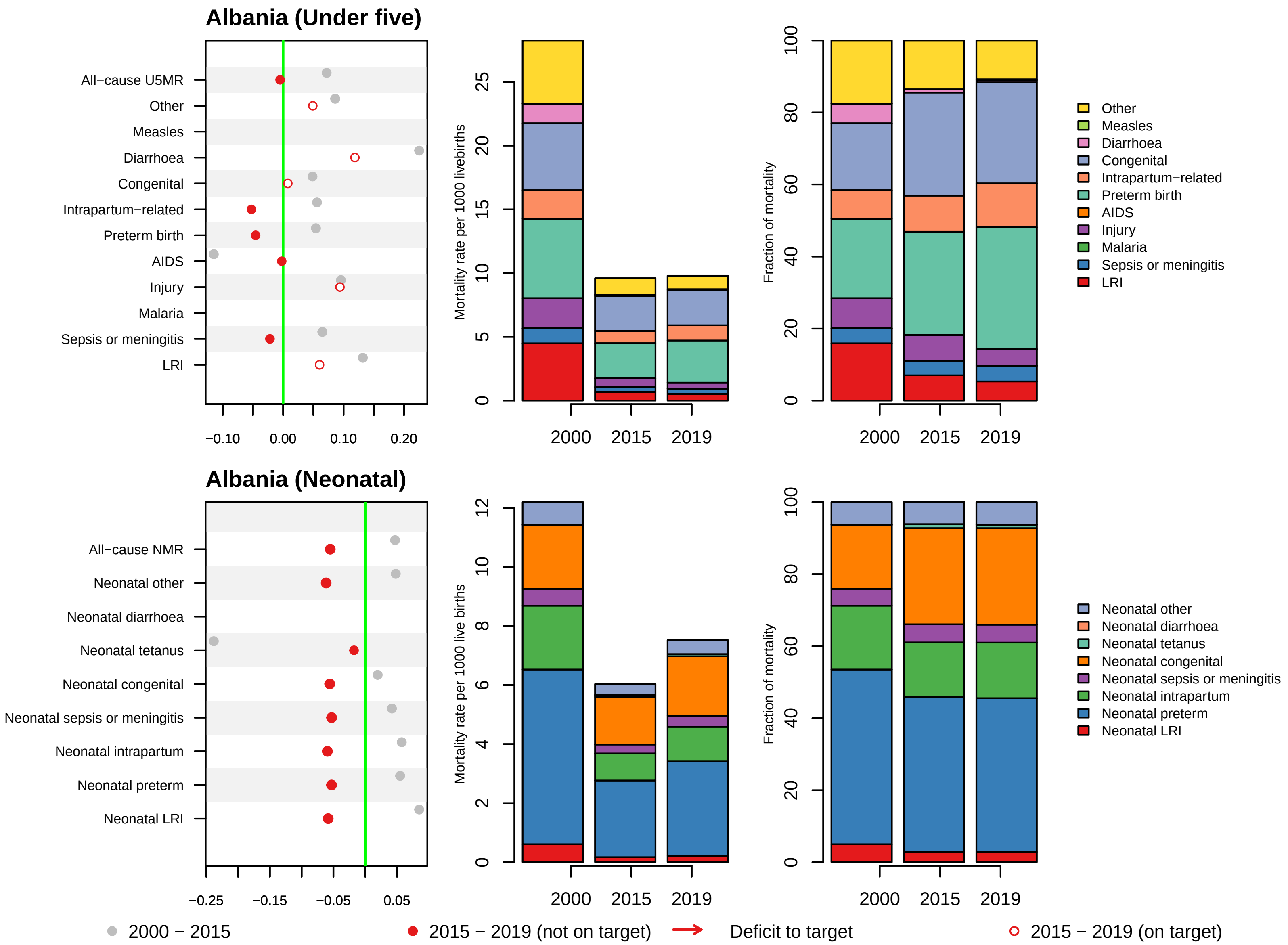

Albania (Under five)
All−cause U5MR
Other
Measles
Diarrhoea
Congenital
Intrapartum−related
Preterm birth
AIDS
Injury
Malaria
Sepsis or meningitis
LRI
−0.10
0.00
0.10
0.20
Mortality rate per 1000 livebirths
Fraction of mortality
2000
2015
2019
Other
Measles
Diarrhoea
Congenital
Intrapartum−related
Preterm birth
AIDS
Injury
Malaria
Sepsis or meningitis
LRI
Albania (Neonatal)
All−cause NMR
Neonatal other
Neonatal diarrhoea
Neonatal tetanus
Neonatal congenital
Neonatal sepsis or meningitis
Neonatal intrapartum
Neonatal preterm
Neonatal LRI
−0.25
−0.15
−0.05
0.05
Mortality rate per 1000 live births
Fraction of mortality
2000
2015
2019
Neonatal other
Neonatal diarrhoea
Neonatal tetanus
Neonatal congenital
Neonatal sepsis or meningitis
Neonatal intrapartum
Neonatal preterm
Neonatal LRI
2000 − 2015
2015 − 2019 (not on target)
Deficit to target
2015 − 2019 (on target)

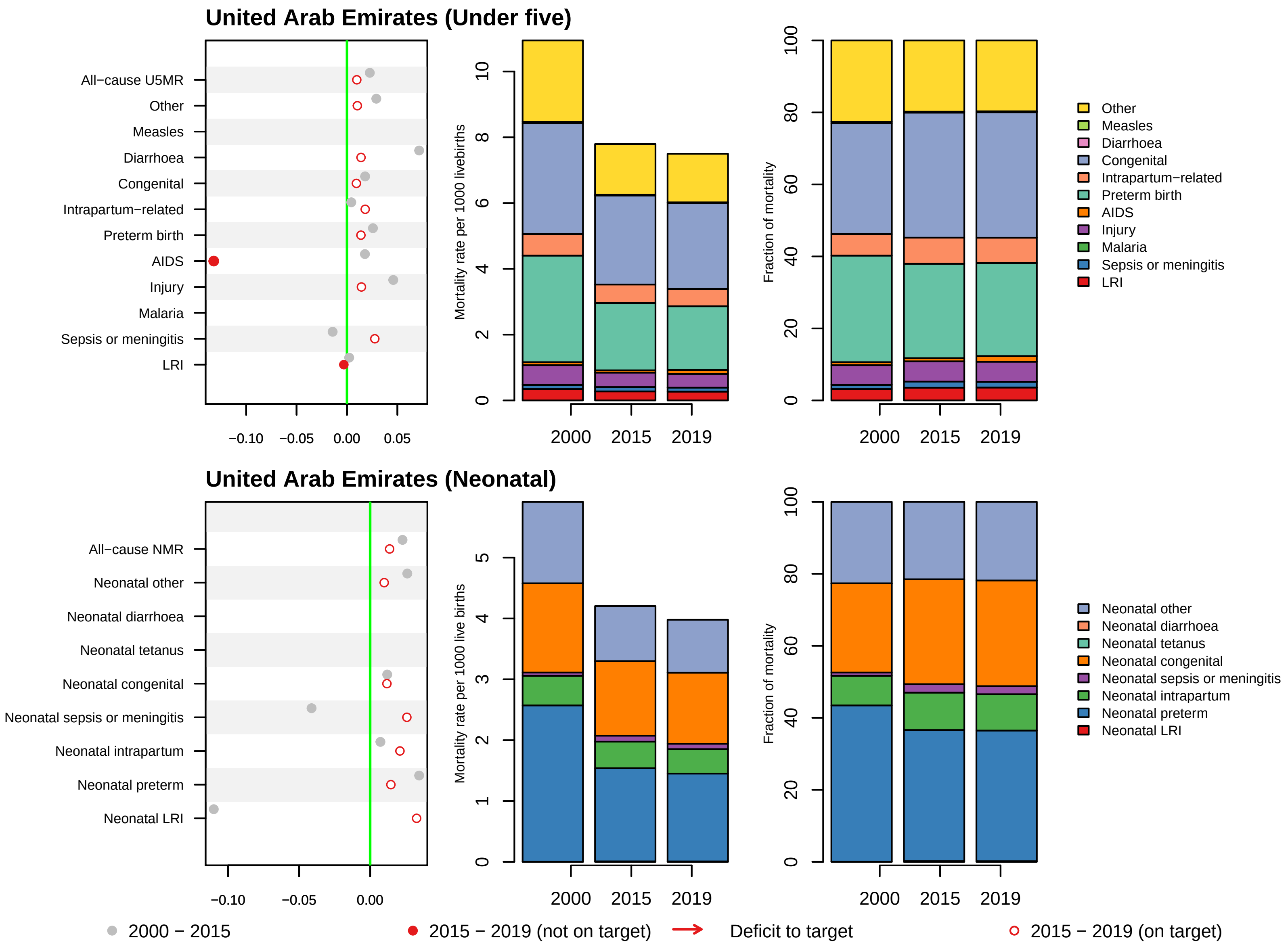
United Arab Emirates (Under five)
All−cause U5MR
Other
Measles
Diarrhoea
Congenital
Intrapartum−related
Preterm birth
AIDS
Injury
Malaria
Sepsis or meningitis
LRI
−0.10
−0.05
0.00
0.05
Mortality rate per 1000 livebirths
0
2
4
6
8
10
2000
2015
2019
Fraction of mortality
0
20
40
60
80
100
Other
Measles
Diarrhoea
Congenital
Intrapartum−related
Preterm birth
AIDS
Injury
Malaria
Sepsis or meningitis
LRI
United Arab Emirates (Neonatal)
All−cause NMR
Neonatal other
Neonatal diarrhoea
Neonatal tetanus
Neonatal congenital
Neonatal sepsis or meningitis
Neonatal intrapartum
Neonatal preterm
Neonatal LRI
−0.10
−0.05
0.00
Mortality rate per 1000 live births
0
1
2
3
4
5
Neonatal other
Neonatal diarrhoea
Neonatal tetanus
Neonatal congenital
Neonatal sepsis or meningitis
Neonatal intrapartum
Neonatal preterm
Neonatal LRI
2000 − 2015
2015 − 2019 (not on target)
Deficit to target
2015 − 2019 (on target)

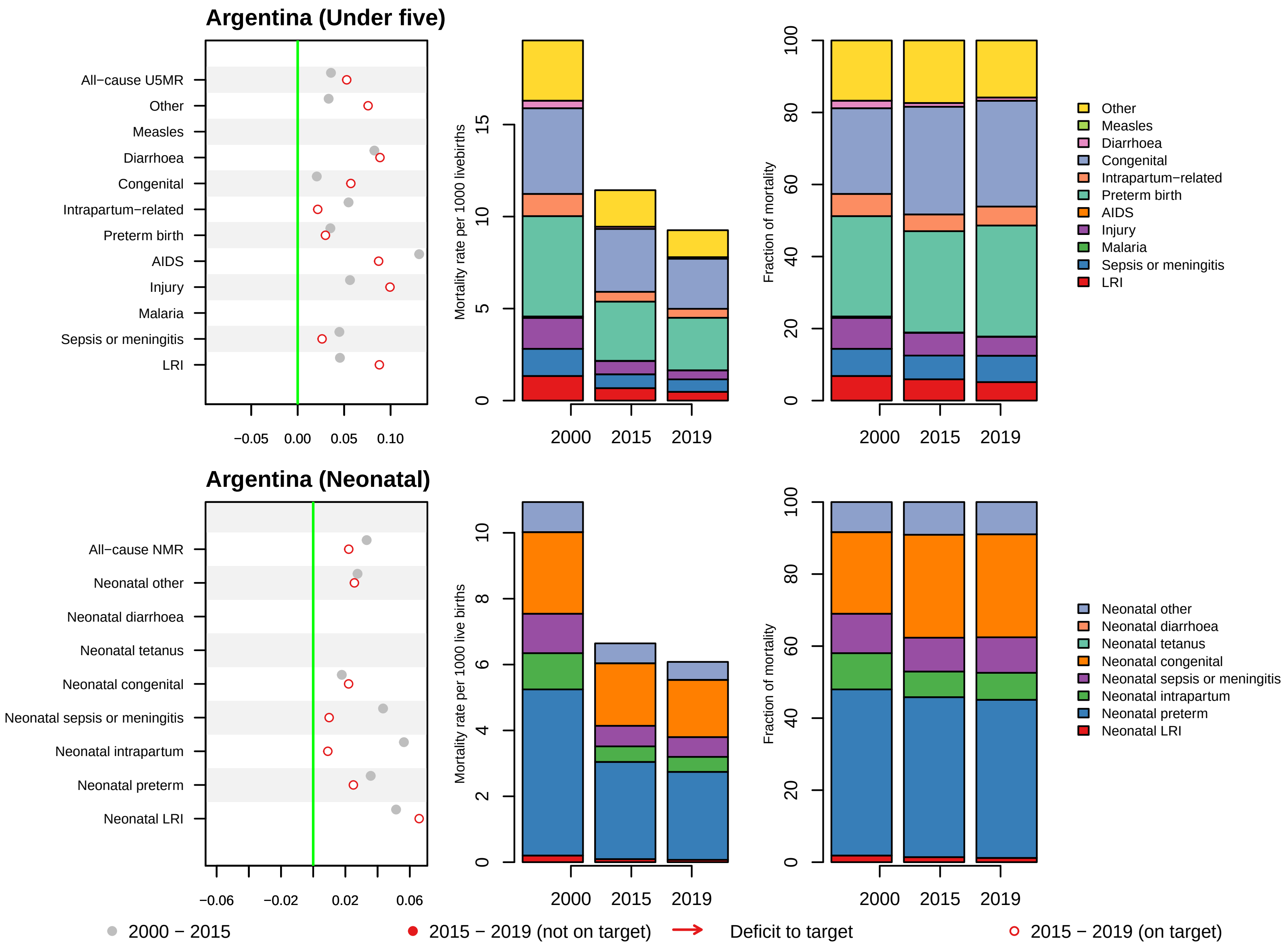
Argentina (Under five)
All−cause U5MR
Other
Measles
Diarrhoea
Congenital
Intrapartum−related
Preterm birth
AIDS
Injury
Malaria
Sepsis or meningitis
LRI
−0.05
0.00
0.05
0.10
Mortality rate per 1000 livebirths
0
5
10
15
2000
2015
2019
Fraction of mortality
0
20
40
60
80
100
Other
Measles
Diarrhoea
Congenital
Intrapartum−related
Preterm birth
AIDS
Injury
Malaria
Sepsis or meningitis
LRI
Argentina (Neonatal)
All−cause NMR
Neonatal other
Neonatal diarrhoea
Neonatal tetanus
Neonatal congenital
Neonatal sepsis or meningitis
Neonatal intrapartum
Neonatal preterm
Neonatal LRI
−0.06
−0.02
0.02
0.06
Mortality rate per 1000 live births
0
2
4
6
8
10
Neonatal other
Neonatal diarrhoea
Neonatal tetanus
Neonatal congenital
Neonatal sepsis or meningitis
Neonatal intrapartum
Neonatal preterm
Neonatal LRI
2000 − 2015
2015 − 2019 (not on target)
Deficit to target
2015 − 2019 (on target)

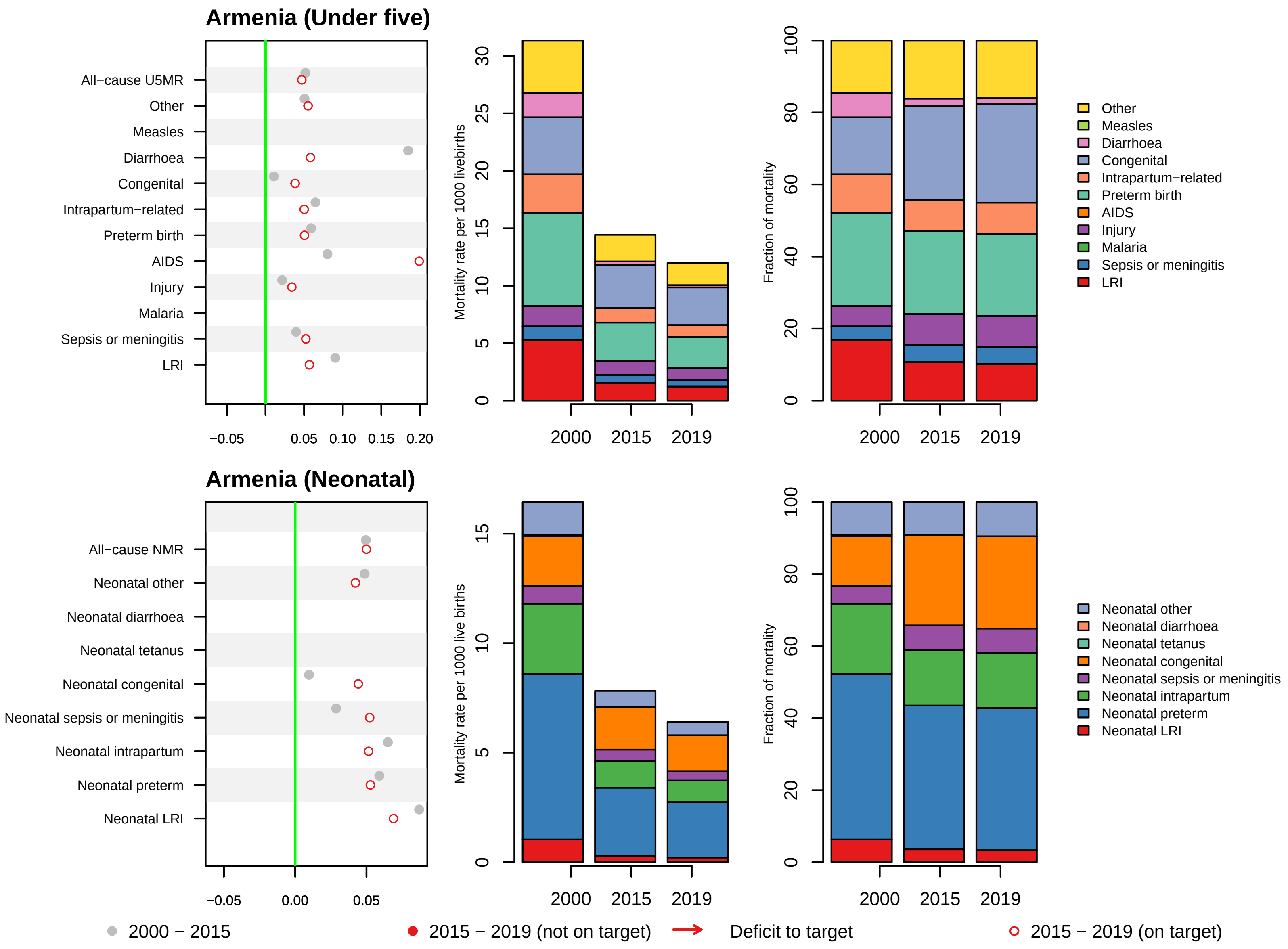
Armenia (Under five)
All−cause U5MR
Other
Measles
Diarrhoea
Congenital
Intrapartum−related
Preterm birth
AIDS
Injury
Malaria
Sepsis or meningitis
LRI
−0.05
0.05
0.10
0.15
0.20
Mortality rate per 1000 livebirths
0
5
10
15
20
25
30
2000
2015
2019
Fraction of mortality
0
20
40
60
80
100
2000
2015
2019
Other
Measles
Diarrhoea
Congenital
Intrapartum−related
Preterm birth
AIDS
Injury
Malaria
Sepsis or meningitis
LRI
Armenia (Neonatal)
All−cause NMR
Neonatal other
Neonatal diarrhoea
Neonatal tetanus
Neonatal congenital
Neonatal sepsis or meningitis
Neonatal intrapartum
Neonatal preterm
Neonatal LRI
−0.05
0.00
0.05
Mortality rate per 1000 live births
0
5
10
15
2000
2015
2019
Fraction of mortality
0
20
40
60
80
100
2000
2015
2019
Neonatal other
Neonatal diarrhoea
Neonatal tetanus
Neonatal congenital
Neonatal sepsis or meningitis
Neonatal intrapartum
Neonatal preterm
Neonatal LRI
2000 − 2015
2015 − 2019 (not on target)
Deficit to target
2015 − 2019 (on target)

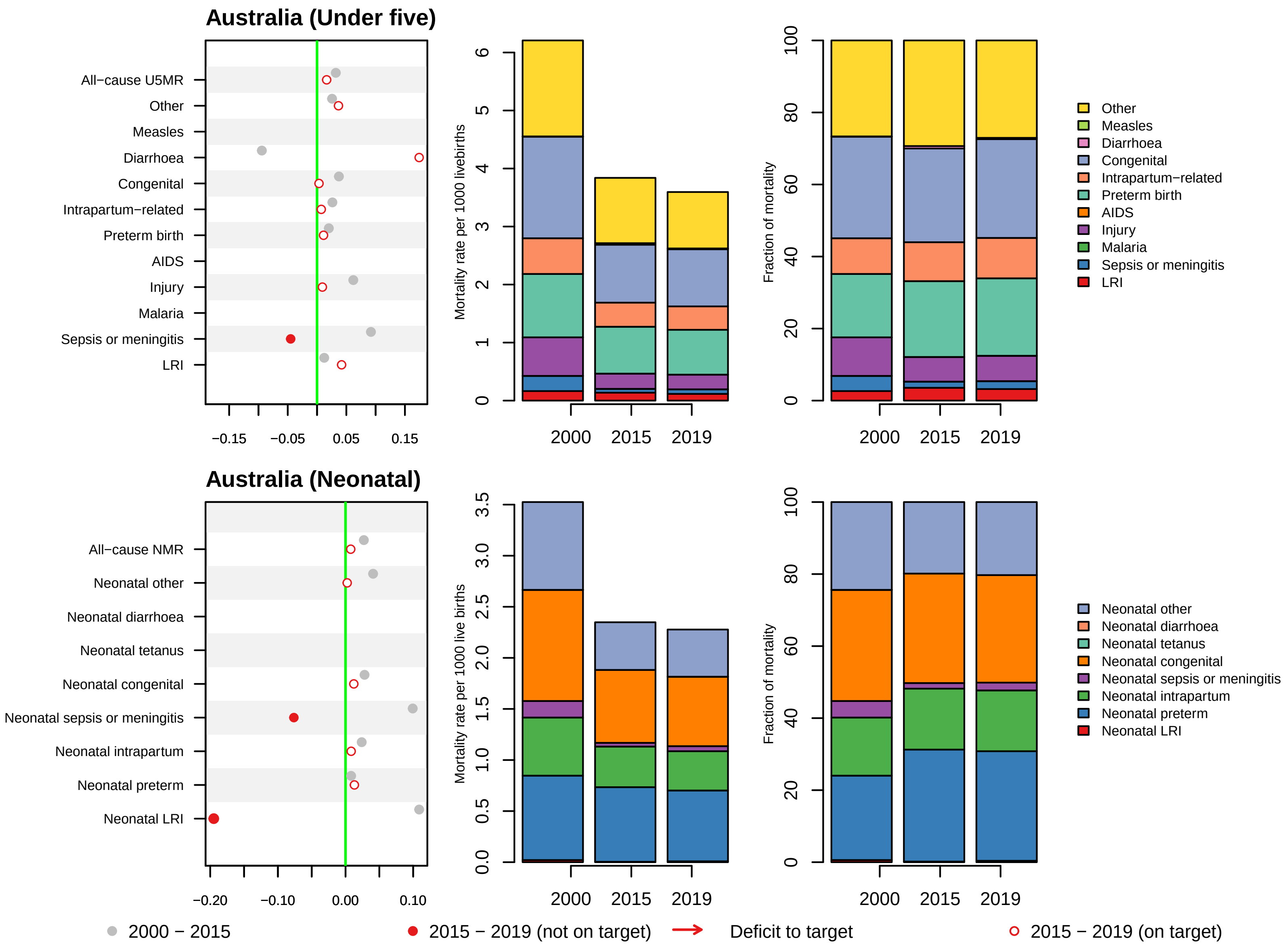
Australia (Under five)
All−cause U5MR
Other
Measles
Diarrhoea
Congenital
Intrapartum−related
Preterm birth
AIDS
Injury
Malaria
Sepsis or meningitis
LRI
−0.15
−0.05
0.05
0.15
Mortality rate per 1000 livebirths
Fraction of mortality
2000
2015
2019
Other
Measles
Diarrhoea
Congenital
Intrapartum−related
Preterm birth
AIDS
Injury
Malaria
Sepsis or meningitis
LRI
Australia (Neonatal)
All−cause NMR
Neonatal other
Neonatal diarrhoea
Neonatal tetanus
Neonatal congenital
Neonatal sepsis or meningitis
Neonatal intrapartum
Neonatal preterm
Neonatal LRI
−0.20
−0.10
0.00
0.10
Mortality rate per 1000 live births
Fraction of mortality
2000
2015
2019
Neonatal other
Neonatal diarrhoea
Neonatal tetanus
Neonatal congenital
Neonatal sepsis or meningitis
Neonatal intrapartum
Neonatal preterm
Neonatal LRI
2000 − 2015
2015 − 2019 (not on target)
Deficit to target
2015 − 2019 (on target)

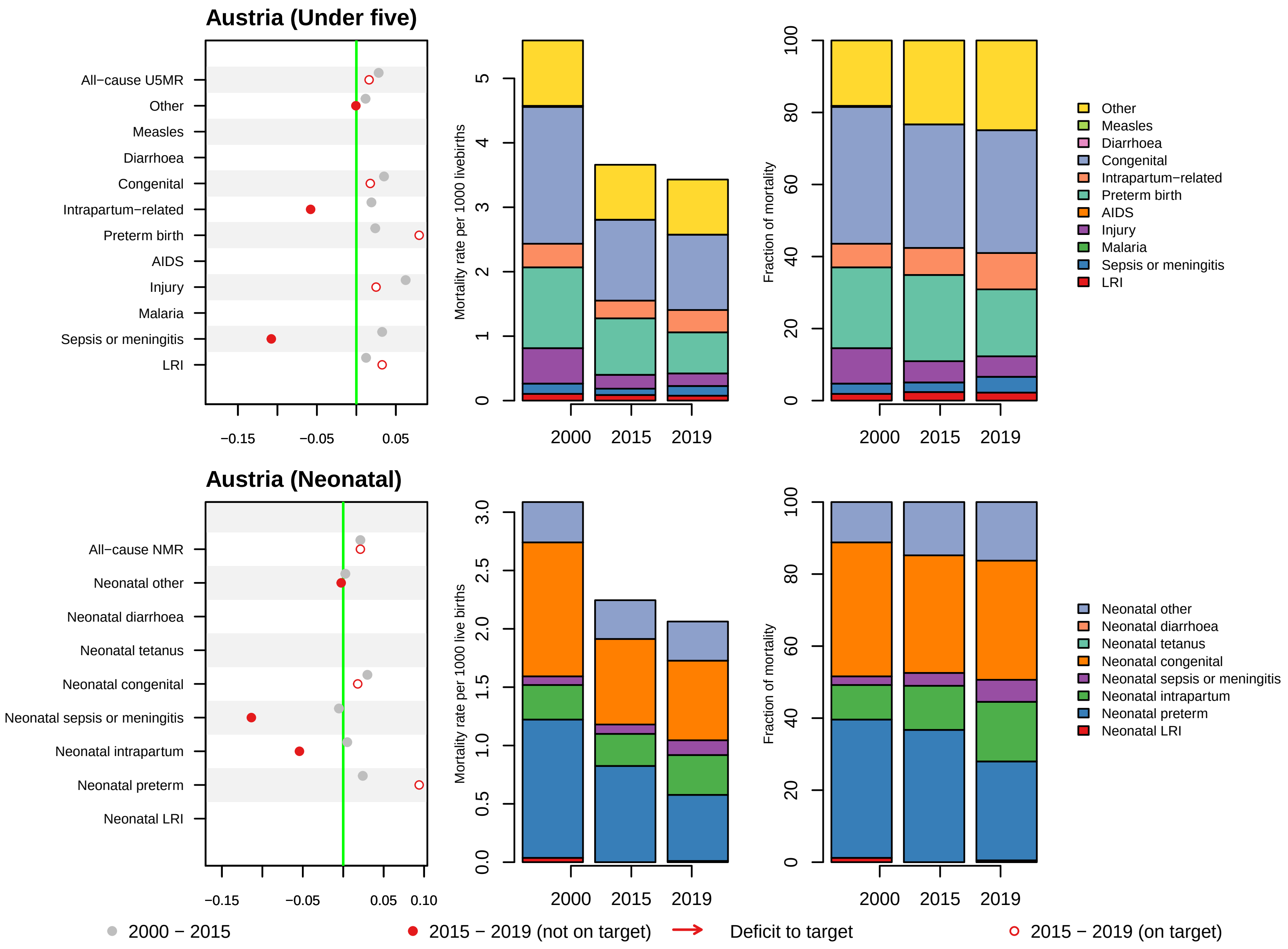

Austria (Under five)
All−cause U5MR
Other
Measles
Diarrhoea
Congenital
Intrapartum−related
Preterm birth
AIDS
Injury
Malaria
Sepsis or meningitis
LRI
−0.15
−0.05
0.05
Mortality rate per 1000 livebirths
0
1
2
3
4
5
2000
2015
2019
Fraction of mortality
0
20
40
60
80
100
Other
Measles
Diarrhoea
Congenital
Intrapartum−related
Preterm birth
AIDS
Injury
Malaria
Sepsis or meningitis
LRI
Austria (Neonatal)
All−cause NMR
Neonatal other
Neonatal diarrhoea
Neonatal tetanus
Neonatal congenital
Neonatal sepsis or meningitis
Neonatal intrapartum
Neonatal preterm
Neonatal LRI
−0.15
−0.05
0.05
0.10
Mortality rate per 1000 live births
0.0
0.5
1.0
1.5
2.0
2.5
3.0
Neonatal other
Neonatal diarrhoea
Neonatal tetanus
Neonatal congenital
Neonatal sepsis or meningitis
Neonatal intrapartum
Neonatal preterm
Neonatal LRI
2000 − 2015
2015 − 2019 (not on target)
Deficit to target
2015 − 2019 (on target)

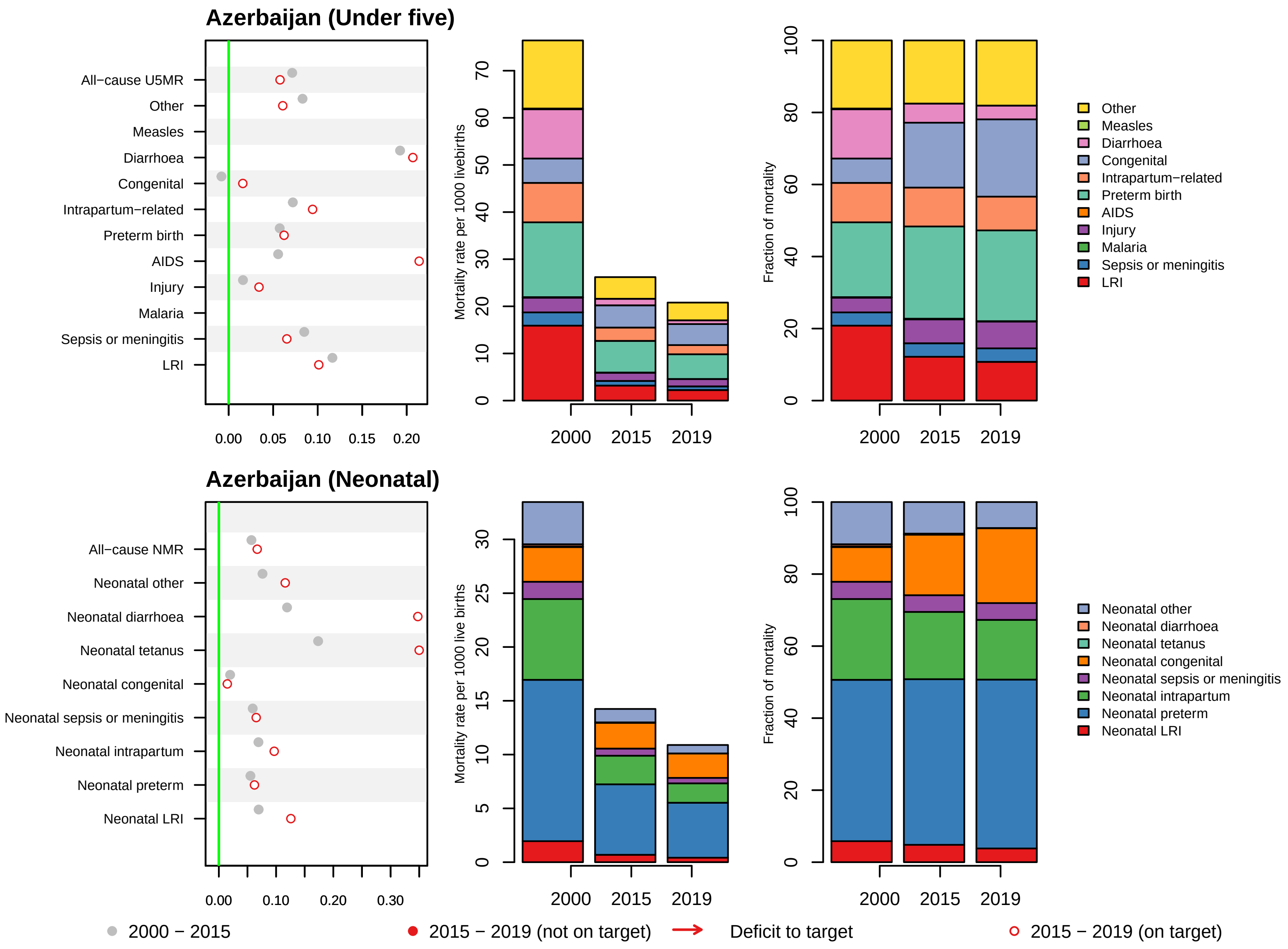
Azerbaijan (Under five)
All−cause U5MR
Other
Measles
Diarrhoea
Congenital
Intrapartum−related
Preterm birth
AIDS
Injury
Malaria
Sepsis or meningitis
LRI
0.00
0.05
0.10
0.15
0.20
Mortality rate per 1000 livebirths
2000
2015
2019
Fraction of mortality
Other
Measles
Diarrhoea
Congenital
Intrapartum−related
Preterm birth
AIDS
Injury
Malaria
Sepsis or meningitis
LRI
Azerbaijan (Neonatal)
All−cause NMR
Neonatal other
Neonatal diarrhoea
Neonatal tetanus
Neonatal congenital
Neonatal sepsis or meningitis
Neonatal intrapartum
Neonatal preterm
Neonatal LRI
0.00
0.10
0.20
0.30
Mortality rate per 1000 live births
Fraction of mortality
Neonatal other
Neonatal diarrhoea
Neonatal tetanus
Neonatal congenital
Neonatal sepsis or meningitis
Neonatal intrapartum
Neonatal preterm
Neonatal LRI
2000 − 2015
2015 − 2019 (not on target)
Deficit to target
2015 − 2019 (on target)

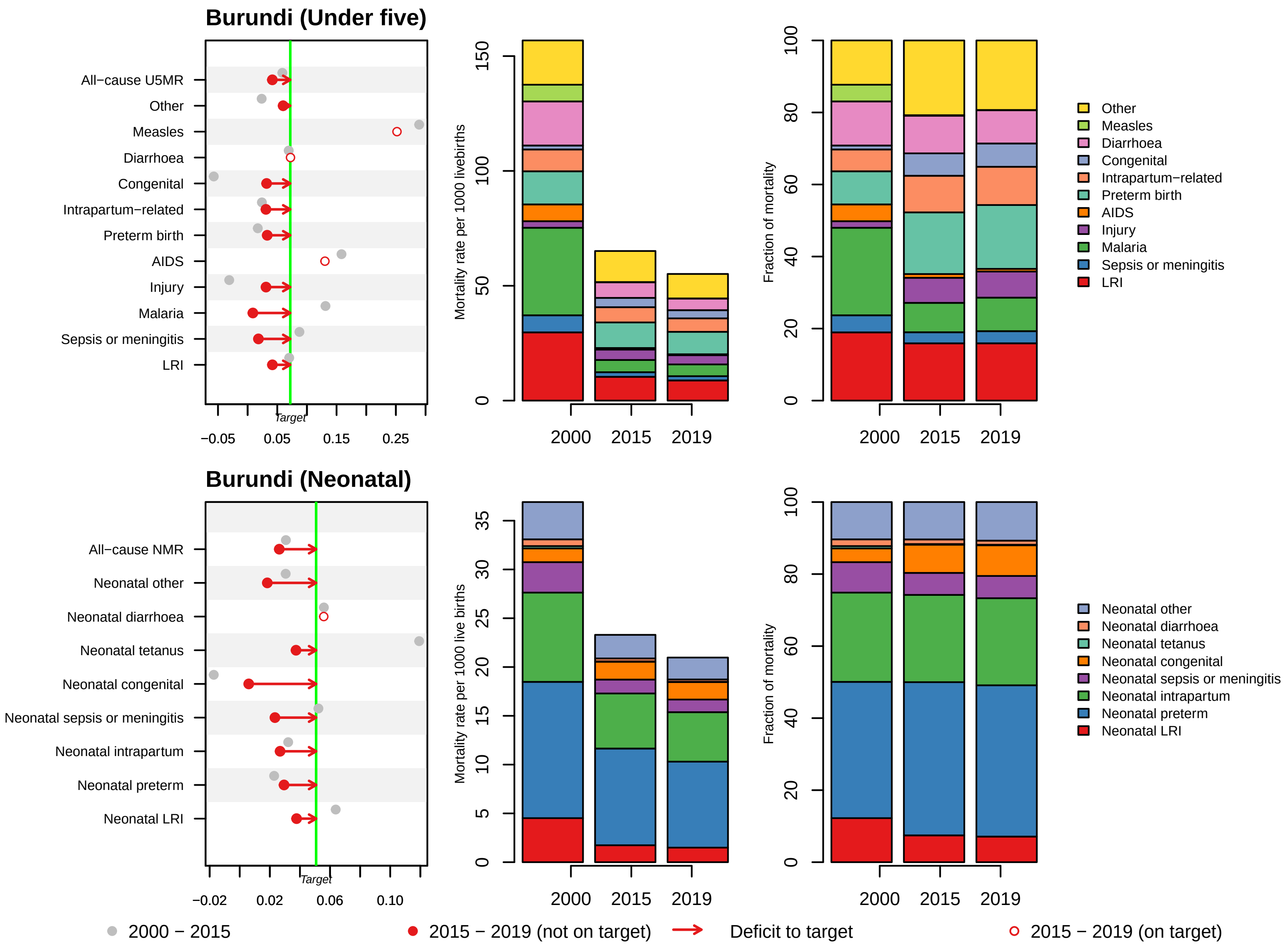

Burundi (Under five)
All−cause U5MR
Other
Measles
Diarrhoea
Congenital
Intrapartum−related
Preterm birth
AIDS
Injury
Malaria
Sepsis or meningitis
LRI
Target
−0.05
0.05
0.15
0.25
Mortality rate per 1000 livebirths
0
50
100
150
2000
2015
2019
Fraction of mortality
0
20
40
60
80
100
Other
Measles
Diarrhoea
Congenital
Intrapartum−related
Preterm birth
AIDS
Injury
Malaria
Sepsis or meningitis
LRI
Burundi (Neonatal)
All−cause NMR
Neonatal other
Neonatal diarrhoea
Neonatal tetanus
Neonatal congenital
Neonatal sepsis or meningitis
Neonatal intrapartum
Neonatal preterm
Neonatal LRI
Target
−0.02
0.02
0.06
0.10
Mortality rate per 1000 live births
0
5
10
15
20
25
30
35
Neonatal other
Neonatal diarrhoea
Neonatal tetanus
Neonatal congenital
Neonatal sepsis or meningitis
Neonatal intrapartum
Neonatal preterm
Neonatal LRI
2000 − 2015
2015 − 2019 (not on target)
Deficit to target
2015 − 2019 (on target)

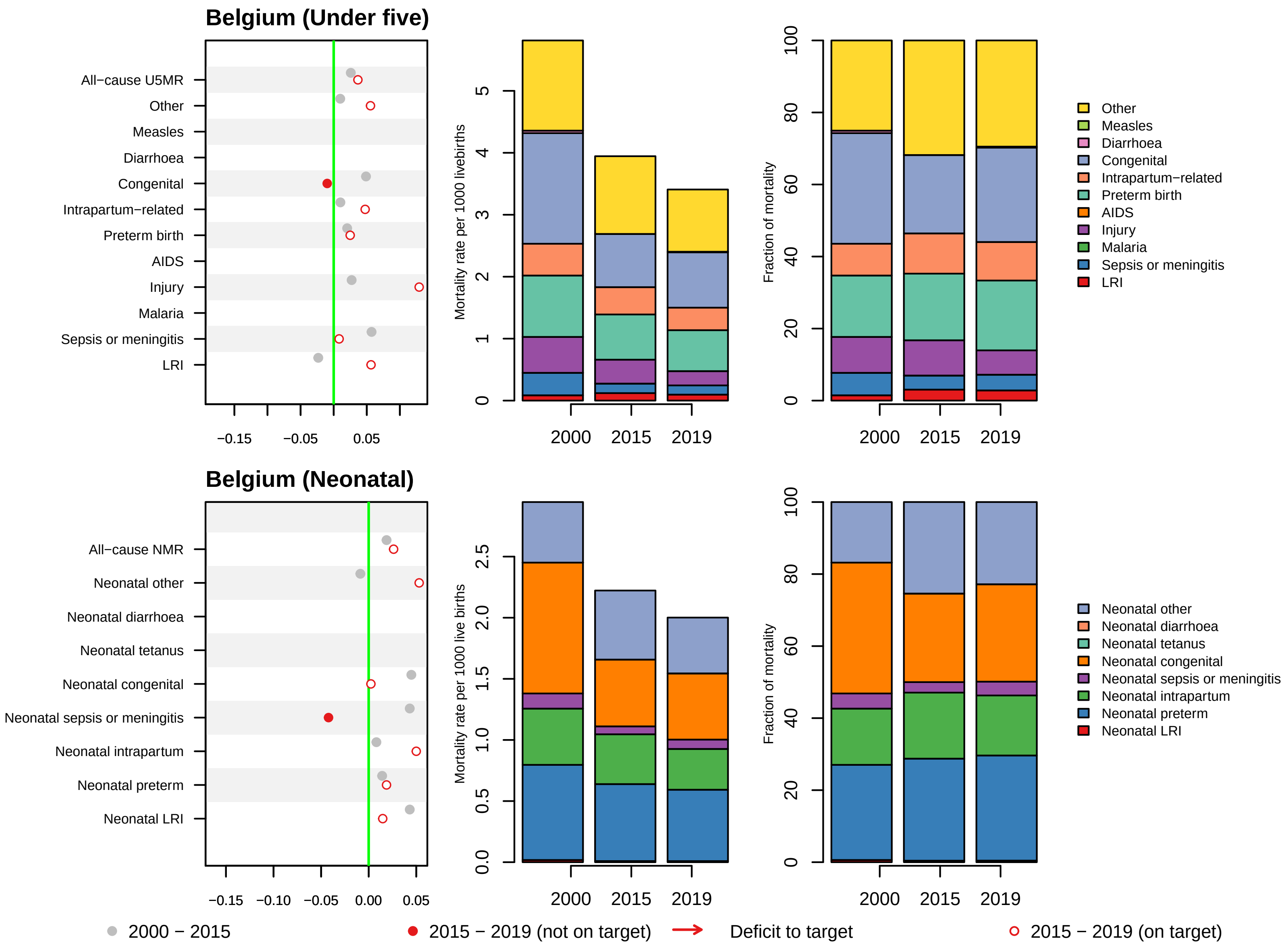

Belgium (Under five)
All−cause U5MR
Other
Measles
Diarrhoea
Congenital
Intrapartum−related
Preterm birth
AIDS
Injury
Malaria
Sepsis or meningitis
LRI
−0.15
−0.05
0.05
Mortality rate per 1000 livebirths
Fraction of mortality
2000
2015
2019
Belgium (Neonatal)
All−cause NMR
Neonatal other
Neonatal diarrhoea
Neonatal tetanus
Neonatal congenital
Neonatal sepsis or meningitis
Neonatal intrapartum
Neonatal preterm
Neonatal LRI
−0.15
−0.10
−0.05
0.00
0.05
Mortality rate per 1000 live births
2000 − 2015
2015 − 2019 (not on target)
Deficit to target
2015 − 2019 (on target)

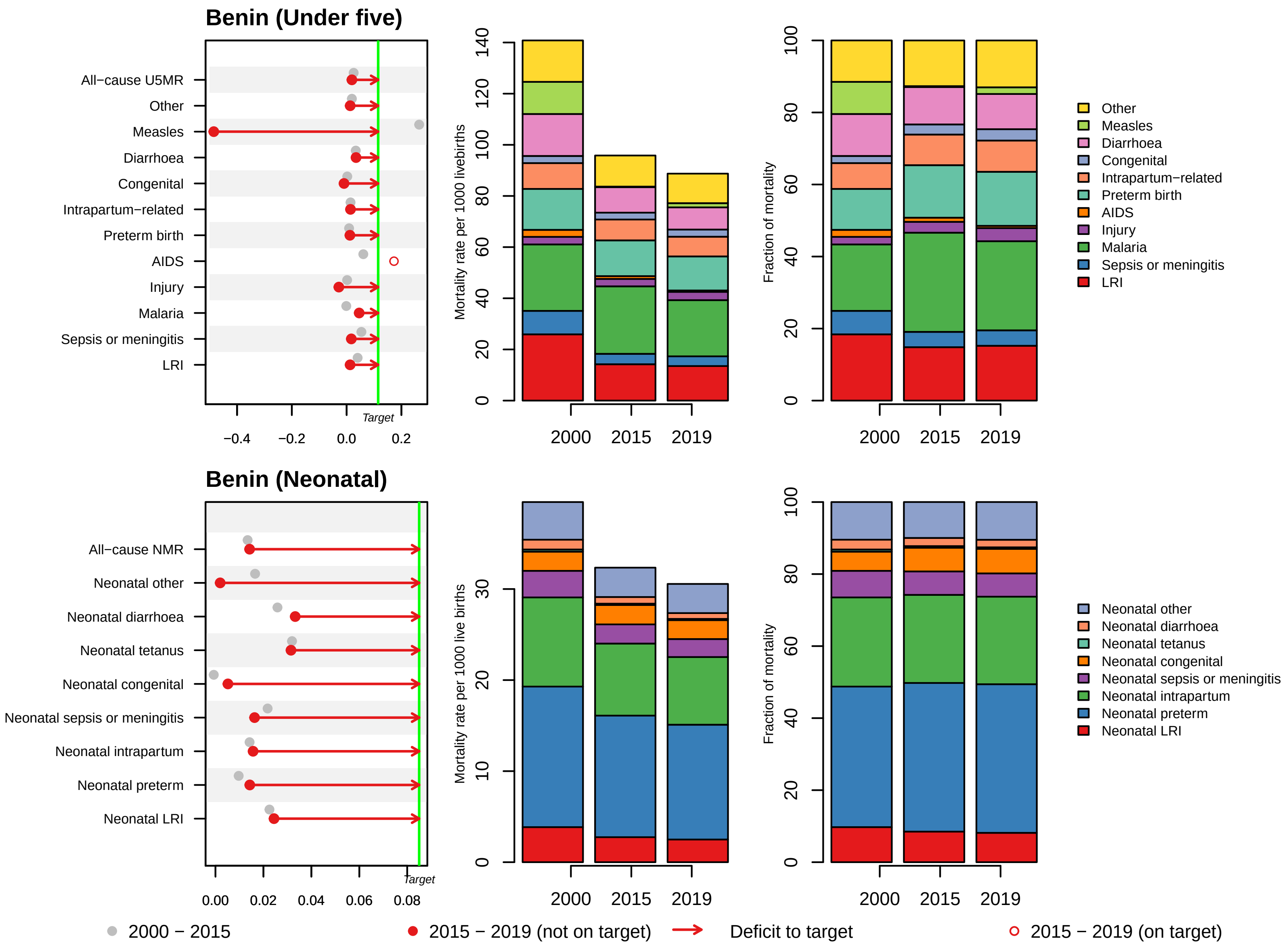

Benin (Under five)
All−cause U5MR
Other
Measles
Diarrhoea
Congenital
Intrapartum−related
Preterm birth
AIDS
Injury
Malaria
Sepsis or meningitis
LRI
Target
−0.4
−0.2
0.0
0.2
Mortality rate per 1000 livebirths
Fraction of mortality
2000
2015
2019
Other
Measles
Diarrhoea
Congenital
Intrapartum−related
Preterm birth
AIDS
Injury
Malaria
Sepsis or meningitis
LRI
Benin (Neonatal)
All−cause NMR
Neonatal other
Neonatal diarrhoea
Neonatal tetanus
Neonatal congenital
Neonatal sepsis or meningitis
Neonatal intrapartum
Neonatal preterm
Neonatal LRI
0.00
0.02
0.04
0.06
0.08
Mortality rate per 1000 live births
Neonatal other
Neonatal diarrhoea
Neonatal tetanus
Neonatal congenital
Neonatal sepsis or meningitis
Neonatal intrapartum
Neonatal preterm
Neonatal LRI
2000 − 2015
2015 − 2019 (not on target)
Deficit to target
2015 − 2019 (on target)

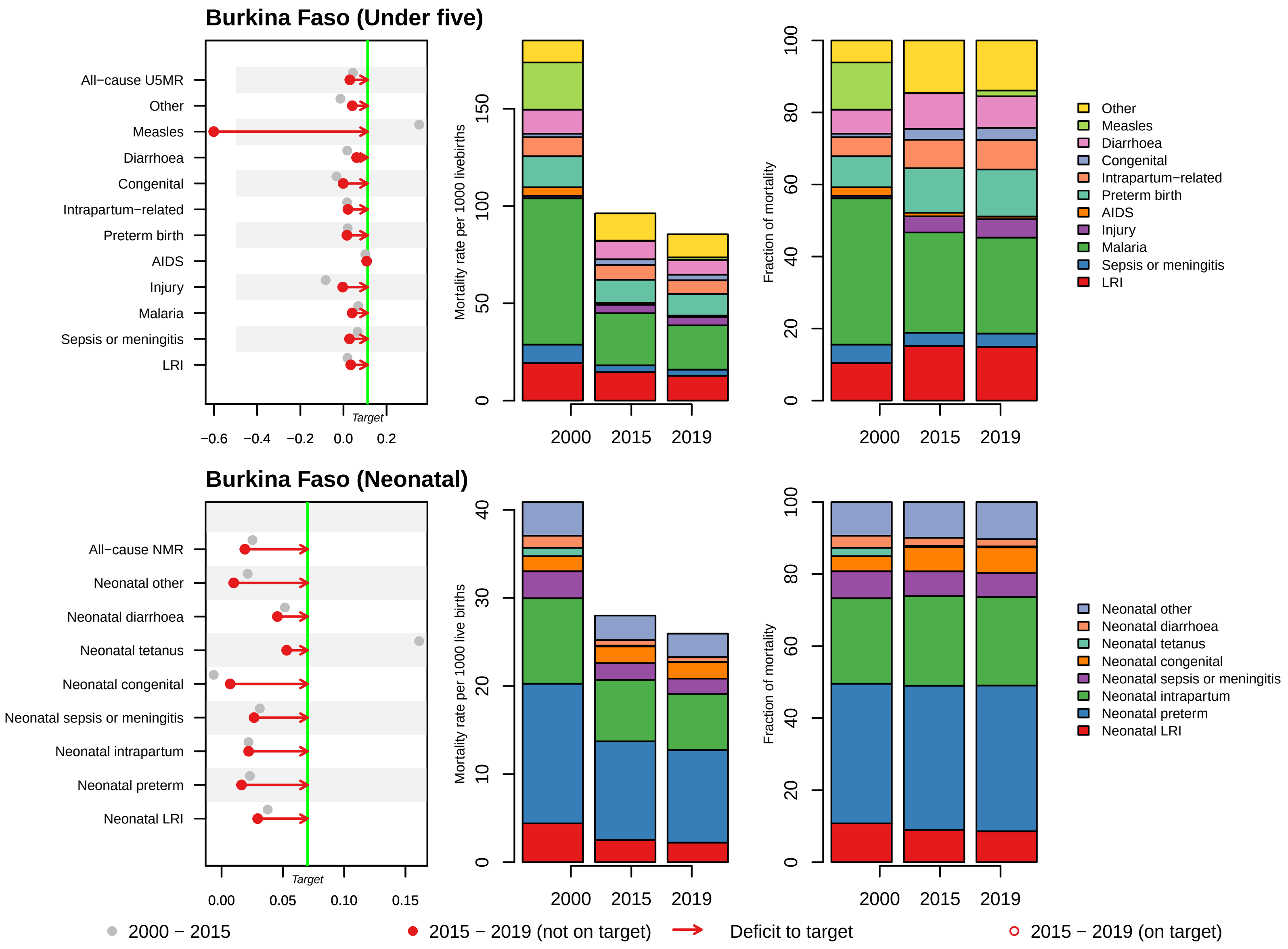
Burkina Faso (Under five)
All−cause U5MR
Other
Measles
Diarrhoea
Congenital
Intrapartum−related
Preterm birth
AIDS
Injury
Malaria
Sepsis or meningitis
LRI
Target
−0.6 −0.4 −0.2 0.0 0.2
Mortality rate per 1000 livebirths
0 50 100 150
2000 2015 2019
Fraction of mortality
0 20 40 60 80 100
2000 2015 2019
Other
Measles
Diarrhoea
Congenital
Intrapartum−related
Preterm birth
AIDS
Injury
Malaria
Sepsis or meningitis
LRI
Burkina Faso (Neonatal)
All−cause NMR
Neonatal other
Neonatal diarrhoea
Neonatal tetanus
Neonatal congenital
Neonatal sepsis or meningitis
Neonatal intrapartum
Neonatal preterm
Neonatal LRI
Target
0.00 0.05 0.10 0.15
Mortality rate per 1000 live births
0 10 20 30 40
2000 2015 2019
Fraction of mortality
0 20 40 60 80 100
2000 2015 2019
Neonatal other
Neonatal diarrhoea
Neonatal tetanus
Neonatal congenital
Neonatal sepsis or meningitis
Neonatal intrapartum
Neonatal preterm
Neonatal LRI
2000 − 2015
2015 − 2019 (not on target)
Deficit to target
2015 − 2019 (on target)

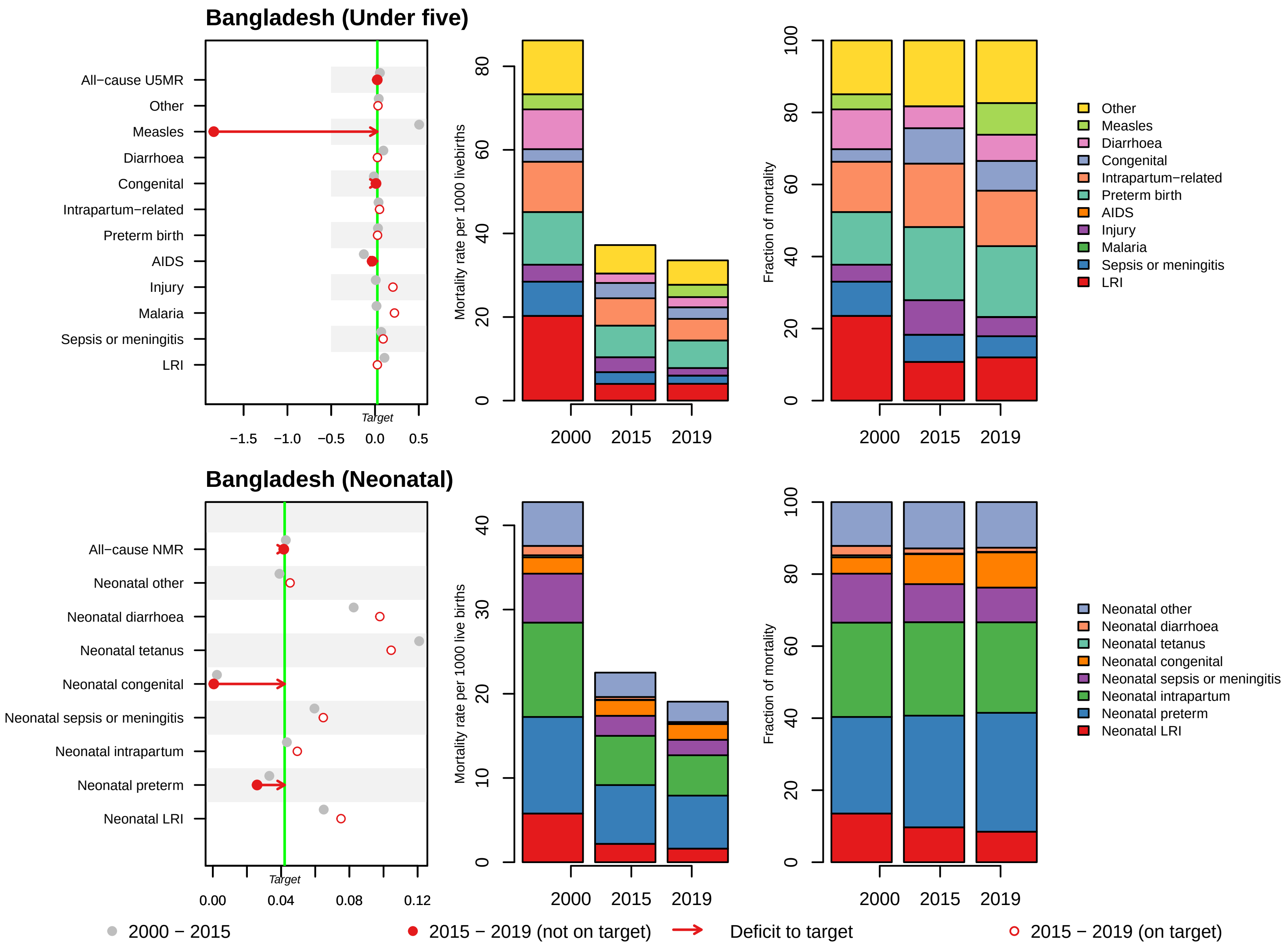

Bangladesh (Under five)
All−cause U5MR
Other
Measles
Diarrhoea
Congenital
Intrapartum−related
Preterm birth
AIDS
Injury
Malaria
Sepsis or meningitis
LRI
Target
−1.5
−1.0
−0.5
0.0
0.5
Mortality rate per 1000 livebirths
0
20
40
60
80
2000
2015
2019
Fraction of mortality
0
20
40
60
80
100
2000
2015
2019
Other
Measles
Diarrhoea
Congenital
Intrapartum−related
Preterm birth
AIDS
Injury
Malaria
Sepsis or meningitis
LRI
Bangladesh (Neonatal)
All−cause NMR
Neonatal other
Neonatal diarrhoea
Neonatal tetanus
Neonatal congenital
Neonatal sepsis or meningitis
Neonatal intrapartum
Neonatal preterm
Neonatal LRI
Target
0.00
0.04
0.08
0.12
Mortality rate per 1000 live births
0
10
20
30
40
2000
2015
2019
Fraction of mortality
0
20
40
60
80
100
2000
2015
2019
Neonatal other
Neonatal diarrhoea
Neonatal tetanus
Neonatal congenital
Neonatal sepsis or meningitis
Neonatal intrapartum
Neonatal preterm
Neonatal LRI
2000 − 2015
2015 − 2019 (not on target)
Deficit to target
2015 − 2019 (on target)

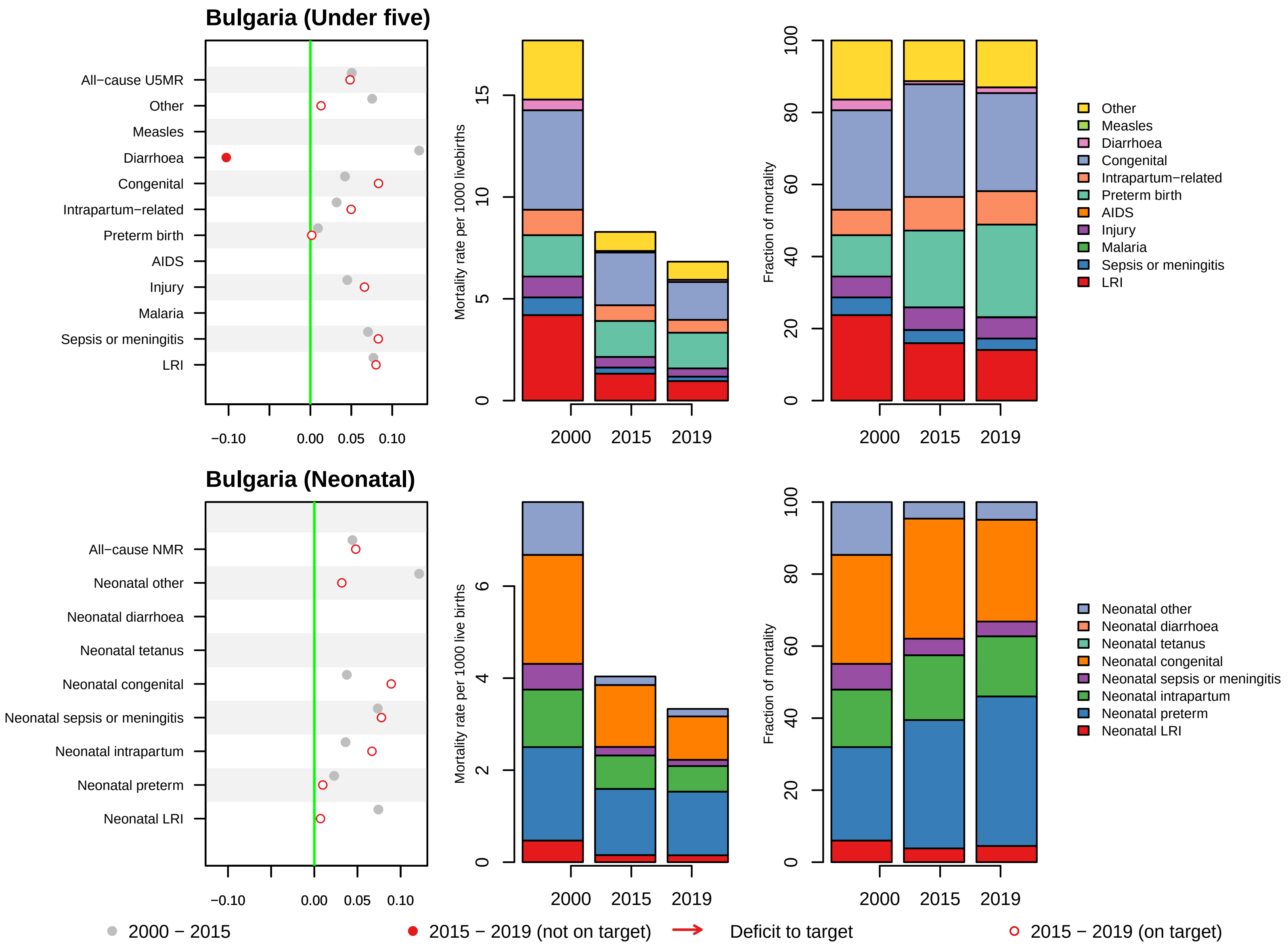
Bulgaria (Under five)
All−cause U5MR
Other
Measles
Diarrhoea
Congenital
Intrapartum−related
Preterm birth
AIDS
Injury
Malaria
Sepsis or meningitis
LRI
−0.10
0.00
0.05
0.10
Mortality rate per 1000 livebirths
0
5
10
15
2000
2015
2019
Fraction of mortality
0
20
40
60
80
100
Other
Measles
Diarrhoea
Congenital
Intrapartum−related
Preterm birth
AIDS
Injury
Malaria
Sepsis or meningitis
LRI
Bulgaria (Neonatal)
All−cause NMR
Neonatal other
Neonatal diarrhoea
Neonatal tetanus
Neonatal congenital
Neonatal sepsis or meningitis
Neonatal intrapartum
Neonatal preterm
Neonatal LRI
Mortality rate per 1000 live births
0
2
4
6
Neonatal other
Neonatal diarrhoea
Neonatal tetanus
Neonatal congenital
Neonatal sepsis or meningitis
Neonatal intrapartum
Neonatal preterm
Neonatal LRI
2000 − 2015
2015 − 2019 (not on target)
Deficit to target
2015 − 2019 (on target)

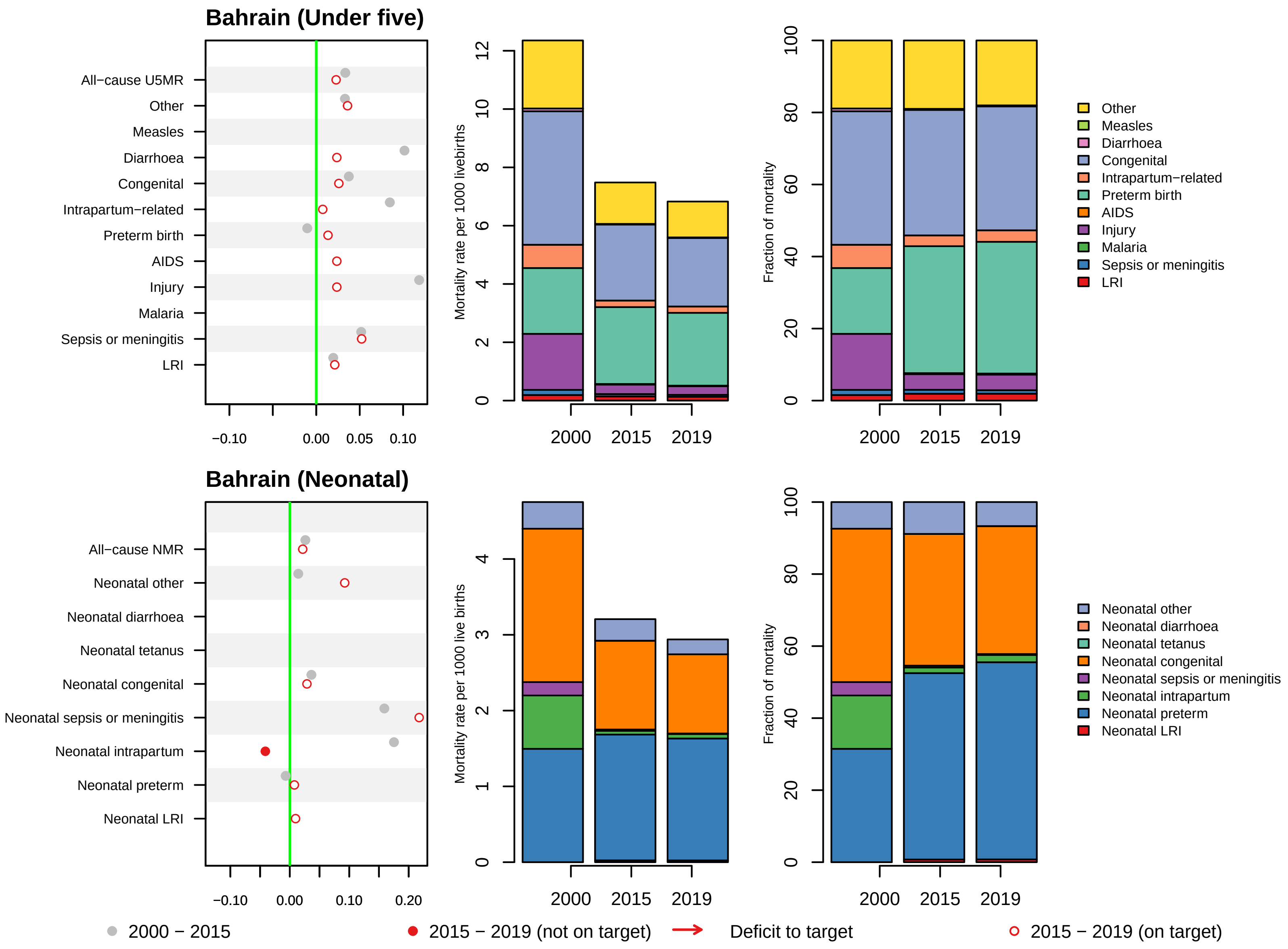

Bahrain (Under five)
All−cause U5MR
Other
Measles
Diarrhoea
Congenital
Intrapartum−related
Preterm birth
AIDS
Injury
Malaria
Sepsis or meningitis
LRI
−0.10
0.00
0.05
0.10
Mortality rate per 1000 livebirths
0
2
4
6
8
10
12
2000
2015
2019
Fraction of mortality
0
20
40
60
80
100
2000
2015
2019
Other
Measles
Diarrhoea
Congenital
Intrapartum−related
Preterm birth
AIDS
Injury
Malaria
Sepsis or meningitis
LRI
Bahrain (Neonatal)
All−cause NMR
Neonatal other
Neonatal diarrhoea
Neonatal tetanus
Neonatal congenital
Neonatal sepsis or meningitis
Neonatal intrapartum
Neonatal preterm
Neonatal LRI
−0.10
0.00
0.10
0.20
Mortality rate per 1000 live births
0
1
2
3
4
2000
2015
2019
Fraction of mortality
0
20
40
60
80
100
2000
2015
2019
Neonatal other
Neonatal diarrhoea
Neonatal tetanus
Neonatal congenital
Neonatal sepsis or meningitis
Neonatal intrapartum
Neonatal preterm
Neonatal LRI
2000 − 2015
2015 − 2019 (not on target)
Deficit to target
2015 − 2019 (on target)

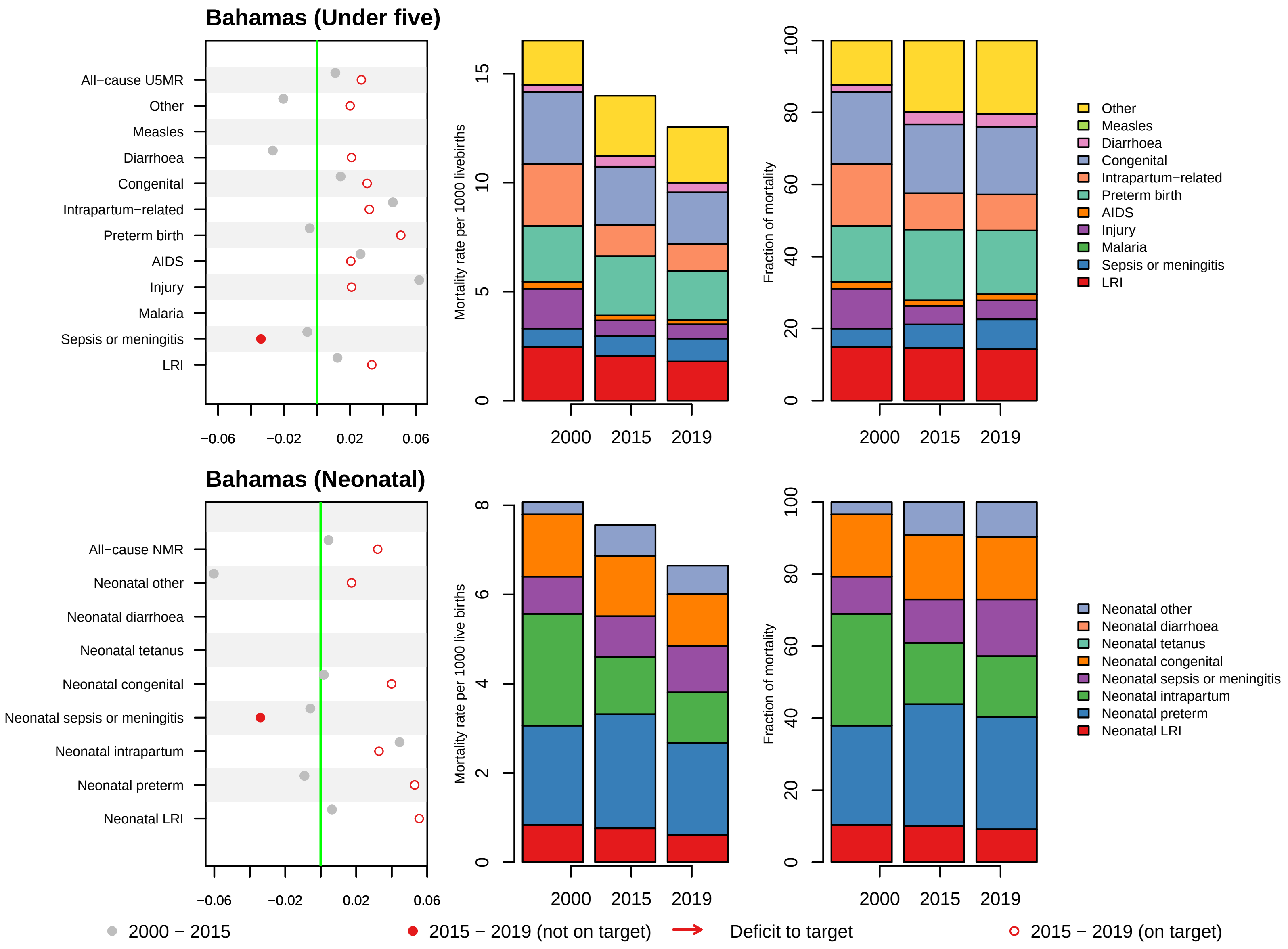

Bahamas (Under five)
All−cause U5MR
Other
Measles
Diarrhoea
Congenital
Intrapartum−related
Preterm birth
AIDS
Injury
Malaria
Sepsis or meningitis
LRI
−0.06 −0.02 0.02 0.06
Mortality rate per 1000 livebirths
Fraction of mortality
2000 2015 2019
Other
Measles
Diarrhoea
Congenital
Intrapartum−related
Preterm birth
AIDS
Injury
Malaria
Sepsis or meningitis
LRI
Bahamas (Neonatal)
All−cause NMR
Neonatal other
Neonatal diarrhoea
Neonatal tetanus
Neonatal congenital
Neonatal sepsis or meningitis
Neonatal intrapartum
Neonatal preterm
Neonatal LRI
Mortality rate per 1000 live births
Neonatal other
Neonatal diarrhoea
Neonatal tetanus
Neonatal congenital
Neonatal sepsis or meningitis
Neonatal intrapartum
Neonatal preterm
Neonatal LRI
2000 − 2015
2015 − 2019 (not on target)
Deficit to target
2015 − 2019 (on target)

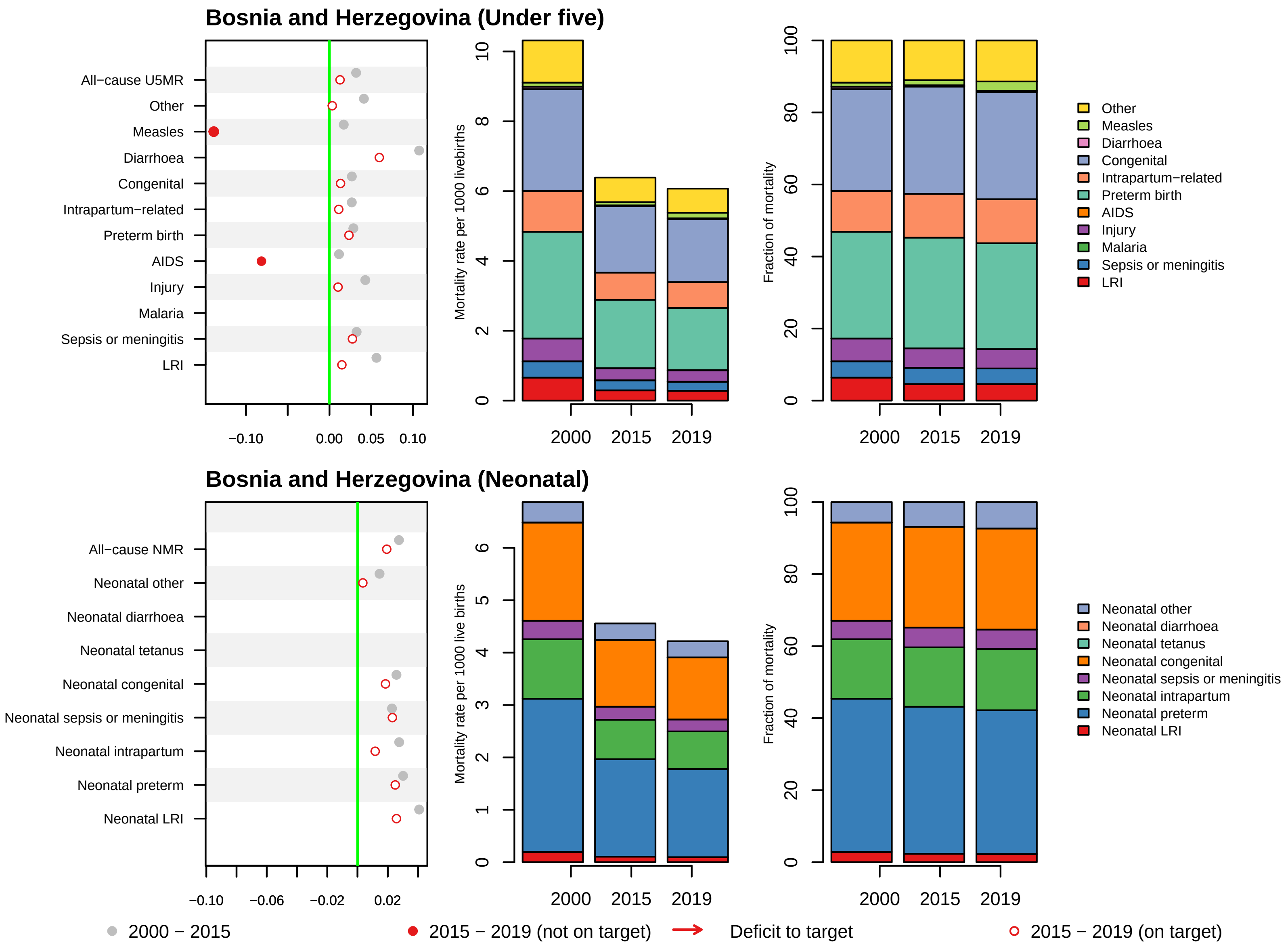
Bosnia and Herzegovina (Under five)
All−cause U5MR
Other
Measles
Diarrhoea
Congenital
Intrapartum−related
Preterm birth
AIDS
Injury
Malaria
Sepsis or meningitis
LRI
−0.10
0.00
0.05
0.10
Mortality rate per 1000 livebirths
Fraction of mortality
2000
2015
2019
Other
Measles
Diarrhoea
Congenital
Intrapartum−related
Preterm birth
AIDS
Injury
Malaria
Sepsis or meningitis
LRI
Bosnia and Herzegovina (Neonatal)
All−cause NMR
Neonatal other
Neonatal diarrhoea
Neonatal tetanus
Neonatal congenital
Neonatal sepsis or meningitis
Neonatal intrapartum
Neonatal preterm
Neonatal LRI
−0.10
−0.06
−0.02
0.02
Mortality rate per 1000 live births
Fraction of mortality
2000
2015
2019
Neonatal other
Neonatal diarrhoea
Neonatal tetanus
Neonatal congenital
Neonatal sepsis or meningitis
Neonatal intrapartum
Neonatal preterm
Neonatal LRI
2000 − 2015
2015 − 2019 (not on target)
Deficit to target
2015 − 2019 (on target)

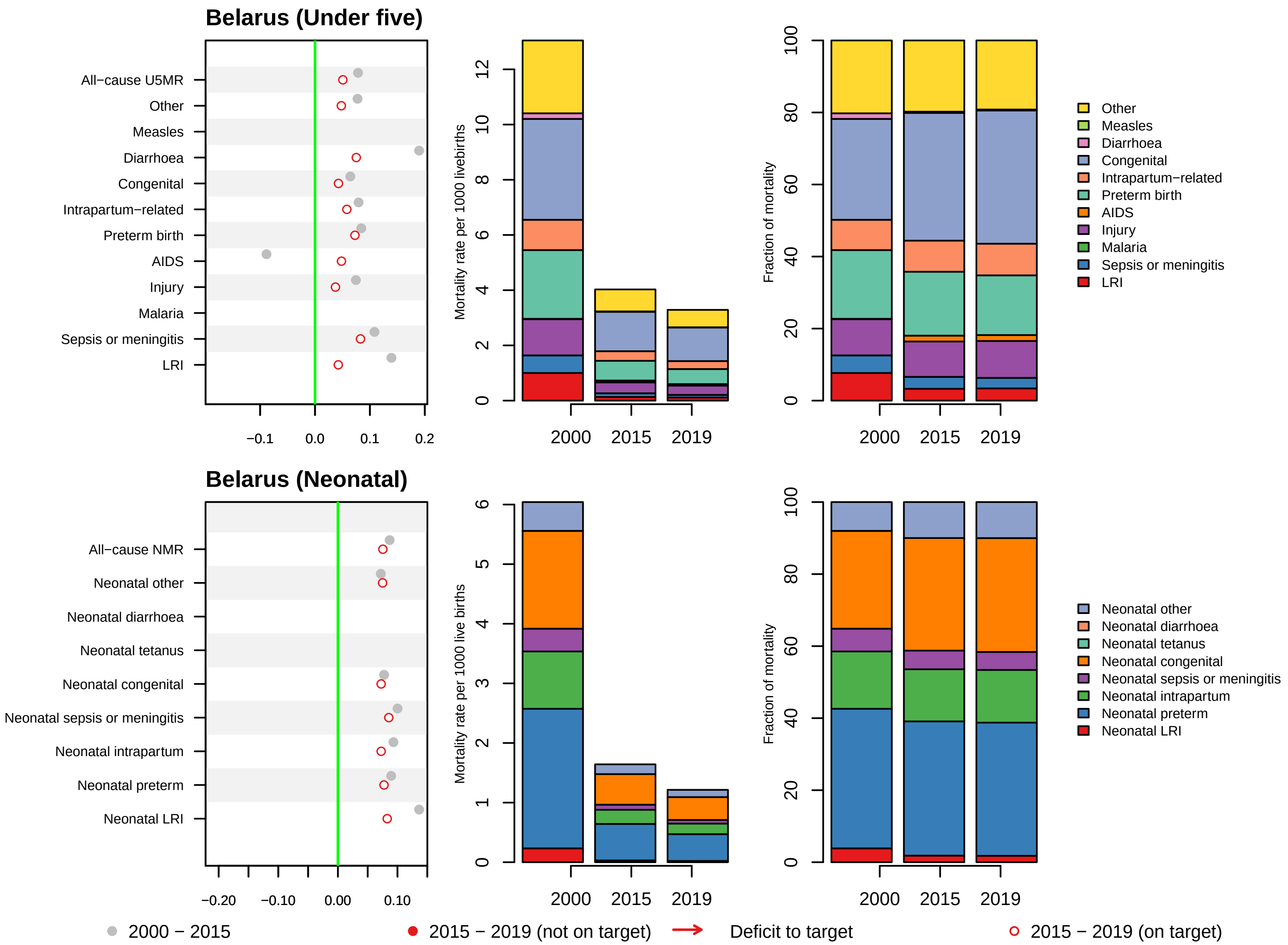
Belarus (Under five)
All−cause U5MR
Other
Measles
Diarrhoea
Congenital
Intrapartum−related
Preterm birth
AIDS
Injury
Malaria
Sepsis or meningitis
LRI
−0.1
0.0
0.1
0.2
Mortality rate per 1000 livebirths
Fraction of mortality
2000
2015
2019
Belarus (Neonatal)
All−cause NMR
Neonatal other
Neonatal diarrhoea
Neonatal tetanus
Neonatal congenital
Neonatal sepsis or meningitis
Neonatal intrapartum
Neonatal preterm
Neonatal LRI
−0.20
−0.10
0.00
0.10
Mortality rate per 1000 live births
2000 − 2015
2015 − 2019 (not on target)
Deficit to target
2015 − 2019 (on target)

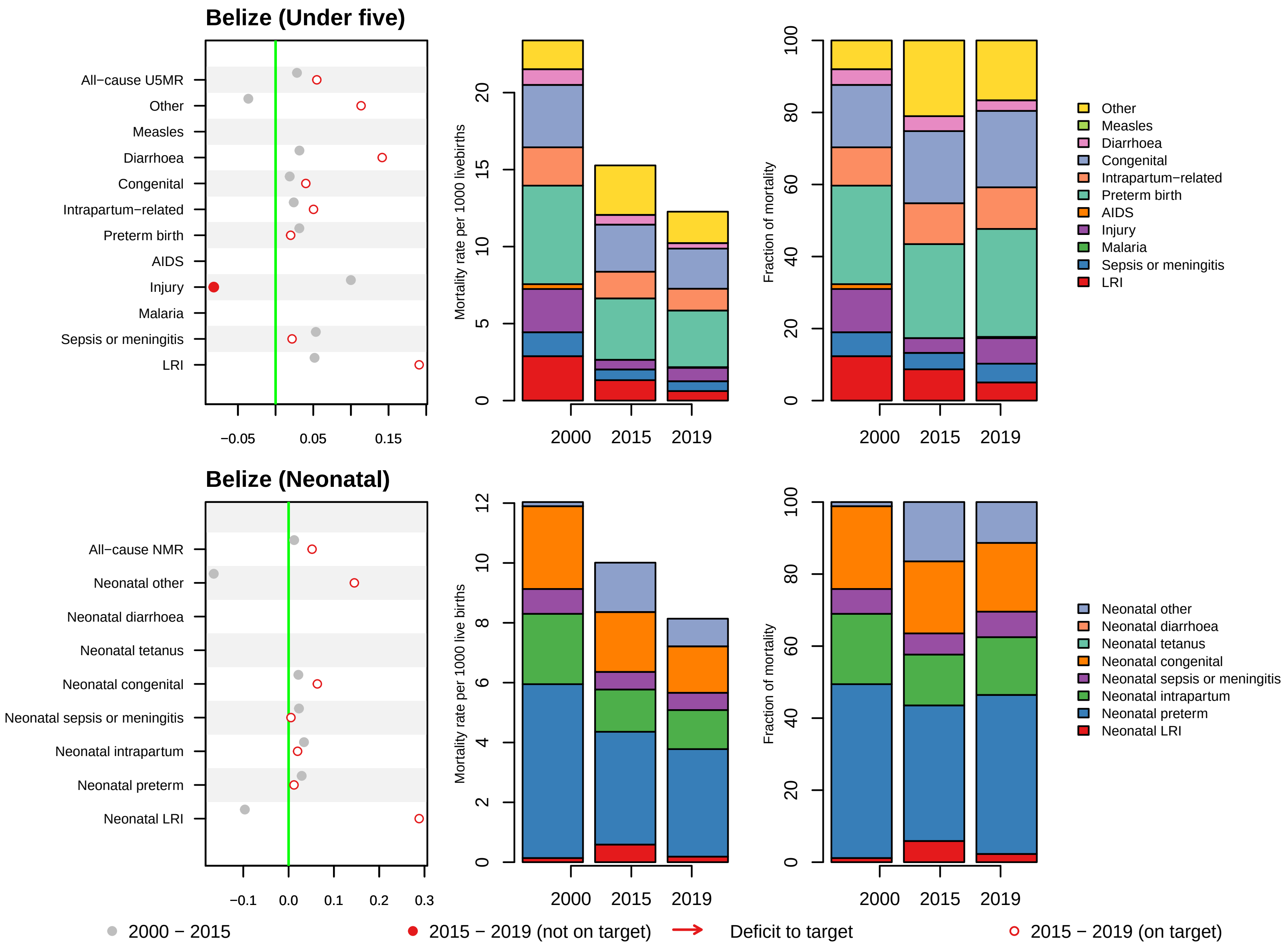

Belize (Under five)
All−cause U5MR
Other
Measles
Diarrhoea
Congenital
Intrapartum−related
Preterm birth
AIDS
Injury
Malaria
Sepsis or meningitis
LRI
−0.05
0.05
0.15
Mortality rate per 1000 livebirths
Fraction of mortality
2000
2015
2019
Other
Measles
Diarrhoea
Congenital
Intrapartum−related
Preterm birth
AIDS
Injury
Malaria
Sepsis or meningitis
LRI
Belize (Neonatal)
All−cause NMR
Neonatal other
Neonatal diarrhoea
Neonatal tetanus
Neonatal congenital
Neonatal sepsis or meningitis
Neonatal intrapartum
Neonatal preterm
Neonatal LRI
−0.1
0.0
0.1
0.2
0.3
Mortality rate per 1000 live births
Fraction of mortality
Neonatal other
Neonatal diarrhoea
Neonatal tetanus
Neonatal congenital
Neonatal sepsis or meningitis
Neonatal intrapartum
Neonatal preterm
Neonatal LRI
2000 − 2015
2015 − 2019 (not on target)
Deficit to target
2015 − 2019 (on target)

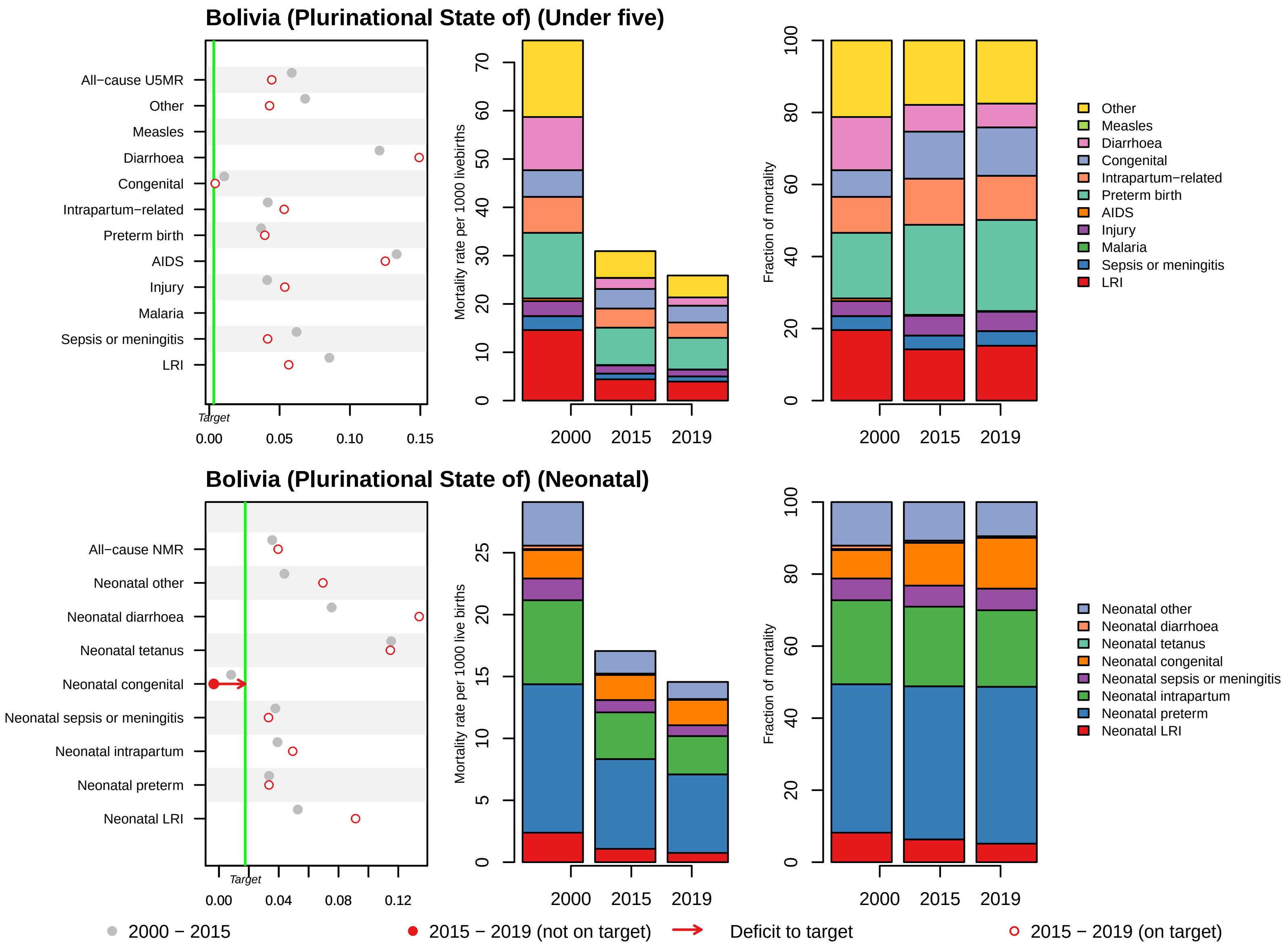

Bolivia (Plurinational State of) (Under five)
All−cause U5MR
Other
Measles
Diarrhoea
Congenital
Intrapartum−related
Preterm birth
AIDS
Injury
Malaria
Sepsis or meningitis
LRI
Target
0.00
0.05
0.10
0.15
Mortality rate per 1000 livebirths
Fraction of mortality
2000
2015
2019
Other
Measles
Diarrhoea
Congenital
Intrapartum−related
Preterm birth
AIDS
Injury
Malaria
Sepsis or meningitis
LRI
Bolivia (Plurinational State of) (Neonatal)
All−cause NMR
Neonatal other
Neonatal diarrhoea
Neonatal tetanus
Neonatal congenital
Neonatal sepsis or meningitis
Neonatal intrapartum
Neonatal preterm
Neonatal LRI
Target
0.00
0.04
0.08
0.12
Mortality rate per 1000 live births
Fraction of mortality
2000
2015
2019
Neonatal other
Neonatal diarrhoea
Neonatal tetanus
Neonatal congenital
Neonatal sepsis or meningitis
Neonatal intrapartum
Neonatal preterm
Neonatal LRI
2000 − 2015
2015 − 2019 (not on target)
Deficit to target
2015 − 2019 (on target)

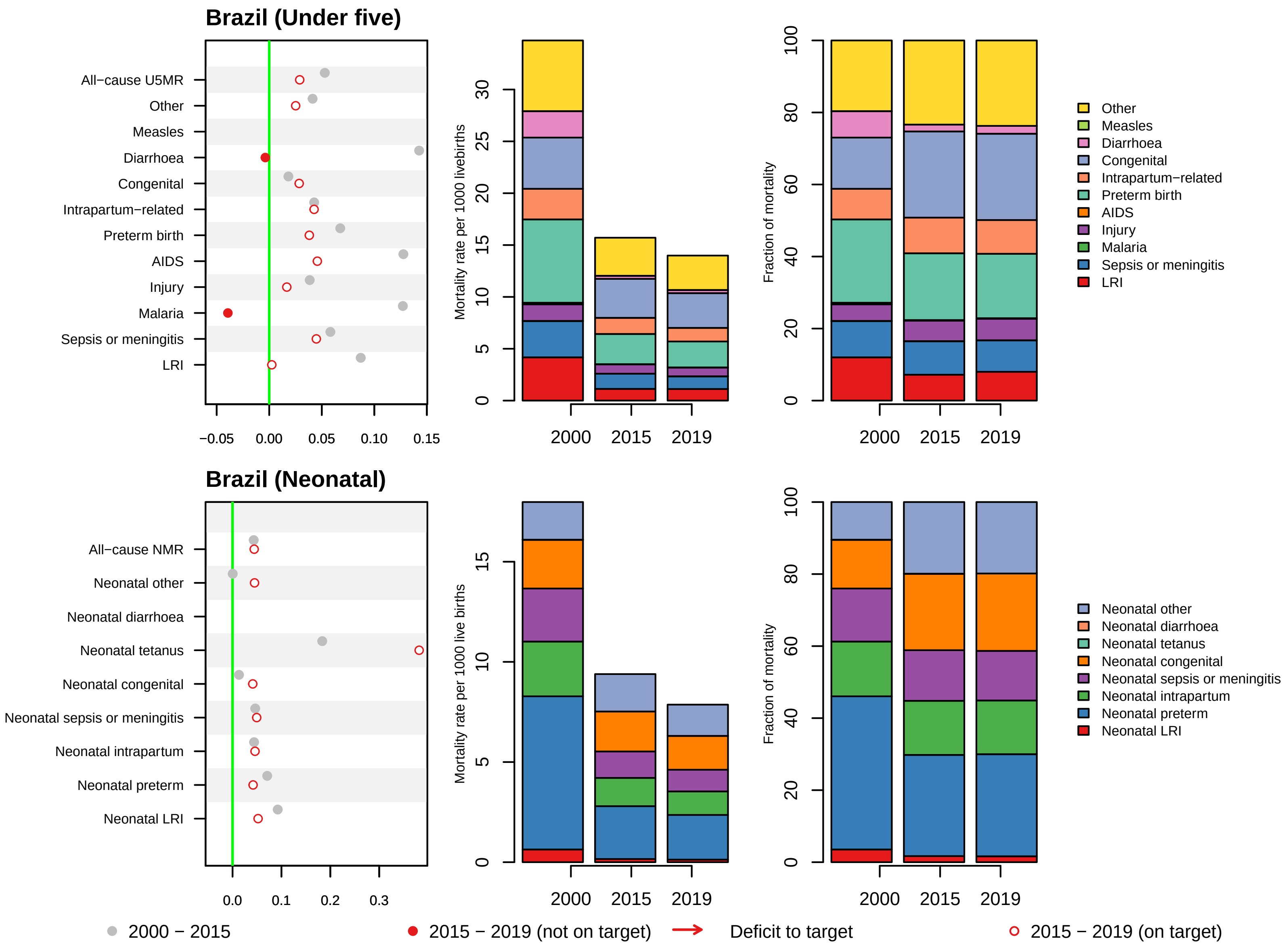

Brazil (Under five)
All−cause U5MR
Other
Measles
Diarrhoea
Congenital
Intrapartum−related
Preterm birth
AIDS
Injury
Malaria
Sepsis or meningitis
LRI
−0.05
0.00
0.05
0.10
0.15
Mortality rate per 1000 livebirths
2000
2015
2019
Fraction of mortality
Other
Measles
Diarrhoea
Congenital
Intrapartum−related
Preterm birth
AIDS
Injury
Malaria
Sepsis or meningitis
LRI
Brazil (Neonatal)
All−cause NMR
Neonatal other
Neonatal diarrhoea
Neonatal tetanus
Neonatal congenital
Neonatal sepsis or meningitis
Neonatal intrapartum
Neonatal preterm
Neonatal LRI
0.0
0.1
0.2
0.3
Mortality rate per 1000 live births
Fraction of mortality
Neonatal other
Neonatal diarrhoea
Neonatal tetanus
Neonatal congenital
Neonatal sepsis or meningitis
Neonatal intrapartum
Neonatal preterm
Neonatal LRI
2000 − 2015
2015 − 2019 (not on target)
Deficit to target
2015 − 2019 (on target)

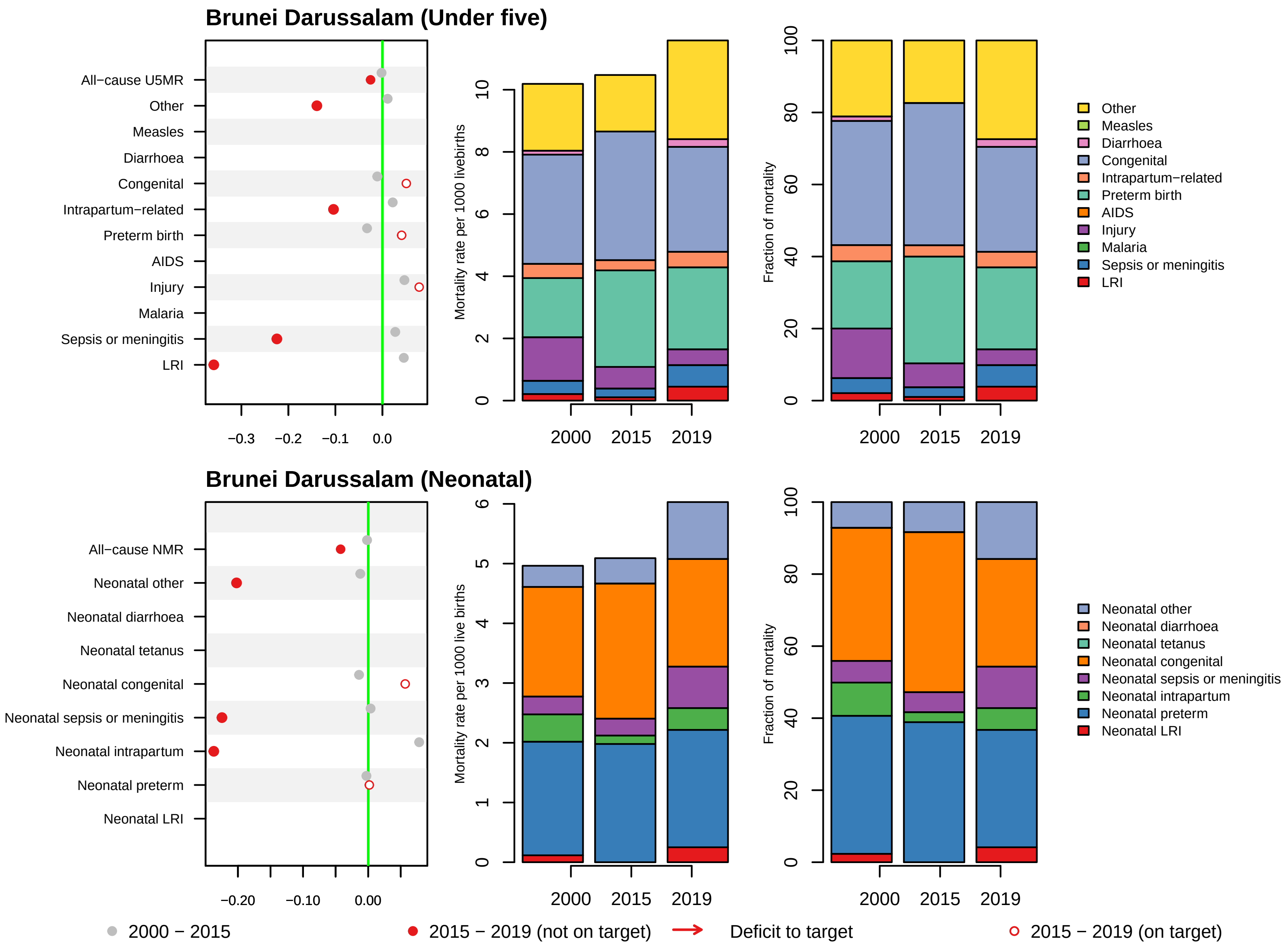

Brunei Darussalam (Under five)
All−cause U5MR
Other
Measles
Diarrhoea
Congenital
Intrapartum−related
Preterm birth
AIDS
Injury
Malaria
Sepsis or meningitis
LRI
−0.3
−0.2
−0.1
0.0
Mortality rate per 1000 livebirths
0
2
4
6
8
10
2000
2015
2019
Fraction of mortality
0
20
40
60
80
100
Other
Measles
Diarrhoea
Congenital
Intrapartum−related
Preterm birth
AIDS
Injury
Malaria
Sepsis or meningitis
LRI
Brunei Darussalam (Neonatal)
All−cause NMR
Neonatal other
Neonatal diarrhoea
Neonatal tetanus
Neonatal congenital
Neonatal sepsis or meningitis
Neonatal intrapartum
Neonatal preterm
Neonatal LRI
−0.20
−0.10
0.00
Mortality rate per 1000 live births
0
1
2
3
4
5
6
Neonatal other
Neonatal diarrhoea
Neonatal tetanus
Neonatal congenital
Neonatal sepsis or meningitis
Neonatal intrapartum
Neonatal preterm
Neonatal LRI
2000 − 2015
2015 − 2019 (not on target)
Deficit to target
2015 − 2019 (on target)

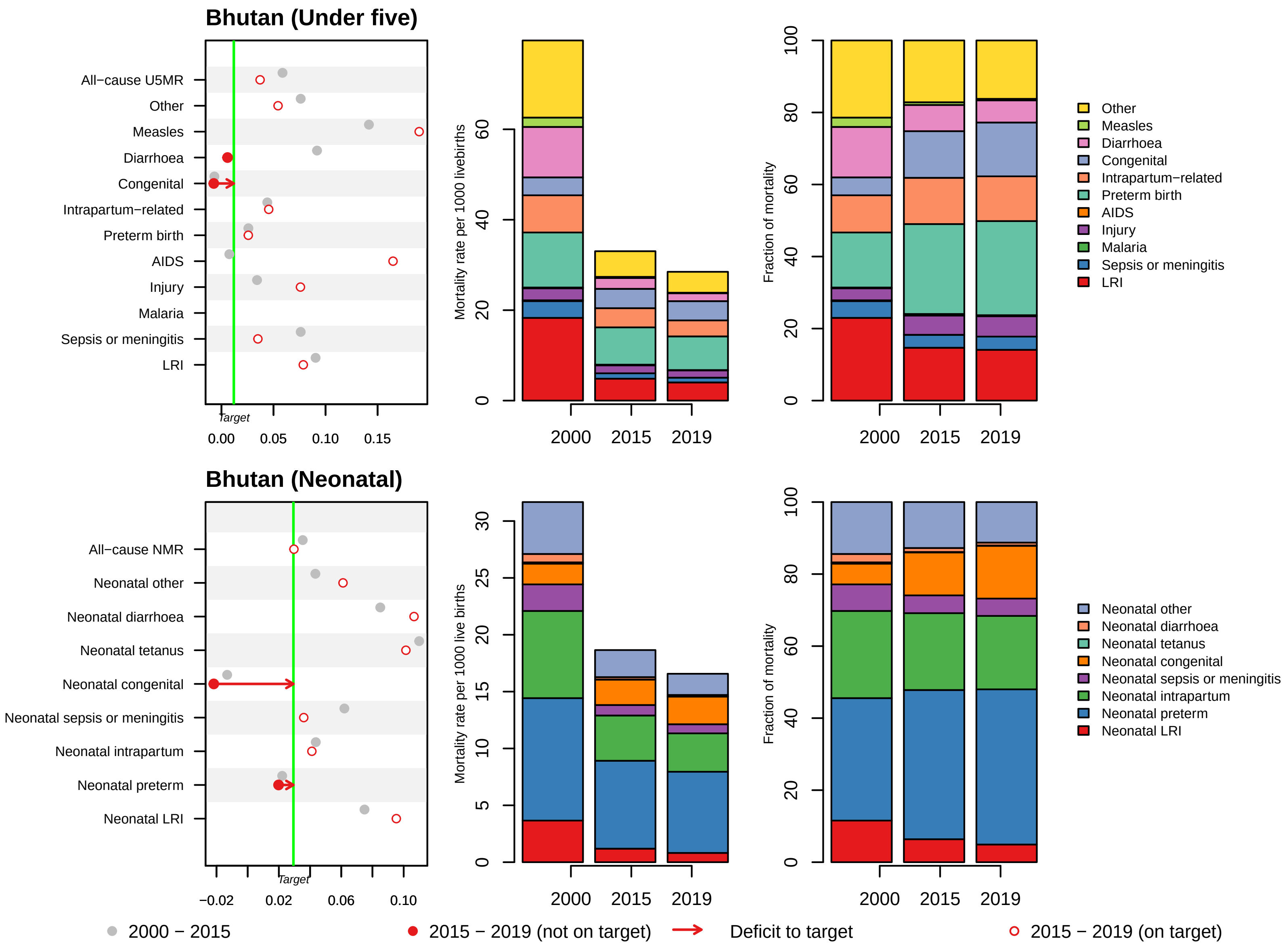

Bhutan (Under five)
All−cause U5MR
Other
Measles
Diarrhoea
Congenital
Intrapartum−related
Preterm birth
AIDS
Injury
Malaria
Sepsis or meningitis
LRI
Target
0.00
0.05
0.10
0.15
Mortality rate per 1000 livebirths
Fraction of mortality
2000
2015
2019
Other
Measles
Diarrhoea
Congenital
Intrapartum−related
Preterm birth
AIDS
Injury
Malaria
Sepsis or meningitis
LRI
Bhutan (Neonatal)
All−cause NMR
Neonatal other
Neonatal diarrhoea
Neonatal tetanus
Neonatal congenital
Neonatal sepsis or meningitis
Neonatal intrapartum
Neonatal preterm
Neonatal LRI
Target
−0.02
0.02
0.06
0.10
Mortality rate per 1000 live births
Fraction of mortality
Neonatal other
Neonatal diarrhoea
Neonatal tetanus
Neonatal congenital
Neonatal sepsis or meningitis
Neonatal intrapartum
Neonatal preterm
Neonatal LRI
2000 − 2015
2015 − 2019 (not on target)
Deficit to target
2015 − 2019 (on target)

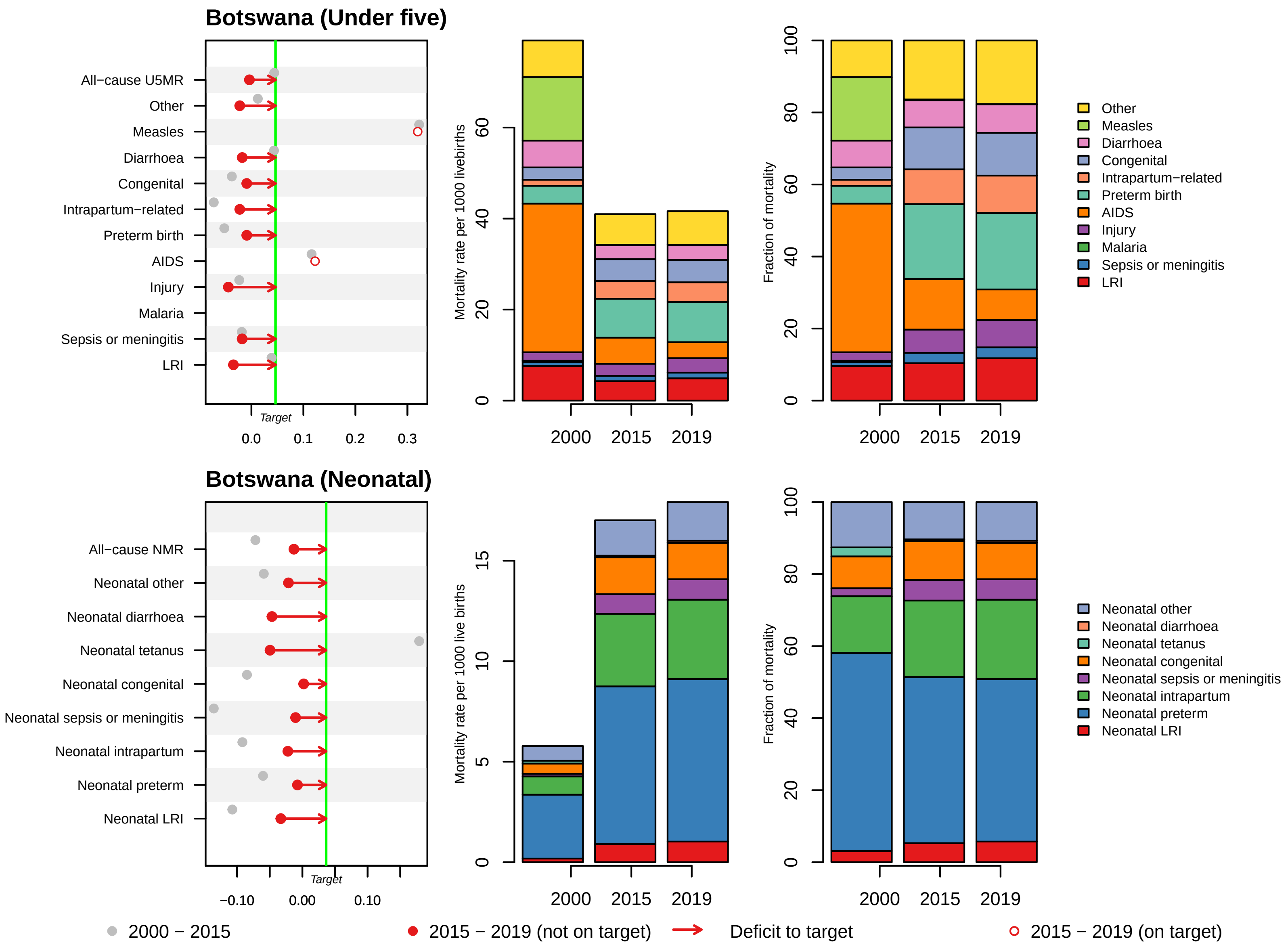

Botswana (Under five)
All−cause U5MR
Other
Measles
Diarrhoea
Congenital
Intrapartum−related
Preterm birth
AIDS
Injury
Malaria
Sepsis or meningitis
LRI
Target
0.0
0.1
0.2
0.3
Mortality rate per 1000 livebirths
0
20
40
60
2000
2015
2019
Fraction of mortality
0
20
40
60
80
100
2000
2015
2019
Other
Measles
Diarrhoea
Congenital
Intrapartum−related
Preterm birth
AIDS
Injury
Malaria
Sepsis or meningitis
LRI
Botswana (Neonatal)
All−cause NMR
Neonatal other
Neonatal diarrhoea
Neonatal tetanus
Neonatal congenital
Neonatal sepsis or meningitis
Neonatal intrapartum
Neonatal preterm
Neonatal LRI
Target
−0.10
0.00
0.10
Mortality rate per 1000 live births
0
5
10
15
2000
2015
2019
Fraction of mortality
0
20
40
60
80
100
2000
2015
2019
Neonatal other
Neonatal diarrhoea
Neonatal tetanus
Neonatal congenital
Neonatal sepsis or meningitis
Neonatal intrapartum
Neonatal preterm
Neonatal LRI
2000 − 2015
2015 − 2019 (not on target)
Deficit to target
2015 − 2019 (on target)

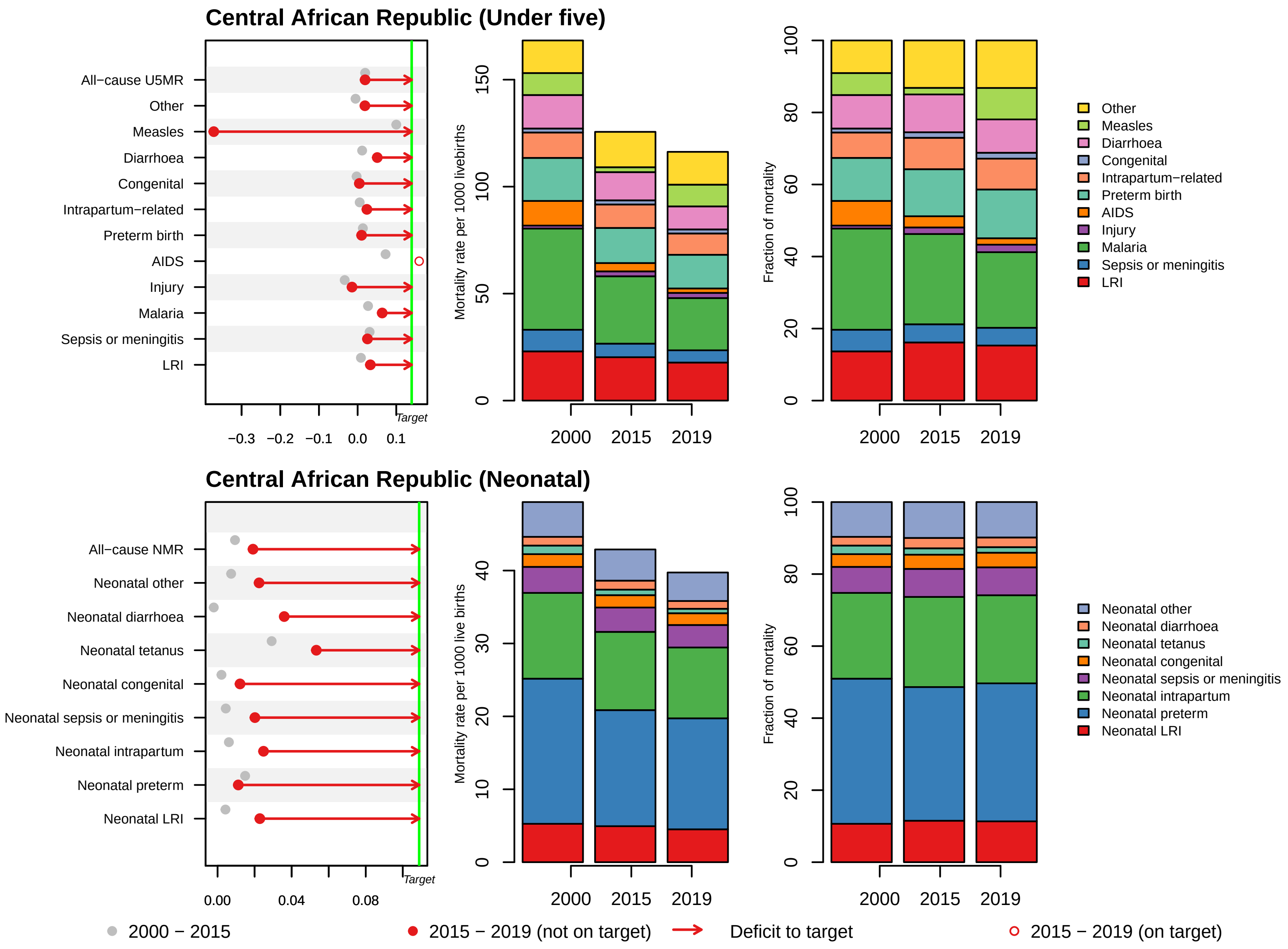

Central African Republic (Under five)
All−cause U5MR
Other
Measles
Diarrhoea
Congenital
Intrapartum−related
Preterm birth
AIDS
Injury
Malaria
Sepsis or meningitis
LRI
Target
−0.3
−0.2
−0.1
0.0
0.1
Mortality rate per 1000 livebirths
0
50
100
150
2000
2015
2019
Fraction of mortality
0
20
40
60
80
100
Central African Republic (Neonatal)
All−cause NMR
Neonatal other
Neonatal diarrhoea
Neonatal tetanus
Neonatal congenital
Neonatal sepsis or meningitis
Neonatal intrapartum
Neonatal preterm
Neonatal LRI
0.00
0.04
0.08
Mortality rate per 1000 live births
10
20
30
40
2000 − 2015
2015 − 2019 (not on target)
Deficit to target
2015 − 2019 (on target)

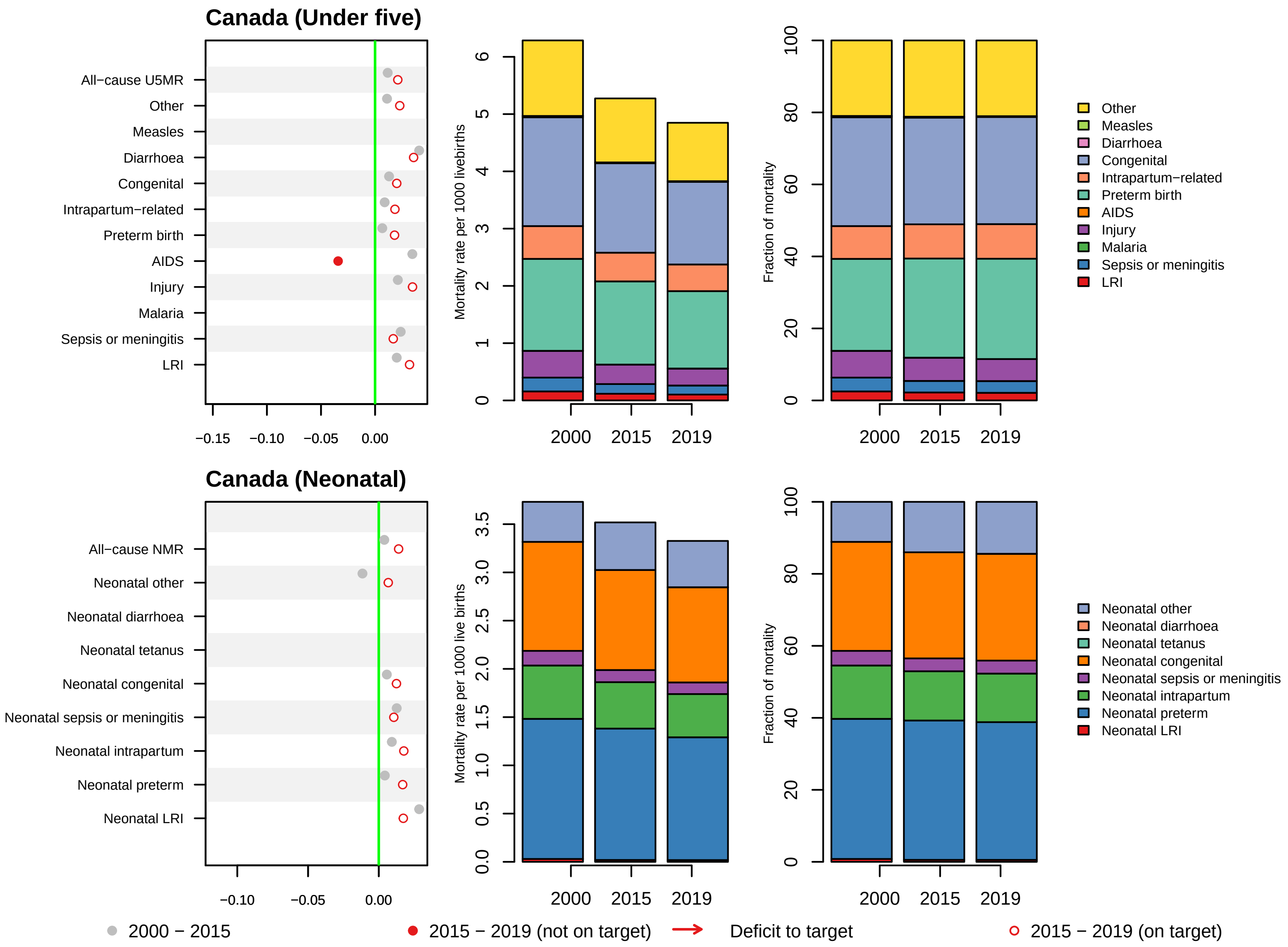

Canada (Under five)
All−cause U5MR
Other
Measles
Diarrhoea
Congenital
Intrapartum−related
Preterm birth
AIDS
Injury
Malaria
Sepsis or meningitis
LRI
−0.15
−0.10
−0.05
0.00
Mortality rate per 1000 livebirths
Fraction of mortality
2000
2015
2019
Other
Measles
Diarrhoea
Congenital
Intrapartum−related
Preterm birth
AIDS
Injury
Malaria
Sepsis or meningitis
LRI
Canada (Neonatal)
All−cause NMR
Neonatal other
Neonatal diarrhoea
Neonatal tetanus
Neonatal congenital
Neonatal sepsis or meningitis
Neonatal intrapartum
Neonatal preterm
Neonatal LRI
Mortality rate per 1000 live births
Neonatal other
Neonatal diarrhoea
Neonatal tetanus
Neonatal congenital
Neonatal sepsis or meningitis
Neonatal intrapartum
Neonatal preterm
Neonatal LRI
2000 − 2015
2015 − 2019 (not on target)
Deficit to target
2015 − 2019 (on target)

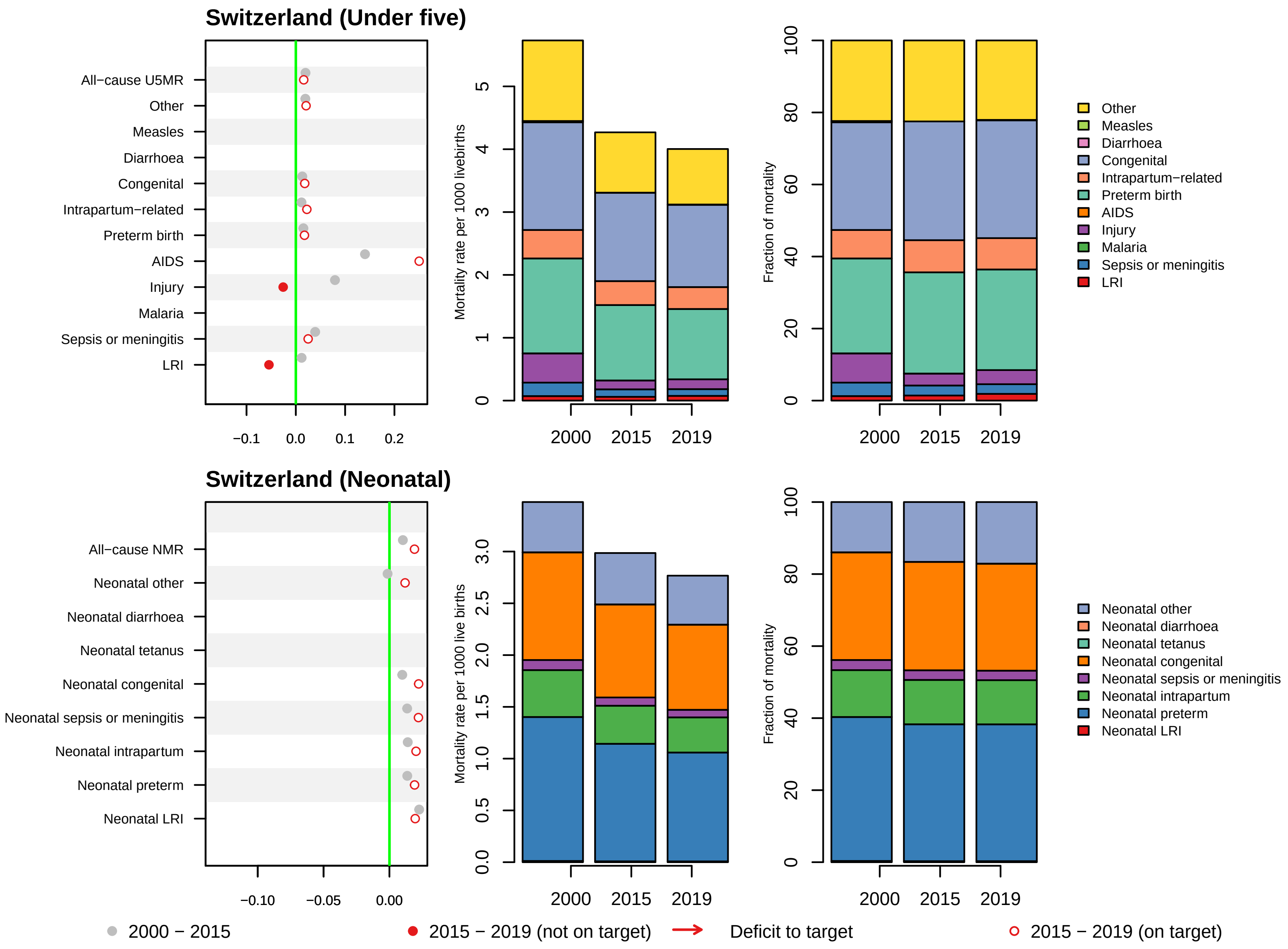

Switzerland (Under five)
All−cause U5MR
Other
Measles
Diarrhoea
Congenital
Intrapartum−related
Preterm birth
AIDS
Injury
Malaria
Sepsis or meningitis
LRI
−0.1
0.0
0.1
0.2
Mortality rate per 1000 livebirths
0
1
2
3
4
5
2000
2015
2019
Fraction of mortality
0
20
40
60
80
100
Other
Measles
Diarrhoea
Congenital
Intrapartum−related
Preterm birth
AIDS
Injury
Malaria
Sepsis or meningitis
LRI
Switzerland (Neonatal)
All−cause NMR
Neonatal other
Neonatal diarrhoea
Neonatal tetanus
Neonatal congenital
Neonatal sepsis or meningitis
Neonatal intrapartum
Neonatal preterm
Neonatal LRI
−0.10
−0.05
0.00
Mortality rate per 1000 live births
0.0
0.5
1.0
1.5
2.0
2.5
3.0
Neonatal other
Neonatal diarrhoea
Neonatal tetanus
Neonatal congenital
Neonatal sepsis or meningitis
Neonatal intrapartum
Neonatal preterm
Neonatal LRI
2000 − 2015
2015 − 2019 (not on target)
Deficit to target
2015 − 2019 (on target)

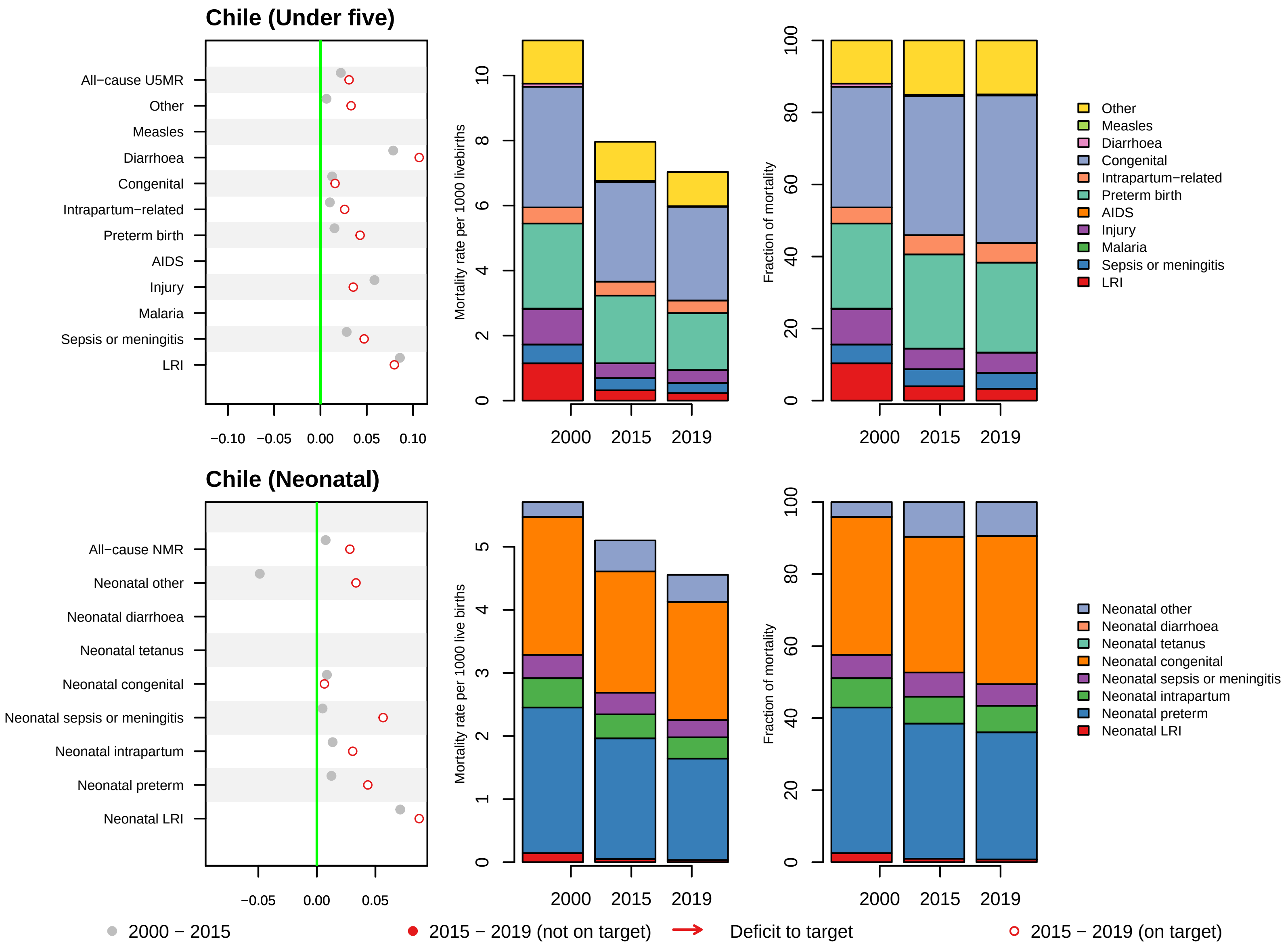

Chile (Under five)
All−cause U5MR
Other
Measles
Diarrhoea
Congenital
Intrapartum−related
Preterm birth
AIDS
Injury
Malaria
Sepsis or meningitis
LRI
−0.10
−0.05
0.00
0.05
0.10
Mortality rate per 1000 livebirths
Fraction of mortality
2000
2015
2019
Other
Measles
Diarrhoea
Congenital
Intrapartum−related
Preterm birth
AIDS
Injury
Malaria
Sepsis or meningitis
LRI
Chile (Neonatal)
All−cause NMR
Neonatal other
Neonatal diarrhoea
Neonatal tetanus
Neonatal congenital
Neonatal sepsis or meningitis
Neonatal intrapartum
Neonatal preterm
Neonatal LRI
−0.05
0.00
0.05
Mortality rate per 1000 live births
Fraction of mortality
2000
2015
2019
Neonatal other
Neonatal diarrhoea
Neonatal tetanus
Neonatal congenital
Neonatal sepsis or meningitis
Neonatal intrapartum
Neonatal preterm
Neonatal LRI
2000 − 2015
2015 − 2019 (not on target)
Deficit to target
2015 − 2019 (on target)

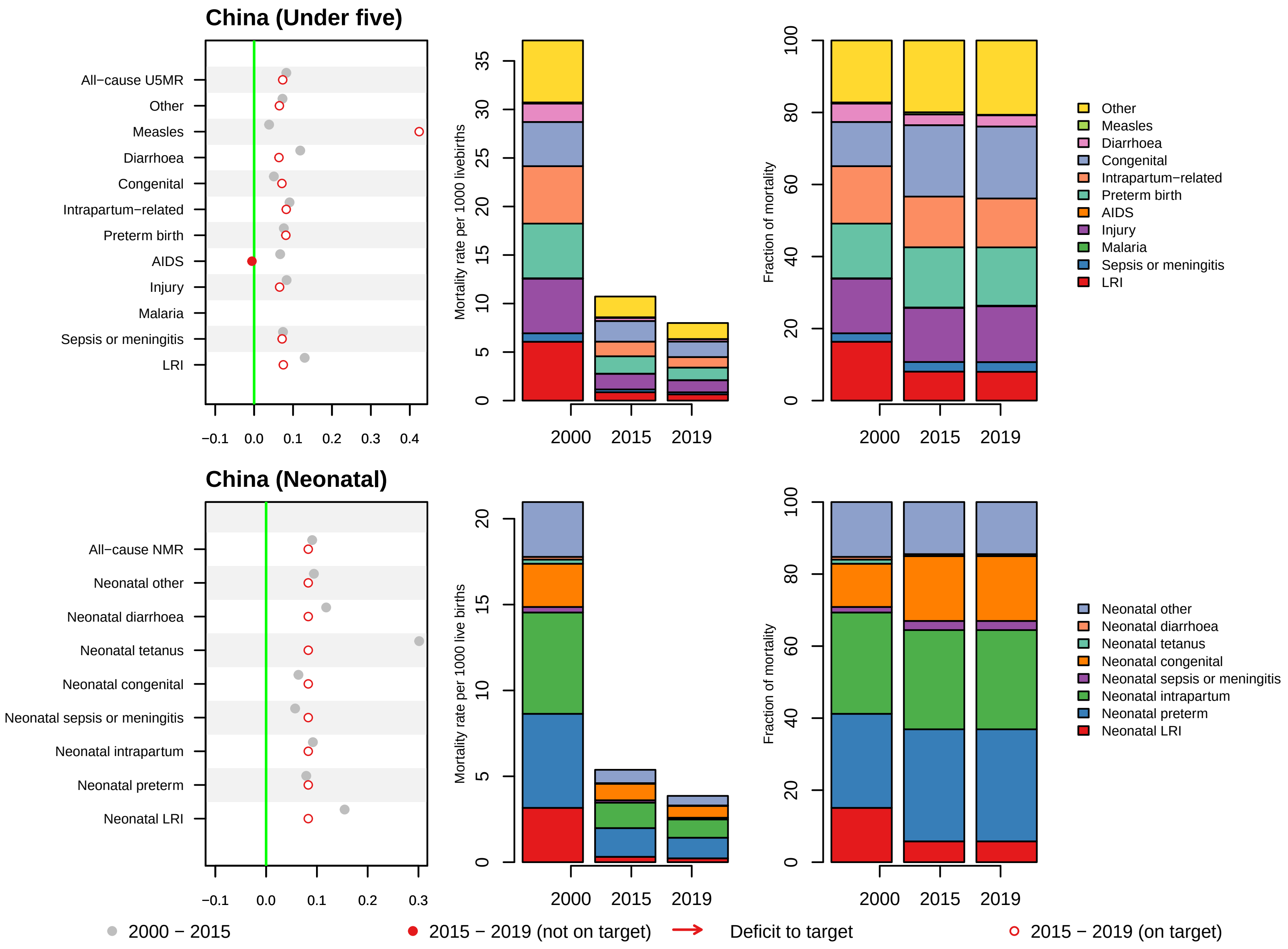
China (Under five)
All−cause U5MR
Other
Measles
Diarrhoea
Congenital
Intrapartum−related
Preterm birth
AIDS
Injury
Malaria
Sepsis or meningitis
LRI
−0.1 0.0 0.1 0.2 0.3 0.4
Mortality rate per 1000 livebirths
Fraction of mortality
2000 2015 2019
Other
Measles
Diarrhoea
Congenital
Intrapartum−related
Preterm birth
AIDS
Injury
Malaria
Sepsis or meningitis
LRI
China (Neonatal)
All−cause NMR
Neonatal other
Neonatal diarrhoea
Neonatal tetanus
Neonatal congenital
Neonatal sepsis or meningitis
Neonatal intrapartum
Neonatal preterm
Neonatal LRI
−0.1 0.0 0.1 0.2 0.3
Mortality rate per 1000 live births
Fraction of mortality
2000 2015 2019
Neonatal other
Neonatal diarrhoea
Neonatal tetanus
Neonatal congenital
Neonatal sepsis or meningitis
Neonatal intrapartum
Neonatal preterm
Neonatal LRI
2000 − 2015
2015 − 2019 (not on target)
Deficit to target
2015 − 2019 (on target)

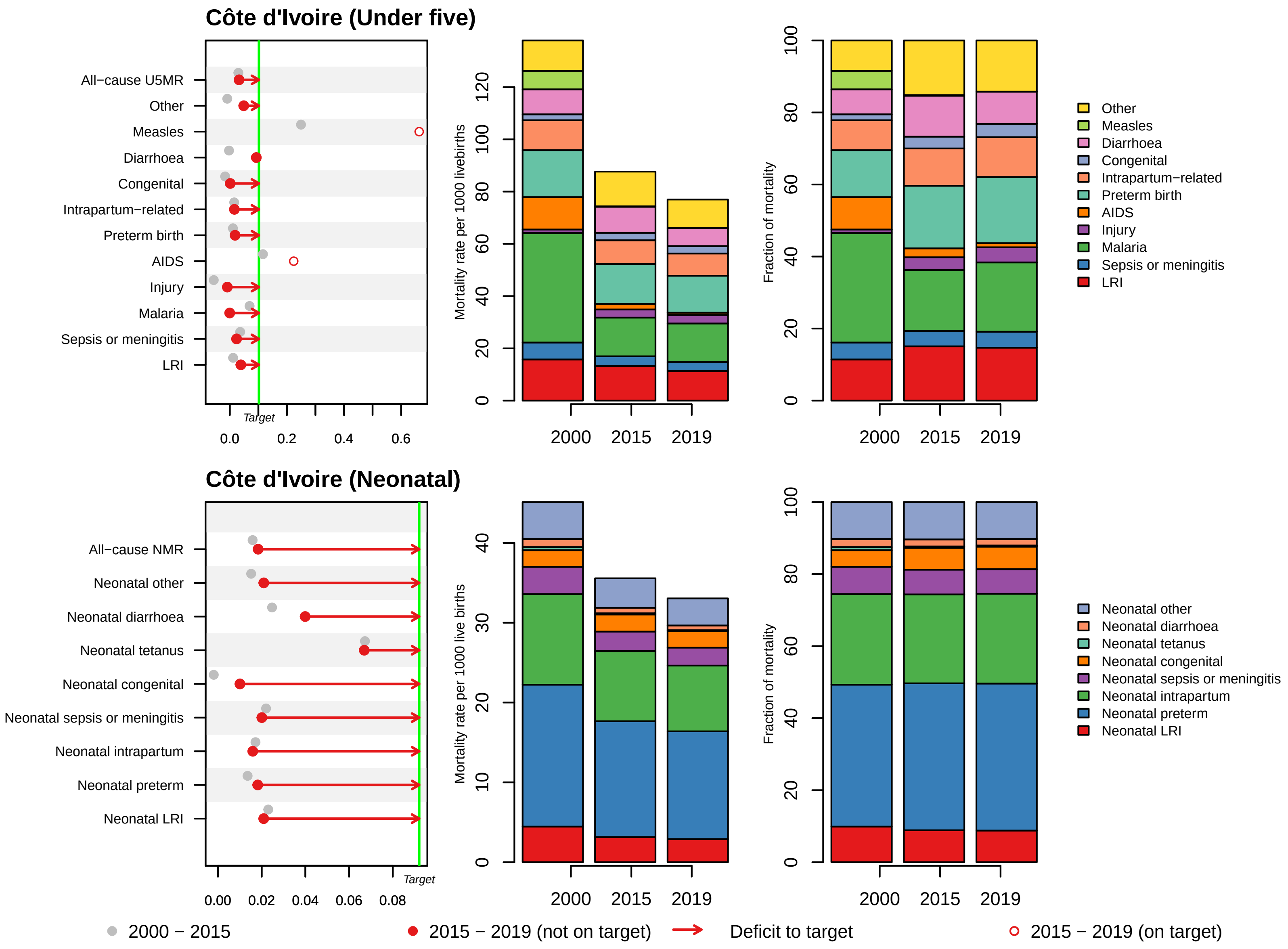

Côte d'Ivoire (Under five)
All−cause U5MR
Other
Measles
Diarrhoea
Congenital
Intrapartum−related
Preterm birth
AIDS
Injury
Malaria
Sepsis or meningitis
LRI
Target
0.0
0.2
0.4
0.6
Mortality rate per 1000 livebirths
Fraction of mortality
2000
2015
2019
Other
Measles
Diarrhoea
Congenital
Intrapartum−related
Preterm birth
AIDS
Injury
Malaria
Sepsis or meningitis
LRI
Côte d'Ivoire (Neonatal)
All−cause NMR
Neonatal other
Neonatal diarrhoea
Neonatal tetanus
Neonatal congenital
Neonatal sepsis or meningitis
Neonatal intrapartum
Neonatal preterm
Neonatal LRI
0.00
0.02
0.04
0.06
0.08
Target
Mortality rate per 1000 live births
Fraction of mortality
Neonatal other
Neonatal diarrhoea
Neonatal tetanus
Neonatal congenital
Neonatal sepsis or meningitis
Neonatal intrapartum
Neonatal preterm
Neonatal LRI
2000 − 2015
2015 − 2019 (not on target)
Deficit to target
2015 − 2019 (on target)

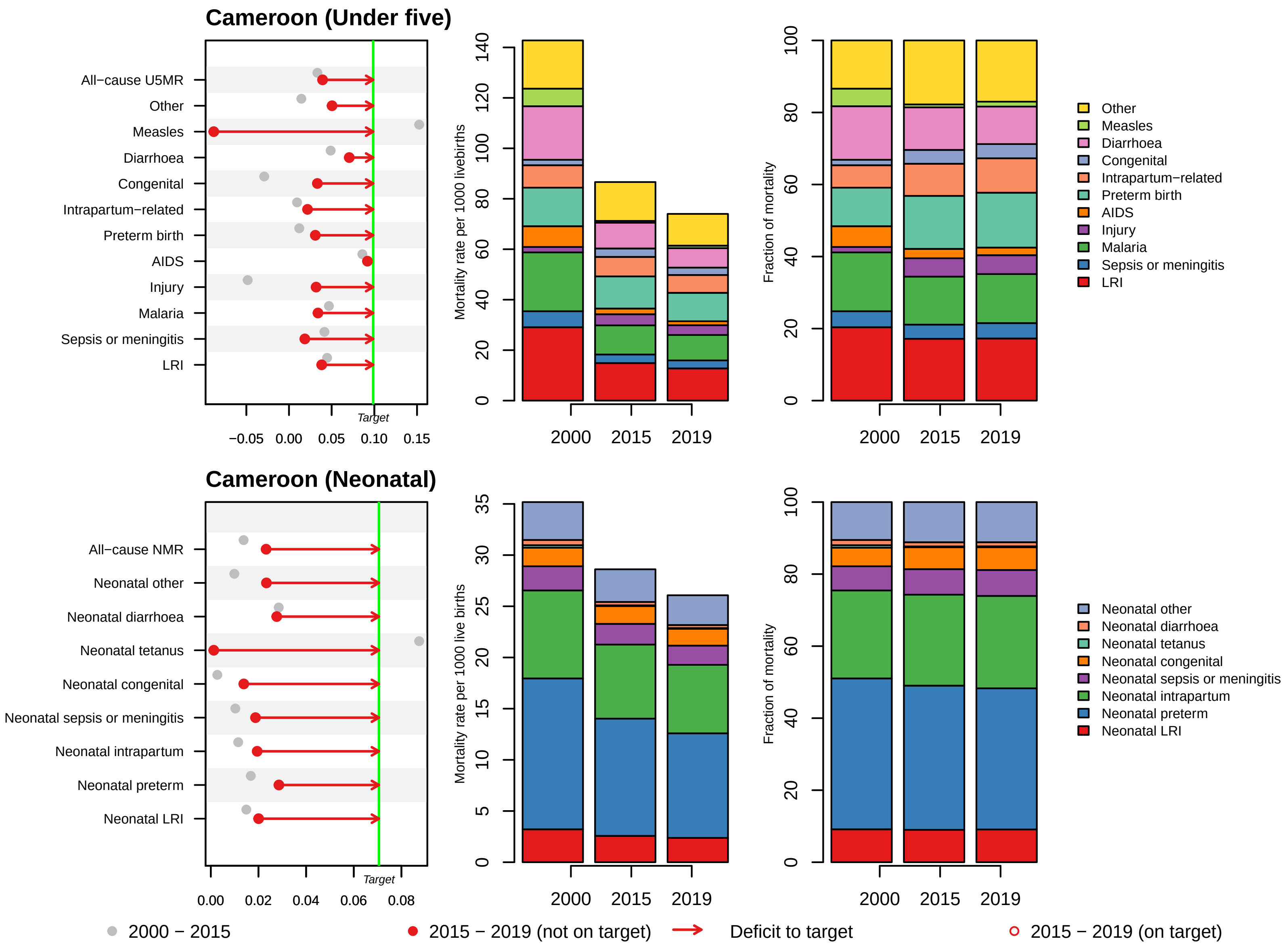

Cameroon (Under five)
All−cause U5MR
Other
Measles
Diarrhoea
Congenital
Intrapartum−related
Preterm birth
AIDS
Injury
Malaria
Sepsis or meningitis
LRI
Target
−0.05 0.00 0.05 0.10 0.15
Mortality rate per 1000 livebirths
0 20 40 60 80 100 120 140
2000 2015 2019
Fraction of mortality
0 20 40 60 80 100
2000 2015 2019
Other
Measles
Diarrhoea
Congenital
Intrapartum−related
Preterm birth
AIDS
Injury
Malaria
Sepsis or meningitis
LRI
Cameroon (Neonatal)
All−cause NMR
Neonatal other
Neonatal diarrhoea
Neonatal tetanus
Neonatal congenital
Neonatal sepsis or meningitis
Neonatal intrapartum
Neonatal preterm
Neonatal LRI
Target
0.00 0.02 0.04 0.06 0.08
Mortality rate per 1000 live births
0 5 10 15 20 25 30 35
2000 2015 2019
Fraction of mortality
0 20 40 60 80 100
2000 2015 2019
Neonatal other
Neonatal diarrhoea
Neonatal tetanus
Neonatal congenital
Neonatal sepsis or meningitis
Neonatal intrapartum
Neonatal preterm
Neonatal LRI
2000 − 2015
2015 − 2019 (not on target)
Deficit to target
2015 − 2019 (on target)

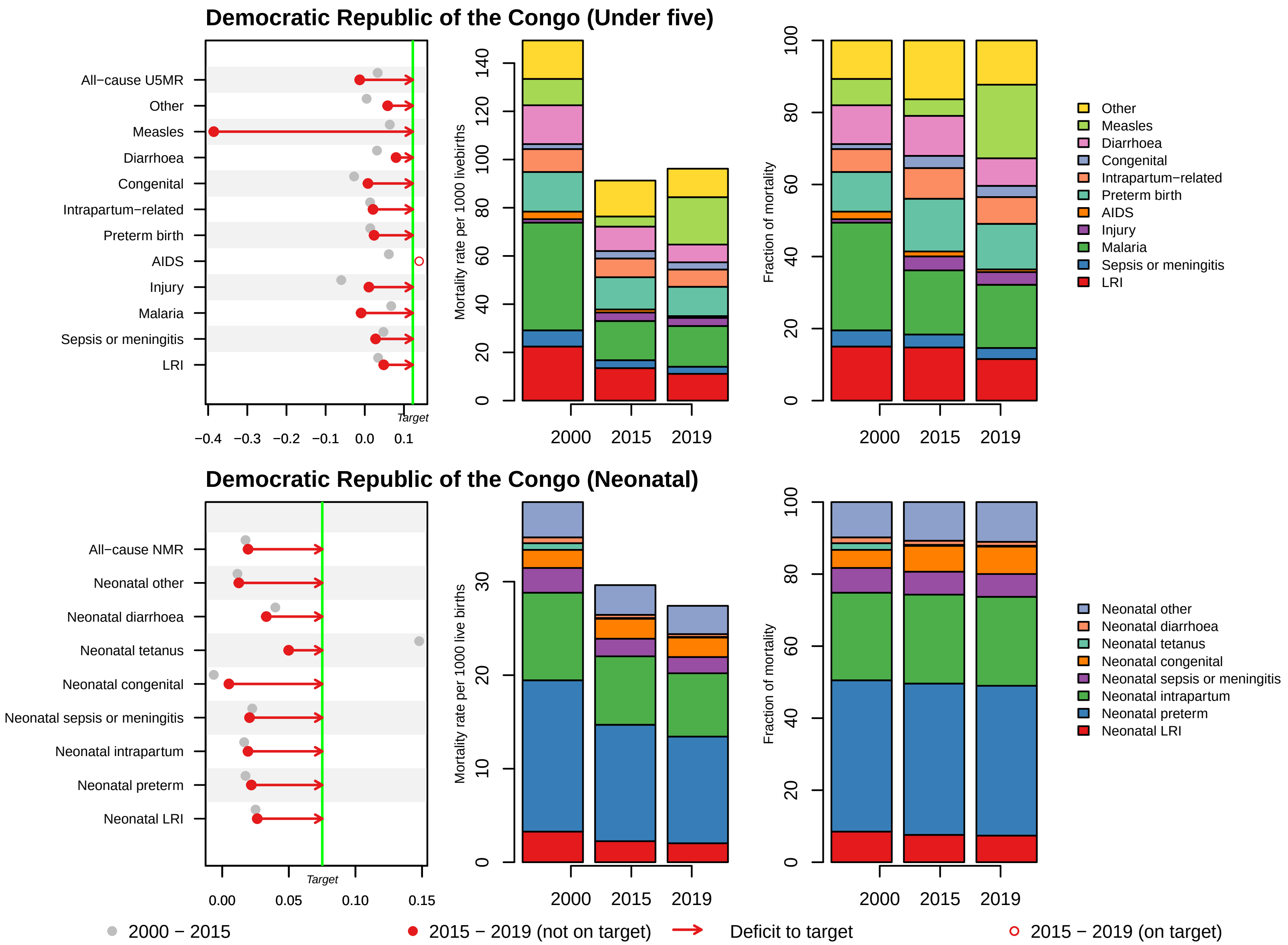
Democratic Republic of the Congo (Under five)
All−cause U5MR
Other
Measles
Diarrhoea
Congenital
Intrapartum−related
Preterm birth
AIDS
Injury
Malaria
Sepsis or meningitis
LRI
Target
−0.4 −0.3 −0.2 −0.1 0.0 0.1
Mortality rate per 1000 livebirths
0 20 40 60 80 100 120 140
2000 2015 2019
Fraction of mortality
0 20 40 60 80 100
2000 2015 2019
Other
Measles
Diarrhoea
Congenital
Intrapartum−related
Preterm birth
AIDS
Injury
Malaria
Sepsis or meningitis
LRI
Democratic Republic of the Congo (Neonatal)
All−cause NMR
Neonatal other
Neonatal diarrhoea
Neonatal tetanus
Neonatal congenital
Neonatal sepsis or meningitis
Neonatal intrapartum
Neonatal preterm
Neonatal LRI
Target
0.00 0.05 0.10 0.15
Mortality rate per 1000 live births
0 10 20 30
2000 2015 2019
Fraction of mortality
0 20 40 60 80 100
2000 2015 2019
Neonatal other
Neonatal diarrhoea
Neonatal tetanus
Neonatal congenital
Neonatal sepsis or meningitis
Neonatal intrapartum
Neonatal preterm
Neonatal LRI
2000 − 2015
2015 − 2019 (not on target)
Deficit to target
2015 − 2019 (on target)

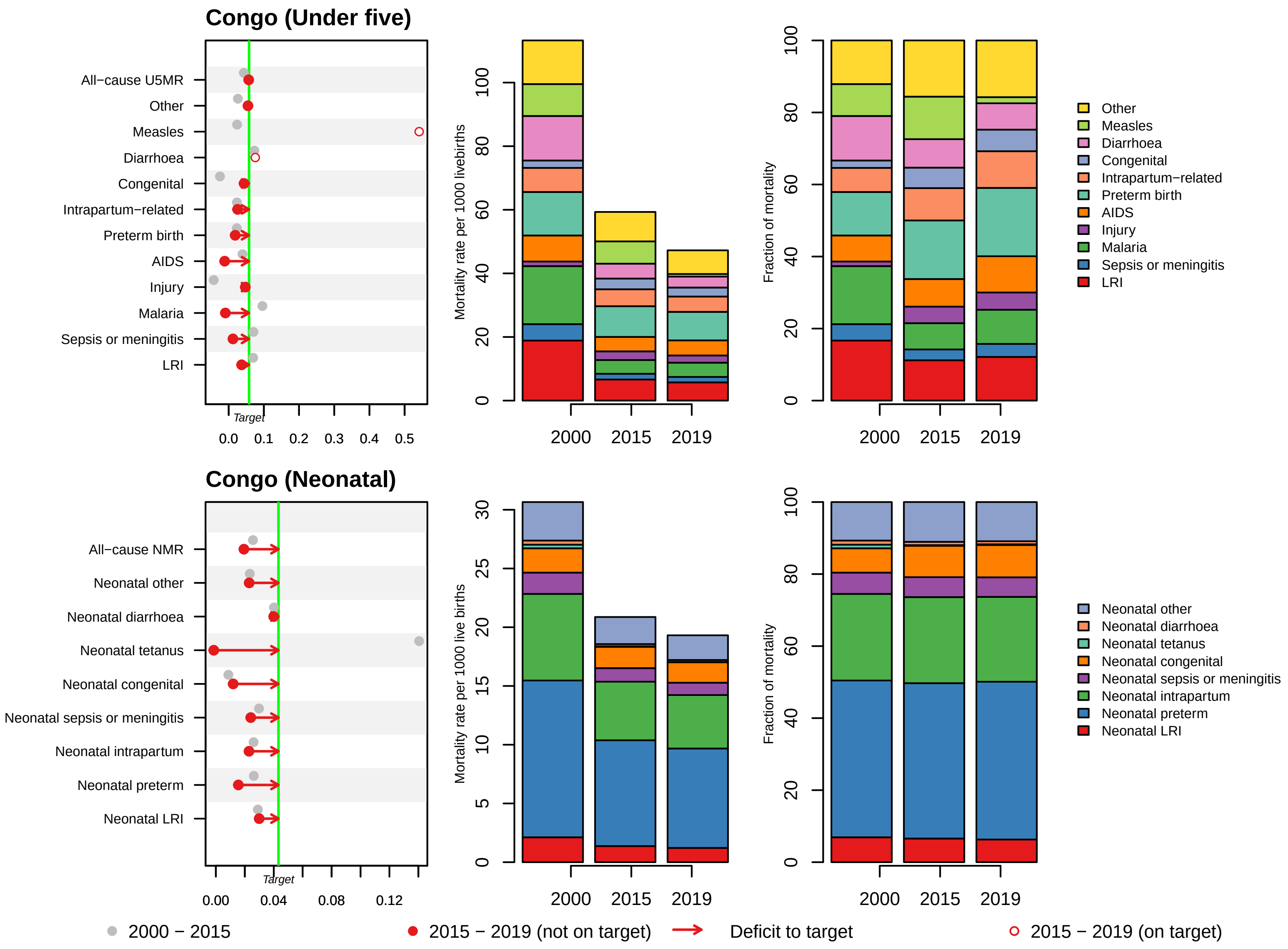

Congo (Under five)
All−cause U5MR
Other
Measles
Diarrhoea
Congenital
Intrapartum−related
Preterm birth
AIDS
Injury
Malaria
Sepsis or meningitis
LRI
Target
0.0
0.1
0.2
0.3
0.4
0.5
Mortality rate per 1000 livebirths
0
20
40
60
80
100
2000
2015
2019
Fraction of mortality
0
20
40
60
80
100
2000
2015
2019
Other
Measles
Diarrhoea
Congenital
Intrapartum−related
Preterm birth
AIDS
Injury
Malaria
Sepsis or meningitis
LRI
Congo (Neonatal)
All−cause NMR
Neonatal other
Neonatal diarrhoea
Neonatal tetanus
Neonatal congenital
Neonatal sepsis or meningitis
Neonatal intrapartum
Neonatal preterm
Neonatal LRI
Target
0.00
0.04
0.08
0.12
Mortality rate per 1000 live births
0
5
10
15
20
25
30
2000
2015
2019
Fraction of mortality
0
20
40
60
80
100
2000
2015
2019
Neonatal other
Neonatal diarrhoea
Neonatal tetanus
Neonatal congenital
Neonatal sepsis or meningitis
Neonatal intrapartum
Neonatal preterm
Neonatal LRI
2000 − 2015
2015 − 2019 (not on target)
Deficit to target
2015 − 2019 (on target)

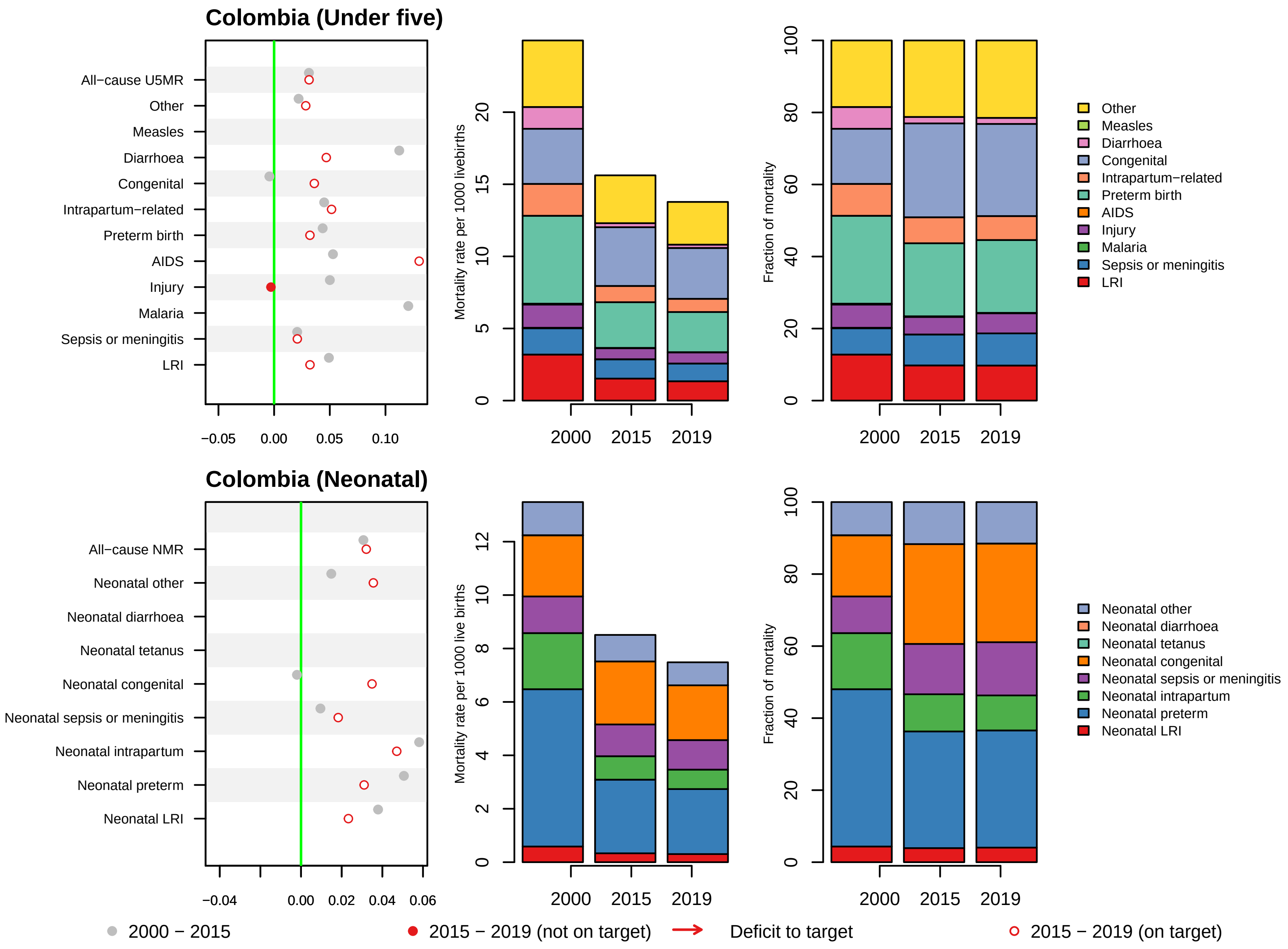

Colombia (Under five)
All−cause U5MR
Other
Measles
Diarrhoea
Congenital
Intrapartum−related
Preterm birth
AIDS
Injury
Malaria
Sepsis or meningitis
LRI
−0.05
0.00
0.05
0.10
Mortality rate per 1000 livebirths
0
5
10
15
20
2000
2015
2019
Fraction of mortality
0
20
40
60
80
100
2000
2015
2019
Other
Measles
Diarrhoea
Congenital
Intrapartum−related
Preterm birth
AIDS
Injury
Malaria
Sepsis or meningitis
LRI
Colombia (Neonatal)
All−cause NMR
Neonatal other
Neonatal diarrhoea
Neonatal tetanus
Neonatal congenital
Neonatal sepsis or meningitis
Neonatal intrapartum
Neonatal preterm
Neonatal LRI
−0.04
0.00
0.02
0.04
0.06
Mortality rate per 1000 live births
0
2
4
6
8
10
12
2000
2015
2019
Fraction of mortality
0
20
40
60
80
100
2000
2015
2019
Neonatal other
Neonatal diarrhoea
Neonatal tetanus
Neonatal congenital
Neonatal sepsis or meningitis
Neonatal intrapartum
Neonatal preterm
Neonatal LRI
2000 − 2015
2015 − 2019 (not on target)
Deficit to target
2015 − 2019 (on target)

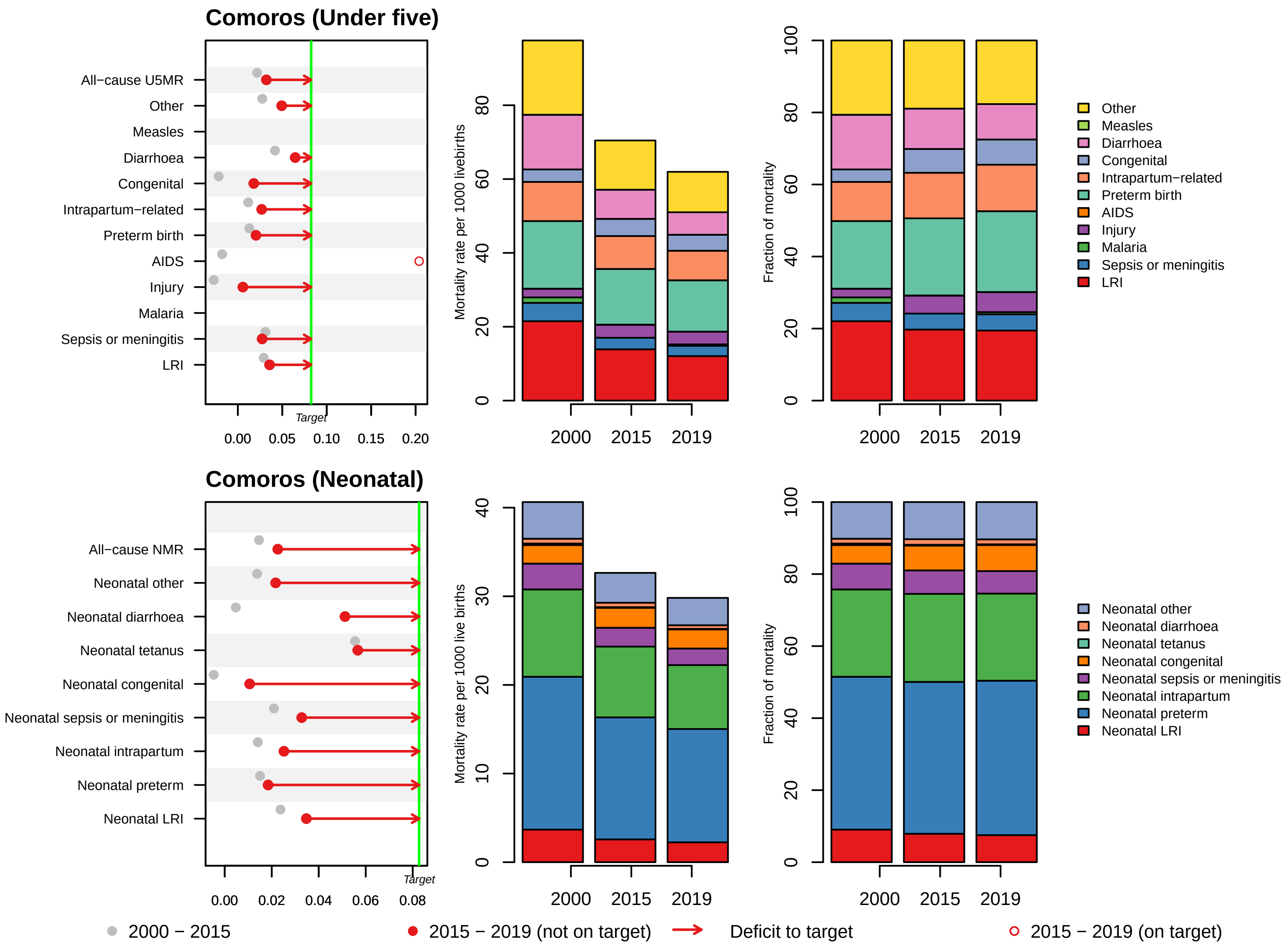
Comoros (Under five)
All−cause U5MR
Other
Measles
Diarrhoea
Congenital
Intrapartum−related
Preterm birth
AIDS
Injury
Malaria
Sepsis or meningitis
LRI
Target
0.00
0.05
0.10
0.15
0.20
Mortality rate per 1000 livebirths
Fraction of mortality
2000
2015
2019
Other
Measles
Diarrhoea
Congenital
Intrapartum−related
Preterm birth
AIDS
Injury
Malaria
Sepsis or meningitis
LRI
Comoros (Neonatal)
All−cause NMR
Neonatal other
Neonatal diarrhoea
Neonatal tetanus
Neonatal congenital
Neonatal sepsis or meningitis
Neonatal intrapartum
Neonatal preterm
Neonatal LRI
Target
0.00
0.02
0.04
0.06
0.08
Mortality rate per 1000 live births
Fraction of mortality
2000
2015
2019
Neonatal other
Neonatal diarrhoea
Neonatal tetanus
Neonatal congenital
Neonatal sepsis or meningitis
Neonatal intrapartum
Neonatal preterm
Neonatal LRI
2000 − 2015
2015 − 2019 (not on target)
Deficit to target
2015 − 2019 (on target)

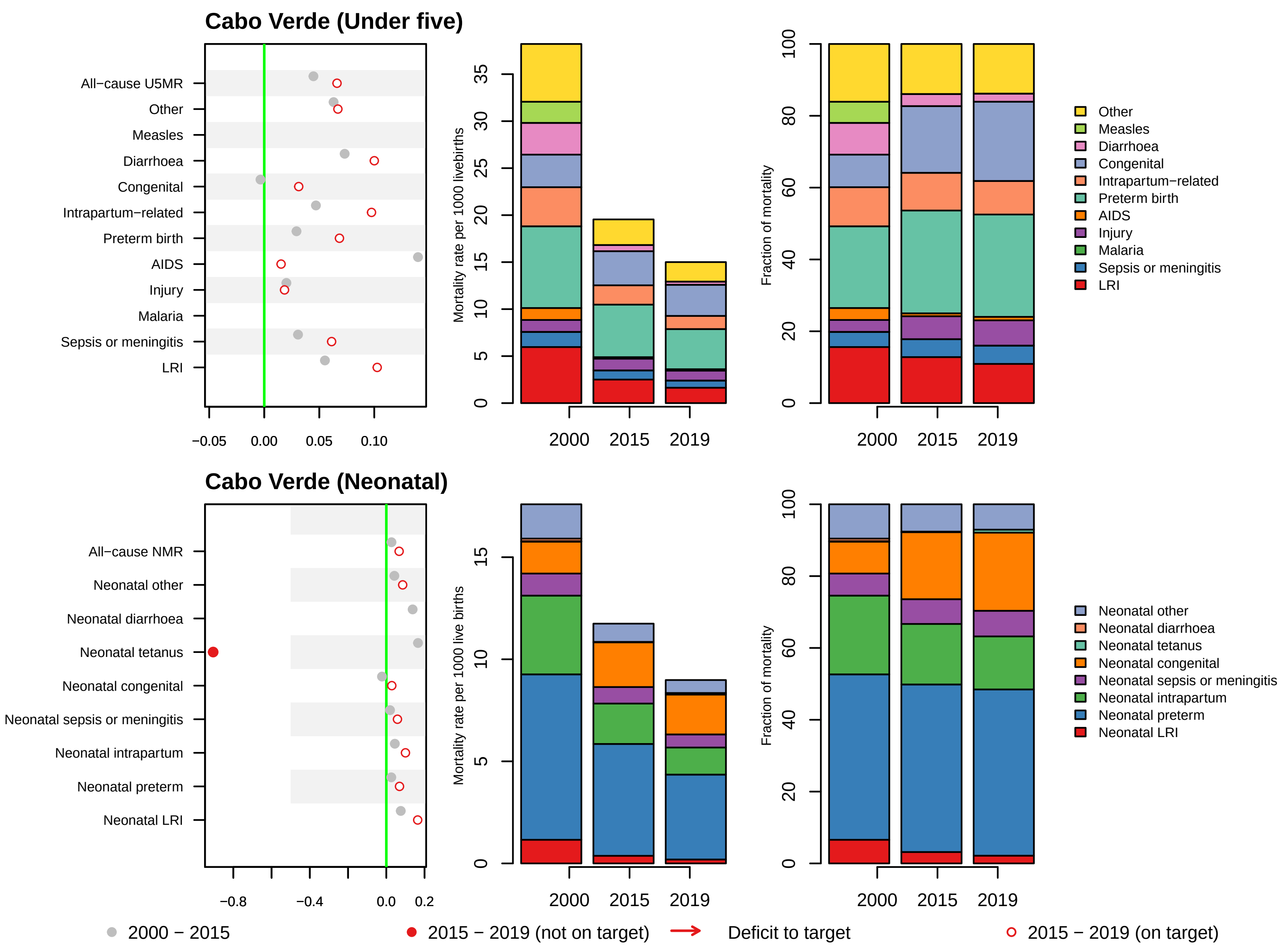

Cabo Verde (Under five)
All−cause U5MR
Other
Measles
Diarrhoea
Congenital
Intrapartum−related
Preterm birth
AIDS
Injury
Malaria
Sepsis or meningitis
LRI
−0.05
0.00
0.05
0.10
Mortality rate per 1000 livebirths
Fraction of mortality
2000
2015
2019
Cabo Verde (Neonatal)
All−cause NMR
Neonatal other
Neonatal diarrhoea
Neonatal tetanus
Neonatal congenital
Neonatal sepsis or meningitis
Neonatal intrapartum
Neonatal preterm
Neonatal LRI
−0.8
−0.4
0.0
0.2
Mortality rate per 1000 live births
2000 − 2015
2015 − 2019 (not on target)
Deficit to target
2015 − 2019 (on target)

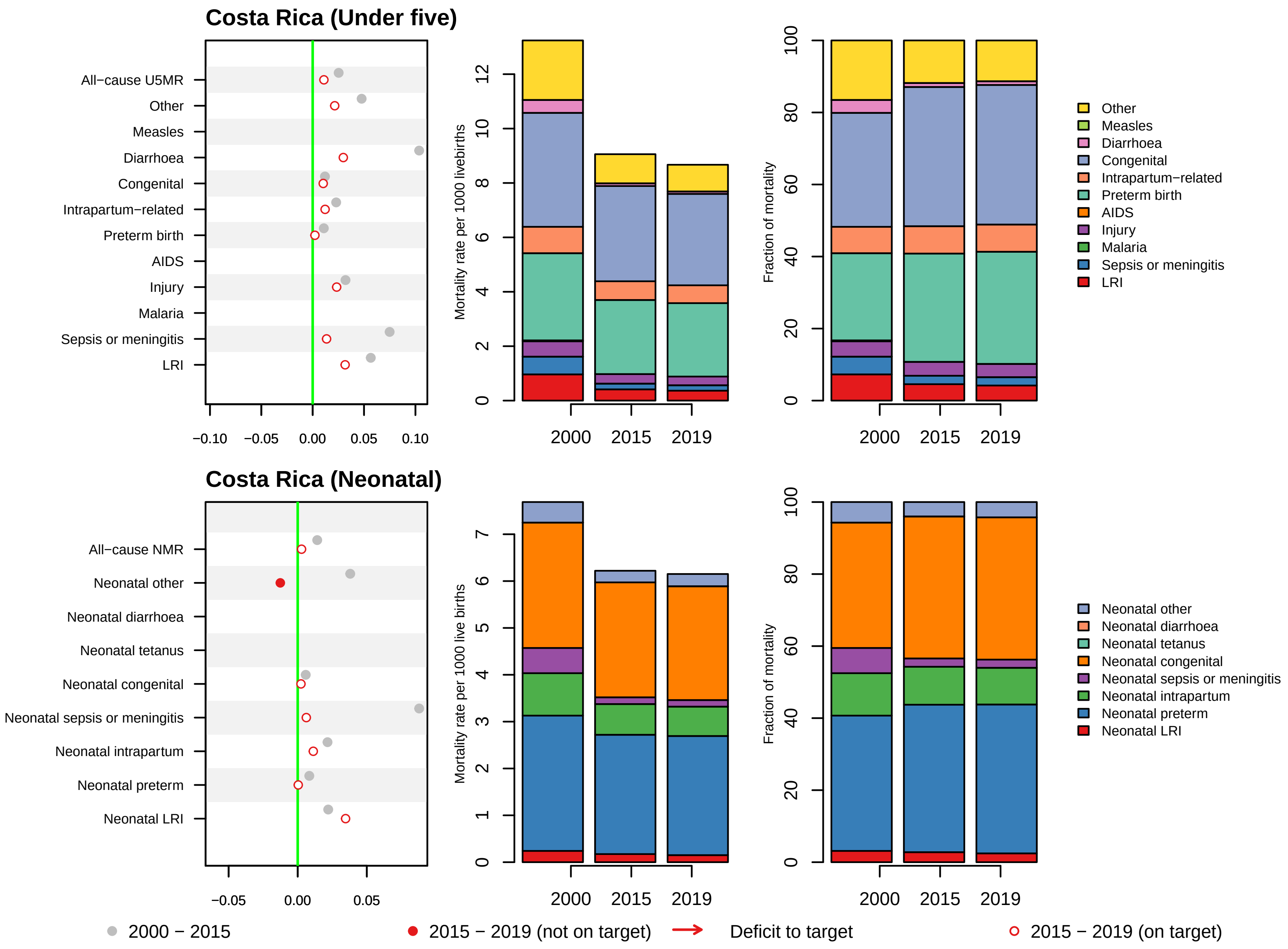
Costa Rica (Under five)
All−cause U5MR
Other
Measles
Diarrhoea
Congenital
Intrapartum−related
Preterm birth
AIDS
Injury
Malaria
Sepsis or meningitis
LRI
−0.10
−0.05
0.00
0.05
0.10
Mortality rate per 1000 livebirths
Fraction of mortality
2000
2015
2019
Other
Measles
Diarrhoea
Congenital
Intrapartum−related
Preterm birth
AIDS
Injury
Malaria
Sepsis or meningitis
LRI
Costa Rica (Neonatal)
All−cause NMR
Neonatal other
Neonatal diarrhoea
Neonatal tetanus
Neonatal congenital
Neonatal sepsis or meningitis
Neonatal intrapartum
Neonatal preterm
Neonatal LRI
−0.05
0.00
0.05
Mortality rate per 1000 live births
Fraction of mortality
2000
2015
2019
Neonatal other
Neonatal diarrhoea
Neonatal tetanus
Neonatal congenital
Neonatal sepsis or meningitis
Neonatal intrapartum
Neonatal preterm
Neonatal LRI
2000 − 2015
2015 − 2019 (not on target)
Deficit to target
2015 − 2019 (on target)

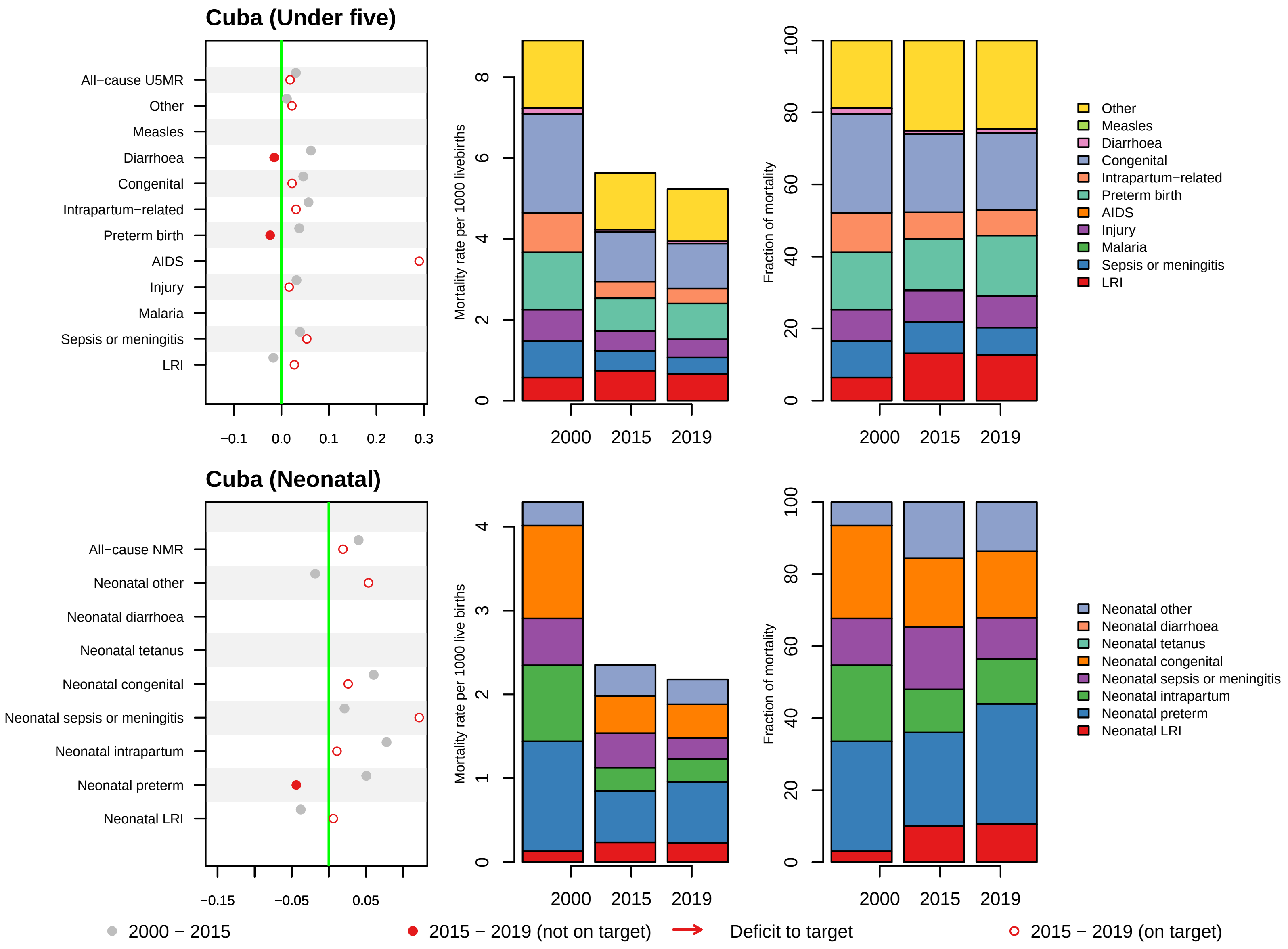

Cuba (Under five)
All−cause U5MR
Other
Measles
Diarrhoea
Congenital
Intrapartum−related
Preterm birth
AIDS
Injury
Malaria
Sepsis or meningitis
LRI
−0.1
0.0
0.1
0.2
0.3
Mortality rate per 1000 livebirths
Fraction of mortality
2000
2015
2019
Other
Measles
Diarrhoea
Congenital
Intrapartum−related
Preterm birth
AIDS
Injury
Malaria
Sepsis or meningitis
LRI
Cuba (Neonatal)
All−cause NMR
Neonatal other
Neonatal diarrhoea
Neonatal tetanus
Neonatal congenital
Neonatal sepsis or meningitis
Neonatal intrapartum
Neonatal preterm
Neonatal LRI
−0.15
−0.05
0.05
Mortality rate per 1000 live births
Fraction of mortality
2000
2015
2019
Neonatal other
Neonatal diarrhoea
Neonatal tetanus
Neonatal congenital
Neonatal sepsis or meningitis
Neonatal intrapartum
Neonatal preterm
Neonatal LRI
2000 − 2015
2015 − 2019 (not on target)
Deficit to target
2015 − 2019 (on target)

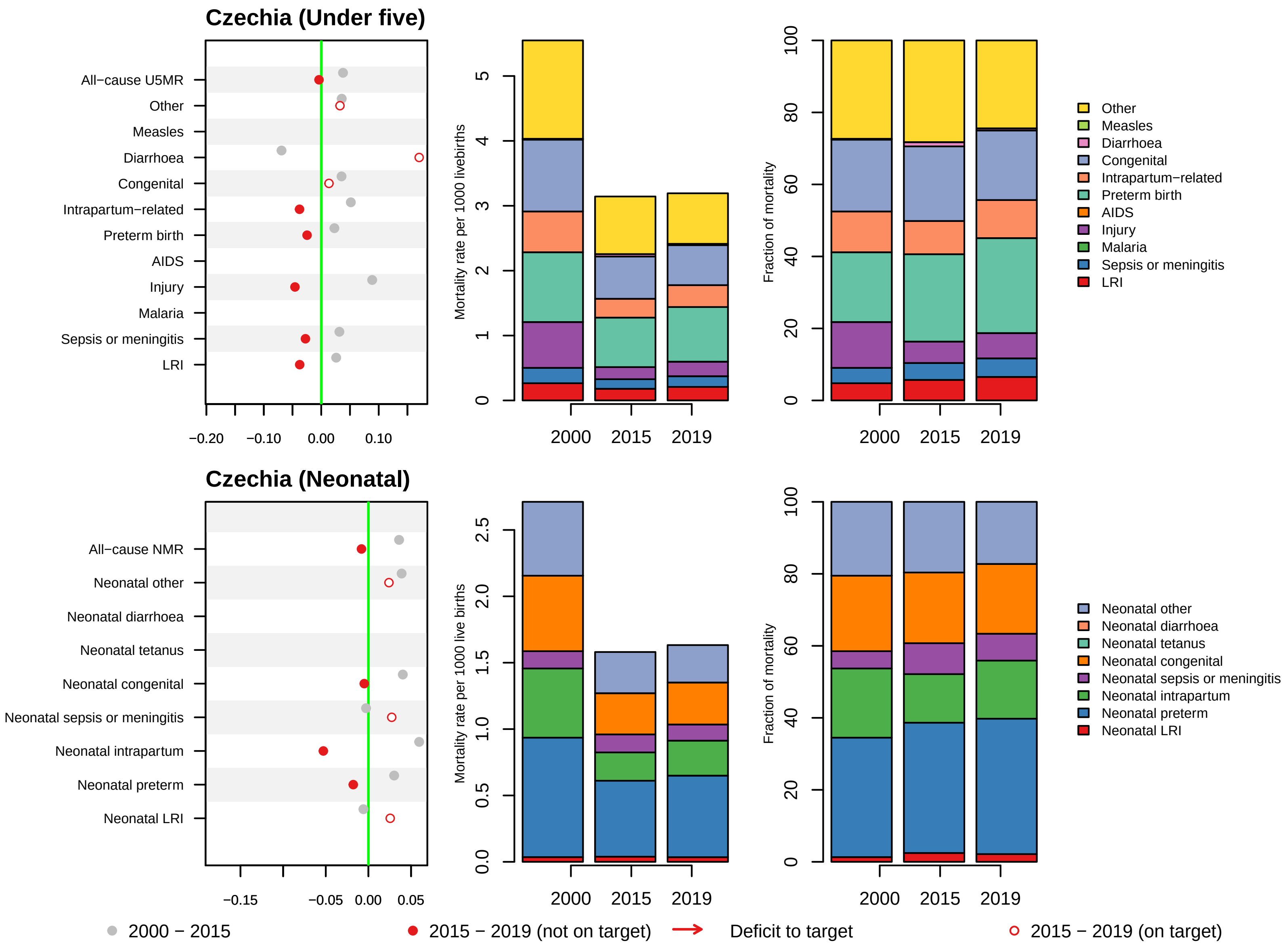

Czechia (Under five)
All−cause U5MR
Other
Measles
Diarrhoea
Congenital
Intrapartum−related
Preterm birth
AIDS
Injury
Malaria
Sepsis or meningitis
LRI
−0.20
−0.10
0.00
0.10
Mortality rate per 1000 livebirths
Fraction of mortality
2000
2015
2019
Other
Measles
Diarrhoea
Congenital
Intrapartum−related
Preterm birth
AIDS
Injury
Malaria
Sepsis or meningitis
LRI
Czechia (Neonatal)
All−cause NMR
Neonatal other
Neonatal diarrhoea
Neonatal tetanus
Neonatal congenital
Neonatal sepsis or meningitis
Neonatal intrapartum
Neonatal preterm
Neonatal LRI
−0.15
−0.05
0.00
0.05
Mortality rate per 1000 live births
Fraction of mortality
2000
2015
2019
Neonatal other
Neonatal diarrhoea
Neonatal tetanus
Neonatal congenital
Neonatal sepsis or meningitis
Neonatal intrapartum
Neonatal preterm
Neonatal LRI
2000 − 2015
2015 − 2019 (not on target)
Deficit to target
2015 − 2019 (on target)

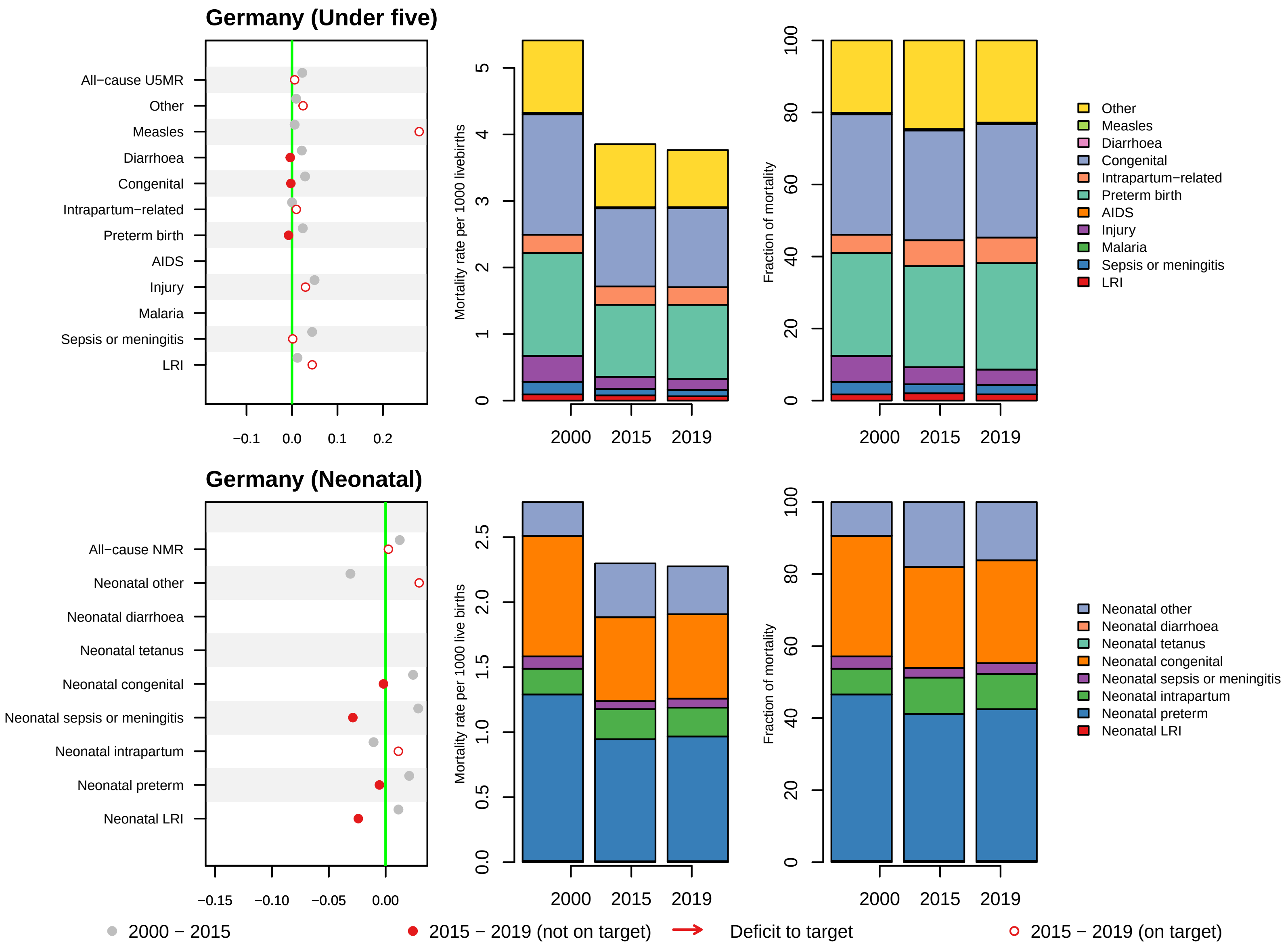
Germany (Under five)
All−cause U5MR
Other
Measles
Diarrhoea
Congenital
Intrapartum−related
Preterm birth
AIDS
Injury
Malaria
Sepsis or meningitis
LRI
−0.1
0.0
0.1
0.2
Mortality rate per 1000 livebirths
Fraction of mortality
2000
2015
2019
Other
Measles
Diarrhoea
Congenital
Intrapartum−related
Preterm birth
AIDS
Injury
Malaria
Sepsis or meningitis
LRI
Germany (Neonatal)
All−cause NMR
Neonatal other
Neonatal diarrhoea
Neonatal tetanus
Neonatal congenital
Neonatal sepsis or meningitis
Neonatal intrapartum
Neonatal preterm
Neonatal LRI
−0.15
−0.10
−0.05
0.00
Mortality rate per 1000 live births
Fraction of mortality
2000
2015
2019
Neonatal other
Neonatal diarrhoea
Neonatal tetanus
Neonatal congenital
Neonatal sepsis or meningitis
Neonatal intrapartum
Neonatal preterm
Neonatal LRI
2000 − 2015
2015 − 2019 (not on target)
Deficit to target
2015 − 2019 (on target)

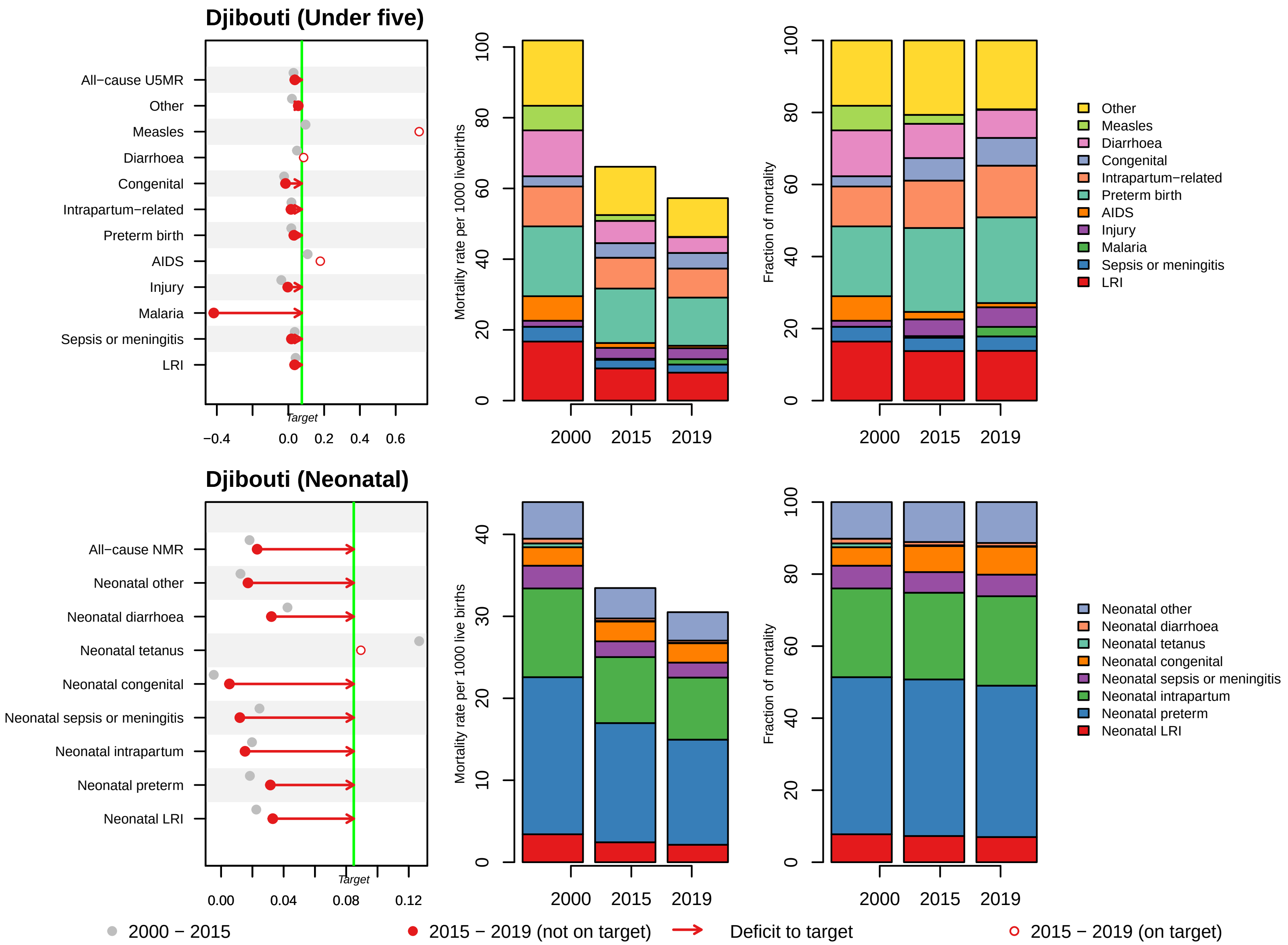

Djibouti (Under five)
All−cause U5MR
Other
Measles
Diarrhoea
Congenital
Intrapartum−related
Preterm birth
AIDS
Injury
Malaria
Sepsis or meningitis
LRI
Target
−0.4
0.0
0.2
0.4
0.6
Mortality rate per 1000 livebirths
Fraction of mortality
2000
2015
2019
Other
Measles
Diarrhoea
Congenital
Intrapartum−related
Preterm birth
AIDS
Injury
Malaria
Sepsis or meningitis
LRI
Djibouti (Neonatal)
All−cause NMR
Neonatal other
Neonatal diarrhoea
Neonatal tetanus
Neonatal congenital
Neonatal sepsis or meningitis
Neonatal intrapartum
Neonatal preterm
Neonatal LRI
Target
0.00
0.04
0.08
0.12
Mortality rate per 1000 live births
Fraction of mortality
Neonatal other
Neonatal diarrhoea
Neonatal tetanus
Neonatal congenital
Neonatal sepsis or meningitis
Neonatal intrapartum
Neonatal preterm
Neonatal LRI
2000 − 2015
2015 − 2019 (not on target)
Deficit to target
2015 − 2019 (on target)

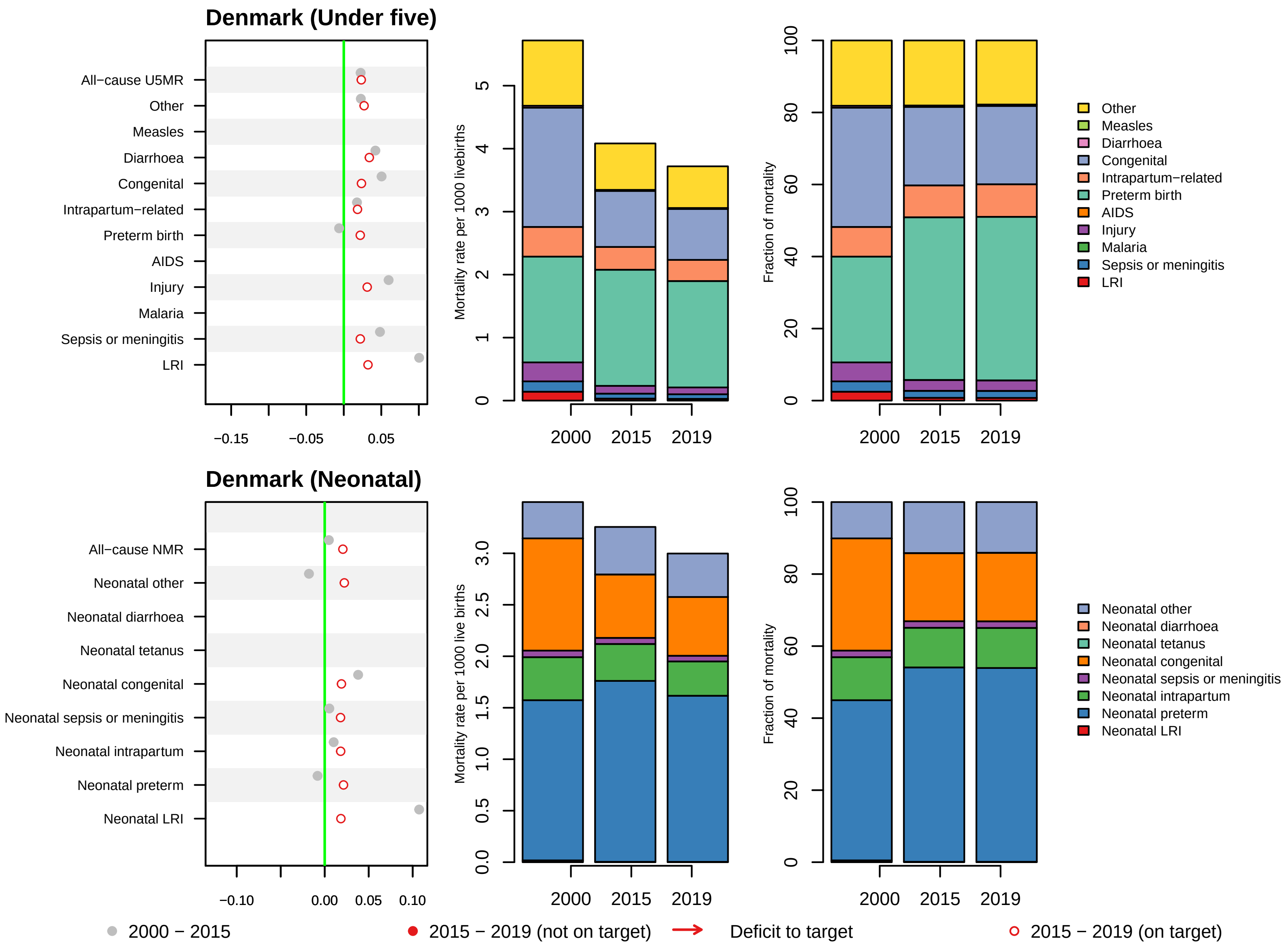

Denmark (Under five)
All−cause U5MR
Other
Measles
Diarrhoea
Congenital
Intrapartum−related
Preterm birth
AIDS
Injury
Malaria
Sepsis or meningitis
LRI
−0.15
−0.05
0.05
Mortality rate per 1000 livebirths
Fraction of mortality
2000
2015
2019
Other
Measles
Diarrhoea
Congenital
Intrapartum−related
Preterm birth
AIDS
Injury
Malaria
Sepsis or meningitis
LRI
Denmark (Neonatal)
All−cause NMR
Neonatal other
Neonatal diarrhoea
Neonatal tetanus
Neonatal congenital
Neonatal sepsis or meningitis
Neonatal intrapartum
Neonatal preterm
Neonatal LRI
−0.10
0.00
0.05
0.10
Mortality rate per 1000 live births
Neonatal other
Neonatal diarrhoea
Neonatal tetanus
Neonatal congenital
Neonatal sepsis or meningitis
Neonatal intrapartum
Neonatal preterm
Neonatal LRI
2000 − 2015
2015 − 2019 (not on target)
Deficit to target
2015 − 2019 (on target)

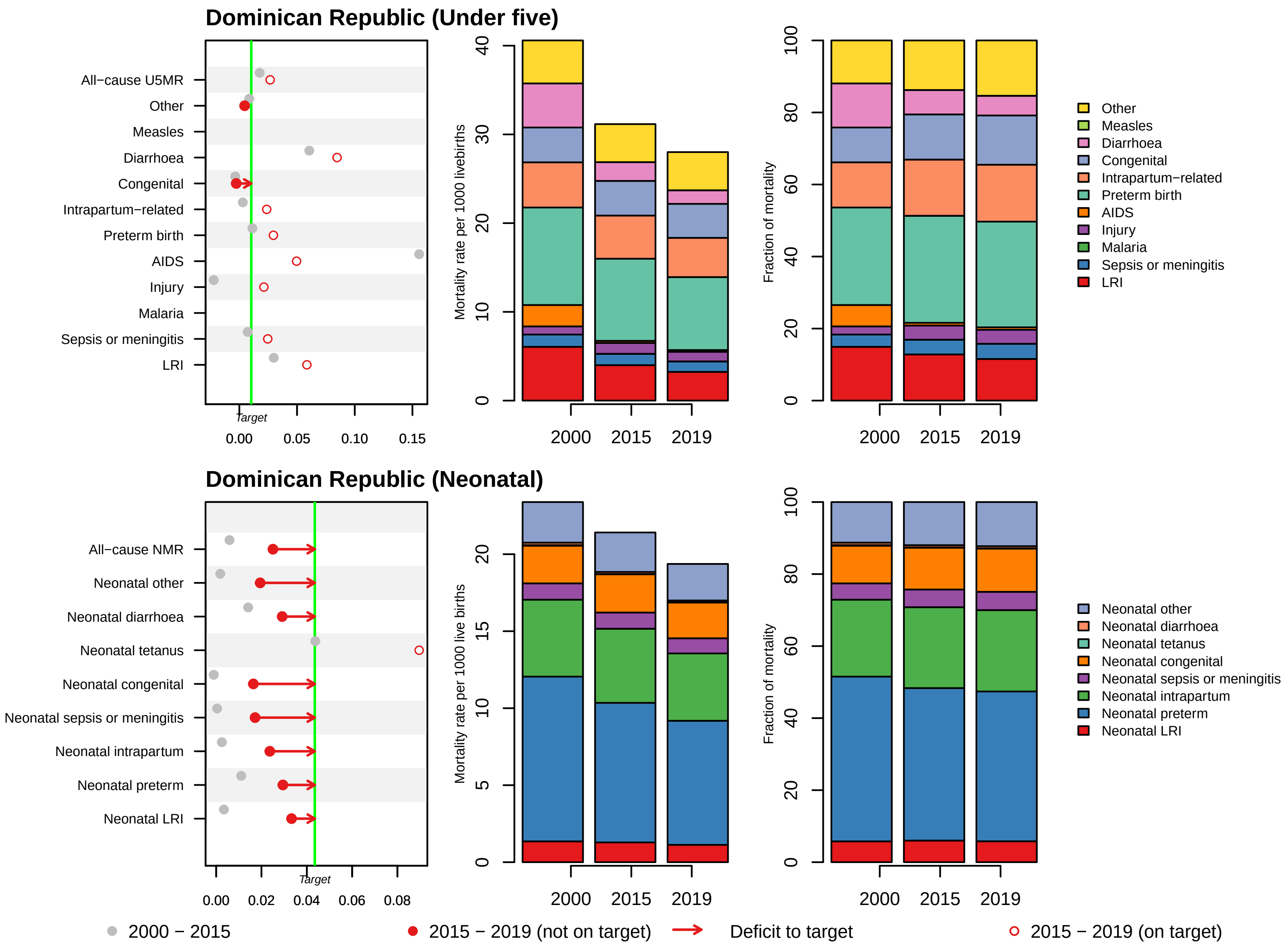

Dominican Republic (Under five)
All−cause U5MR
Other
Measles
Diarrhoea
Congenital
Intrapartum−related
Preterm birth
AIDS
Injury
Malaria
Sepsis or meningitis
LRI
Target
0.00 0.05 0.10 0.15
Mortality rate per 1000 livebirths
0 10 20 30 40
2000 2015 2019
Fraction of mortality
0 20 40 60 80 100
2000 2015 2019
Other
Measles
Diarrhoea
Congenital
Intrapartum−related
Preterm birth
AIDS
Injury
Malaria
Sepsis or meningitis
LRI
Dominican Republic (Neonatal)
All−cause NMR
Neonatal other
Neonatal diarrhoea
Neonatal tetanus
Neonatal congenital
Neonatal sepsis or meningitis
Neonatal intrapartum
Neonatal preterm
Neonatal LRI
Target
0.00 0.02 0.04 0.06 0.08
Mortality rate per 1000 live births
0 5 10 15 20
2000 2015 2019
Fraction of mortality
0 20 40 60 80 100
2000 2015 2019
Neonatal other
Neonatal diarrhoea
Neonatal tetanus
Neonatal congenital
Neonatal sepsis or meningitis
Neonatal intrapartum
Neonatal preterm
Neonatal LRI
2000 − 2015
2015 − 2019 (not on target)
Deficit to target
2015 − 2019 (on target)

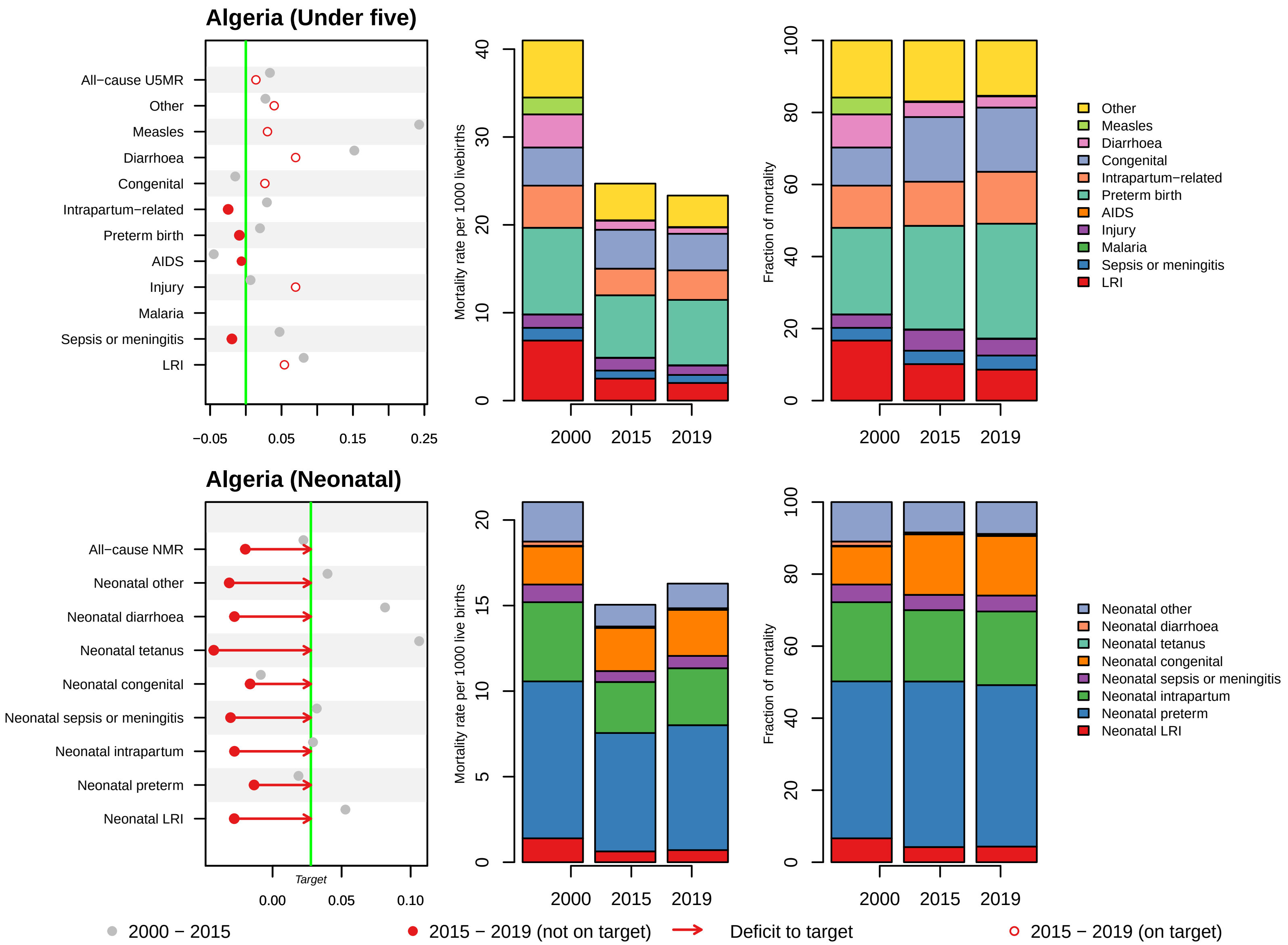
Algeria (Under five)
All−cause U5MR
Other
Measles
Diarrhoea
Congenital
Intrapartum−related
Preterm birth
AIDS
Injury
Malaria
Sepsis or meningitis
LRI
−0.05
0.05
0.15
0.25
Mortality rate per 1000 livebirths
Fraction of mortality
2000
2015
2019
Other
Measles
Diarrhoea
Congenital
Intrapartum−related
Preterm birth
AIDS
Injury
Malaria
Sepsis or meningitis
LRI
Algeria (Neonatal)
All−cause NMR
Neonatal other
Neonatal diarrhoea
Neonatal tetanus
Neonatal congenital
Neonatal sepsis or meningitis
Neonatal intrapartum
Neonatal preterm
Neonatal LRI
Target
0.00
0.05
0.10
Mortality rate per 1000 live births
Fraction of mortality
Neonatal other
Neonatal diarrhoea
Neonatal tetanus
Neonatal congenital
Neonatal sepsis or meningitis
Neonatal intrapartum
Neonatal preterm
Neonatal LRI
2000 − 2015
2015 − 2019 (not on target)
Deficit to target
2015 − 2019 (on target)

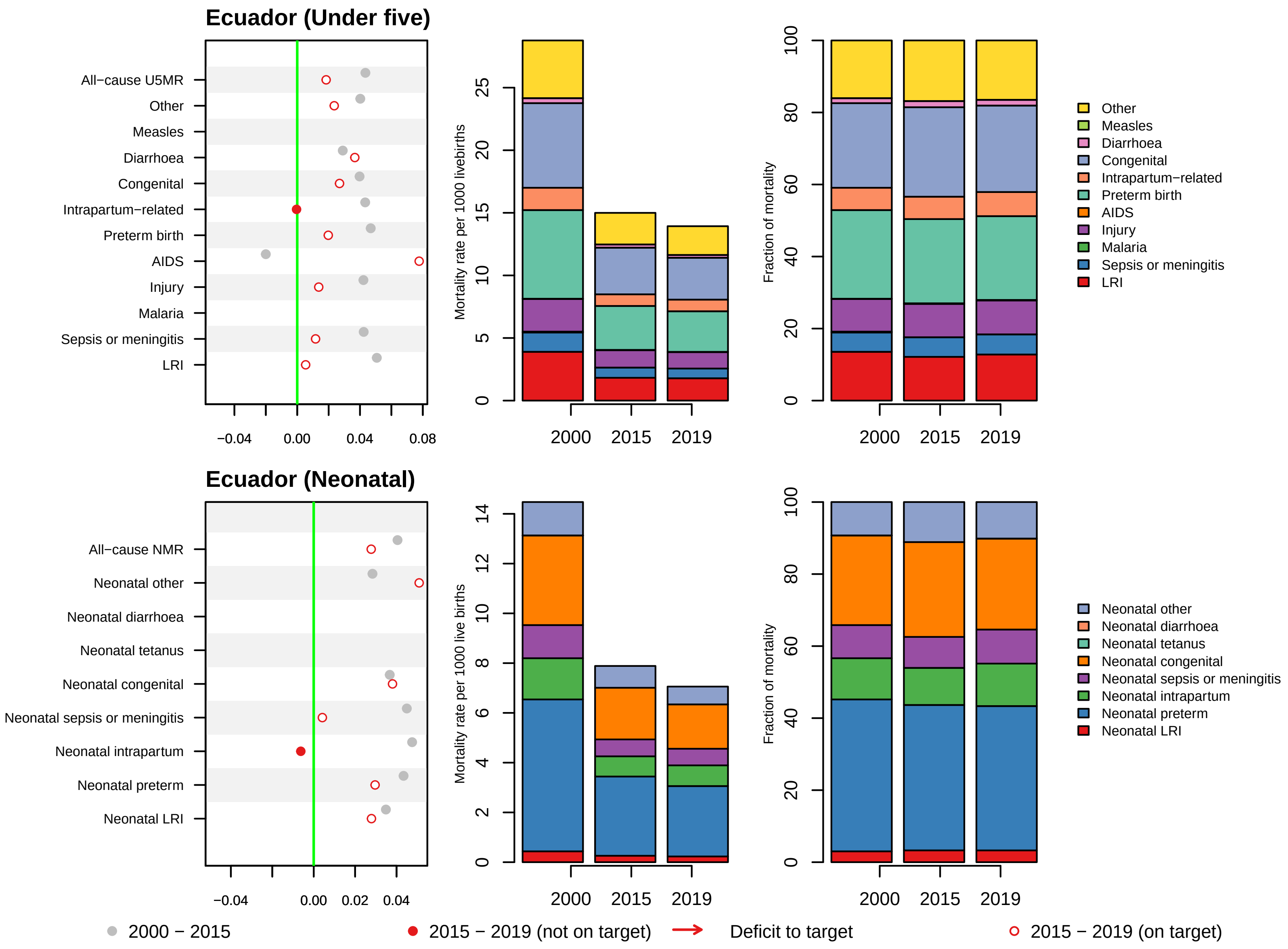

Ecuador (Under five)
All−cause U5MR
Other
Measles
Diarrhoea
Congenital
Intrapartum−related
Preterm birth
AIDS
Injury
Malaria
Sepsis or meningitis
LRI
−0.04
0.00
0.04
0.08
Mortality rate per 1000 livebirths
Fraction of mortality
2000
2015
2019
Other
Measles
Diarrhoea
Congenital
Intrapartum−related
Preterm birth
AIDS
Injury
Malaria
Sepsis or meningitis
LRI
Ecuador (Neonatal)
All−cause NMR
Neonatal other
Neonatal diarrhoea
Neonatal tetanus
Neonatal congenital
Neonatal sepsis or meningitis
Neonatal intrapartum
Neonatal preterm
Neonatal LRI
−0.04
0.00
0.02
0.04
Mortality rate per 1000 live births
Fraction of mortality
2000
2015
2019
Neonatal other
Neonatal diarrhoea
Neonatal tetanus
Neonatal congenital
Neonatal sepsis or meningitis
Neonatal intrapartum
Neonatal preterm
Neonatal LRI
2000 − 2015
2015 − 2019 (not on target)
Deficit to target
2015 − 2019 (on target)

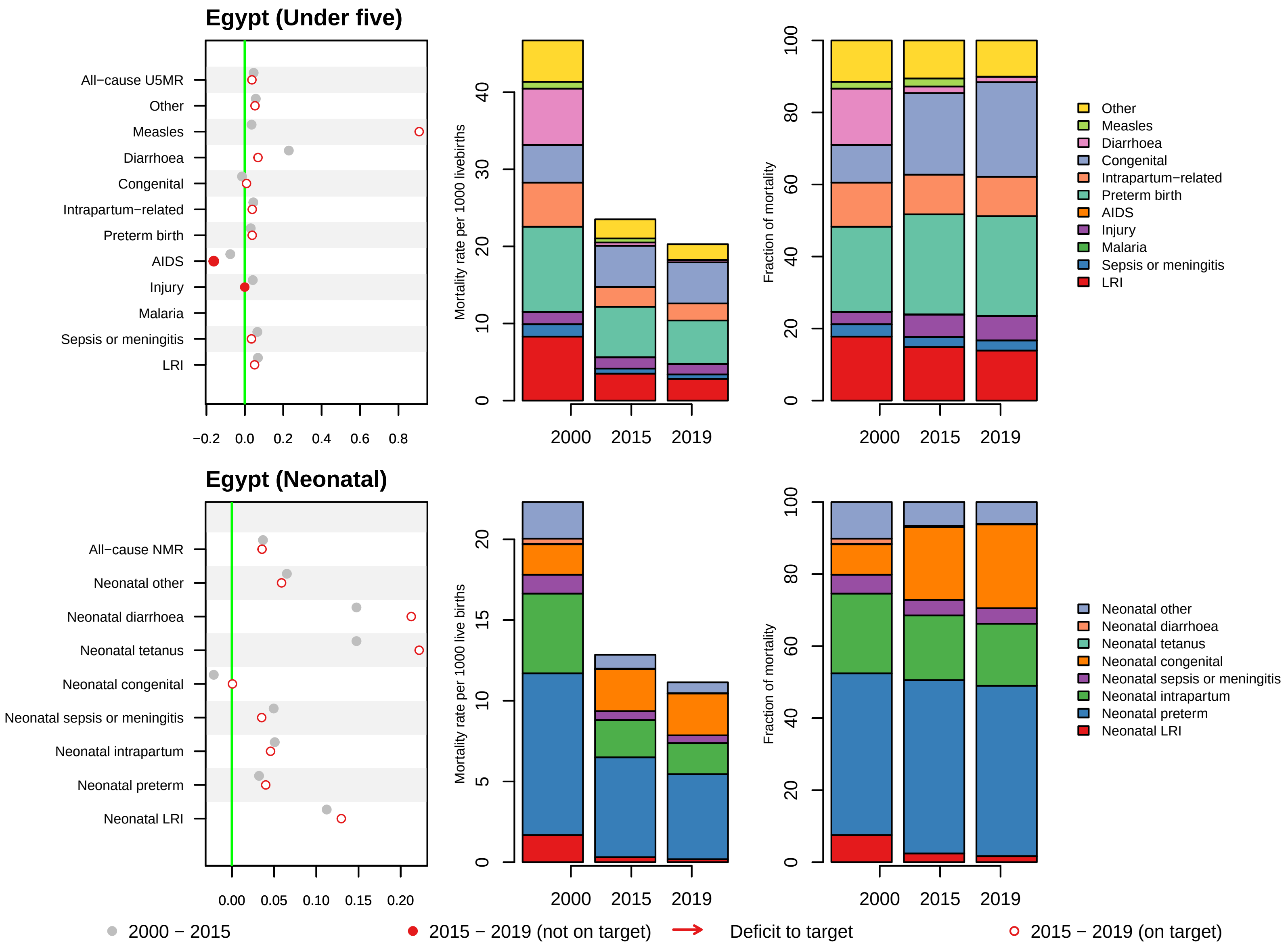

Egypt (Under five)
All−cause U5MR
Other
Measles
Diarrhoea
Congenital
Intrapartum−related
Preterm birth
AIDS
Injury
Malaria
Sepsis or meningitis
LRI
Mortality rate per 1000 livebirths
Fraction of mortality
2000
2015
2019
Egypt (Neonatal)
All−cause NMR
Neonatal other
Neonatal diarrhoea
Neonatal tetanus
Neonatal congenital
Neonatal sepsis or meningitis
Neonatal intrapartum
Neonatal preterm
Neonatal LRI
Mortality rate per 1000 live births
2000 − 2015
2015 − 2019 (not on target)
Deficit to target
2015 − 2019 (on target)

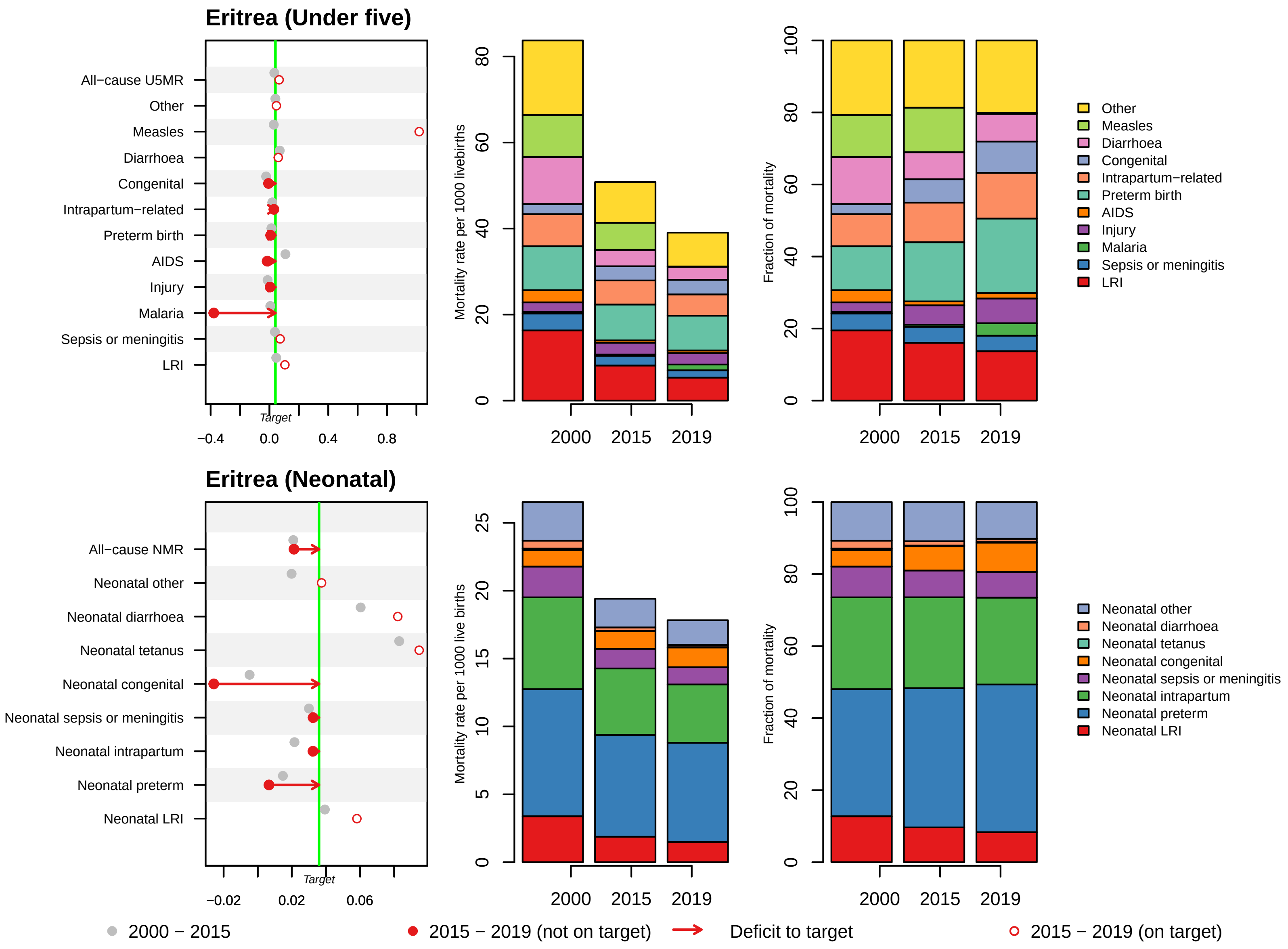

Eritrea (Under five)
All−cause U5MR
Other
Measles
Diarrhoea
Congenital
Intrapartum−related
Preterm birth
AIDS
Injury
Malaria
Sepsis or meningitis
LRI
Target
−0.4
0.0
0.4
0.8
Mortality rate per 1000 livebirths
Fraction of mortality
2000
2015
2019
Other
Measles
Diarrhoea
Congenital
Intrapartum−related
Preterm birth
AIDS
Injury
Malaria
Sepsis or meningitis
LRI
Eritrea (Neonatal)
All−cause NMR
Neonatal other
Neonatal diarrhoea
Neonatal tetanus
Neonatal congenital
Neonatal sepsis or meningitis
Neonatal intrapartum
Neonatal preterm
Neonatal LRI
−0.02
0.02
0.06
Mortality rate per 1000 live births
Neonatal other
Neonatal diarrhoea
Neonatal tetanus
Neonatal congenital
Neonatal sepsis or meningitis
Neonatal intrapartum
Neonatal preterm
Neonatal LRI
2000 − 2015
2015 − 2019 (not on target)
Deficit to target
2015 − 2019 (on target)

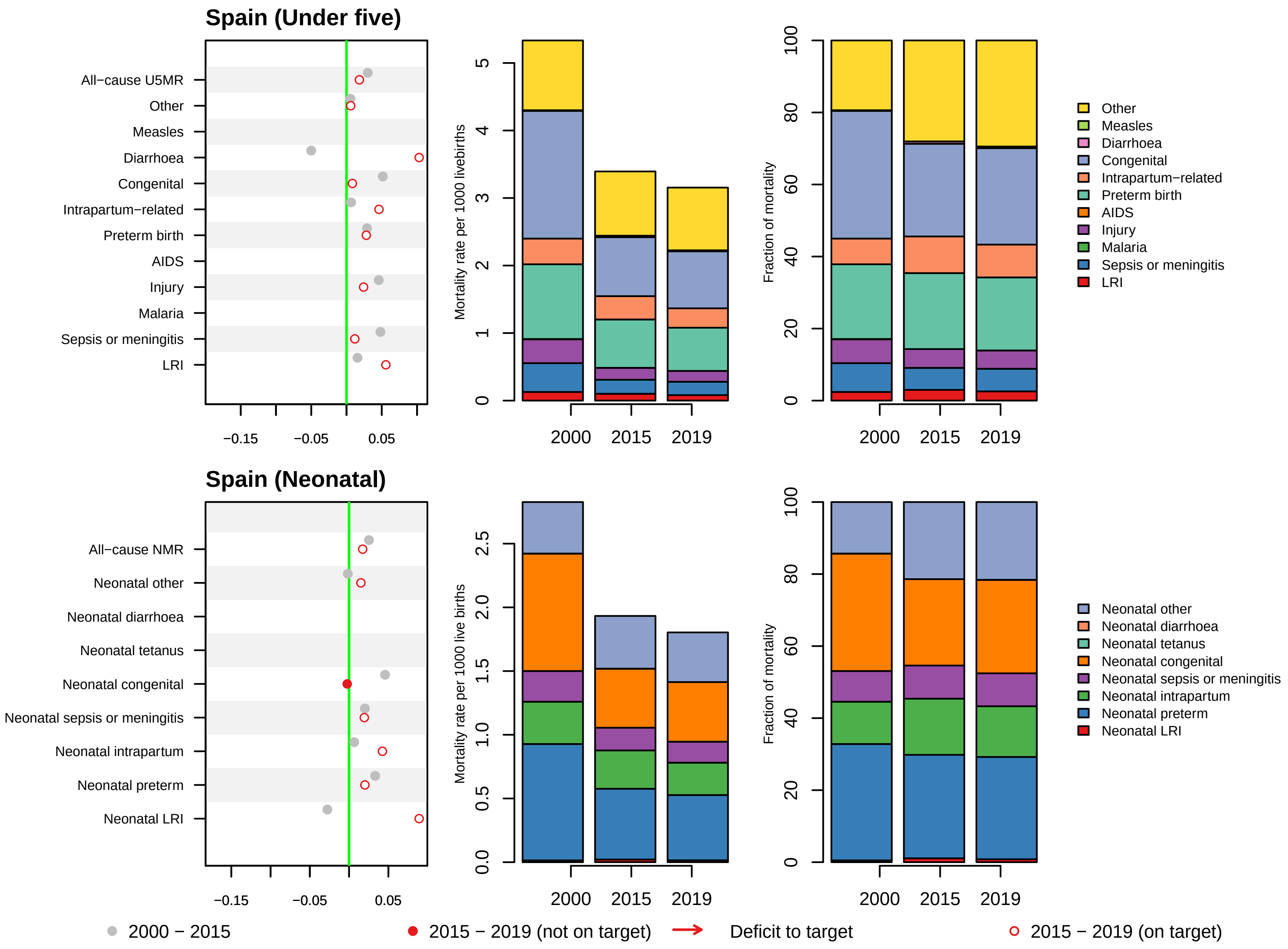
Spain (Under five)
All−cause U5MR
Other
Measles
Diarrhoea
Congenital
Intrapartum−related
Preterm birth
AIDS
Injury
Malaria
Sepsis or meningitis
LRI
−0.15
−0.05
0.05
Mortality rate per 1000 livebirths
0
1
2
3
4
5
2000
2015
2019
Fraction of mortality
0
20
40
60
80
100
Other
Measles
Diarrhoea
Congenital
Intrapartum−related
Preterm birth
AIDS
Injury
Malaria
Sepsis or meningitis
LRI
Spain (Neonatal)
All−cause NMR
Neonatal other
Neonatal diarrhoea
Neonatal tetanus
Neonatal congenital
Neonatal sepsis or meningitis
Neonatal intrapartum
Neonatal preterm
Neonatal LRI
Mortality rate per 1000 live births
0.0
0.5
1.0
1.5
2.0
2.5
Neonatal other
Neonatal diarrhoea
Neonatal tetanus
Neonatal congenital
Neonatal sepsis or meningitis
Neonatal intrapartum
Neonatal preterm
Neonatal LRI
2000 − 2015
2015 − 2019 (not on target)
Deficit to target
2015 − 2019 (on target)

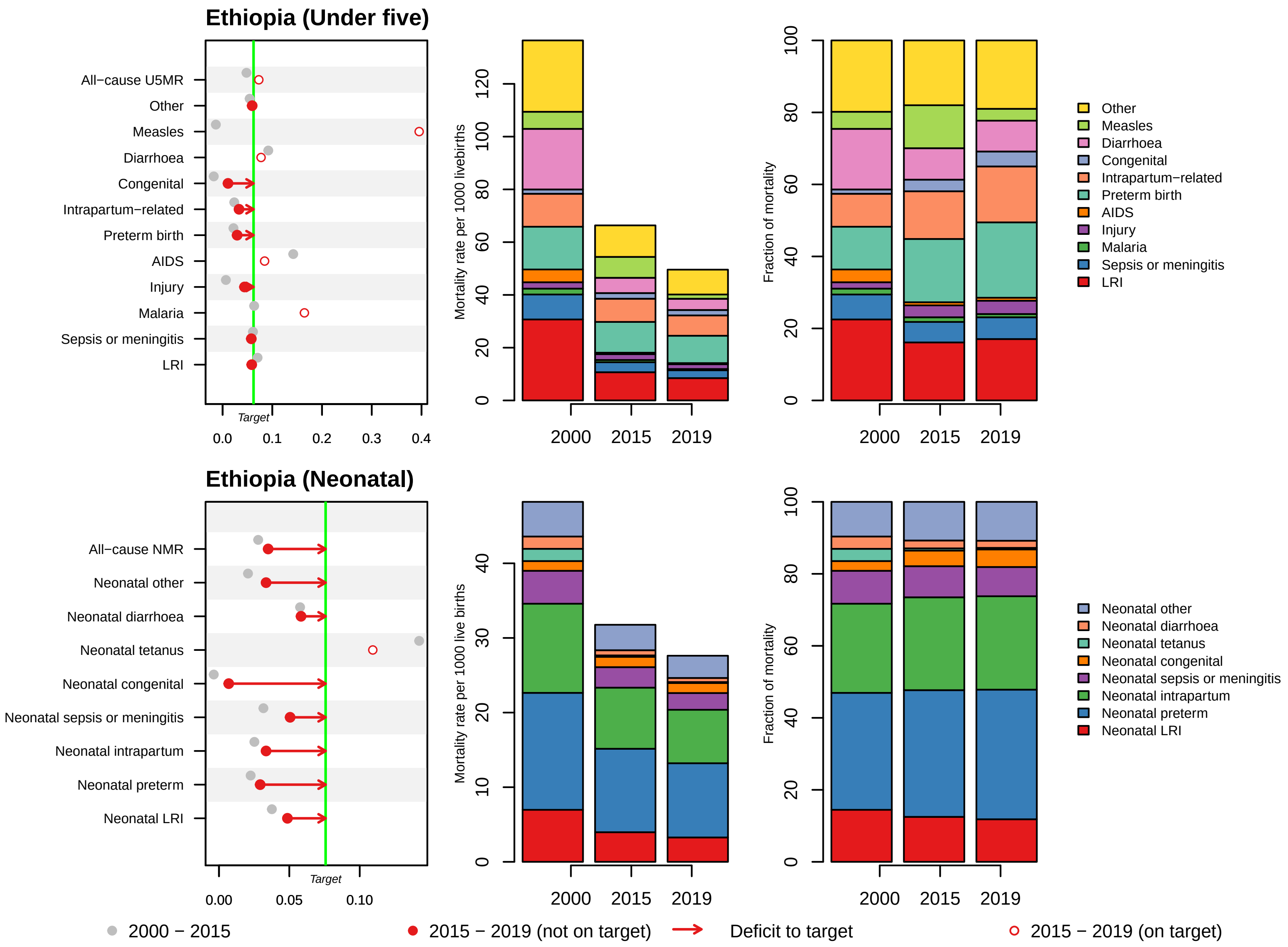

Ethiopia (Under five)
All−cause U5MR
Other
Measles
Diarrhoea
Congenital
Intrapartum−related
Preterm birth
AIDS
Injury
Malaria
Sepsis or meningitis
LRI
Target
0.0
0.1
0.2
0.3
0.4
Mortality rate per 1000 livebirths
0
20
40
60
80
100
120
2000
2015
2019
Fraction of mortality
Other
Measles
Diarrhoea
Congenital
Intrapartum−related
Preterm birth
AIDS
Injury
Malaria
Sepsis or meningitis
LRI
Ethiopia (Neonatal)
All−cause NMR
Neonatal other
Neonatal diarrhoea
Neonatal tetanus
Neonatal congenital
Neonatal sepsis or meningitis
Neonatal intrapartum
Neonatal preterm
Neonatal LRI
0.00
0.05
0.10
Mortality rate per 1000 live births
10
30
Neonatal other
Neonatal diarrhoea
Neonatal tetanus
Neonatal congenital
Neonatal sepsis or meningitis
Neonatal intrapartum
Neonatal preterm
Neonatal LRI
2000 − 2015
2015 − 2019 (not on target)
Deficit to target
2015 − 2019 (on target)

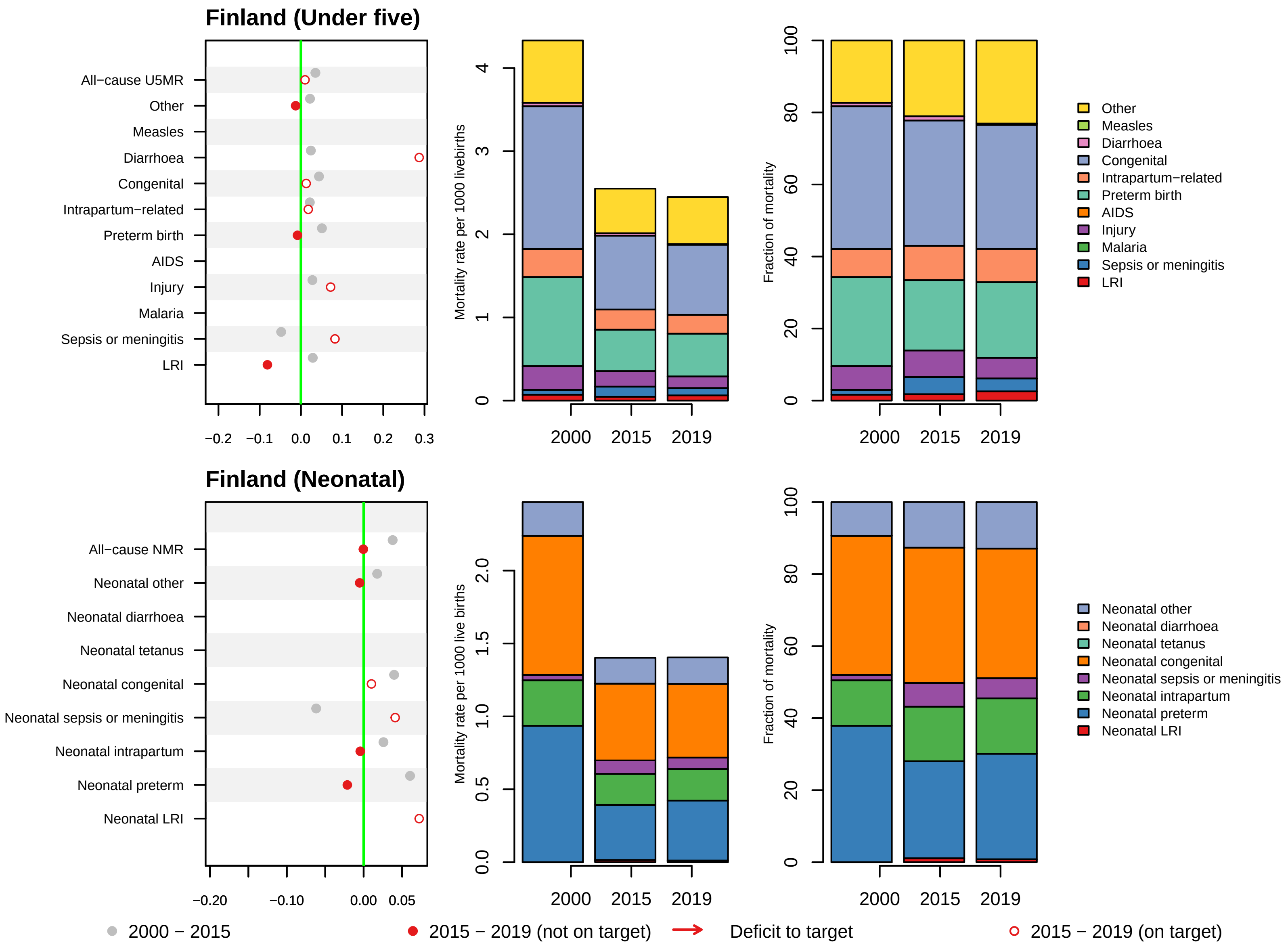
Finland (Under five)
All−cause U5MR
Other
Measles
Diarrhoea
Congenital
Intrapartum−related
Preterm birth
AIDS
Injury
Malaria
Sepsis or meningitis
LRI
−0.2
−0.1
0.0
0.1
0.2
0.3
Mortality rate per 1000 livebirths
2000
2015
2019
Fraction of mortality
Other
Measles
Diarrhoea
Congenital
Intrapartum−related
Preterm birth
AIDS
Injury
Malaria
Sepsis or meningitis
LRI
Finland (Neonatal)
All−cause NMR
Neonatal other
Neonatal diarrhoea
Neonatal tetanus
Neonatal congenital
Neonatal sepsis or meningitis
Neonatal intrapartum
Neonatal preterm
Neonatal LRI
−0.20
−0.10
0.00
0.05
Mortality rate per 1000 live births
Fraction of mortality
Neonatal other
Neonatal diarrhoea
Neonatal tetanus
Neonatal congenital
Neonatal sepsis or meningitis
Neonatal intrapartum
Neonatal preterm
Neonatal LRI
2000 − 2015
2015 − 2019 (not on target)
Deficit to target
2015 − 2019 (on target)

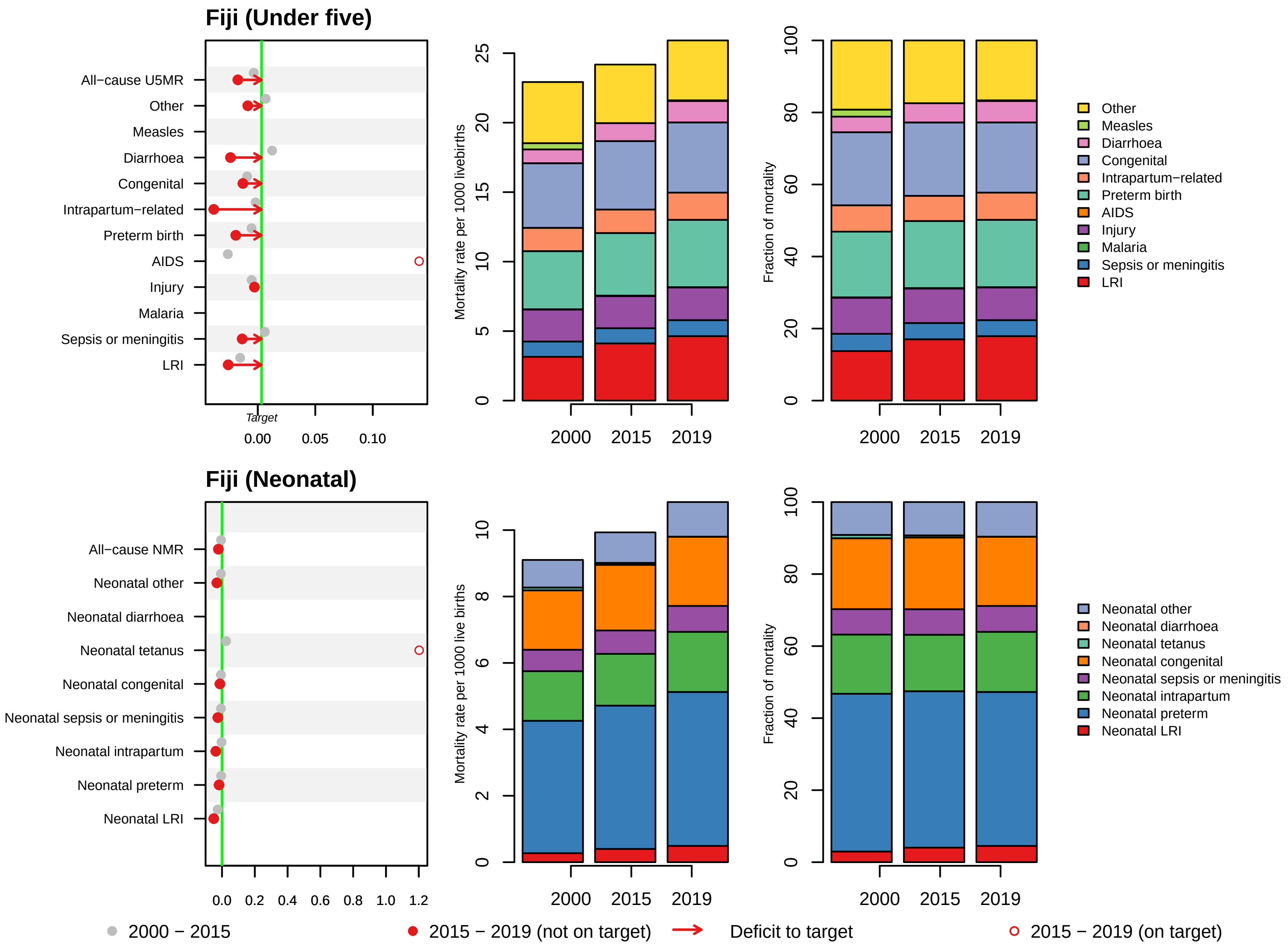
Fiji (Under five)
All−cause U5MR
Other
Measles
Diarrhoea
Congenital
Intrapartum−related
Preterm birth
AIDS
Injury
Malaria
Sepsis or meningitis
LRI
Target
0.00
0.05
0.10
Mortality rate per 1000 livebirths
Fraction of mortality
2000
2015
2019
Other
Measles
Diarrhoea
Congenital
Intrapartum−related
Preterm birth
AIDS
Injury
Malaria
Sepsis or meningitis
LRI
Fiji (Neonatal)
All−cause NMR
Neonatal other
Neonatal diarrhoea
Neonatal tetanus
Neonatal congenital
Neonatal sepsis or meningitis
Neonatal intrapartum
Neonatal preterm
Neonatal LRI
0.0
0.2
0.4
0.6
0.8
1.0
1.2
Mortality rate per 1000 live births
Fraction of mortality
Neonatal other
Neonatal diarrhoea
Neonatal tetanus
Neonatal congenital
Neonatal sepsis or meningitis
Neonatal intrapartum
Neonatal preterm
Neonatal LRI
2000 − 2015
2015 − 2019 (not on target)
Deficit to target
2015 − 2019 (on target)

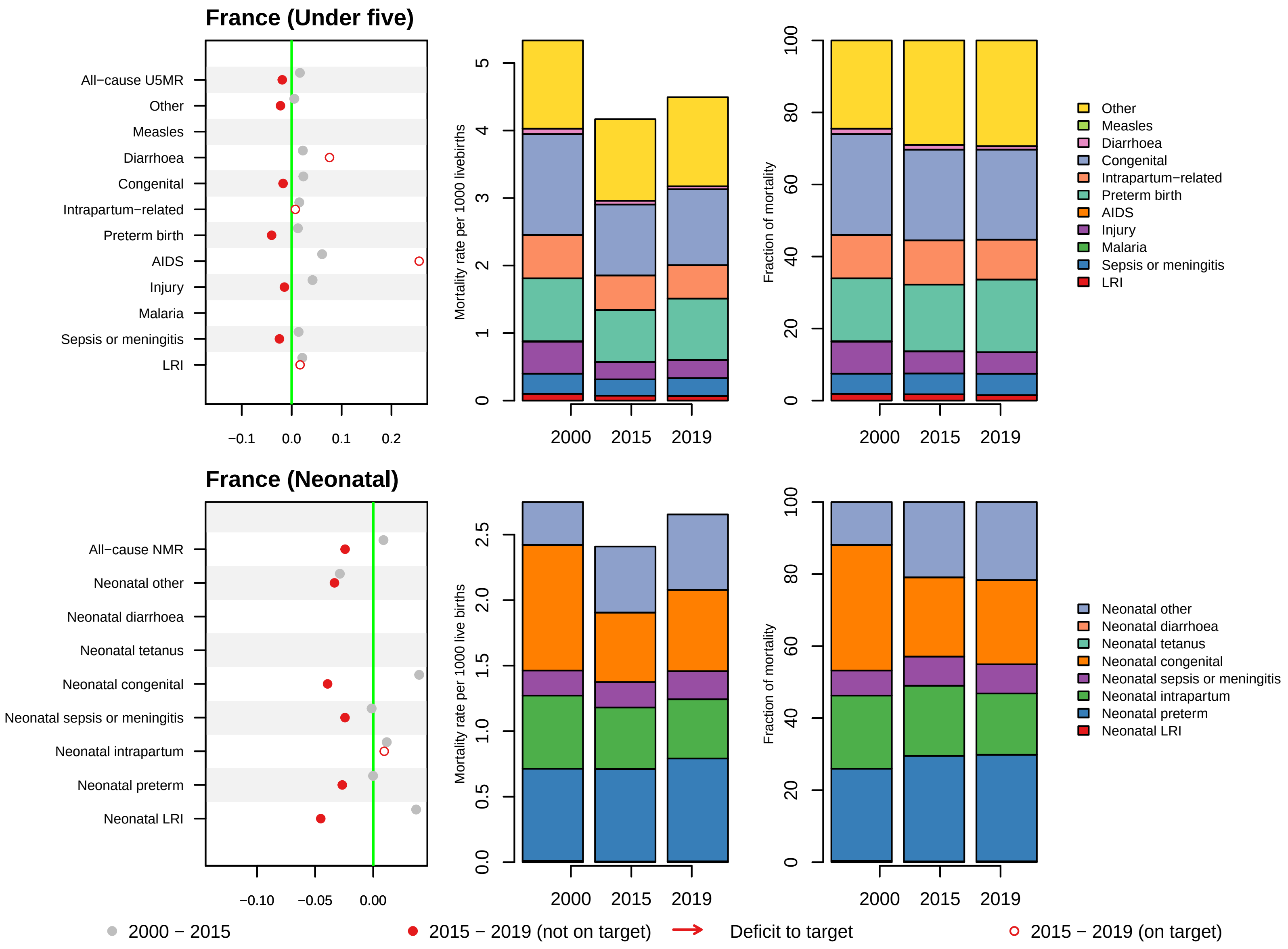
France (Under five)
All−cause U5MR
Other
Measles
Diarrhoea
Congenital
Intrapartum−related
Preterm birth
AIDS
Injury
Malaria
Sepsis or meningitis
LRI
−0.1
0.0
0.1
0.2
Mortality rate per 1000 livebirths
Fraction of mortality
2000
2015
2019
Other
Measles
Diarrhoea
Congenital
Intrapartum−related
Preterm birth
AIDS
Injury
Malaria
Sepsis or meningitis
LRI
France (Neonatal)
All−cause NMR
Neonatal other
Neonatal diarrhoea
Neonatal tetanus
Neonatal congenital
Neonatal sepsis or meningitis
Neonatal intrapartum
Neonatal preterm
Neonatal LRI
−0.10
−0.05
0.00
Mortality rate per 1000 live births
Fraction of mortality
2000
2015
2019
Neonatal other
Neonatal diarrhoea
Neonatal tetanus
Neonatal congenital
Neonatal sepsis or meningitis
Neonatal intrapartum
Neonatal preterm
Neonatal LRI
2000 − 2015
2015 − 2019 (not on target)
Deficit to target
2015 − 2019 (on target)

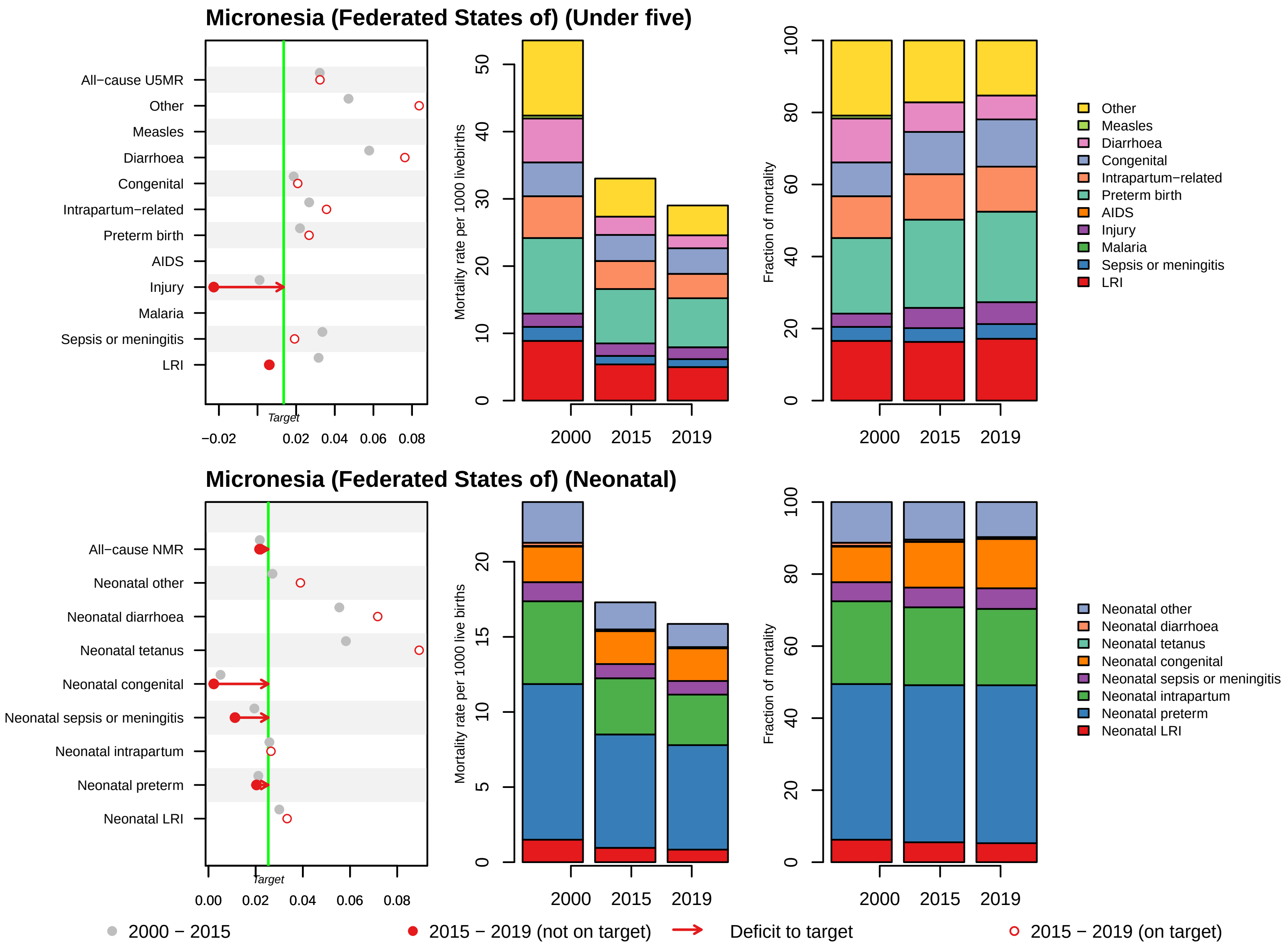
Micronesia (Federated States of) (Under five)
All−cause U5MR
Other
Measles
Diarrhoea
Congenital
Intrapartum−related
Preterm birth
AIDS
Injury
Malaria
Sepsis or meningitis
LRI
Target
−0.02
0.02
0.04
0.06
0.08
Mortality rate per 1000 livebirths
0
10
20
30
40
50
2000
2015
2019
Fraction of mortality
0
20
40
60
80
100
Other
Measles
Diarrhoea
Congenital
Intrapartum−related
Preterm birth
AIDS
Injury
Malaria
Sepsis or meningitis
LRI
Micronesia (Federated States of) (Neonatal)
All−cause NMR
Neonatal other
Neonatal diarrhoea
Neonatal tetanus
Neonatal congenital
Neonatal sepsis or meningitis
Neonatal intrapartum
Neonatal preterm
Neonatal LRI
Target
0.00
0.02
0.04
0.06
0.08
Mortality rate per 1000 live births
0
5
10
15
20
Neonatal other
Neonatal diarrhoea
Neonatal tetanus
Neonatal congenital
Neonatal sepsis or meningitis
Neonatal intrapartum
Neonatal preterm
Neonatal LRI
2000 − 2015
2015 − 2019 (not on target)
Deficit to target
2015 − 2019 (on target)

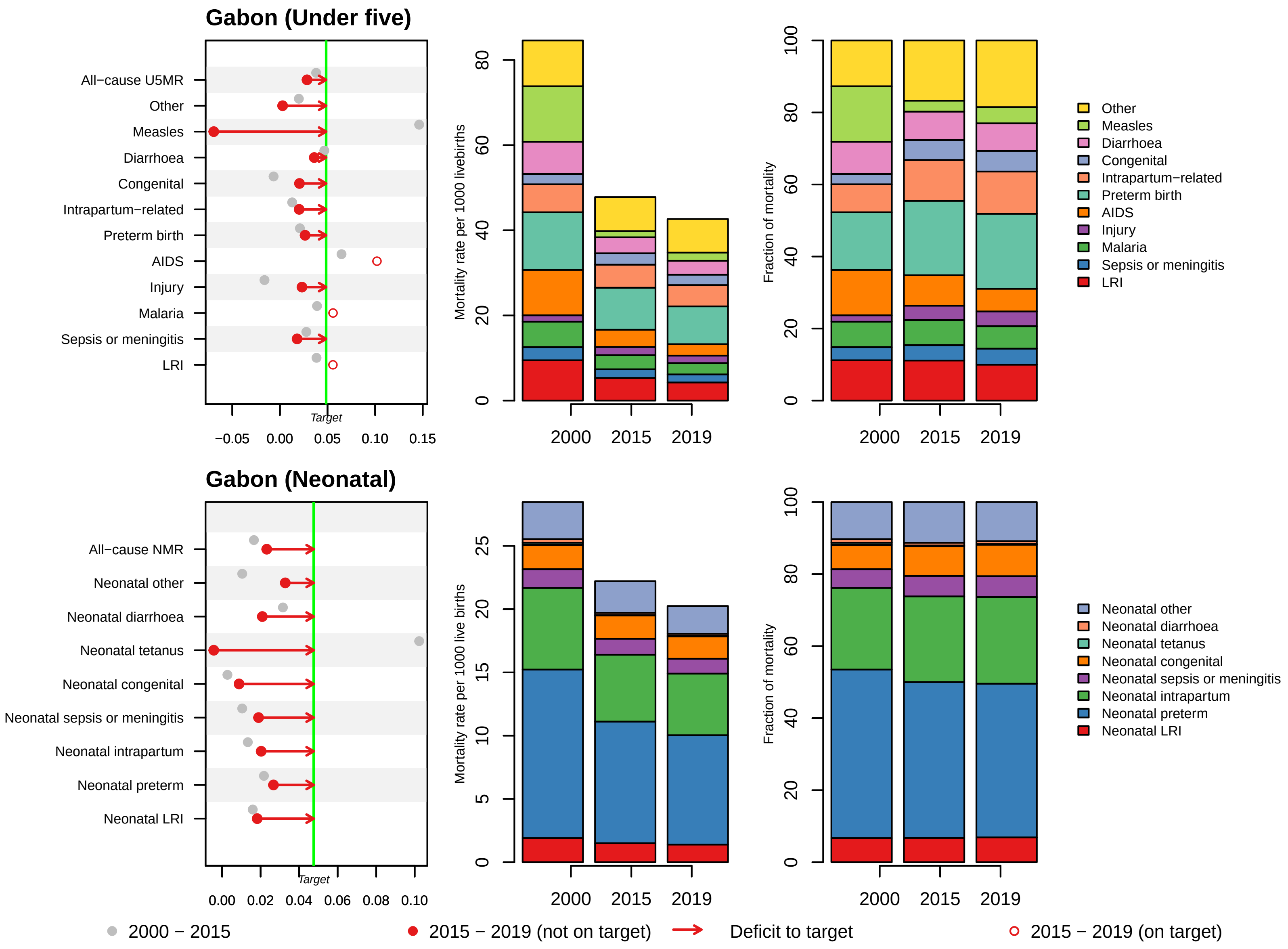

Gabon (Under five)
All−cause U5MR
Other
Measles
Diarrhoea
Congenital
Intrapartum−related
Preterm birth
AIDS
Injury
Malaria
Sepsis or meningitis
LRI
Target
−0.05
0.00
0.05
0.10
0.15
Mortality rate per 1000 livebirths
0
20
40
60
80
2000
2015
2019
Fraction of mortality
0
20
40
60
80
100
Other
Measles
Diarrhoea
Congenital
Intrapartum−related
Preterm birth
AIDS
Injury
Malaria
Sepsis or meningitis
LRI
Gabon (Neonatal)
All−cause NMR
Neonatal other
Neonatal diarrhoea
Neonatal tetanus
Neonatal congenital
Neonatal sepsis or meningitis
Neonatal intrapartum
Neonatal preterm
Neonatal LRI
Target
0.00
0.02
0.04
0.06
0.08
0.10
Mortality rate per 1000 live births
0
5
10
15
20
25
Neonatal other
Neonatal diarrhoea
Neonatal tetanus
Neonatal congenital
Neonatal sepsis or meningitis
Neonatal intrapartum
Neonatal preterm
Neonatal LRI
2000 − 2015
2015 − 2019 (not on target)
Deficit to target
2015 − 2019 (on target)

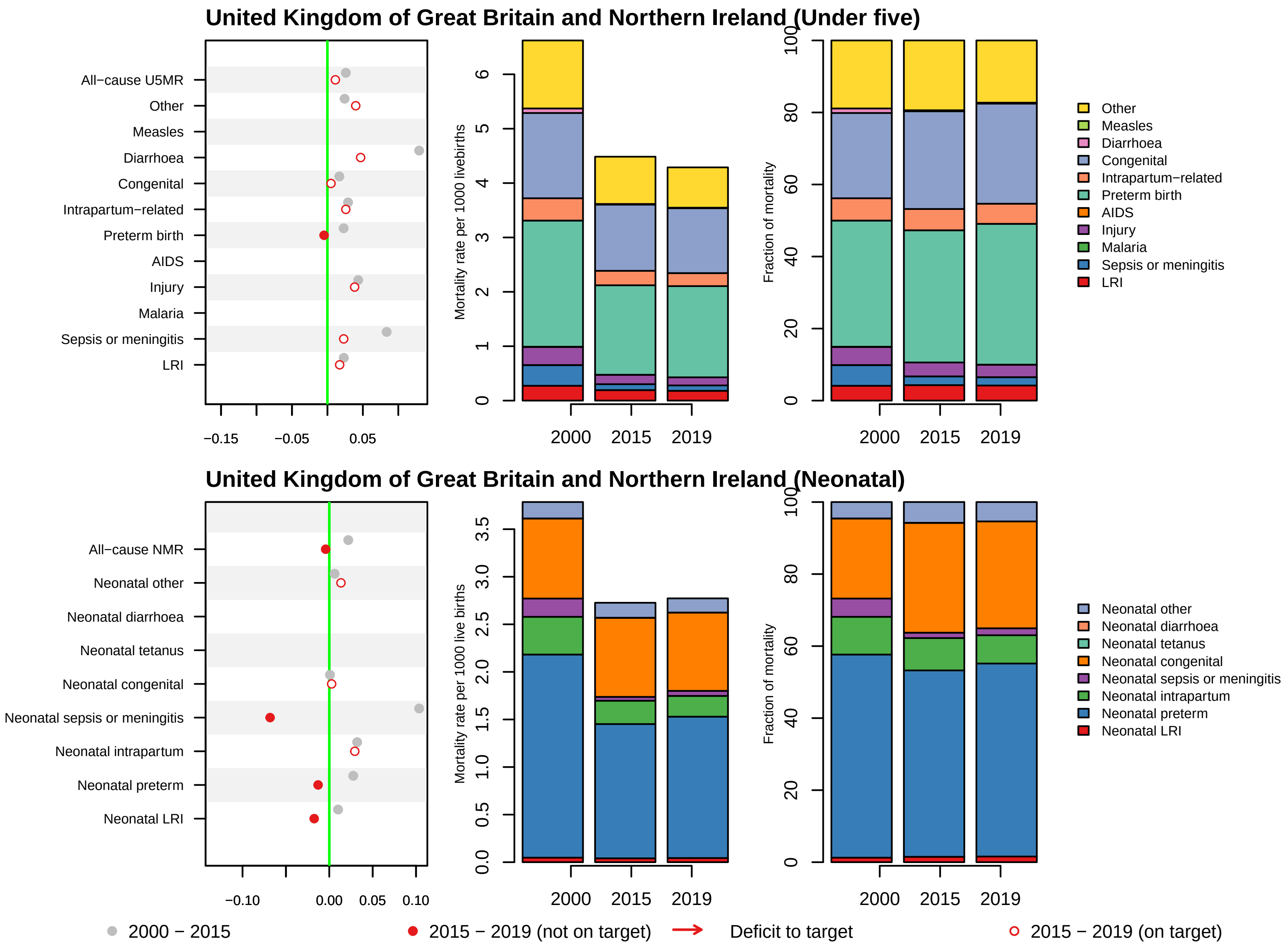
United Kingdom of Great Britain and Northern Ireland (Under five)
All−cause U5MR
Other
Measles
Diarrhoea
Congenital
Intrapartum−related
Preterm birth
AIDS
Injury
Malaria
Sepsis or meningitis
LRI
−0.15
−0.05
0.05
Mortality rate per 1000 livebirths
Fraction of mortality
2000
2015
2019
United Kingdom of Great Britain and Northern Ireland (Neonatal)
All−cause NMR
Neonatal other
Neonatal diarrhoea
Neonatal tetanus
Neonatal congenital
Neonatal sepsis or meningitis
Neonatal intrapartum
Neonatal preterm
Neonatal LRI
−0.10
0.00
0.05
0.10
Mortality rate per 1000 live births
2000 − 2015
2015 − 2019 (not on target)
Deficit to target
2015 − 2019 (on target)

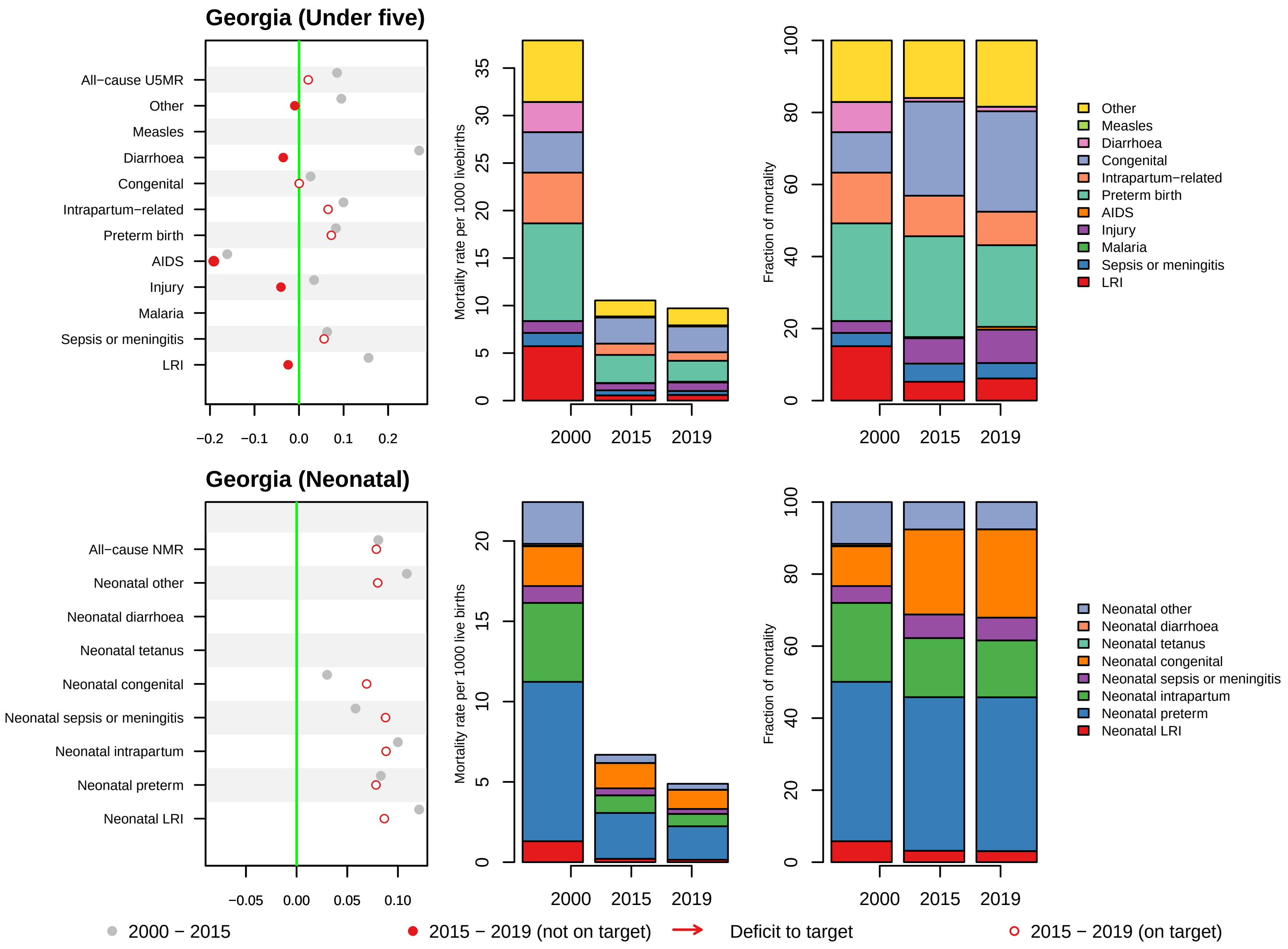

Georgia (Under five)
All−cause U5MR
Other
Measles
Diarrhoea
Congenital
Intrapartum−related
Preterm birth
AIDS
Injury
Malaria
Sepsis or meningitis
LRI
−0.2
−0.1
0.0
0.1
0.2
Mortality rate per 1000 livebirths
2000
2015
2019
Fraction of mortality
Other
Measles
Diarrhoea
Congenital
Intrapartum−related
Preterm birth
AIDS
Injury
Malaria
Sepsis or meningitis
LRI
Georgia (Neonatal)
All−cause NMR
Neonatal other
Neonatal diarrhoea
Neonatal tetanus
Neonatal congenital
Neonatal sepsis or meningitis
Neonatal intrapartum
Neonatal preterm
Neonatal LRI
−0.05
0.00
0.05
0.10
Mortality rate per 1000 live births
Fraction of mortality
Neonatal other
Neonatal diarrhoea
Neonatal tetanus
Neonatal congenital
Neonatal sepsis or meningitis
Neonatal intrapartum
Neonatal preterm
Neonatal LRI
2000 − 2015
2015 − 2019 (not on target)
Deficit to target
2015 − 2019 (on target)

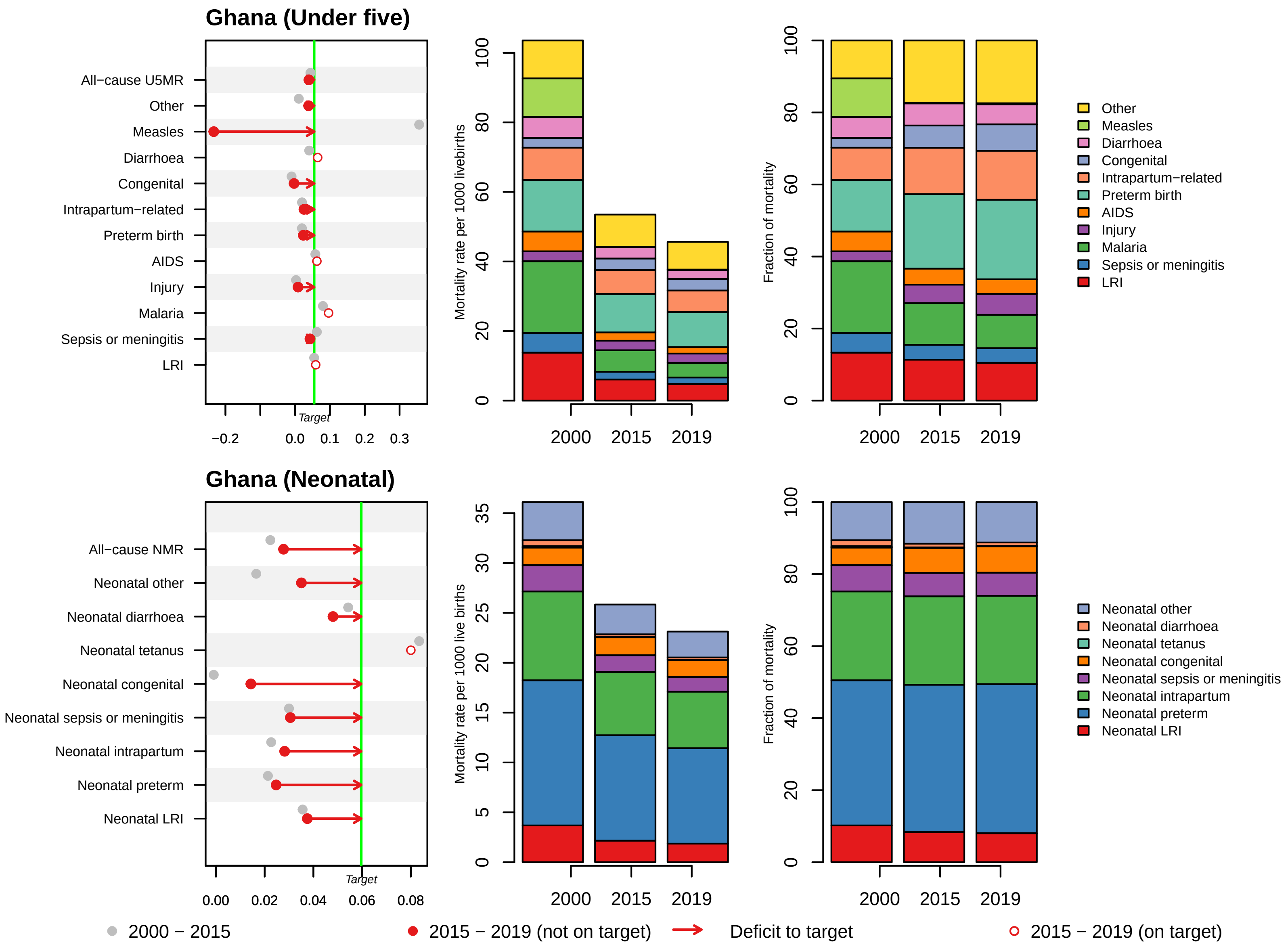

Ghana (Under five)
All−cause U5MR
Other
Measles
Diarrhoea
Congenital
Intrapartum−related
Preterm birth
AIDS
Injury
Malaria
Sepsis or meningitis
LRI
Target
−0.2
0.0
0.1
0.2
0.3
Mortality rate per 1000 livebirths
Fraction of mortality
2000
2015
2019
Other
Measles
Diarrhoea
Congenital
Intrapartum−related
Preterm birth
AIDS
Injury
Malaria
Sepsis or meningitis
LRI
Ghana (Neonatal)
All−cause NMR
Neonatal other
Neonatal diarrhoea
Neonatal tetanus
Neonatal congenital
Neonatal sepsis or meningitis
Neonatal intrapartum
Neonatal preterm
Neonatal LRI
0.00
0.02
0.04
0.06
0.08
Mortality rate per 1000 live births
2000 − 2015
2015 − 2019 (not on target)
Deficit to target
2015 − 2019 (on target)

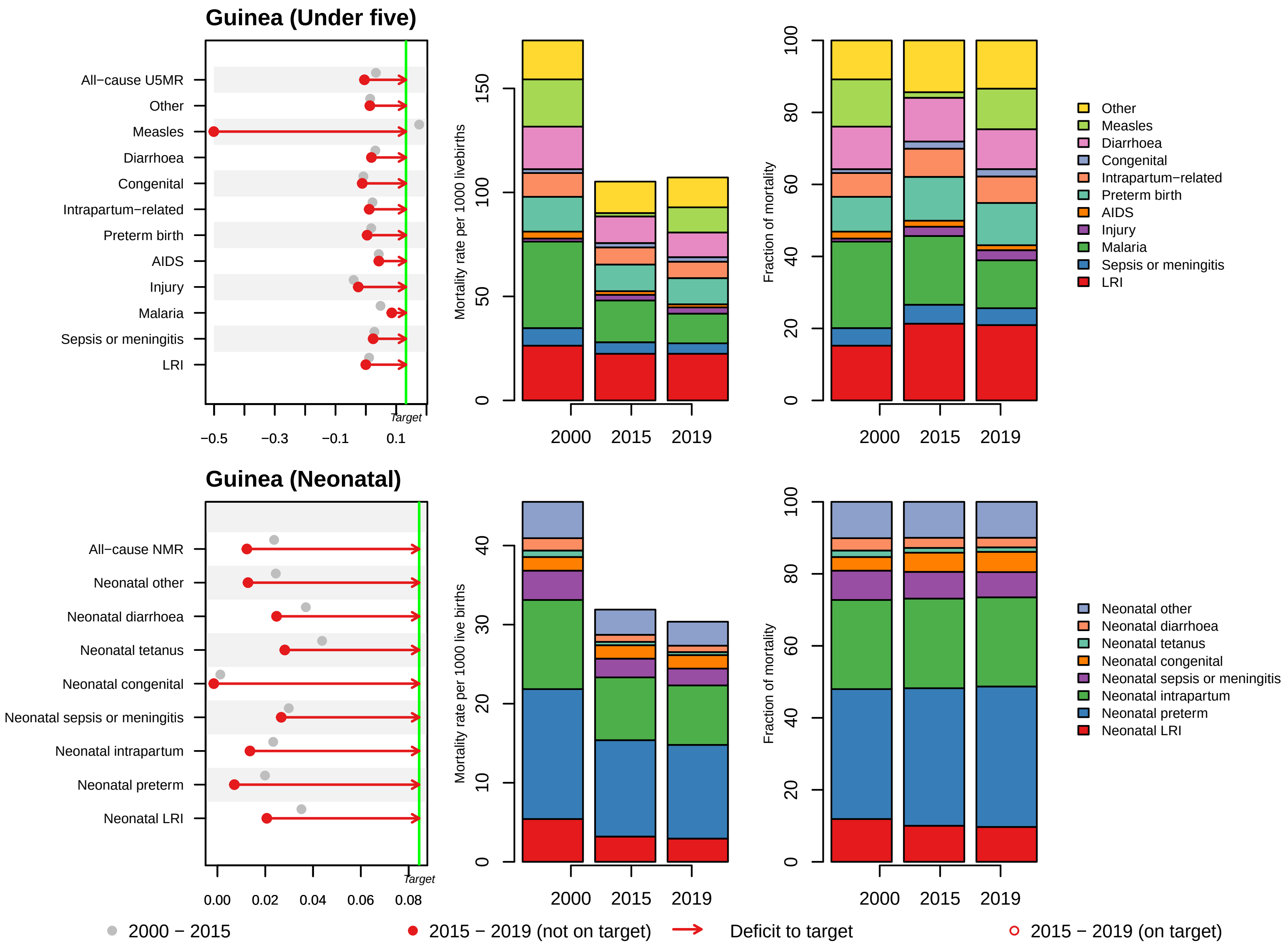
Guinea (Under five)
All−cause U5MR
Other
Measles
Diarrhoea
Congenital
Intrapartum−related
Preterm birth
AIDS
Injury
Malaria
Sepsis or meningitis
LRI
Target
−0.5
−0.3
−0.1
0.1
Mortality rate per 1000 livebirths
0
50
100
150
2000
2015
2019
Fraction of mortality
0
20
40
60
80
100
Other
Measles
Diarrhoea
Congenital
Intrapartum−related
Preterm birth
AIDS
Injury
Malaria
Sepsis or meningitis
LRI
Guinea (Neonatal)
All−cause NMR
Neonatal other
Neonatal diarrhoea
Neonatal tetanus
Neonatal congenital
Neonatal sepsis or meningitis
Neonatal intrapartum
Neonatal preterm
Neonatal LRI
Target
0.00
0.02
0.04
0.06
0.08
Mortality rate per 1000 live births
0
10
20
30
40
Neonatal other
Neonatal diarrhoea
Neonatal tetanus
Neonatal congenital
Neonatal sepsis or meningitis
Neonatal intrapartum
Neonatal preterm
Neonatal LRI
2000 − 2015
2015 − 2019 (not on target)
Deficit to target
2015 − 2019 (on target)

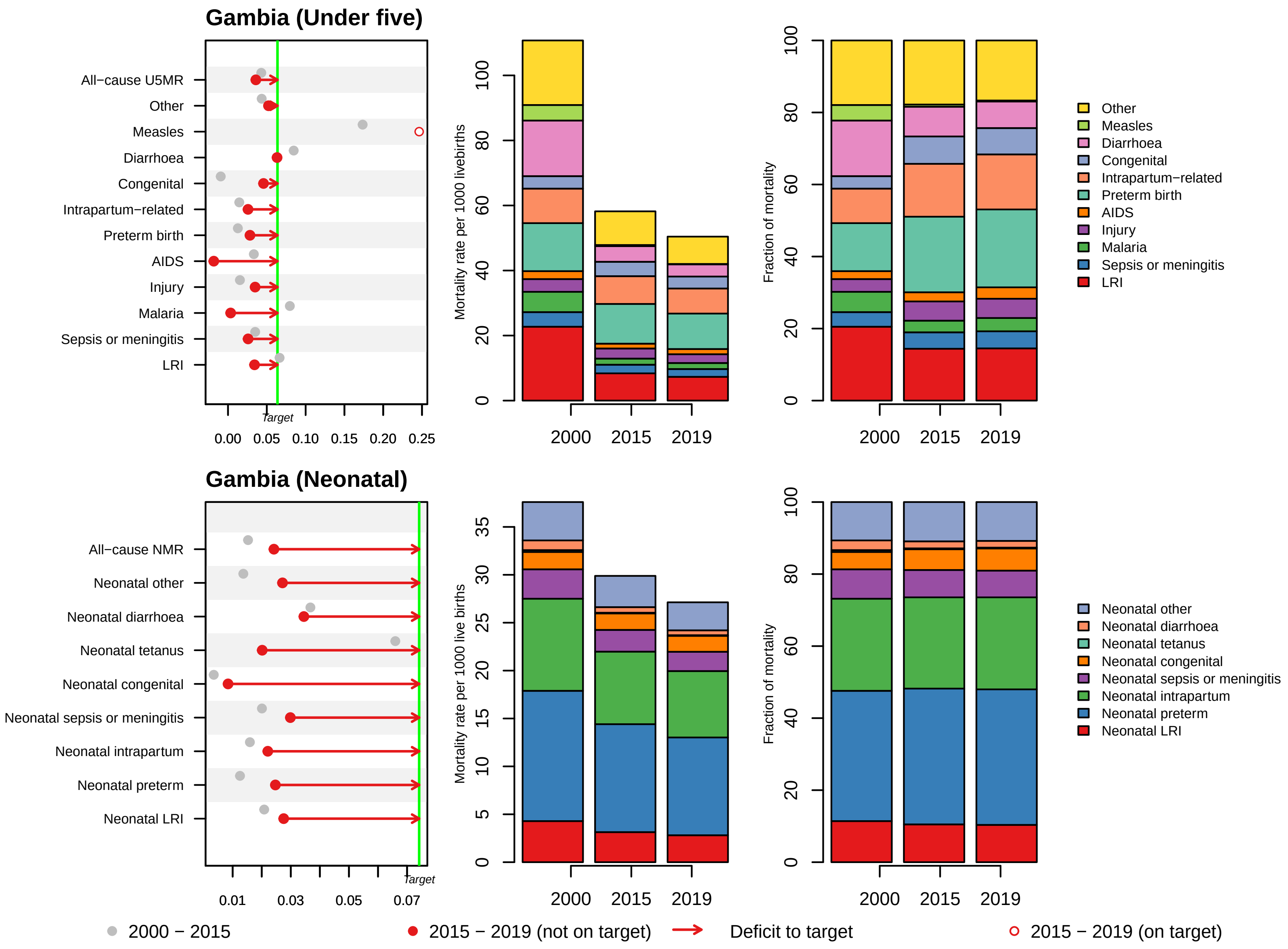

Gambia (Under five)
All−cause U5MR
Other
Measles
Diarrhoea
Congenital
Intrapartum−related
Preterm birth
AIDS
Injury
Malaria
Sepsis or meningitis
LRI
Target
0.00 0.05 0.10 0.15 0.20 0.25
Mortality rate per 1000 livebirths
0 20 40 60 80 100
2000 2015 2019
Fraction of mortality
0 20 40 60 80 100
2000 2015 2019
Other
Measles
Diarrhoea
Congenital
Intrapartum−related
Preterm birth
AIDS
Injury
Malaria
Sepsis or meningitis
LRI
Gambia (Neonatal)
All−cause NMR
Neonatal other
Neonatal diarrhoea
Neonatal tetanus
Neonatal congenital
Neonatal sepsis or meningitis
Neonatal intrapartum
Neonatal preterm
Neonatal LRI
Target
0.01 0.03 0.05 0.07
Mortality rate per 1000 live births
0 5 10 15 20 25 30 35
2000 2015 2019
Fraction of mortality
0 20 40 60 80 100
2000 2015 2019
Neonatal other
Neonatal diarrhoea
Neonatal tetanus
Neonatal congenital
Neonatal sepsis or meningitis
Neonatal intrapartum
Neonatal preterm
Neonatal LRI
2000 − 2015
2015 − 2019 (not on target)
Deficit to target
2015 − 2019 (on target)

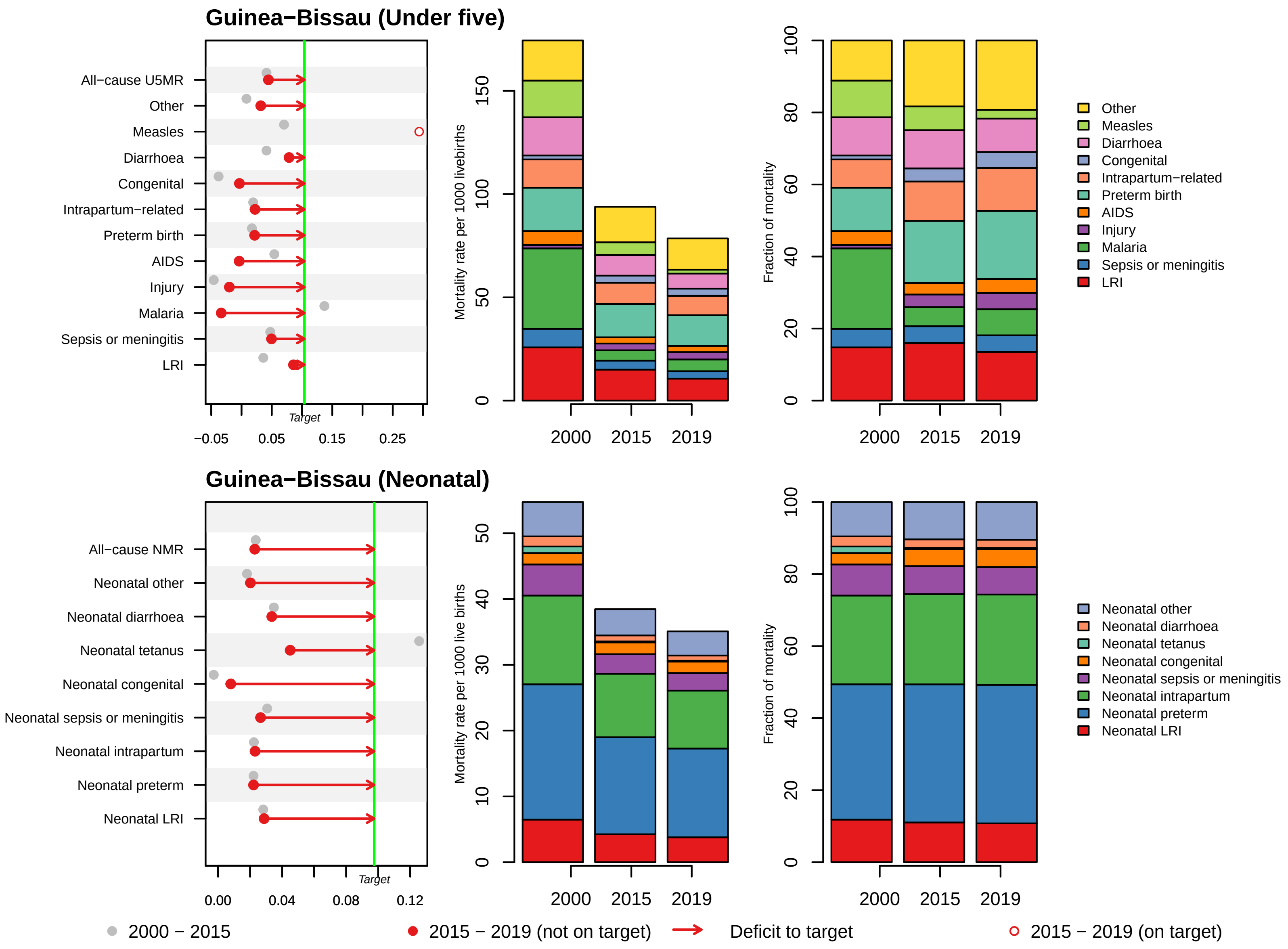
Guinea−Bissau (Under five)
All−cause U5MR
Other
Measles
Diarrhoea
Congenital
Intrapartum−related
Preterm birth
AIDS
Injury
Malaria
Sepsis or meningitis
LRI
Target
−0.05
0.05
0.15
0.25
Mortality rate per 1000 livebirths
Fraction of mortality
2000
2015
2019
Other
Measles
Diarrhoea
Congenital
Intrapartum−related
Preterm birth
AIDS
Injury
Malaria
Sepsis or meningitis
LRI
Guinea−Bissau (Neonatal)
All−cause NMR
Neonatal other
Neonatal diarrhoea
Neonatal tetanus
Neonatal congenital
Neonatal sepsis or meningitis
Neonatal intrapartum
Neonatal preterm
Neonatal LRI
Target
0.00
0.04
0.08
0.12
Mortality rate per 1000 live births
Fraction of mortality
2000
2015
2019
Neonatal other
Neonatal diarrhoea
Neonatal tetanus
Neonatal congenital
Neonatal sepsis or meningitis
Neonatal intrapartum
Neonatal preterm
Neonatal LRI
2000 − 2015
2015 − 2019 (not on target)
Deficit to target
2015 − 2019 (on target)

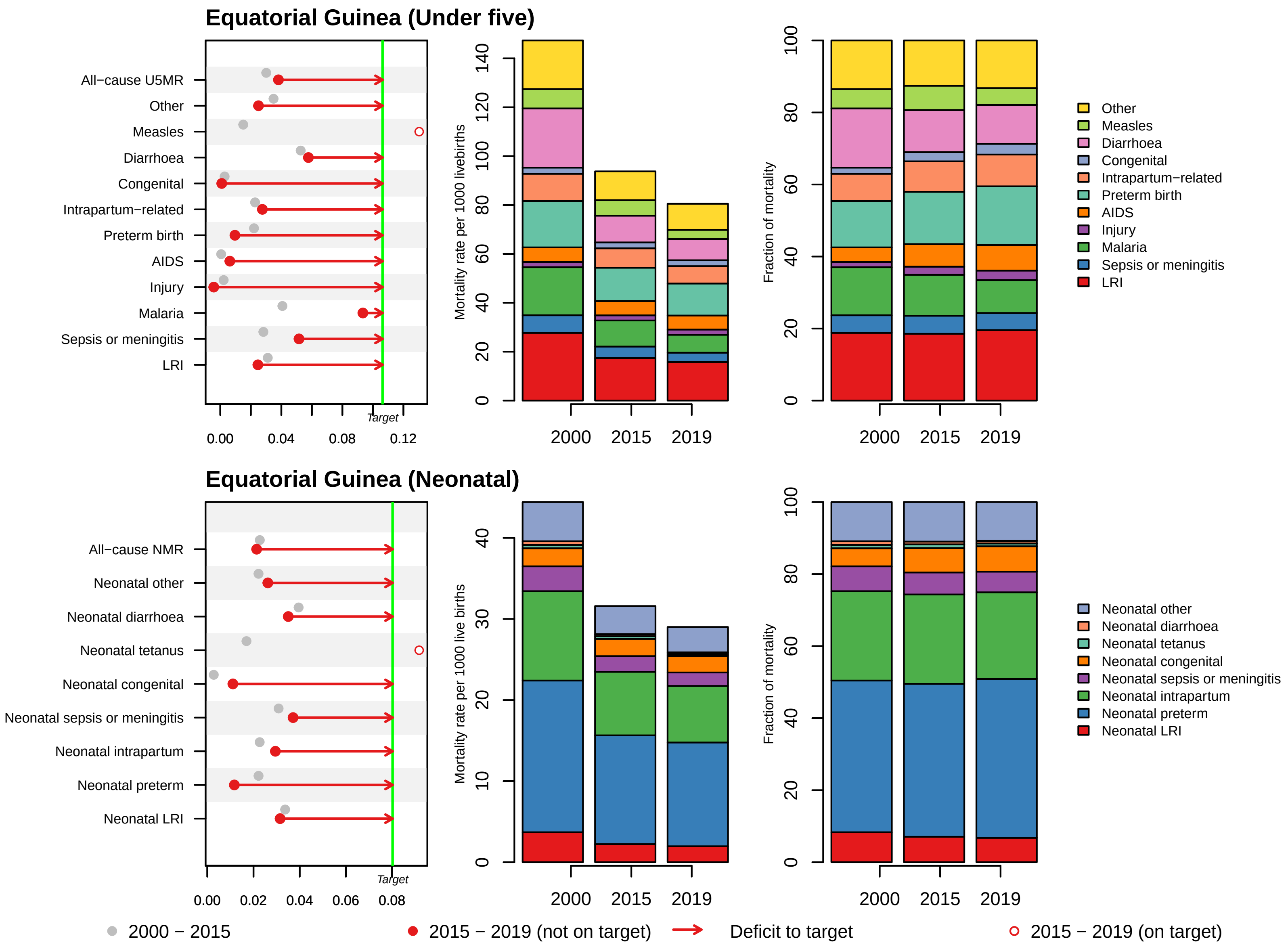

Equatorial Guinea (Under five)
All−cause U5MR
Other
Measles
Diarrhoea
Congenital
Intrapartum−related
Preterm birth
AIDS
Injury
Malaria
Sepsis or meningitis
LRI
Target
0.00
0.04
0.08
0.12
Mortality rate per 1000 livebirths
Fraction of mortality
2000
2015
2019
Other
Measles
Diarrhoea
Congenital
Intrapartum−related
Preterm birth
AIDS
Injury
Malaria
Sepsis or meningitis
LRI
Equatorial Guinea (Neonatal)
All−cause NMR
Neonatal other
Neonatal diarrhoea
Neonatal tetanus
Neonatal congenital
Neonatal sepsis or meningitis
Neonatal intrapartum
Neonatal preterm
Neonatal LRI
0.00
0.02
0.04
0.06
0.08
Mortality rate per 1000 live births
Neonatal other
Neonatal diarrhoea
Neonatal tetanus
Neonatal congenital
Neonatal sepsis or meningitis
Neonatal intrapartum
Neonatal preterm
Neonatal LRI
2000 − 2015
2015 − 2019 (not on target)
Deficit to target
2015 − 2019 (on target)

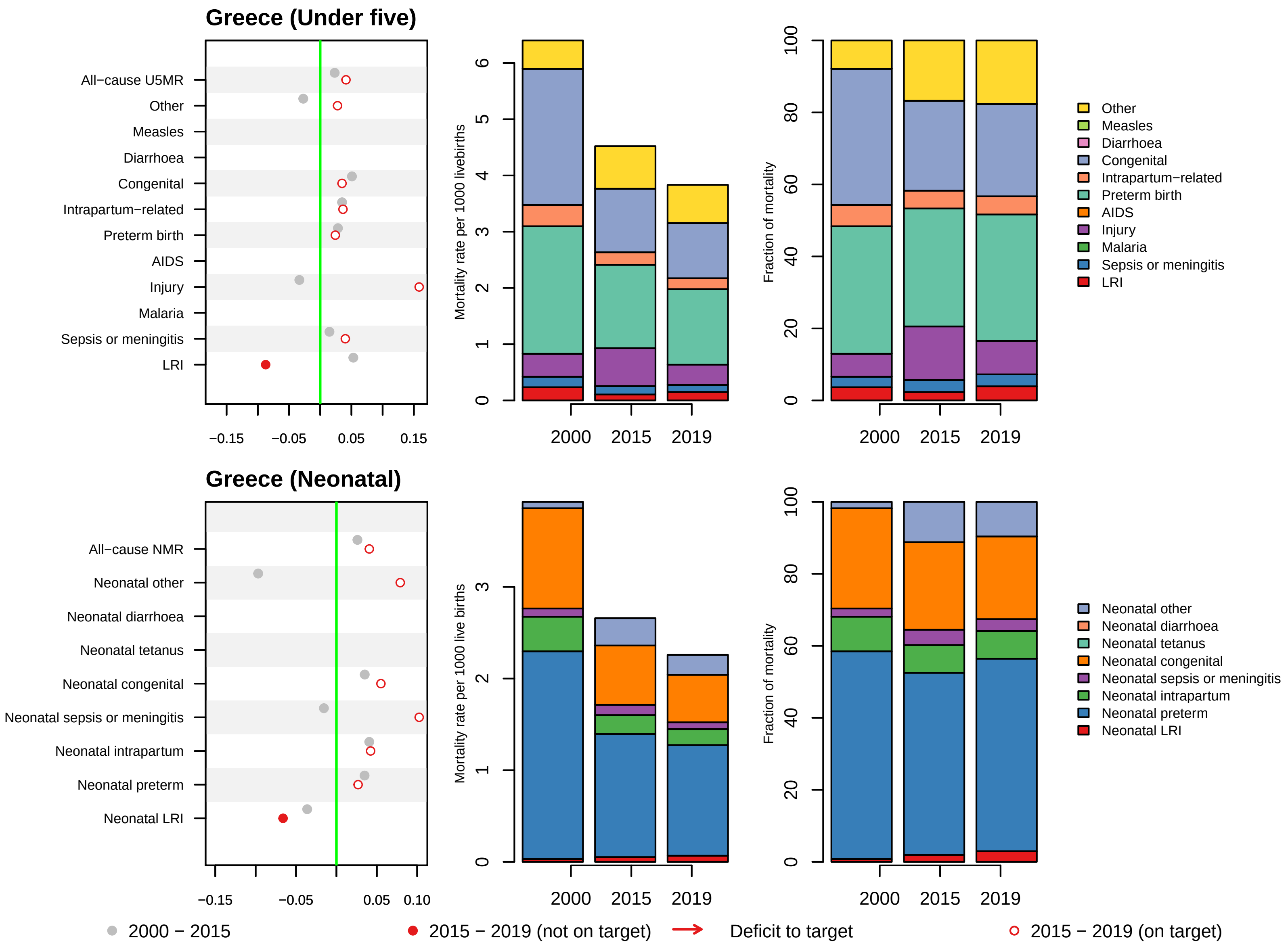

Greece (Under five)
All−cause U5MR
Other
Measles
Diarrhoea
Congenital
Intrapartum−related
Preterm birth
AIDS
Injury
Malaria
Sepsis or meningitis
LRI
−0.15
−0.05
0.05
0.15
Mortality rate per 1000 livebirths
0
1
2
3
4
5
6
2000
2015
2019
Fraction of mortality
0
20
40
60
80
100
Other
Measles
Diarrhoea
Congenital
Intrapartum−related
Preterm birth
AIDS
Injury
Malaria
Sepsis or meningitis
LRI
Greece (Neonatal)
All−cause NMR
Neonatal other
Neonatal diarrhoea
Neonatal tetanus
Neonatal congenital
Neonatal sepsis or meningitis
Neonatal intrapartum
Neonatal preterm
Neonatal LRI
−0.15
−0.05
0.05
0.10
Mortality rate per 1000 live births
0
1
2
3
Neonatal other
Neonatal diarrhoea
Neonatal tetanus
Neonatal congenital
Neonatal sepsis or meningitis
Neonatal intrapartum
Neonatal preterm
Neonatal LRI
2000 − 2015
2015 − 2019 (not on target)
Deficit to target
2015 − 2019 (on target)

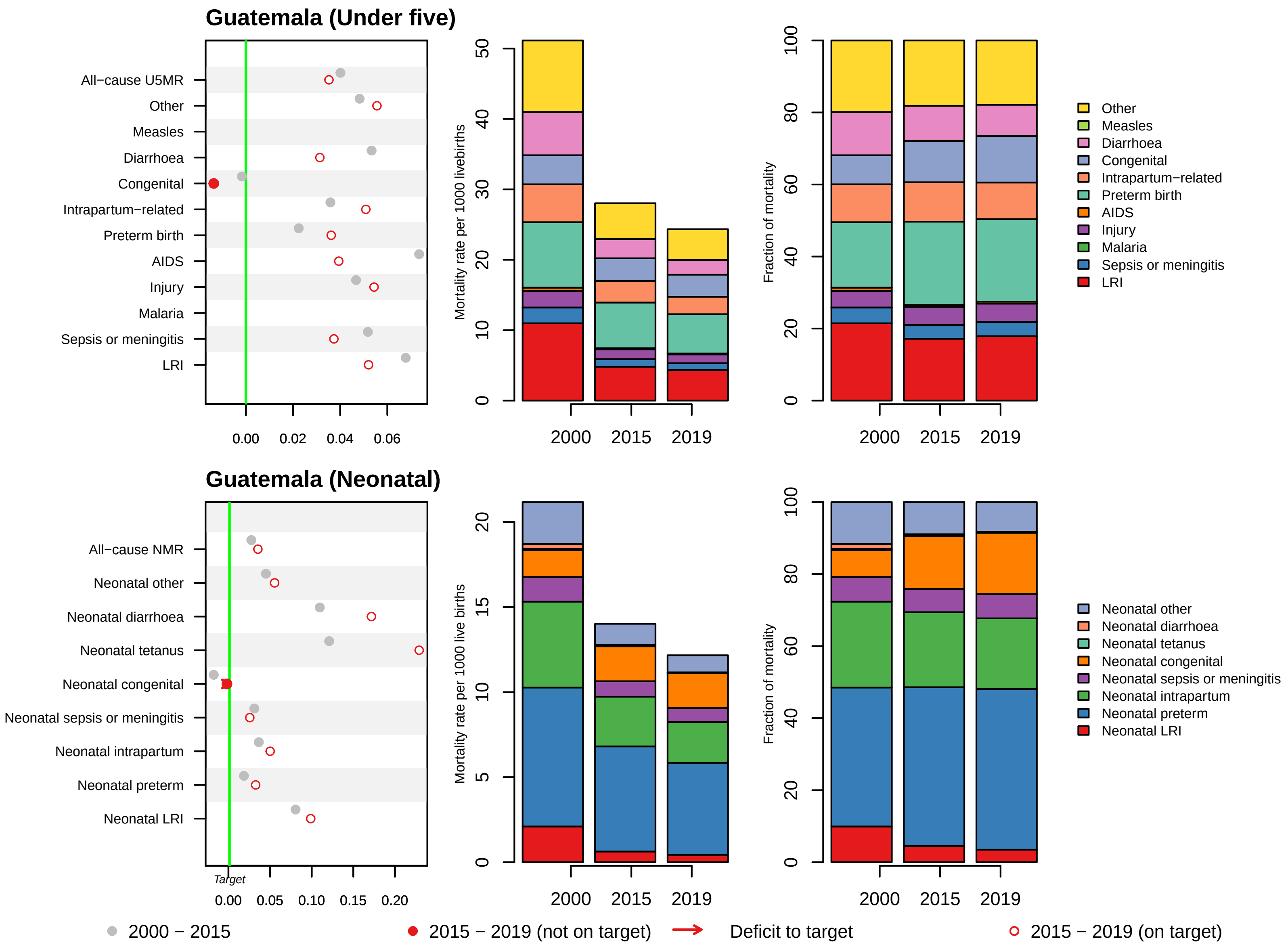

Guatemala (Under five)
All−cause U5MR
Other
Measles
Diarrhoea
Congenital
Intrapartum−related
Preterm birth
AIDS
Injury
Malaria
Sepsis or meningitis
LRI
0.00
0.02
0.04
0.06
Mortality rate per 1000 livebirths
Fraction of mortality
2000
2015
2019
Other
Measles
Diarrhoea
Congenital
Intrapartum−related
Preterm birth
AIDS
Injury
Malaria
Sepsis or meningitis
LRI
Guatemala (Neonatal)
All−cause NMR
Neonatal other
Neonatal diarrhoea
Neonatal tetanus
Neonatal congenital
Neonatal sepsis or meningitis
Neonatal intrapartum
Neonatal preterm
Neonatal LRI
Target
0.00
0.05
0.10
0.15
0.20
Mortality rate per 1000 live births
Fraction of mortality
2000
2015
2019
Neonatal other
Neonatal diarrhoea
Neonatal tetanus
Neonatal congenital
Neonatal sepsis or meningitis
Neonatal intrapartum
Neonatal preterm
Neonatal LRI
2000 − 2015
2015 − 2019 (not on target)
Deficit to target
2015 − 2019 (on target)

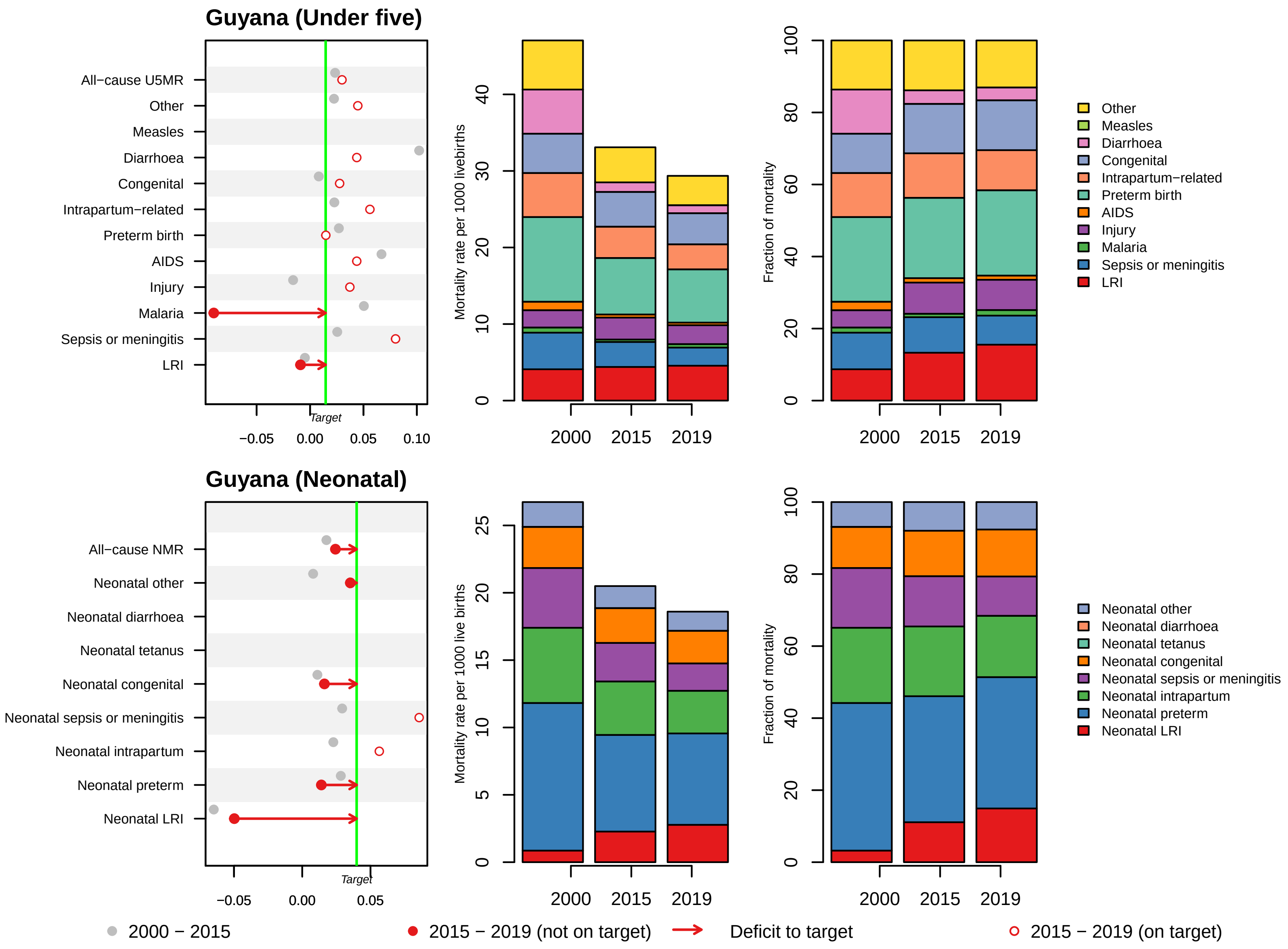
Guyana (Under five)
All−cause U5MR
Other
Measles
Diarrhoea
Congenital
Intrapartum−related
Preterm birth
AIDS
Injury
Malaria
Sepsis or meningitis
LRI
Target
−0.05
0.00
0.05
0.10
Mortality rate per 1000 livebirths
0
10
20
30
40
2000
2015
2019
Fraction of mortality
0
20
40
60
80
100
2000
2015
2019
Other
Measles
Diarrhoea
Congenital
Intrapartum−related
Preterm birth
AIDS
Injury
Malaria
Sepsis or meningitis
LRI
Guyana (Neonatal)
All−cause NMR
Neonatal other
Neonatal diarrhoea
Neonatal tetanus
Neonatal congenital
Neonatal sepsis or meningitis
Neonatal intrapartum
Neonatal preterm
Neonatal LRI
Target
−0.05
0.00
0.05
Mortality rate per 1000 live births
0
5
10
15
20
25
2000
2015
2019
Fraction of mortality
0
20
40
60
80
100
2000
2015
2019
Neonatal other
Neonatal diarrhoea
Neonatal tetanus
Neonatal congenital
Neonatal sepsis or meningitis
Neonatal intrapartum
Neonatal preterm
Neonatal LRI
2000 − 2015
2015 − 2019 (not on target)
Deficit to target
2015 − 2019 (on target)

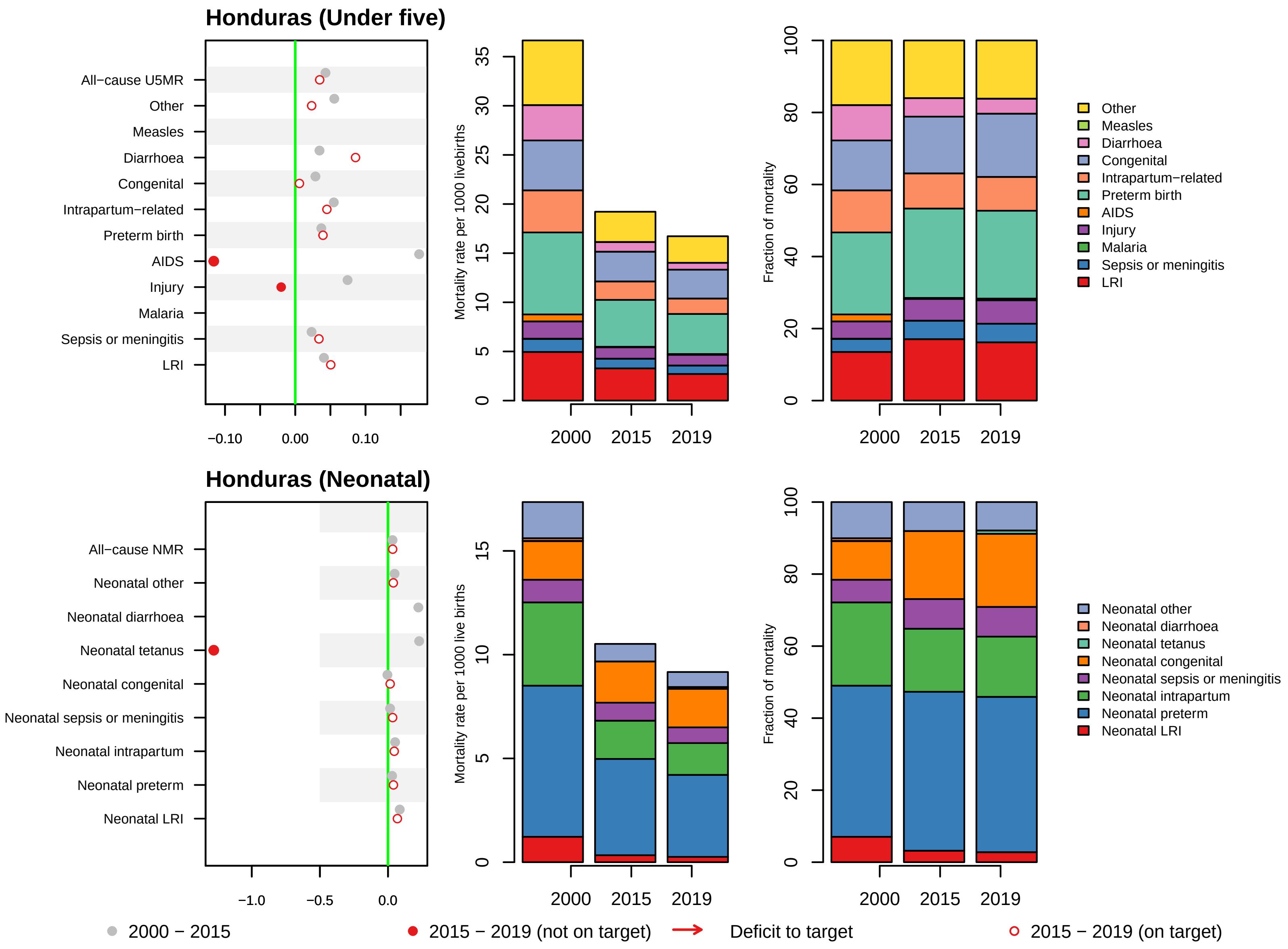

Honduras (Under five)
All−cause U5MR
Other
Measles
Diarrhoea
Congenital
Intrapartum−related
Preterm birth
AIDS
Injury
Malaria
Sepsis or meningitis
LRI
−0.10
0.00
0.10
Mortality rate per 1000 livebirths
2000
2015
2019
Fraction of mortality
Other
Measles
Diarrhoea
Congenital
Intrapartum−related
Preterm birth
AIDS
Injury
Malaria
Sepsis or meningitis
LRI
Honduras (Neonatal)
All−cause NMR
Neonatal other
Neonatal diarrhoea
Neonatal tetanus
Neonatal congenital
Neonatal sepsis or meningitis
Neonatal intrapartum
Neonatal preterm
Neonatal LRI
−1.0
−0.5
0.0
Mortality rate per 1000 live births
Fraction of mortality
Neonatal other
Neonatal diarrhoea
Neonatal tetanus
Neonatal congenital
Neonatal sepsis or meningitis
Neonatal intrapartum
Neonatal preterm
Neonatal LRI
2000 − 2015
2015 − 2019 (not on target)
Deficit to target
2015 − 2019 (on target)

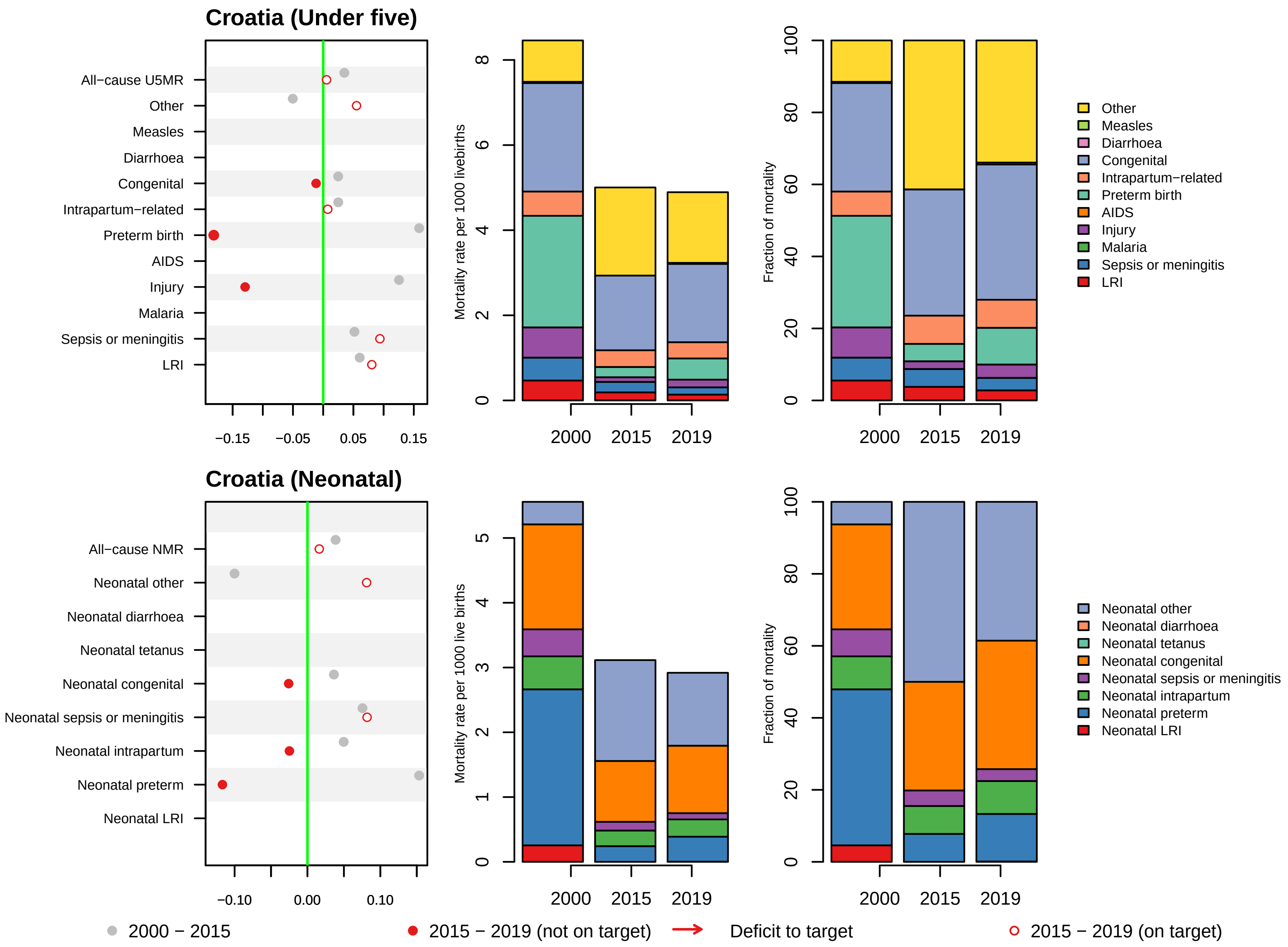
Croatia (Under five)
All−cause U5MR
Other
Measles
Diarrhoea
Congenital
Intrapartum−related
Preterm birth
AIDS
Injury
Malaria
Sepsis or meningitis
LRI
−0.15
−0.05
0.05
0.15
Mortality rate per 1000 livebirths
Fraction of mortality
2000
2015
2019
Other
Measles
Diarrhoea
Congenital
Intrapartum−related
Preterm birth
AIDS
Injury
Malaria
Sepsis or meningitis
LRI
Croatia (Neonatal)
All−cause NMR
Neonatal other
Neonatal diarrhoea
Neonatal tetanus
Neonatal congenital
Neonatal sepsis or meningitis
Neonatal intrapartum
Neonatal preterm
Neonatal LRI
−0.10
0.00
0.10
Mortality rate per 1000 live births
Fraction of mortality
2000
2015
2019
Neonatal other
Neonatal diarrhoea
Neonatal tetanus
Neonatal congenital
Neonatal sepsis or meningitis
Neonatal intrapartum
Neonatal preterm
Neonatal LRI
2000 − 2015
2015 − 2019 (not on target)
Deficit to target
2015 − 2019 (on target)

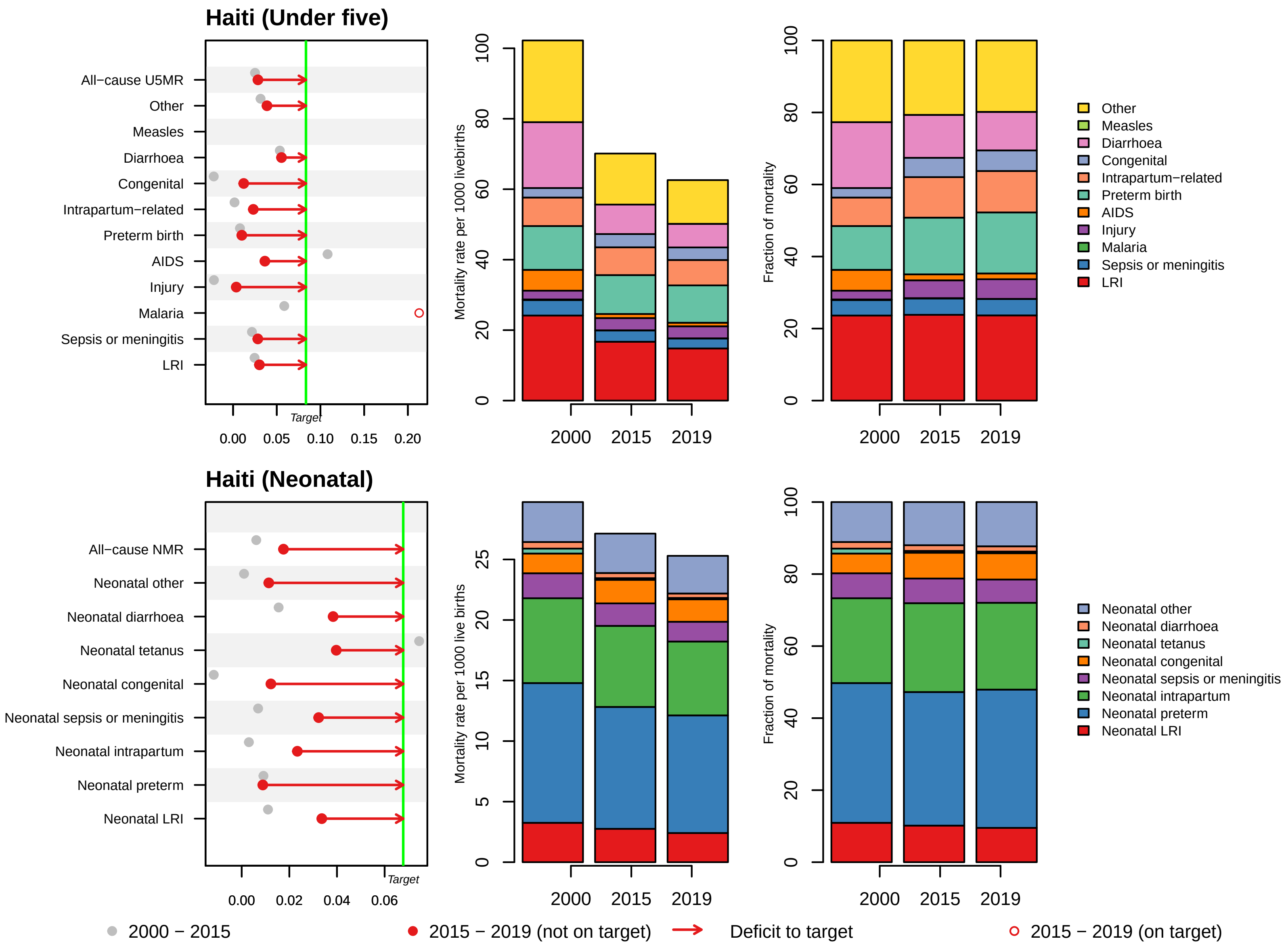
Haiti (Under five)
All−cause U5MR
Other
Measles
Diarrhoea
Congenital
Intrapartum−related
Preterm birth
AIDS
Injury
Malaria
Sepsis or meningitis
LRI
Target
0.00 0.05 0.10 0.15 0.20
Mortality rate per 1000 livebirths
Fraction of mortality
2000 2015 2019
Other
Measles
Diarrhoea
Congenital
Intrapartum−related
Preterm birth
AIDS
Injury
Malaria
Sepsis or meningitis
LRI
Haiti (Neonatal)
All−cause NMR
Neonatal other
Neonatal diarrhoea
Neonatal tetanus
Neonatal congenital
Neonatal sepsis or meningitis
Neonatal intrapartum
Neonatal preterm
Neonatal LRI
0.00 0.02 0.04 0.06
Mortality rate per 1000 live births
Neonatal other
Neonatal diarrhoea
Neonatal tetanus
Neonatal congenital
Neonatal sepsis or meningitis
Neonatal intrapartum
Neonatal preterm
Neonatal LRI
2000 − 2015
2015 − 2019 (not on target)
Deficit to target
2015 − 2019 (on target)

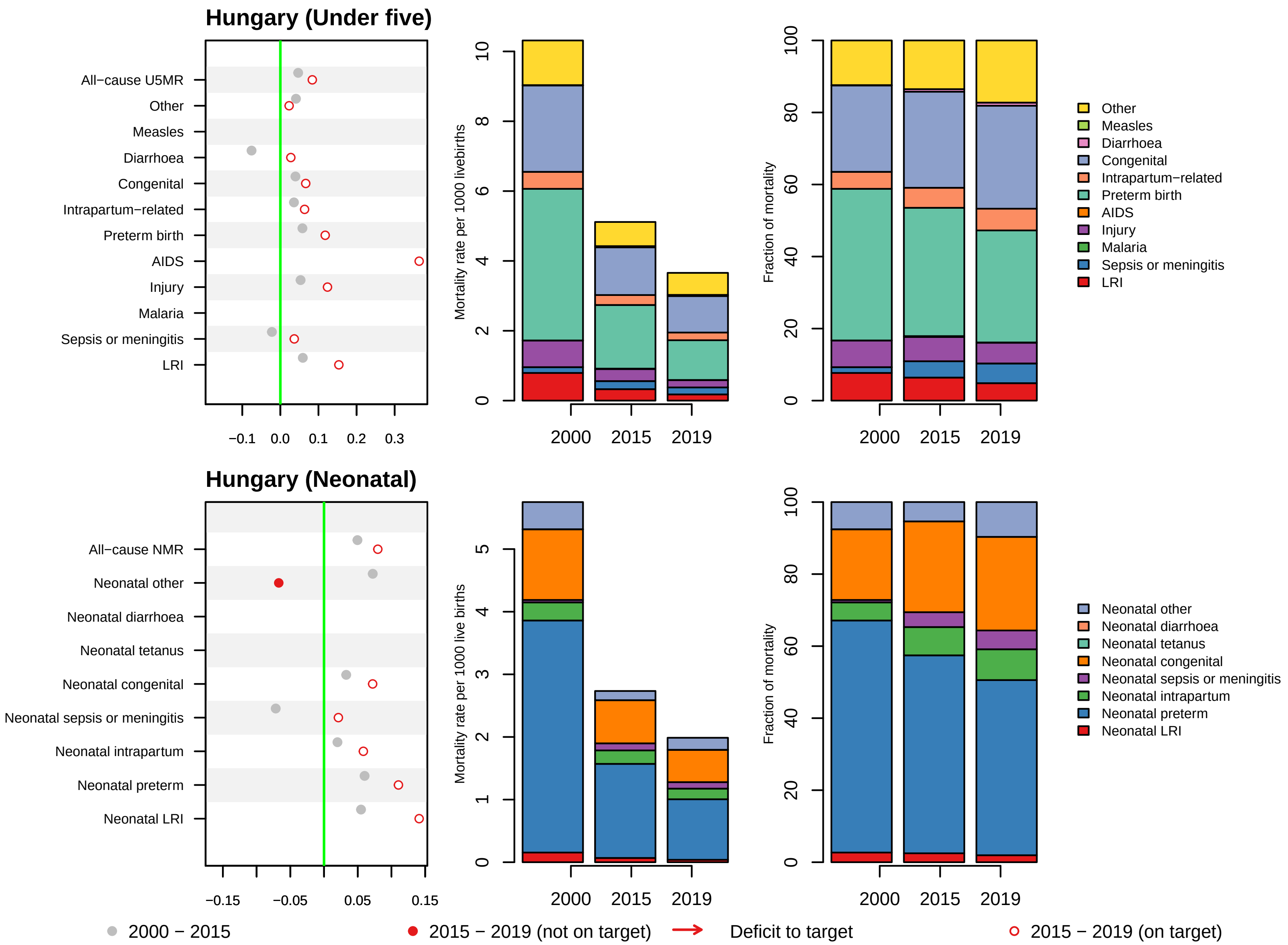
Hungary (Under five)
All−cause U5MR
Other
Measles
Diarrhoea
Congenital
Intrapartum−related
Preterm birth
AIDS
Injury
Malaria
Sepsis or meningitis
LRI
−0.1
0.0
0.1
0.2
0.3
Mortality rate per 1000 livebirths
2000
2015
2019
Fraction of mortality
Other
Measles
Diarrhoea
Congenital
Intrapartum−related
Preterm birth
AIDS
Injury
Malaria
Sepsis or meningitis
LRI
Hungary (Neonatal)
All−cause NMR
Neonatal other
Neonatal diarrhoea
Neonatal tetanus
Neonatal congenital
Neonatal sepsis or meningitis
Neonatal intrapartum
Neonatal preterm
Neonatal LRI
−0.15
−0.05
0.05
0.15
Mortality rate per 1000 live births
2000
2015
2019
Fraction of mortality
Neonatal other
Neonatal diarrhoea
Neonatal tetanus
Neonatal congenital
Neonatal sepsis or meningitis
Neonatal intrapartum
Neonatal preterm
Neonatal LRI
2000 − 2015
2015 − 2019 (not on target)
Deficit to target
2015 − 2019 (on target)

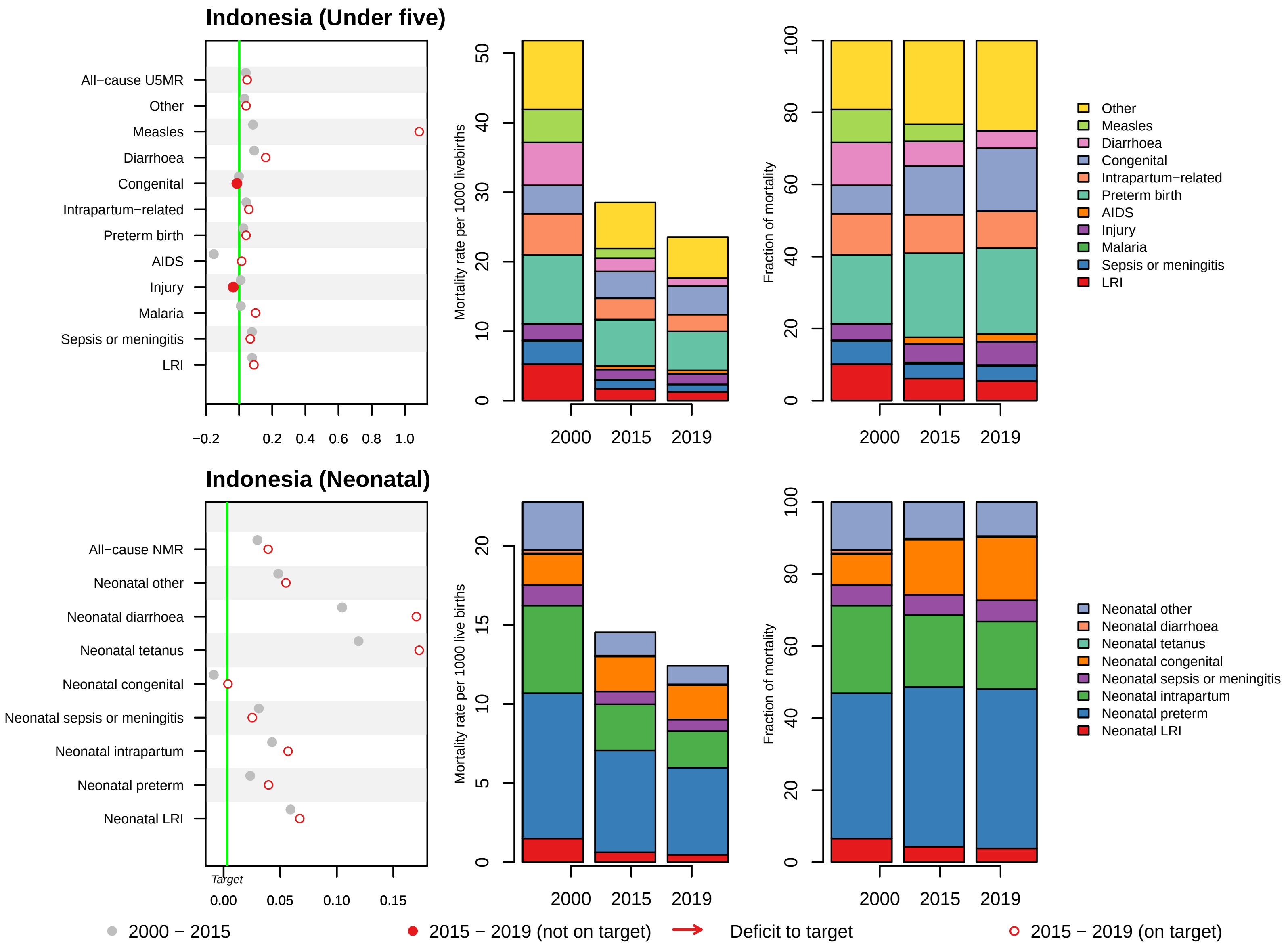

Indonesia (Under five)
All−cause U5MR
Other
Measles
Diarrhoea
Congenital
Intrapartum−related
Preterm birth
AIDS
Injury
Malaria
Sepsis or meningitis
LRI
−0.2
0.2
0.4
0.6
0.8
1.0
Mortality rate per 1000 livebirths
Fraction of mortality
2000
2015
2019
Other
Measles
Diarrhoea
Congenital
Intrapartum−related
Preterm birth
AIDS
Injury
Malaria
Sepsis or meningitis
LRI
Indonesia (Neonatal)
All−cause NMR
Neonatal other
Neonatal diarrhoea
Neonatal tetanus
Neonatal congenital
Neonatal sepsis or meningitis
Neonatal intrapartum
Neonatal preterm
Neonatal LRI
Target
0.00
0.05
0.10
0.15
Mortality rate per 1000 live births
Fraction of mortality
Neonatal other
Neonatal diarrhoea
Neonatal tetanus
Neonatal congenital
Neonatal sepsis or meningitis
Neonatal intrapartum
Neonatal preterm
Neonatal LRI
2000 − 2015
2015 − 2019 (not on target)
Deficit to target
2015 − 2019 (on target)

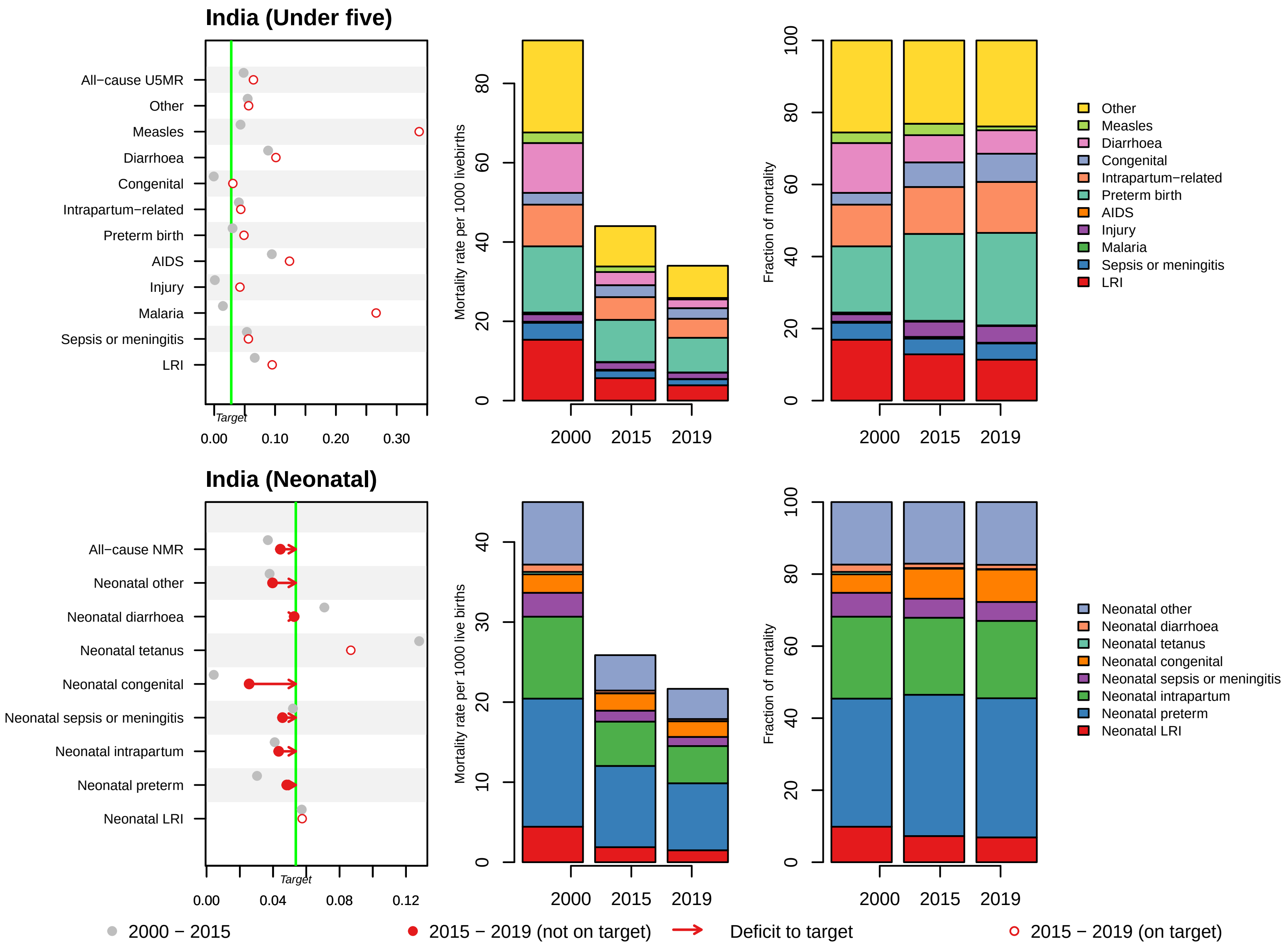
India (Under five)
All−cause U5MR
Other
Measles
Diarrhoea
Congenital
Intrapartum−related
Preterm birth
AIDS
Injury
Malaria
Sepsis or meningitis
LRI
Target
0.00
0.10
0.20
0.30
Mortality rate per 1000 livebirths
0
20
40
60
80
2000
2015
2019
Fraction of mortality
0
20
40
60
80
100
2000
2015
2019
Other
Measles
Diarrhoea
Congenital
Intrapartum−related
Preterm birth
AIDS
Injury
Malaria
Sepsis or meningitis
LRI
India (Neonatal)
All−cause NMR
Neonatal other
Neonatal diarrhoea
Neonatal tetanus
Neonatal congenital
Neonatal sepsis or meningitis
Neonatal intrapartum
Neonatal preterm
Neonatal LRI
Target
0.00
0.04
0.08
0.12
Mortality rate per 1000 live births
0
10
20
30
40
2000
2015
2019
Fraction of mortality
0
20
40
60
80
100
2000
2015
2019
Neonatal other
Neonatal diarrhoea
Neonatal tetanus
Neonatal congenital
Neonatal sepsis or meningitis
Neonatal intrapartum
Neonatal preterm
Neonatal LRI
2000 − 2015
2015 − 2019 (not on target)
Deficit to target
2015 − 2019 (on target)

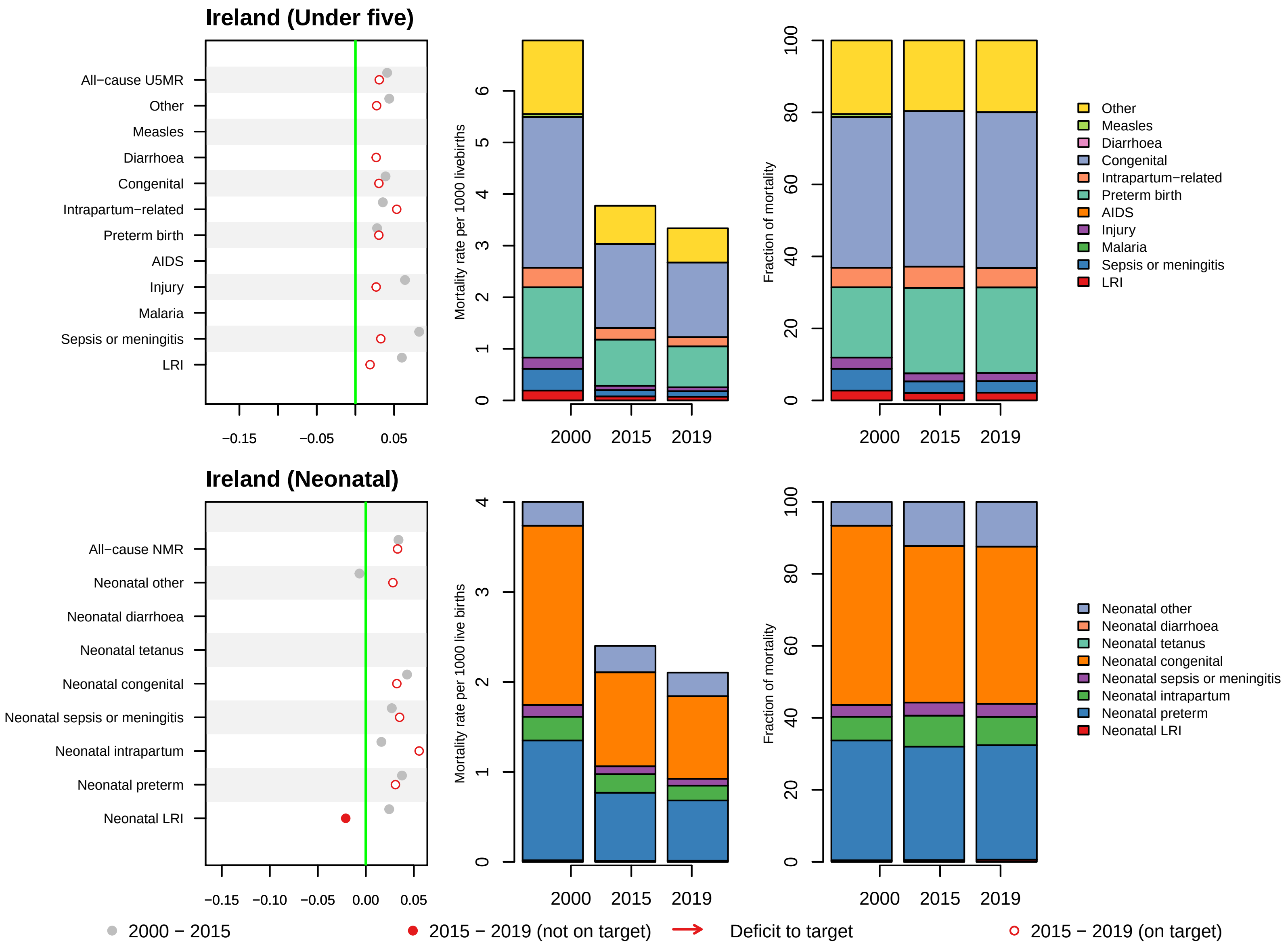

Ireland (Under five)
All−cause U5MR
Other
Measles
Diarrhoea
Congenital
Intrapartum−related
Preterm birth
AIDS
Injury
Malaria
Sepsis or meningitis
LRI
−0.15
−0.05
0.05
Mortality rate per 1000 livebirths
0
1
2
3
4
5
6
2000
2015
2019
Fraction of mortality
0
20
40
60
80
100
Other
Measles
Diarrhoea
Congenital
Intrapartum−related
Preterm birth
AIDS
Injury
Malaria
Sepsis or meningitis
LRI
Ireland (Neonatal)
All−cause NMR
Neonatal other
Neonatal diarrhoea
Neonatal tetanus
Neonatal congenital
Neonatal sepsis or meningitis
Neonatal intrapartum
Neonatal preterm
Neonatal LRI
−0.15
−0.10
−0.05
0.00
0.05
Mortality rate per 1000 live births
0
1
2
3
4
Neonatal other
Neonatal diarrhoea
Neonatal tetanus
Neonatal congenital
Neonatal sepsis or meningitis
Neonatal intrapartum
Neonatal preterm
Neonatal LRI
2000 − 2015
2015 − 2019 (not on target)
Deficit to target
2015 − 2019 (on target)

Iran (Islamic Republic of) (Under five)

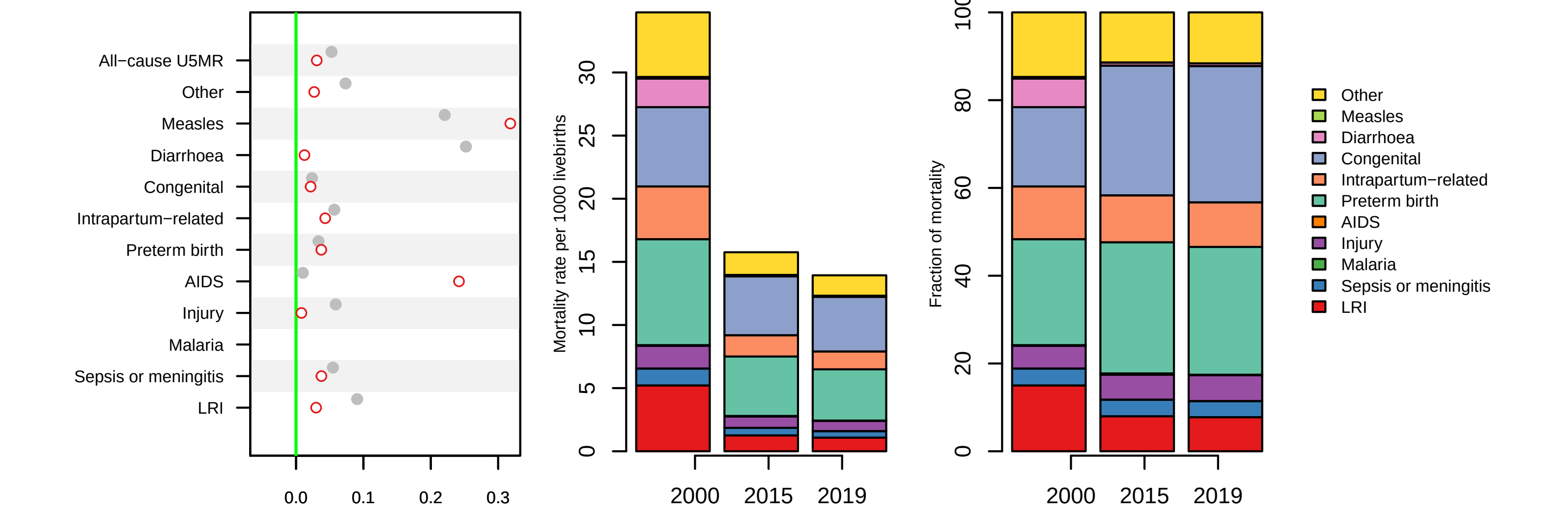

Iran (Islamic Republic of) (Neonatal)

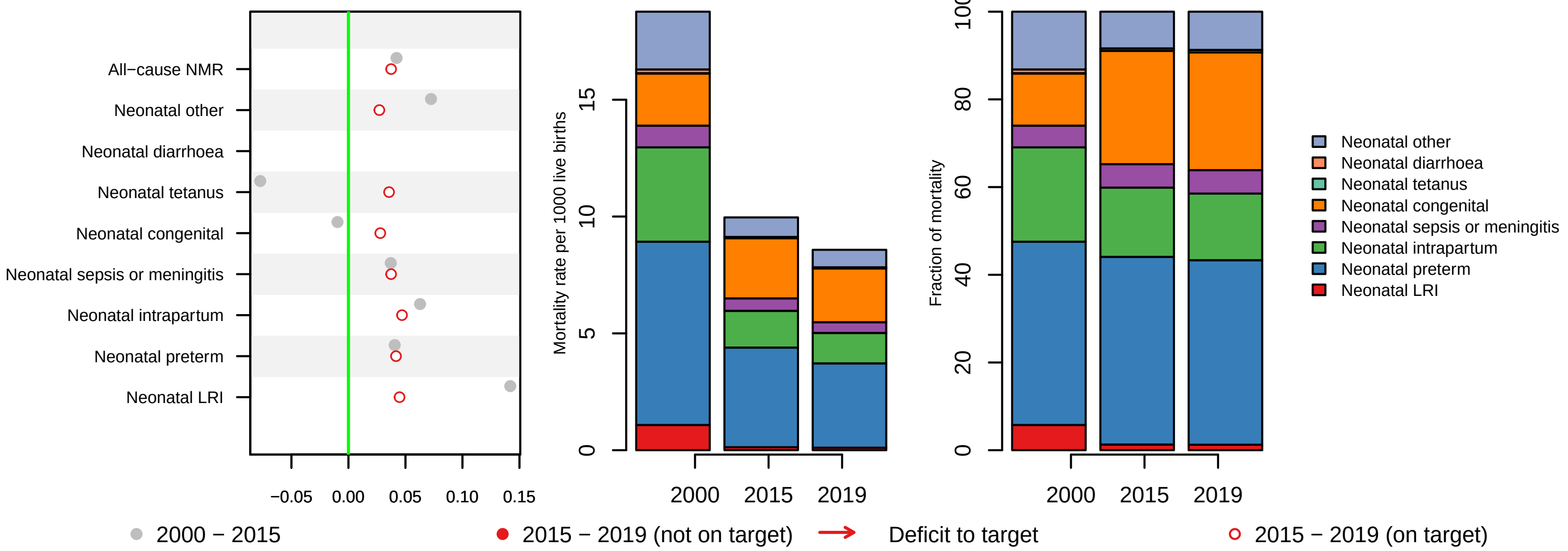

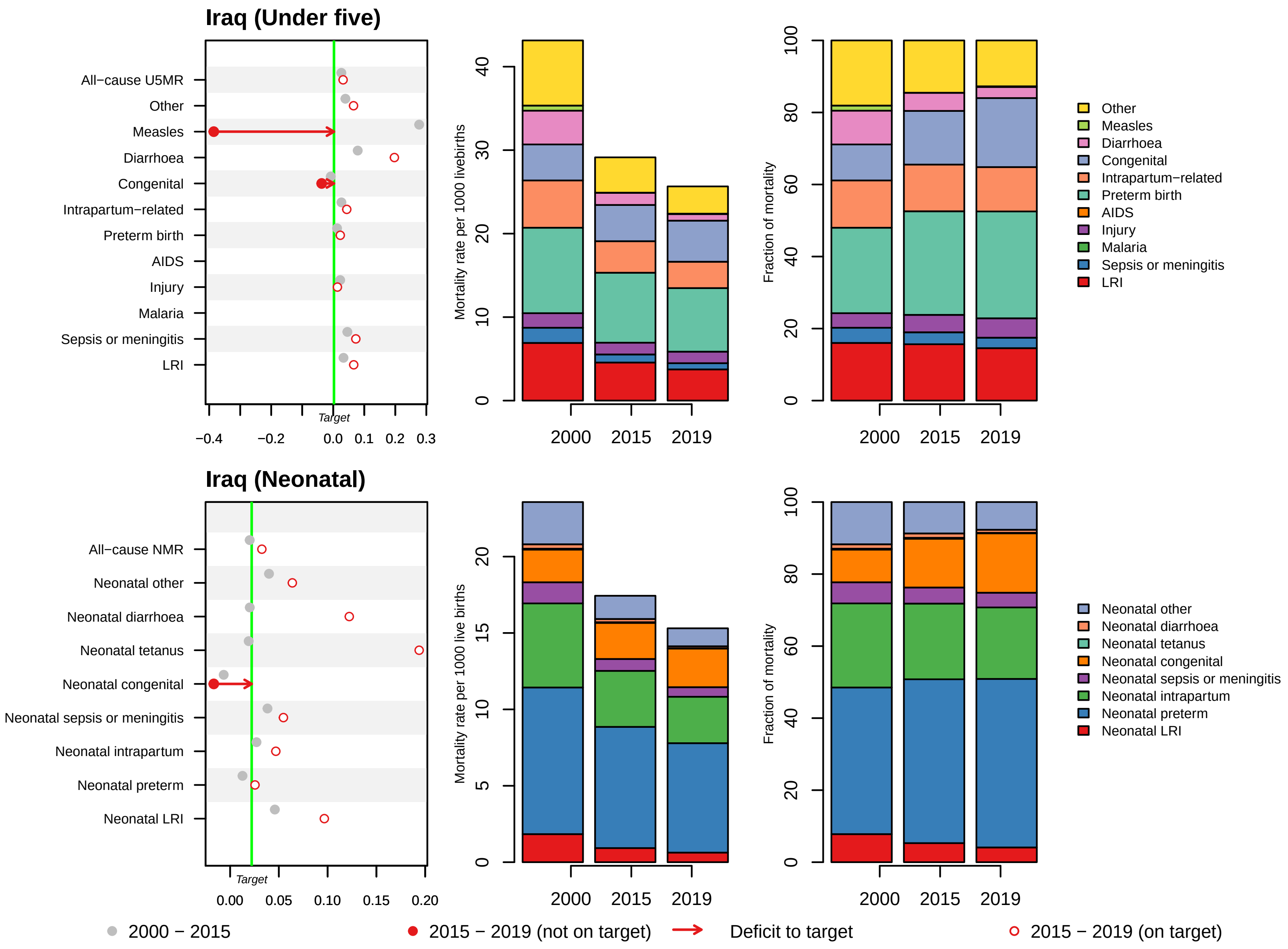

Iraq (Under five)
All−cause U5MR
Other
Measles
Diarrhoea
Congenital
Intrapartum−related
Preterm birth
AIDS
Injury
Malaria
Sepsis or meningitis
LRI
Target
−0.4
−0.2
0.0
0.1
0.2
0.3
Mortality rate per 1000 livebirths
0
10
20
30
40
2000
2015
2019
Fraction of mortality
0
20
40
60
80
100
Other
Measles
Diarrhoea
Congenital
Intrapartum−related
Preterm birth
AIDS
Injury
Malaria
Sepsis or meningitis
LRI
Iraq (Neonatal)
All−cause NMR
Neonatal other
Neonatal diarrhoea
Neonatal tetanus
Neonatal congenital
Neonatal sepsis or meningitis
Neonatal intrapartum
Neonatal preterm
Neonatal LRI
Target
0.00
0.05
0.10
0.15
0.20
Mortality rate per 1000 live births
0
5
10
15
20
2000
2015
2019
Fraction of mortality
0
20
40
60
80
100
Neonatal other
Neonatal diarrhoea
Neonatal tetanus
Neonatal congenital
Neonatal sepsis or meningitis
Neonatal intrapartum
Neonatal preterm
Neonatal LRI
2000 − 2015
2015 − 2019 (not on target)
Deficit to target
2015 − 2019 (on target)

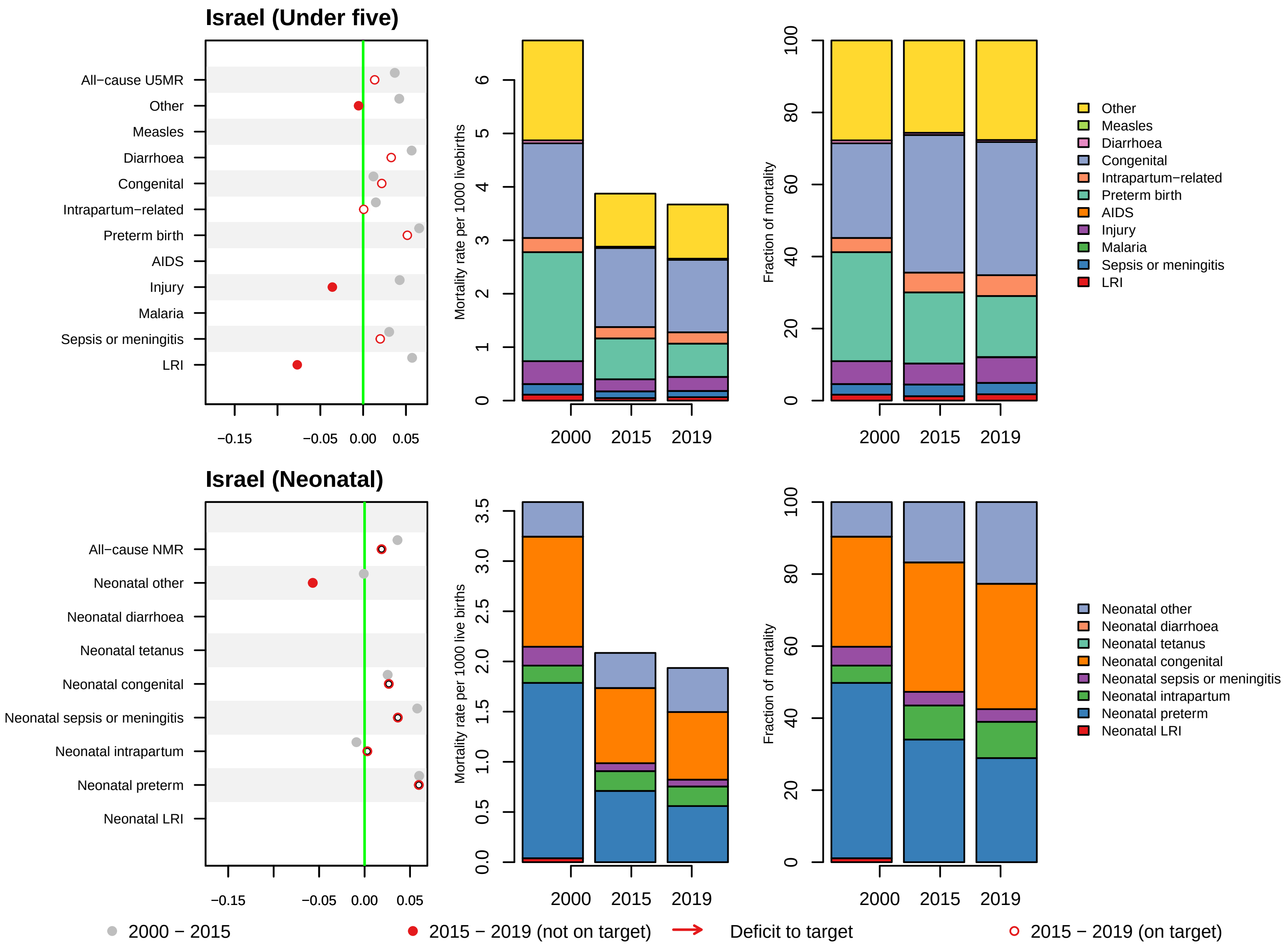

Israel (Under five)
All−cause U5MR
Other
Measles
Diarrhoea
Congenital
Intrapartum−related
Preterm birth
AIDS
Injury
Malaria
Sepsis or meningitis
LRI
−0.15
−0.05
0.00
0.05
Mortality rate per 1000 livebirths
2000
2015
2019
Fraction of mortality
Other
Measles
Diarrhoea
Congenital
Intrapartum−related
Preterm birth
AIDS
Injury
Malaria
Sepsis or meningitis
LRI
Israel (Neonatal)
All−cause NMR
Neonatal other
Neonatal diarrhoea
Neonatal tetanus
Neonatal congenital
Neonatal sepsis or meningitis
Neonatal intrapartum
Neonatal preterm
Neonatal LRI
Mortality rate per 1000 live births
Fraction of mortality
Neonatal other
Neonatal diarrhoea
Neonatal tetanus
Neonatal congenital
Neonatal sepsis or meningitis
Neonatal intrapartum
Neonatal preterm
Neonatal LRI
2000 − 2015
2015 − 2019 (not on target)
Deficit to target
2015 − 2019 (on target)

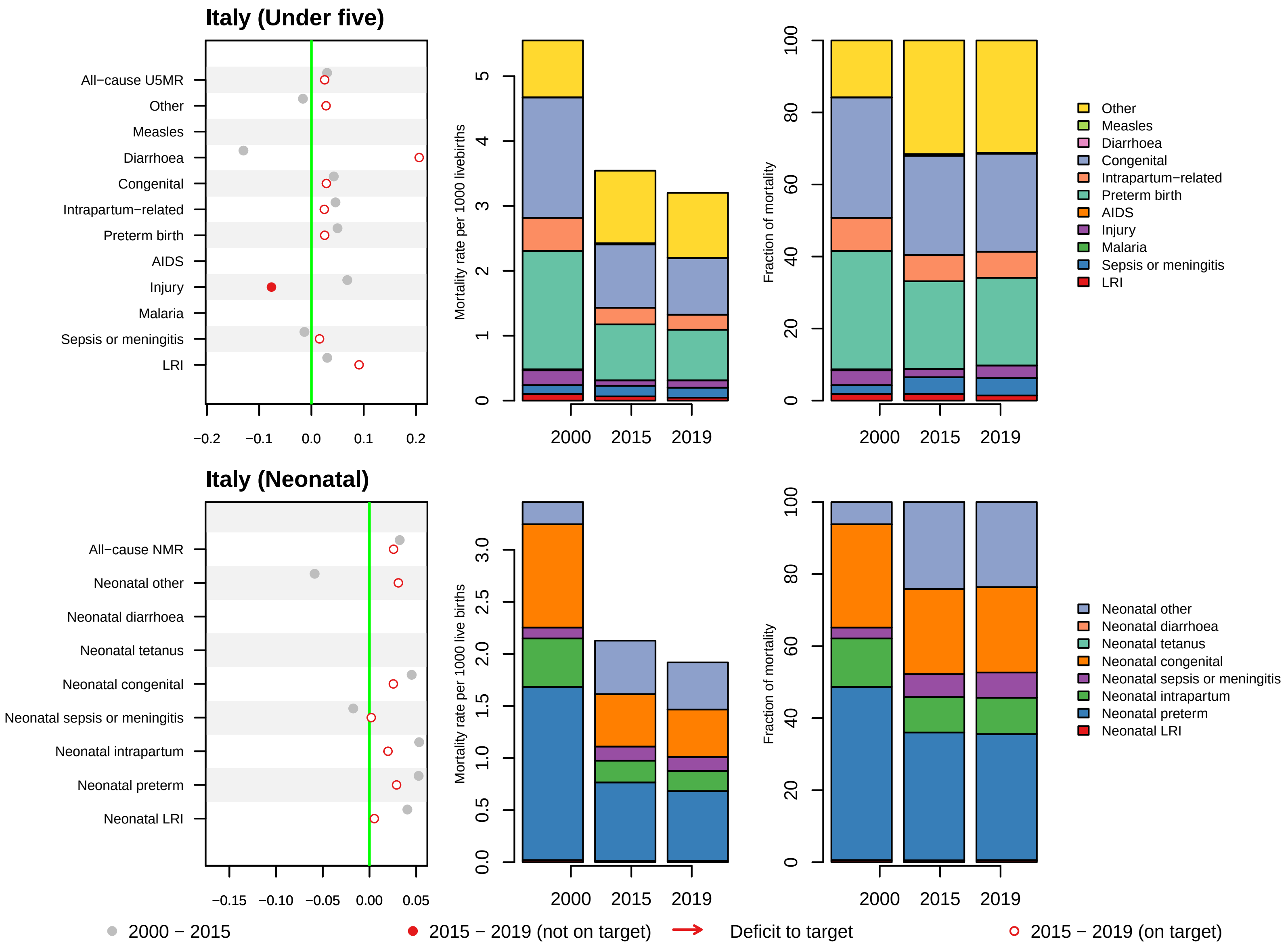

Italy (Under five)
All−cause U5MR
Other
Measles
Diarrhoea
Congenital
Intrapartum−related
Preterm birth
AIDS
Injury
Malaria
Sepsis or meningitis
LRI
−0.2
−0.1
0.0
0.1
0.2
Mortality rate per 1000 livebirths
2000
2015
2019
Fraction of mortality
Other
Measles
Diarrhoea
Congenital
Intrapartum−related
Preterm birth
AIDS
Injury
Malaria
Sepsis or meningitis
LRI
Italy (Neonatal)
All−cause NMR
Neonatal other
Neonatal diarrhoea
Neonatal tetanus
Neonatal congenital
Neonatal sepsis or meningitis
Neonatal intrapartum
Neonatal preterm
Neonatal LRI
−0.15
−0.10
−0.05
0.00
0.05
Mortality rate per 1000 live births
2000
2015
2019
Fraction of mortality
Neonatal other
Neonatal diarrhoea
Neonatal tetanus
Neonatal congenital
Neonatal sepsis or meningitis
Neonatal intrapartum
Neonatal preterm
Neonatal LRI
2000 − 2015
2015 − 2019 (not on target)
Deficit to target
2015 − 2019 (on target)

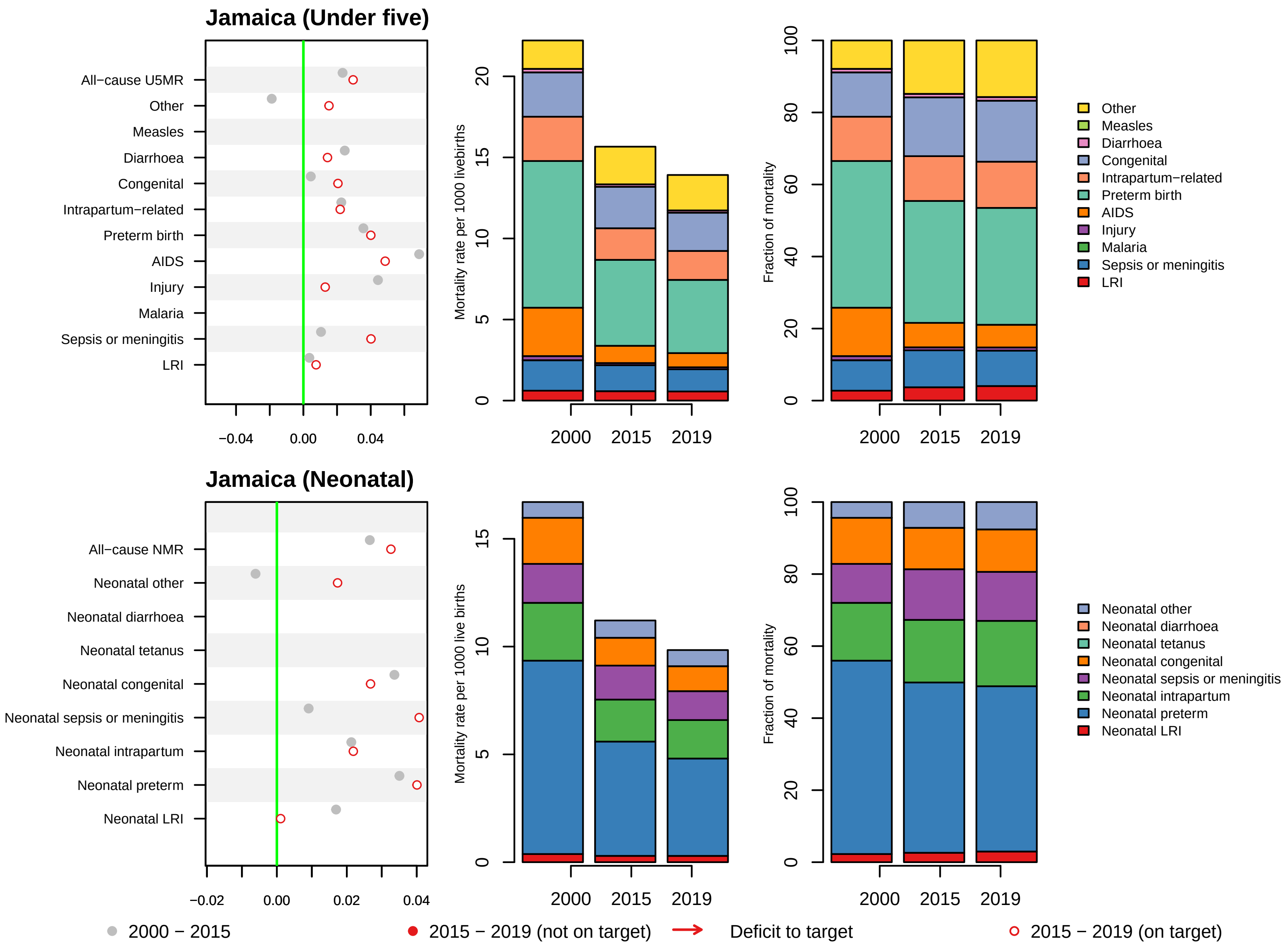

Jamaica (Under five)
All−cause U5MR
Other
Measles
Diarrhoea
Congenital
Intrapartum−related
Preterm birth
AIDS
Injury
Malaria
Sepsis or meningitis
LRI
−0.04
0.00
0.04
Mortality rate per 1000 livebirths
0
5
10
15
20
2000
2015
2019
Fraction of mortality
0
20
40
60
80
100
Other
Measles
Diarrhoea
Congenital
Intrapartum−related
Preterm birth
AIDS
Injury
Malaria
Sepsis or meningitis
LRI
Jamaica (Neonatal)
All−cause NMR
Neonatal other
Neonatal diarrhoea
Neonatal tetanus
Neonatal congenital
Neonatal sepsis or meningitis
Neonatal intrapartum
Neonatal preterm
Neonatal LRI
−0.02
0.00
0.02
0.04
Mortality rate per 1000 live births
Neonatal other
Neonatal diarrhoea
Neonatal tetanus
Neonatal congenital
Neonatal sepsis or meningitis
Neonatal intrapartum
Neonatal preterm
Neonatal LRI
2000 − 2015
2015 − 2019 (not on target)
Deficit to target
2015 − 2019 (on target)

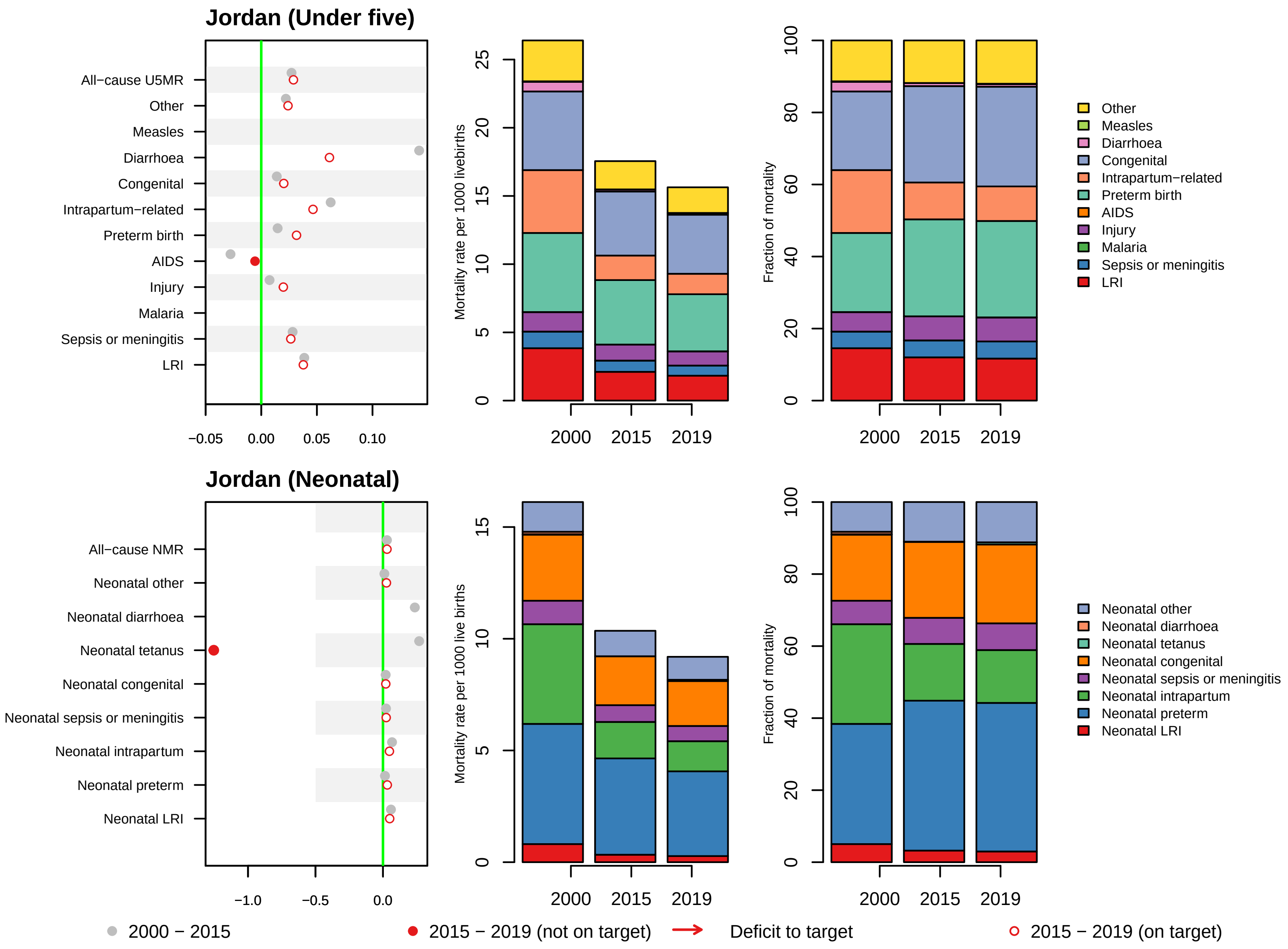

Jordan (Under five)
All−cause U5MR
Other
Measles
Diarrhoea
Congenital
Intrapartum−related
Preterm birth
AIDS
Injury
Malaria
Sepsis or meningitis
LRI
−0.05
0.00
0.05
0.10
Mortality rate per 1000 livebirths
Fraction of mortality
2000
2015
2019
Other
Measles
Diarrhoea
Congenital
Intrapartum−related
Preterm birth
AIDS
Injury
Malaria
Sepsis or meningitis
LRI
Jordan (Neonatal)
All−cause NMR
Neonatal other
Neonatal diarrhoea
Neonatal tetanus
Neonatal congenital
Neonatal sepsis or meningitis
Neonatal intrapartum
Neonatal preterm
Neonatal LRI
−1.0
−0.5
0.0
Mortality rate per 1000 live births
Fraction of mortality
2000
2015
2019
Neonatal other
Neonatal diarrhoea
Neonatal tetanus
Neonatal congenital
Neonatal sepsis or meningitis
Neonatal intrapartum
Neonatal preterm
Neonatal LRI
2000 − 2015
2015 − 2019 (not on target)
Deficit to target
2015 − 2019 (on target)

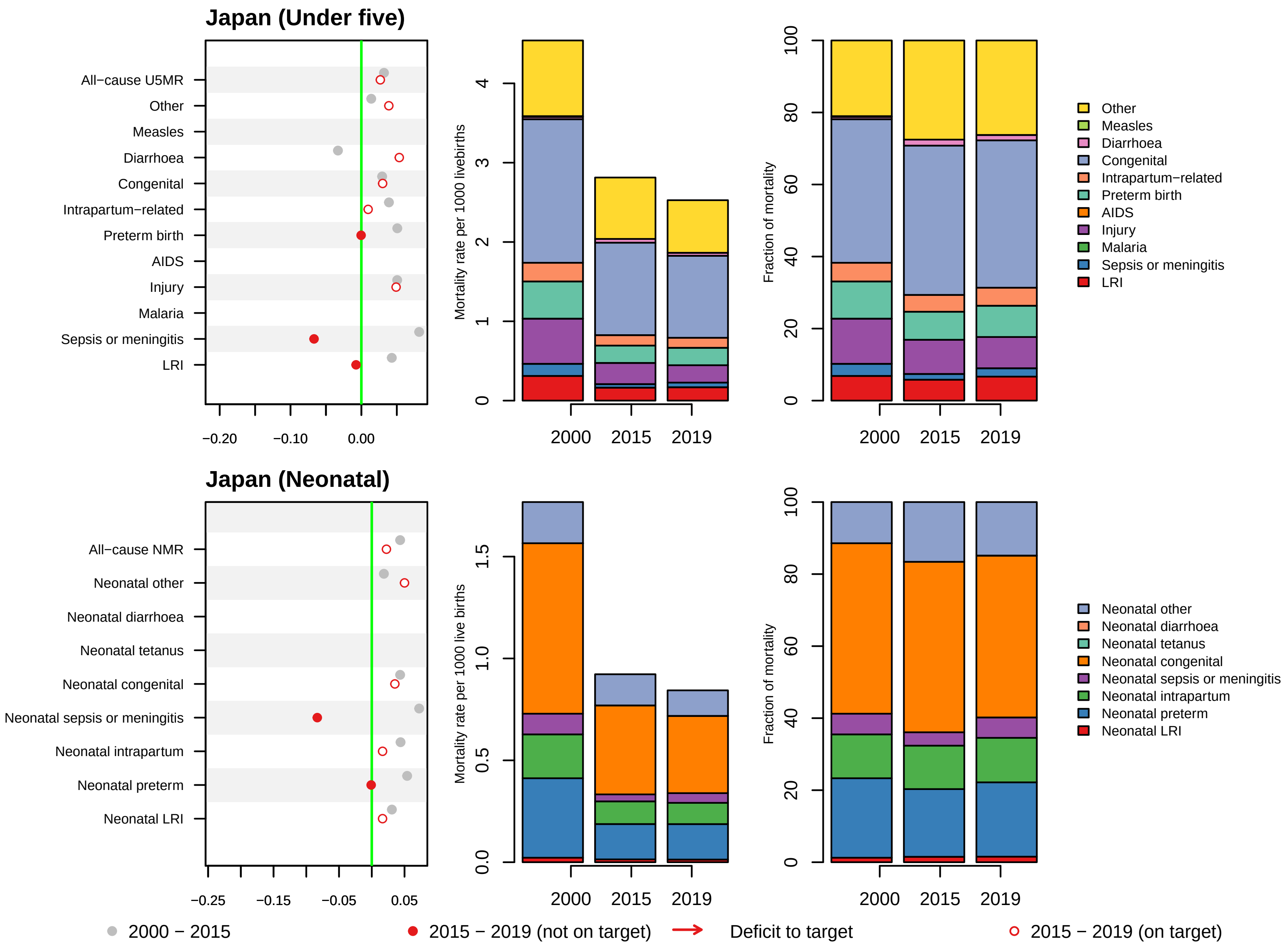

Japan (Under five)
All−cause U5MR
Other
Measles
Diarrhoea
Congenital
Intrapartum−related
Preterm birth
AIDS
Injury
Malaria
Sepsis or meningitis
LRI
−0.20
−0.10
0.00
Mortality rate per 1000 livebirths
Fraction of mortality
2000
2015
2019
Other
Measles
Diarrhoea
Congenital
Intrapartum−related
Preterm birth
AIDS
Injury
Malaria
Sepsis or meningitis
LRI
Japan (Neonatal)
All−cause NMR
Neonatal other
Neonatal diarrhoea
Neonatal tetanus
Neonatal congenital
Neonatal sepsis or meningitis
Neonatal intrapartum
Neonatal preterm
Neonatal LRI
−0.25
−0.15
−0.05
0.05
Mortality rate per 1000 live births
Fraction of mortality
Neonatal other
Neonatal diarrhoea
Neonatal tetanus
Neonatal congenital
Neonatal sepsis or meningitis
Neonatal intrapartum
Neonatal preterm
Neonatal LRI
2000 − 2015
2015 − 2019 (not on target)
Deficit to target
2015 − 2019 (on target)

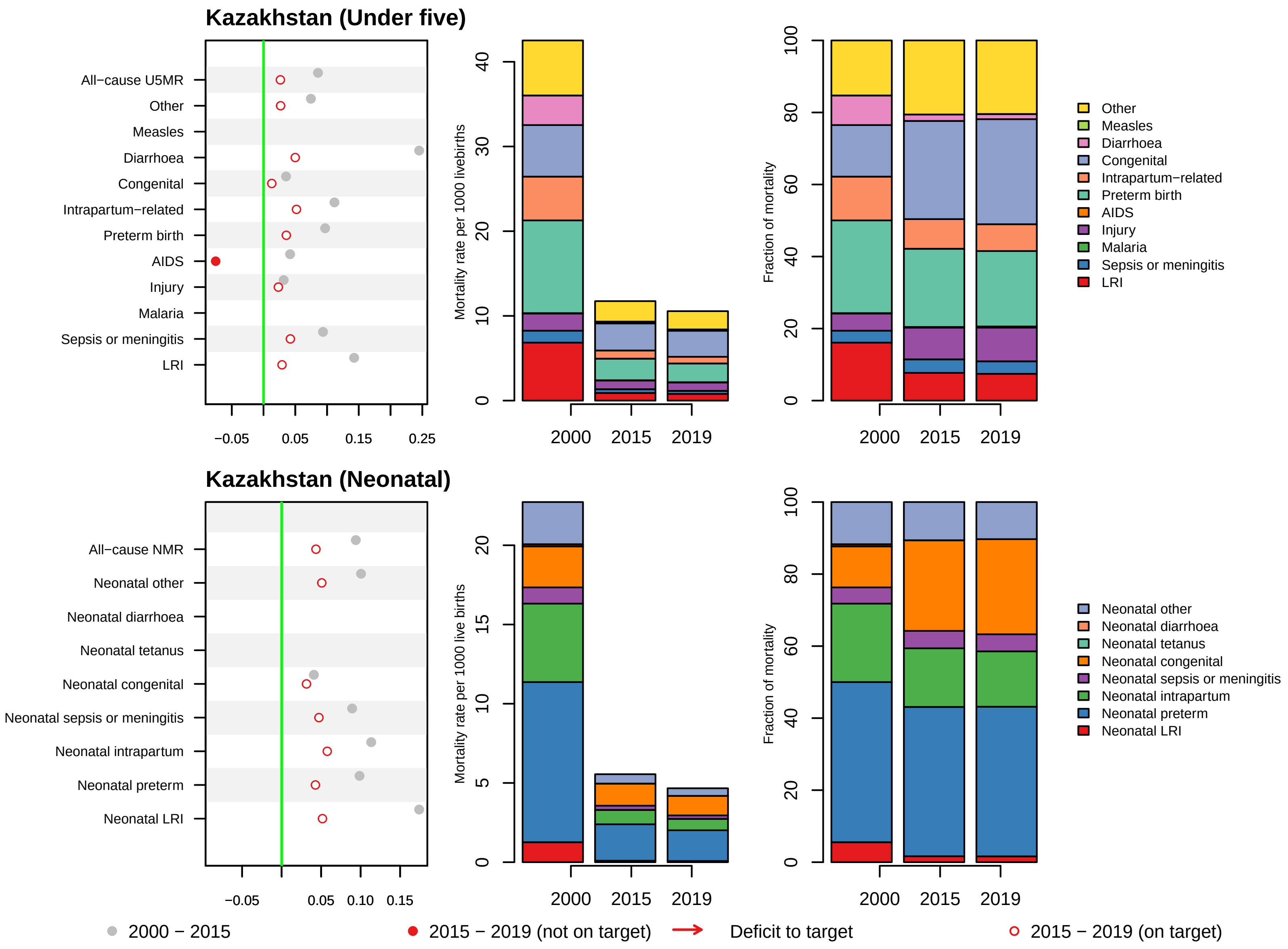

Kazakhstan (Under five)
All−cause U5MR
Other
Measles
Diarrhoea
Congenital
Intrapartum−related
Preterm birth
AIDS
Injury
Malaria
Sepsis or meningitis
LRI
−0.05
0.05
0.15
0.25
Mortality rate per 1000 livebirths
Fraction of mortality
2000
2015
2019
Other
Measles
Diarrhoea
Congenital
Intrapartum−related
Preterm birth
AIDS
Injury
Malaria
Sepsis or meningitis
LRI
Kazakhstan (Neonatal)
All−cause NMR
Neonatal other
Neonatal diarrhoea
Neonatal tetanus
Neonatal congenital
Neonatal sepsis or meningitis
Neonatal intrapartum
Neonatal preterm
Neonatal LRI
−0.05
0.05
0.10
0.15
Mortality rate per 1000 live births
Fraction of mortality
2000
2015
2019
2000 − 2015
2015 − 2019 (not on target)
Deficit to target
2015 − 2019 (on target)

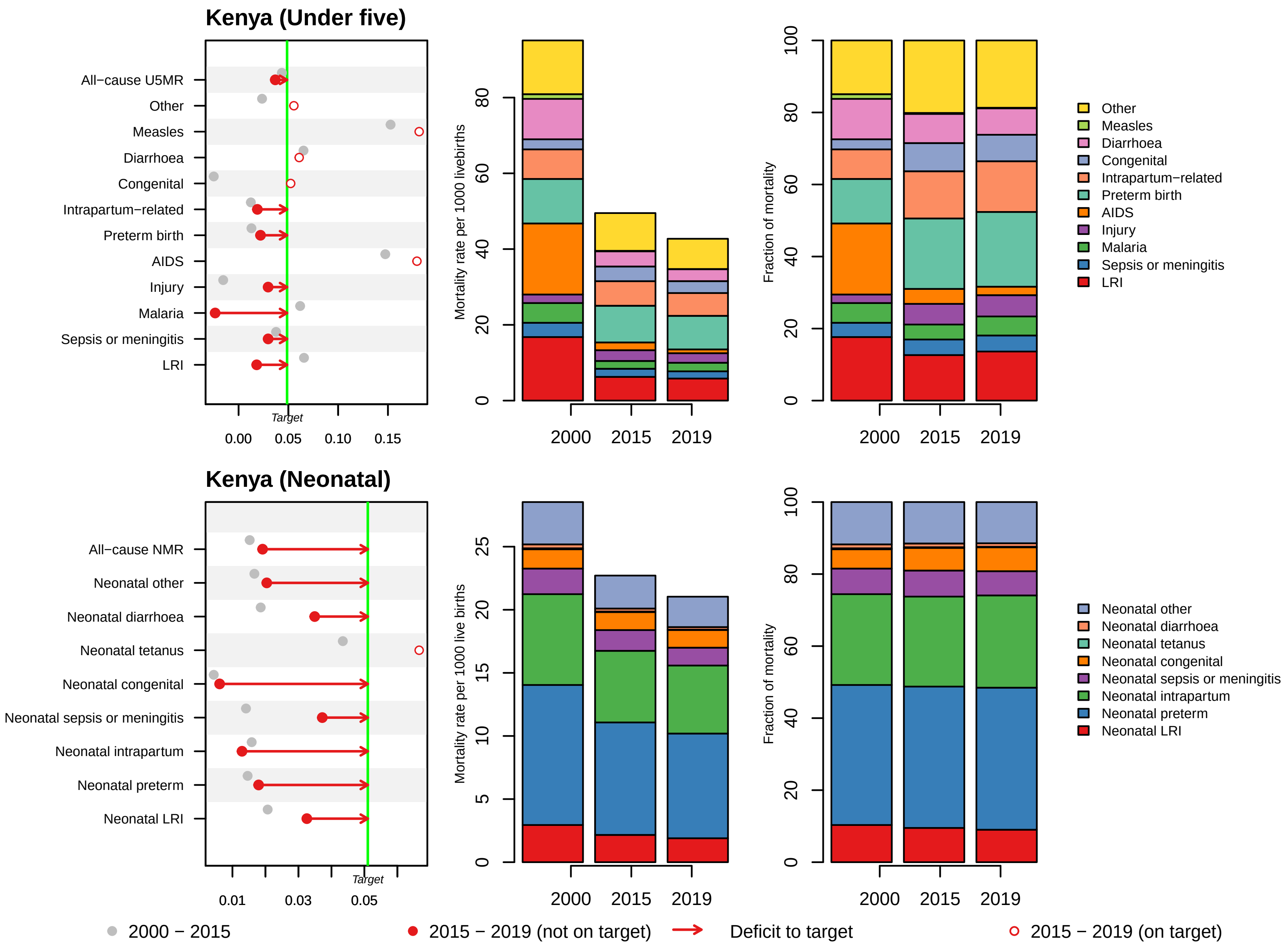
Kenya (Under five)
All−cause U5MR
Other
Measles
Diarrhoea
Congenital
Intrapartum−related
Preterm birth
AIDS
Injury
Malaria
Sepsis or meningitis
LRI
Target
0.00
0.05
0.10
0.15
Mortality rate per 1000 livebirths
Fraction of mortality
2000
2015
2019
Other
Measles
Diarrhoea
Congenital
Intrapartum−related
Preterm birth
AIDS
Injury
Malaria
Sepsis or meningitis
LRI
Kenya (Neonatal)
All−cause NMR
Neonatal other
Neonatal diarrhoea
Neonatal tetanus
Neonatal congenital
Neonatal sepsis or meningitis
Neonatal intrapartum
Neonatal preterm
Neonatal LRI
Target
0.01
0.03
0.05
Mortality rate per 1000 live births
Fraction of mortality
2000 − 2015
2015 − 2019 (not on target)
Deficit to target
2015 − 2019 (on target)

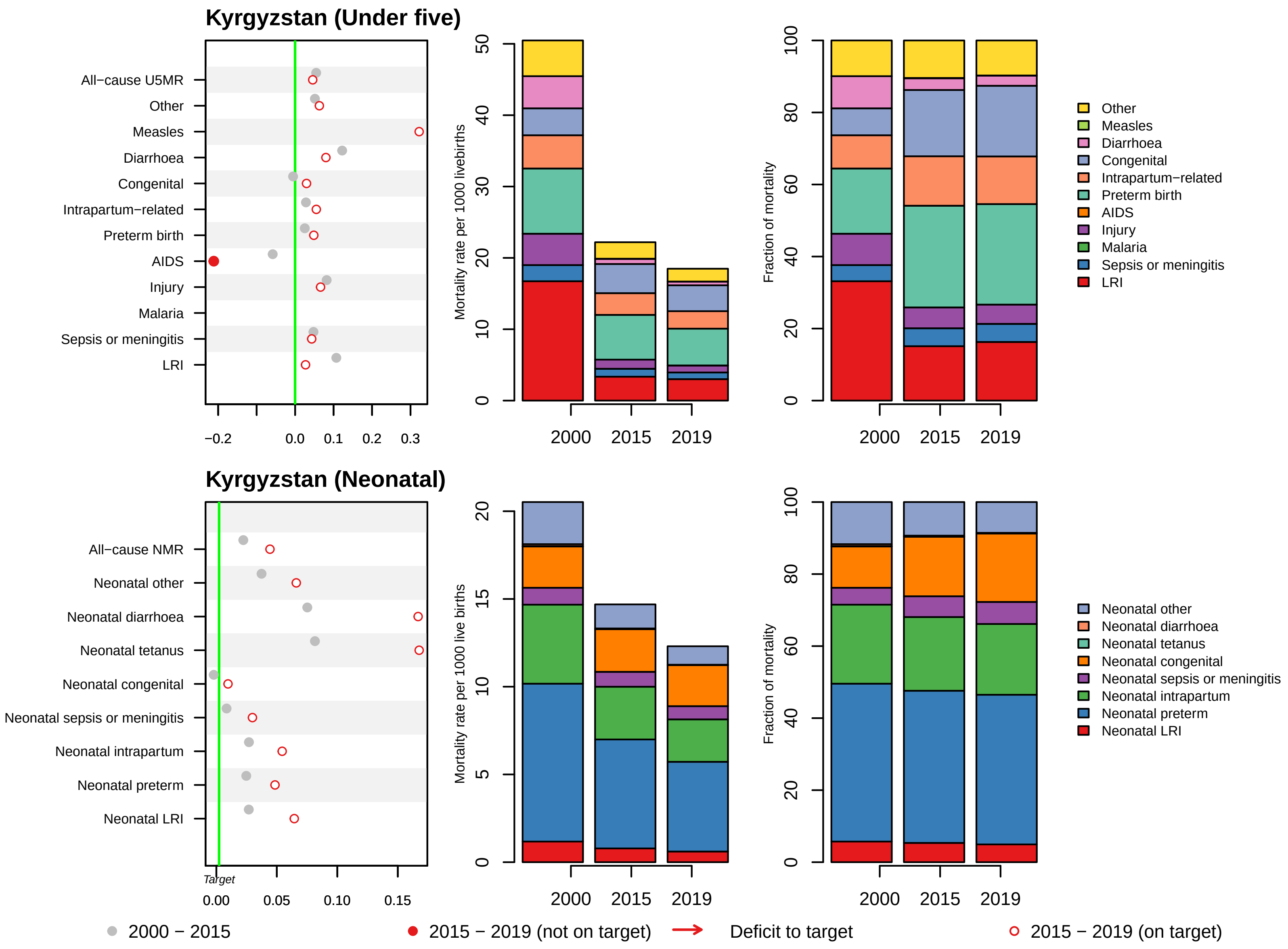
Kyrgyzstan (Under five)
All−cause U5MR
Other
Measles
Diarrhoea
Congenital
Intrapartum−related
Preterm birth
AIDS
Injury
Malaria
Sepsis or meningitis
LRI
−0.2
0.0
0.1
0.2
0.3
Mortality rate per 1000 livebirths
Fraction of mortality
2000
2015
2019
Other
Measles
Diarrhoea
Congenital
Intrapartum−related
Preterm birth
AIDS
Injury
Malaria
Sepsis or meningitis
LRI
Kyrgyzstan (Neonatal)
All−cause NMR
Neonatal other
Neonatal diarrhoea
Neonatal tetanus
Neonatal congenital
Neonatal sepsis or meningitis
Neonatal intrapartum
Neonatal preterm
Neonatal LRI
Target
0.00
0.05
0.10
0.15
Mortality rate per 1000 live births
Neonatal other
Neonatal diarrhoea
Neonatal tetanus
Neonatal congenital
Neonatal sepsis or meningitis
Neonatal intrapartum
Neonatal preterm
Neonatal LRI
2000 − 2015
2015 − 2019 (not on target)
Deficit to target
2015 − 2019 (on target)

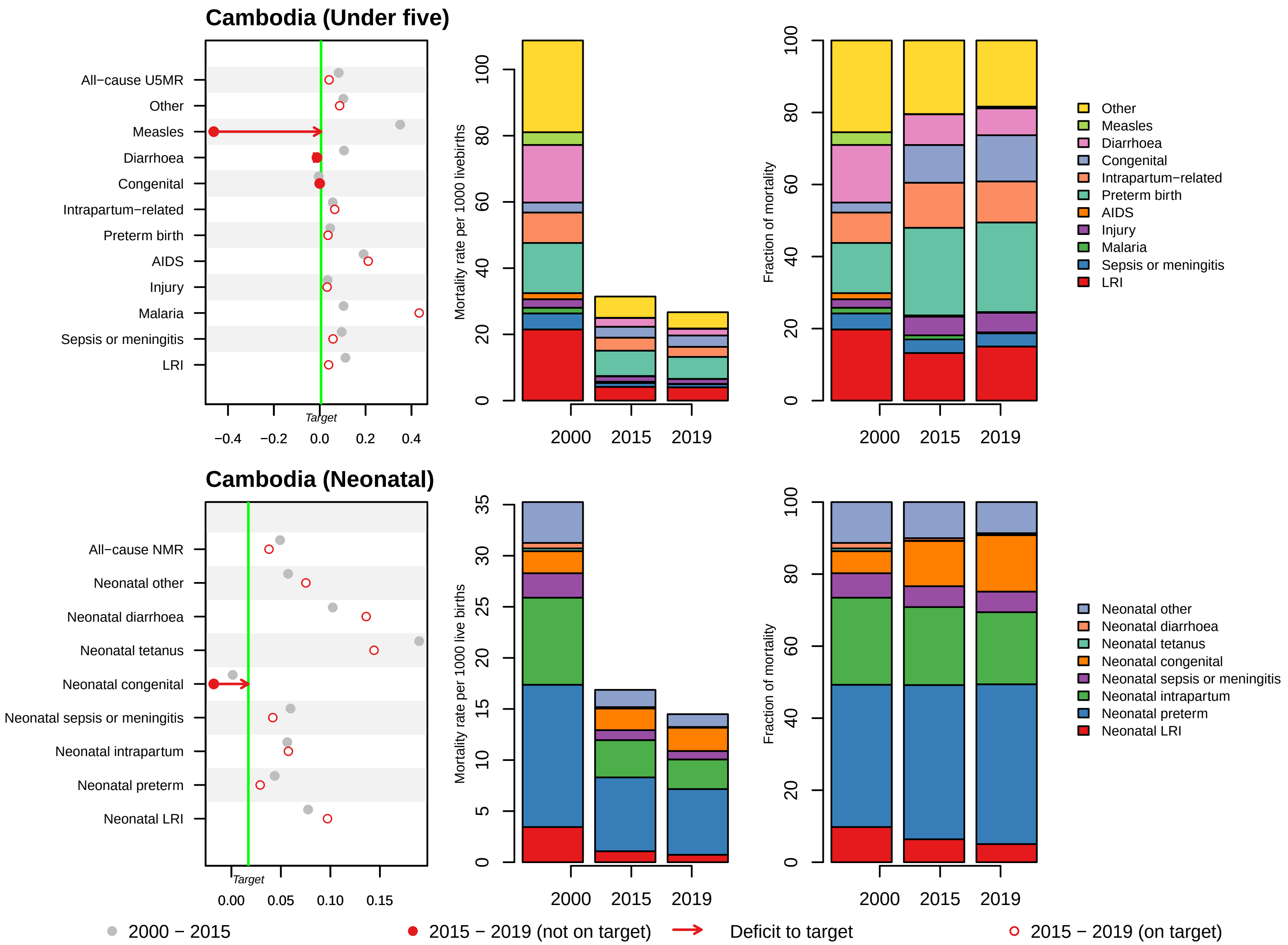
Cambodia (Under five)
All−cause U5MR
Other
Measles
Diarrhoea
Congenital
Intrapartum−related
Preterm birth
AIDS
Injury
Malaria
Sepsis or meningitis
LRI
Target
−0.4
−0.2
0.0
0.2
0.4
Mortality rate per 1000 livebirths
Fraction of mortality
2000
2015
2019
Other
Measles
Diarrhoea
Congenital
Intrapartum−related
Preterm birth
AIDS
Injury
Malaria
Sepsis or meningitis
LRI
Cambodia (Neonatal)
All−cause NMR
Neonatal other
Neonatal diarrhoea
Neonatal tetanus
Neonatal congenital
Neonatal sepsis or meningitis
Neonatal intrapartum
Neonatal preterm
Neonatal LRI
Target
0.00
0.05
0.10
0.15
Mortality rate per 1000 live births
Fraction of mortality
2000 − 2015
2015 − 2019 (not on target)
Deficit to target
2015 − 2019 (on target)

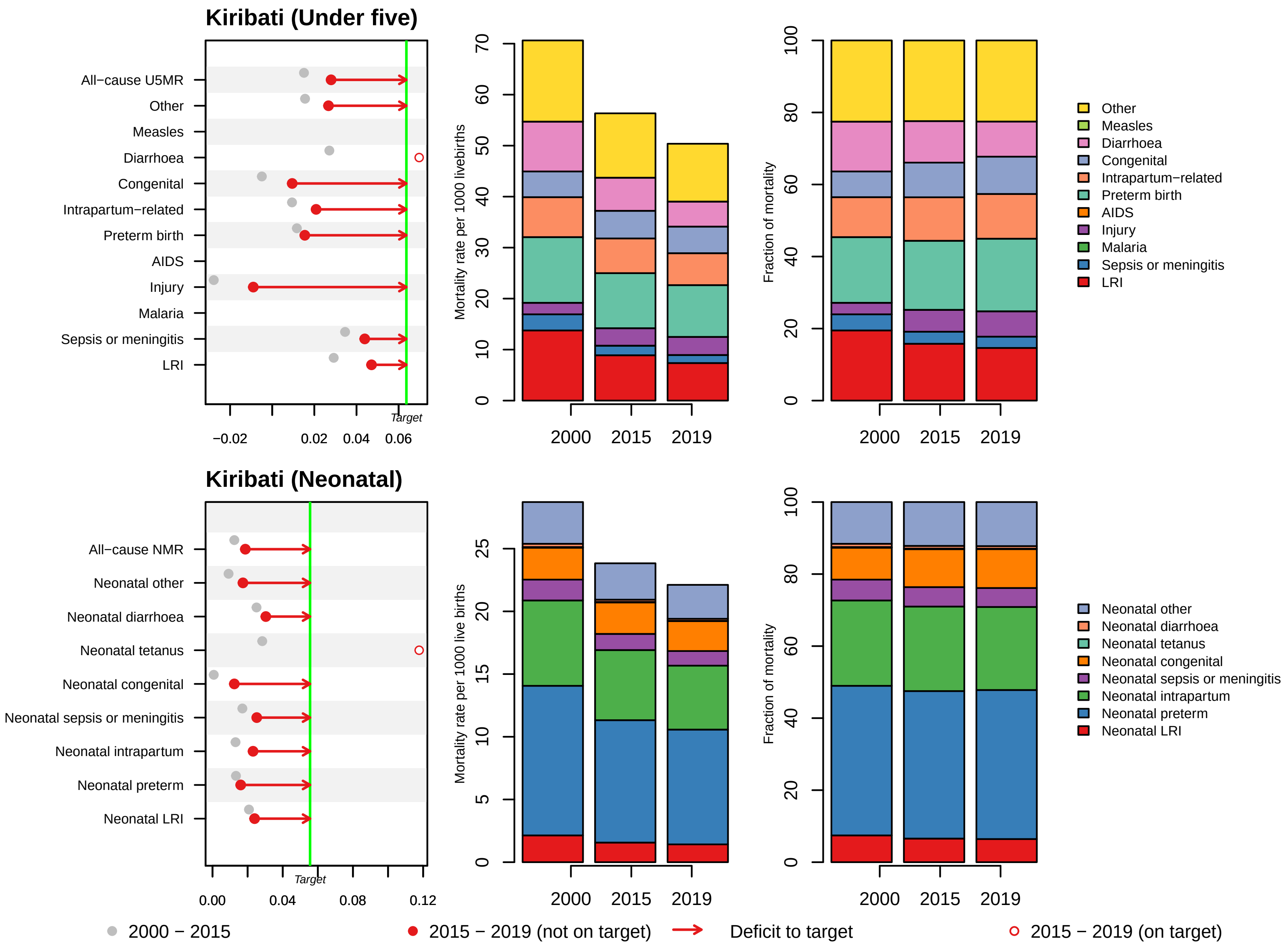
Kiribati (Under five)
All−cause U5MR
Other
Measles
Diarrhoea
Congenital
Intrapartum−related
Preterm birth
AIDS
Injury
Malaria
Sepsis or meningitis
LRI
Target
−0.02
0.02
0.04
0.06
Mortality rate per 1000 livebirths
0
10
20
30
40
50
60
70
2000
2015
2019
Fraction of mortality
0
20
40
60
80
100
Other
Measles
Diarrhoea
Congenital
Intrapartum−related
Preterm birth
AIDS
Injury
Malaria
Sepsis or meningitis
LRI
Kiribati (Neonatal)
All−cause NMR
Neonatal other
Neonatal diarrhoea
Neonatal tetanus
Neonatal congenital
Neonatal sepsis or meningitis
Neonatal intrapartum
Neonatal preterm
Neonatal LRI
Target
0.00
0.04
0.08
0.12
Mortality rate per 1000 live births
0
5
10
15
20
25
Neonatal other
Neonatal diarrhoea
Neonatal tetanus
Neonatal congenital
Neonatal sepsis or meningitis
Neonatal intrapartum
Neonatal preterm
Neonatal LRI
2000 − 2015
2015 − 2019 (not on target)
Deficit to target
2015 − 2019 (on target)

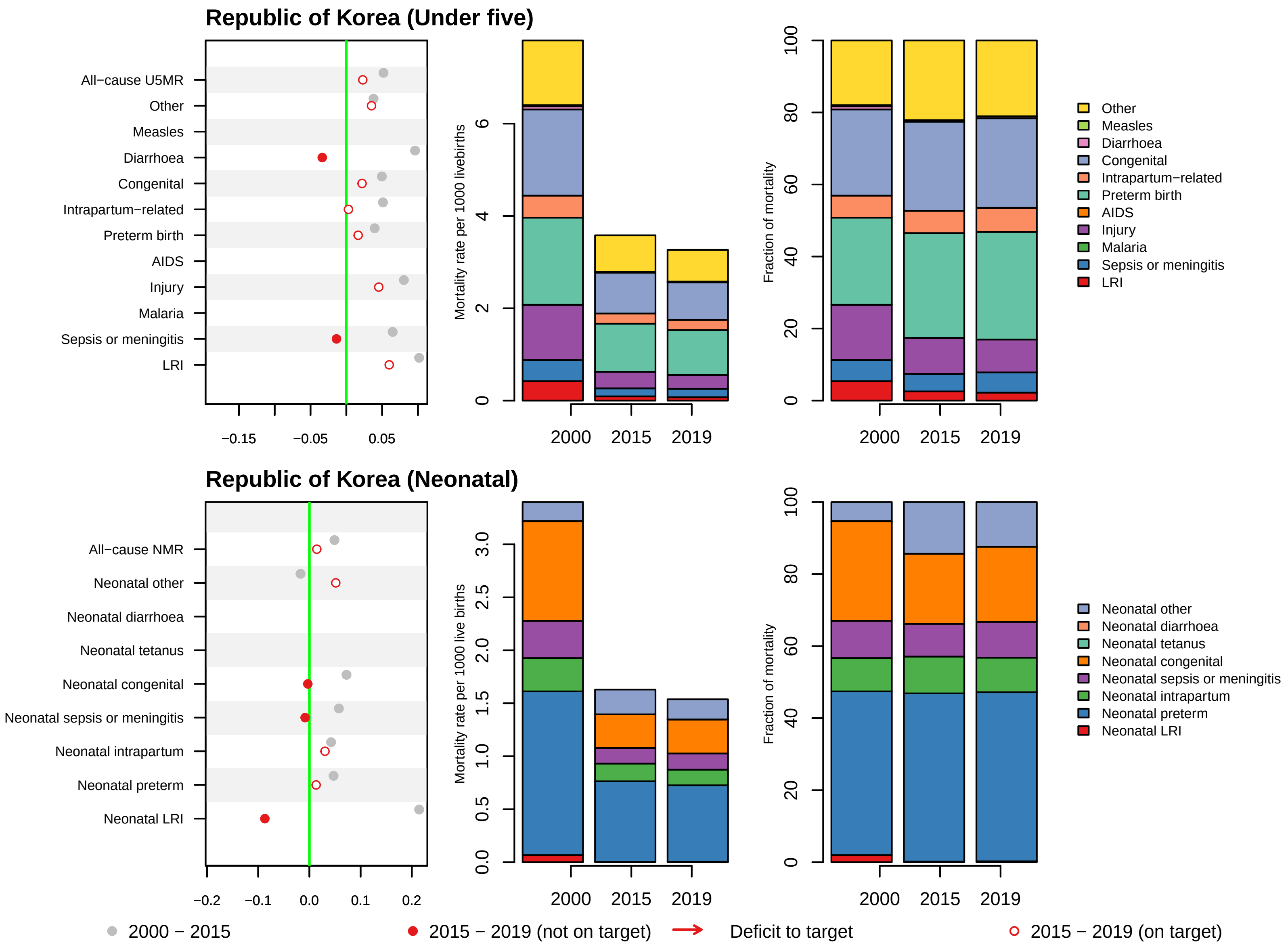

Republic of Korea (Under five)
All−cause U5MR
Other
Measles
Diarrhoea
Congenital
Intrapartum−related
Preterm birth
AIDS
Injury
Malaria
Sepsis or meningitis
LRI
−0.15
−0.05
0.05
Mortality rate per 1000 livebirths
0
2
4
6
2000
2015
2019
Fraction of mortality
0
20
40
60
80
100
Other
Measles
Diarrhoea
Congenital
Intrapartum−related
Preterm birth
AIDS
Injury
Malaria
Sepsis or meningitis
LRI
Republic of Korea (Neonatal)
All−cause NMR
Neonatal other
Neonatal diarrhoea
Neonatal tetanus
Neonatal congenital
Neonatal sepsis or meningitis
Neonatal intrapartum
Neonatal preterm
Neonatal LRI
−0.2
−0.1
0.0
0.1
0.2
Mortality rate per 1000 live births
0.0
0.5
1.0
1.5
2.0
2.5
3.0
Neonatal other
Neonatal diarrhoea
Neonatal tetanus
Neonatal congenital
Neonatal sepsis or meningitis
Neonatal intrapartum
Neonatal preterm
Neonatal LRI
2000 − 2015
2015 − 2019 (not on target)
Deficit to target
2015 − 2019 (on target)

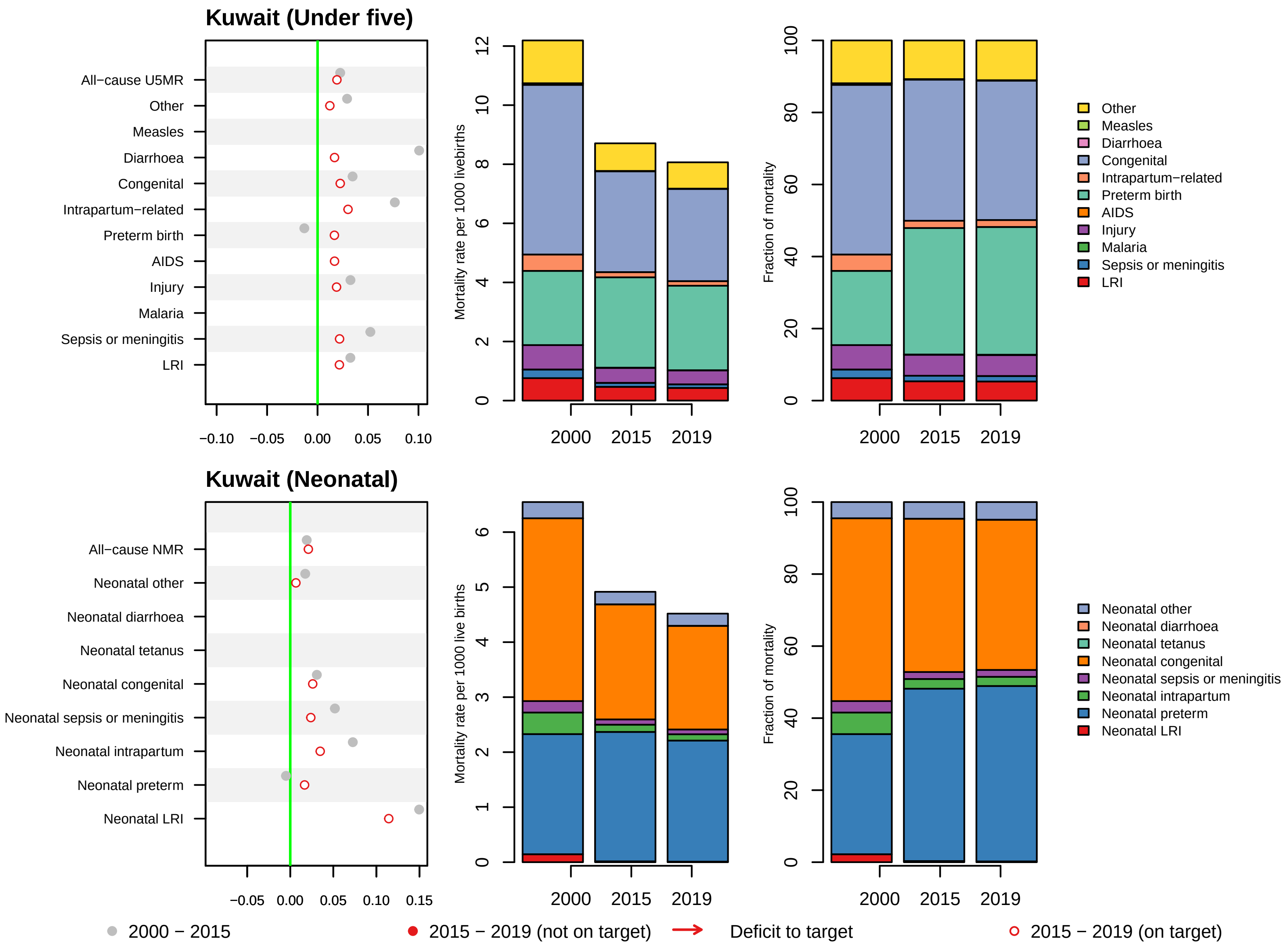

Kuwait (Under five)
All−cause U5MR
Other
Measles
Diarrhoea
Congenital
Intrapartum−related
Preterm birth
AIDS
Injury
Malaria
Sepsis or meningitis
LRI
−0.10
−0.05
0.00
0.05
0.10
Mortality rate per 1000 livebirths
0
2
4
6
8
10
12
2000
2015
2019
Fraction of mortality
0
20
40
60
80
100
Other
Measles
Diarrhoea
Congenital
Intrapartum−related
Preterm birth
AIDS
Injury
Malaria
Sepsis or meningitis
LRI
Kuwait (Neonatal)
All−cause NMR
Neonatal other
Neonatal diarrhoea
Neonatal tetanus
Neonatal congenital
Neonatal sepsis or meningitis
Neonatal intrapartum
Neonatal preterm
Neonatal LRI
−0.05
0.00
0.05
0.10
0.15
Mortality rate per 1000 live births
0
1
2
3
4
5
6
Neonatal other
Neonatal diarrhoea
Neonatal tetanus
Neonatal congenital
Neonatal sepsis or meningitis
Neonatal intrapartum
Neonatal preterm
Neonatal LRI
2000 − 2015
2015 − 2019 (not on target)
Deficit to target
2015 − 2019 (on target)

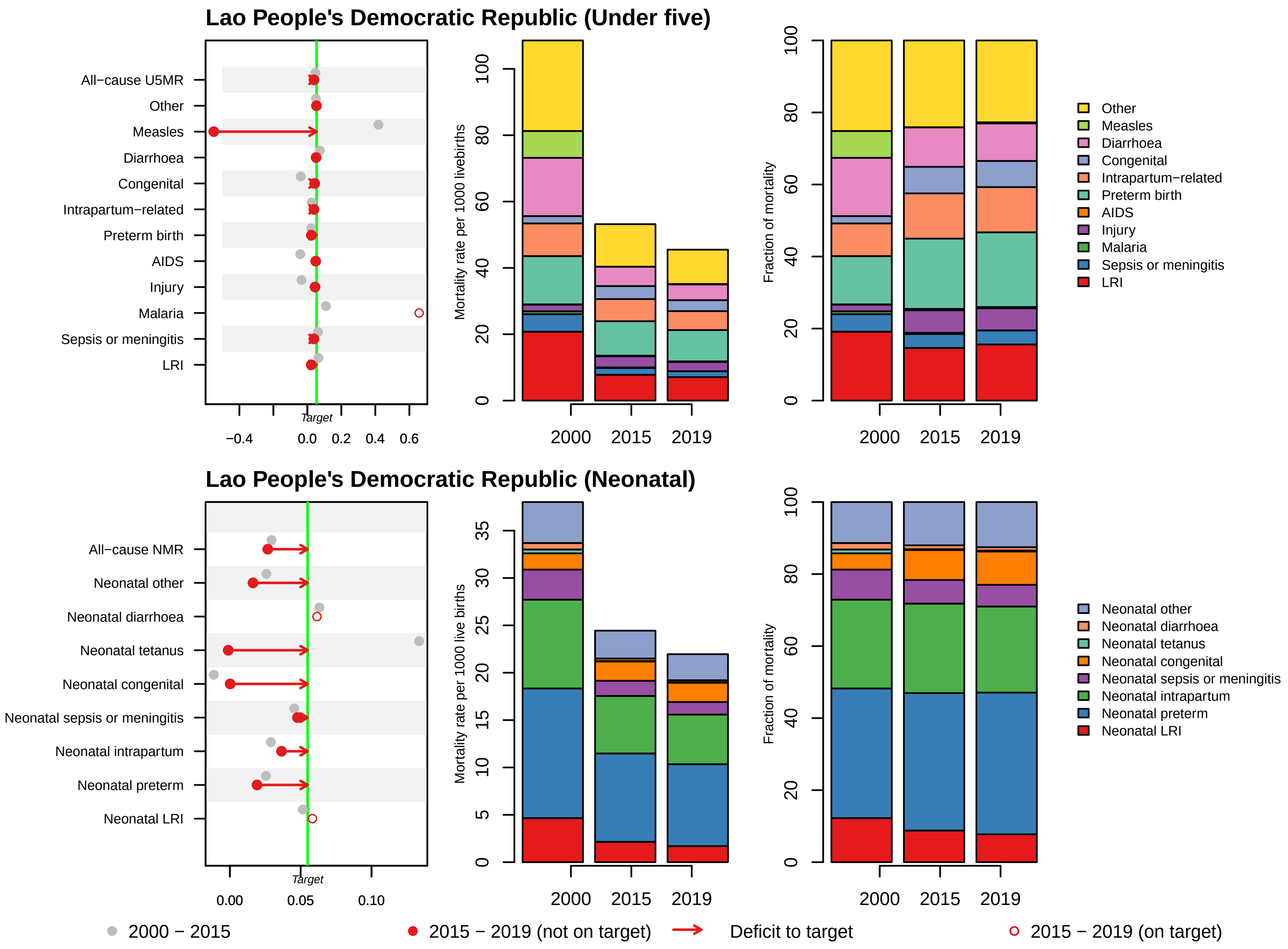
Lao People's Democratic Republic (Under five)
All−cause U5MR
Other
Measles
Diarrhoea
Congenital
Intrapartum−related
Preterm birth
AIDS
Injury
Malaria
Sepsis or meningitis
LRI
Target
−0.4
0.0
0.2
0.4
0.6
Mortality rate per 1000 livebirths
0
20
40
60
80
100
2000
2015
2019
Fraction of mortality
0
20
40
60
80
100
2000
2015
2019
Other
Measles
Diarrhoea
Congenital
Intrapartum−related
Preterm birth
AIDS
Injury
Malaria
Sepsis or meningitis
LRI
Lao People's Democratic Republic (Neonatal)
All−cause NMR
Neonatal other
Neonatal diarrhoea
Neonatal tetanus
Neonatal congenital
Neonatal sepsis or meningitis
Neonatal intrapartum
Neonatal preterm
Neonatal LRI
Target
0.00
0.05
0.10
Mortality rate per 1000 live births
0
5
10
15
20
25
30
35
2000
2015
2019
Fraction of mortality
0
20
40
60
80
100
2000
2015
2019
Neonatal other
Neonatal diarrhoea
Neonatal tetanus
Neonatal congenital
Neonatal sepsis or meningitis
Neonatal intrapartum
Neonatal preterm
Neonatal LRI
2000 − 2015
2015 − 2019 (not on target)
Deficit to target
2015 − 2019 (on target)

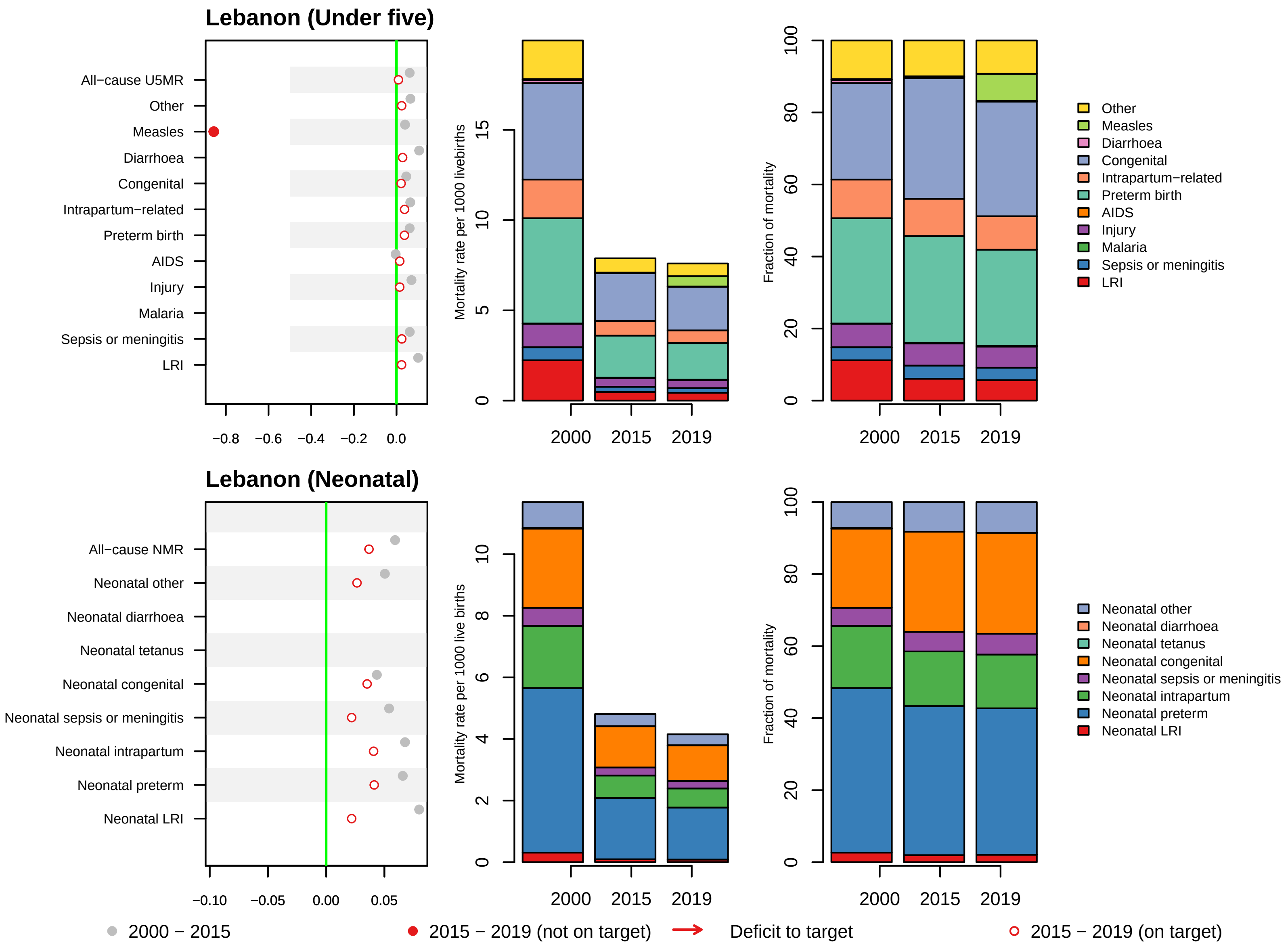
Lebanon (Under five)
All−cause U5MR
Other
Measles
Diarrhoea
Congenital
Intrapartum−related
Preterm birth
AIDS
Injury
Malaria
Sepsis or meningitis
LRI
−0.8
−0.6
−0.4
−0.2
0.0
Mortality rate per 1000 livebirths
Fraction of mortality
2000
2015
2019
Lebanon (Neonatal)
All−cause NMR
Neonatal other
Neonatal diarrhoea
Neonatal tetanus
Neonatal congenital
Neonatal sepsis or meningitis
Neonatal intrapartum
Neonatal preterm
Neonatal LRI
−0.10
−0.05
0.00
0.05
Mortality rate per 1000 live births
2000 − 2015
2015 − 2019 (not on target)
Deficit to target
2015 − 2019 (on target)

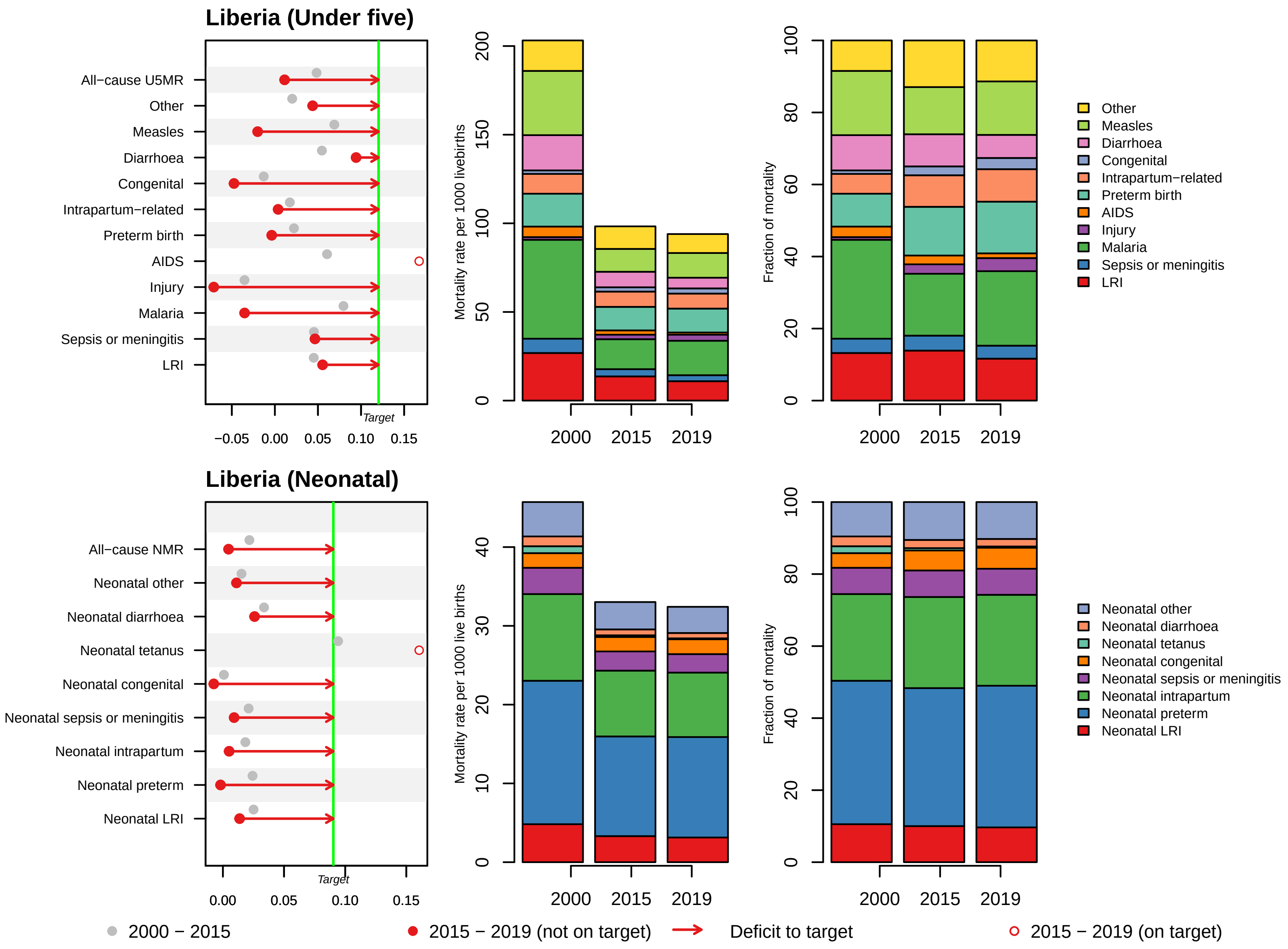

Liberia (Under five)
All−cause U5MR
Other
Measles
Diarrhoea
Congenital
Intrapartum−related
Preterm birth
AIDS
Injury
Malaria
Sepsis or meningitis
LRI
Target
−0.05
0.00
0.05
0.10
0.15
Mortality rate per 1000 livebirths
0
50
100
150
200
2000
2015
2019
Fraction of mortality
0
20
40
60
80
100
Other
Measles
Diarrhoea
Congenital
Intrapartum−related
Preterm birth
AIDS
Injury
Malaria
Sepsis or meningitis
LRI
Liberia (Neonatal)
All−cause NMR
Neonatal other
Neonatal diarrhoea
Neonatal tetanus
Neonatal congenital
Neonatal sepsis or meningitis
Neonatal intrapartum
Neonatal preterm
Neonatal LRI
Target
0.00
0.05
0.10
0.15
Mortality rate per 1000 live births
0
10
20
30
40
Neonatal other
Neonatal diarrhoea
Neonatal tetanus
Neonatal congenital
Neonatal sepsis or meningitis
Neonatal intrapartum
Neonatal preterm
Neonatal LRI
2000 − 2015
2015 − 2019 (not on target)
Deficit to target
2015 − 2019 (on target)

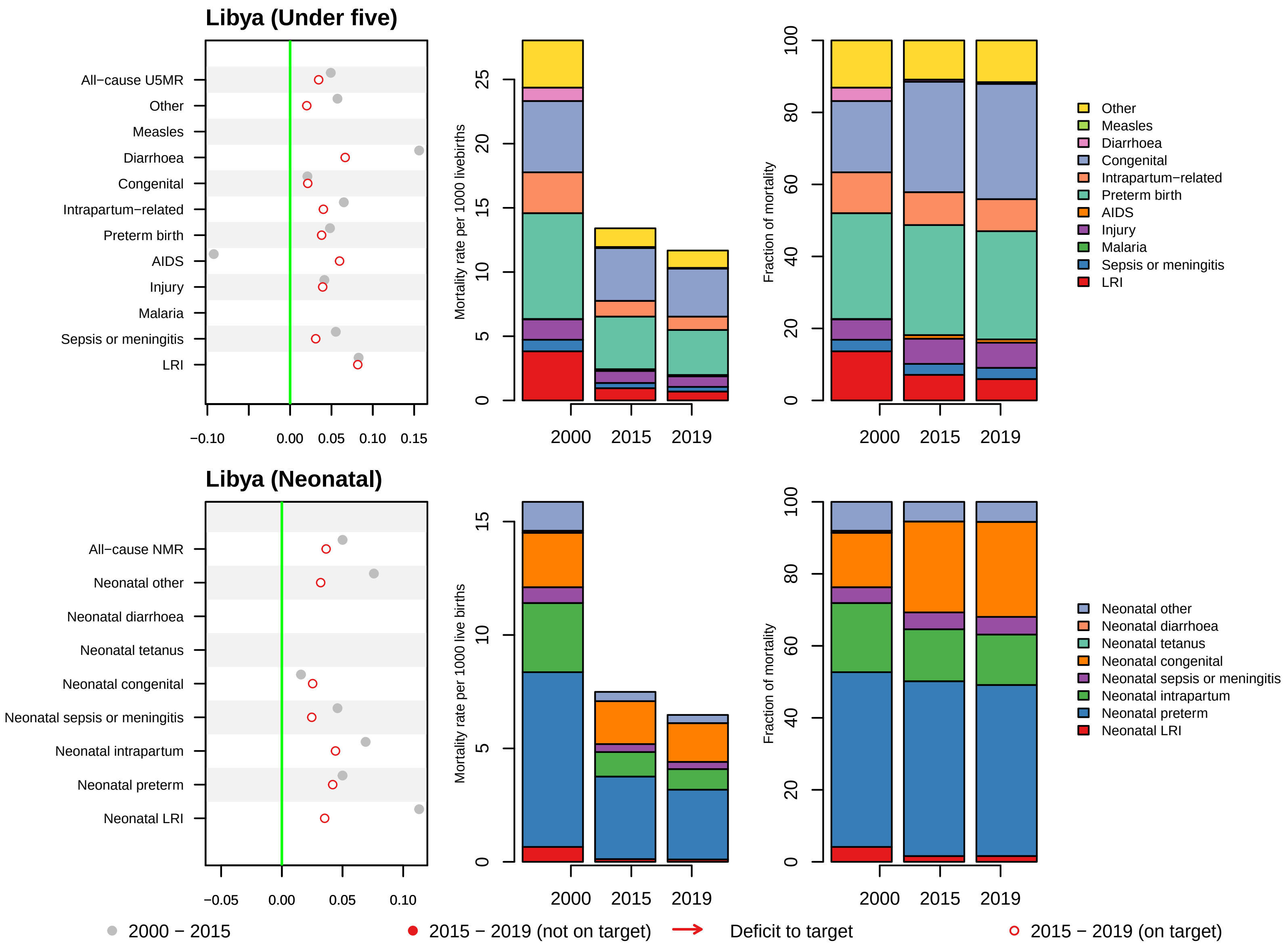
Libya (Under five)
All−cause U5MR
Other
Measles
Diarrhoea
Congenital
Intrapartum−related
Preterm birth
AIDS
Injury
Malaria
Sepsis or meningitis
LRI
−0.10
0.00
0.05
0.10
0.15
Mortality rate per 1000 livebirths
0
5
10
15
20
25
2000
2015
2019
Fraction of mortality
0
20
40
60
80
100
Other
Measles
Diarrhoea
Congenital
Intrapartum−related
Preterm birth
AIDS
Injury
Malaria
Sepsis or meningitis
LRI
Libya (Neonatal)
All−cause NMR
Neonatal other
Neonatal diarrhoea
Neonatal tetanus
Neonatal congenital
Neonatal sepsis or meningitis
Neonatal intrapartum
Neonatal preterm
Neonatal LRI
−0.05
0.00
0.05
0.10
Mortality rate per 1000 live births
0
5
10
15
Neonatal other
Neonatal diarrhoea
Neonatal tetanus
Neonatal congenital
Neonatal sepsis or meningitis
Neonatal intrapartum
Neonatal preterm
Neonatal LRI
2000 − 2015
2015 − 2019 (not on target)
Deficit to target
2015 − 2019 (on target)

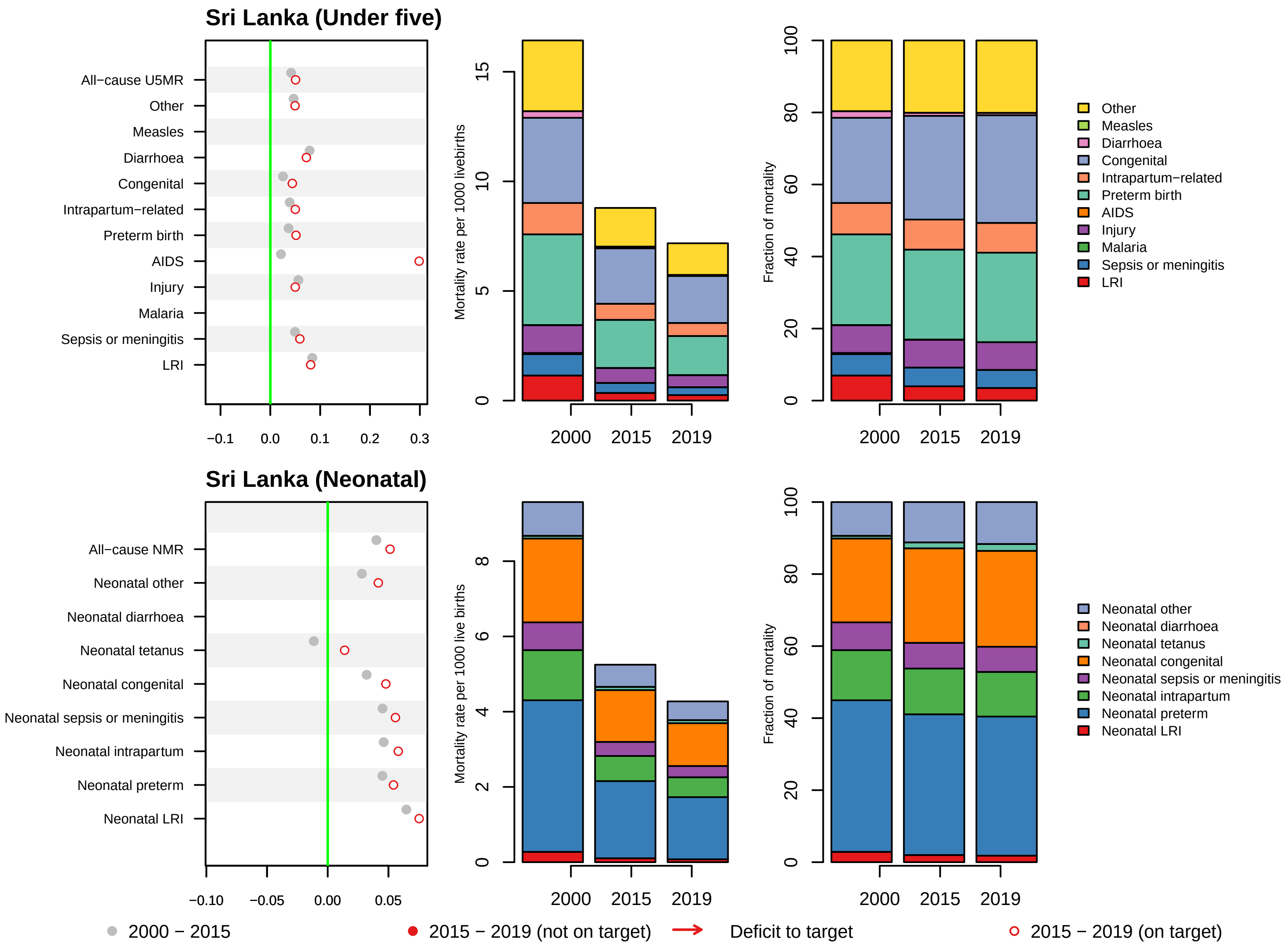

Sri Lanka (Under five)
All−cause U5MR
Other
Measles
Diarrhoea
Congenital
Intrapartum−related
Preterm birth
AIDS
Injury
Malaria
Sepsis or meningitis
LRI
−0.1
0.0
0.1
0.2
0.3
Mortality rate per 1000 livebirths
0
5
10
15
2000
2015
2019
Fraction of mortality
0
20
40
60
80
100
Other
Measles
Diarrhoea
Congenital
Intrapartum−related
Preterm birth
AIDS
Injury
Malaria
Sepsis or meningitis
LRI
Sri Lanka (Neonatal)
All−cause NMR
Neonatal other
Neonatal diarrhoea
Neonatal tetanus
Neonatal congenital
Neonatal sepsis or meningitis
Neonatal intrapartum
Neonatal preterm
Neonatal LRI
−0.10
−0.05
0.00
0.05
Mortality rate per 1000 live births
0
2
4
6
8
Neonatal other
Neonatal diarrhoea
Neonatal tetanus
Neonatal congenital
Neonatal sepsis or meningitis
Neonatal intrapartum
Neonatal preterm
Neonatal LRI
2000 − 2015
2015 − 2019 (not on target)
Deficit to target
2015 − 2019 (on target)

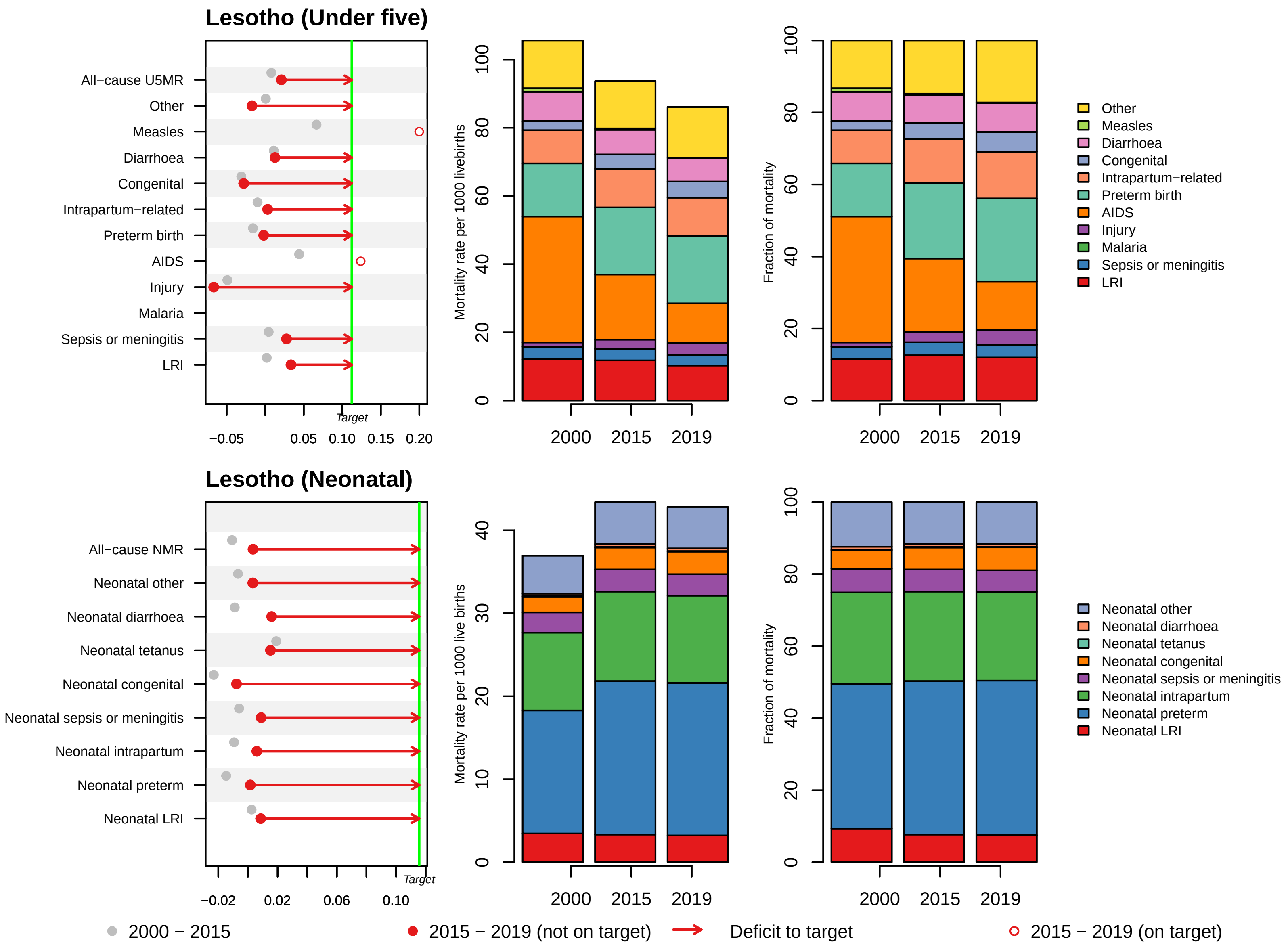

Lesotho (Under five)
All−cause U5MR
Other
Measles
Diarrhoea
Congenital
Intrapartum−related
Preterm birth
AIDS
Injury
Malaria
Sepsis or meningitis
LRI
Target
−0.05
0.05
0.10
0.15
0.20
Mortality rate per 1000 livebirths
Fraction of mortality
2000
2015
2019
Other
Measles
Diarrhoea
Congenital
Intrapartum−related
Preterm birth
AIDS
Injury
Malaria
Sepsis or meningitis
LRI
Lesotho (Neonatal)
All−cause NMR
Neonatal other
Neonatal diarrhoea
Neonatal tetanus
Neonatal congenital
Neonatal sepsis or meningitis
Neonatal intrapartum
Neonatal preterm
Neonatal LRI
Target
−0.02
0.02
0.06
0.10
Mortality rate per 1000 live births
Neonatal other
Neonatal diarrhoea
Neonatal tetanus
Neonatal congenital
Neonatal sepsis or meningitis
Neonatal intrapartum
Neonatal preterm
Neonatal LRI
2000 − 2015
2015 − 2019 (not on target)
Deficit to target
2015 − 2019 (on target)

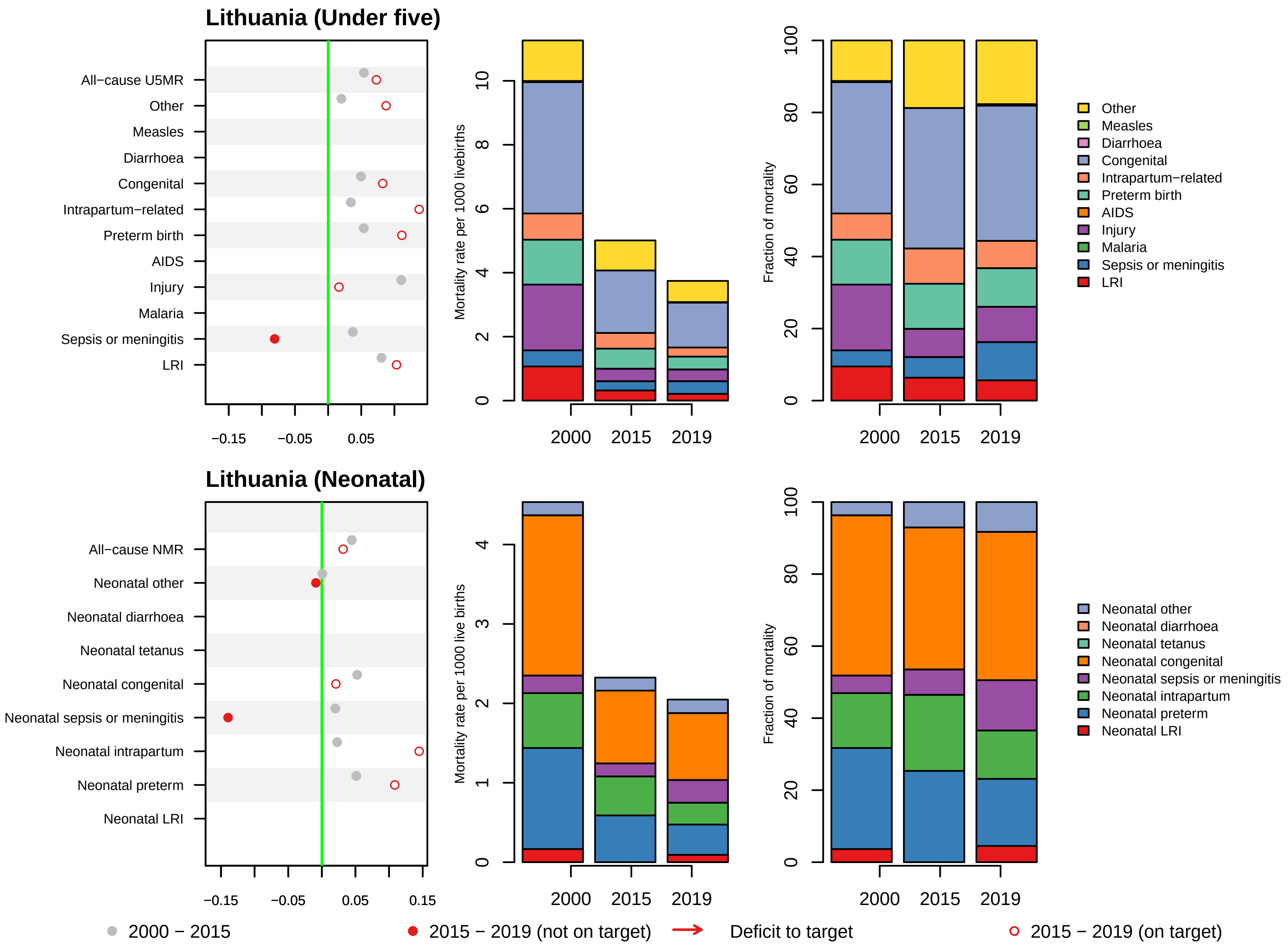

Lithuania (Under five)
All−cause U5MR
Other
Measles
Diarrhoea
Congenital
Intrapartum−related
Preterm birth
AIDS
Injury
Malaria
Sepsis or meningitis
LRI
−0.15
−0.05
0.05
Mortality rate per 1000 livebirths
Fraction of mortality
2000
2015
2019
Other
Measles
Diarrhoea
Congenital
Intrapartum−related
Preterm birth
AIDS
Injury
Malaria
Sepsis or meningitis
LRI
Lithuania (Neonatal)
All−cause NMR
Neonatal other
Neonatal diarrhoea
Neonatal tetanus
Neonatal congenital
Neonatal sepsis or meningitis
Neonatal intrapartum
Neonatal preterm
Neonatal LRI
−0.15
−0.05
0.05
0.15
Mortality rate per 1000 live births
Fraction of mortality
2000
2015
2019
Neonatal other
Neonatal diarrhoea
Neonatal tetanus
Neonatal congenital
Neonatal sepsis or meningitis
Neonatal intrapartum
Neonatal preterm
Neonatal LRI
2000 − 2015
2015 − 2019 (not on target)
Deficit to target
2015 − 2019 (on target)

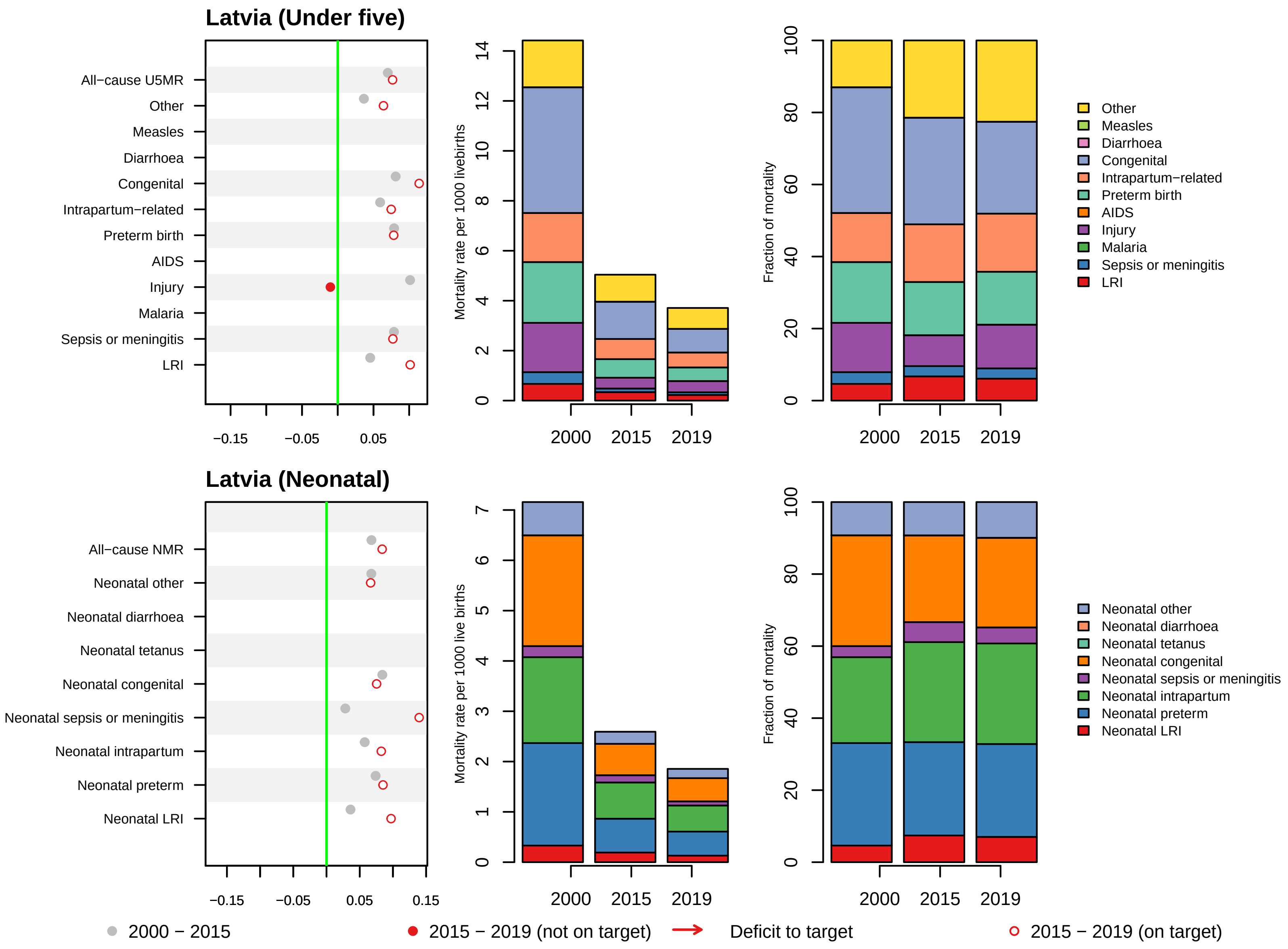

Latvia (Under five)
All−cause U5MR
Other
Measles
Diarrhoea
Congenital
Intrapartum−related
Preterm birth
AIDS
Injury
Malaria
Sepsis or meningitis
LRI
−0.15
−0.05
0.05
Mortality rate per 1000 livebirths
Fraction of mortality
2000
2015
2019
Other
Measles
Diarrhoea
Congenital
Intrapartum−related
Preterm birth
AIDS
Injury
Malaria
Sepsis or meningitis
LRI
Latvia (Neonatal)
All−cause NMR
Neonatal other
Neonatal diarrhoea
Neonatal tetanus
Neonatal congenital
Neonatal sepsis or meningitis
Neonatal intrapartum
Neonatal preterm
Neonatal LRI
−0.15
−0.05
0.05
0.15
Mortality rate per 1000 live births
Fraction of mortality
Neonatal other
Neonatal diarrhoea
Neonatal tetanus
Neonatal congenital
Neonatal sepsis or meningitis
Neonatal intrapartum
Neonatal preterm
Neonatal LRI
2000 − 2015
2015 − 2019 (not on target)
Deficit to target
2015 − 2019 (on target)

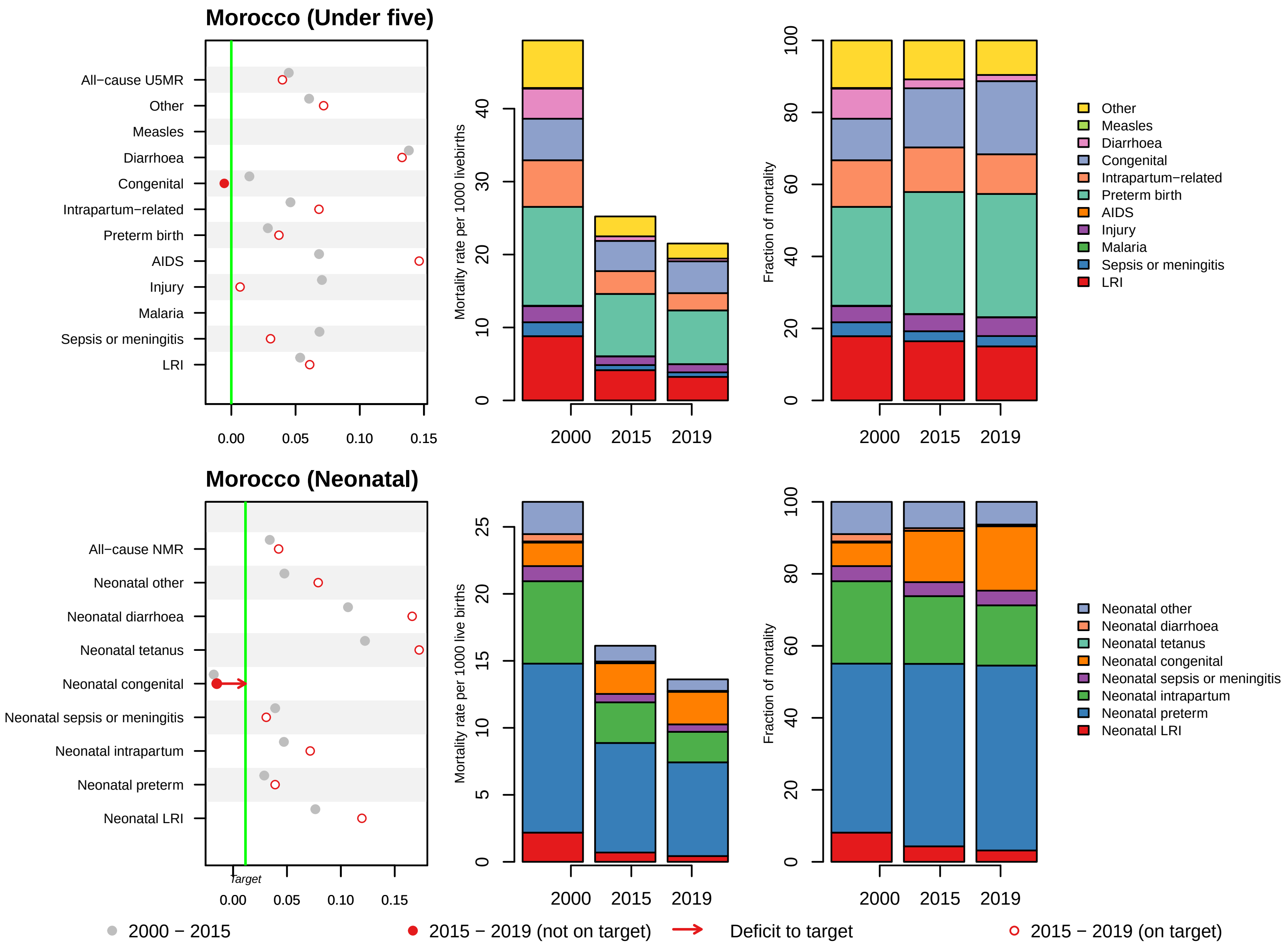

Morocco (Under five)
All−cause U5MR
Other
Measles
Diarrhoea
Congenital
Intrapartum−related
Preterm birth
AIDS
Injury
Malaria
Sepsis or meningitis
LRI
0.00
0.05
0.10
0.15
Mortality rate per 1000 livebirths
0
10
20
30
40
2000
2015
2019
Fraction of mortality
0
20
40
60
80
100
Other
Measles
Diarrhoea
Congenital
Intrapartum−related
Preterm birth
AIDS
Injury
Malaria
Sepsis or meningitis
LRI
Morocco (Neonatal)
All−cause NMR
Neonatal other
Neonatal diarrhoea
Neonatal tetanus
Neonatal congenital
Neonatal sepsis or meningitis
Neonatal intrapartum
Neonatal preterm
Neonatal LRI
Target
0.00
0.05
0.10
0.15
Mortality rate per 1000 live births
0
5
10
15
20
25
2000
2015
2019
Fraction of mortality
0
20
40
60
80
100
Neonatal other
Neonatal diarrhoea
Neonatal tetanus
Neonatal congenital
Neonatal sepsis or meningitis
Neonatal intrapartum
Neonatal preterm
Neonatal LRI
2000 − 2015
2015 − 2019 (not on target)
Deficit to target
2015 − 2019 (on target)

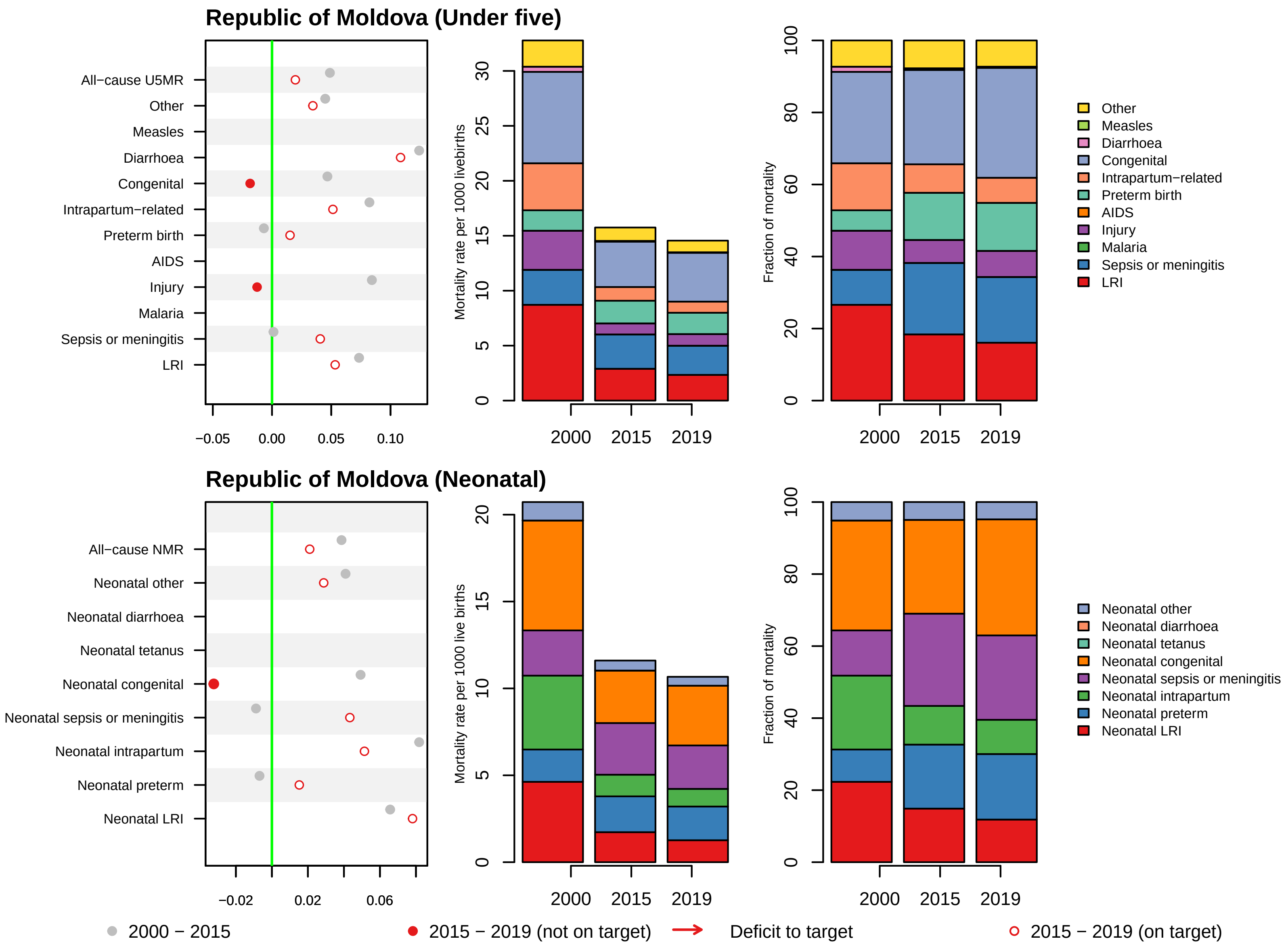

Republic of Moldova (Under five)
All−cause U5MR
Other
Measles
Diarrhoea
Congenital
Intrapartum−related
Preterm birth
AIDS
Injury
Malaria
Sepsis or meningitis
LRI
−0.05
0.00
0.05
0.10
Mortality rate per 1000 livebirths
Fraction of mortality
2000
2015
2019
Other
Measles
Diarrhoea
Congenital
Intrapartum−related
Preterm birth
AIDS
Injury
Malaria
Sepsis or meningitis
LRI
Republic of Moldova (Neonatal)
All−cause NMR
Neonatal other
Neonatal diarrhoea
Neonatal tetanus
Neonatal congenital
Neonatal sepsis or meningitis
Neonatal intrapartum
Neonatal preterm
Neonatal LRI
−0.02
0.02
0.06
Mortality rate per 1000 live births
Fraction of mortality
2000
2015
2019
Neonatal other
Neonatal diarrhoea
Neonatal tetanus
Neonatal congenital
Neonatal sepsis or meningitis
Neonatal intrapartum
Neonatal preterm
Neonatal LRI
2000 − 2015
2015 − 2019 (not on target)
Deficit to target
2015 − 2019 (on target)

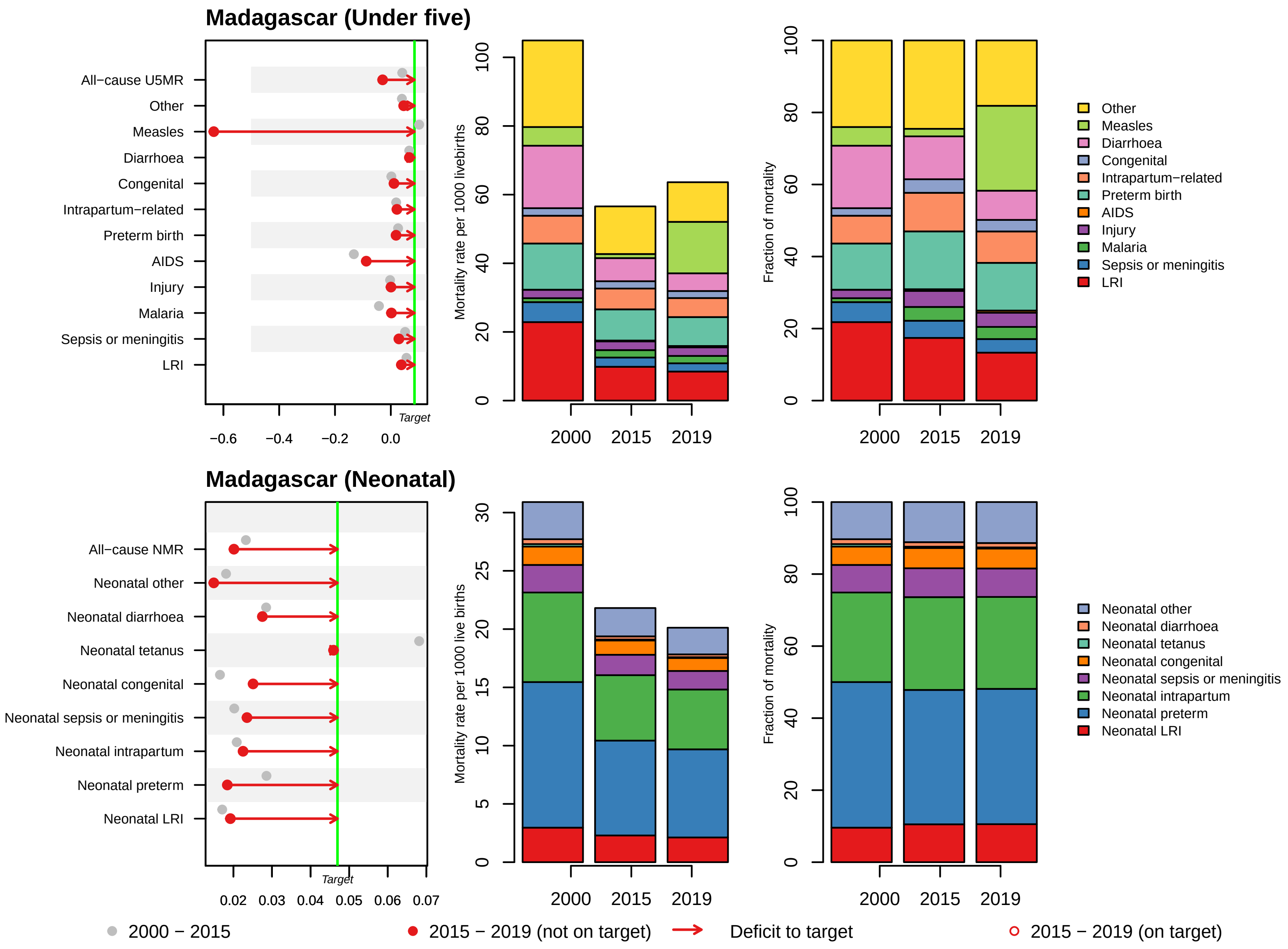

Madagascar (Under five)
All−cause U5MR
Other
Measles
Diarrhoea
Congenital
Intrapartum−related
Preterm birth
AIDS
Injury
Malaria
Sepsis or meningitis
LRI
Target
−0.6
−0.4
−0.2
0.0
Mortality rate per 1000 livebirths
0
20
40
60
80
100
2000
2015
2019
Fraction of mortality
Other
Measles
Diarrhoea
Congenital
Intrapartum−related
Preterm birth
AIDS
Injury
Malaria
Sepsis or meningitis
LRI
Madagascar (Neonatal)
All−cause NMR
Neonatal other
Neonatal diarrhoea
Neonatal tetanus
Neonatal congenital
Neonatal sepsis or meningitis
Neonatal intrapartum
Neonatal preterm
Neonatal LRI
Target
0.02
0.03
0.04
0.05
0.06
0.07
Mortality rate per 1000 live births
0
5
10
15
20
25
30
Neonatal other
Neonatal diarrhoea
Neonatal tetanus
Neonatal congenital
Neonatal sepsis or meningitis
Neonatal intrapartum
Neonatal preterm
Neonatal LRI
2000 − 2015
2015 − 2019 (not on target)
Deficit to target
2015 − 2019 (on target)

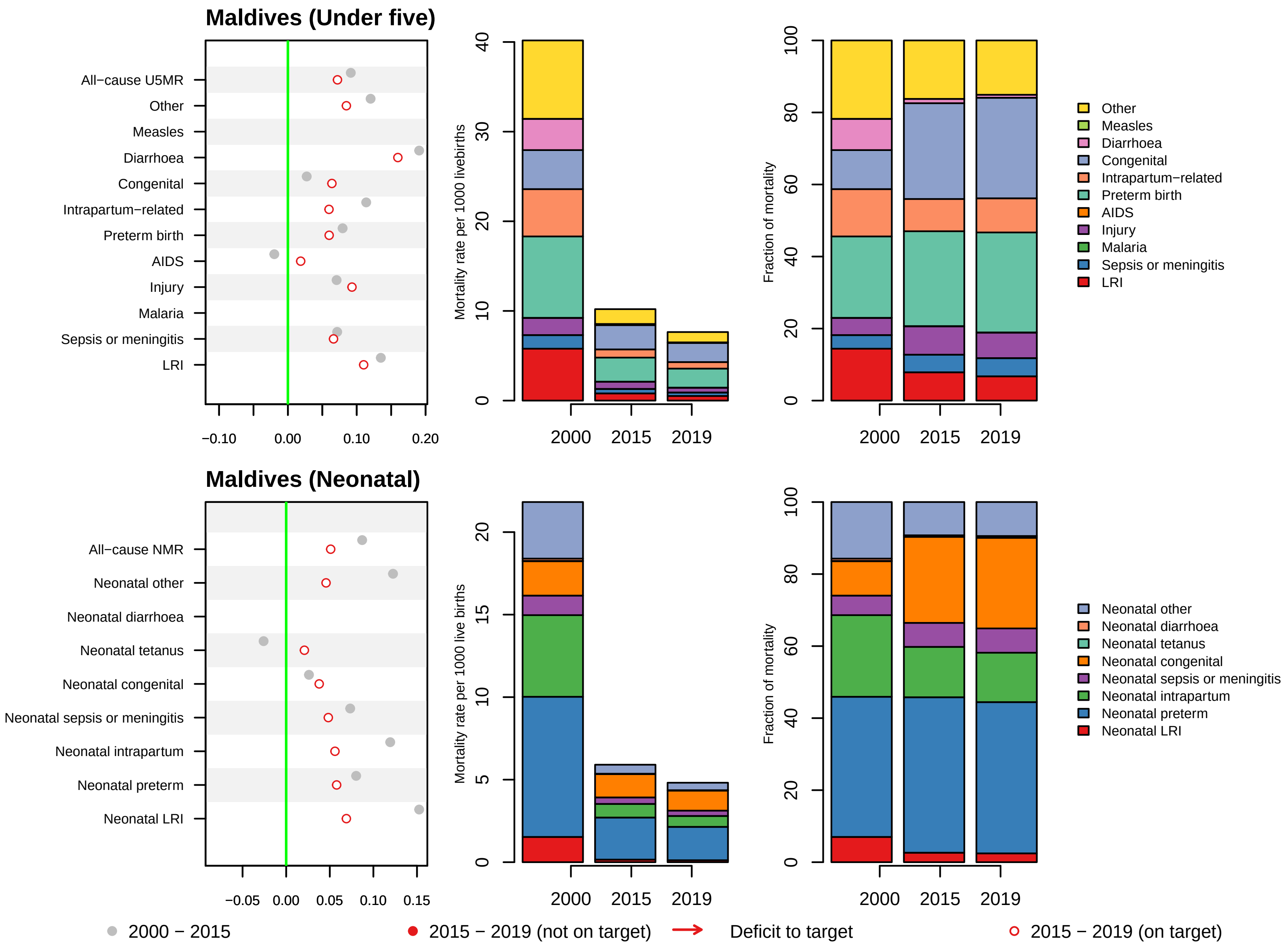
Maldives (Under five)
All−cause U5MR
Other
Measles
Diarrhoea
Congenital
Intrapartum−related
Preterm birth
AIDS
Injury
Malaria
Sepsis or meningitis
LRI
−0.10
0.00
0.10
0.20
Mortality rate per 1000 livebirths
Fraction of mortality
2000
2015
2019
Other
Measles
Diarrhoea
Congenital
Intrapartum−related
Preterm birth
AIDS
Injury
Malaria
Sepsis or meningitis
LRI
Maldives (Neonatal)
All−cause NMR
Neonatal other
Neonatal diarrhoea
Neonatal tetanus
Neonatal congenital
Neonatal sepsis or meningitis
Neonatal intrapartum
Neonatal preterm
Neonatal LRI
−0.05
0.00
0.05
0.10
0.15
Mortality rate per 1000 live births
Neonatal other
Neonatal diarrhoea
Neonatal tetanus
Neonatal congenital
Neonatal sepsis or meningitis
Neonatal intrapartum
Neonatal preterm
Neonatal LRI
2000 − 2015
2015 − 2019 (not on target)
Deficit to target
2015 − 2019 (on target)

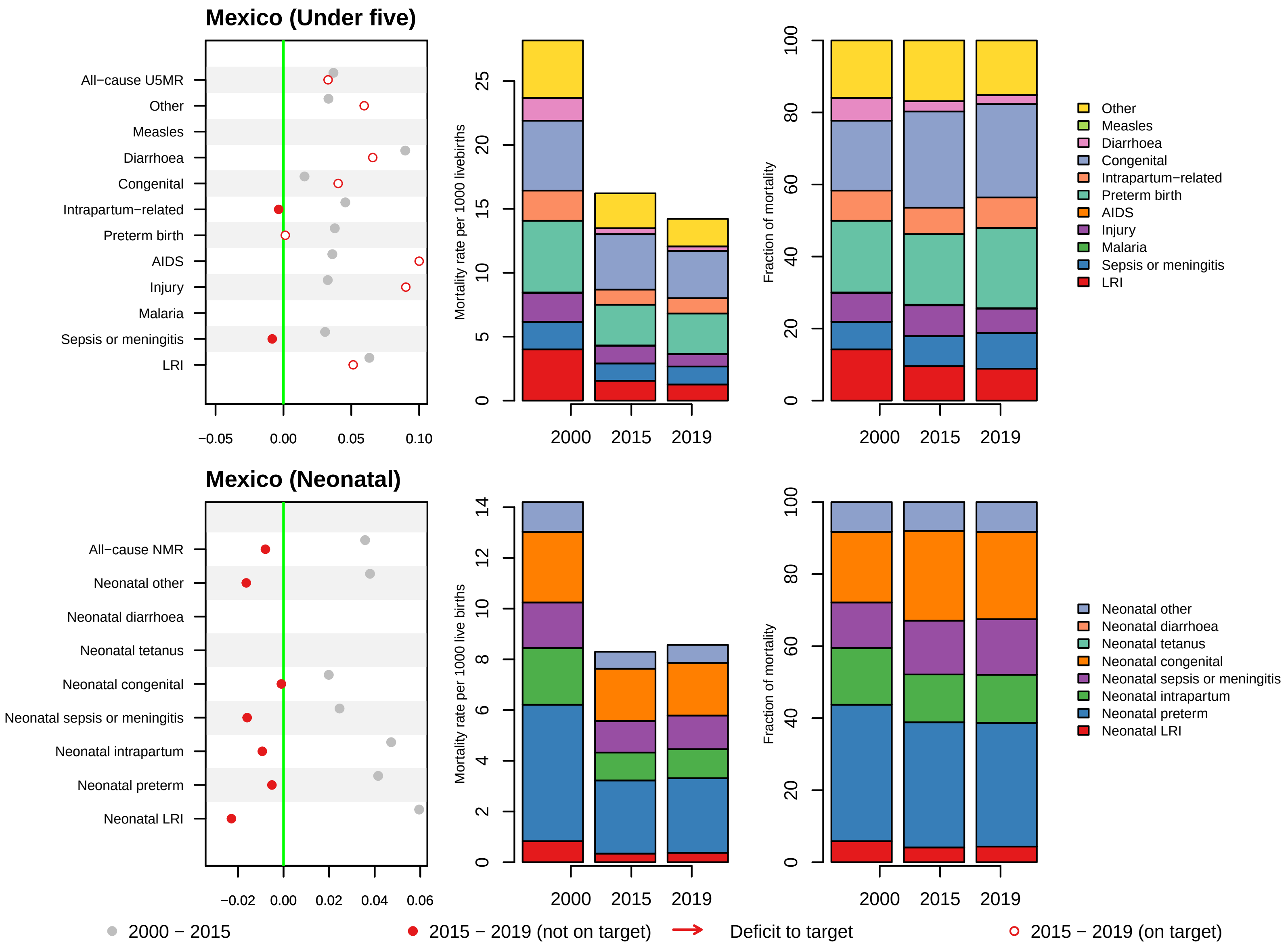
Mexico (Under five)
All−cause U5MR
Other
Measles
Diarrhoea
Congenital
Intrapartum−related
Preterm birth
AIDS
Injury
Malaria
Sepsis or meningitis
LRI
−0.05
0.00
0.05
0.10
Mortality rate per 1000 livebirths
Fraction of mortality
2000
2015
2019
Other
Measles
Diarrhoea
Congenital
Intrapartum−related
Preterm birth
AIDS
Injury
Malaria
Sepsis or meningitis
LRI
Mexico (Neonatal)
All−cause NMR
Neonatal other
Neonatal diarrhoea
Neonatal tetanus
Neonatal congenital
Neonatal sepsis or meningitis
Neonatal intrapartum
Neonatal preterm
Neonatal LRI
−0.02
0.00
0.02
0.04
0.06
Mortality rate per 1000 live births
Fraction of mortality
2000
2015
2019
2000 − 2015
2015 − 2019 (not on target)
Deficit to target
2015 − 2019 (on target)

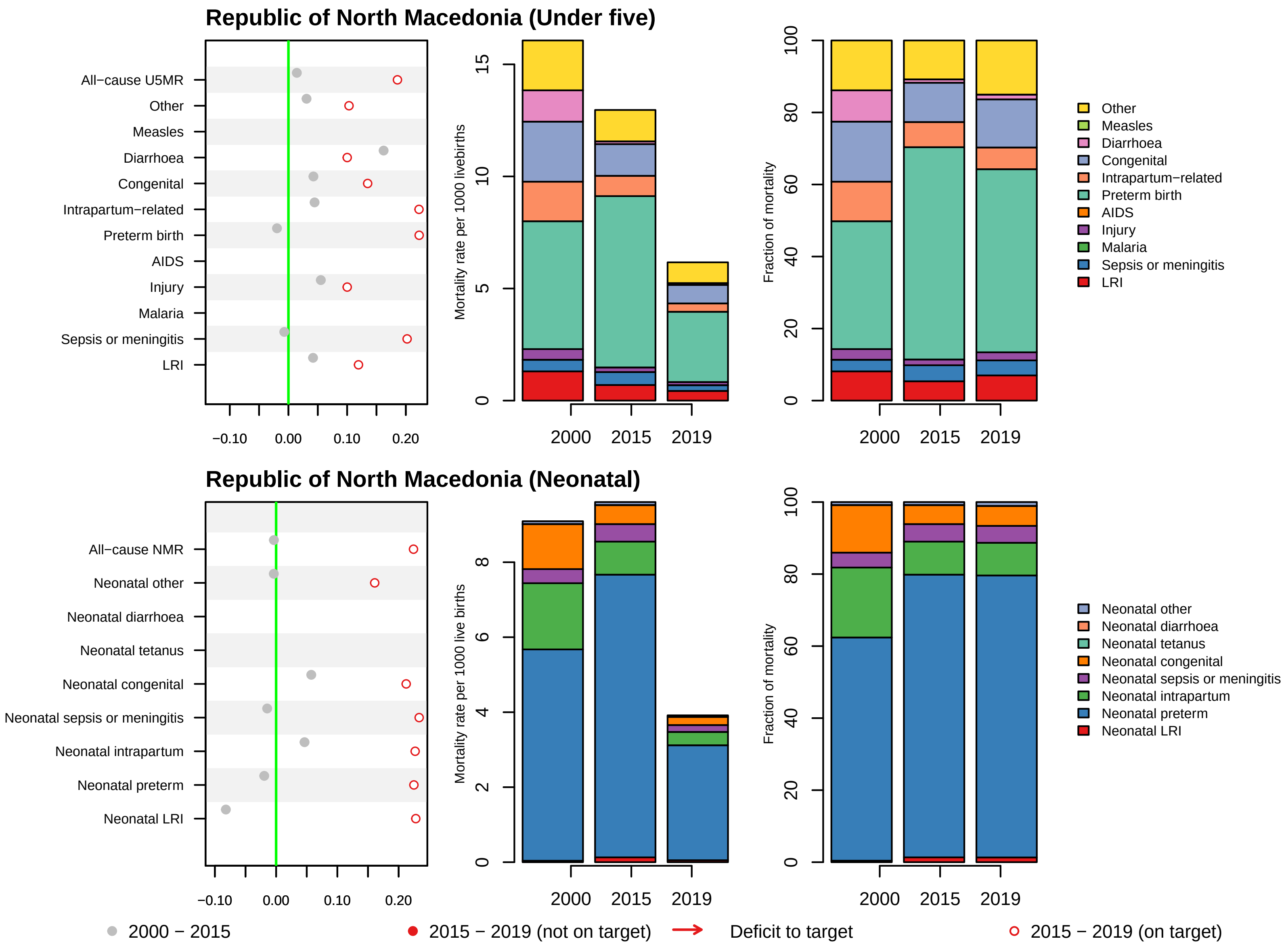
Republic of North Macedonia (Under five)
All−cause U5MR
Other
Measles
Diarrhoea
Congenital
Intrapartum−related
Preterm birth
AIDS
Injury
Malaria
Sepsis or meningitis
LRI
−0.10
0.00
0.10
0.20
Mortality rate per 1000 livebirths
0
5
10
15
2000
2015
2019
Fraction of mortality
0
20
40
60
80
100
Other
Measles
Diarrhoea
Congenital
Intrapartum−related
Preterm birth
AIDS
Injury
Malaria
Sepsis or meningitis
LRI
Republic of North Macedonia (Neonatal)
All−cause NMR
Neonatal other
Neonatal diarrhoea
Neonatal tetanus
Neonatal congenital
Neonatal sepsis or meningitis
Neonatal intrapartum
Neonatal preterm
Neonatal LRI
−0.10
0.00
0.10
0.20
Mortality rate per 1000 live births
0
2
4
6
8
2000
2015
2019
Fraction of mortality
0
20
40
60
80
100
Neonatal other
Neonatal diarrhoea
Neonatal tetanus
Neonatal congenital
Neonatal sepsis or meningitis
Neonatal intrapartum
Neonatal preterm
Neonatal LRI
2000 − 2015
2015 − 2019 (not on target)
Deficit to target
2015 − 2019 (on target)

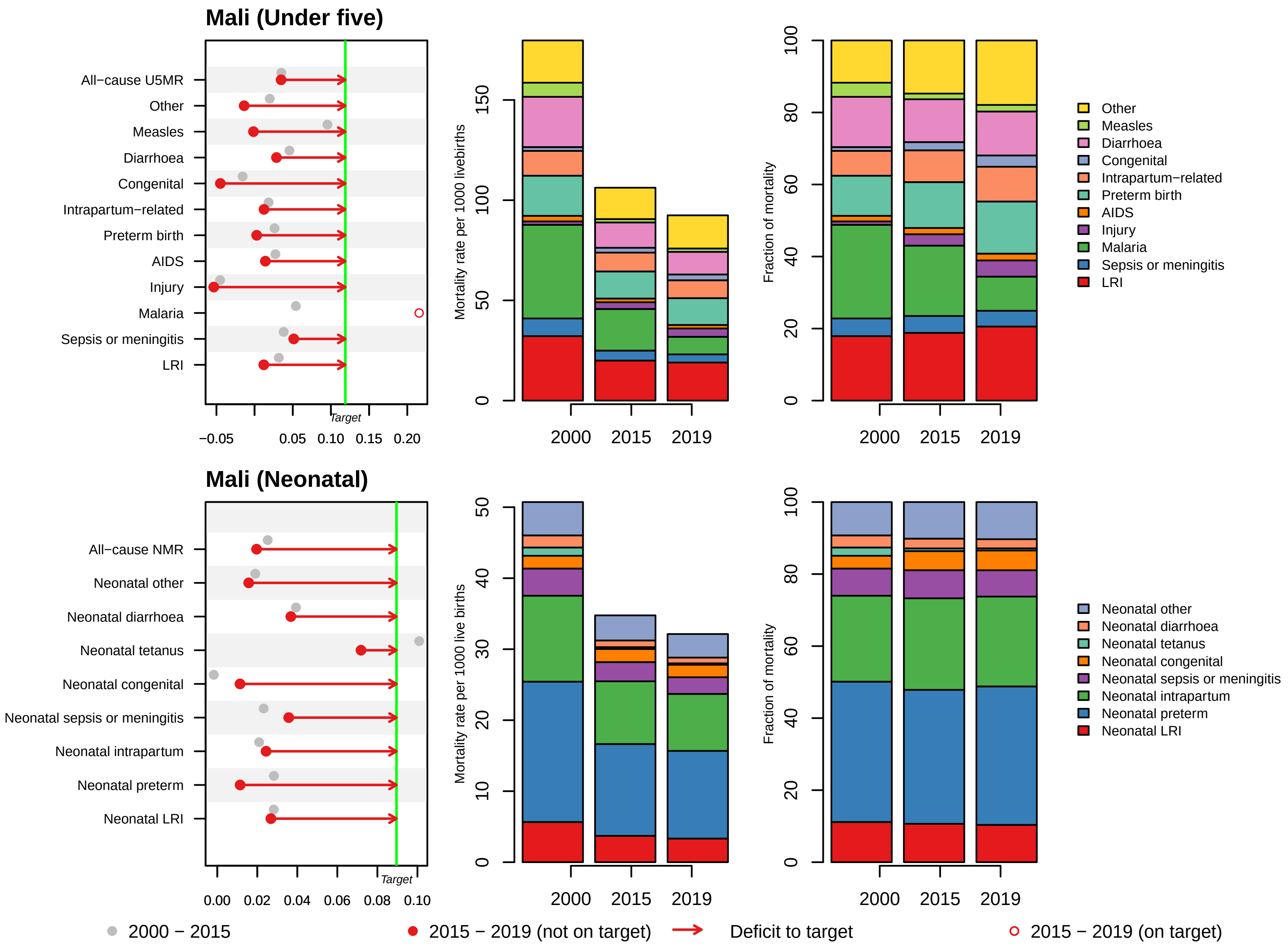
Mali (Under five)
All−cause U5MR
Other
Measles
Diarrhoea
Congenital
Intrapartum−related
Preterm birth
AIDS
Injury
Malaria
Sepsis or meningitis
LRI
Target
−0.05
0.05
0.10
0.15
0.20
Mortality rate per 1000 livebirths
0
50
100
150
2000
2015
2019
Fraction of mortality
0
20
40
60
80
100
Other
Measles
Diarrhoea
Congenital
Intrapartum−related
Preterm birth
AIDS
Injury
Malaria
Sepsis or meningitis
LRI
Mali (Neonatal)
All−cause NMR
Neonatal other
Neonatal diarrhoea
Neonatal tetanus
Neonatal congenital
Neonatal sepsis or meningitis
Neonatal intrapartum
Neonatal preterm
Neonatal LRI
Target
0.00
0.02
0.04
0.06
0.08
0.10
Mortality rate per 1000 live births
0
10
20
30
40
50
Neonatal other
Neonatal diarrhoea
Neonatal tetanus
Neonatal congenital
Neonatal sepsis or meningitis
Neonatal intrapartum
Neonatal preterm
Neonatal LRI
2000 − 2015
2015 − 2019 (not on target)
Deficit to target
2015 − 2019 (on target)

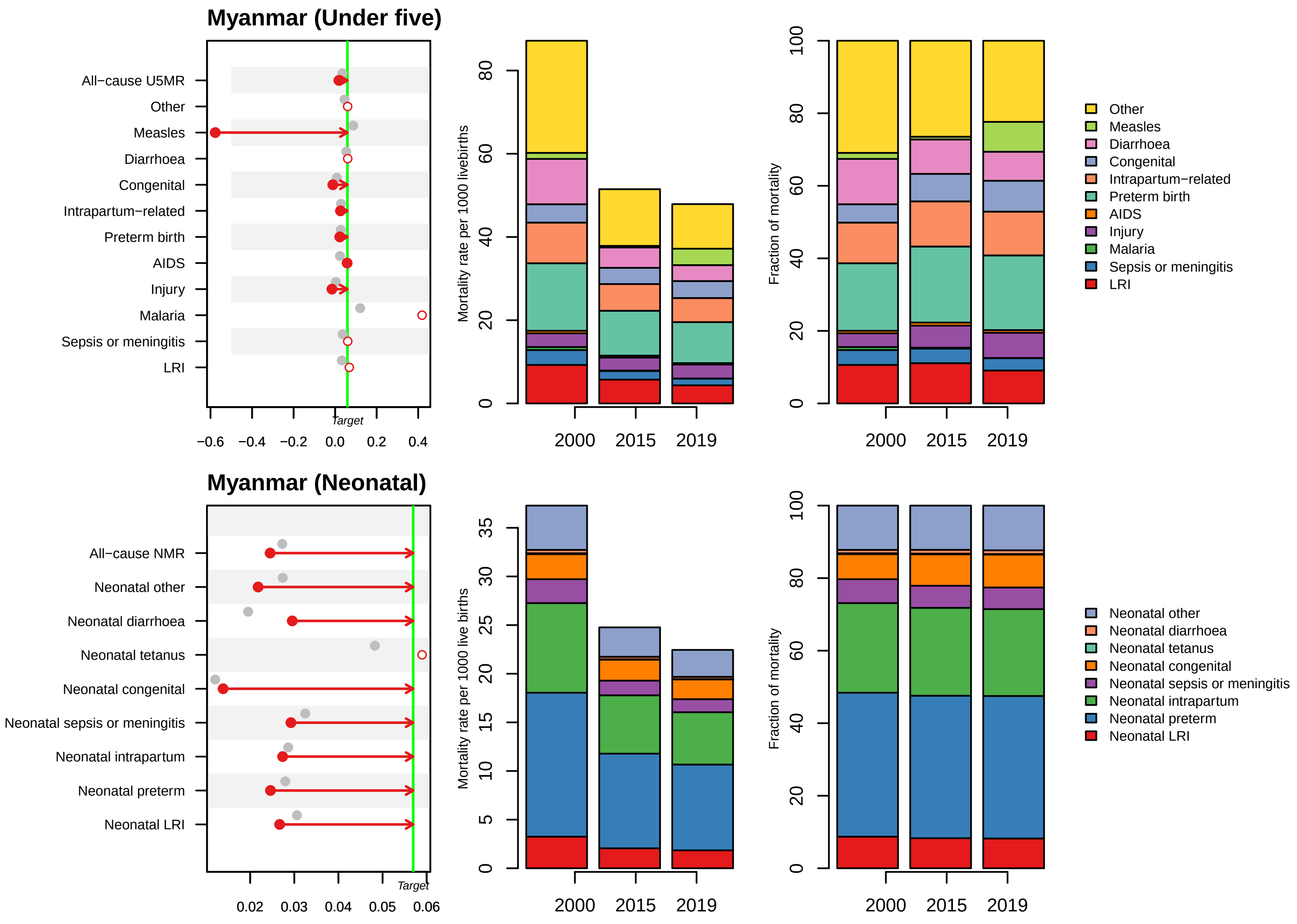

2000 − 2015 | 2015 − 2019 (not on target) | Deficit to target | 2015 − 2019 (on target)

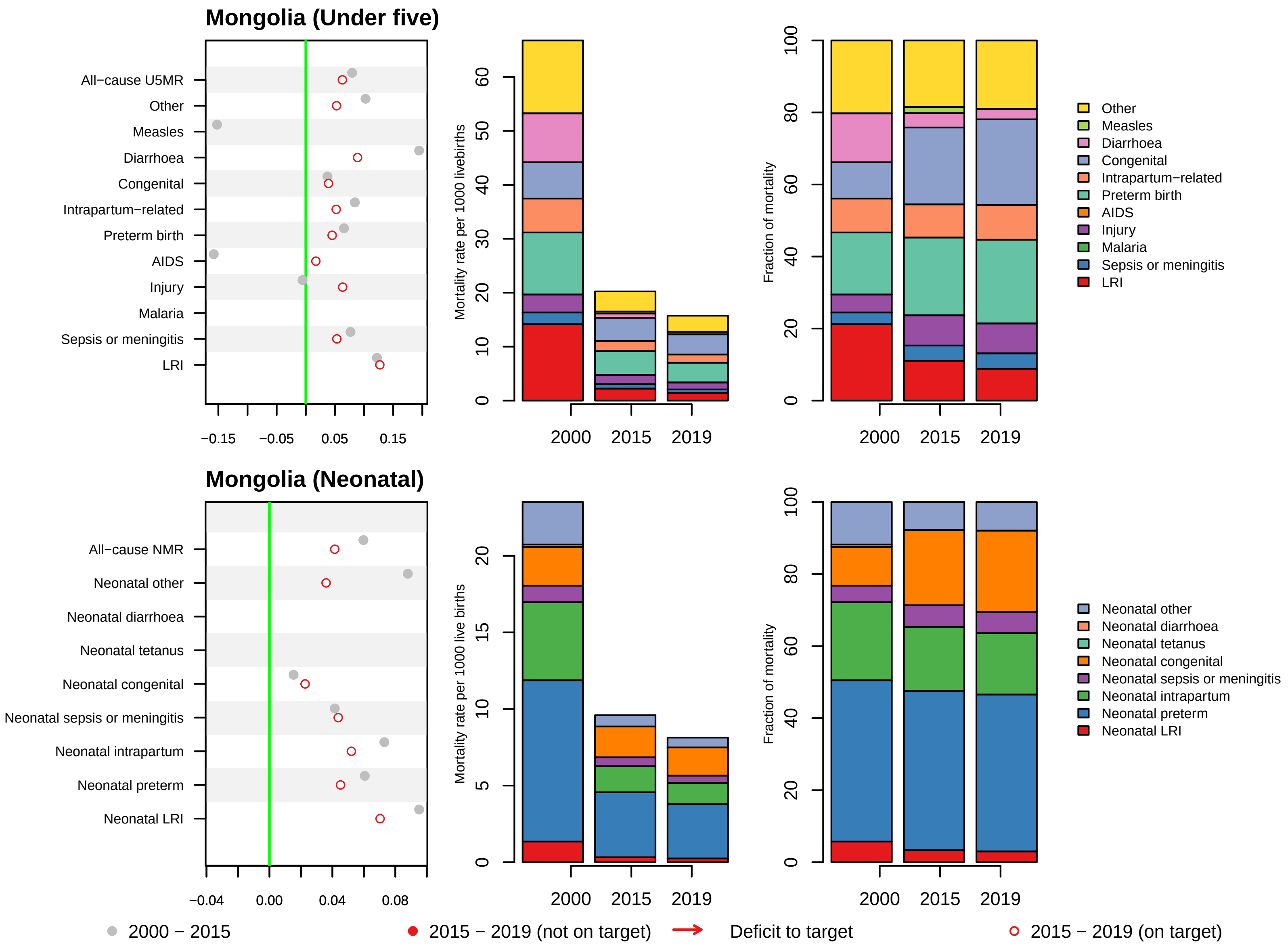

Mongolia (Under five)
All−cause U5MR
Other
Measles
Diarrhoea
Congenital
Intrapartum−related
Preterm birth
AIDS
Injury
Malaria
Sepsis or meningitis
LRI
−0.15
−0.05
0.05
0.15
Mortality rate per 1000 livebirths
2000
2015
2019
Fraction of mortality
Mongolia (Neonatal)
All−cause NMR
Neonatal other
Neonatal diarrhoea
Neonatal tetanus
Neonatal congenital
Neonatal sepsis or meningitis
Neonatal intrapartum
Neonatal preterm
Neonatal LRI
−0.04
0.00
0.04
0.08
Mortality rate per 1000 live births
2000 − 2015
2015 − 2019 (not on target)
Deficit to target
2015 − 2019 (on target)

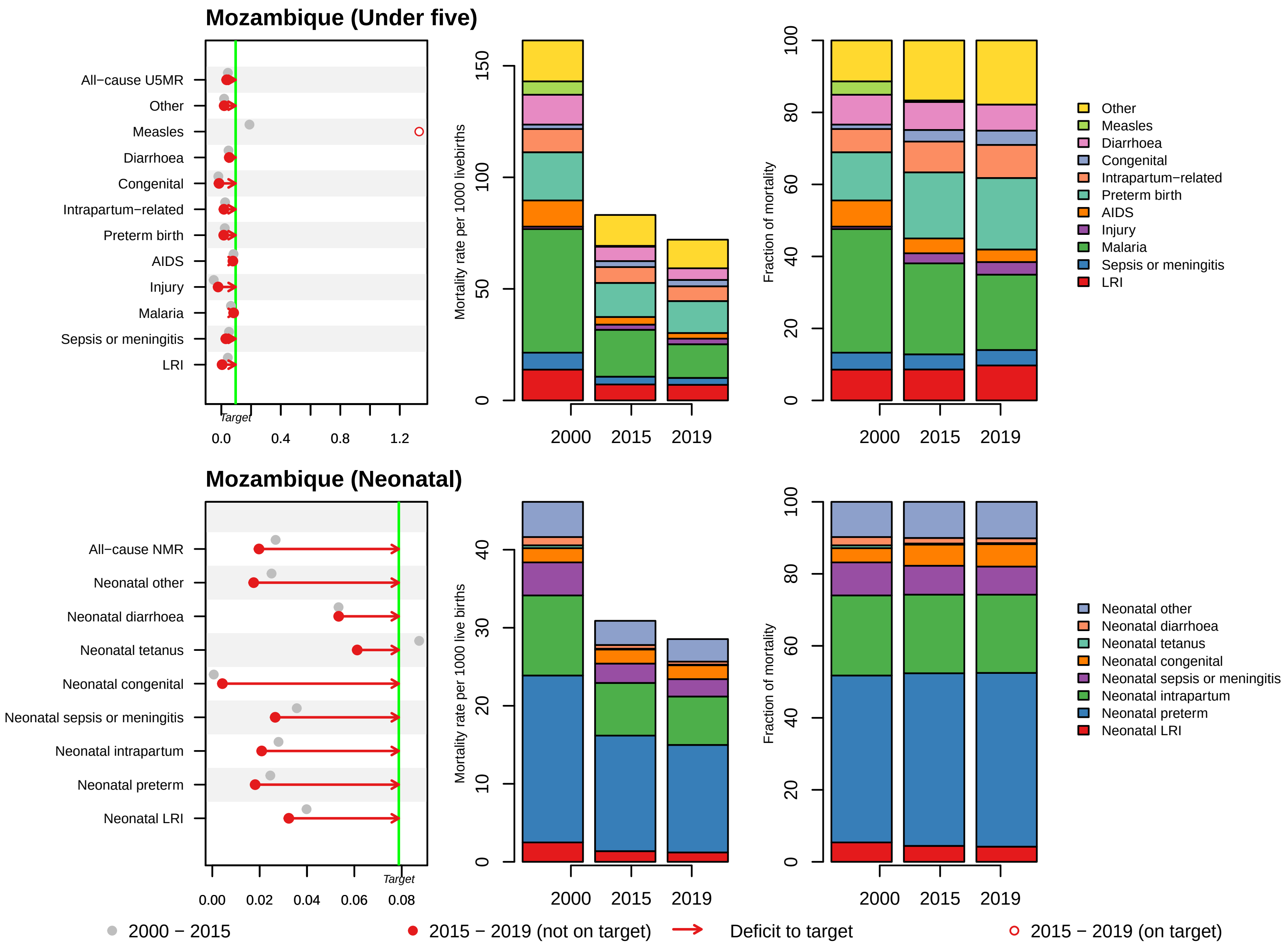
Mozambique (Under five)
All−cause U5MR
Other
Measles
Diarrhoea
Congenital
Intrapartum−related
Preterm birth
AIDS
Injury
Malaria
Sepsis or meningitis
LRI
Target
0.0
0.4
0.8
1.2
Mortality rate per 1000 livebirths
0
50
100
150
2000
2015
2019
Fraction of mortality
0
20
40
60
80
100
Other
Measles
Diarrhoea
Congenital
Intrapartum−related
Preterm birth
AIDS
Injury
Malaria
Sepsis or meningitis
LRI
Mozambique (Neonatal)
All−cause NMR
Neonatal other
Neonatal diarrhoea
Neonatal tetanus
Neonatal congenital
Neonatal sepsis or meningitis
Neonatal intrapartum
Neonatal preterm
Neonatal LRI
Target
0.00
0.02
0.04
0.06
0.08
Mortality rate per 1000 live births
0
10
20
30
40
Neonatal other
Neonatal diarrhoea
Neonatal tetanus
Neonatal congenital
Neonatal sepsis or meningitis
Neonatal intrapartum
Neonatal preterm
Neonatal LRI
2000 − 2015
2015 − 2019 (not on target)
Deficit to target
2015 − 2019 (on target)

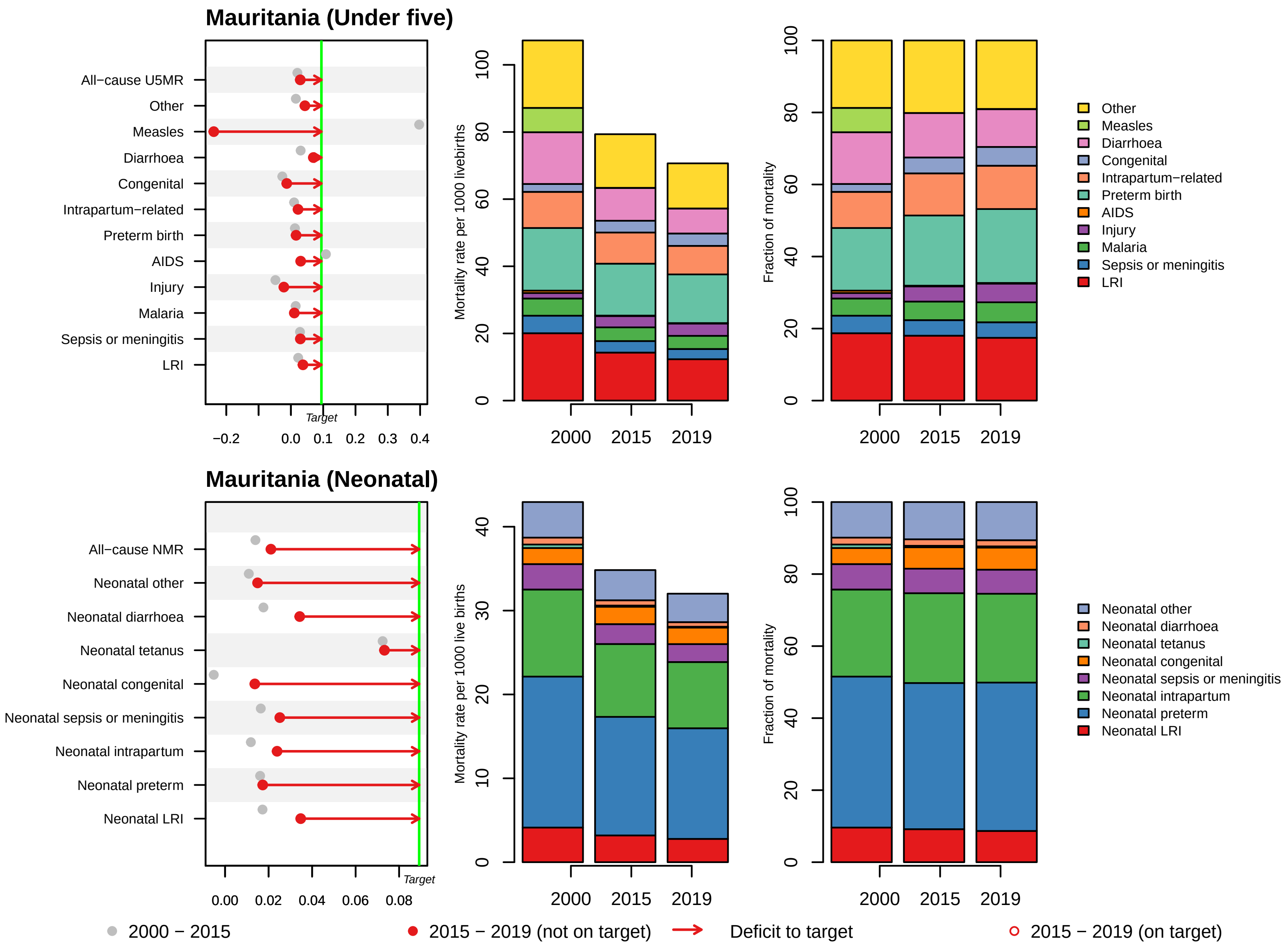
Mauritania (Under five)
All−cause U5MR
Other
Measles
Diarrhoea
Congenital
Intrapartum−related
Preterm birth
AIDS
Injury
Malaria
Sepsis or meningitis
LRI
Target
−0.2
0.0
0.1
0.2
0.3
0.4
Mortality rate per 1000 livebirths
Fraction of mortality
2000
2015
2019
Other
Measles
Diarrhoea
Congenital
Intrapartum−related
Preterm birth
AIDS
Injury
Malaria
Sepsis or meningitis
LRI
Mauritania (Neonatal)
All−cause NMR
Neonatal other
Neonatal diarrhoea
Neonatal tetanus
Neonatal congenital
Neonatal sepsis or meningitis
Neonatal intrapartum
Neonatal preterm
Neonatal LRI
0.00
0.02
0.04
0.06
0.08
Target
Mortality rate per 1000 live births
Fraction of mortality
2000
2015
2019
Neonatal other
Neonatal diarrhoea
Neonatal tetanus
Neonatal congenital
Neonatal sepsis or meningitis
Neonatal intrapartum
Neonatal preterm
Neonatal LRI
2000 − 2015
2015 − 2019 (not on target)
Deficit to target
2015 − 2019 (on target)

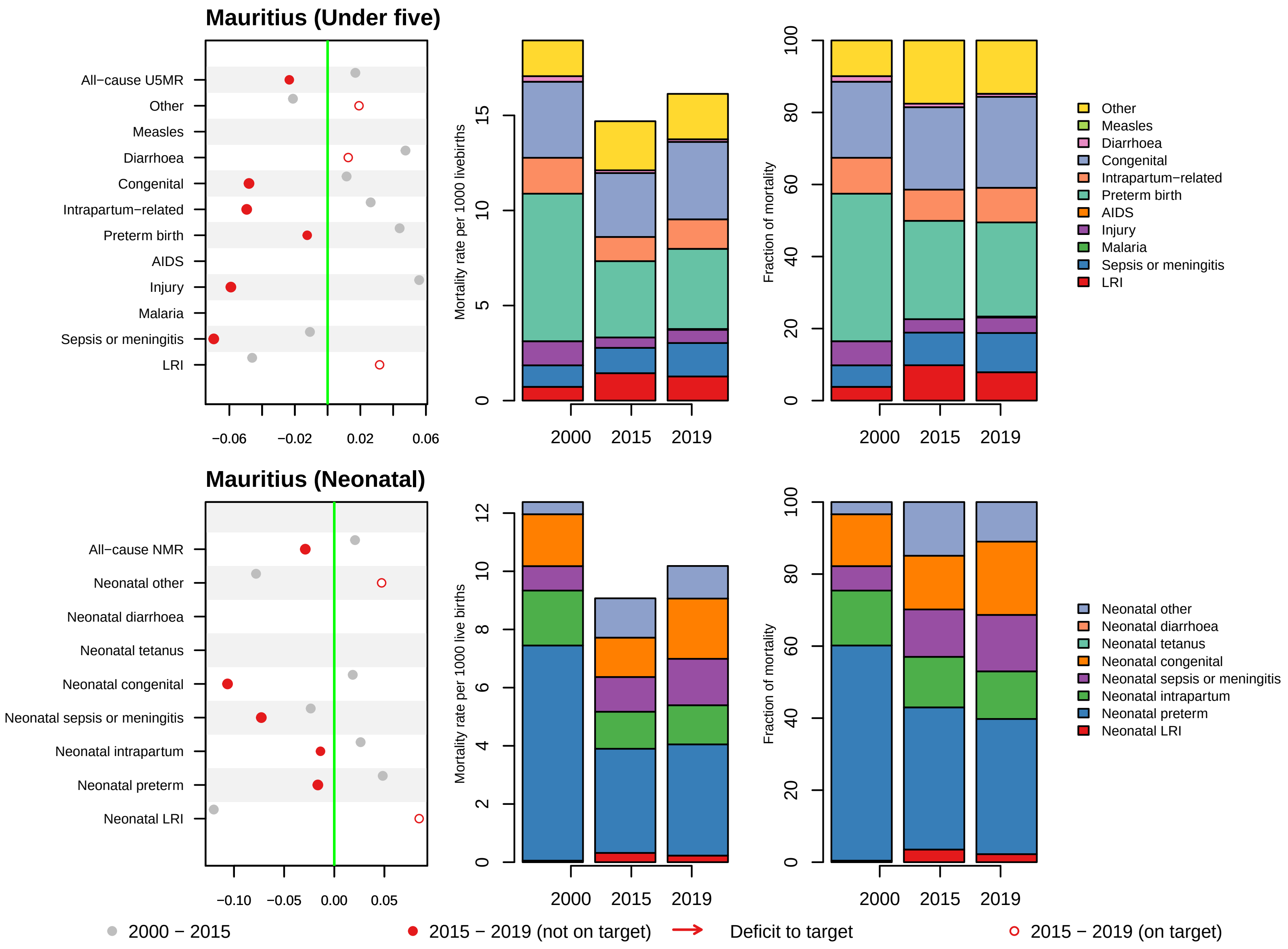

Mauritius (Under five)
All−cause U5MR
Other
Measles
Diarrhoea
Congenital
Intrapartum−related
Preterm birth
AIDS
Injury
Malaria
Sepsis or meningitis
LRI
−0.06
−0.02
0.02
0.06
Mortality rate per 1000 livebirths
Fraction of mortality
2000
2015
2019
Other
Measles
Diarrhoea
Congenital
Intrapartum−related
Preterm birth
AIDS
Injury
Malaria
Sepsis or meningitis
LRI
Mauritius (Neonatal)
All−cause NMR
Neonatal other
Neonatal diarrhoea
Neonatal tetanus
Neonatal congenital
Neonatal sepsis or meningitis
Neonatal intrapartum
Neonatal preterm
Neonatal LRI
−0.10
−0.05
0.00
0.05
Mortality rate per 1000 live births
Fraction of mortality
2000
2015
2019
Neonatal other
Neonatal diarrhoea
Neonatal tetanus
Neonatal congenital
Neonatal sepsis or meningitis
Neonatal intrapartum
Neonatal preterm
Neonatal LRI
2000 − 2015
2015 − 2019 (not on target)
Deficit to target
2015 − 2019 (on target)

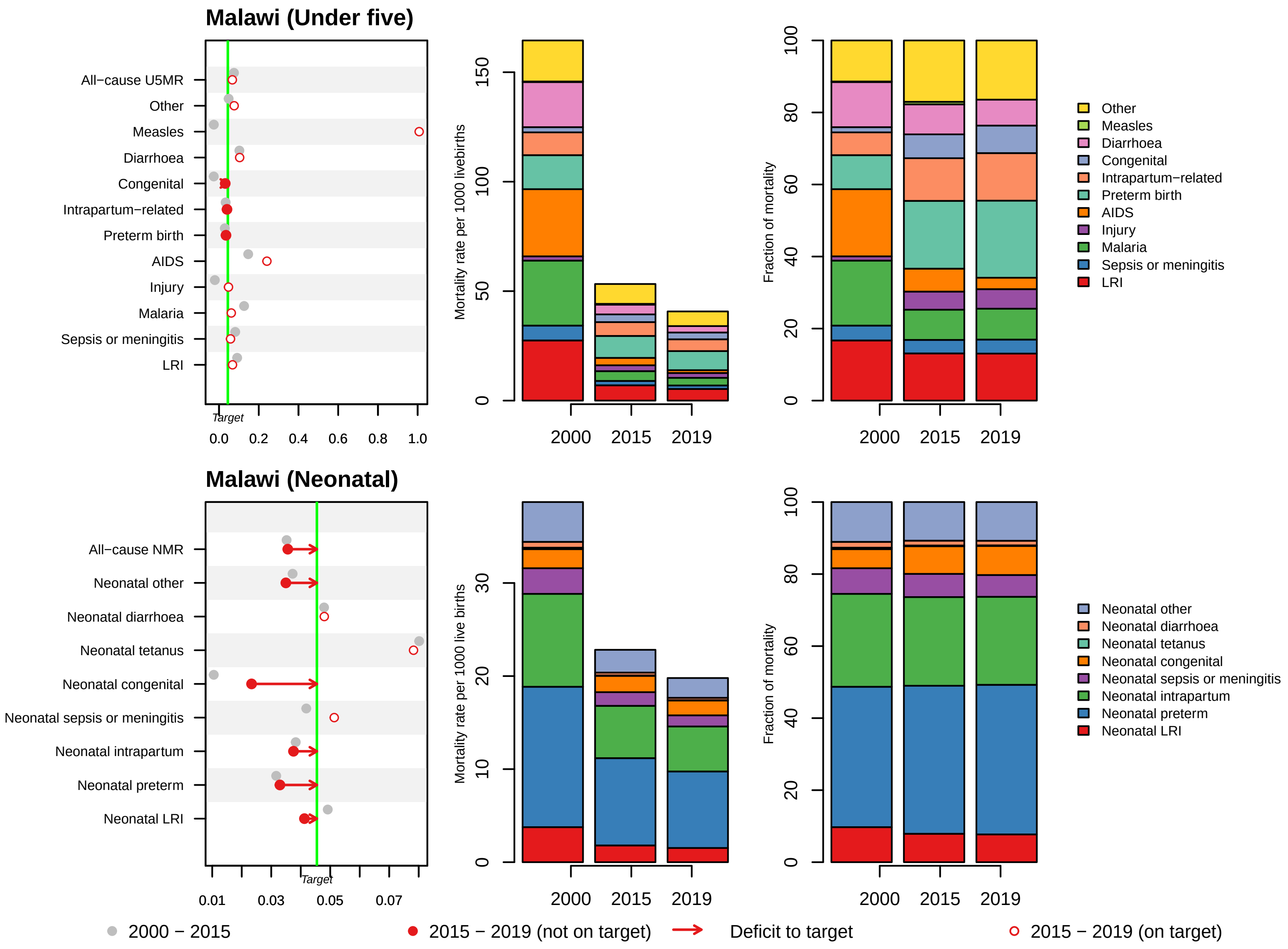

Malawi (Under five)
All−cause U5MR
Other
Measles
Diarrhoea
Congenital
Intrapartum−related
Preterm birth
AIDS
Injury
Malaria
Sepsis or meningitis
LRI
Target
0.0
0.2
0.4
0.6
0.8
1.0
Mortality rate per 1000 livebirths
Fraction of mortality
2000
2015
2019
Other
Measles
Diarrhoea
Congenital
Intrapartum−related
Preterm birth
AIDS
Injury
Malaria
Sepsis or meningitis
LRI
Malawi (Neonatal)
All−cause NMR
Neonatal other
Neonatal diarrhoea
Neonatal tetanus
Neonatal congenital
Neonatal sepsis or meningitis
Neonatal intrapartum
Neonatal preterm
Neonatal LRI
Target
0.01
0.03
0.05
0.07
Mortality rate per 1000 live births
Fraction of mortality
Neonatal other
Neonatal diarrhoea
Neonatal tetanus
Neonatal congenital
Neonatal sepsis or meningitis
Neonatal intrapartum
Neonatal preterm
Neonatal LRI
2000 − 2015
2015 − 2019 (not on target)
Deficit to target
2015 − 2019 (on target)

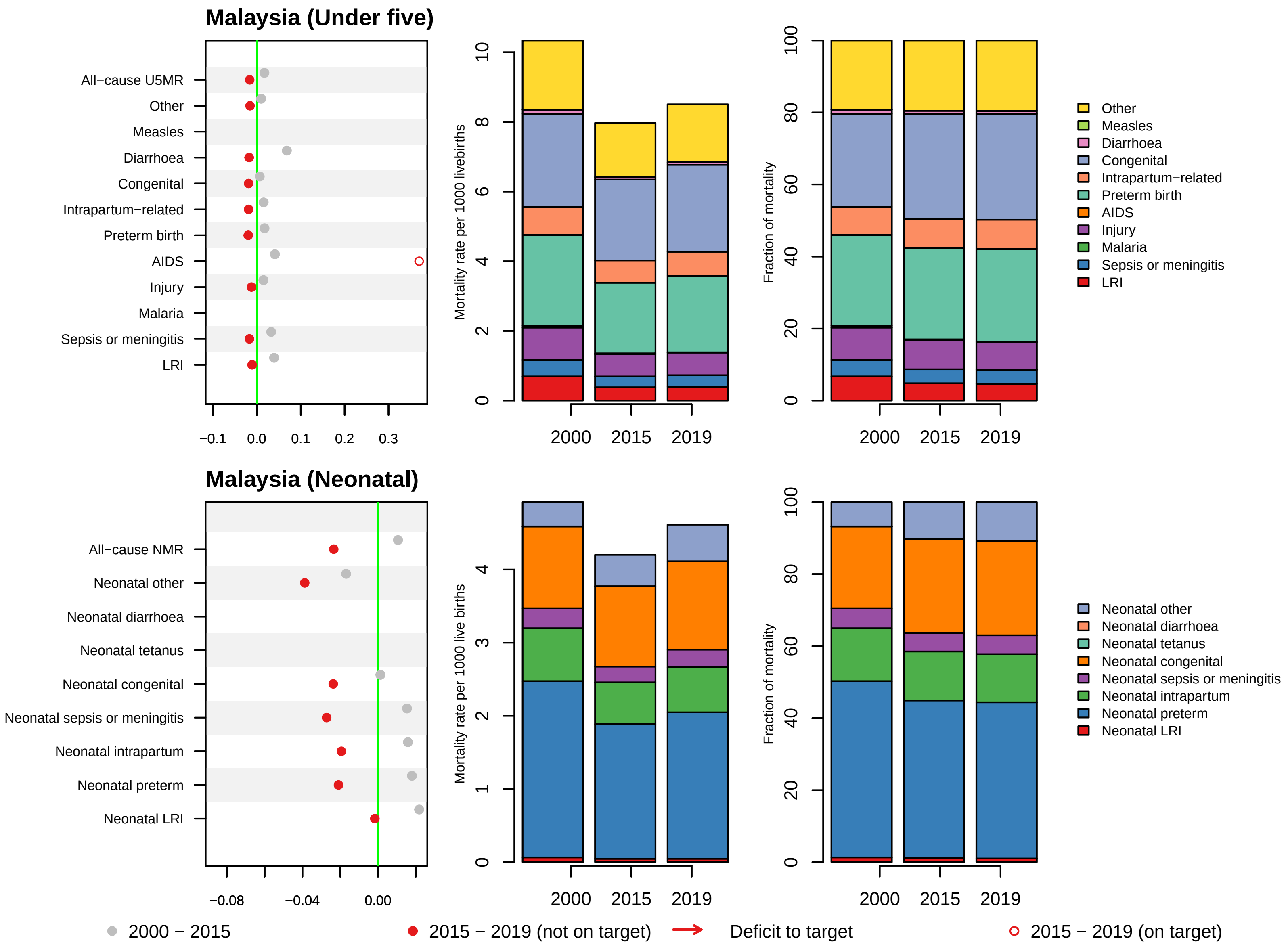
Malaysia (Under five)
All−cause U5MR
Other
Measles
Diarrhoea
Congenital
Intrapartum−related
Preterm birth
AIDS
Injury
Malaria
Sepsis or meningitis
LRI
−0.1
0.0
0.1
0.2
0.3
Mortality rate per 1000 livebirths
Fraction of mortality
2000
2015
2019
Other
Measles
Diarrhoea
Congenital
Intrapartum−related
Preterm birth
AIDS
Injury
Malaria
Sepsis or meningitis
LRI
Malaysia (Neonatal)
All−cause NMR
Neonatal other
Neonatal diarrhoea
Neonatal tetanus
Neonatal congenital
Neonatal sepsis or meningitis
Neonatal intrapartum
Neonatal preterm
Neonatal LRI
−0.08
−0.04
0.00
Mortality rate per 1000 live births
Fraction of mortality
Neonatal other
Neonatal diarrhoea
Neonatal tetanus
Neonatal congenital
Neonatal sepsis or meningitis
Neonatal intrapartum
Neonatal preterm
Neonatal LRI
2000 − 2015
2015 − 2019 (not on target)
Deficit to target
2015 − 2019 (on target)

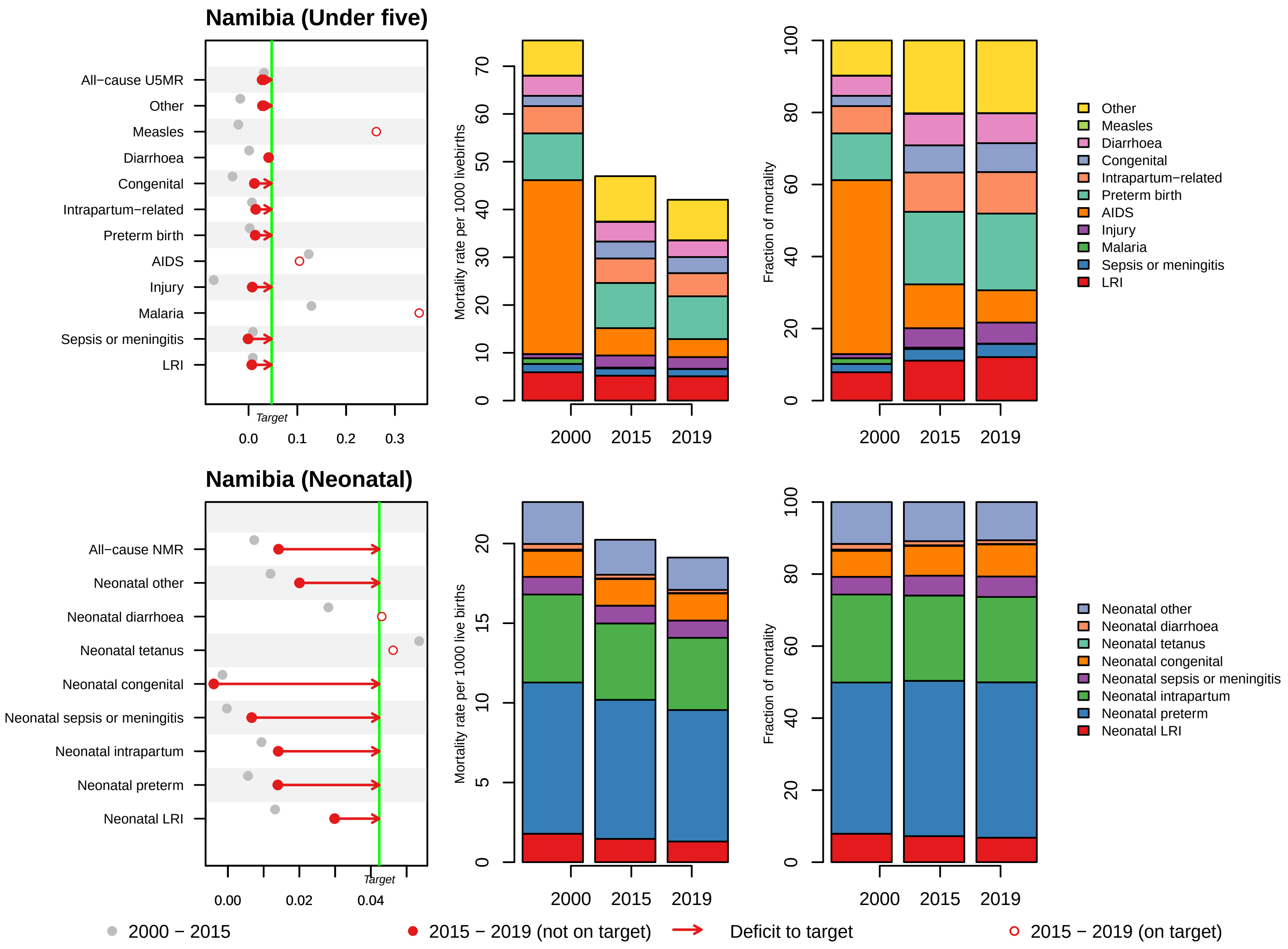

Namibia (Under five)
All−cause U5MR
Other
Measles
Diarrhoea
Congenital
Intrapartum−related
Preterm birth
AIDS
Injury
Malaria
Sepsis or meningitis
LRI
Target
0.0
0.1
0.2
0.3
Mortality rate per 1000 livebirths
Fraction of mortality
2000
2015
2019
Other
Measles
Diarrhoea
Congenital
Intrapartum−related
Preterm birth
AIDS
Injury
Malaria
Sepsis or meningitis
LRI
Namibia (Neonatal)
All−cause NMR
Neonatal other
Neonatal diarrhoea
Neonatal tetanus
Neonatal congenital
Neonatal sepsis or meningitis
Neonatal intrapartum
Neonatal preterm
Neonatal LRI
Target
0.00
0.02
0.04
Mortality rate per 1000 live births
Fraction of mortality
2000
2015
2019
Neonatal other
Neonatal diarrhoea
Neonatal tetanus
Neonatal congenital
Neonatal sepsis or meningitis
Neonatal intrapartum
Neonatal preterm
Neonatal LRI
2000 − 2015
2015 − 2019 (not on target)
Deficit to target
2015 − 2019 (on target)

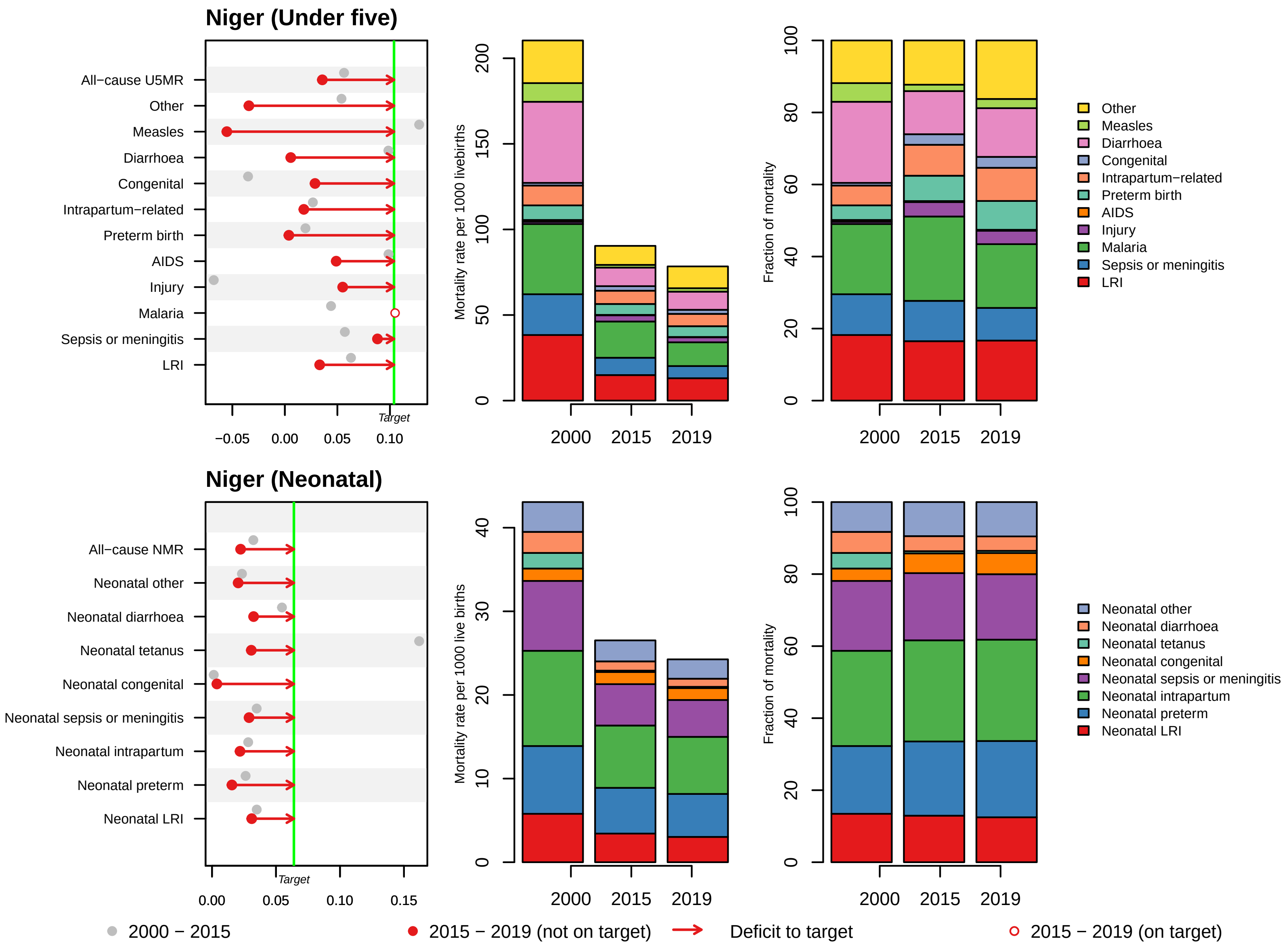

Niger (Under five)
All−cause U5MR
Other
Measles
Diarrhoea
Congenital
Intrapartum−related
Preterm birth
AIDS
Injury
Malaria
Sepsis or meningitis
LRI
Target
−0.05
0.00
0.05
0.10
Mortality rate per 1000 livebirths
0
50
100
150
200
2000
2015
2019
Fraction of mortality
0
20
40
60
80
100
Other
Measles
Diarrhoea
Congenital
Intrapartum−related
Preterm birth
AIDS
Injury
Malaria
Sepsis or meningitis
LRI
Niger (Neonatal)
All−cause NMR
Neonatal other
Neonatal diarrhoea
Neonatal tetanus
Neonatal congenital
Neonatal sepsis or meningitis
Neonatal intrapartum
Neonatal preterm
Neonatal LRI
Target
0.00
0.05
0.10
0.15
Mortality rate per 1000 live births
0
10
20
30
40
Neonatal other
Neonatal diarrhoea
Neonatal tetanus
Neonatal congenital
Neonatal sepsis or meningitis
Neonatal intrapartum
Neonatal preterm
Neonatal LRI
2000 − 2015
2015 − 2019 (not on target)
Deficit to target
2015 − 2019 (on target)

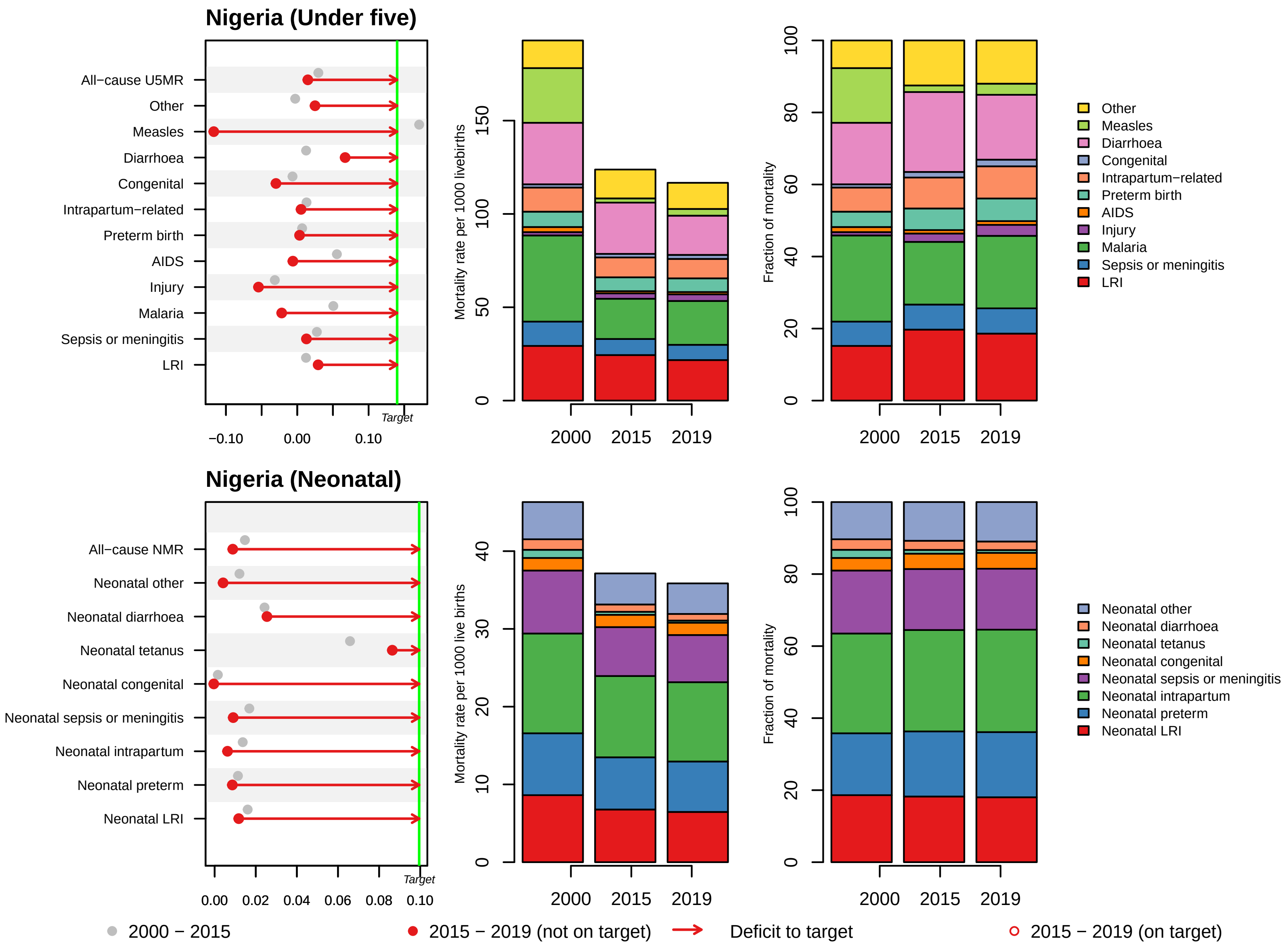
Nigeria (Under five)
All−cause U5MR
Other
Measles
Diarrhoea
Congenital
Intrapartum−related
Preterm birth
AIDS
Injury
Malaria
Sepsis or meningitis
LRI
Target
−0.10
0.00
0.10
Mortality rate per 1000 livebirths
0
50
100
150
2000
2015
2019
Fraction of mortality
0
20
40
60
80
100
Other
Measles
Diarrhoea
Congenital
Intrapartum−related
Preterm birth
AIDS
Injury
Malaria
Sepsis or meningitis
LRI
Nigeria (Neonatal)
All−cause NMR
Neonatal other
Neonatal diarrhoea
Neonatal tetanus
Neonatal congenital
Neonatal sepsis or meningitis
Neonatal intrapartum
Neonatal preterm
Neonatal LRI
Target
0.00
0.02
0.04
0.06
0.08
0.10
Mortality rate per 1000 live births
0
10
20
30
40
Neonatal other
Neonatal diarrhoea
Neonatal tetanus
Neonatal congenital
Neonatal sepsis or meningitis
Neonatal intrapartum
Neonatal preterm
Neonatal LRI
2000 − 2015
2015 − 2019 (not on target)
Deficit to target
2015 − 2019 (on target)

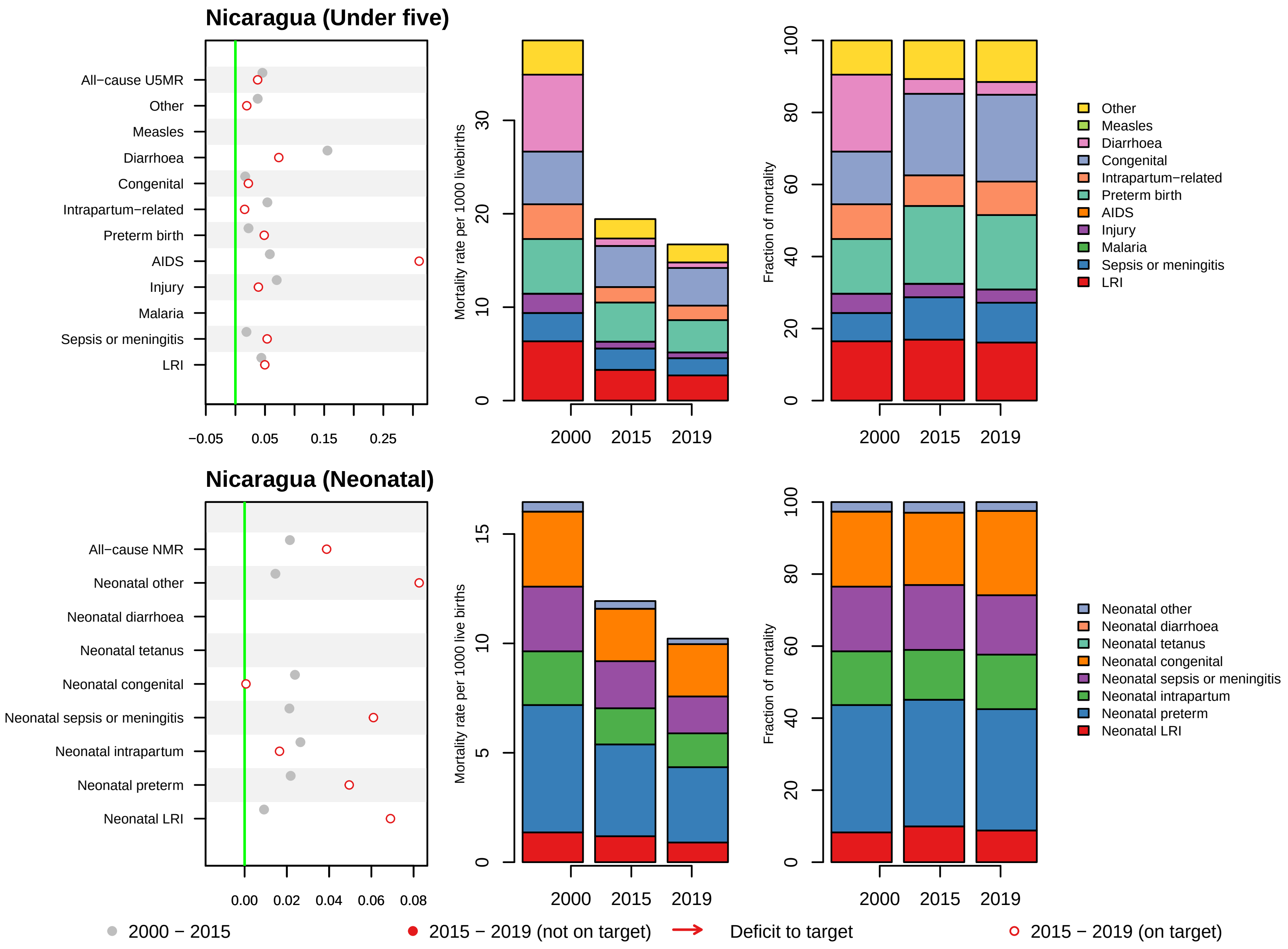

Nicaragua (Under five)
All−cause U5MR
Other
Measles
Diarrhoea
Congenital
Intrapartum−related
Preterm birth
AIDS
Injury
Malaria
Sepsis or meningitis
LRI
−0.05
0.05
0.15
0.25
Mortality rate per 1000 livebirths
Fraction of mortality
2000
2015
2019
Nicaragua (Neonatal)
All−cause NMR
Neonatal other
Neonatal diarrhoea
Neonatal tetanus
Neonatal congenital
Neonatal sepsis or meningitis
Neonatal intrapartum
Neonatal preterm
Neonatal LRI
0.00
0.02
0.04
0.06
0.08
Mortality rate per 1000 live births
2000 − 2015
2015 − 2019 (not on target)
Deficit to target
2015 − 2019 (on target)

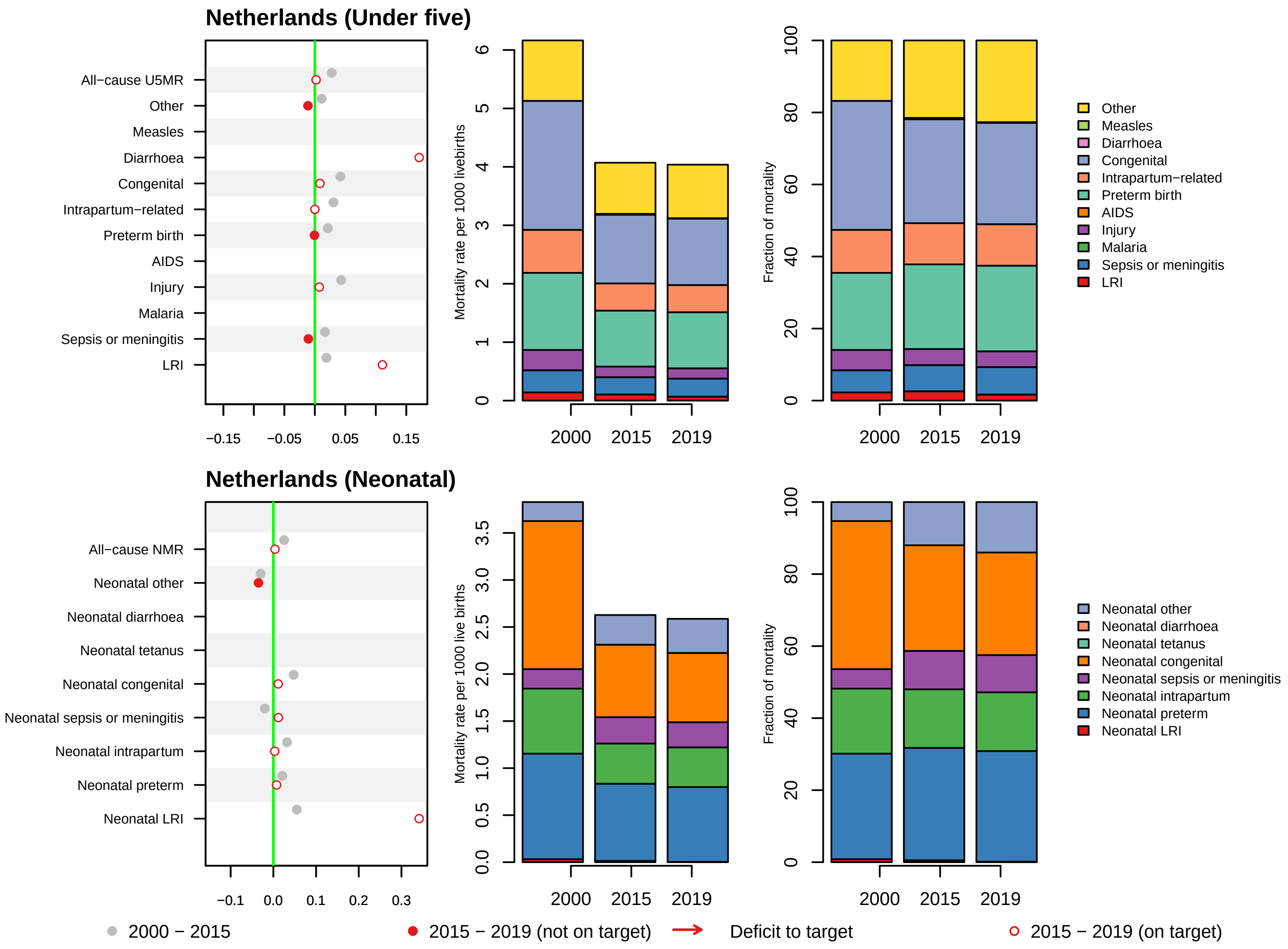

Netherlands (Under five)
All−cause U5MR
Other
Measles
Diarrhoea
Congenital
Intrapartum−related
Preterm birth
AIDS
Injury
Malaria
Sepsis or meningitis
LRI
−0.15
−0.05
0.05
0.15
Mortality rate per 1000 livebirths
2000
2015
2019
Fraction of mortality
Other
Measles
Diarrhoea
Congenital
Intrapartum−related
Preterm birth
AIDS
Injury
Malaria
Sepsis or meningitis
LRI
Netherlands (Neonatal)
All−cause NMR
Neonatal other
Neonatal diarrhoea
Neonatal tetanus
Neonatal congenital
Neonatal sepsis or meningitis
Neonatal intrapartum
Neonatal preterm
Neonatal LRI
−0.1
0.0
0.1
0.2
0.3
Mortality rate per 1000 live births
Fraction of mortality
Neonatal other
Neonatal diarrhoea
Neonatal tetanus
Neonatal congenital
Neonatal sepsis or meningitis
Neonatal intrapartum
Neonatal preterm
Neonatal LRI
2000 − 2015
2015 − 2019 (not on target)
Deficit to target
2015 − 2019 (on target)

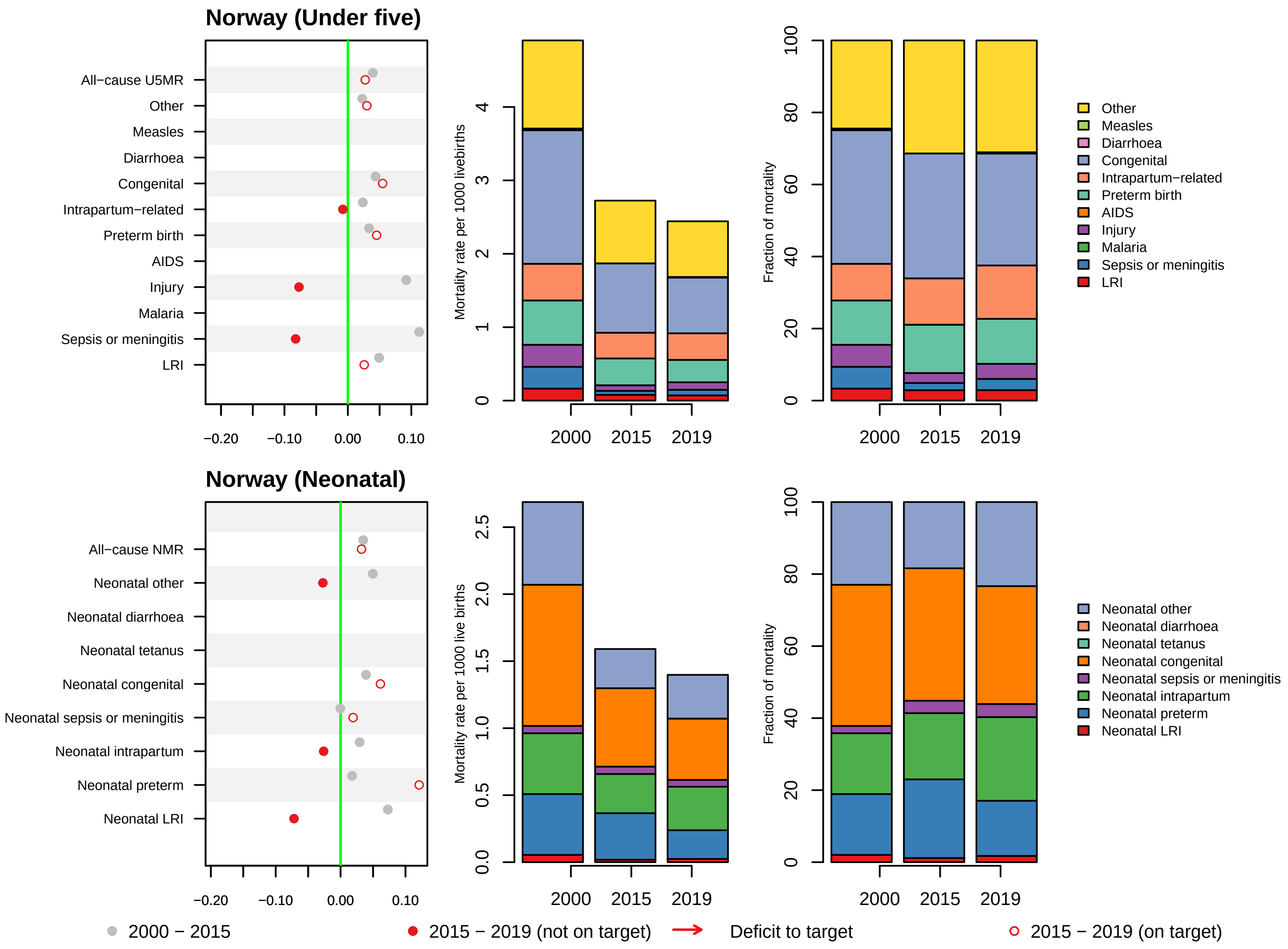

Norway (Under five)
All−cause U5MR
Other
Measles
Diarrhoea
Congenital
Intrapartum−related
Preterm birth
AIDS
Injury
Malaria
Sepsis or meningitis
LRI
−0.20
−0.10
0.00
0.10
Mortality rate per 1000 livebirths
2000
2015
2019
Fraction of mortality
Norway (Neonatal)
All−cause NMR
Neonatal other
Neonatal diarrhoea
Neonatal tetanus
Neonatal congenital
Neonatal sepsis or meningitis
Neonatal intrapartum
Neonatal preterm
Neonatal LRI
Mortality rate per 1000 live births
2000 − 2015
2015 − 2019 (not on target)
Deficit to target
2015 − 2019 (on target)

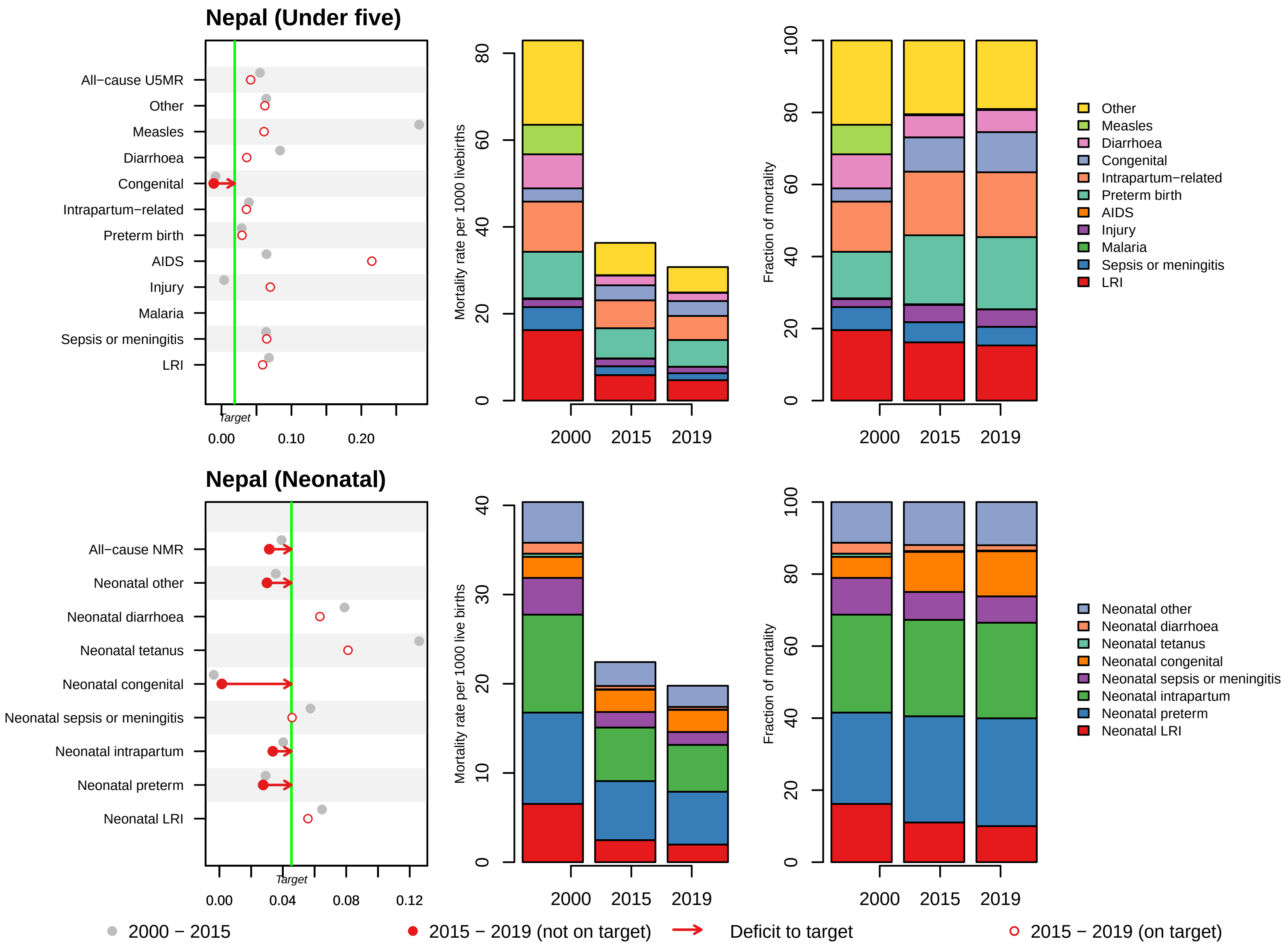

Nepal (Under five)
All−cause U5MR
Other
Measles
Diarrhoea
Congenital
Intrapartum−related
Preterm birth
AIDS
Injury
Malaria
Sepsis or meningitis
LRI
Target
0.00
0.10
0.20
Mortality rate per 1000 livebirths
0
20
40
60
80
2000
2015
2019
Fraction of mortality
0
20
40
60
80
100
2000
2015
2019
Other
Measles
Diarrhoea
Congenital
Intrapartum−related
Preterm birth
AIDS
Injury
Malaria
Sepsis or meningitis
LRI
Nepal (Neonatal)
All−cause NMR
Neonatal other
Neonatal diarrhoea
Neonatal tetanus
Neonatal congenital
Neonatal sepsis or meningitis
Neonatal intrapartum
Neonatal preterm
Neonatal LRI
Target
0.00
0.04
0.08
0.12
Mortality rate per 1000 live births
0
10
20
30
40
2000
2015
2019
Fraction of mortality
0
20
40
60
80
100
2000
2015
2019
Neonatal other
Neonatal diarrhoea
Neonatal tetanus
Neonatal congenital
Neonatal sepsis or meningitis
Neonatal intrapartum
Neonatal preterm
Neonatal LRI
2000 − 2015
2015 − 2019 (not on target)
Deficit to target
2015 − 2019 (on target)

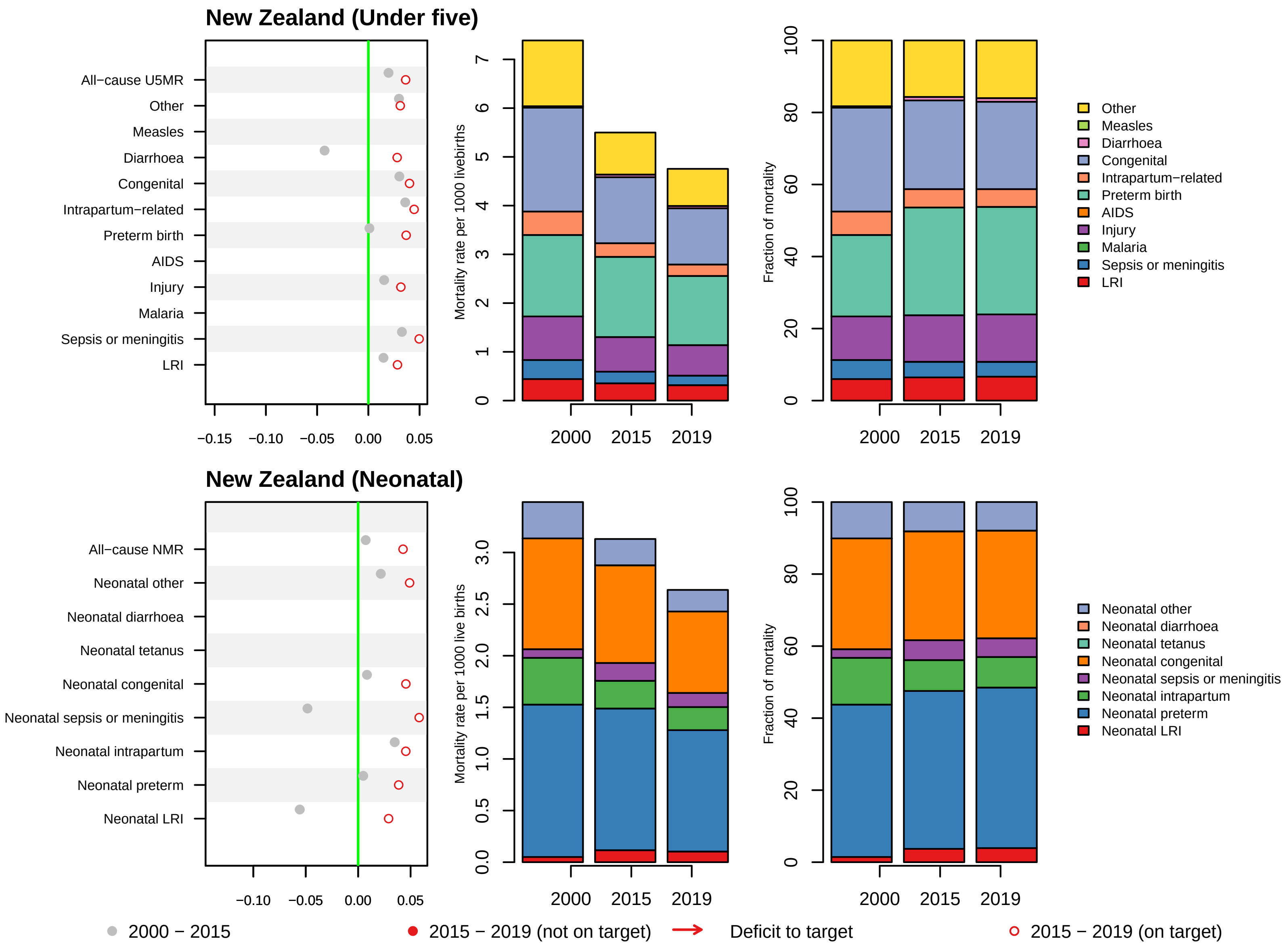
New Zealand (Under five)
All−cause U5MR
Other
Measles
Diarrhoea
Congenital
Intrapartum−related
Preterm birth
AIDS
Injury
Malaria
Sepsis or meningitis
LRI
−0.15
−0.10
−0.05
0.00
0.05
Mortality rate per 1000 livebirths
2000
2015
2019
Fraction of mortality
Other
Measles
Diarrhoea
Congenital
Intrapartum−related
Preterm birth
AIDS
Injury
Malaria
Sepsis or meningitis
LRI
New Zealand (Neonatal)
All−cause NMR
Neonatal other
Neonatal diarrhoea
Neonatal tetanus
Neonatal congenital
Neonatal sepsis or meningitis
Neonatal intrapartum
Neonatal preterm
Neonatal LRI
Mortality rate per 1000 live births
Fraction of mortality
Neonatal other
Neonatal diarrhoea
Neonatal tetanus
Neonatal congenital
Neonatal sepsis or meningitis
Neonatal intrapartum
Neonatal preterm
Neonatal LRI
2000 − 2015
2015 − 2019 (not on target)
Deficit to target
2015 − 2019 (on target)

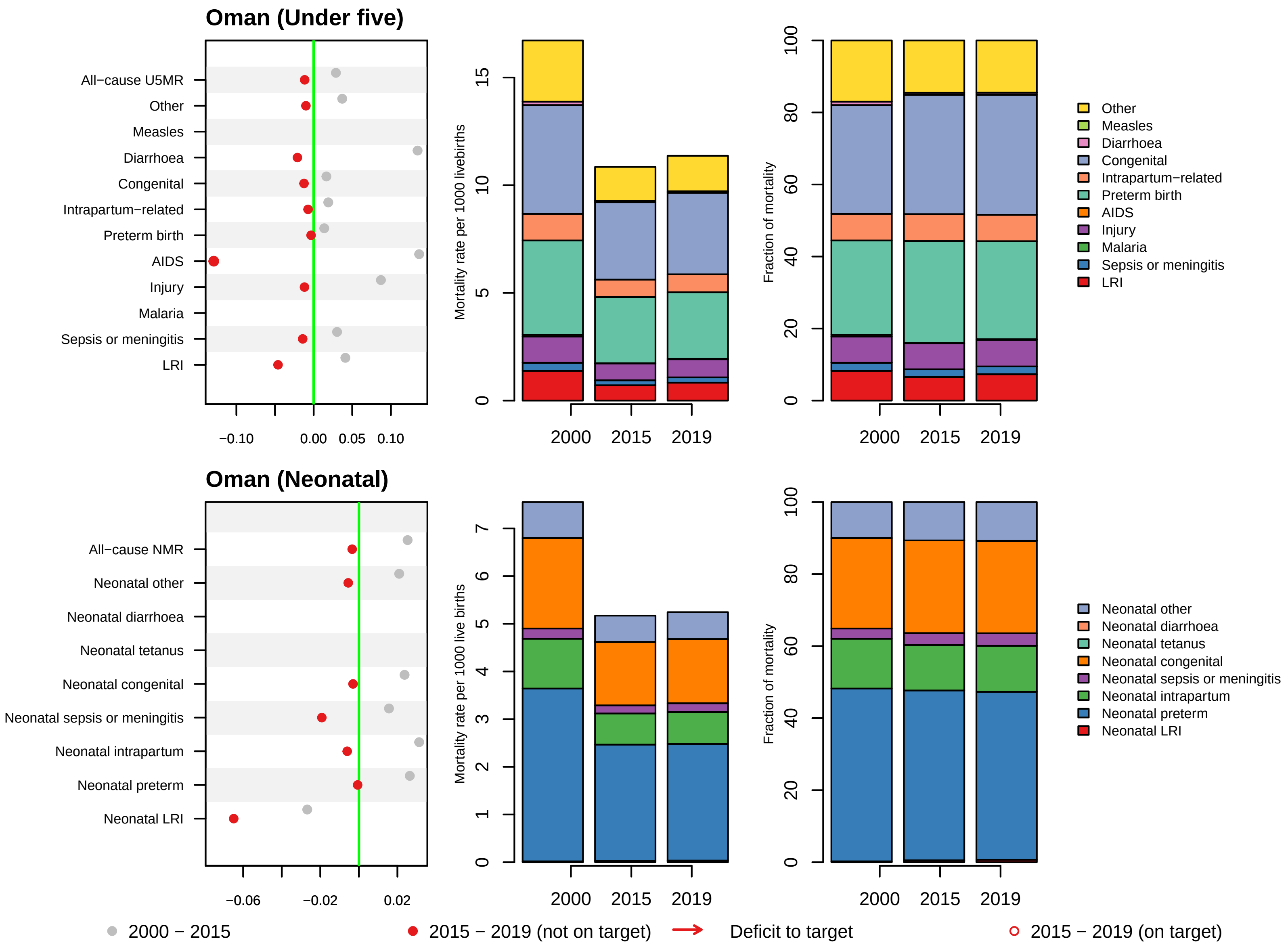

Oman (Under five)
All−cause U5MR
Other
Measles
Diarrhoea
Congenital
Intrapartum−related
Preterm birth
AIDS
Injury
Malaria
Sepsis or meningitis
LRI
−0.10
0.00
0.05
0.10
Mortality rate per 1000 livebirths
Fraction of mortality
2000
2015
2019
Other
Measles
Diarrhoea
Congenital
Intrapartum−related
Preterm birth
AIDS
Injury
Malaria
Sepsis or meningitis
LRI
Oman (Neonatal)
All−cause NMR
Neonatal other
Neonatal diarrhoea
Neonatal tetanus
Neonatal congenital
Neonatal sepsis or meningitis
Neonatal intrapartum
Neonatal preterm
Neonatal LRI
−0.06
−0.02
0.02
Mortality rate per 1000 live births
Fraction of mortality
Neonatal other
Neonatal diarrhoea
Neonatal tetanus
Neonatal congenital
Neonatal sepsis or meningitis
Neonatal intrapartum
Neonatal preterm
Neonatal LRI
2000 − 2015
2015 − 2019 (not on target)
Deficit to target
2015 − 2019 (on target)

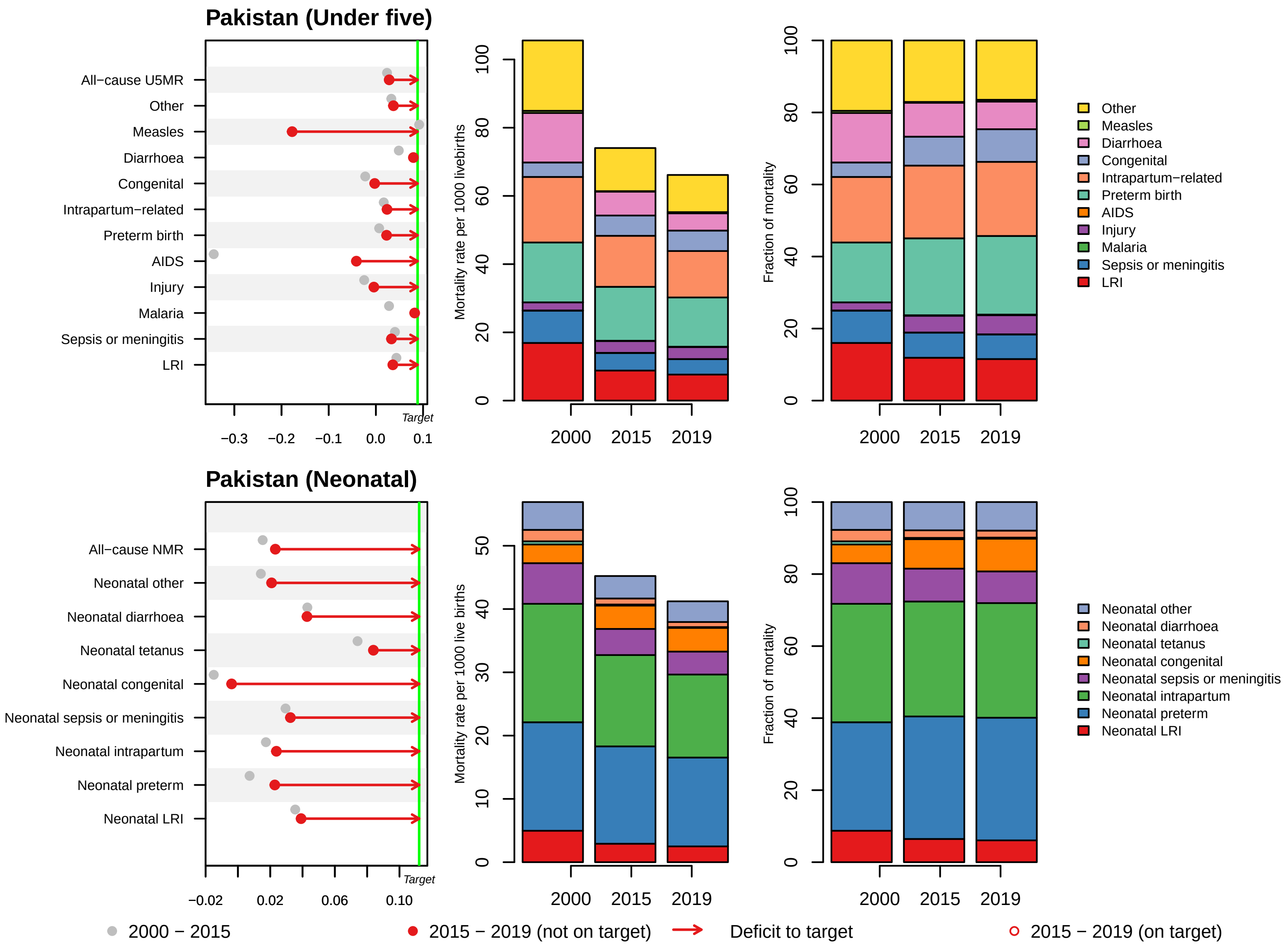
Pakistan (Under five)
All−cause U5MR
Other
Measles
Diarrhoea
Congenital
Intrapartum−related
Preterm birth
AIDS
Injury
Malaria
Sepsis or meningitis
LRI
Target
−0.3
−0.2
−0.1
0.0
0.1
Mortality rate per 1000 livebirths
0
20
40
60
80
100
2000
2015
2019
Fraction of mortality
Other
Measles
Diarrhoea
Congenital
Intrapartum−related
Preterm birth
AIDS
Injury
Malaria
Sepsis or meningitis
LRI
Pakistan (Neonatal)
All−cause NMR
Neonatal other
Neonatal diarrhoea
Neonatal tetanus
Neonatal congenital
Neonatal sepsis or meningitis
Neonatal intrapartum
Neonatal preterm
Neonatal LRI
−0.02
0.02
0.06
0.10
Mortality rate per 1000 live births
10
30
50
Neonatal other
Neonatal diarrhoea
Neonatal tetanus
Neonatal congenital
Neonatal sepsis or meningitis
Neonatal intrapartum
Neonatal preterm
Neonatal LRI
2000 − 2015
2015 − 2019 (not on target)
Deficit to target
2015 − 2019 (on target)

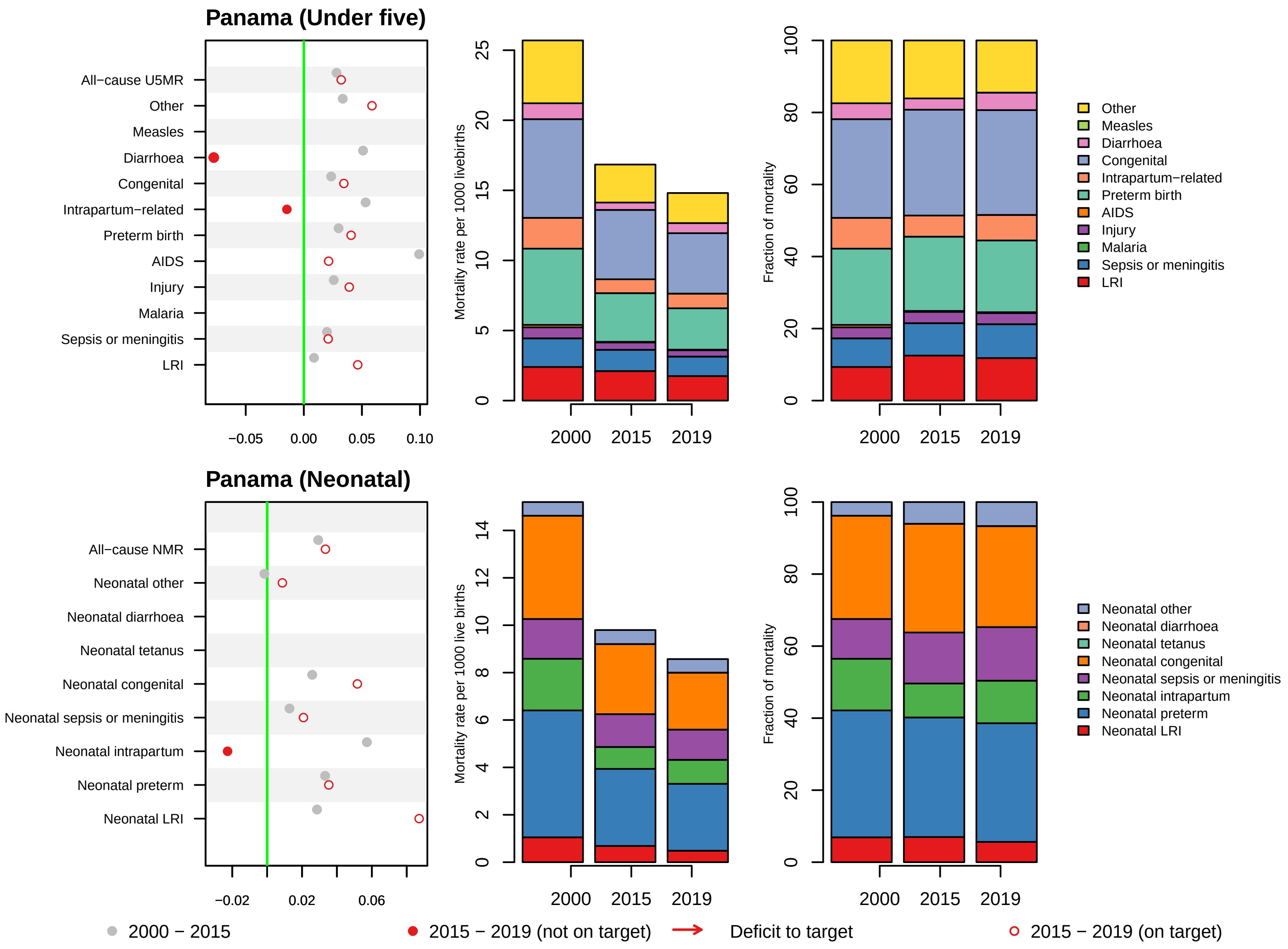

Panama (Under five)
All−cause U5MR
Other
Measles
Diarrhoea
Congenital
Intrapartum−related
Preterm birth
AIDS
Injury
Malaria
Sepsis or meningitis
LRI
−0.05
0.00
0.05
0.10
Mortality rate per 1000 livebirths
0
5
10
15
20
25
2000
2015
2019
Fraction of mortality
0
20
40
60
80
100
Other
Measles
Diarrhoea
Congenital
Intrapartum−related
Preterm birth
AIDS
Injury
Malaria
Sepsis or meningitis
LRI
Panama (Neonatal)
All−cause NMR
Neonatal other
Neonatal diarrhoea
Neonatal tetanus
Neonatal congenital
Neonatal sepsis or meningitis
Neonatal intrapartum
Neonatal preterm
Neonatal LRI
−0.02
0.02
0.06
Mortality rate per 1000 live births
0
2
4
6
8
10
12
14
Neonatal other
Neonatal diarrhoea
Neonatal tetanus
Neonatal congenital
Neonatal sepsis or meningitis
Neonatal intrapartum
Neonatal preterm
Neonatal LRI
2000 − 2015
2015 − 2019 (not on target)
Deficit to target
2015 − 2019 (on target)

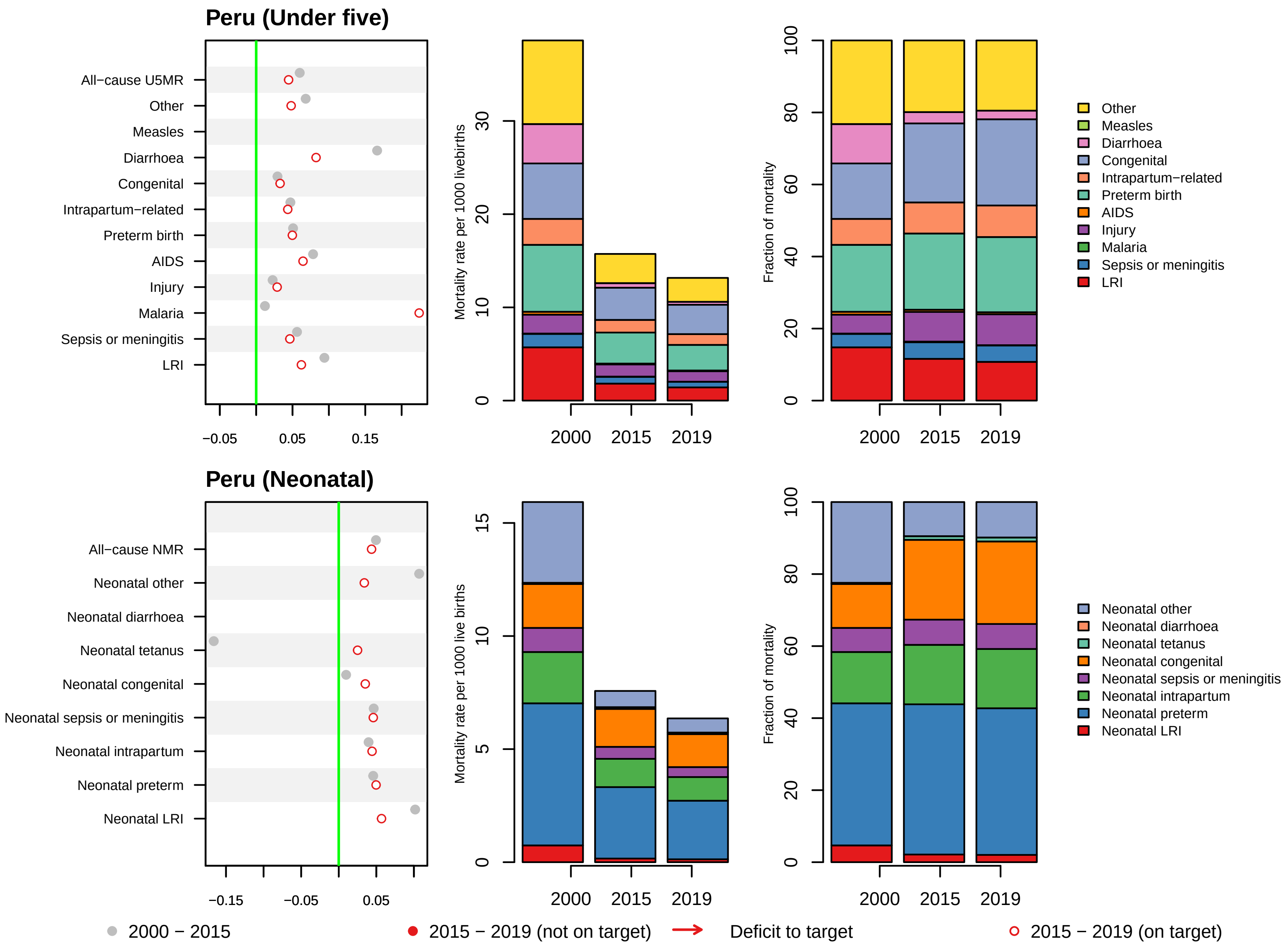

Peru (Under five)
All−cause U5MR
Other
Measles
Diarrhoea
Congenital
Intrapartum−related
Preterm birth
AIDS
Injury
Malaria
Sepsis or meningitis
LRI
−0.05
0.05
0.15
Mortality rate per 1000 livebirths
Fraction of mortality
2000
2015
2019
Peru (Neonatal)
All−cause NMR
Neonatal other
Neonatal diarrhoea
Neonatal tetanus
Neonatal congenital
Neonatal sepsis or meningitis
Neonatal intrapartum
Neonatal preterm
Neonatal LRI
−0.15
−0.05
0.05
Mortality rate per 1000 live births
2000 − 2015
2015 − 2019 (not on target)
Deficit to target
2015 − 2019 (on target)

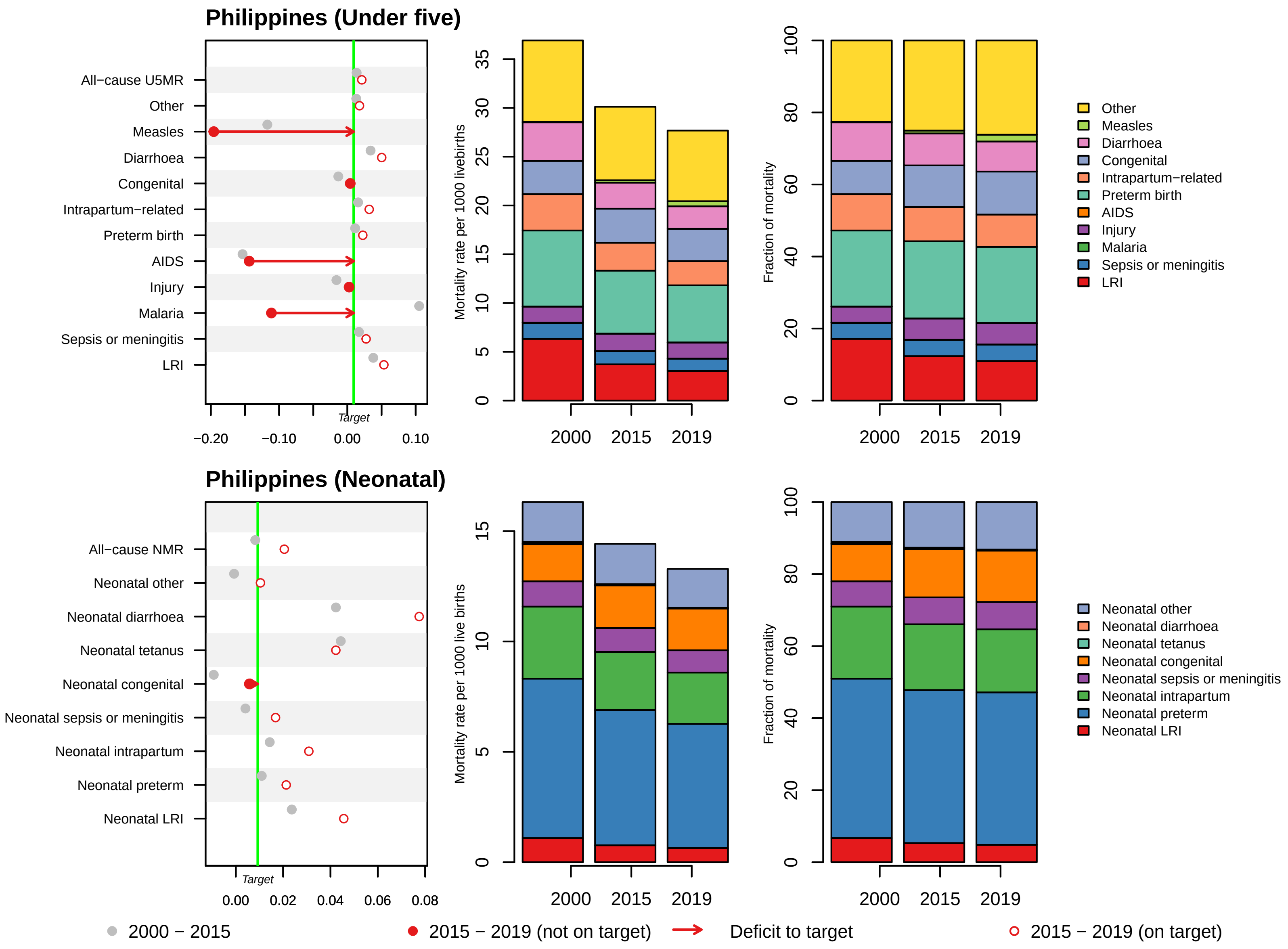

Philippines (Under five)
All−cause U5MR
Other
Measles
Diarrhoea
Congenital
Intrapartum−related
Preterm birth
AIDS
Injury
Malaria
Sepsis or meningitis
LRI
Target
−0.20
−0.10
0.00
0.10
Mortality rate per 1000 livebirths
Fraction of mortality
2000
2015
2019
Philippines (Neonatal)
All−cause NMR
Neonatal other
Neonatal diarrhoea
Neonatal tetanus
Neonatal congenital
Neonatal sepsis or meningitis
Neonatal intrapartum
Neonatal preterm
Neonatal LRI
Mortality rate per 1000 live births
Neonatal LRI
2000 − 2015
2015 − 2019 (not on target)
Deficit to target
2015 − 2019 (on target)

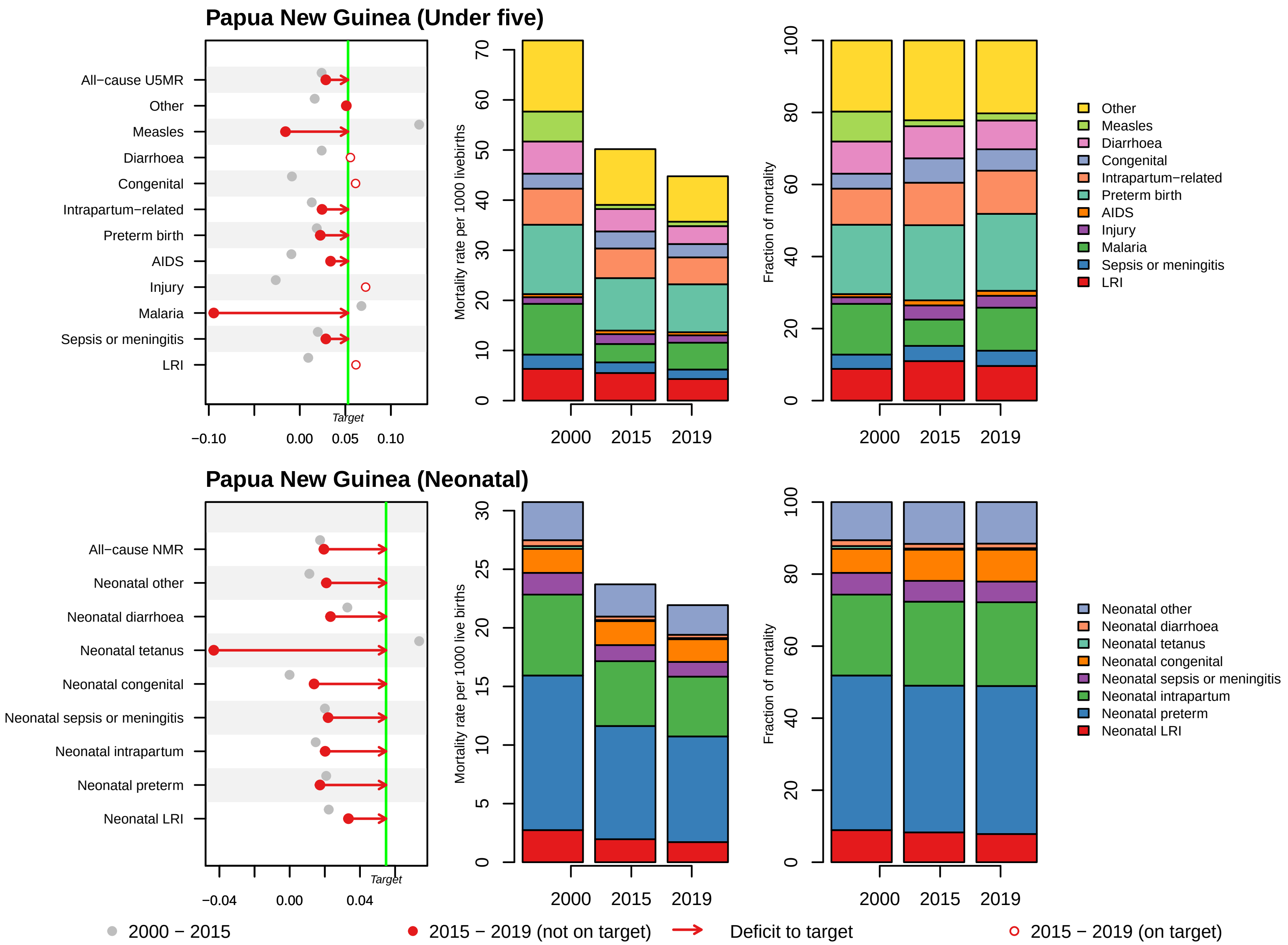
Papua New Guinea (Under five)
All−cause U5MR
Other
Measles
Diarrhoea
Congenital
Intrapartum−related
Preterm birth
AIDS
Injury
Malaria
Sepsis or meningitis
LRI
Target
−0.10
0.00
0.05
0.10
Mortality rate per 1000 livebirths
Fraction of mortality
2000
2015
2019
Other
Measles
Diarrhoea
Congenital
Intrapartum−related
Preterm birth
AIDS
Injury
Malaria
Sepsis or meningitis
LRI
Papua New Guinea (Neonatal)
All−cause NMR
Neonatal other
Neonatal diarrhoea
Neonatal tetanus
Neonatal congenital
Neonatal sepsis or meningitis
Neonatal intrapartum
Neonatal preterm
Neonatal LRI
Target
−0.04
0.00
0.04
Mortality rate per 1000 live births
Fraction of mortality
Neonatal other
Neonatal diarrhoea
Neonatal tetanus
Neonatal congenital
Neonatal sepsis or meningitis
Neonatal intrapartum
Neonatal preterm
Neonatal LRI
2000 − 2015
2015 − 2019 (not on target)
Deficit to target
2015 − 2019 (on target)

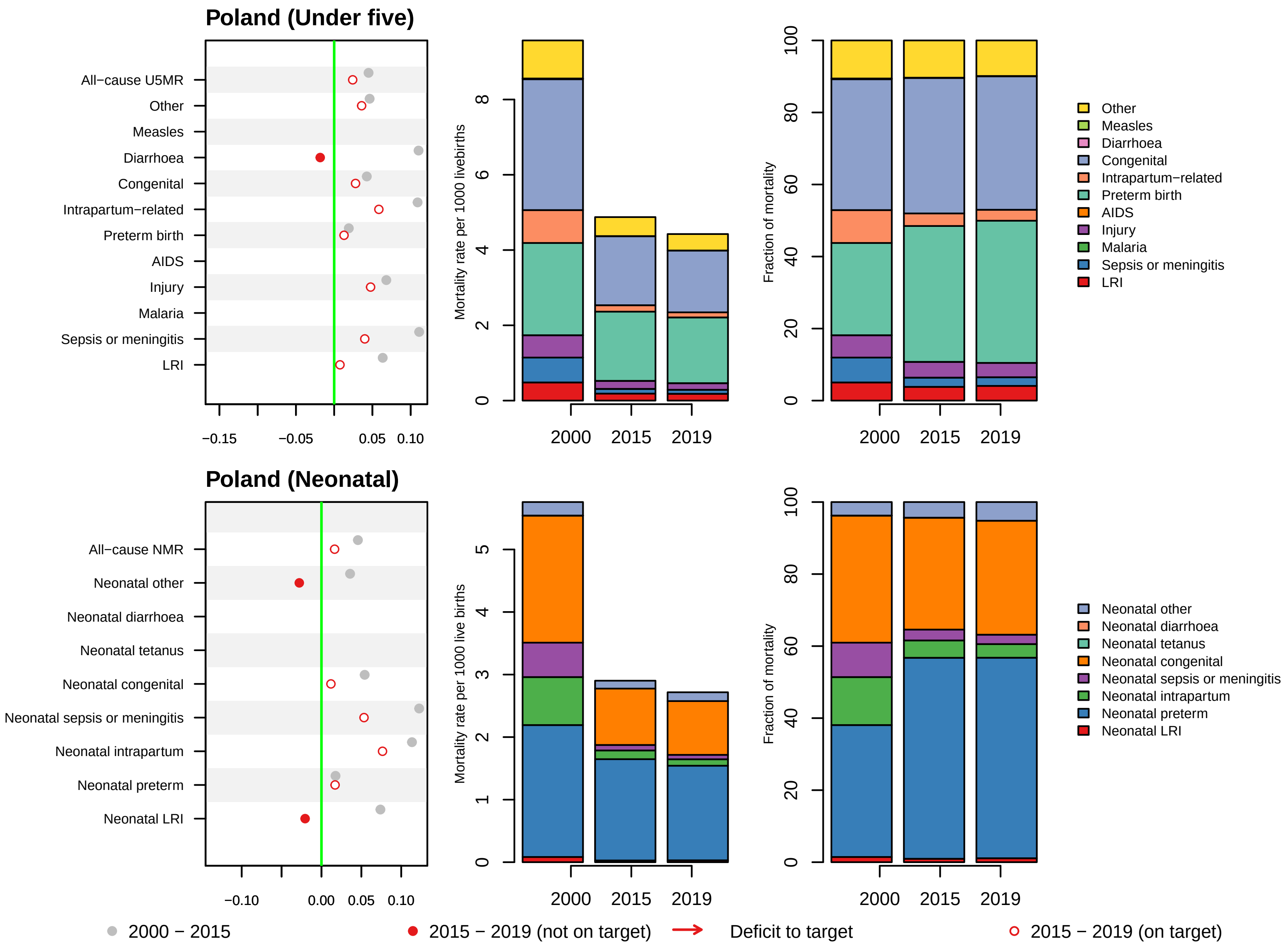

Poland (Under five)
All−cause U5MR
Other
Measles
Diarrhoea
Congenital
Intrapartum−related
Preterm birth
AIDS
Injury
Malaria
Sepsis or meningitis
LRI
−0.15
−0.05
0.05
0.10
Mortality rate per 1000 livebirths
Fraction of mortality
2000
2015
2019
Other
Measles
Diarrhoea
Congenital
Intrapartum−related
Preterm birth
AIDS
Injury
Malaria
Sepsis or meningitis
LRI
Poland (Neonatal)
All−cause NMR
Neonatal other
Neonatal diarrhoea
Neonatal tetanus
Neonatal congenital
Neonatal sepsis or meningitis
Neonatal intrapartum
Neonatal preterm
Neonatal LRI
−0.10
0.00
0.05
0.10
Mortality rate per 1000 live births
Fraction of mortality
Neonatal other
Neonatal diarrhoea
Neonatal tetanus
Neonatal congenital
Neonatal sepsis or meningitis
Neonatal intrapartum
Neonatal preterm
Neonatal LRI
2000 − 2015
2015 − 2019 (not on target)
Deficit to target
2015 − 2019 (on target)

Democratic People's Republic of Korea (Under five)

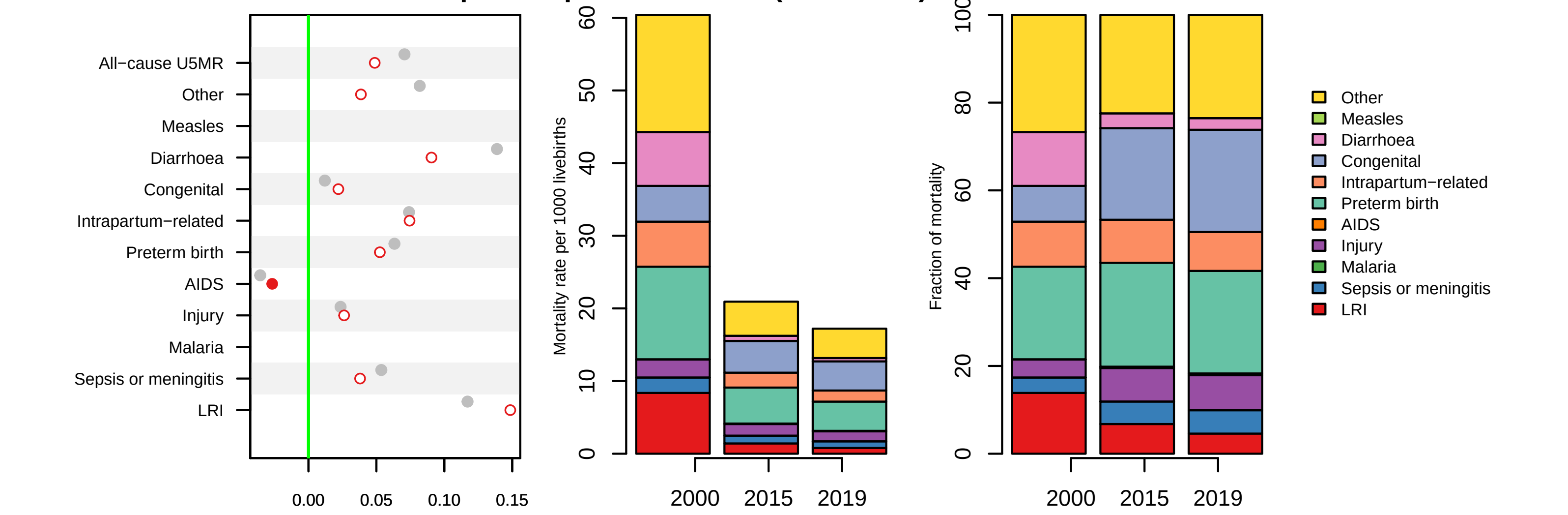

Democratic People's Republic of Korea (Neonatal)

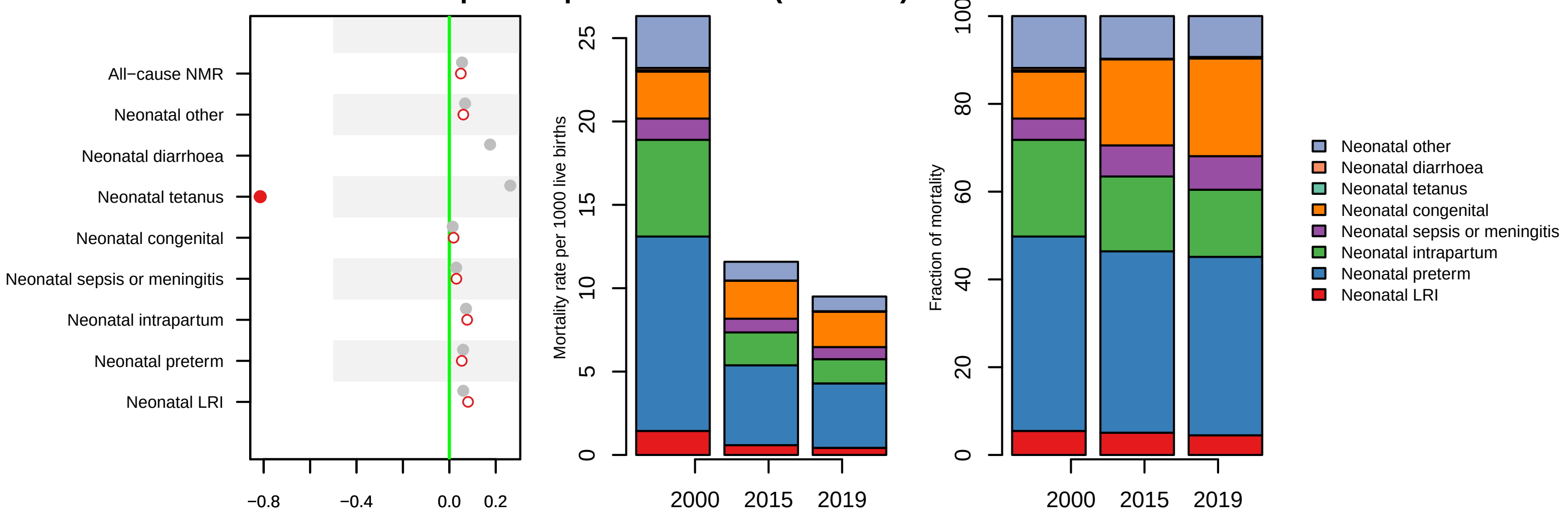

2000 − 2015 | 2015 − 2019 (not on target) | Deficit to target | 2015 − 2019 (on target)

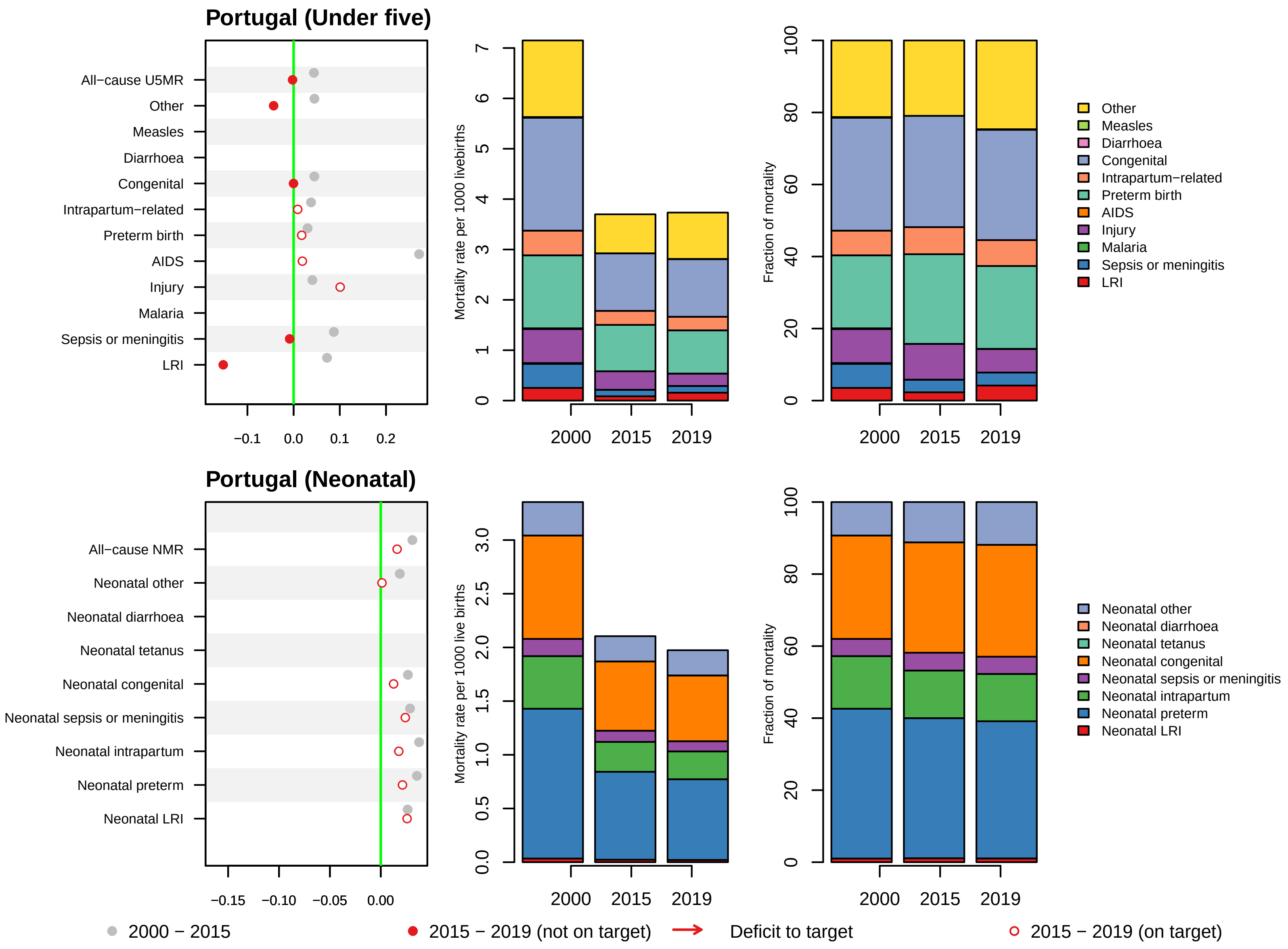

Portugal (Under five)
All−cause U5MR
Other
Measles
Diarrhoea
Congenital
Intrapartum−related
Preterm birth
AIDS
Injury
Malaria
Sepsis or meningitis
LRI
−0.1
0.0
0.1
0.2
Mortality rate per 1000 livebirths
Fraction of mortality
2000
2015
2019
Other
Measles
Diarrhoea
Congenital
Intrapartum−related
Preterm birth
AIDS
Injury
Malaria
Sepsis or meningitis
LRI
Portugal (Neonatal)
All−cause NMR
Neonatal other
Neonatal diarrhoea
Neonatal tetanus
Neonatal congenital
Neonatal sepsis or meningitis
Neonatal intrapartum
Neonatal preterm
Neonatal LRI
−0.15
−0.10
−0.05
0.00
Mortality rate per 1000 live births
Fraction of mortality
2000
2015
2019
Neonatal other
Neonatal diarrhoea
Neonatal tetanus
Neonatal congenital
Neonatal sepsis or meningitis
Neonatal intrapartum
Neonatal preterm
Neonatal LRI
2000 − 2015
2015 − 2019 (not on target)
Deficit to target
2015 − 2019 (on target)

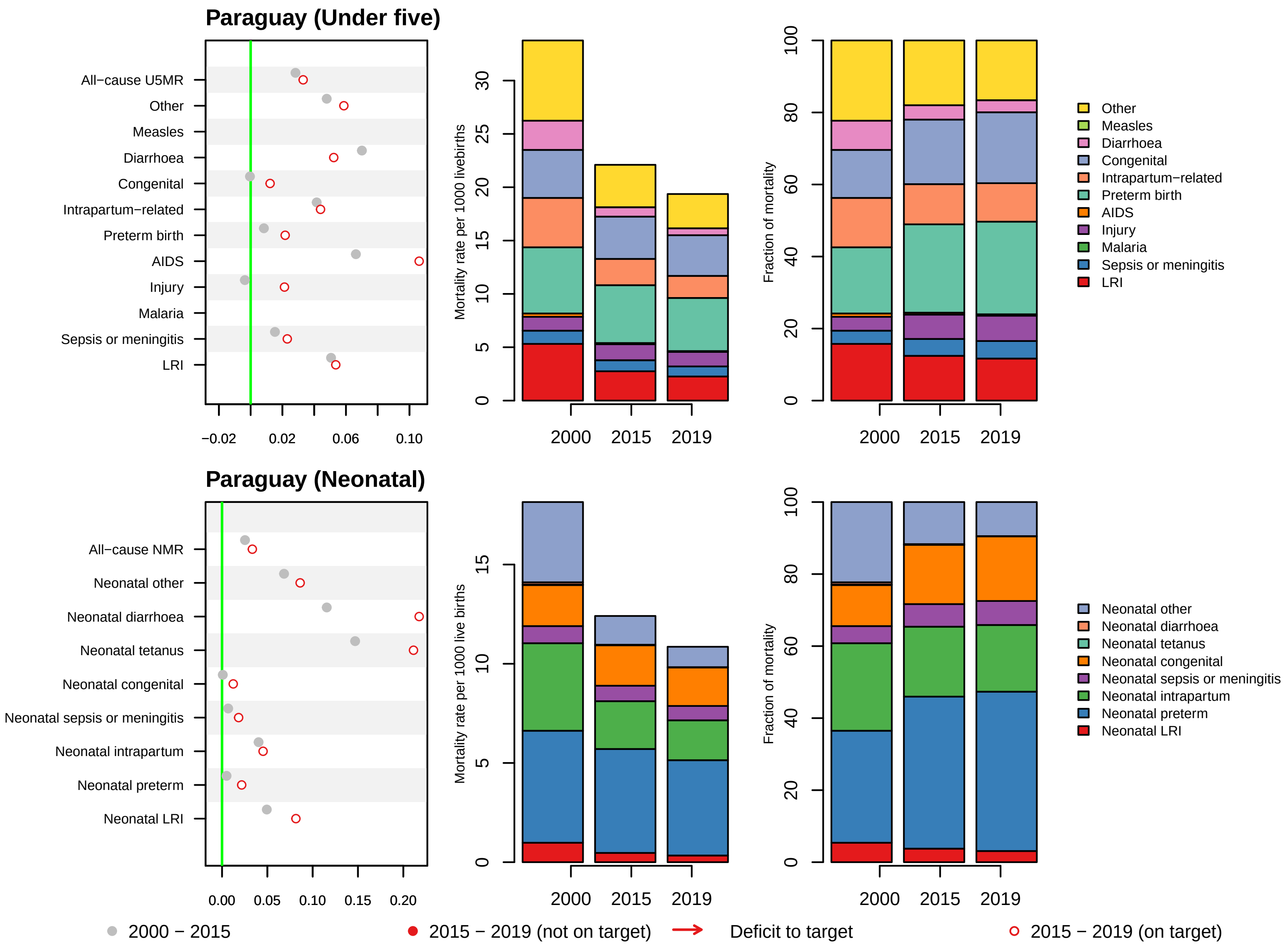

Paraguay (Under five)
All−cause U5MR
Other
Measles
Diarrhoea
Congenital
Intrapartum−related
Preterm birth
AIDS
Injury
Malaria
Sepsis or meningitis
LRI
−0.02
0.02
0.06
0.10
Mortality rate per 1000 livebirths
0
5
10
15
20
25
30
2000
2015
2019
Fraction of mortality
0
20
40
60
80
100
2000
2015
2019
Other
Measles
Diarrhoea
Congenital
Intrapartum−related
Preterm birth
AIDS
Injury
Malaria
Sepsis or meningitis
LRI
Paraguay (Neonatal)
All−cause NMR
Neonatal other
Neonatal diarrhoea
Neonatal tetanus
Neonatal congenital
Neonatal sepsis or meningitis
Neonatal intrapartum
Neonatal preterm
Neonatal LRI
0.00
0.05
0.10
0.15
0.20
Mortality rate per 1000 live births
0
5
10
15
2000
2015
2019
Fraction of mortality
0
20
40
60
80
100
2000
2015
2019
Neonatal other
Neonatal diarrhoea
Neonatal tetanus
Neonatal congenital
Neonatal sepsis or meningitis
Neonatal intrapartum
Neonatal preterm
Neonatal LRI
2000 − 2015
2015 − 2019 (not on target)
Deficit to target
2015 − 2019 (on target)

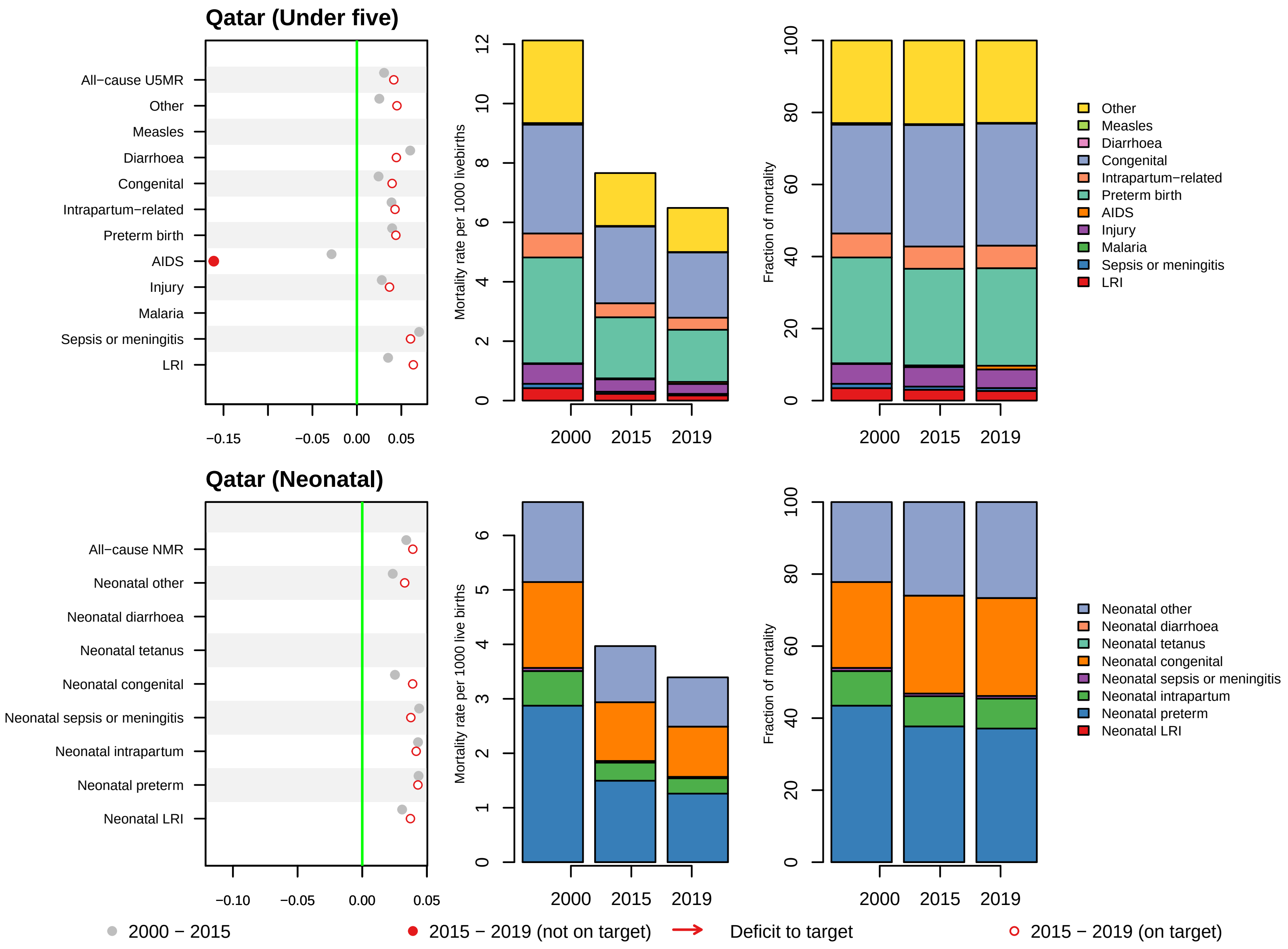

Qatar (Under five)
All−cause U5MR
Other
Measles
Diarrhoea
Congenital
Intrapartum−related
Preterm birth
AIDS
Injury
Malaria
Sepsis or meningitis
LRI
−0.15
−0.05 0.00 0.05
Mortality rate per 1000 livebirths
0 2 4 6 8 10 12
2000 2015 2019
Fraction of mortality
0 20 40 60 80 100
2000 2015 2019
Other
Measles
Diarrhoea
Congenital
Intrapartum−related
Preterm birth
AIDS
Injury
Malaria
Sepsis or meningitis
LRI
Qatar (Neonatal)
All−cause NMR
Neonatal other
Neonatal diarrhoea
Neonatal tetanus
Neonatal congenital
Neonatal sepsis or meningitis
Neonatal intrapartum
Neonatal preterm
Neonatal LRI
−0.10 −0.05 0.00 0.05
Mortality rate per 1000 live births
0 1 2 3 4 5 6
2000 2015 2019
Fraction of mortality
0 20 40 60 80 100
2000 2015 2019
Neonatal other
Neonatal diarrhoea
Neonatal tetanus
Neonatal congenital
Neonatal sepsis or meningitis
Neonatal intrapartum
Neonatal preterm
Neonatal LRI
2000 − 2015
2015 − 2019 (not on target)
Deficit to target
2015 − 2019 (on target)

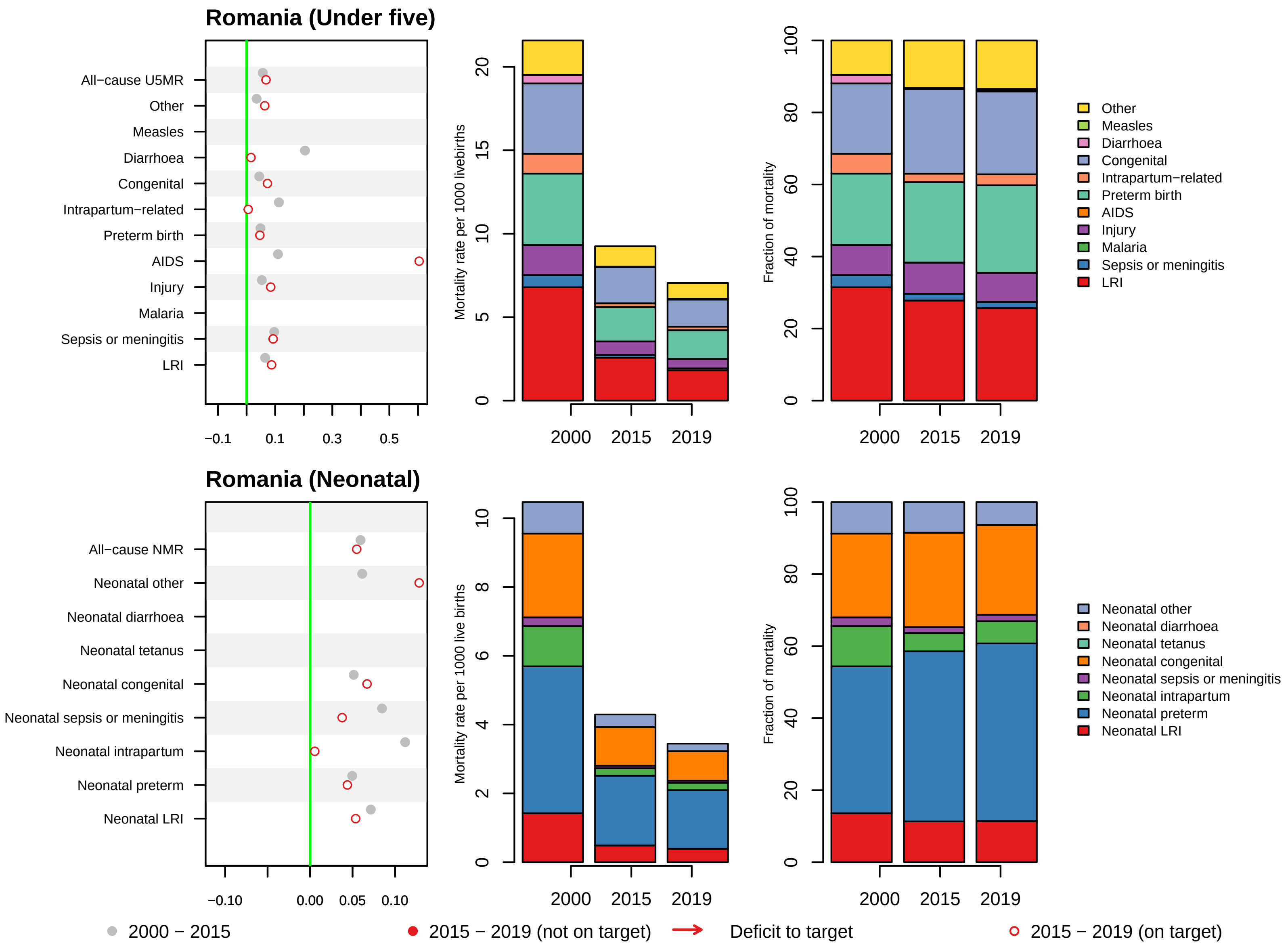
Romania (Under five)
All−cause U5MR
Other
Measles
Diarrhoea
Congenital
Intrapartum−related
Preterm birth
AIDS
Injury
Malaria
Sepsis or meningitis
LRI
−0.1
0.1
0.3
0.5
Mortality rate per 1000 livebirths
0
5
10
15
20
2000
2015
2019
Fraction of mortality
0
20
40
60
80
100
Other
Measles
Diarrhoea
Congenital
Intrapartum−related
Preterm birth
AIDS
Injury
Malaria
Sepsis or meningitis
LRI
Romania (Neonatal)
All−cause NMR
Neonatal other
Neonatal diarrhoea
Neonatal tetanus
Neonatal congenital
Neonatal sepsis or meningitis
Neonatal intrapartum
Neonatal preterm
Neonatal LRI
−0.10
0.00
0.05
0.10
Mortality rate per 1000 live births
0
2
4
6
8
10
Neonatal other
Neonatal diarrhoea
Neonatal tetanus
Neonatal congenital
Neonatal sepsis or meningitis
Neonatal intrapartum
Neonatal preterm
Neonatal LRI
2000 − 2015
2015 − 2019 (not on target)
Deficit to target
2015 − 2019 (on target)

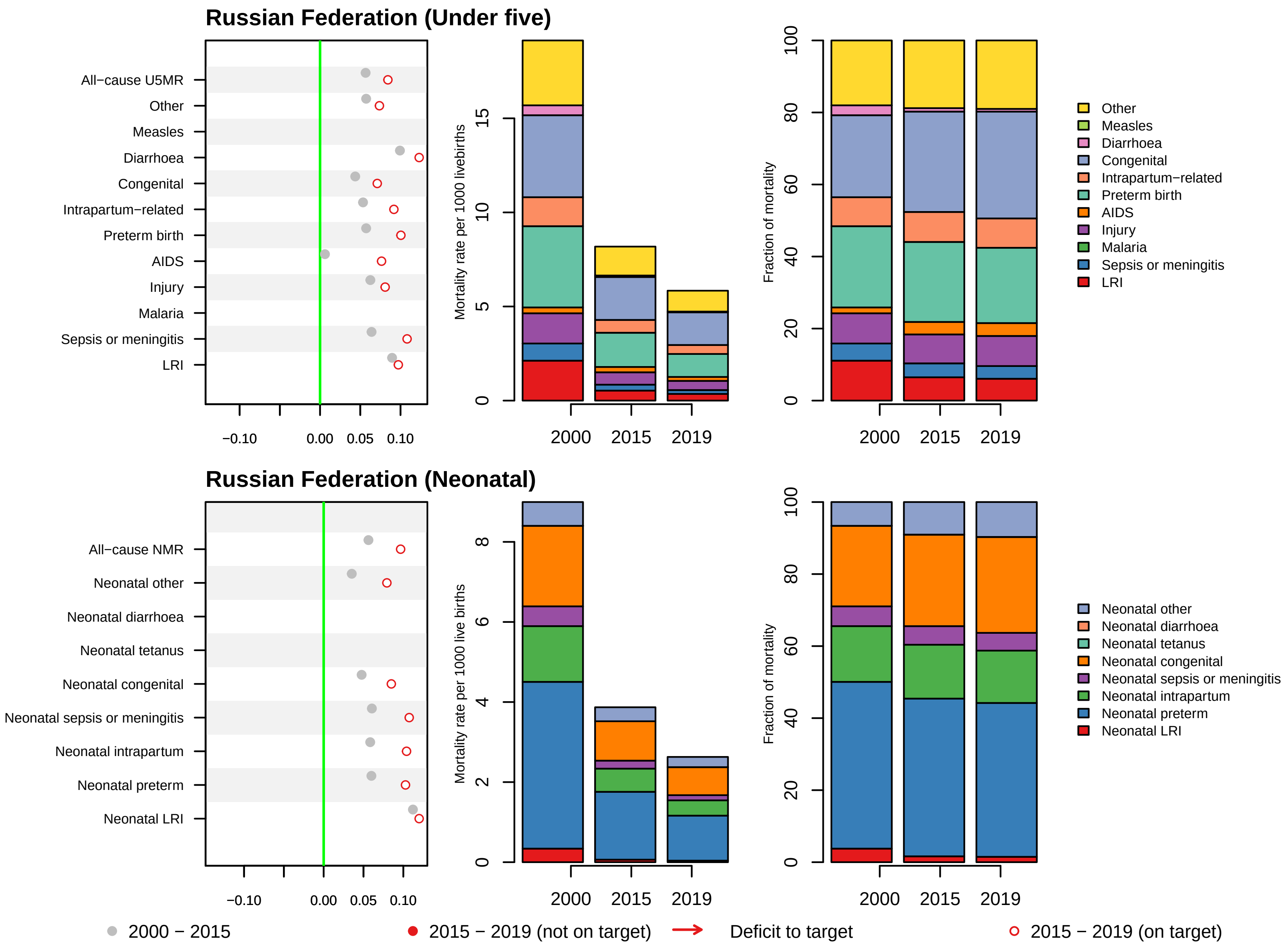

Russian Federation (Under five)
All−cause U5MR
Other
Measles
Diarrhoea
Congenital
Intrapartum−related
Preterm birth
AIDS
Injury
Malaria
Sepsis or meningitis
LRI
−0.10
0.00
0.05
0.10
Mortality rate per 1000 livebirths
0
5
10
15
2000
2015
2019
Fraction of mortality
0
20
40
60
80
100
Other
Measles
Diarrhoea
Congenital
Intrapartum−related
Preterm birth
AIDS
Injury
Malaria
Sepsis or meningitis
LRI
Russian Federation (Neonatal)
All−cause NMR
Neonatal other
Neonatal diarrhoea
Neonatal tetanus
Neonatal congenital
Neonatal sepsis or meningitis
Neonatal intrapartum
Neonatal preterm
Neonatal LRI
Mortality rate per 1000 live births
0
2
4
6
8
Neonatal other
Neonatal diarrhoea
Neonatal tetanus
Neonatal congenital
Neonatal sepsis or meningitis
Neonatal intrapartum
Neonatal preterm
Neonatal LRI
2000 − 2015
2015 − 2019 (not on target)
Deficit to target
2015 − 2019 (on target)

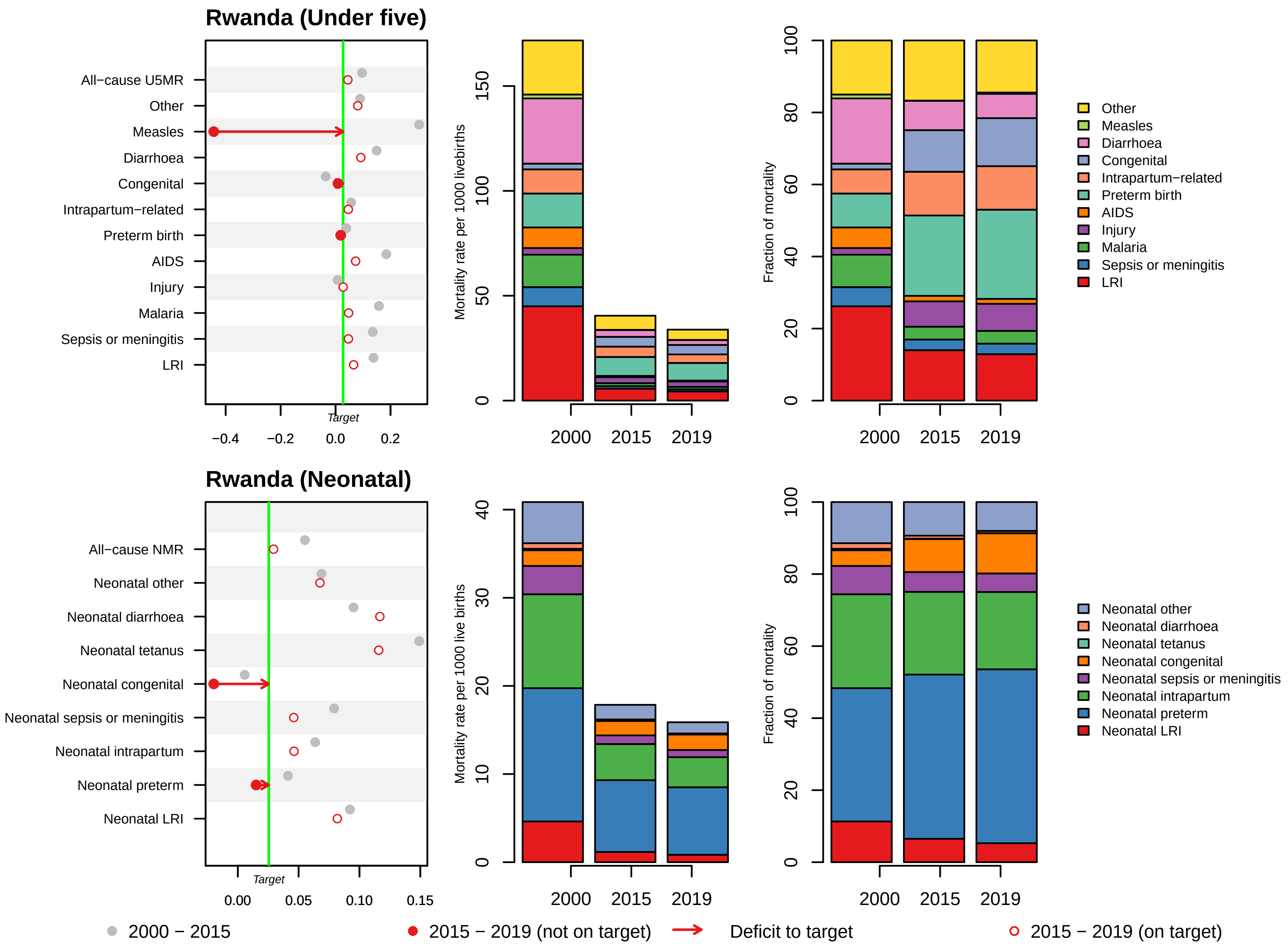

Rwanda (Under five)
All−cause U5MR
Other
Measles
Diarrhoea
Congenital
Intrapartum−related
Preterm birth
AIDS
Injury
Malaria
Sepsis or meningitis
LRI
Target
−0.4
−0.2
0.0
0.2
Mortality rate per 1000 livebirths
Fraction of mortality
2000
2015
2019
Other
Measles
Diarrhoea
Congenital
Intrapartum−related
Preterm birth
AIDS
Injury
Malaria
Sepsis or meningitis
LRI
Rwanda (Neonatal)
All−cause NMR
Neonatal other
Neonatal diarrhoea
Neonatal tetanus
Neonatal congenital
Neonatal sepsis or meningitis
Neonatal intrapartum
Neonatal preterm
Neonatal LRI
Target
0.00
0.05
0.10
0.15
Mortality rate per 1000 live births
Fraction of mortality
Neonatal other
Neonatal diarrhoea
Neonatal tetanus
Neonatal congenital
Neonatal sepsis or meningitis
Neonatal intrapartum
Neonatal preterm
Neonatal LRI
2000 − 2015
2015 − 2019 (not on target)
Deficit to target
2015 − 2019 (on target)

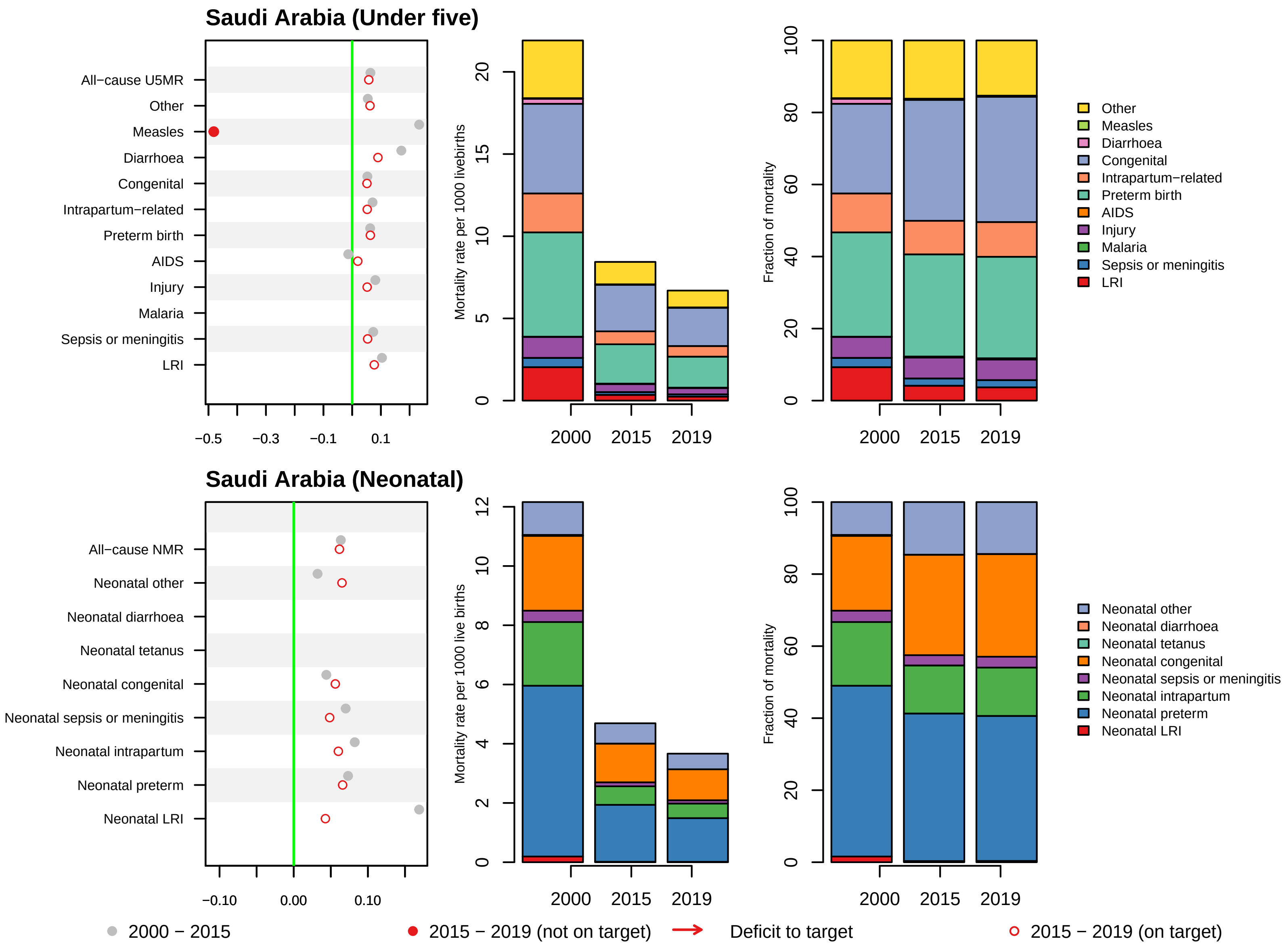
Saudi Arabia (Under five)
All−cause U5MR
Other
Measles
Diarrhoea
Congenital
Intrapartum−related
Preterm birth
AIDS
Injury
Malaria
Sepsis or meningitis
LRI
−0.5
−0.3
−0.1
0.1
Mortality rate per 1000 livebirths
0
5
10
15
20
2000
2015
2019
Fraction of mortality
0
20
40
60
80
100
2000
2015
2019
Other
Measles
Diarrhoea
Congenital
Intrapartum−related
Preterm birth
AIDS
Injury
Malaria
Sepsis or meningitis
LRI
Saudi Arabia (Neonatal)
All−cause NMR
Neonatal other
Neonatal diarrhoea
Neonatal tetanus
Neonatal congenital
Neonatal sepsis or meningitis
Neonatal intrapartum
Neonatal preterm
Neonatal LRI
−0.10
0.00
0.10
Mortality rate per 1000 live births
0
2
4
6
8
10
12
2000
2015
2019
Fraction of mortality
0
20
40
60
80
100
2000
2015
2019
Neonatal other
Neonatal diarrhoea
Neonatal tetanus
Neonatal congenital
Neonatal sepsis or meningitis
Neonatal intrapartum
Neonatal preterm
Neonatal LRI
2000 − 2015
2015 − 2019 (not on target)
Deficit to target
2015 − 2019 (on target)

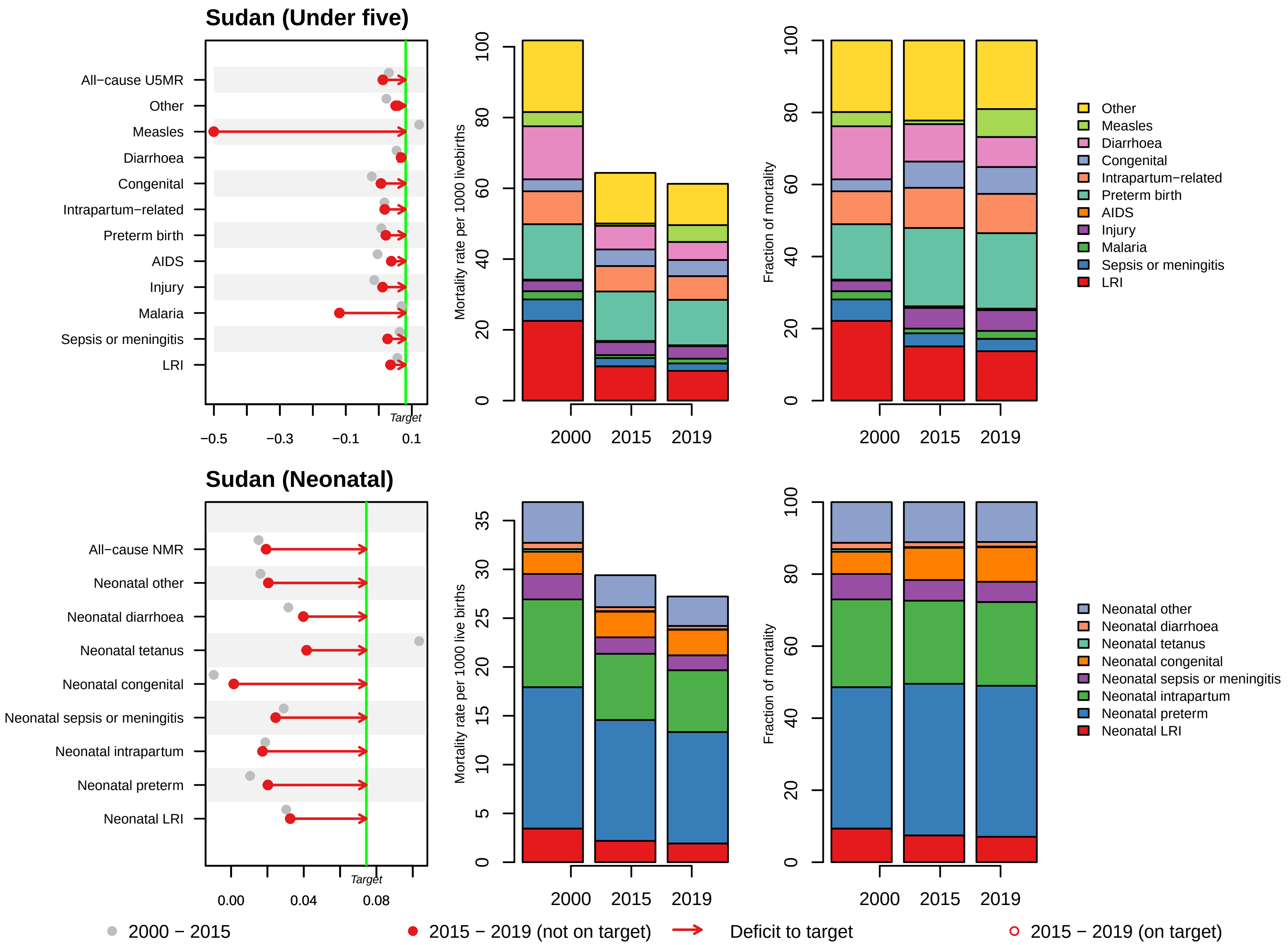
Sudan (Under five)
All−cause U5MR
Other
Measles
Diarrhoea
Congenital
Intrapartum−related
Preterm birth
AIDS
Injury
Malaria
Sepsis or meningitis
LRI
Target
−0.5
−0.3
−0.1
0.1
Mortality rate per 1000 livebirths
0
20
40
60
80
100
2000
2015
2019
Fraction of mortality
Other
Measles
Diarrhoea
Congenital
Intrapartum−related
Preterm birth
AIDS
Injury
Malaria
Sepsis or meningitis
LRI
Sudan (Neonatal)
All−cause NMR
Neonatal other
Neonatal diarrhoea
Neonatal tetanus
Neonatal congenital
Neonatal sepsis or meningitis
Neonatal intrapartum
Neonatal preterm
Neonatal LRI
Target
0.00
0.04
0.08
Mortality rate per 1000 live births
0
5
10
15
20
25
30
35
Fraction of mortality
Neonatal other
Neonatal diarrhoea
Neonatal tetanus
Neonatal congenital
Neonatal sepsis or meningitis
Neonatal intrapartum
Neonatal preterm
Neonatal LRI
2000 − 2015
2015 − 2019 (not on target)
Deficit to target
2015 − 2019 (on target)

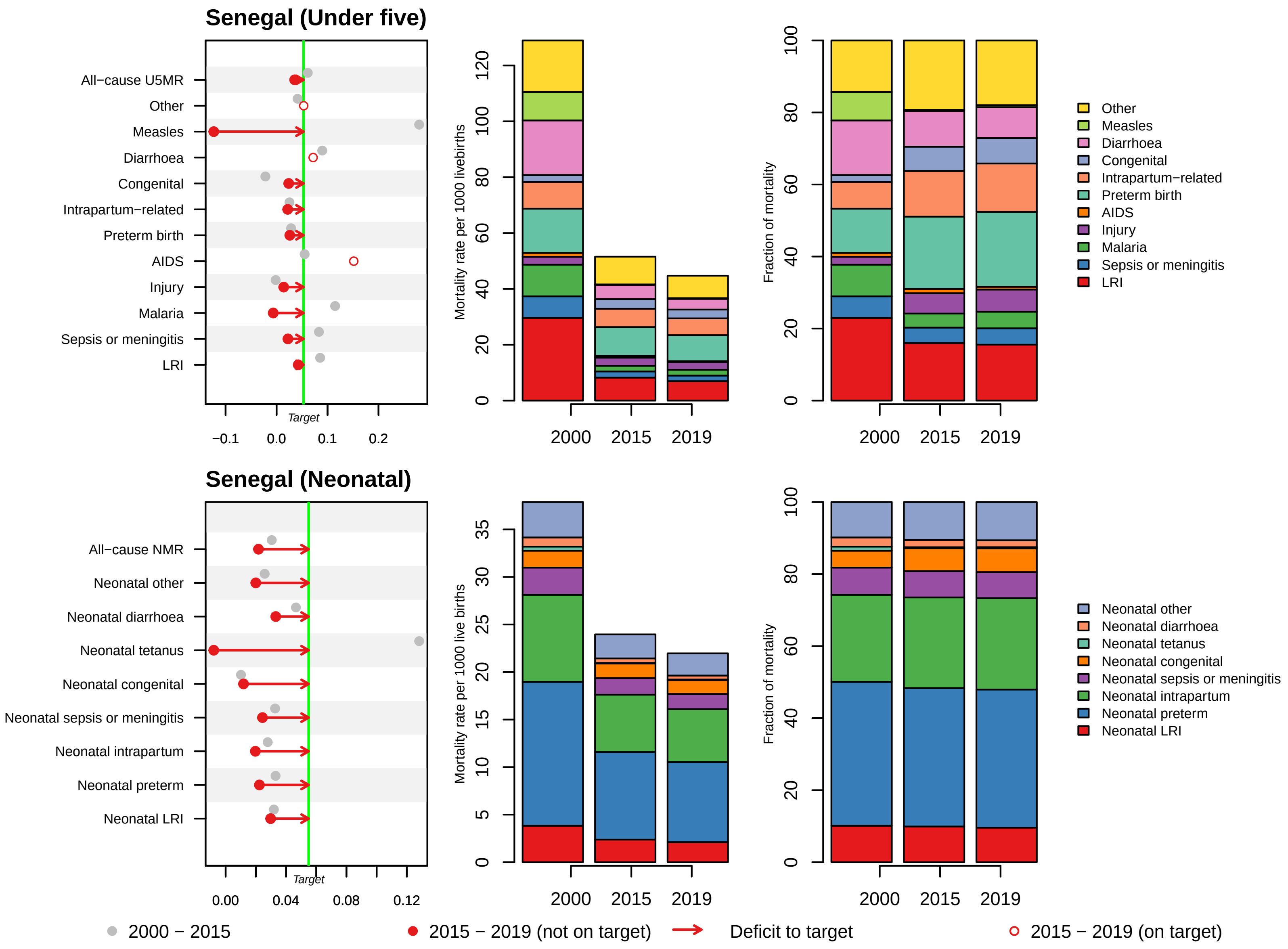

Senegal (Under five)
All−cause U5MR
Other
Measles
Diarrhoea
Congenital
Intrapartum−related
Preterm birth
AIDS
Injury
Malaria
Sepsis or meningitis
LRI
Target
−0.1
0.0
0.1
0.2
Mortality rate per 1000 livebirths
Fraction of mortality
2000
2015
2019
Other
Measles
Diarrhoea
Congenital
Intrapartum−related
Preterm birth
AIDS
Injury
Malaria
Sepsis or meningitis
LRI
Senegal (Neonatal)
All−cause NMR
Neonatal other
Neonatal diarrhoea
Neonatal tetanus
Neonatal congenital
Neonatal sepsis or meningitis
Neonatal intrapartum
Neonatal preterm
Neonatal LRI
0.00
0.04
0.08
0.12
Mortality rate per 1000 live births
Neonatal other
Neonatal diarrhoea
Neonatal tetanus
Neonatal congenital
Neonatal sepsis or meningitis
Neonatal intrapartum
Neonatal preterm
Neonatal LRI
2000 − 2015
2015 − 2019 (not on target)
Deficit to target
2015 − 2019 (on target)

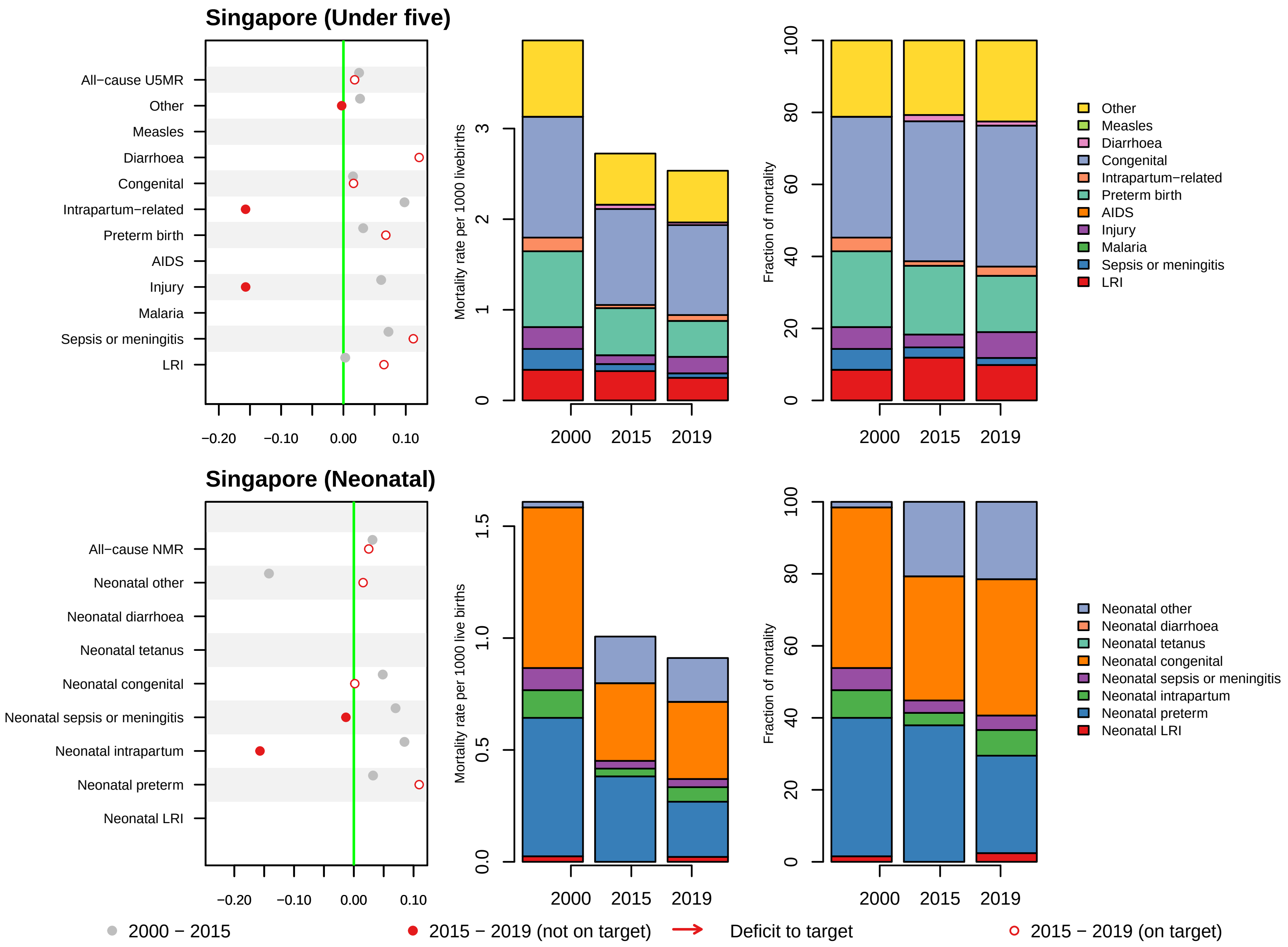
Singapore (Under five)
All−cause U5MR
Other
Measles
Diarrhoea
Congenital
Intrapartum−related
Preterm birth
AIDS
Injury
Malaria
Sepsis or meningitis
LRI
−0.20
−0.10
0.00
0.10
Mortality rate per 1000 livebirths
Fraction of mortality
2000
2015
2019
Other
Measles
Diarrhoea
Congenital
Intrapartum−related
Preterm birth
AIDS
Injury
Malaria
Sepsis or meningitis
LRI
Singapore (Neonatal)
All−cause NMR
Neonatal other
Neonatal diarrhoea
Neonatal tetanus
Neonatal congenital
Neonatal sepsis or meningitis
Neonatal intrapartum
Neonatal preterm
Neonatal LRI
Mortality rate per 1000 live births
Fraction of mortality
2000 − 2015
2015 − 2019 (not on target)
Deficit to target
2015 − 2019 (on target)

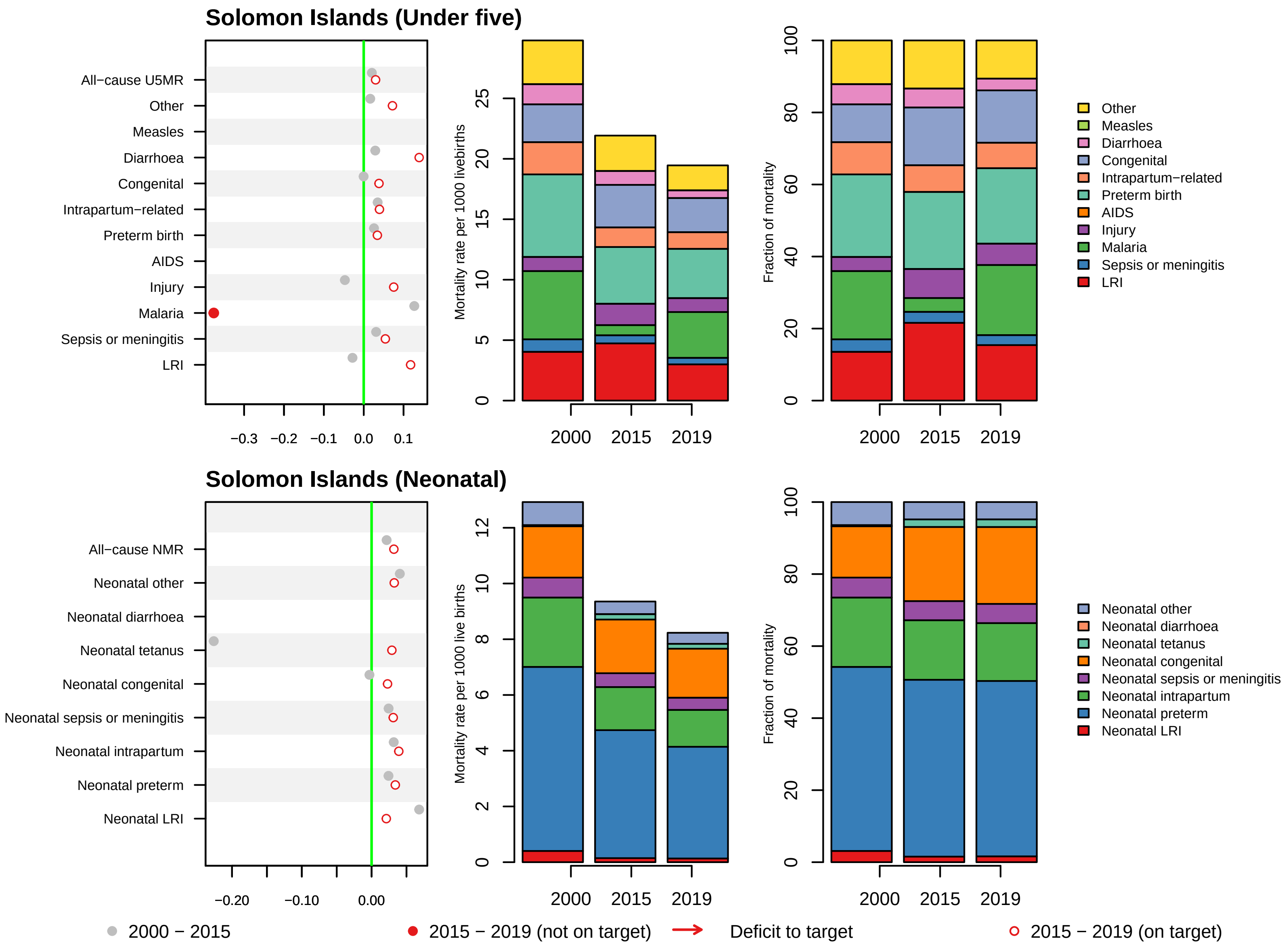
Solomon Islands (Under five)
All−cause U5MR
Other
Measles
Diarrhoea
Congenital
Intrapartum−related
Preterm birth
AIDS
Injury
Malaria
Sepsis or meningitis
LRI
−0.3
−0.2
−0.1
0.0
0.1
Mortality rate per 1000 livebirths
0
5
10
15
20
25
2000
2015
2019
Fraction of mortality
0
20
40
60
80
100
Other
Measles
Diarrhoea
Congenital
Intrapartum−related
Preterm birth
AIDS
Injury
Malaria
Sepsis or meningitis
LRI
Solomon Islands (Neonatal)
All−cause NMR
Neonatal other
Neonatal diarrhoea
Neonatal tetanus
Neonatal congenital
Neonatal sepsis or meningitis
Neonatal intrapartum
Neonatal preterm
Neonatal LRI
−0.20
−0.10
0.00
Mortality rate per 1000 live births
0
2
4
6
8
10
12
Neonatal other
Neonatal diarrhoea
Neonatal tetanus
Neonatal congenital
Neonatal sepsis or meningitis
Neonatal intrapartum
Neonatal preterm
Neonatal LRI
2000 − 2015
2015 − 2019 (not on target)
Deficit to target
2015 − 2019 (on target)

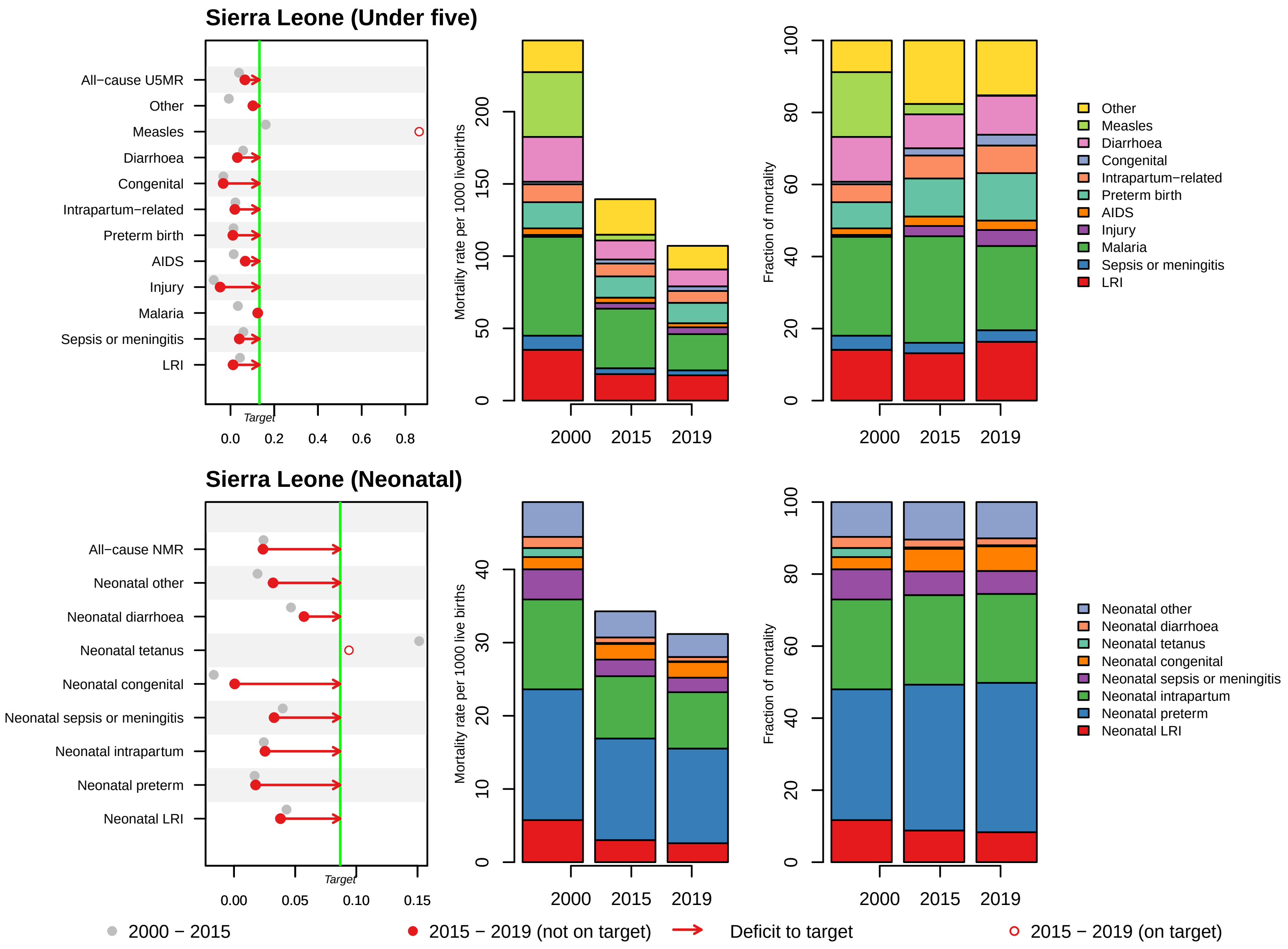

Sierra Leone (Under five)
All−cause U5MR
Other
Measles
Diarrhoea
Congenital
Intrapartum−related
Preterm birth
AIDS
Injury
Malaria
Sepsis or meningitis
LRI
Target
0.0
0.2
0.4
0.6
0.8
Mortality rate per 1000 livebirths
Fraction of mortality
2000
2015
2019
Sierra Leone (Neonatal)
All−cause NMR
Neonatal other
Neonatal diarrhoea
Neonatal tetanus
Neonatal congenital
Neonatal sepsis or meningitis
Neonatal intrapartum
Neonatal preterm
Neonatal LRI
0.00
0.05
0.10
0.15
Mortality rate per 1000 live births
2000 − 2015
2015 − 2019 (not on target)
Deficit to target
2015 − 2019 (on target)

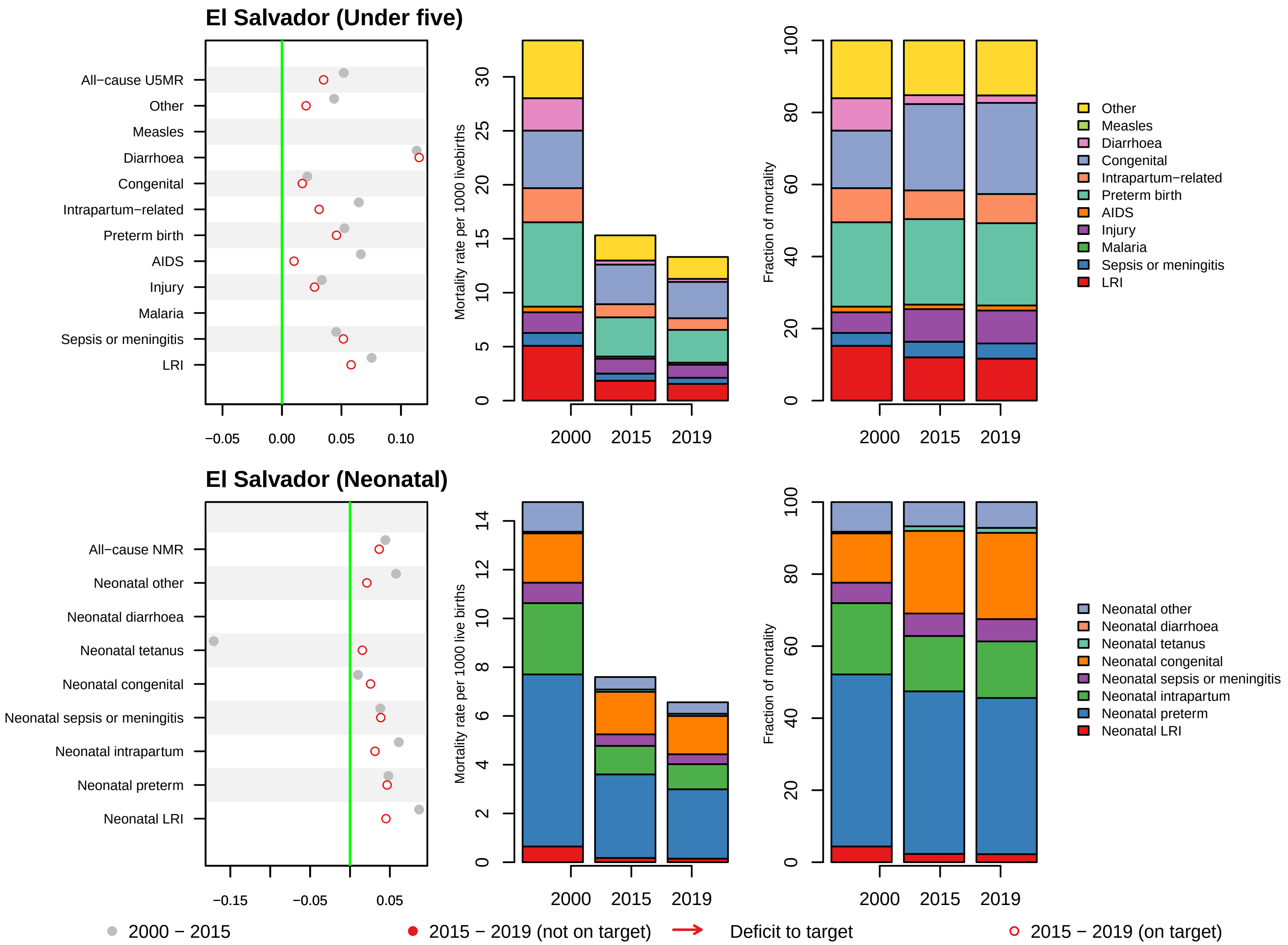

El Salvador (Under five)
All−cause U5MR
Other
Measles
Diarrhoea
Congenital
Intrapartum−related
Preterm birth
AIDS
Injury
Malaria
Sepsis or meningitis
LRI
−0.05
0.00
0.05
0.10
Mortality rate per 1000 livebirths
Fraction of mortality
2000
2015
2019
El Salvador (Neonatal)
All−cause NMR
Neonatal other
Neonatal diarrhoea
Neonatal tetanus
Neonatal congenital
Neonatal sepsis or meningitis
Neonatal intrapartum
Neonatal preterm
Neonatal LRI
−0.15
−0.05
0.05
Mortality rate per 1000 live births
2000 − 2015
2015 − 2019 (not on target)
Deficit to target
2015 − 2019 (on target)

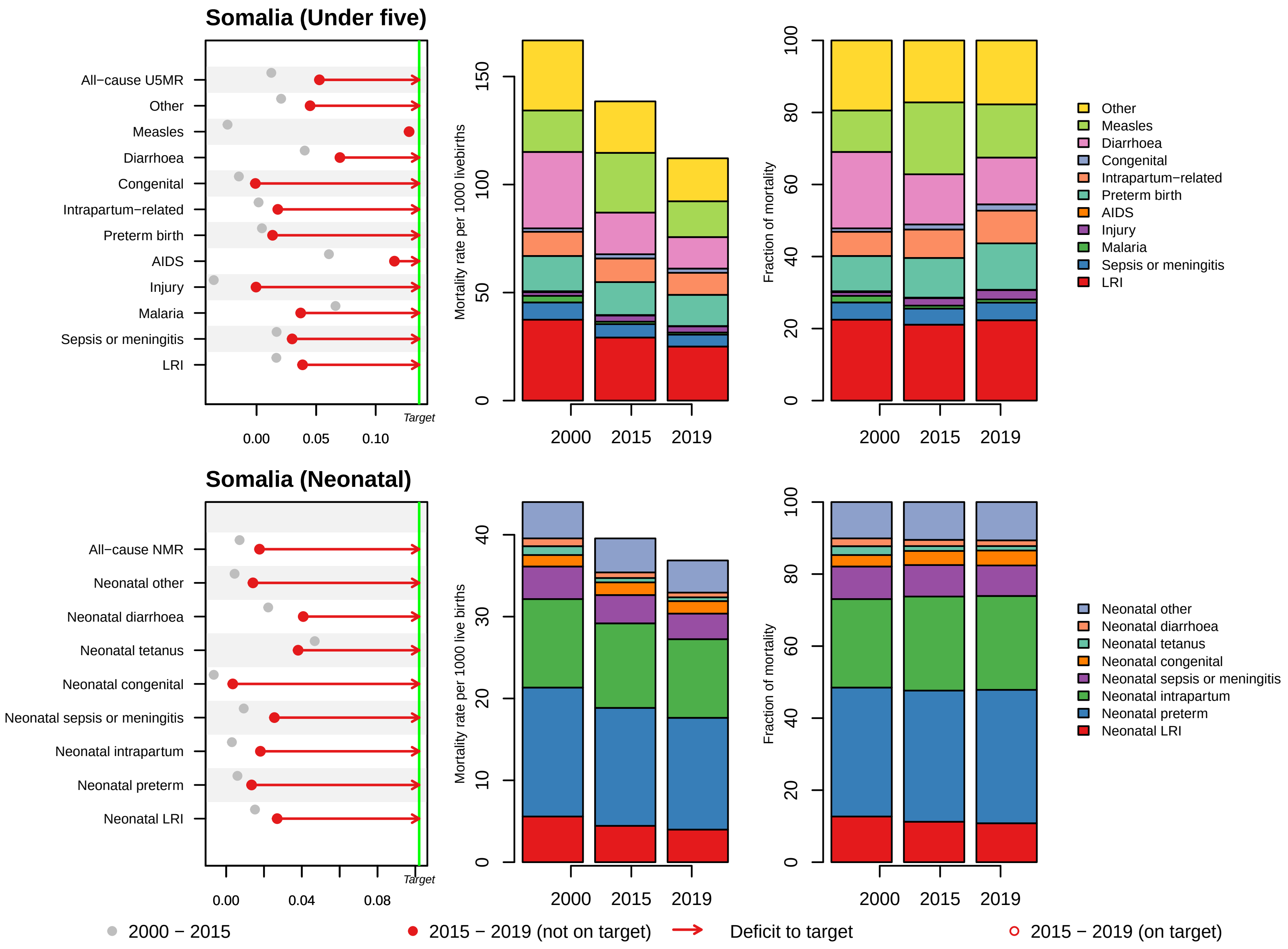

Somalia (Under five)
All−cause U5MR
Other
Measles
Diarrhoea
Congenital
Intrapartum−related
Preterm birth
AIDS
Injury
Malaria
Sepsis or meningitis
LRI
Target
0.00
0.05
0.10
Mortality rate per 1000 livebirths
0
50
100
150
2000
2015
2019
Fraction of mortality
0
20
40
60
80
100
Other
Measles
Diarrhoea
Congenital
Intrapartum−related
Preterm birth
AIDS
Injury
Malaria
Sepsis or meningitis
LRI
Somalia (Neonatal)
All−cause NMR
Neonatal other
Neonatal diarrhoea
Neonatal tetanus
Neonatal congenital
Neonatal sepsis or meningitis
Neonatal intrapartum
Neonatal preterm
Neonatal LRI
Target
0.00
0.04
0.08
Mortality rate per 1000 live births
0
10
20
30
40
2000
2015
2019
Fraction of mortality
0
20
40
60
80
100
Neonatal other
Neonatal diarrhoea
Neonatal tetanus
Neonatal congenital
Neonatal sepsis or meningitis
Neonatal intrapartum
Neonatal preterm
Neonatal LRI
2000 − 2015
2015 − 2019 (not on target)
Deficit to target
2015 − 2019 (on target)

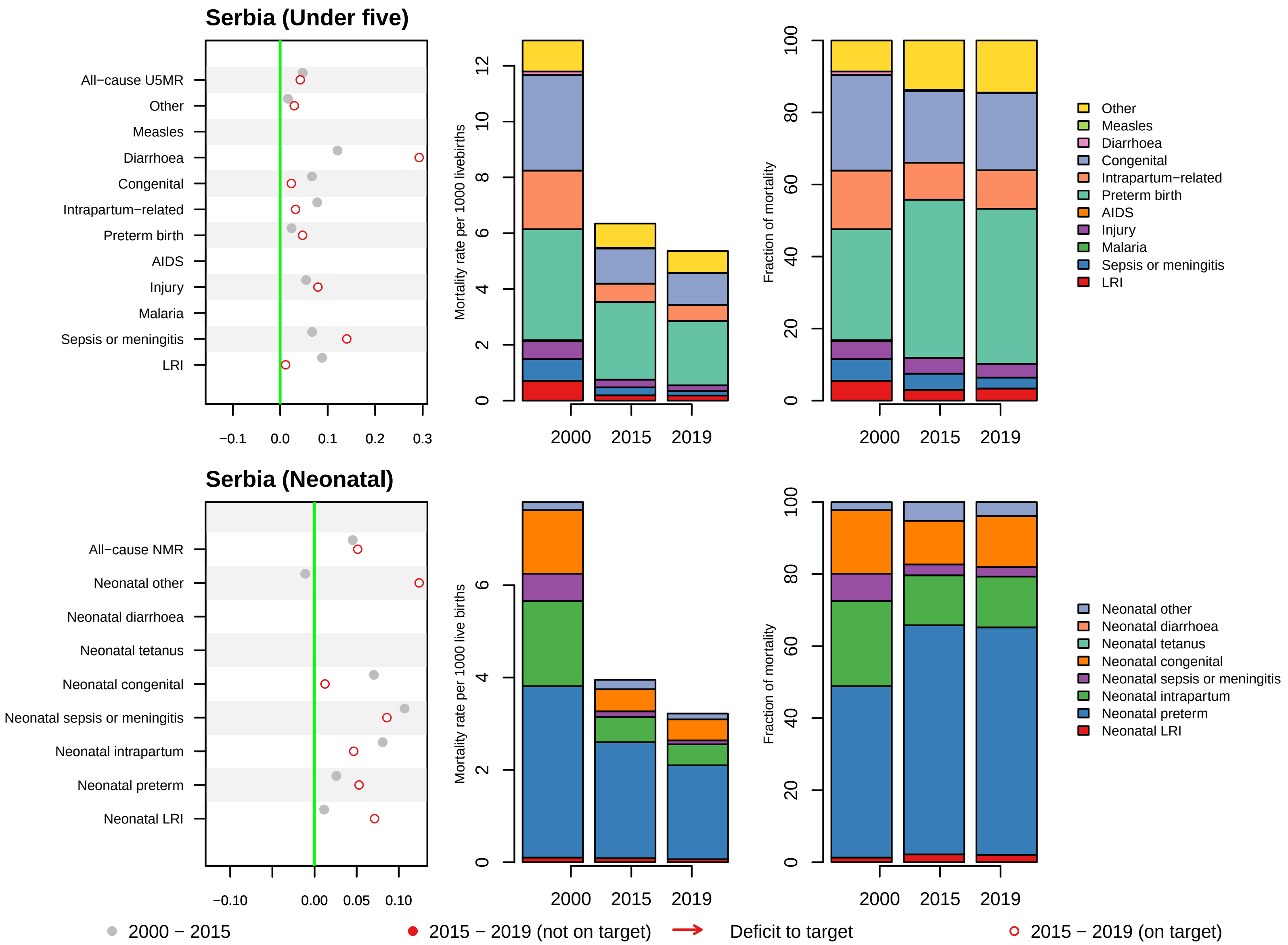
Serbia (Under five)
All−cause U5MR
Other
Measles
Diarrhoea
Congenital
Intrapartum−related
Preterm birth
AIDS
Injury
Malaria
Sepsis or meningitis
LRI
−0.1
0.0
0.1
0.2
0.3
Mortality rate per 1000 livebirths
Fraction of mortality
2000
2015
2019
Other
Measles
Diarrhoea
Congenital
Intrapartum−related
Preterm birth
AIDS
Injury
Malaria
Sepsis or meningitis
LRI
Serbia (Neonatal)
All−cause NMR
Neonatal other
Neonatal diarrhoea
Neonatal tetanus
Neonatal congenital
Neonatal sepsis or meningitis
Neonatal intrapartum
Neonatal preterm
Neonatal LRI
−0.10
0.00
0.05
0.10
Mortality rate per 1000 live births
Fraction of mortality
2000
2015
2019
Neonatal other
Neonatal diarrhoea
Neonatal tetanus
Neonatal congenital
Neonatal sepsis or meningitis
Neonatal intrapartum
Neonatal preterm
Neonatal LRI
2000 − 2015
2015 − 2019 (not on target)
Deficit to target
2015 − 2019 (on target)

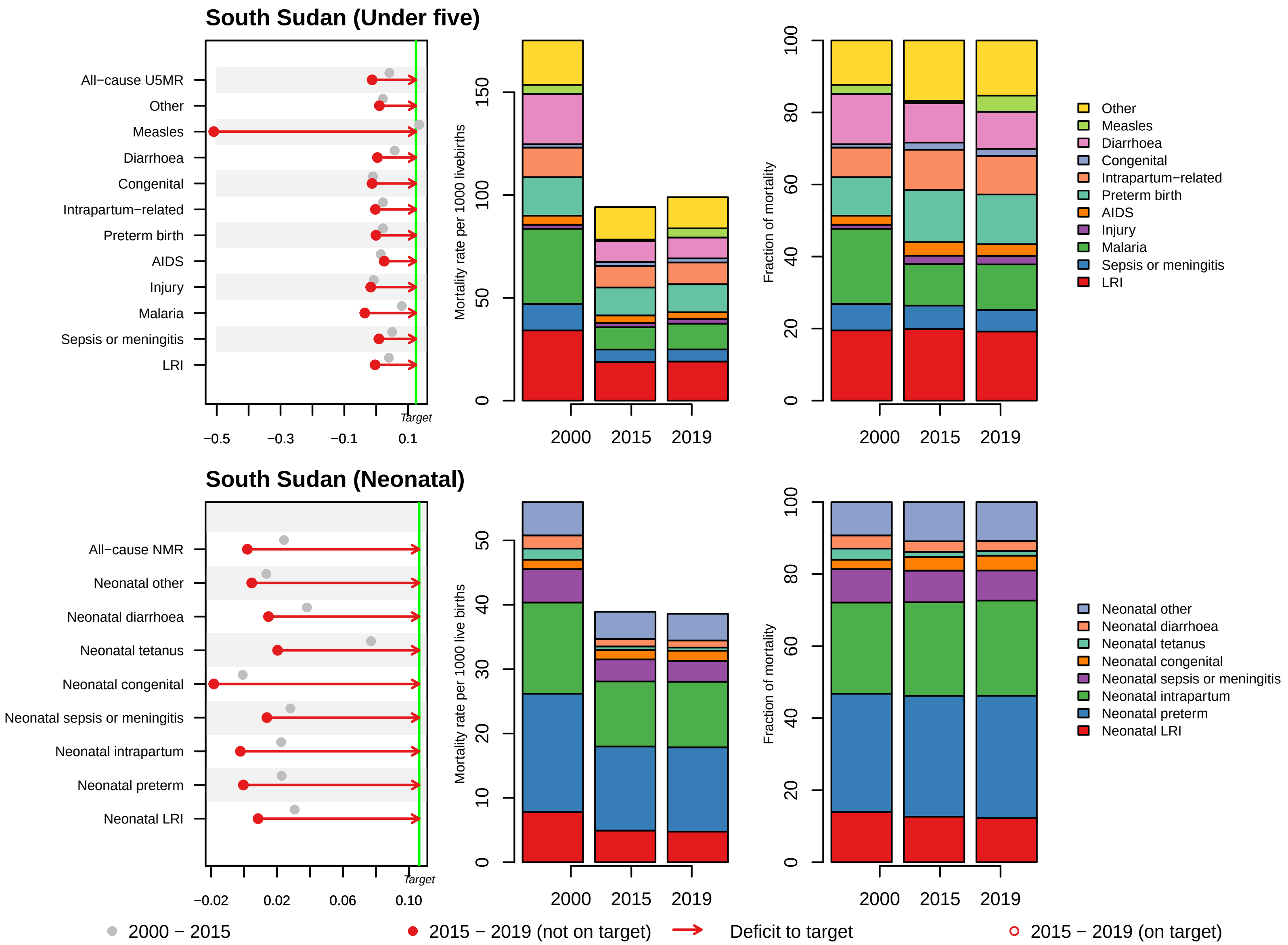
South Sudan (Under five)
All−cause U5MR
Other
Measles
Diarrhoea
Congenital
Intrapartum−related
Preterm birth
AIDS
Injury
Malaria
Sepsis or meningitis
LRI
Target
−0.5
−0.3
−0.1
0.1
Mortality rate per 1000 livebirths
Fraction of mortality
2000
2015
2019
Other
Measles
Diarrhoea
Congenital
Intrapartum−related
Preterm birth
AIDS
Injury
Malaria
Sepsis or meningitis
LRI
South Sudan (Neonatal)
All−cause NMR
Neonatal other
Neonatal diarrhoea
Neonatal tetanus
Neonatal congenital
Neonatal sepsis or meningitis
Neonatal intrapartum
Neonatal preterm
Neonatal LRI
−0.02
0.02
0.06
0.10
Mortality rate per 1000 live births
Neonatal other
Neonatal diarrhoea
Neonatal tetanus
Neonatal congenital
Neonatal sepsis or meningitis
Neonatal intrapartum
Neonatal preterm
Neonatal LRI
2000 − 2015
2015 − 2019 (not on target)
Deficit to target
2015 − 2019 (on target)

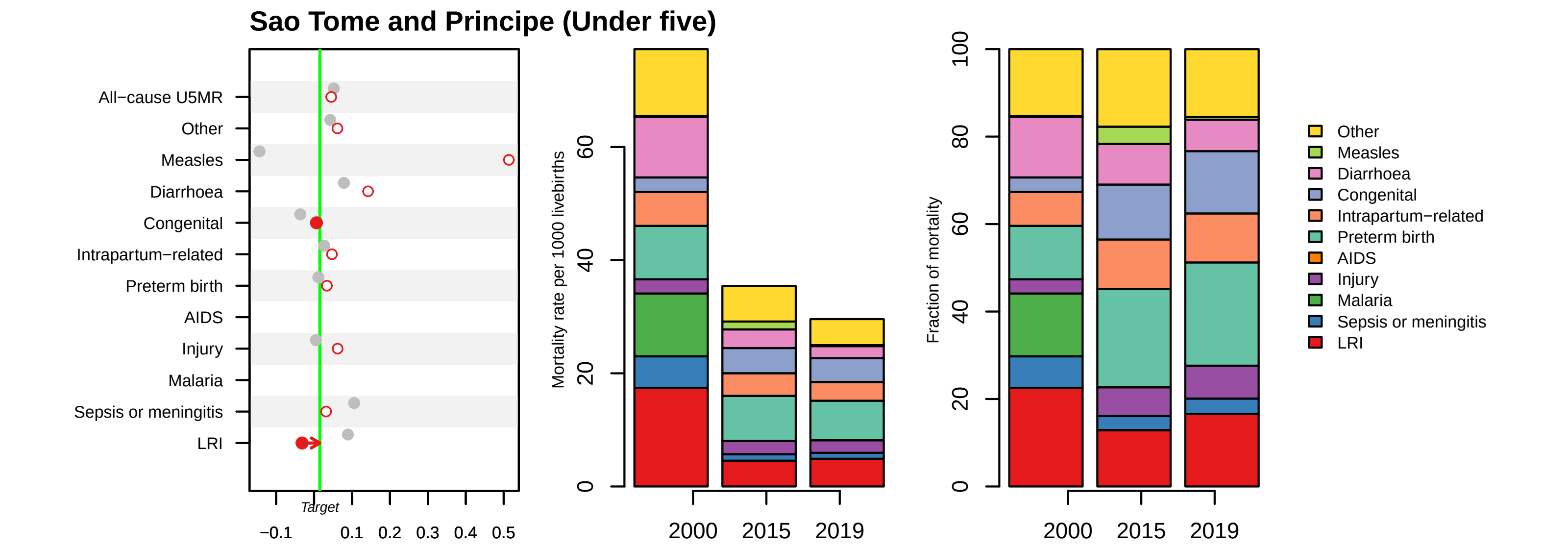
Sao Tome and Principe (Under five)
All−cause U5MR
Other
Measles
Diarrhoea
Congenital
Intrapartum−related
Preterm birth
AIDS
Injury
Malaria
Sepsis or meningitis
LRI
Target
−0.1
0.1
0.2
0.3
0.4
0.5
Mortality rate per 1000 livebirths
Fraction of mortality
2000
2015
2019
Other
Measles
Diarrhoea
Congenital
Intrapartum−related
Preterm birth
AIDS
Injury
Malaria
Sepsis or meningitis
LRI

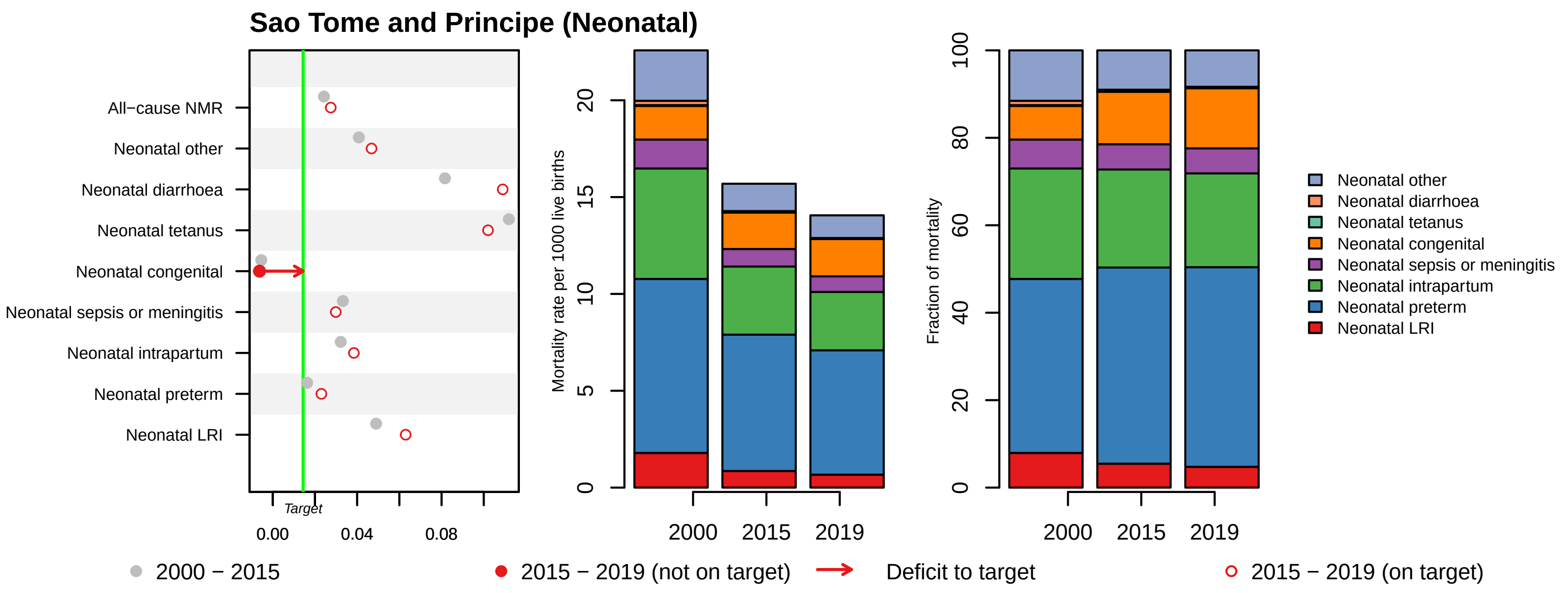
Sao Tome and Principe (Neonatal)
All−cause NMR
Neonatal other
Neonatal diarrhoea
Neonatal tetanus
Neonatal congenital
Neonatal sepsis or meningitis
Neonatal intrapartum
Neonatal preterm
Neonatal LRI
Target
0.00
0.04
0.08
Mortality rate per 1000 live births
Fraction of mortality
2000
2015
2019
Neonatal other
Neonatal diarrhoea
Neonatal tetanus
Neonatal congenital
Neonatal sepsis or meningitis
Neonatal intrapartum
Neonatal preterm
Neonatal LRI
2000 − 2015
2015 − 2019 (not on target)
Deficit to target
2015 − 2019 (on target)

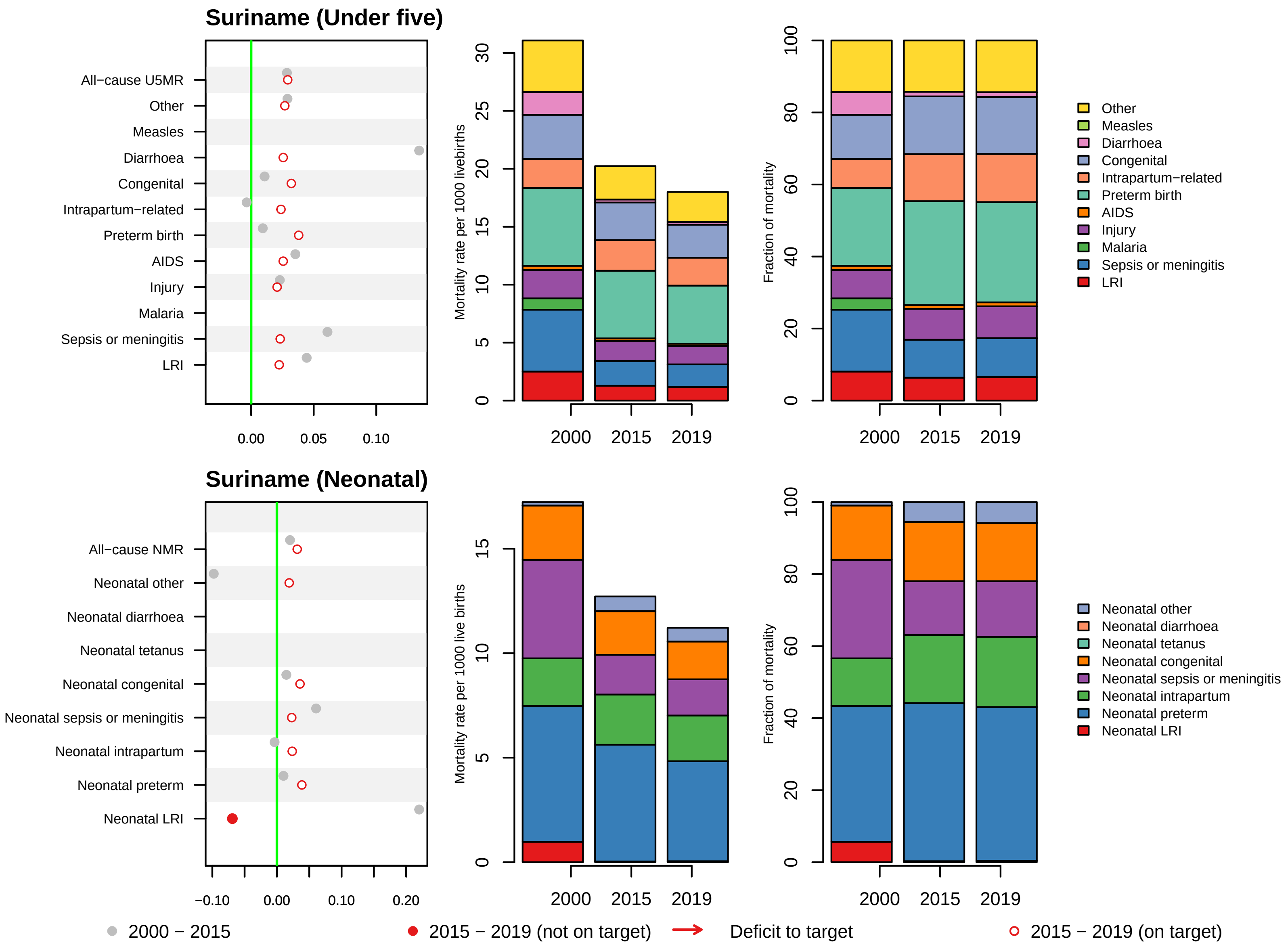

Suriname (Under five)
All−cause U5MR
Other
Measles
Diarrhoea
Congenital
Intrapartum−related
Preterm birth
AIDS
Injury
Malaria
Sepsis or meningitis
LRI
0.00
0.05
0.10
Mortality rate per 1000 livebirths
0
5
10
15
20
25
30
2000
2015
2019
Fraction of mortality
0
20
40
60
80
100
Suriname (Neonatal)
All−cause NMR
Neonatal other
Neonatal diarrhoea
Neonatal tetanus
Neonatal congenital
Neonatal sepsis or meningitis
Neonatal intrapartum
Neonatal preterm
Neonatal LRI
−0.10
0.00
0.10
0.20
Mortality rate per 1000 live births
2000 − 2015
2015 − 2019 (not on target)
Deficit to target
2015 − 2019 (on target)

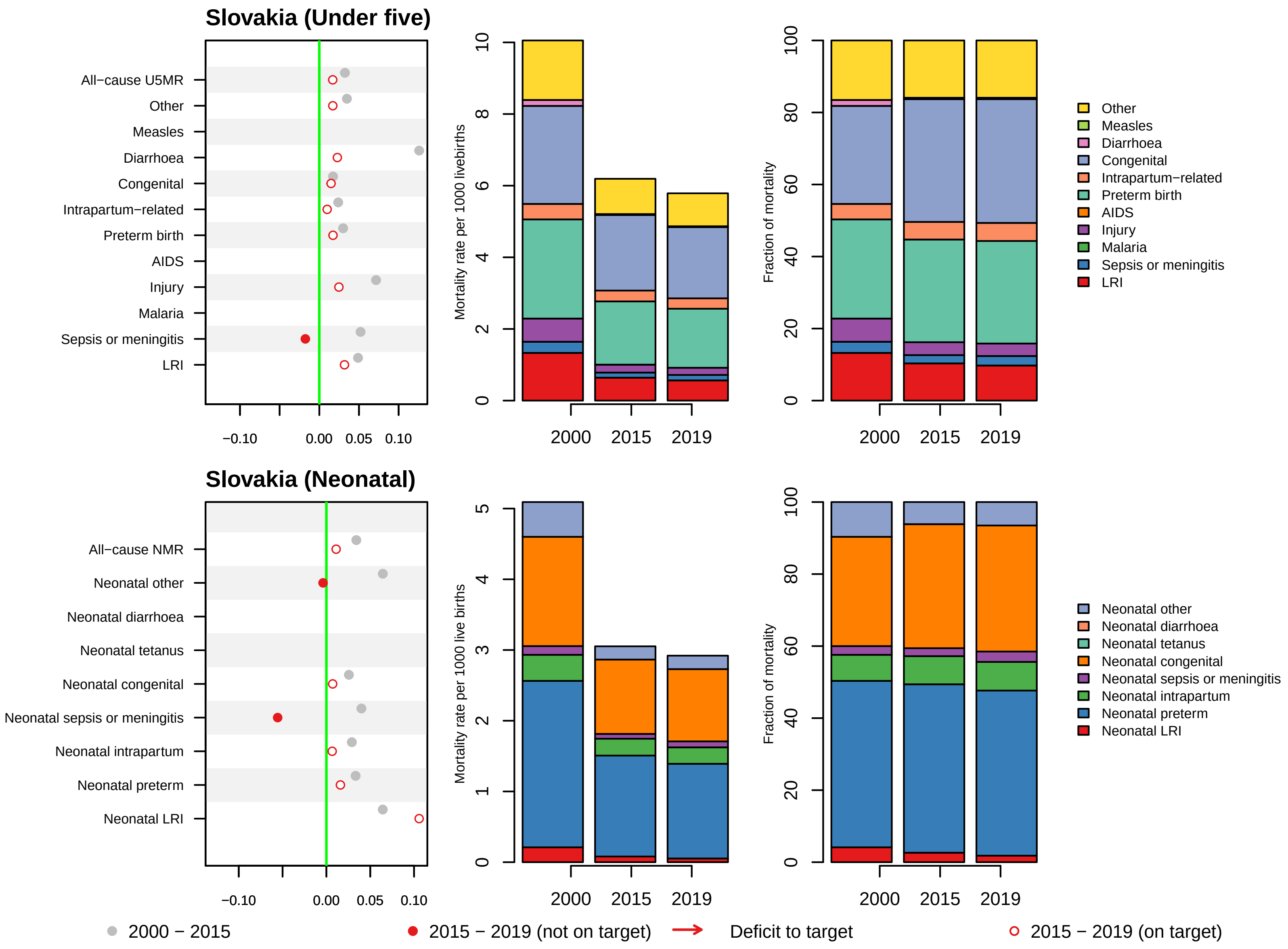

Slovakia (Under five)
All−cause U5MR
Other
Measles
Diarrhoea
Congenital
Intrapartum−related
Preterm birth
AIDS
Injury
Malaria
Sepsis or meningitis
LRI
−0.10
0.00
0.05
0.10
Mortality rate per 1000 livebirths
Fraction of mortality
2000
2015
2019
Other
Measles
Diarrhoea
Congenital
Intrapartum−related
Preterm birth
AIDS
Injury
Malaria
Sepsis or meningitis
LRI
Slovakia (Neonatal)
All−cause NMR
Neonatal other
Neonatal diarrhoea
Neonatal tetanus
Neonatal congenital
Neonatal sepsis or meningitis
Neonatal intrapartum
Neonatal preterm
Neonatal LRI
Mortality rate per 1000 live births
Fraction of mortality
2000
2015
2019
2000 − 2015
2015 − 2019 (not on target)
Deficit to target
2015 − 2019 (on target)

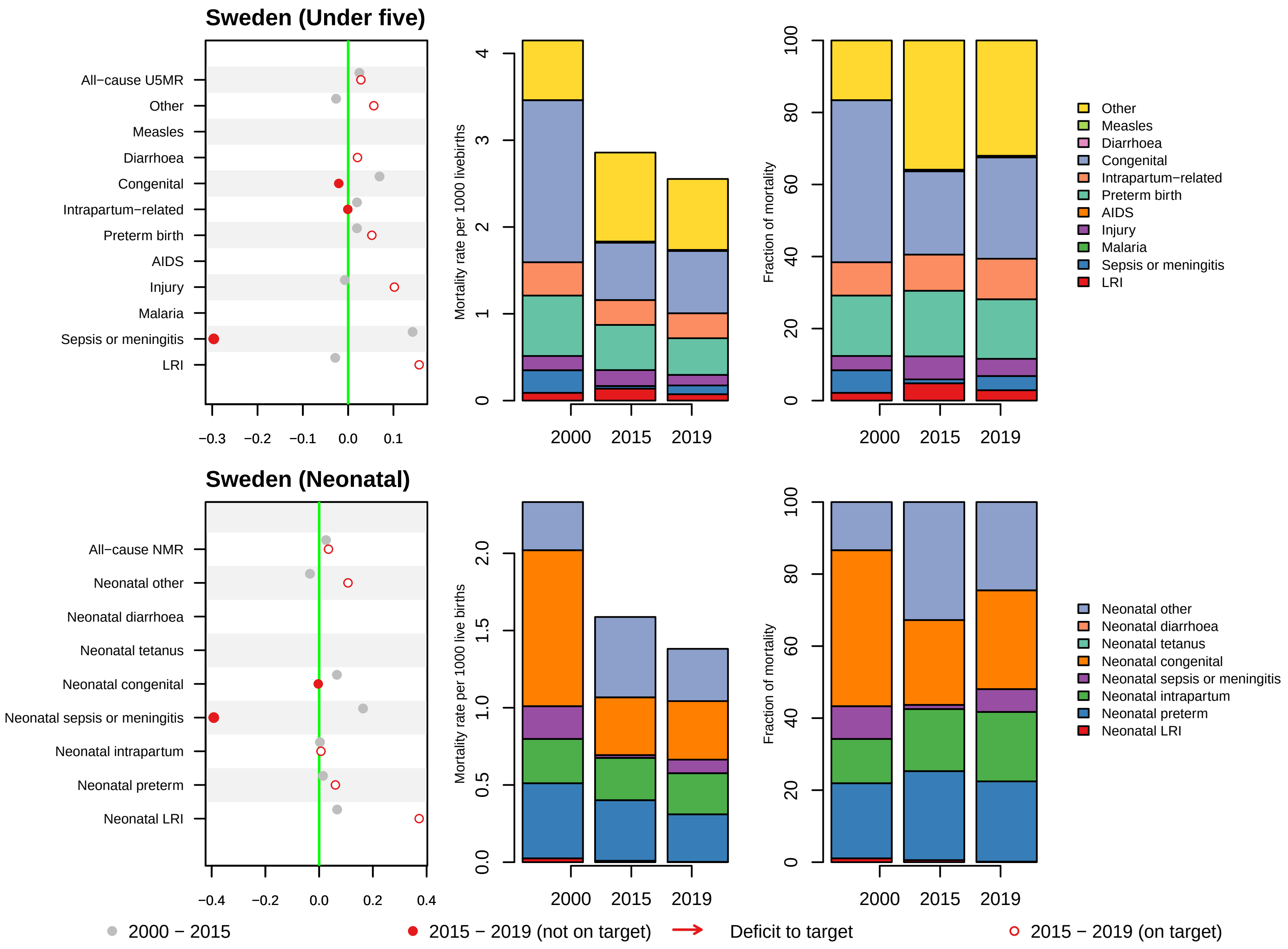

Sweden (Under five)
All−cause U5MR
Other
Measles
Diarrhoea
Congenital
Intrapartum−related
Preterm birth
AIDS
Injury
Malaria
Sepsis or meningitis
LRI
−0.3 −0.2 −0.1 0.0 0.1
Mortality rate per 1000 livebirths
0 1 2 3 4
2000 2015 2019
Fraction of mortality
0 20 40 60 80 100
2000 2015 2019
Other
Measles
Diarrhoea
Congenital
Intrapartum−related
Preterm birth
AIDS
Injury
Malaria
Sepsis or meningitis
LRI
Sweden (Neonatal)
All−cause NMR
Neonatal other
Neonatal diarrhoea
Neonatal tetanus
Neonatal congenital
Neonatal sepsis or meningitis
Neonatal intrapartum
Neonatal preterm
Neonatal LRI
−0.4 −0.2 0.0 0.2 0.4
Mortality rate per 1000 live births
0.0 0.5 1.0 1.5 2.0
2000 2015 2019
Fraction of mortality
0 20 40 60 80 100
2000 2015 2019
Neonatal other
Neonatal diarrhoea
Neonatal tetanus
Neonatal congenital
Neonatal sepsis or meningitis
Neonatal intrapartum
Neonatal preterm
Neonatal LRI
2000 − 2015
2015 − 2019 (not on target)
Deficit to target
2015 − 2019 (on target)

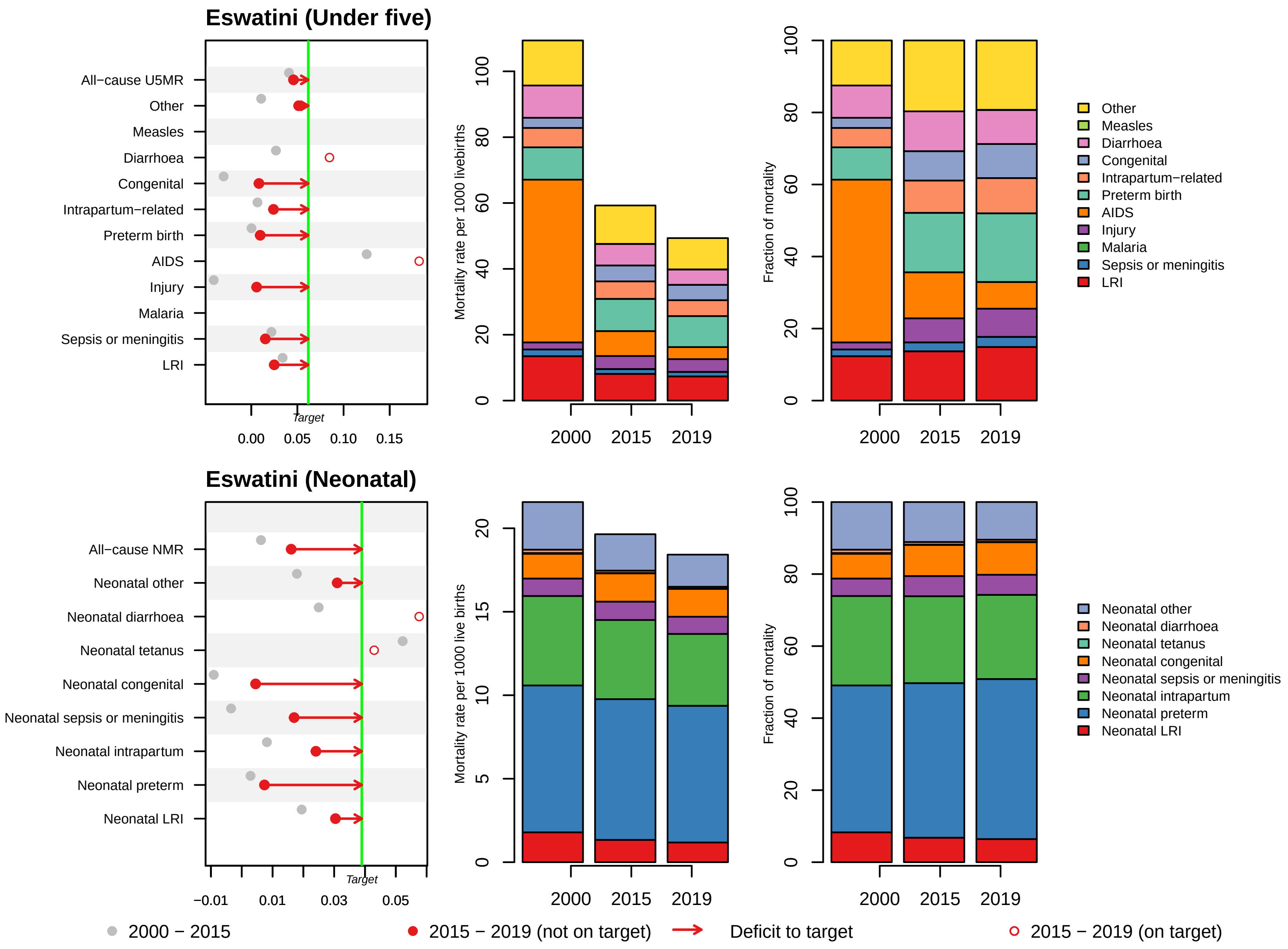

Eswatini (Under five)
All−cause U5MR
Other
Measles
Diarrhoea
Congenital
Intrapartum−related
Preterm birth
AIDS
Injury
Malaria
Sepsis or meningitis
LRI
Target
0.00
0.05
0.10
0.15
Mortality rate per 1000 livebirths
Fraction of mortality
2000
2015
2019
Eswatini (Neonatal)
All−cause NMR
Neonatal other
Neonatal diarrhoea
Neonatal tetanus
Neonatal congenital
Neonatal sepsis or meningitis
Neonatal intrapartum
Neonatal preterm
Neonatal LRI
−0.01
0.01
0.03
0.05
Mortality rate per 1000 live births
Neonatal LRI
2000 − 2015
2015 − 2019 (not on target)
Deficit to target
2015 − 2019 (on target)

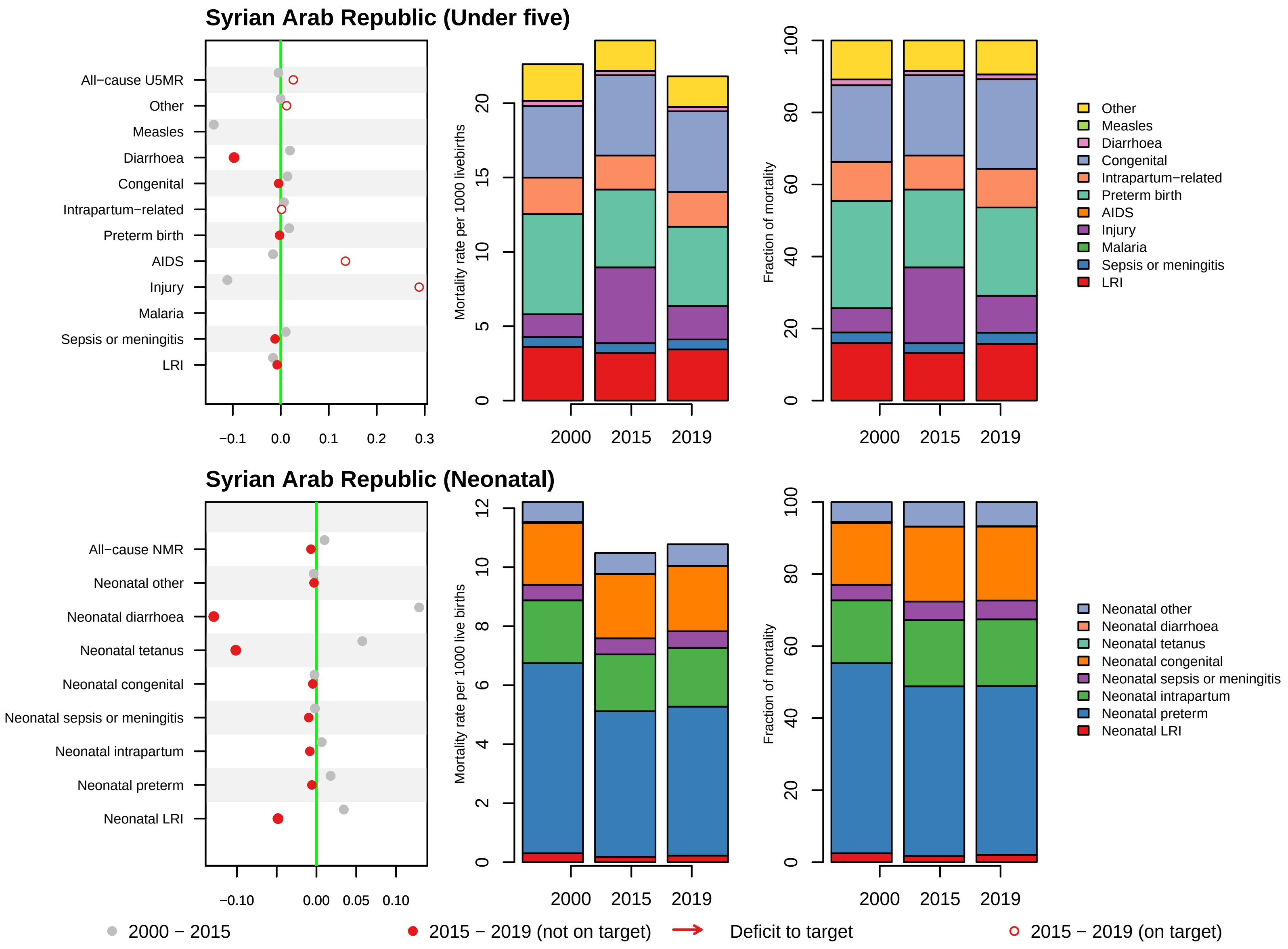

Syrian Arab Republic (Under five)
All−cause U5MR
Other
Measles
Diarrhoea
Congenital
Intrapartum−related
Preterm birth
AIDS
Injury
Malaria
Sepsis or meningitis
LRI
−0.1
0.0
0.1
0.2
0.3
Mortality rate per 1000 livebirths
0
5
10
15
20
2000
2015
2019
Fraction of mortality
0
20
40
60
80
100
Other
Measles
Diarrhoea
Congenital
Intrapartum−related
Preterm birth
AIDS
Injury
Malaria
Sepsis or meningitis
LRI
Syrian Arab Republic (Neonatal)
All−cause NMR
Neonatal other
Neonatal diarrhoea
Neonatal tetanus
Neonatal congenital
Neonatal sepsis or meningitis
Neonatal intrapartum
Neonatal preterm
Neonatal LRI
−0.10
0.00
0.05
0.10
Mortality rate per 1000 live births
0
2
4
6
8
10
12
Neonatal other
Neonatal diarrhoea
Neonatal tetanus
Neonatal congenital
Neonatal sepsis or meningitis
Neonatal intrapartum
Neonatal preterm
Neonatal LRI
2000 − 2015
2015 − 2019 (not on target)
Deficit to target
2015 − 2019 (on target)

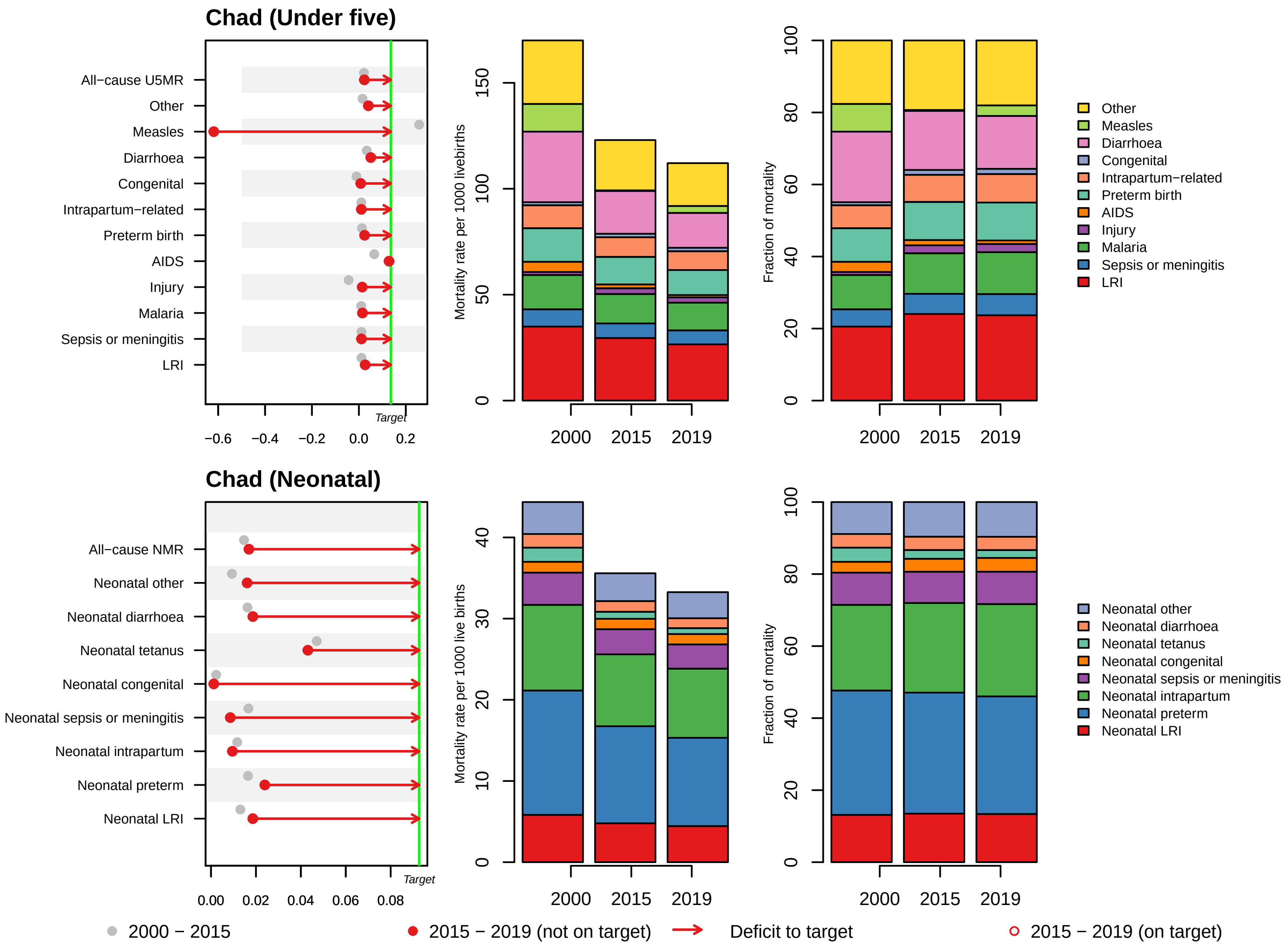
Chad (Under five)
All−cause U5MR
Other
Measles
Diarrhoea
Congenital
Intrapartum−related
Preterm birth
AIDS
Injury
Malaria
Sepsis or meningitis
LRI
−0.6
−0.4
−0.2
0.0
0.2
Target
Mortality rate per 1000 livebirths
0
50
100
150
2000
2015
2019
Fraction of mortality
0
20
40
60
80
100
Other
Measles
Diarrhoea
Congenital
Intrapartum−related
Preterm birth
AIDS
Injury
Malaria
Sepsis or meningitis
LRI
Chad (Neonatal)
All−cause NMR
Neonatal other
Neonatal diarrhoea
Neonatal tetanus
Neonatal congenital
Neonatal sepsis or meningitis
Neonatal intrapartum
Neonatal preterm
Neonatal LRI
0.00
0.02
0.04
0.06
0.08
Target
Mortality rate per 1000 live births
0
10
20
30
40
Neonatal other
Neonatal diarrhoea
Neonatal tetanus
Neonatal congenital
Neonatal sepsis or meningitis
Neonatal intrapartum
Neonatal preterm
Neonatal LRI
2000 − 2015
2015 − 2019 (not on target)
Deficit to target
2015 − 2019 (on target)

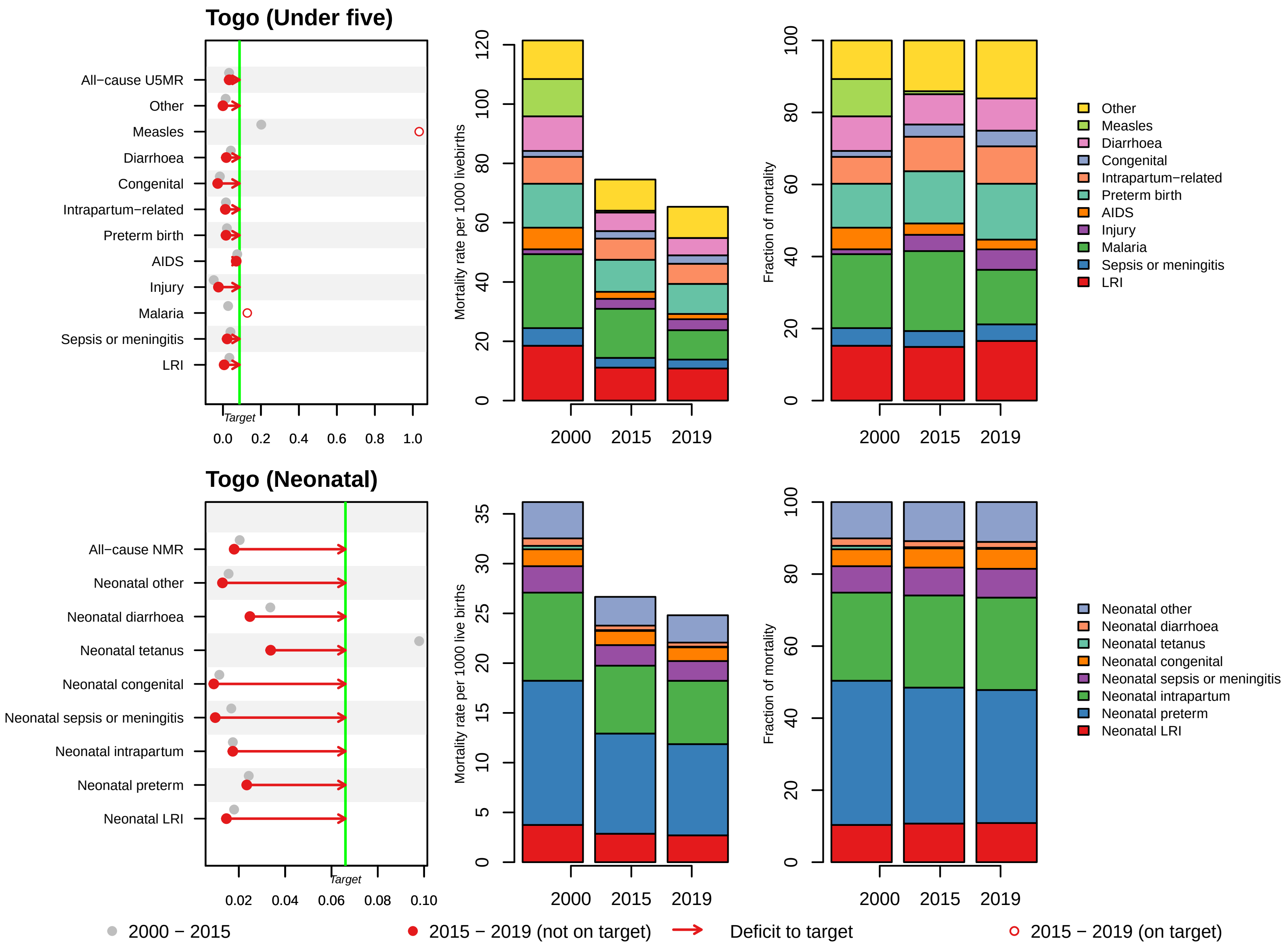

Togo (Under five)
All−cause U5MR
Other
Measles
Diarrhoea
Congenital
Intrapartum−related
Preterm birth
AIDS
Injury
Malaria
Sepsis or meningitis
LRI
Target
0.0
0.2
0.4
0.6
0.8
1.0
Mortality rate per 1000 livebirths
0
20
40
60
80
100
120
2000
2015
2019
Fraction of mortality
Other
Measles
Diarrhoea
Congenital
Intrapartum−related
Preterm birth
AIDS
Injury
Malaria
Sepsis or meningitis
LRI
Togo (Neonatal)
All−cause NMR
Neonatal other
Neonatal diarrhoea
Neonatal tetanus
Neonatal congenital
Neonatal sepsis or meningitis
Neonatal intrapartum
Neonatal preterm
Neonatal LRI
0.02
0.04
0.06
0.08
0.10
Mortality rate per 1000 live births
0
5
10
15
20
25
30
35
Neonatal other
Neonatal diarrhoea
Neonatal tetanus
Neonatal congenital
Neonatal sepsis or meningitis
Neonatal intrapartum
Neonatal preterm
Neonatal LRI
2000 − 2015
2015 − 2019 (not on target)
Deficit to target
2015 − 2019 (on target)

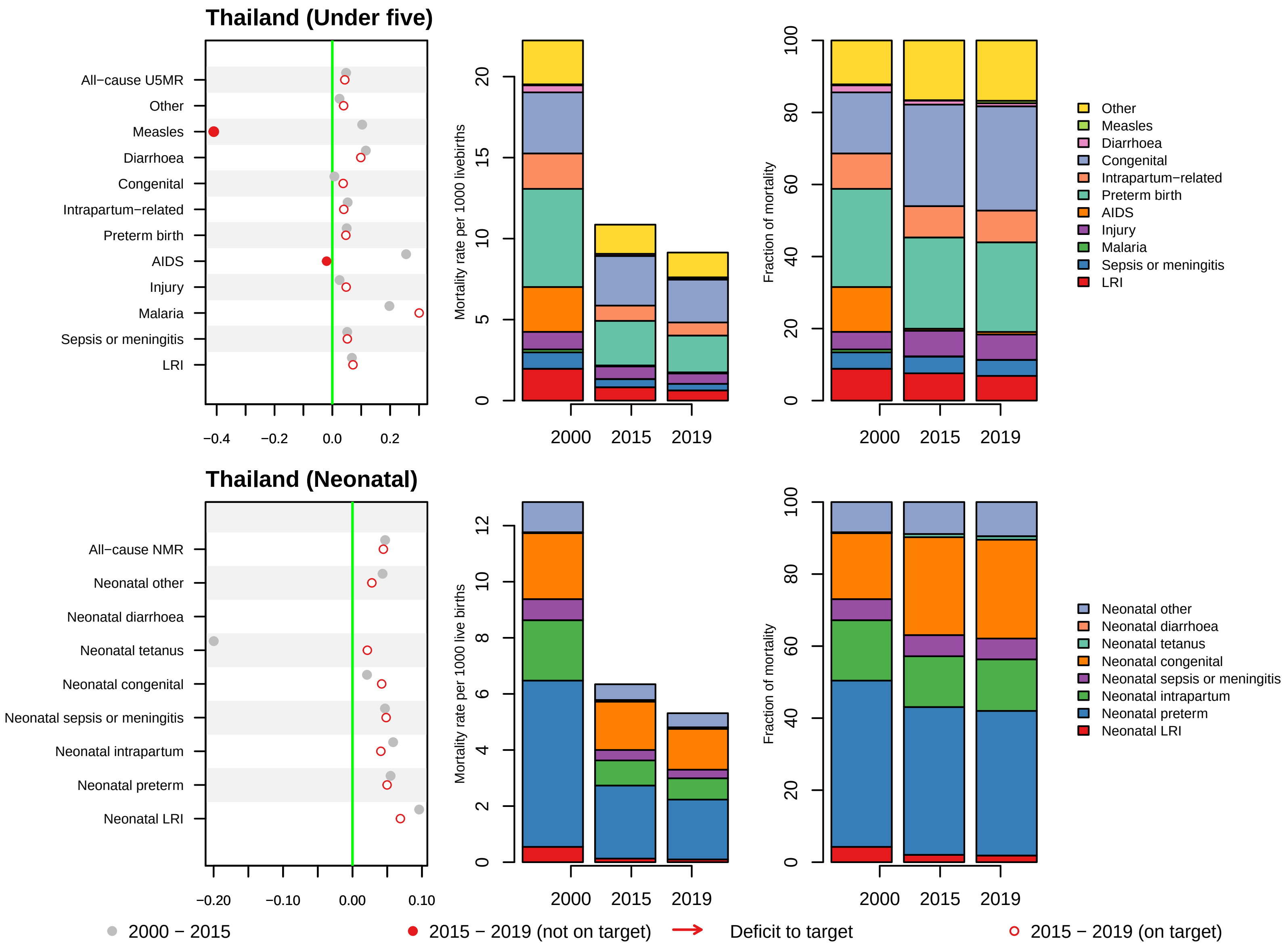

Thailand (Under five)
All−cause U5MR
Other
Measles
Diarrhoea
Congenital
Intrapartum−related
Preterm birth
AIDS
Injury
Malaria
Sepsis or meningitis
LRI
Mortality rate per 1000 livebirths
Fraction of mortality
Other
Measles
Diarrhoea
Congenital
Intrapartum−related
Preterm birth
AIDS
Injury
Malaria
Sepsis or meningitis
LRI
Thailand (Neonatal)
All−cause NMR
Neonatal other
Neonatal diarrhoea
Neonatal tetanus
Neonatal congenital
Neonatal sepsis or meningitis
Neonatal intrapartum
Neonatal preterm
Neonatal LRI
Mortality rate per 1000 live births
Fraction of mortality
Neonatal other
Neonatal diarrhoea
Neonatal tetanus
Neonatal congenital
Neonatal sepsis or meningitis
Neonatal intrapartum
Neonatal preterm
Neonatal LRI
2000 − 2015
2015 − 2019 (not on target)
Deficit to target
2015 − 2019 (on target)

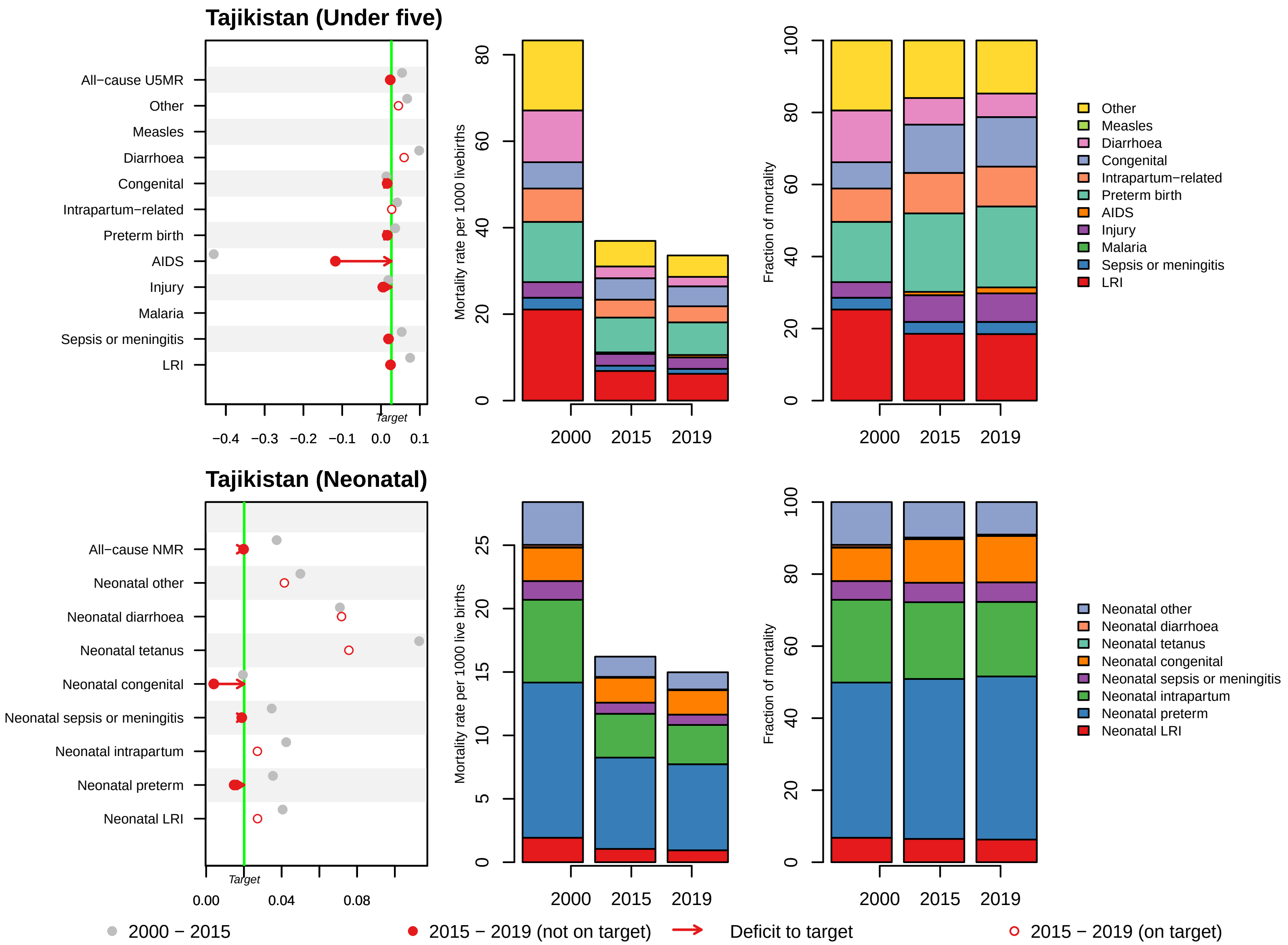

Tajikistan (Under five)
All−cause U5MR
Other
Measles
Diarrhoea
Congenital
Intrapartum−related
Preterm birth
AIDS
Injury
Malaria
Sepsis or meningitis
LRI
Target
−0.4 −0.3 −0.2 −0.1 0.0 0.1
Mortality rate per 1000 livebirths
Fraction of mortality
2000 2015 2019
Other
Measles
Diarrhoea
Congenital
Intrapartum−related
Preterm birth
AIDS
Injury
Malaria
Sepsis or meningitis
LRI
Tajikistan (Neonatal)
All−cause NMR
Neonatal other
Neonatal diarrhoea
Neonatal tetanus
Neonatal congenital
Neonatal sepsis or meningitis
Neonatal intrapartum
Neonatal preterm
Neonatal LRI
Target
0.00 0.04 0.08
Mortality rate per 1000 live births
Fraction of mortality
Neonatal other
Neonatal diarrhoea
Neonatal tetanus
Neonatal congenital
Neonatal sepsis or meningitis
Neonatal intrapartum
Neonatal preterm
Neonatal LRI
2000 − 2015
2015 − 2019 (not on target)
Deficit to target
2015 − 2019 (on target)

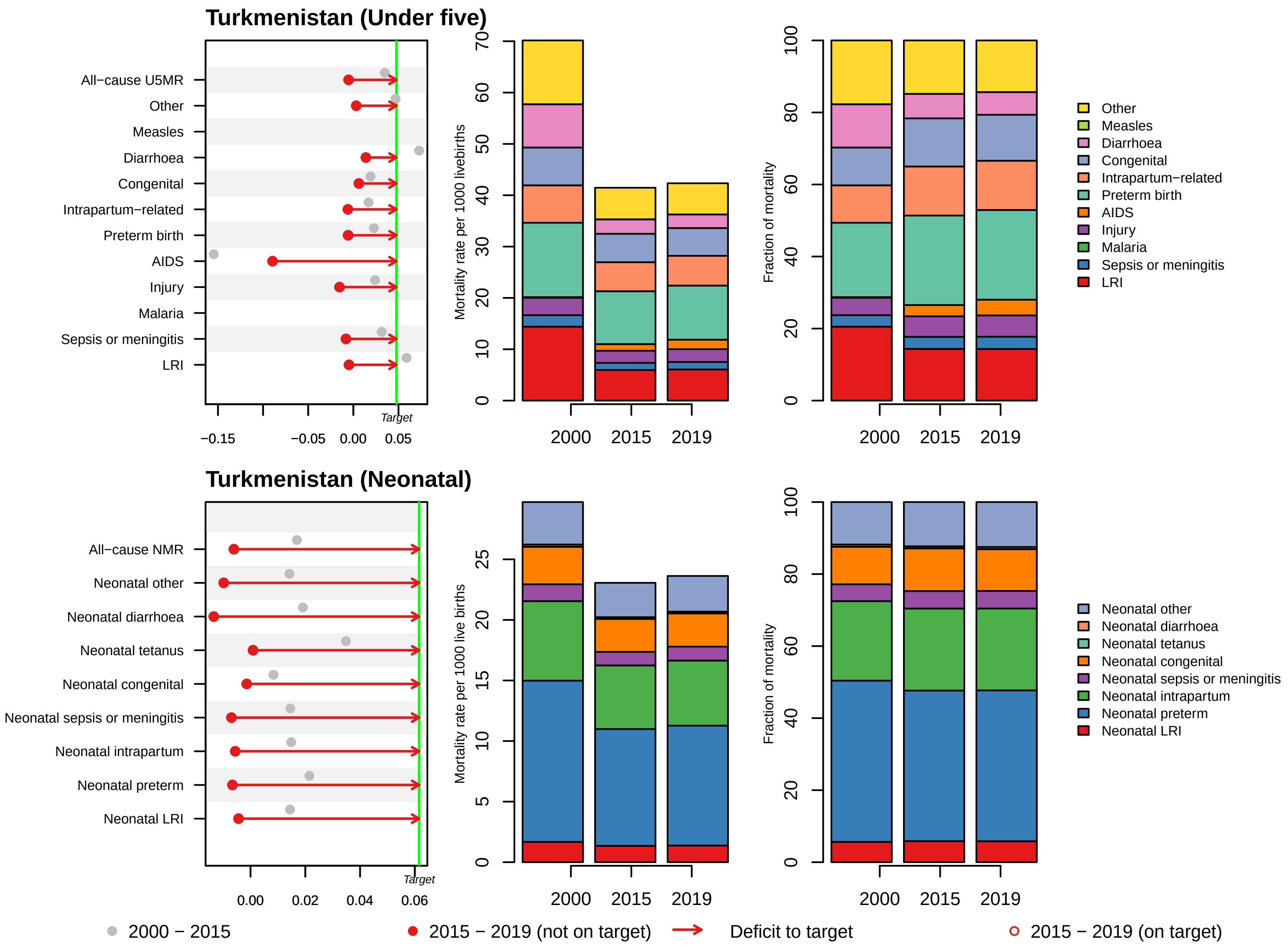
Turkmenistan (Under five)
All−cause U5MR
Other
Measles
Diarrhoea
Congenital
Intrapartum−related
Preterm birth
AIDS
Injury
Malaria
Sepsis or meningitis
LRI
Target
−0.15
−0.05
0.00
0.05
Mortality rate per 1000 livebirths
0
10
20
30
40
50
60
70
2000
2015
2019
Fraction of mortality
0
20
40
60
80
100
Other
Measles
Diarrhoea
Congenital
Intrapartum−related
Preterm birth
AIDS
Injury
Malaria
Sepsis or meningitis
LRI
Turkmenistan (Neonatal)
All−cause NMR
Neonatal other
Neonatal diarrhoea
Neonatal tetanus
Neonatal congenital
Neonatal sepsis or meningitis
Neonatal intrapartum
Neonatal preterm
Neonatal LRI
Target
0.00
0.02
0.04
0.06
Mortality rate per 1000 live births
0
5
10
15
20
25
Neonatal other
Neonatal diarrhoea
Neonatal tetanus
Neonatal congenital
Neonatal sepsis or meningitis
Neonatal intrapartum
Neonatal preterm
Neonatal LRI
2000 − 2015
2015 − 2019 (not on target)
Deficit to target
2015 − 2019 (on target)

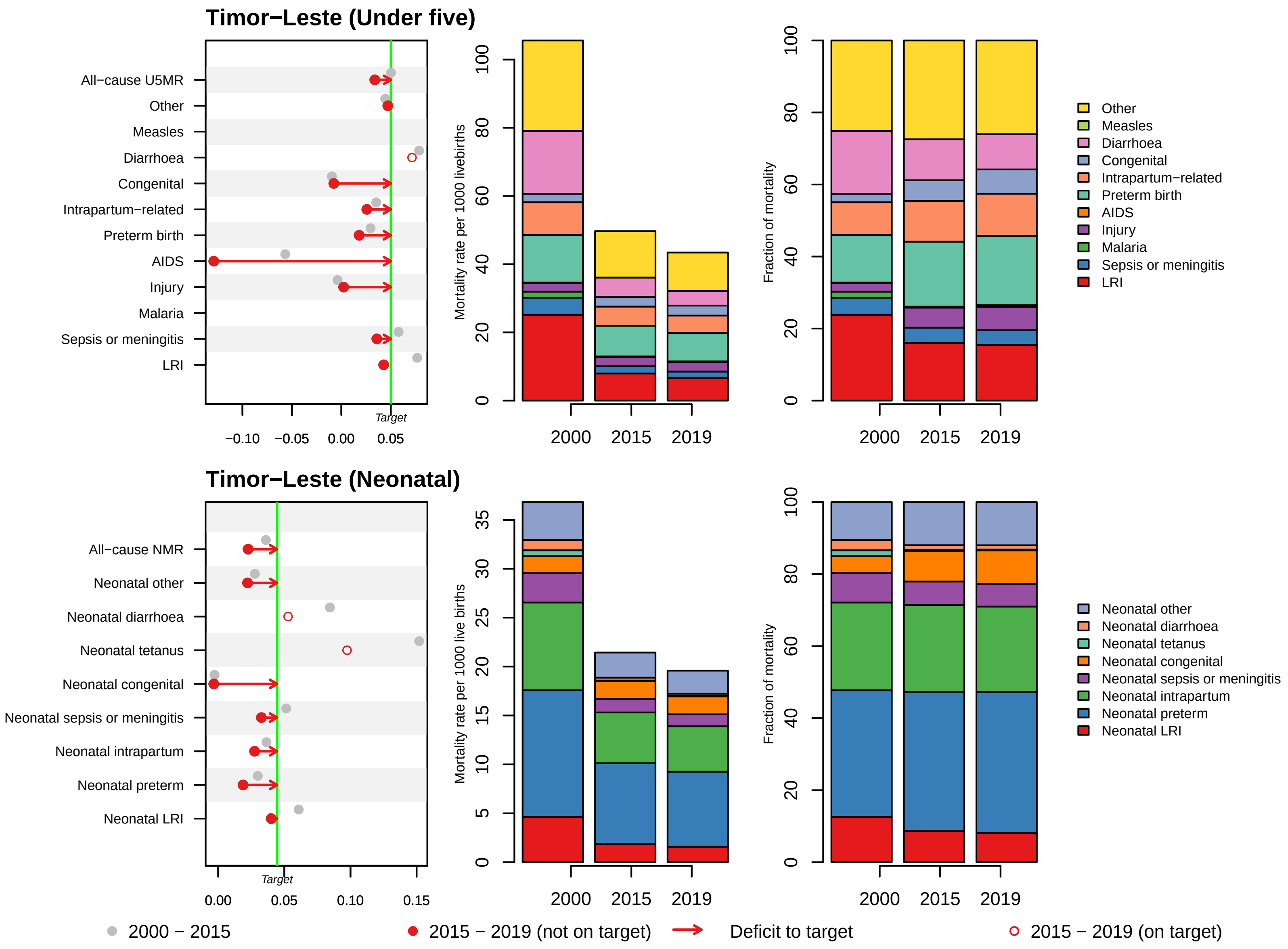

Timor−Leste (Under five)
All−cause U5MR
Other
Measles
Diarrhoea
Congenital
Intrapartum−related
Preterm birth
AIDS
Injury
Malaria
Sepsis or meningitis
LRI
Target
−0.10
−0.05
0.00
0.05
Mortality rate per 1000 livebirths
0
20
40
60
80
100
2000
2015
2019
Fraction of mortality
0
20
40
60
80
100
2000
2015
2019
Other
Measles
Diarrhoea
Congenital
Intrapartum−related
Preterm birth
AIDS
Injury
Malaria
Sepsis or meningitis
LRI
Timor−Leste (Neonatal)
All−cause NMR
Neonatal other
Neonatal diarrhoea
Neonatal tetanus
Neonatal congenital
Neonatal sepsis or meningitis
Neonatal intrapartum
Neonatal preterm
Neonatal LRI
Target
0.00
0.05
0.10
0.15
Mortality rate per 1000 live births
0
5
10
15
20
25
30
35
2000
2015
2019
Fraction of mortality
0
20
40
60
80
100
2000
2015
2019
Neonatal other
Neonatal diarrhoea
Neonatal tetanus
Neonatal congenital
Neonatal sepsis or meningitis
Neonatal intrapartum
Neonatal preterm
Neonatal LRI
2000 − 2015
2015 − 2019 (not on target)
Deficit to target
2015 − 2019 (on target)

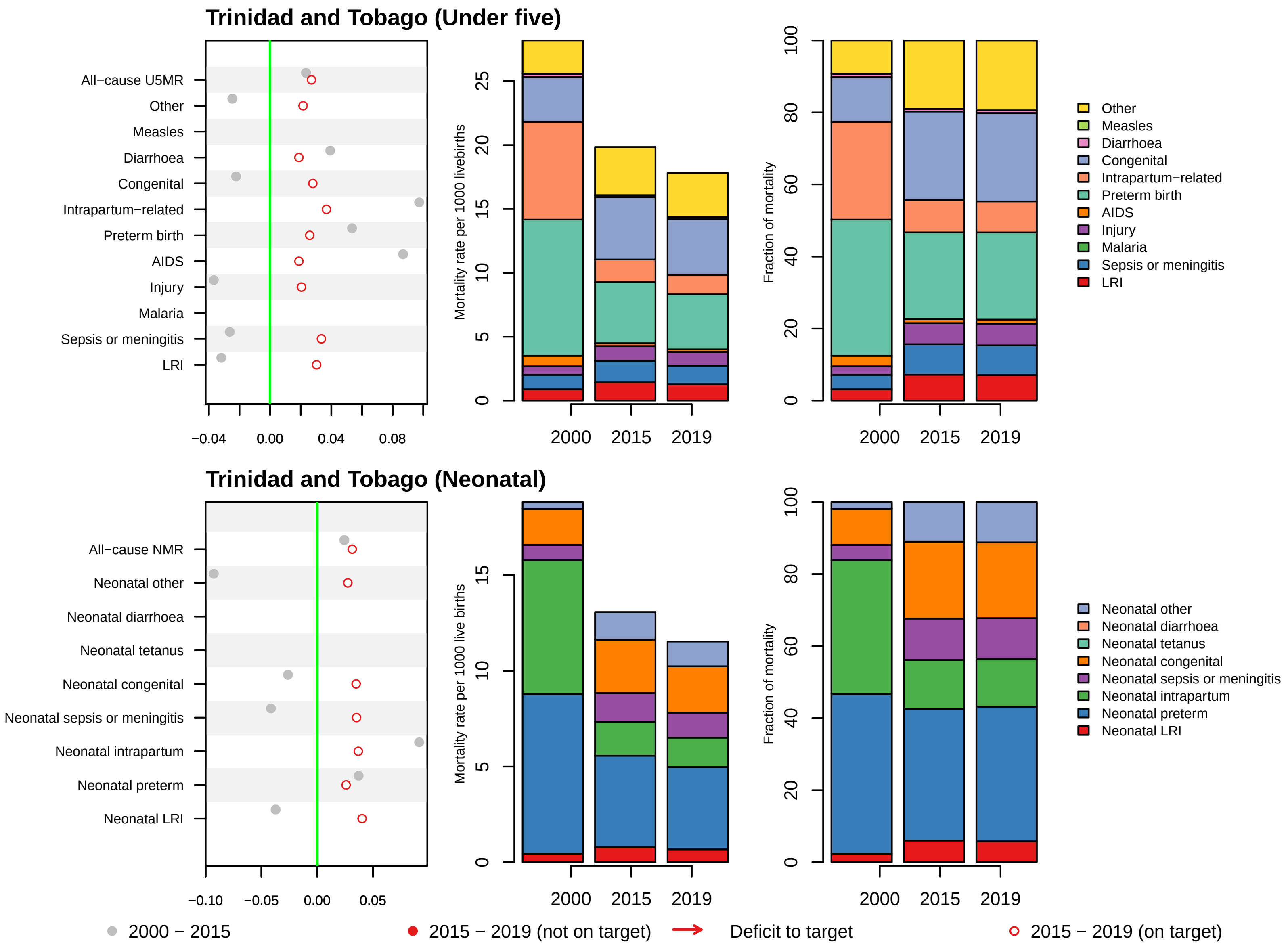

Trinidad and Tobago (Under five)
All−cause U5MR
Other
Measles
Diarrhoea
Congenital
Intrapartum−related
Preterm birth
AIDS
Injury
Malaria
Sepsis or meningitis
LRI
−0.04
0.00
0.04
0.08
Mortality rate per 1000 livebirths
0
5
10
15
20
25
2000
2015
2019
Fraction of mortality
0
20
40
60
80
100
Other
Measles
Diarrhoea
Congenital
Intrapartum−related
Preterm birth
AIDS
Injury
Malaria
Sepsis or meningitis
LRI
Trinidad and Tobago (Neonatal)
All−cause NMR
Neonatal other
Neonatal diarrhoea
Neonatal tetanus
Neonatal congenital
Neonatal sepsis or meningitis
Neonatal intrapartum
Neonatal preterm
Neonatal LRI
−0.10
−0.05
0.00
0.05
Mortality rate per 1000 live births
0
5
10
15
2000
2015
2019
Fraction of mortality
0
20
40
60
80
100
Neonatal other
Neonatal diarrhoea
Neonatal tetanus
Neonatal congenital
Neonatal sepsis or meningitis
Neonatal intrapartum
Neonatal preterm
Neonatal LRI
2000 − 2015
2015 − 2019 (not on target)
Deficit to target
2015 − 2019 (on target)

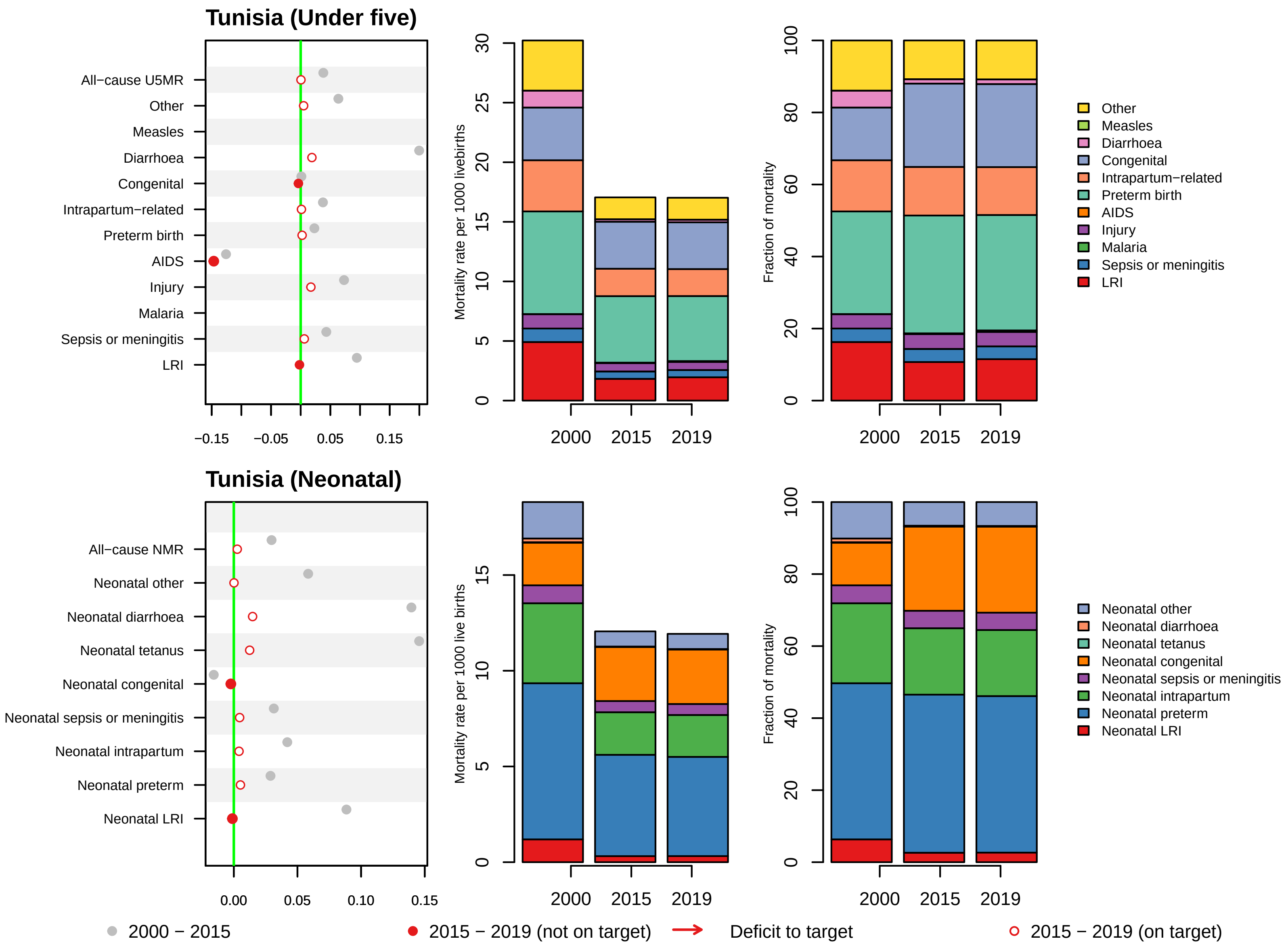

Tunisia (Under five)
All−cause U5MR
Other
Measles
Diarrhoea
Congenital
Intrapartum−related
Preterm birth
AIDS
Injury
Malaria
Sepsis or meningitis
LRI
−0.15
−0.05
0.05
0.15
Mortality rate per 1000 livebirths
Fraction of mortality
2000
2015
2019
Other
Measles
Diarrhoea
Congenital
Intrapartum−related
Preterm birth
AIDS
Injury
Malaria
Sepsis or meningitis
LRI
Tunisia (Neonatal)
All−cause NMR
Neonatal other
Neonatal diarrhoea
Neonatal tetanus
Neonatal congenital
Neonatal sepsis or meningitis
Neonatal intrapartum
Neonatal preterm
Neonatal LRI
0.00
0.05
0.10
0.15
Mortality rate per 1000 live births
Fraction of mortality
Neonatal other
Neonatal diarrhoea
Neonatal tetanus
Neonatal congenital
Neonatal sepsis or meningitis
Neonatal intrapartum
Neonatal preterm
Neonatal LRI
2000 − 2015
2015 − 2019 (not on target)
Deficit to target
2015 − 2019 (on target)

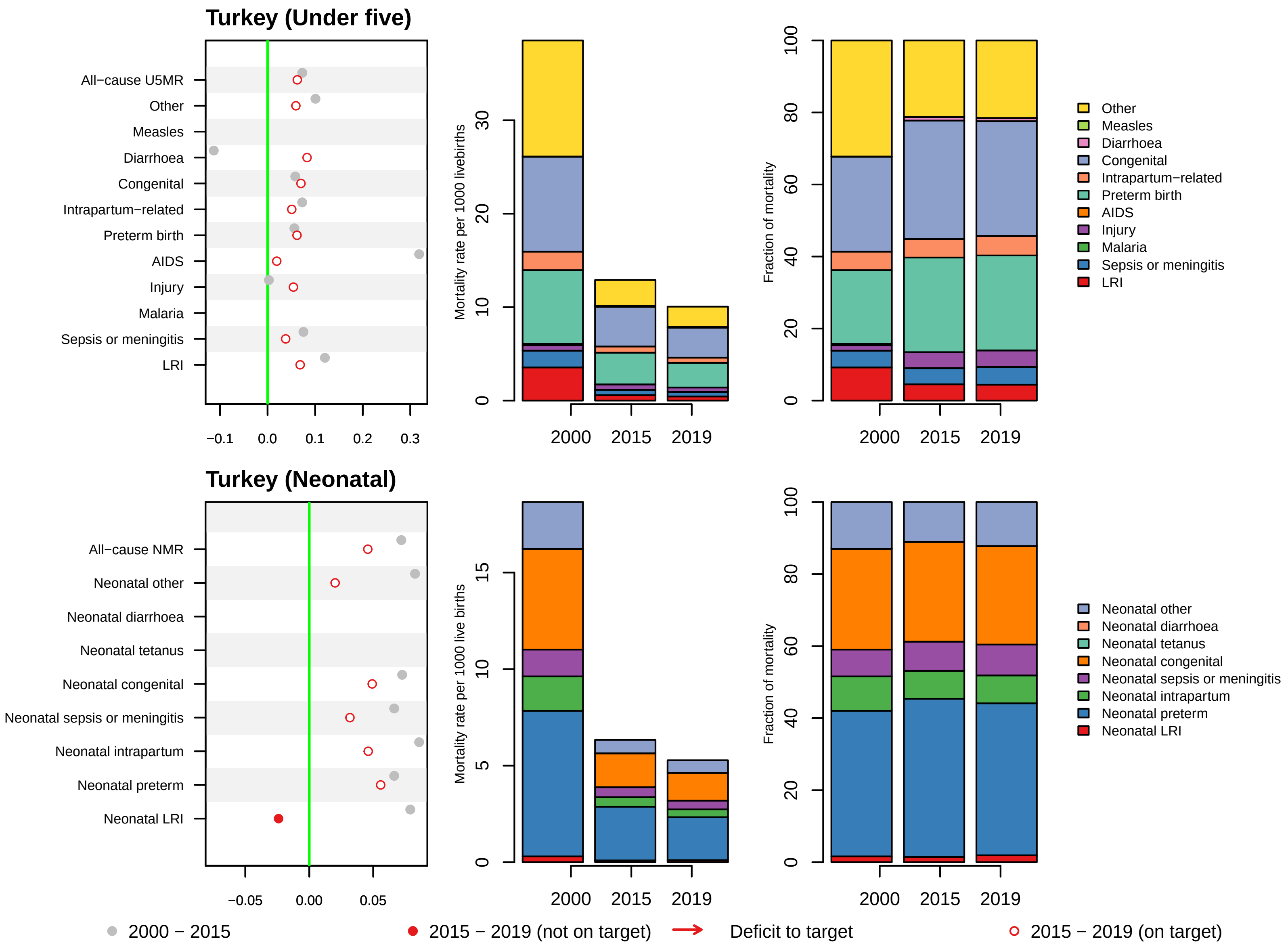

Turkey (Under five)
All−cause U5MR
Other
Measles
Diarrhoea
Congenital
Intrapartum−related
Preterm birth
AIDS
Injury
Malaria
Sepsis or meningitis
LRI
−0.1
0.0
0.1
0.2
0.3
Mortality rate per 1000 livebirths
Fraction of mortality
2000
2015
2019
Other
Measles
Diarrhoea
Congenital
Intrapartum−related
Preterm birth
AIDS
Injury
Malaria
Sepsis or meningitis
LRI
Turkey (Neonatal)
All−cause NMR
Neonatal other
Neonatal diarrhoea
Neonatal tetanus
Neonatal congenital
Neonatal sepsis or meningitis
Neonatal intrapartum
Neonatal preterm
Neonatal LRI
−0.05
0.00
0.05
Mortality rate per 1000 live births
Neonatal other
Neonatal diarrhoea
Neonatal tetanus
Neonatal congenital
Neonatal sepsis or meningitis
Neonatal intrapartum
Neonatal preterm
Neonatal LRI
2000 − 2015
2015 − 2019 (not on target)
Deficit to target
2015 − 2019 (on target)

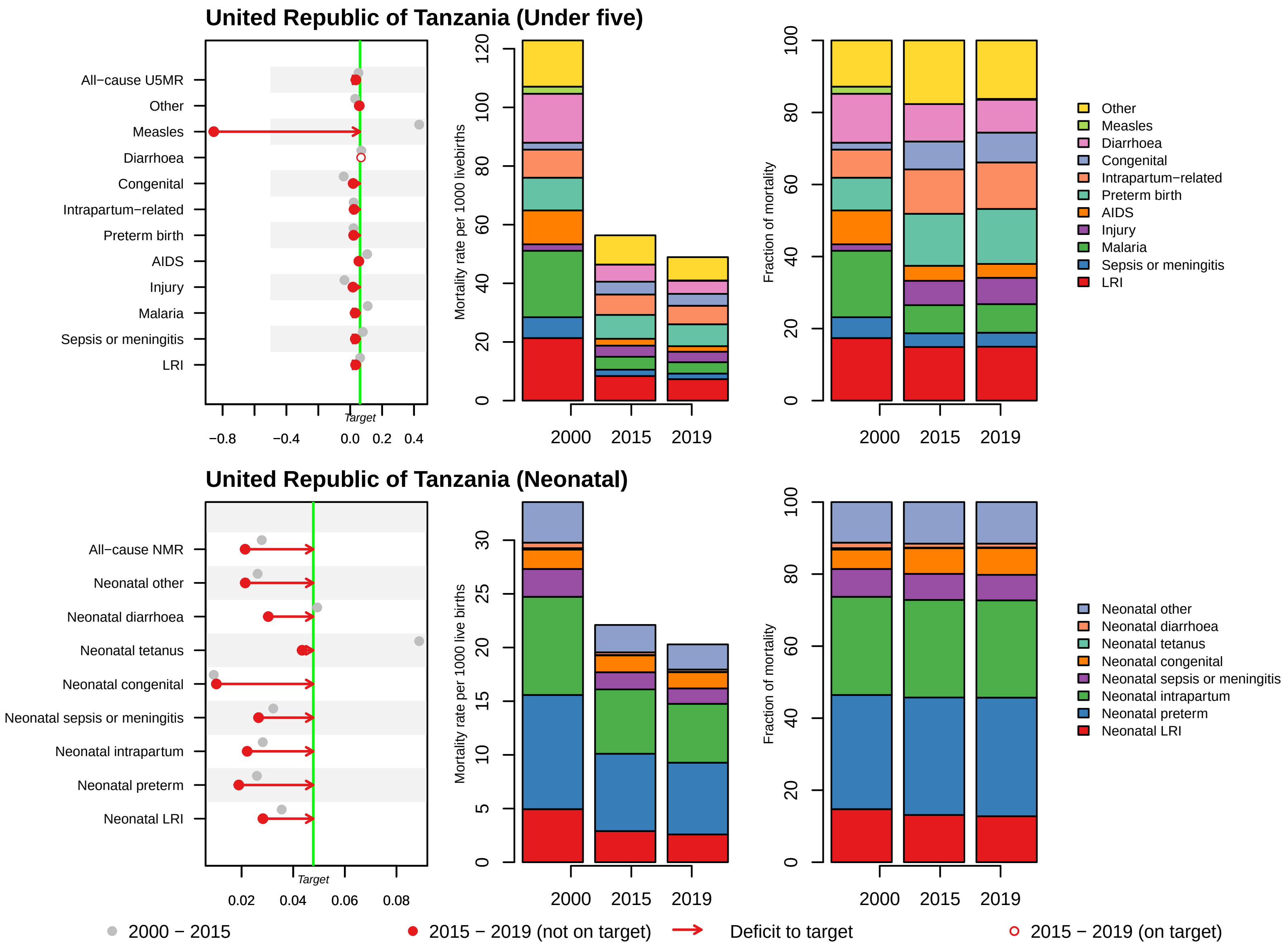

United Republic of Tanzania (Under five)
All−cause U5MR
Other
Measles
Diarrhoea
Congenital
Intrapartum−related
Preterm birth
AIDS
Injury
Malaria
Sepsis or meningitis
LRI
Target
−0.8
−0.4
0.0
0.2
0.4
Mortality rate per 1000 livebirths
0
20
40
60
80
100
120
2000
2015
2019
Fraction of mortality
0
20
40
60
80
100
2000
2015
2019
Other
Measles
Diarrhoea
Congenital
Intrapartum−related
Preterm birth
AIDS
Injury
Malaria
Sepsis or meningitis
LRI
United Republic of Tanzania (Neonatal)
All−cause NMR
Neonatal other
Neonatal diarrhoea
Neonatal tetanus
Neonatal congenital
Neonatal sepsis or meningitis
Neonatal intrapartum
Neonatal preterm
Neonatal LRI
Target
0.02
0.04
0.06
0.08
Mortality rate per 1000 live births
0
5
10
15
20
25
30
2000
2015
2019
Fraction of mortality
0
20
40
60
80
100
2000
2015
2019
Neonatal other
Neonatal diarrhoea
Neonatal tetanus
Neonatal congenital
Neonatal sepsis or meningitis
Neonatal intrapartum
Neonatal preterm
Neonatal LRI
2000 − 2015
2015 − 2019 (not on target)
Deficit to target
2015 − 2019 (on target)

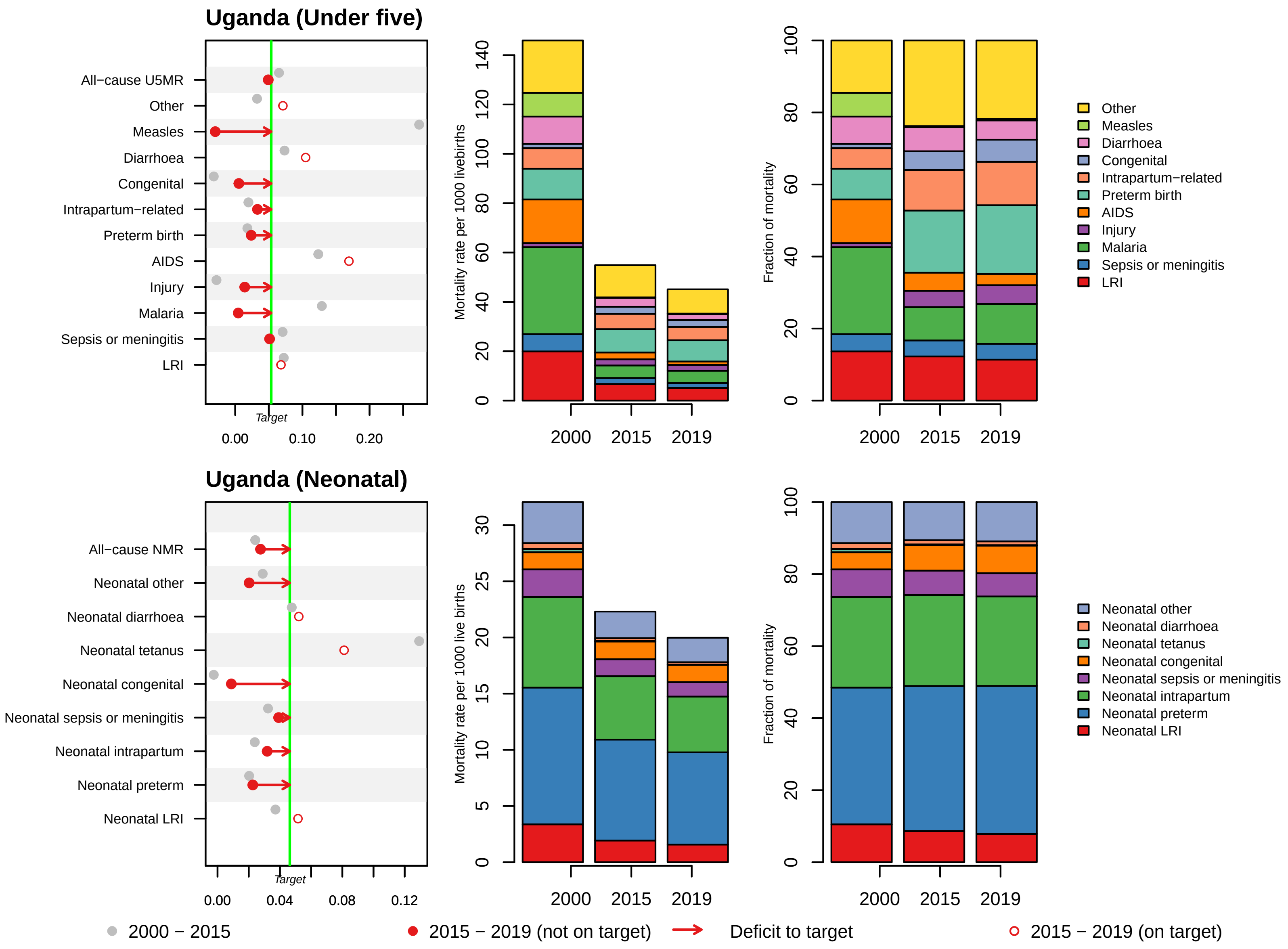

Uganda (Under five)
All−cause U5MR
Other
Measles
Diarrhoea
Congenital
Intrapartum−related
Preterm birth
AIDS
Injury
Malaria
Sepsis or meningitis
LRI
Target
0.00
0.10
0.20
Mortality rate per 1000 livebirths
Fraction of mortality
2000
2015
2019
Other
Measles
Diarrhoea
Congenital
Intrapartum−related
Preterm birth
AIDS
Injury
Malaria
Sepsis or meningitis
LRI
Uganda (Neonatal)
All−cause NMR
Neonatal other
Neonatal diarrhoea
Neonatal tetanus
Neonatal congenital
Neonatal sepsis or meningitis
Neonatal intrapartum
Neonatal preterm
Neonatal LRI
0.00
0.04
0.08
0.12
Mortality rate per 1000 live births
Neonatal other
Neonatal diarrhoea
Neonatal tetanus
Neonatal congenital
Neonatal sepsis or meningitis
Neonatal intrapartum
Neonatal preterm
Neonatal LRI
2000 − 2015
2015 − 2019 (not on target)
Deficit to target
2015 − 2019 (on target)

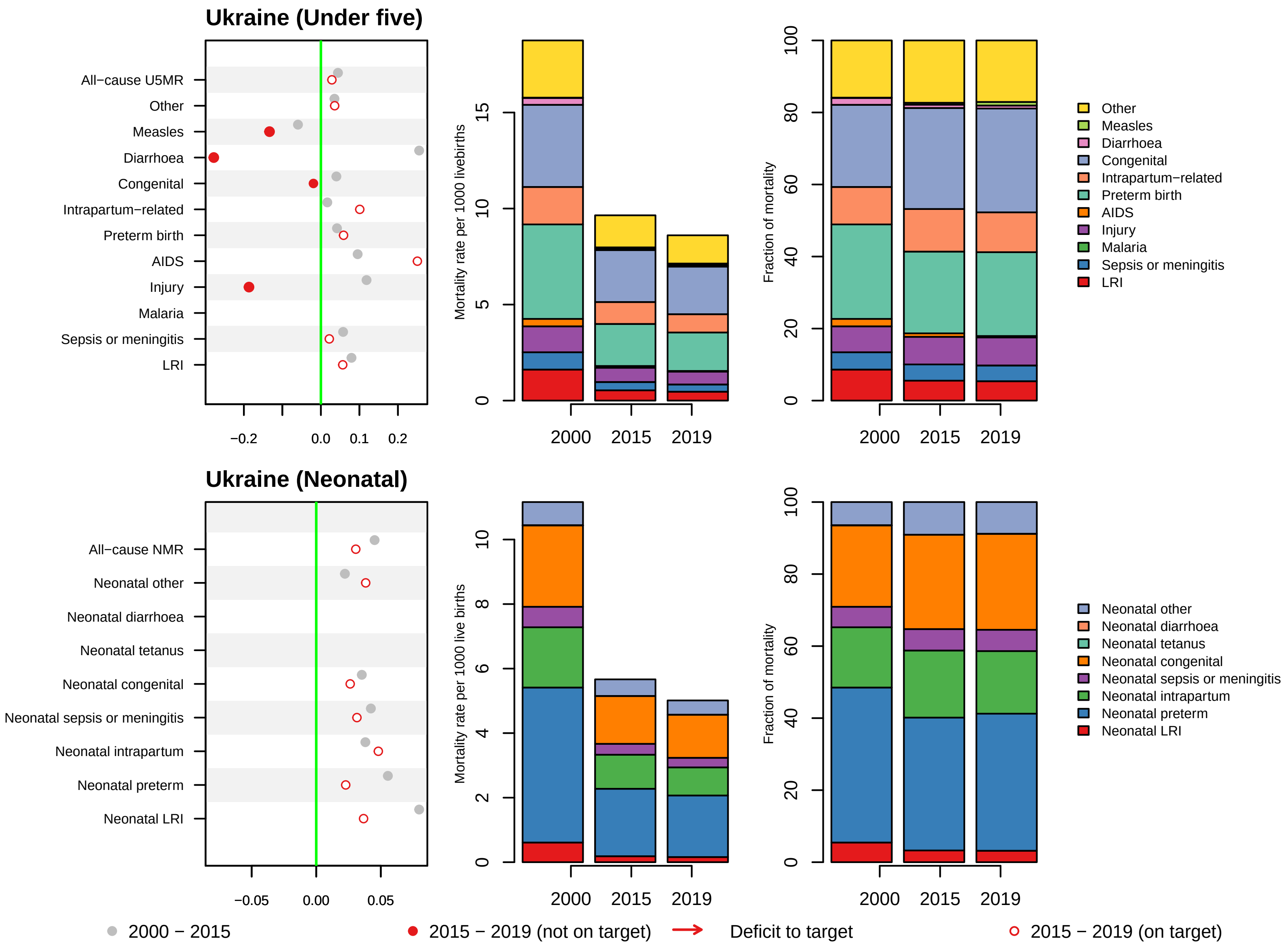

Ukraine (Under five)
All−cause U5MR
Other
Measles
Diarrhoea
Congenital
Intrapartum−related
Preterm birth
AIDS
Injury
Malaria
Sepsis or meningitis
LRI
−0.2
0.0
0.1
0.2
Mortality rate per 1000 livebirths
Fraction of mortality
2000
2015
2019
Other
Measles
Diarrhoea
Congenital
Intrapartum−related
Preterm birth
AIDS
Injury
Malaria
Sepsis or meningitis
LRI
Ukraine (Neonatal)
All−cause NMR
Neonatal other
Neonatal diarrhoea
Neonatal tetanus
Neonatal congenital
Neonatal sepsis or meningitis
Neonatal intrapartum
Neonatal preterm
Neonatal LRI
−0.05
0.00
0.05
Mortality rate per 1000 live births
Fraction of mortality
Neonatal other
Neonatal diarrhoea
Neonatal tetanus
Neonatal congenital
Neonatal sepsis or meningitis
Neonatal intrapartum
Neonatal preterm
Neonatal LRI
2000 − 2015
2015 − 2019 (not on target)
Deficit to target
2015 − 2019 (on target)

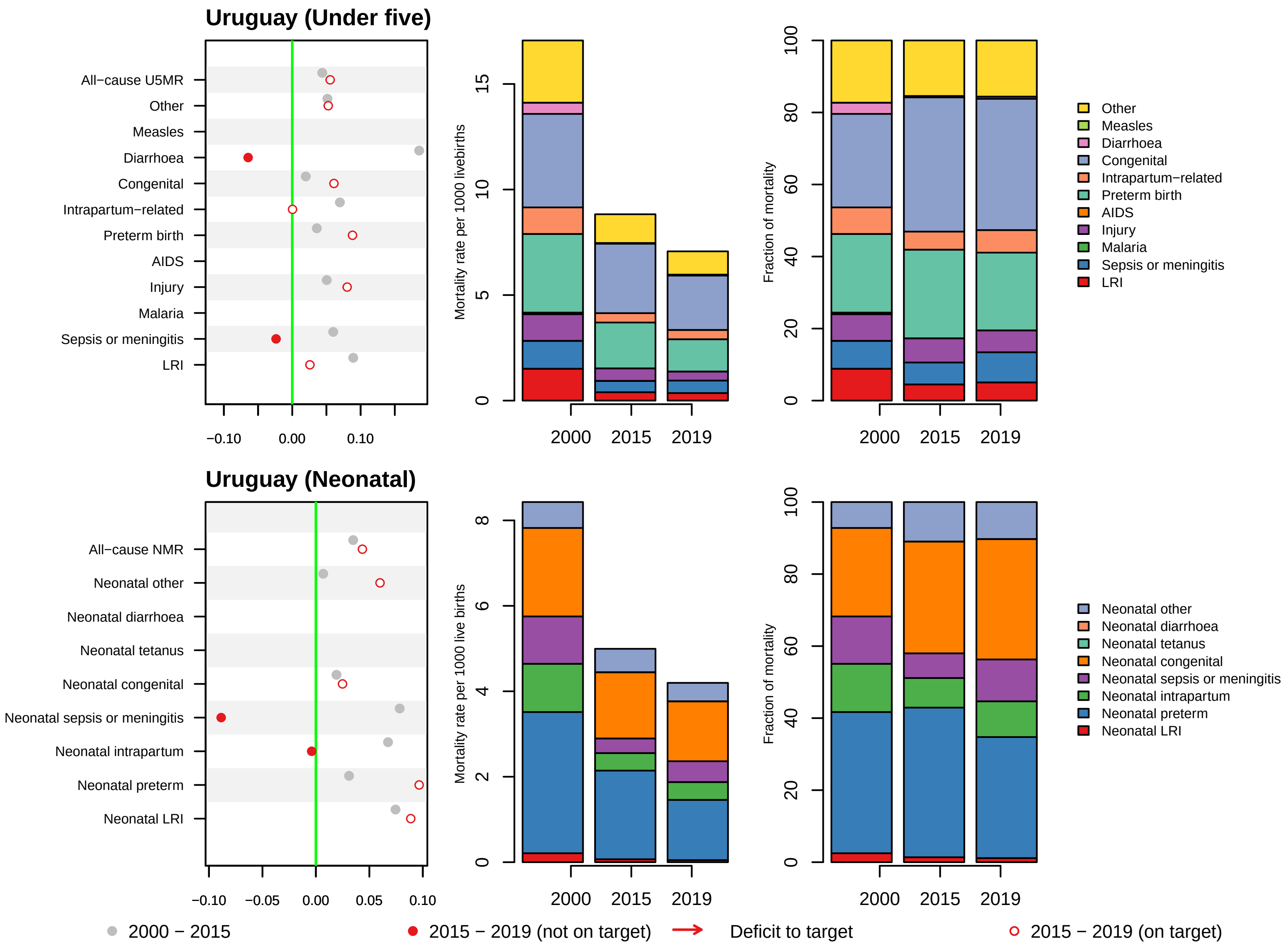

Uruguay (Under five)
All−cause U5MR
Other
Measles
Diarrhoea
Congenital
Intrapartum−related
Preterm birth
AIDS
Injury
Malaria
Sepsis or meningitis
LRI
−0.10
0.00
0.10
Mortality rate per 1000 livebirths
Fraction of mortality
2000
2015
2019
Other
Measles
Diarrhoea
Congenital
Intrapartum−related
Preterm birth
AIDS
Injury
Malaria
Sepsis or meningitis
LRI
Uruguay (Neonatal)
All−cause NMR
Neonatal other
Neonatal diarrhoea
Neonatal tetanus
Neonatal congenital
Neonatal sepsis or meningitis
Neonatal intrapartum
Neonatal preterm
Neonatal LRI
−0.10
−0.05
0.00
0.05
0.10
Mortality rate per 1000 live births
Fraction of mortality
2000
2015
2019
Neonatal other
Neonatal diarrhoea
Neonatal tetanus
Neonatal congenital
Neonatal sepsis or meningitis
Neonatal intrapartum
Neonatal preterm
Neonatal LRI
2000 − 2015
2015 − 2019 (not on target)
Deficit to target
2015 − 2019 (on target)

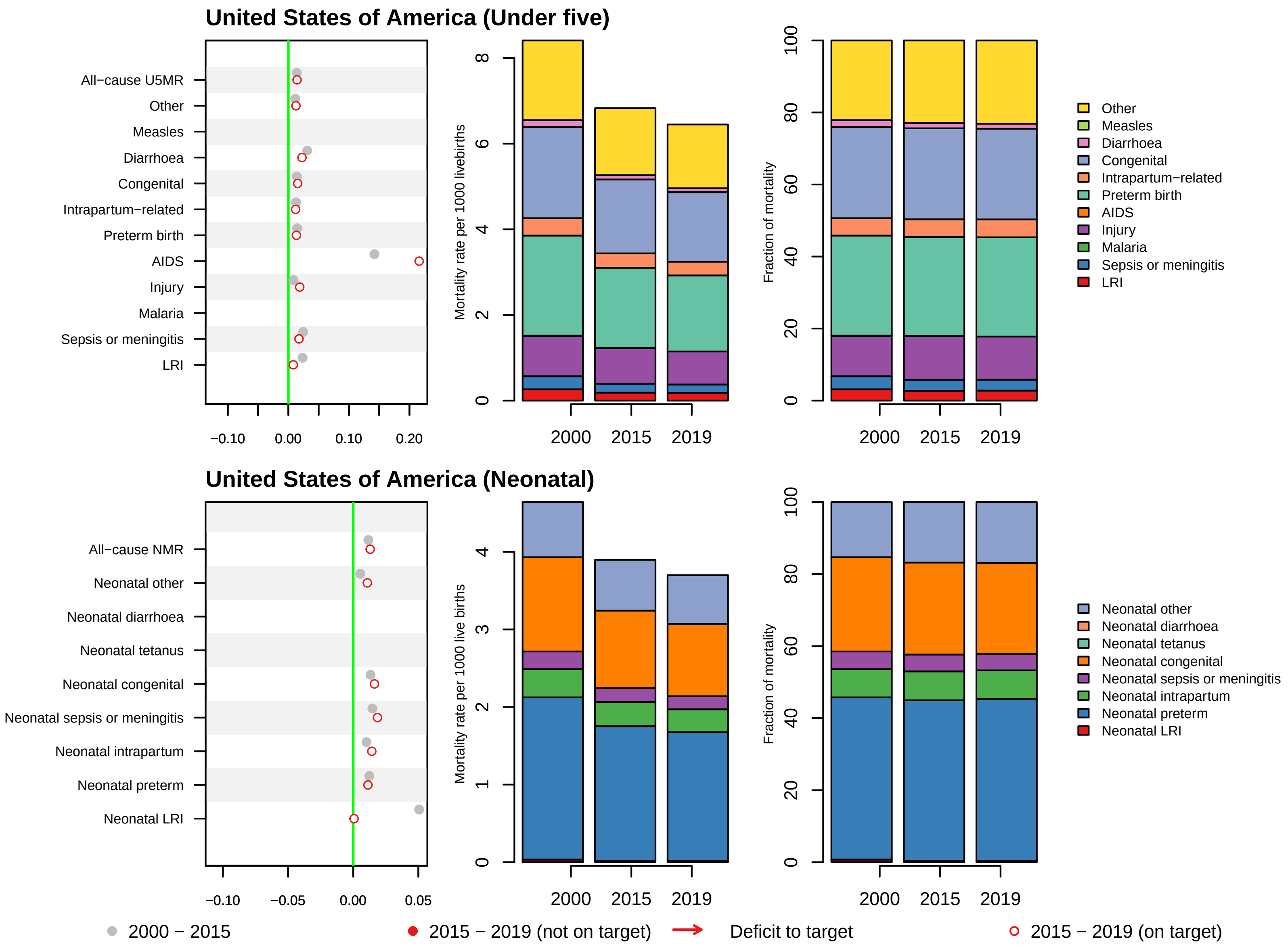

United States of America (Under five)
All−cause U5MR
Other
Measles
Diarrhoea
Congenital
Intrapartum−related
Preterm birth
AIDS
Injury
Malaria
Sepsis or meningitis
LRI
−0.10
0.00
0.10
0.20
Mortality rate per 1000 livebirths
0
2
4
6
8
2000
2015
2019
Fraction of mortality
0
20
40
60
80
100
Other
Measles
Diarrhoea
Congenital
Intrapartum−related
Preterm birth
AIDS
Injury
Malaria
Sepsis or meningitis
LRI
United States of America (Neonatal)
All−cause NMR
Neonatal other
Neonatal diarrhoea
Neonatal tetanus
Neonatal congenital
Neonatal sepsis or meningitis
Neonatal intrapartum
Neonatal preterm
Neonatal LRI
−0.10
−0.05
0.00
0.05
Mortality rate per 1000 live births
0
1
2
3
4
Neonatal other
Neonatal diarrhoea
Neonatal tetanus
Neonatal congenital
Neonatal sepsis or meningitis
Neonatal intrapartum
Neonatal preterm
Neonatal LRI
2000 − 2015
2015 − 2019 (not on target)
Deficit to target
2015 − 2019 (on target)

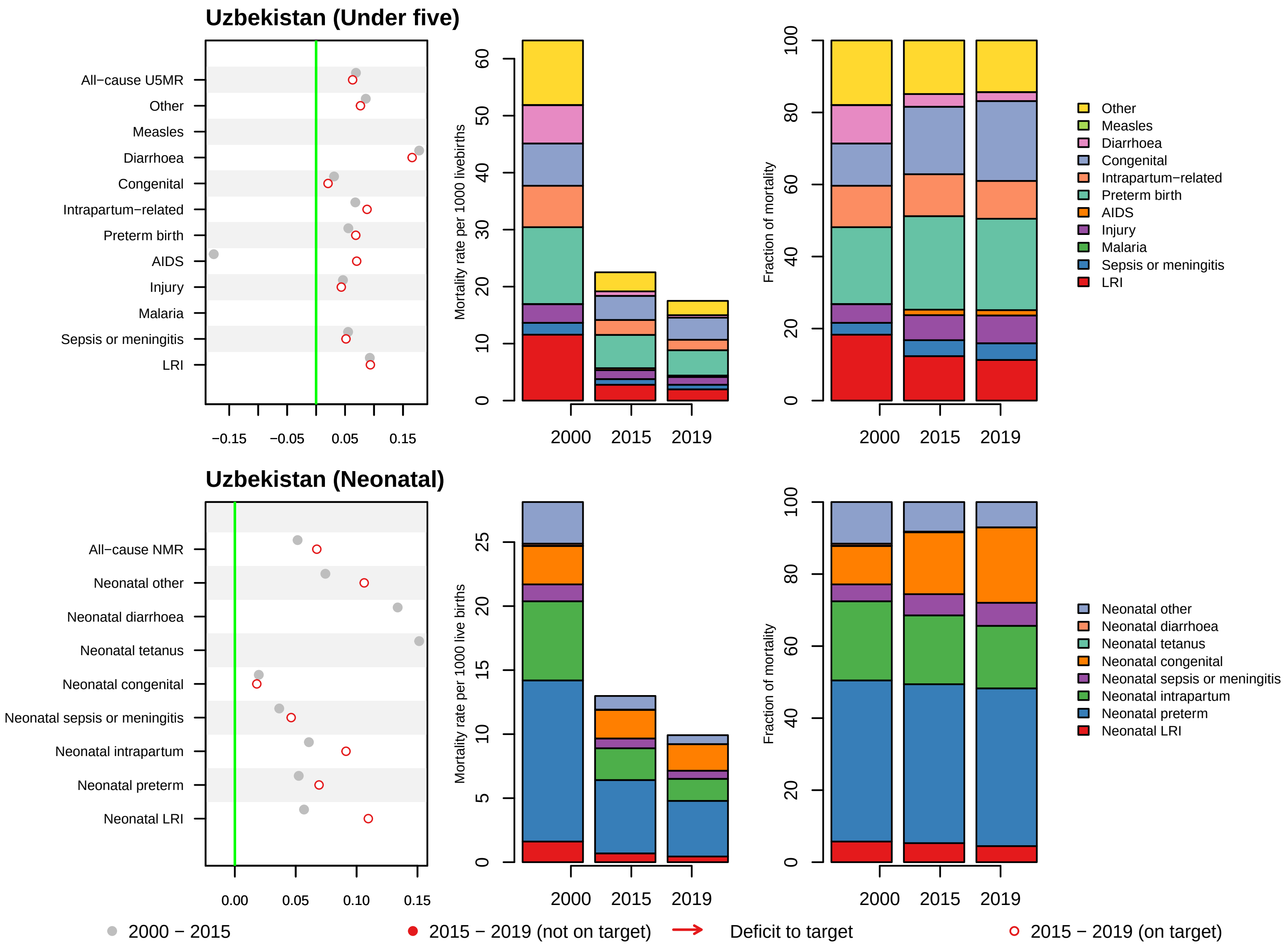

Uzbekistan (Under five)
All−cause U5MR
Other
Measles
Diarrhoea
Congenital
Intrapartum−related
Preterm birth
AIDS
Injury
Malaria
Sepsis or meningitis
LRI
−0.15
−0.05
0.05
0.15
Mortality rate per 1000 livebirths
Fraction of mortality
2000
2015
2019
Uzbekistan (Neonatal)
All−cause NMR
Neonatal other
Neonatal diarrhoea
Neonatal tetanus
Neonatal congenital
Neonatal sepsis or meningitis
Neonatal intrapartum
Neonatal preterm
Neonatal LRI
0.00
0.05
0.10
0.15
Mortality rate per 1000 live births
2000 − 2015
2015 − 2019 (not on target)
Deficit to target
2015 − 2019 (on target)

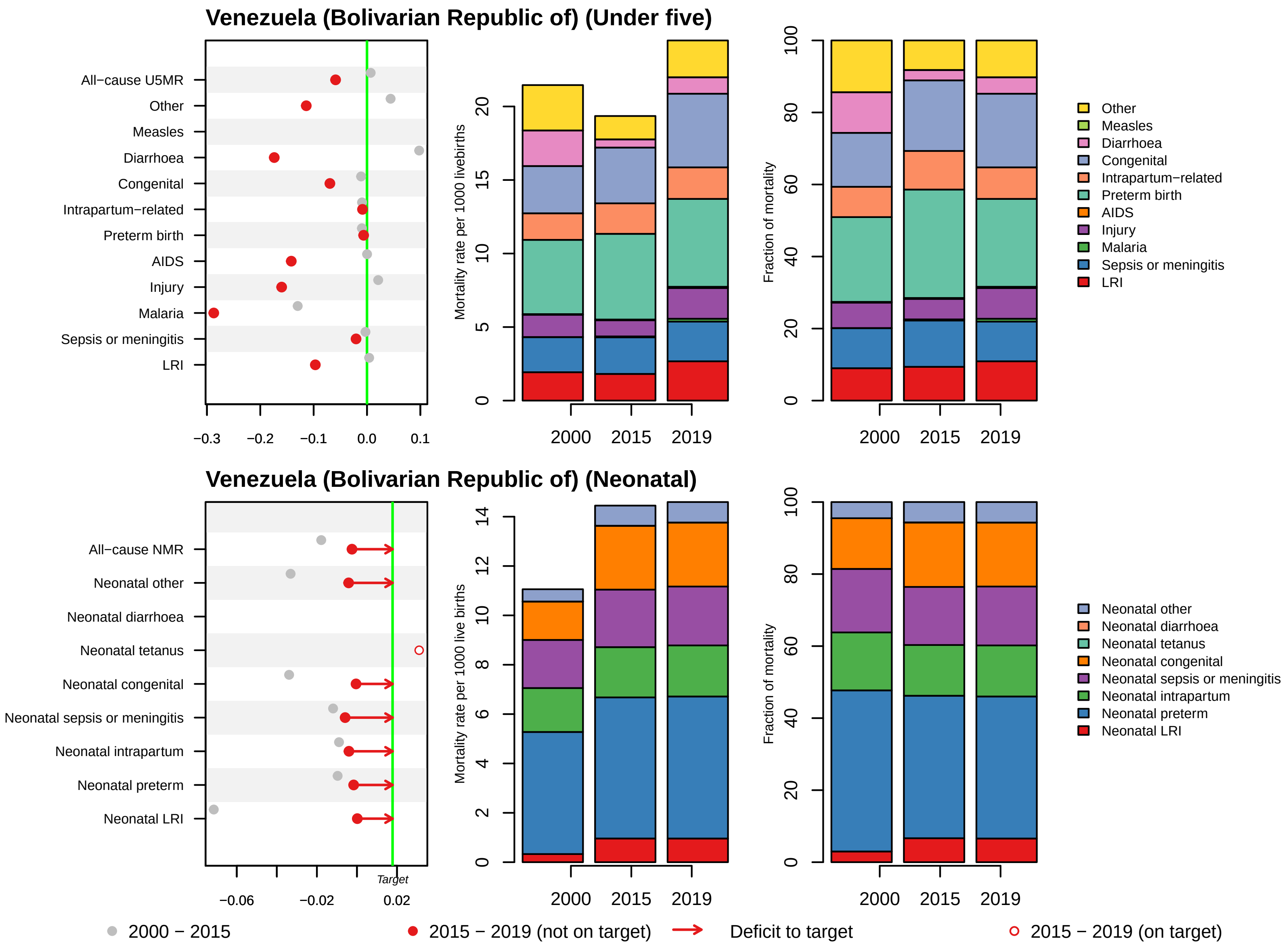

Venezuela (Bolivarian Republic of) (Under five)
All−cause U5MR
Other
Measles
Diarrhoea
Congenital
Intrapartum−related
Preterm birth
AIDS
Injury
Malaria
Sepsis or meningitis
LRI
−0.3 −0.2 −0.1 0.0 0.1
Mortality rate per 1000 livebirths
Fraction of mortality
2000 2015 2019
Other
Measles
Diarrhoea
Congenital
Intrapartum−related
Preterm birth
AIDS
Injury
Malaria
Sepsis or meningitis
LRI
Venezuela (Bolivarian Republic of) (Neonatal)
All−cause NMR
Neonatal other
Neonatal diarrhoea
Neonatal tetanus
Neonatal congenital
Neonatal sepsis or meningitis
Neonatal intrapartum
Neonatal preterm
Neonatal LRI
Target
−0.06 −0.02 0.02
Mortality rate per 1000 live births
Fraction of mortality
2000 2015 2019
Neonatal other
Neonatal diarrhoea
Neonatal tetanus
Neonatal congenital
Neonatal sepsis or meningitis
Neonatal intrapartum
Neonatal preterm
Neonatal LRI
2000 − 2015
2015 − 2019 (not on target)
Deficit to target
2015 − 2019 (on target)

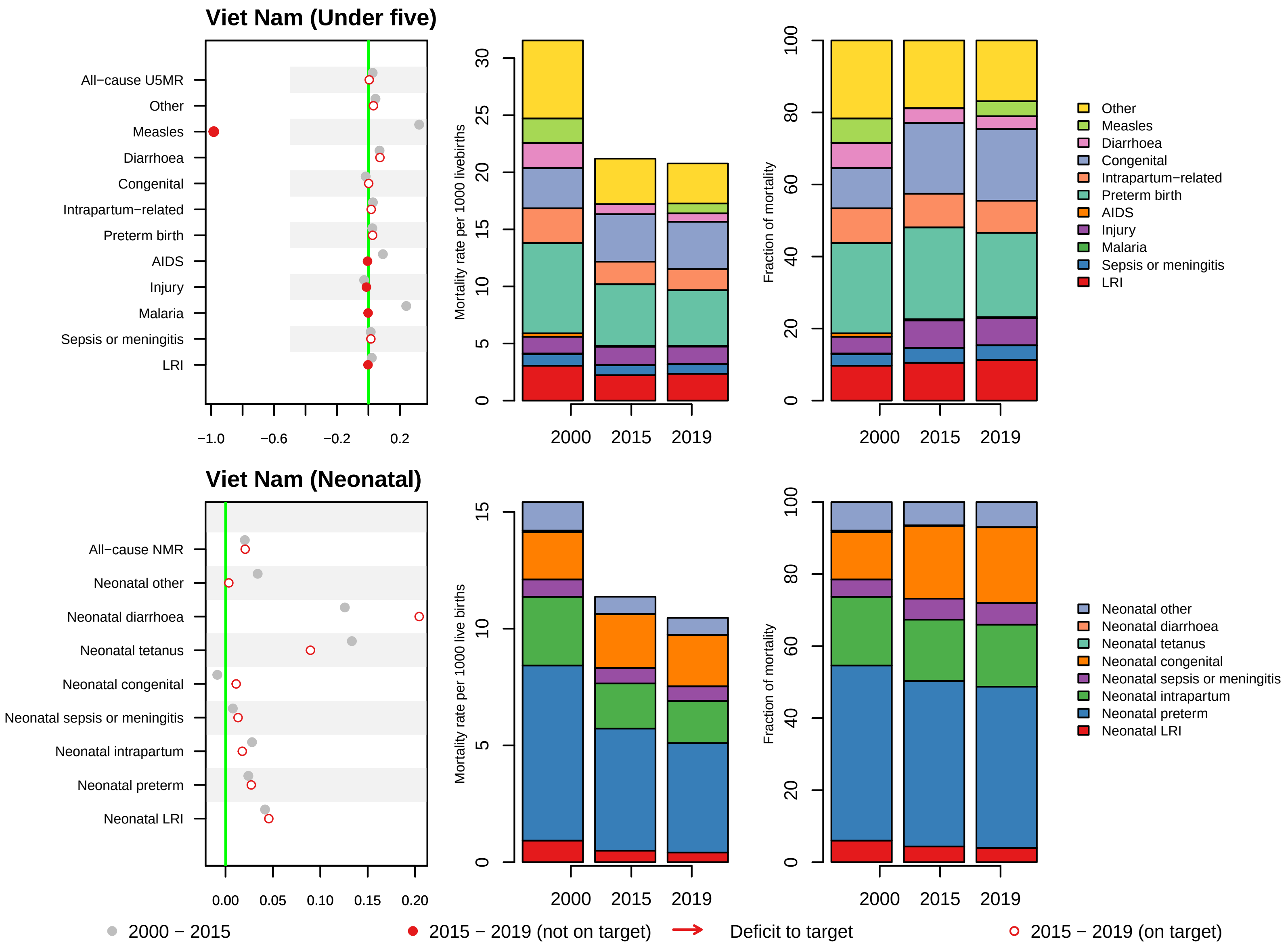

Viet Nam (Under five)
All−cause U5MR
Other
Measles
Diarrhoea
Congenital
Intrapartum−related
Preterm birth
AIDS
Injury
Malaria
Sepsis or meningitis
LRI
−1.0
−0.6
−0.2
0.2
Mortality rate per 1000 livebirths
Fraction of mortality
2000
2015
2019
Viet Nam (Neonatal)
All−cause NMR
Neonatal other
Neonatal diarrhoea
Neonatal tetanus
Neonatal congenital
Neonatal sepsis or meningitis
Neonatal intrapartum
Neonatal preterm
Neonatal LRI
0.00
0.05
0.10
0.15
0.20
Mortality rate per 1000 live births
2000 − 2015
2015 − 2019 (not on target)
Deficit to target
2015 − 2019 (on target)

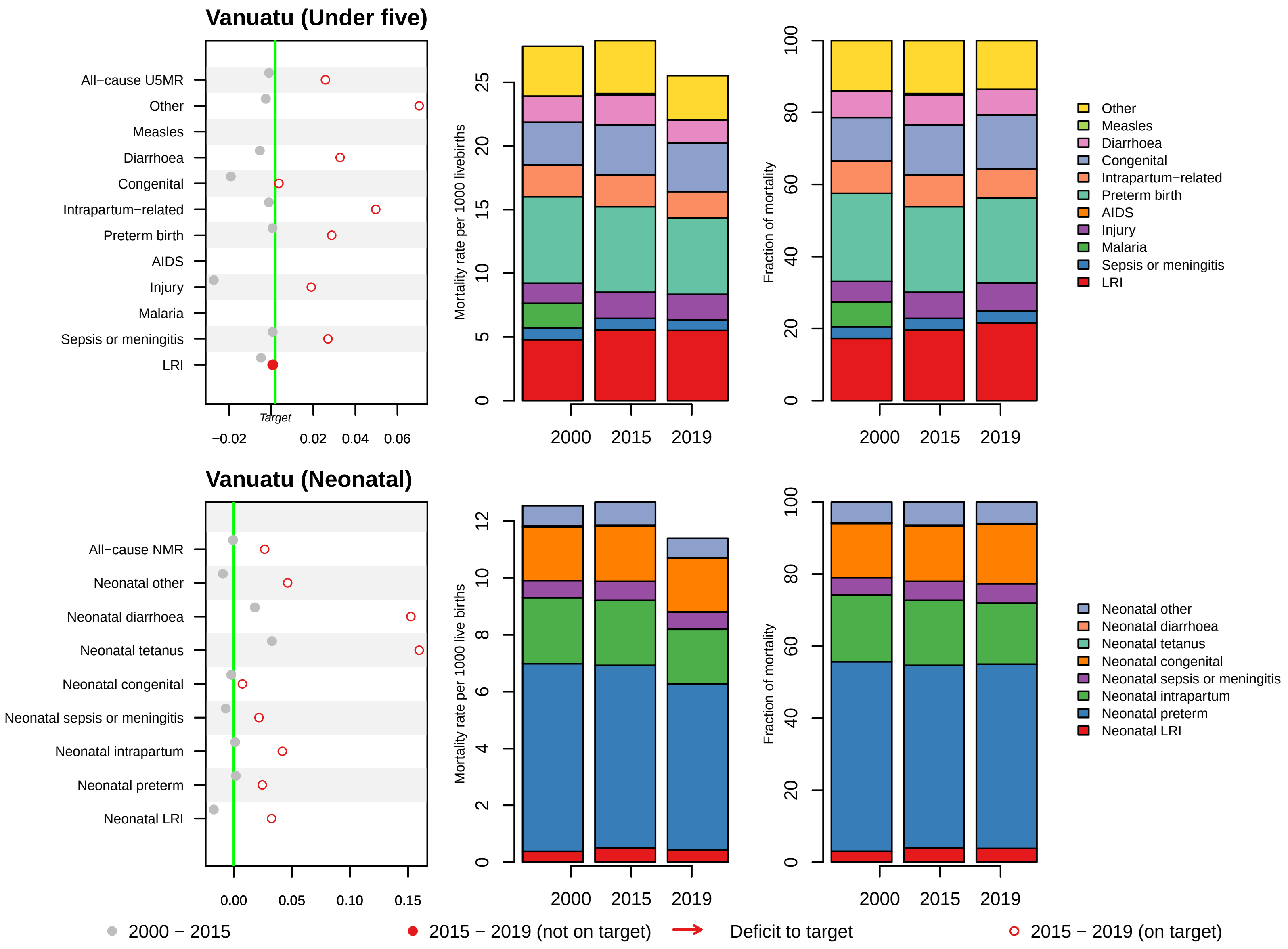
Vanuatu (Under five)
All−cause U5MR
Other
Measles
Diarrhoea
Congenital
Intrapartum−related
Preterm birth
AIDS
Injury
Malaria
Sepsis or meningitis
LRI
Target
−0.02
0.02
0.04
0.06
Mortality rate per 1000 livebirths
Fraction of mortality
2000
2015
2019
Other
Measles
Diarrhoea
Congenital
Intrapartum−related
Preterm birth
AIDS
Injury
Malaria
Sepsis or meningitis
LRI
Vanuatu (Neonatal)
All−cause NMR
Neonatal other
Neonatal diarrhoea
Neonatal tetanus
Neonatal congenital
Neonatal sepsis or meningitis
Neonatal intrapartum
Neonatal preterm
Neonatal LRI
0.00
0.05
0.10
0.15
Mortality rate per 1000 live births
Fraction of mortality
2000
2015
2019
Neonatal other
Neonatal diarrhoea
Neonatal tetanus
Neonatal congenital
Neonatal sepsis or meningitis
Neonatal intrapartum
Neonatal preterm
Neonatal LRI
2000 − 2015
2015 − 2019 (not on target)
Deficit to target
2015 − 2019 (on target)

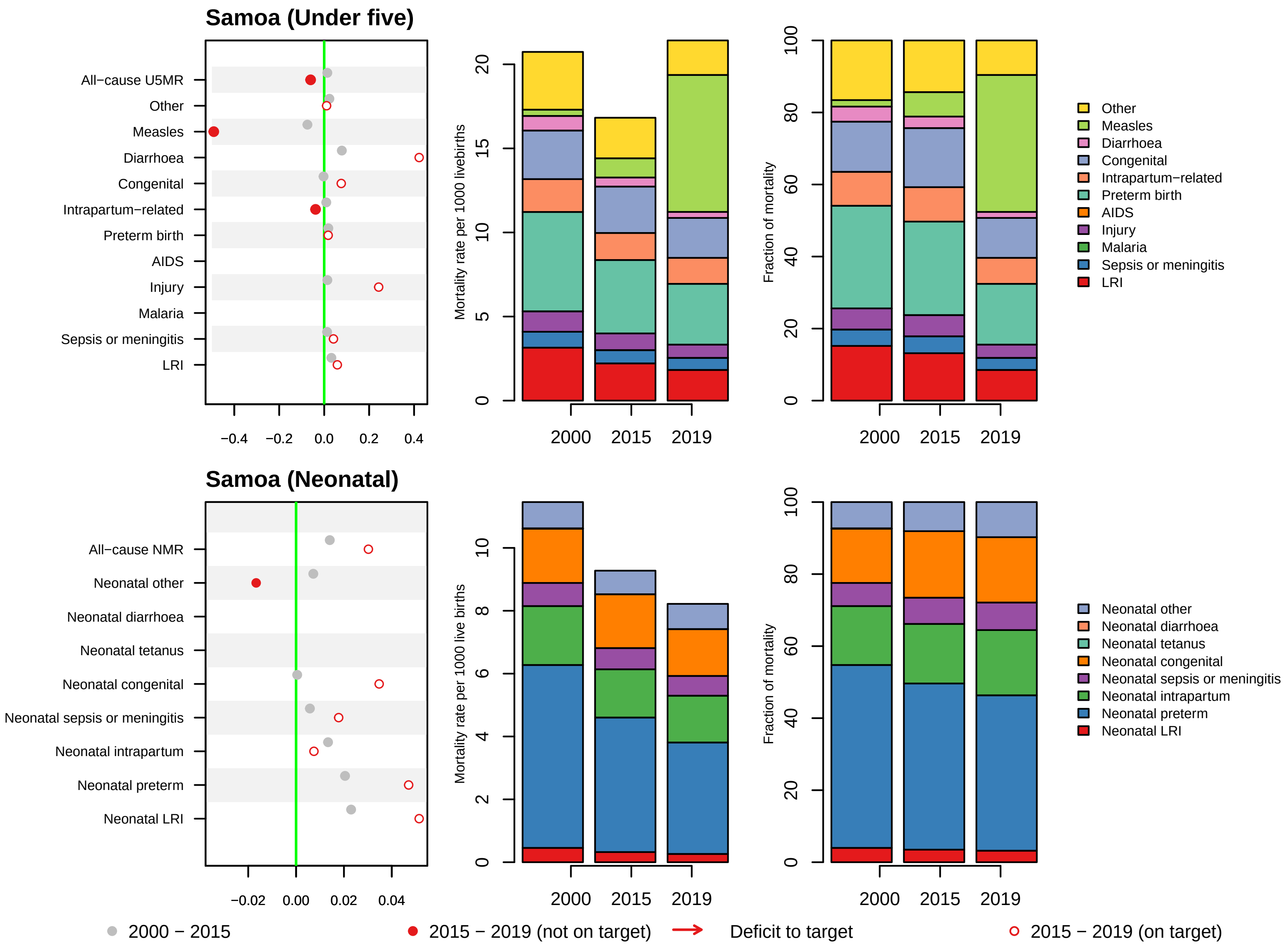
Samoa (Under five)
All−cause U5MR
Other
Measles
Diarrhoea
Congenital
Intrapartum−related
Preterm birth
AIDS
Injury
Malaria
Sepsis or meningitis
LRI
−0.4
−0.2
0.0
0.2
0.4
Mortality rate per 1000 livebirths
0
5
10
15
20
2000
2015
2019
Fraction of mortality
0
20
40
60
80
100
2000
2015
2019
Other
Measles
Diarrhoea
Congenital
Intrapartum−related
Preterm birth
AIDS
Injury
Malaria
Sepsis or meningitis
LRI
Samoa (Neonatal)
All−cause NMR
Neonatal other
Neonatal diarrhoea
Neonatal tetanus
Neonatal congenital
Neonatal sepsis or meningitis
Neonatal intrapartum
Neonatal preterm
Neonatal LRI
−0.02
0.00
0.02
0.04
Mortality rate per 1000 live births
0
2
4
6
8
10
2000
2015
2019
Fraction of mortality
0
20
40
60
80
100
2000
2015
2019
Neonatal other
Neonatal diarrhoea
Neonatal tetanus
Neonatal congenital
Neonatal sepsis or meningitis
Neonatal intrapartum
Neonatal preterm
Neonatal LRI
2000 − 2015
2015 − 2019 (not on target)
Deficit to target
2015 − 2019 (on target)

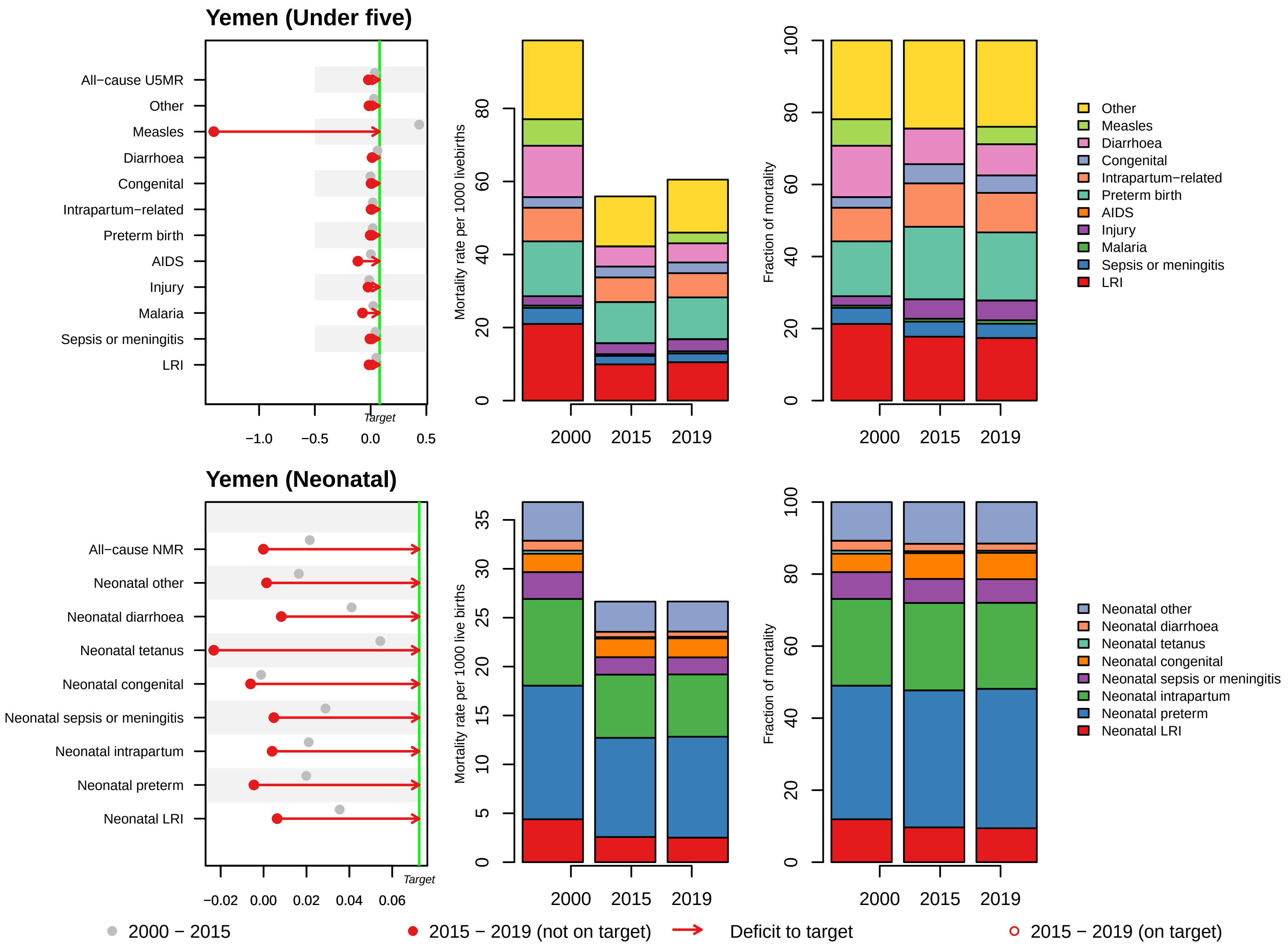

Yemen (Under five)
All−cause U5MR
Other
Measles
Diarrhoea
Congenital
Intrapartum−related
Preterm birth
AIDS
Injury
Malaria
Sepsis or meningitis
LRI
Target
−1.0
−0.5
0.0
0.5
Mortality rate per 1000 livebirths
Fraction of mortality
2000
2015
2019
Yemen (Neonatal)
All−cause NMR
Neonatal other
Neonatal diarrhoea
Neonatal tetanus
Neonatal congenital
Neonatal sepsis or meningitis
Neonatal intrapartum
Neonatal preterm
Neonatal LRI
−0.02
0.00
0.02
0.04
0.06
Mortality rate per 1000 live births
2000 − 2015
2015 − 2019 (not on target)
Deficit to target
2015 − 2019 (on target)

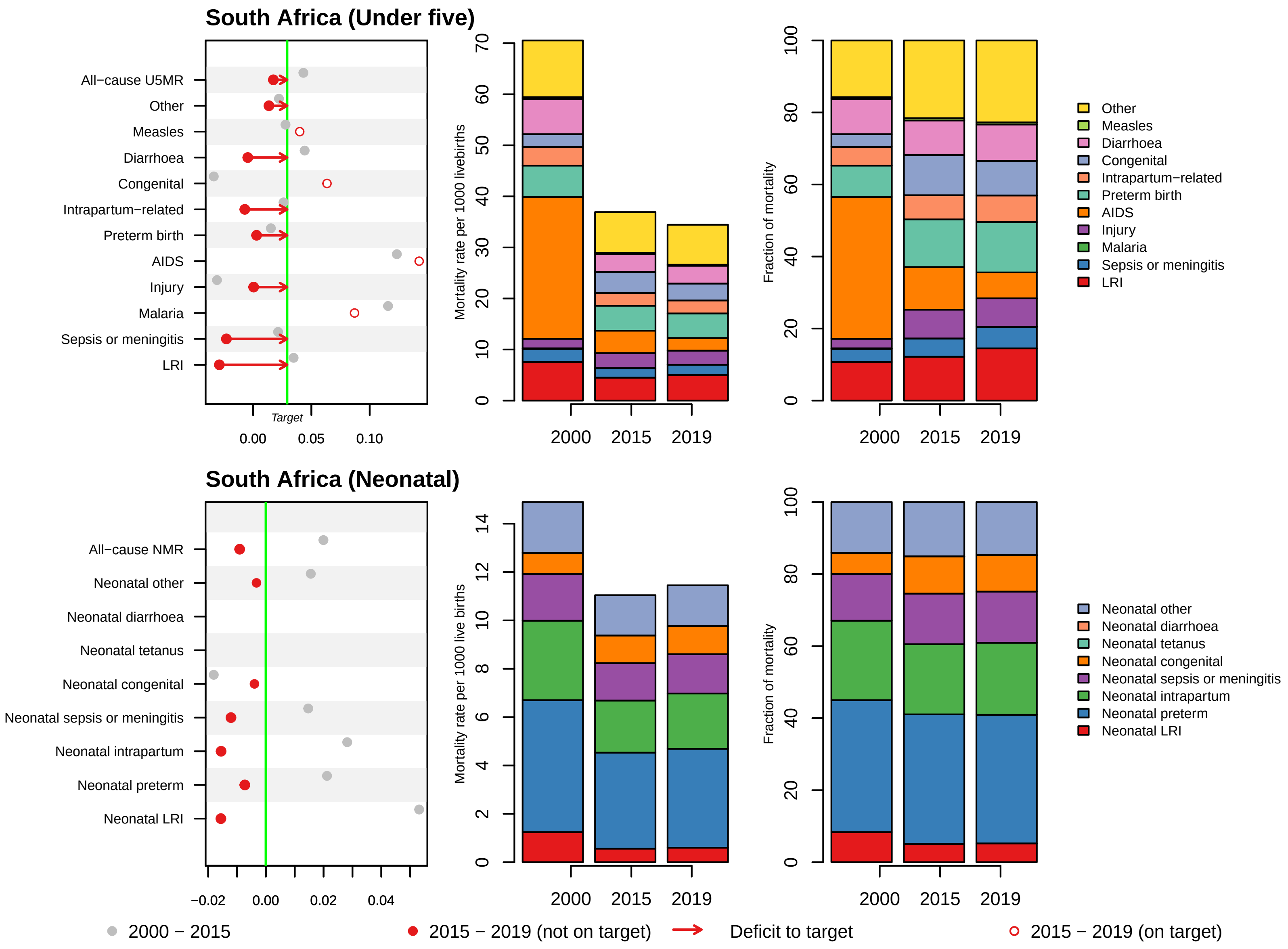
South Africa (Under five)
All−cause U5MR
Other
Measles
Diarrhoea
Congenital
Intrapartum−related
Preterm birth
AIDS
Injury
Malaria
Sepsis or meningitis
LRI
Target
0.00
0.05
0.10
Mortality rate per 1000 livebirths
2000
2015
2019
Fraction of mortality
Other
Measles
Diarrhoea
Congenital
Intrapartum−related
Preterm birth
AIDS
Injury
Malaria
Sepsis or meningitis
LRI
South Africa (Neonatal)
All−cause NMR
Neonatal other
Neonatal diarrhoea
Neonatal tetanus
Neonatal congenital
Neonatal sepsis or meningitis
Neonatal intrapartum
Neonatal preterm
Neonatal LRI
−0.02
0.00
0.02
0.04
Mortality rate per 1000 live births
2000
2015
2019
Fraction of mortality
Neonatal other
Neonatal diarrhoea
Neonatal tetanus
Neonatal congenital
Neonatal sepsis or meningitis
Neonatal intrapartum
Neonatal preterm
Neonatal LRI
2000 − 2015
2015 − 2019 (not on target)
Deficit to target
2015 − 2019 (on target)

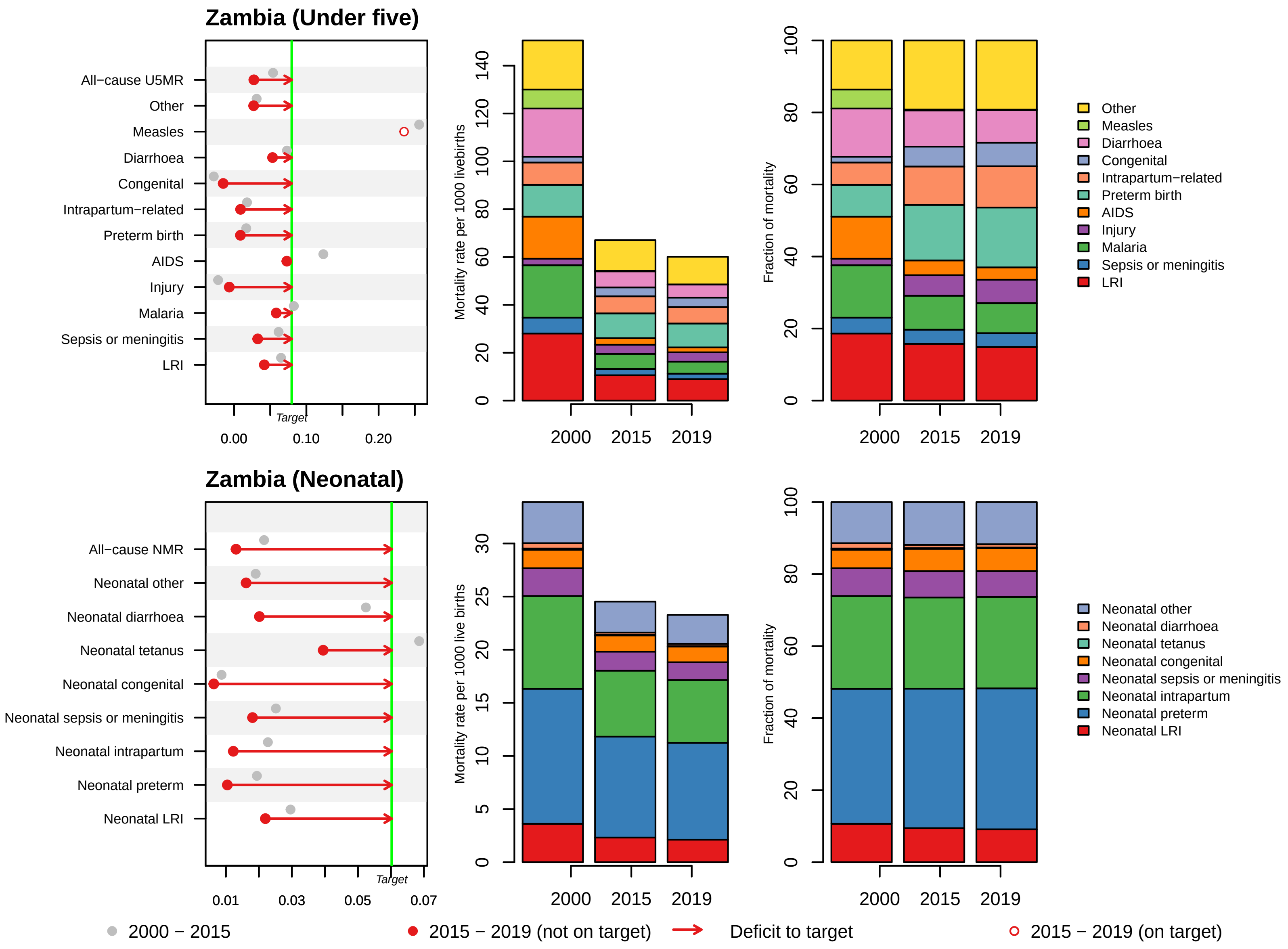

Zambia (Under five)
All−cause U5MR
Other
Measles
Diarrhoea
Congenital
Intrapartum−related
Preterm birth
AIDS
Injury
Malaria
Sepsis or meningitis
LRI
Target
0.00
0.10
0.20
Mortality rate per 1000 livebirths
Fraction of mortality
2000
2015
2019
Other
Measles
Diarrhoea
Congenital
Intrapartum−related
Preterm birth
AIDS
Injury
Malaria
Sepsis or meningitis
LRI
Zambia (Neonatal)
All−cause NMR
Neonatal other
Neonatal diarrhoea
Neonatal tetanus
Neonatal congenital
Neonatal sepsis or meningitis
Neonatal intrapartum
Neonatal preterm
Neonatal LRI
0.01
0.03
0.05
0.07
Mortality rate per 1000 live births
Neonatal other
Neonatal diarrhoea
Neonatal tetanus
Neonatal congenital
Neonatal sepsis or meningitis
Neonatal intrapartum
Neonatal preterm
Neonatal LRI
2000 − 2015
2015 − 2019 (not on target)
Deficit to target
2015 − 2019 (on target)

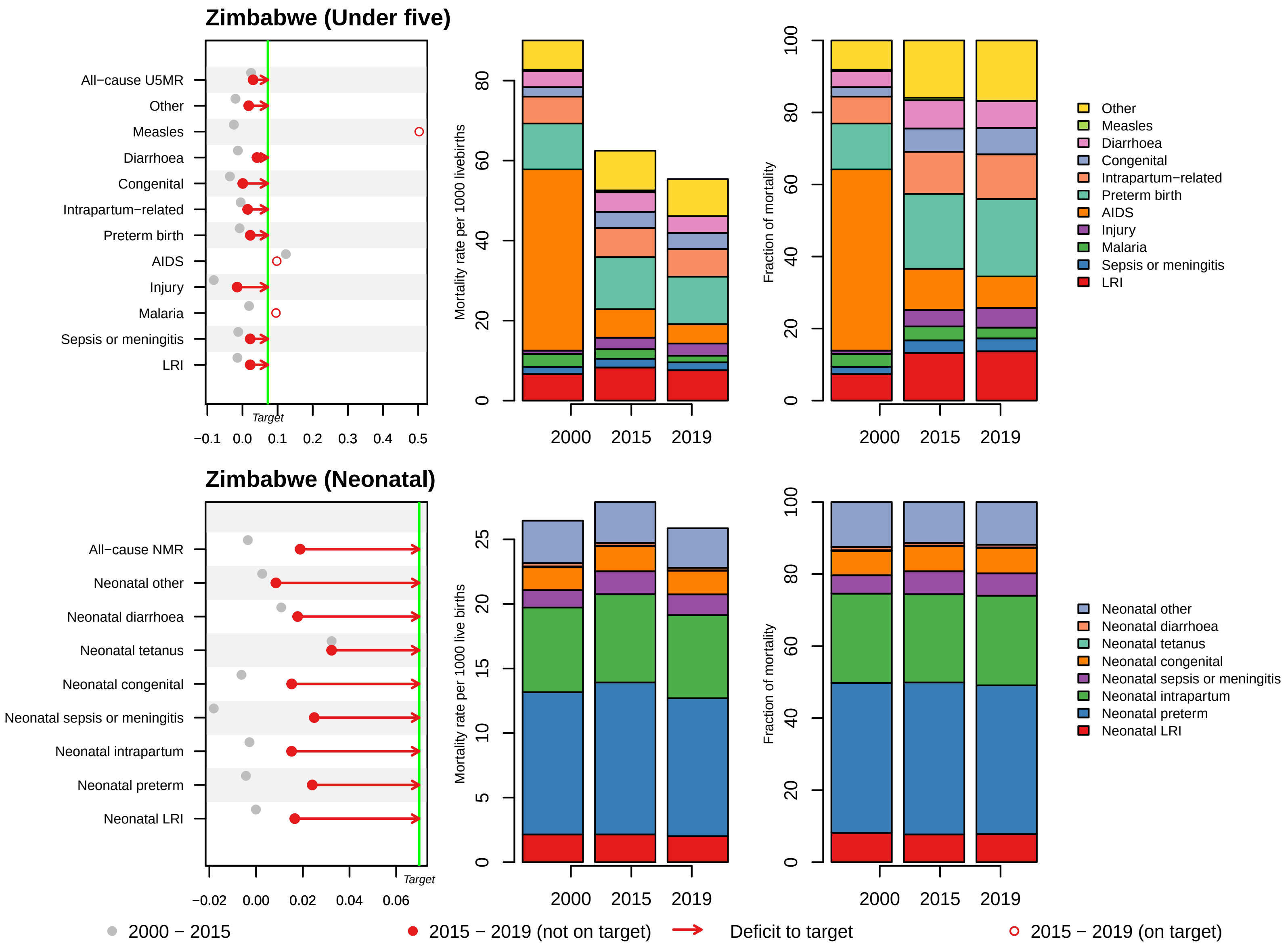

Zimbabwe (Under five)
All−cause U5MR
Other
Measles
Diarrhoea
Congenital
Intrapartum−related
Preterm birth
AIDS
Injury
Malaria
Sepsis or meningitis
LRI
Target
−0.1 0.0 0.1 0.2 0.3 0.4 0.5
Mortality rate per 1000 livebirths
0 20 40 60 80
2000 2015 2019
Fraction of mortality
0 20 40 60 80 100
2000 2015 2019
Other
Measles
Diarrhoea
Congenital
Intrapartum−related
Preterm birth
AIDS
Injury
Malaria
Sepsis or meningitis
LRI
Zimbabwe (Neonatal)
All−cause NMR
Neonatal other
Neonatal diarrhoea
Neonatal tetanus
Neonatal congenital
Neonatal sepsis or meningitis
Neonatal intrapartum
Neonatal preterm
Neonatal LRI
Target
−0.02 0.00 0.02 0.04 0.06
Mortality rate per 1000 live births
0 5 10 15 20 25
2000 2015 2019
Fraction of mortality
0 20 40 60 80 100
2000 2015 2019
Neonatal other
Neonatal diarrhoea
Neonatal tetanus
Neonatal congenital
Neonatal sepsis or meningitis
Neonatal intrapartum
Neonatal preterm
Neonatal LRI
2000 − 2015
2015 − 2019 (not on target)
Deficit to target
2015 − 2019 (on target)
